# Understanding the Regiodivergence between Hydroarylation and Trifluoromethylarylation of 1,3-Dienes Using Anilines in HFIP

**DOI:** 10.1021/jacsau.4c00162

**Published:** 2024-05-10

**Authors:** Carlos Corral Suarez, Israel Fernández, Ignacio Colomer

**Affiliations:** † Instituto de Química Orgánica General (IQOG-CSIC), Juan de la Cierva 3, 28006 Madrid, Spain; ‡ Departamento de Química Orgánica and Centro de Innovación en Química Avanzada (ORFEO−CINQA), Facultad de Ciencias Químicas, Universidad Complutense de Madrid, Ciudad Universitaria, 28040 Madrid, Spain

**Keywords:** arylation, trifluoromethyl, diene, aniline, regiodivergence

## Abstract

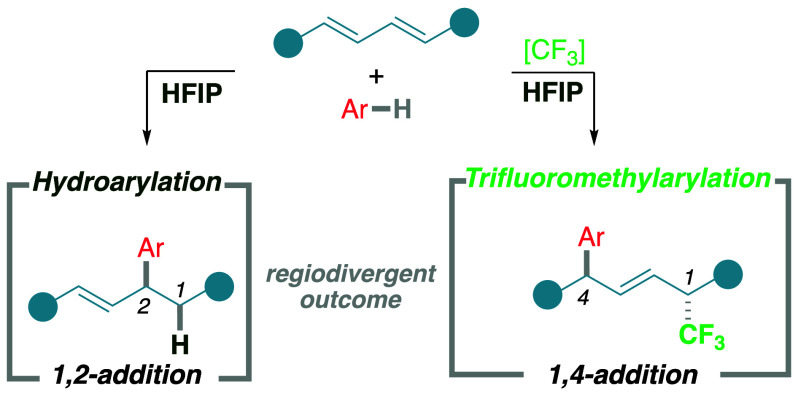

Conjugated dienes (1,3-dienes) are versatile and valuable chemical feedstocks that can be used as two-carbon or four-carbon synthons with vast applications across the chemical industry. However, the main challenge for their productive incorporation in synthetic routes is their chemo-, regio-, and stereoselective functionalization. Herein, we introduce a unified strategy for the 1,2-hydroarylation and 1,4-trifluoromethylarylation of 1,3-dienes using anilines in hexafluoroisopropanol. DFT calculations point toward a kinetically controlled process in both transformations, particularly in the trifluoromethylarylation, to explain the regiodivergent outcome. In addition, we perform an extensive program of functionalization and diversification of the products obtained, including hydrogenation, oxidation, cyclizations, or cross-coupling reactions, that allows access to a library of high-value species in a straightforward manner.

Olefins are one of the most abundant species within chemical feedstock, such that ethylene and propylene are the basic building blocks in the chemical industry. Their conjugated diene counterparts (i.e., 1,3-dienes) are versatile and valuable chemical species that can be used as two-carbon or four-carbon synthons; 1,3-butadiene production is in the million metric tons every year and constantly increasing.^1−4^ Although conjugated dienes feature a relatively simple and easily accessible structure, their chemical properties go far beyond those of simple olefins. While their reactivity has been extensively studied and it represents a rich area of research with continuous innovations, mainly using transition metal catalysis, competitive metal-free approaches are still lacking.^5−11^ From all of the possible strategies, the hydrofunctionalization of 1,3-dienes represents the most basic transformation from a chemical structure modification perspective. However, if we focus, for example, on the hydroarylation of conjugated dienes, which has attracted the attention of chemists and represents a versatile tool, there are still clear limitations when it comes to the use of nonprefunctionalized aromatic substrates via C–H functionalization (Scheme 1A). Seminal contributions have used prefunctionalized substrates as the aromatic component, such as boronic esters in a palladium-catalyzed process with modest enantioselectivity.^12,13^ This strategy has been expanded recently using aryl boronic acids under nickel catalysis with excellent enantioselectivities.^14−16^ Connected to this approach, aryl fluorides have been used either using nickel or copper catalysts,^17,18^ a strategy that has been expanded recently to aryl bromides and iodides under nickel catalysis.^19^ Otherwise, a net hydroarylation has been reported via hydroboration of conjugated dienes followed by transmetalation of a palladium-catalyzed aryl bromide oxidative addition.^20^ Alternatively, by using homoallyl tosylates or carbonates under palladium or nickel catalysis, respectively, conjugated dienes are invoked as intermediates via metal hydride species to engage in the hydroarylation reactivity.^21,22^

**Scheme 1:**
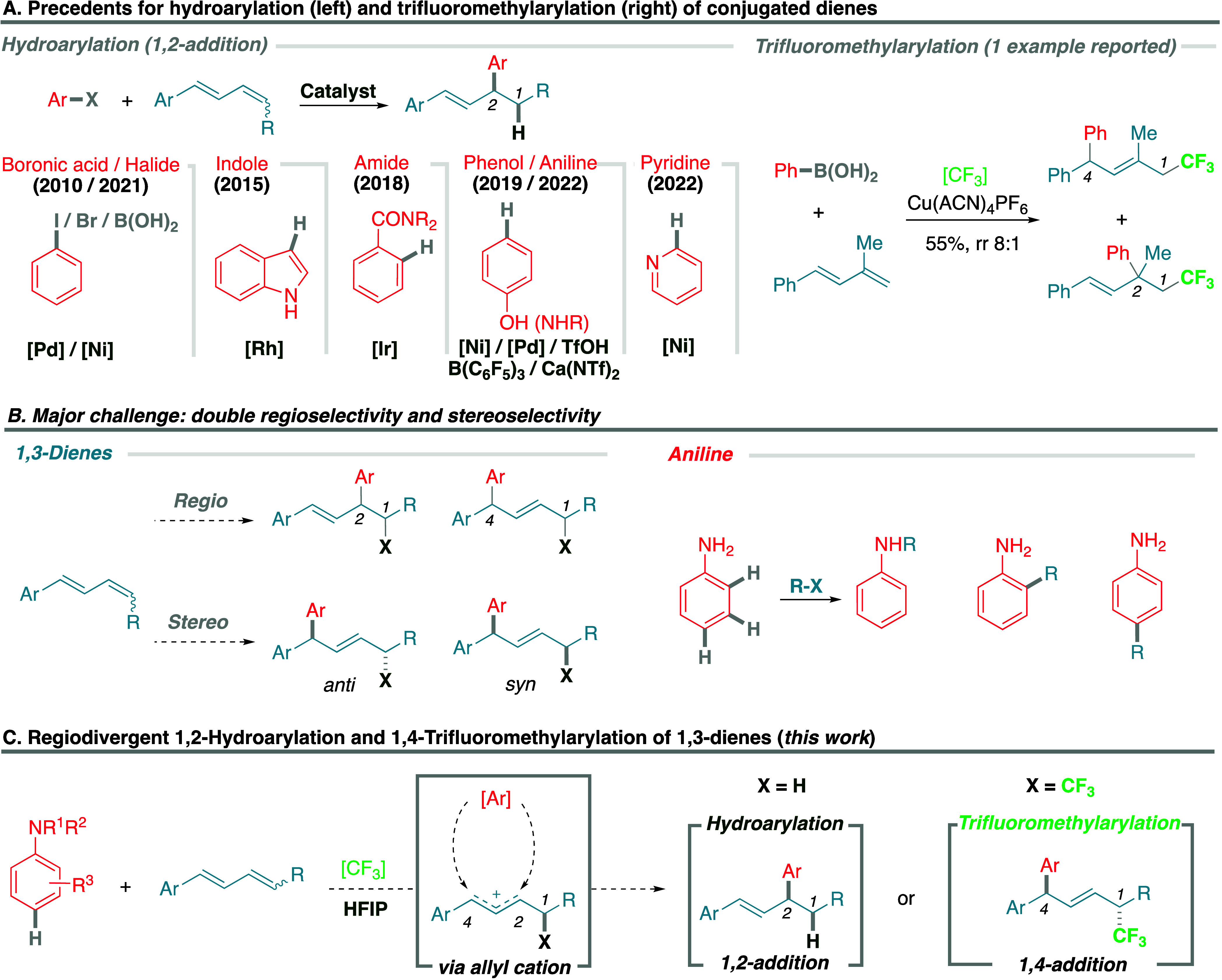
Hydroarylation and Trifluoromethylarylation of 1,3-Dienes: (A) Previous Examples for Hydroarylation and a Unique Example of Trifluoromethylarylation, (B) the Main Challenges of Regioselectivity and Stereoselectivity, and (C) This Work’s Unified Strategy for 1,2-Hydroarylation and 1,4-Trifluoromethylarylation Using Anilines in HFIP

As a step forward, one of the first examples of nonprefunctionalized aryl substrates relied on indoles using rhodium complexes with carbodicarbene ligands; it was first used in a racemic protocol and, later on, extended to its enantioselective version.^23−25^ Soon after, benzamides were incorporated in a C–H activation process triggered by an iridium catalyst where the amide serves as a directing group.^26^ Recently, phenols engaged in this transformation in a process catalyzed by palladium or nickel complexes, triflic acid, or triarylboranes.^27−30^ The latter approach was expanded to diarylamines^31^ that also work under Ca(II)/hexafluoroisopropanol (HFIP) conditions.^32^ A very recent contribution incorporates pyridines in an enantioselective catalytic transformation on the basis of a groundbreaking Ni–Al bimetallic system.^33^ It is important to emphasize that, generally speaking, the 1,2-addition product is dominant for the hydroarylation examples reported.^34^ If one looks at expanding the scope of this transformation to more complex structures, the natural extension would be the selective difunctionalization of 1,3-dienes to create two new C–C bonds. Diarylation,^35−37^ vinylarylation,^38^ and alkylarylation^39,40^ have been scarcely approached; however, it is notably surprising that the trifluoromethylarylation of 1,3-dienes still remains elusive with one single isolated example reported in modest yield and regioselectivity using a terminal diene that does not allow for study of the stereoselectivity of the process (Scheme 1A).^41^

Thus, the main challenges regarding the functionalization of conjugated dienes are starting material double bond chemo- and regioselectivity, as well as product stereoselectivity (diastereo-, enantio-, and remaining olefin *E*/*Z* selection (Scheme 1B). This already arduous regioselective process becomes even more difficult if we aim at incorporating nonprefunctionalized aromatic substrates with multiple reactive positions and no directing groups, such as anilines^42^ (Scheme 1B). Within this context, we have recently developed a metal-, photocatalyst-, and additive-free protocol for the hydroarylation^43^ and trifluoromethylarylation^44^ of simple olefins using anilines as aromatic components and HFIP as a unique solvent.^45,46^ This prompted us to evaluate the behavior of conjugated dienes under our newly developed conditions to find a regiodivergent^47,48^ outcome between hydroarylation and trifluoromethylarylation (Scheme 1C). Both transformations take place through an allylic carbocation, and computational studies help to determine the origin of the opposite regiochemistry.

Considering first the hydroarylation manifold and using model substrates *N*-benzyl aniline **1a** and diene **2a** under previously described conditions for nonconjugated alkenes (HFIP at 80 °C),^43^ we obtained the hydroarylation product **4a** in 62% yield as a single regioisomer via 1,2-addition (Chart 1). In sharp contrast, by next considering the trifluoromethylarylation transformation and using the same substrates **1a** and **2a** under previously optimized conditions for nonconjugated alkenes (with 1.0 equiv of **3** in HFIP at 40 °C),^44^ trifluoromethylarylation product **5a** was obtained in 55% yield as a single regioisomer via 1,4-addition (Chart 1). It is important to emphasize that these transformations work only if HFIP is used as solvent or cosolvent (see the Supporting Information for further screening details). Finally, in both cases, the remaining olefin was isolated as a single *E* diastereoisomer, and the aniline was incorporated exclusively via the *para* position.^49−56^

**Chart 1:**
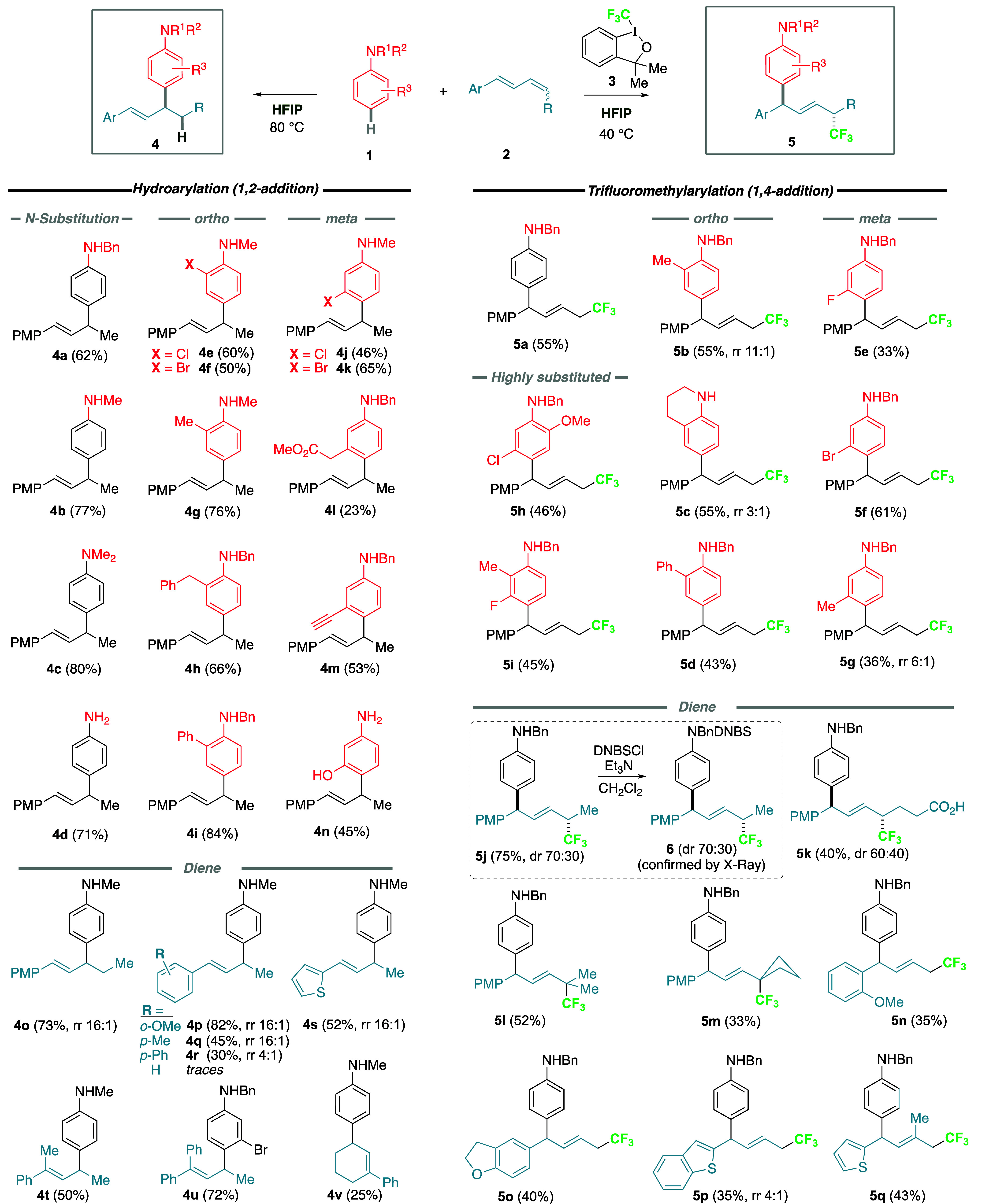
Scope of the Hydroarylation and Trifluoromethylarylation of 1,3-Dienes Using Anilines in HFIPa

a General conditions for hydroarylation: 0.2 mmol of aniline **1** and 0.2–0.4 mmol of alkene **2** in 1.0 mL of HFIP. For trifluoromethylarylation: 0.2 mmol of aniline **1**, 0.2 mmol of alkene **2**, and 0.2 mmol of **3** in 0.5 mL of HFIP. PMP: *para*-methoxyphenyl. DNBSCl: 2,4-dinitrobenzenesulfonyl chloride.

With these interesting results in hand, which point toward a regiodivergent arylation outcome, we evaluated the scope for both transformations (Chart 1). Hydroarylation of model diene **2a** was studied using a broad variety of anilines, which generally afforded the 1,2-addition product as a single regioisomer (in a regioisomeric ratio, rr, > 20:1). Mono- and disubstitution of the amino group (**4a**–**c**), as well as free aniline (**4d**), performed extremely well. Substitution in the *ortho* position of the aromatic ring was well tolerated, including halides (**4e**,**f**), aliphatic (**4g**), benzylic (**4h**), and aromatic (**4i**) groups. Comparably, the *meta* position supported halides (**4j**,**k**), a pendant aliphatic ester (**4l**), alkynyl (**4m**), or heteroatom (**4n**) motifs with good results and as single regioisomers. The diene counterpart was briefly examined and was not limited to terminal dienes (**4o**). Substitution on the aromatic ring was viable, for example, *ortho*-OMe, *para*-Me, and *para*-Ph (**4p**–**r**), or even heteroaromatic, such as thiophene (**4s**). While neutral substrate 1-phenyl-1,3-butadiene gave only traces of product, introducing a Me or Ph substituent in the diene system allowed expansion of the scope using both acyclic (**4t**–**u**) and cyclic dienes (**4v**).

The scope of the trifluoromethylarylation reaction was similarly interrogated. Using model diene **2a**, the *ortho* position of the aniline supports both acyclic (**5b**) and cyclic (**5c**) aliphatic substituents, as well as aromatic (**5d**), though the former two showed a diminished regioselectivity. Supported modifications in the *meta* position include halides (**5e**,**f**) or a methyl group (**5g**); however, the latter suffers from a lower yield and regioselectivity. Finally, substrates bearing multiple substituents with different patterns of substitution and electronic and steric properties complete the scope (**5h**,**i**). Moving on to the scope of the diene, we were particularly interested in using nonterminal dienes, that would allow us to study the diastereoselective outcome of the process. It is important to emphasize that there is only one related example described in the literature using a terminal diene, so the stereochemical properties of this process have not been approached.^41^ Using diene **2b** with a methyl substitution in the diene (R = Me), product **5j** was obtained in high yield and regio- and stereoselective manner (75% yield, dr 70:30). The *anti* relative stereochemistry of the major diasteroisomer was confirmed via X-ray crystallography of a sulfonamide derivative, **6**,^57^ where both diastereoisomers cocrystallized in a 70:30 ratio, which allowed the identification of the major one. A similar result was observed for the analogous example with pendant carboxylic acid **5k**. Not only do terminal and 1,4-disubstituted dienes work, but trisubstituted analogues also allow the accommodation of *gem*-dimethyl or cyclobutyl moieties with all-carbon quaternary centers supporting a CF_3_ group (**5l**,**m**). The diene can accommodate other aromatic groups, such as dihydrobenzofuran or benzothiophene (**5n**–**p**), or substitution in the internal position of the diene (**5q**), to afford in all cases the 1,4-addition product. Limitations in the scope of the diene for both transformations include electrodeficient aromatic or aliphatic substitution (Supporting Information).

The allyl anilines obtained, **4** and **5**, represent new, versatile, and underexplored structures that can be used to access and explore a diverse chemical space (Scheme 2). Further functionalization of the products includes hydrogenation of the remaining olefin to afford saturated aliphatic chain derivative **7** or oxidative cleavage^58^ of the double bond leading to valuable homobenzylic aldehyde **8**. Starting from **4n**, intramolecular hydroetherification and iodoetherification of the remaining alkene gives highly functionalized chromans **9** and **10**, respectively. Moreover, Pd-catalyzed oxidative cyclization^59^ of the same substrate affords interesting amino benzofuran **11**. Alternatively, by starting from **5f**, the aryl bromide functional group could be used as a handle for a wide array of palladium-catalyzed diversifications, including borylation (**12**), Suzuki (**13**), and Heck (**14**) cross-coupling reactions. Selective aniline *ortho* lithiation^60^ followed by Friedel–Craft acyl chloride trapping gives interesting benzophenone derivative **15**. Finally, HF elimination^61^ of the allyl CF_3_ moiety affords the beautiful *gem*-difluoro diene **16** in a challenging transformation.

**Scheme 2:**
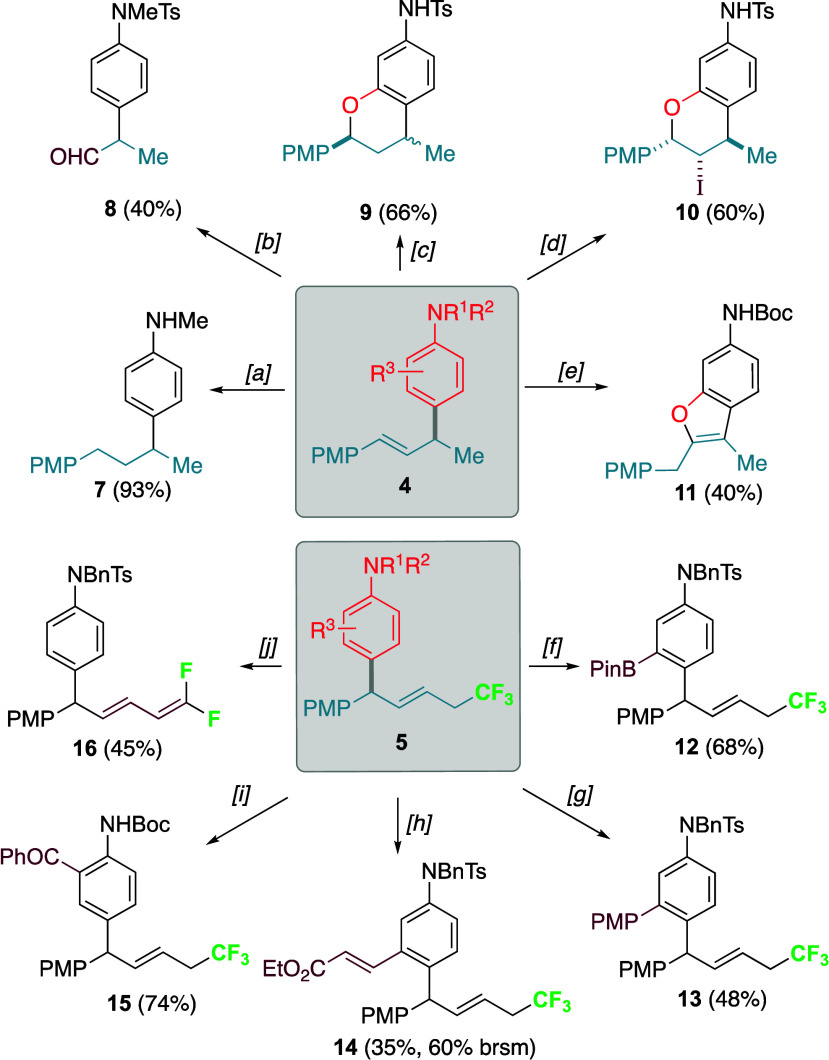
Functionalization and Diversification of the Arylation Products Compound **4** (1.0 equiv), Pd/C (1 mol %), H_2_ (balloon), MeOH, rt. Compound **4** (1.0 equiv), RuCl_3_ (5 mol %), PhI(OAc)_2_ (3.0 equiv), CH_2_Cl_2_/H_2_O, rt. Compound **4** (1.0 equiv), TfOH (5 mol %), CH_2_Cl_2_, rt. Compound **4** (1.0 equiv), NaHCO_3_ (3.0 equiv), I_2_ (3.0 equiv), MeCN, 0 °C. Compound **4** (1.0 equiv), Pd(MeCN)_2_Cl_2_ (5 mol %), benzoquinone (1.0 equiv), 1,4 dioxane, 80 °C. Compound **5** (1.0 equiv), Pd(*o*-tolyl_3_P)_2_Cl_2_ (20 mol %), B_2_pin_2_ (2.0 equiv), AcOH (3.0 equiv), K_2_CO_3_ (5.0 equiv), DMF (0.1 M), 80 °C. Compound **5** (1.0 equiv), Pd(OAc)_2_ (1 mol %), Ph_3_P (4 mol %), PMPB(OH)_2_ (1.2 equiv), Na_2_CO_3_ (2.5 equiv), PhMe/EtOH/H_2_O, 80 °C. Compound **5** (1.0 equiv), Pd(OAc)_2_ (10 mol %), Ph_3_P (20 mol %), ethyl acrylate (3.0 equiv), Et_3_N (5.0 equiv), DMF, 120 °C. Compound **5** (1.0 equiv), ^*t*^BuLi (2.4 equiv), PhCOCl (2.4 equiv), THF, –78 °C. Compound **5** (1.0 equiv), NaHMDS (1.1 equiv) in THF at −78°C. PMP: *para*-methoxyphenyl. Pin: pinacol

Density functional theory (DFT) calculations at the dispersion-corrected PCM(HFIP)-M06-2X/def2-TZVPP//PCM(HFIP)-B3LYP-D3/def2-SVP level (see computational details in the Supporting Information) were carried out to understand the origin of the regioselectivity in both transformations. To this end, we explored the alternative 1,2- versus 1,4-additions of aniline **1b** to the allyl cation intermediates **AC-a** (R = H) and **AC-b** (R = CF_3_), which are proposed as the key species formed upon the initial electrophile transfer (proton for the hydroarylation and CF_3_ for the trifluoromethylarylation)^43,44,62,63^ to the conjugated diene (see the “Mechanistic experiments” section in the Supporting Information).

According to the computed reaction profile shown in Figure 1a, the process begins from a van der Waals reactant complex (**RC**), which leads to the corresponding reaction products through the corresponding transition states **TS1**,**2**/**TS1**,**4**. From the data in Figure 1a, it becomes evident that the 1,2-addition products are thermodynamically favored for both processes. Interestingly, while the 1,2-addition is also slightly kinetically favored in the hydroarylation reaction (ΔΔG^‡^ = −0.9 kcal/mol), the transition state associated with the 1,4-addition is clearly favored in the trifluoromethylarylation reaction (ΔΔG^‡^ = 2.6 kcal/mol). Therefore, our calculations suggest that the 1,2-addition hydroarylation takes place preferentially under both kinetic and thermodynamic control, whereas the analogous 1,4-addition is preferred kinetically for the CF_3_-substituted substrates.

**Figure 1:**
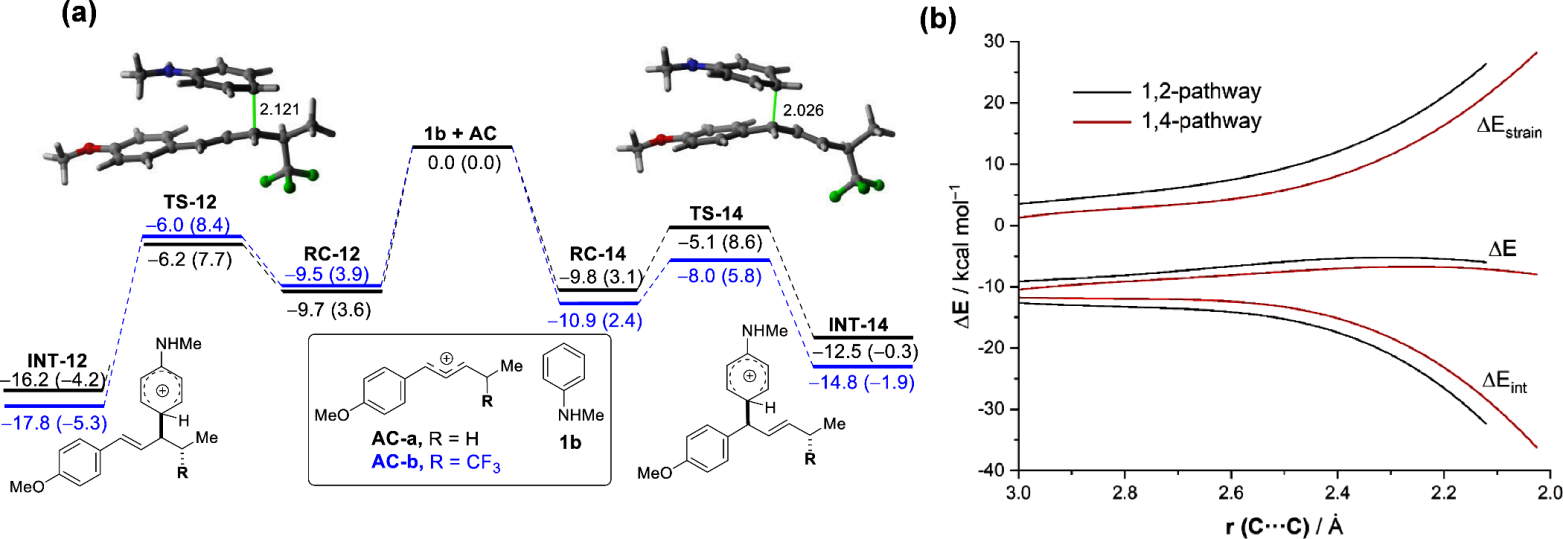
(a) Computed reaction profile for the reaction of aniline **1b** and allyl cationic intermediates **AC-a** (black) and **AC-b** (blue) involving the alternative 1,2- vs 1,4-addition reactions. Relative energies (ΔE, plain values) and free energies (ΔG, at 298 K) are given in kcal/mol. (b) Comparative activation strain analyses for both pathways involved in the **1b** + **AC-b** reaction projected onto the C···C bond-forming distance. All data were computed at the PCM(HFIP)-M06-2X/def2-TZVPP//PCM(HFIP)-B3LYP-D3/def2-SVP level.

To gain more insight into the factors leading to the 1,4-regioselectivity in the trifluoromethylarylation reaction, we applied the activation strain model (ASM) of reactivity.^64,65^ This analysis involves decomposing the electronic energy (ΔE) into two terms, namely, the strain (ΔE_strain_) that results from the distortion of the individual reactants and the interaction (ΔE_int_) between the increasingly deformed reactants along the reaction coordinate. As graphically shown in Figure 1b, which shows the corresponding diagrams for both possible approaches from the beginning of the transformation up to the corresponding transition states and projected onto the C···C bond-forming distance, it becomes clear that the lower barrier computed for the 1,4-pathway is not at all due to the interaction between the deformed reactants, which is actually stronger (i.e., more stabilizing) for the 1,2-pathway. Instead, the strain energy is clearly less destabilizing for the 1,4-approach and can compensate for the stabilizing effect of ΔE_int_, which is ultimately translated into the lower barrier computed for this pathway. A similar activation strain diagram is found for the analogous hydroarylation reaction involving **AC-a** (see Figure S6 in the Supporting Information). However, in this particular case, the strain energy is rather similar for both approaches, which renders the 1,2-addition favored as a consequence of the stronger interaction energy computed for this approach.

In summary, we have developed a unified strategy to selectively functionalize 1,3-dienes in a regiocontrolled manner using anilines in hexafluoroisopropanol. While the hydroarylation transformation gives the 1,2-addition product, the trifluoromethylarylation manifold affords the 1,4-addition analogue. DFT calculations support a mechanism through a similar allyl carbocation species where the regiodivergent outcome follows a kinetically controlled pathway, particularly for the trifluoromethylarylation reaction. It is important to emphasize that these transformations uncover for the first time a double regioselective protocol, both with respect to the diene (1,2- vs 1,4-) and the aniline (*N*- vs *ortho* vs *meta* vs *para*) functionalization.

## Supporting Information Available

Experimental procedures and characterization data of all new compounds, including NMR spectra (PDF)
Crystallographic data for **6** (CIF)

## Supplementary Material

au4c00162_si_001.pdf

au4c00162_si_002.cif
