# Supplementary material for: Understanding the Regiodivergence between Hydroarylation and Trifluoromethylarylation of 1,3-Dienes Using Anilines in HFIP
Source: JACS Au. 2024 May 10;4(5):1744–51. doi: 10.1021/jacsau.4c00162 (PMC11134361; doi:10.1021/jacsau.4c00162)
Supplement: Supplementary file 1 — au4c00162_si_001.pdf [file au4c00162_si_001.pdf]

Understanding the Regiodivergence Between Hydroarylation and Trifluoromethylarylation of 1,3-Dienes using anilines in HFIP

Carlos Corral Suarez, [a] Israel Fernández[b] and Ignacio Colomer*[a]

[a] Instituto de Química Orgánica General (IQOG), CSIC, Juan de la Cierva 3, 28006 Madrid, Spain.

[b] Departamento de Química Orgánica and Centro de Innovación en Química Avanzada (ORFEO-CINQA), Facultad de Ciencias Químicas, Universidad Complutense de Madrid, Ciudad Universitaria, 28040-Madrid, Spain.

Table of Contents

1. **General experimental details** S3
2. **Synthesis of starting materials** S3
3. **Optimization** S5
4. **General procedure for the hydroarylation of dienes** S6
5. **General procedure for the trifluoromethylarylation of dienes** S19
6. **Synthesis of sulfonamides and carbamates** S33
7. **Functionalization of hydroarylation products 4** S38
8. **Functionalization of trifluoromethylarylation products 5** S44
9. **Mechanistic experiments** S49
10. **Computational details** S54
11. **References** S68
12. **X-Ray diffraction analysis** S69
13. **NMR spectra** S82

1. General experimental details

^{1}H NMR and ^{13}C NMR spectra were recorded on a Bruker DPX 400 MHz or VARIAN INOVA-300 MHz spectrometer in $CDCl_3$, DMSO-d_6 or MeOH-d_4 and referenced to residual solvent peaks. Chemical shifts are quoted in ppm (parts per million) to the nearest 0.01 ppm with signal splitting recorded as singlet (s), doublet (d), triplet (t), quartet (q), quintet (quint), multiplet (m) and broad singlet (br s). Coupling constants, *J*, are measured in Hz to the nearest 0.1 Hz. ^{1}H, ^{19}F and ^{13}C NMR spectra were recorded at room temperature. High resolution mass spectra are given to four decimal places and were registered in a spectrometer GCT Agilent Technologies 6890N using electrospray (ESI) and Time-of-Flight (TOF) detector. Melting points (m.p.) were obtained from recrystallized samples using a koffler block microscope and are uncorrected. The solvent systems used for recrystallization are quoted in parentheses. Flash column chromatography was performed using silica gel (60 Å, 0.033-0.070 mm, BDH). TLC analyses were performed on Merck Kiesegel 60 F_{254} 0.25 mm precoated silica plates. Reagents obtained from Sigma-Aldrich, Alfa, Fluorochem, Apollo, BLD and TCI suppliers were used directly as supplied. All anhydrous reactions were carried out in flame dried glassware and under an inert atmosphere of argon. All reactions were stirred with magnetic followers.

2. Synthesis of starting materials

2.1. Synthesis of *N*-Benzylanilines

N-Benzylanilines were obtained following well established reductive amination procedure.

General procedure for reductive amination

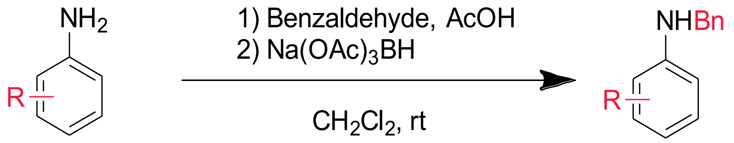

S1 **1**

A solution of benzaldehyde (1.0 equiv.), aniline **S1** (1.1 equiv.) and AcOH (0.25 equiv.) in CH_2Cl_2 (0.15 M) was stirred for 30 min at room temperature and sodium triacetoxyborohydride (1.5 equiv.) was added in one portion. The reaction mixture was monitored until completion by TLC and quenched with saturated NH_4Cl aqueous solution. The organic layer was separated, and the aqueous layer was extracted with dichloromethane. The combined organic layers were dried over Na_2SO_4, concentrated under reduced pressure, and purified by flash column chromatography (EtOAc/Hexane) to afford product **1**.

2.2. Synthesis of dienes

Conjugated dienes **2a-d**, **2f-h** and **2j-2o** were obtained following well established Wittig olefination procedure using the corresponding α,β-unsaturated aldehyde.

Diene **2i** was synthesised following described procedure.[1]

α,β-unsaturated aldehyde for **2e**, **2f** and **2m** were synthesised following the described procedure.[2]

α,β-unsaturated aldehyde for **2g** and **2o** were synthesised following the described procedure.[3]

α,β-unsaturated aldehyde for **2n** was synthesised following the described procedure.[4]

General procedure for Wittig olefination

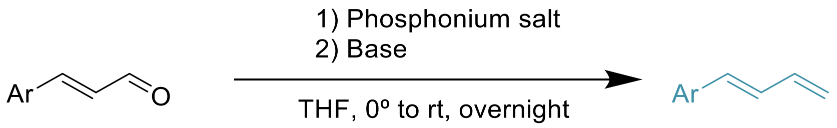

S2 **2**

A suspension of the triphenylphosphonium bromide (1.3 equiv.) in dry THF (5.0 mL/mmol) was placed in a flame-dried round-bottom flask. The solution was cooled to 0 °C and kept under argon. The base (1.5 - 1.8 equiv. of *t*-BuOK, or *n*-BuLi) was added in one portion. After stirring at 0 °C for 30 min the solution turns into an intense bright colour, then the corresponding aldehyde **S2** (1.0 equiv.) was added. The reaction mixture was gradually warmed to room temperature. After stirring overnight, the reaction was quenched by the slow addition of saturated NH_4Cl. The phases were separated, and the aqueous phase was extracted twice with Et_2O. The combined organic layers were dried over Na_2SO_4. The solvent was evaporated under reduced pressure to give the corresponding diene **2**, that was purified by chromatography on silica gel using the appropriate mixture of eluents.

Synthesis of diene 2e

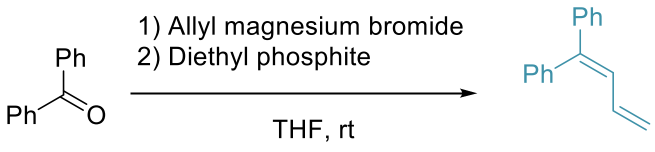

benzophenone **2e**

To a solution of benzophenone (5.0 mmol, 1.0 equiv.) in dry THF (50 mL) was added a solution of allyl magnesium bromide (11 mL, 11 mmol, 1 M in THF, 2.2 equiv.) under nitrogen atmosphere at room temperature. The mixture was stirred for 30 min, monitored by TLC, then diethyl

phosphite (772 uL, 6.0 mmol, 1.2 equiv.) was added and the reaction mixture was stirred for 4 h. The reaction mixture was quenched with water and extracted twice with EtOAc and the combined organic layers were dried over Na_2SO_4 and evaporated under reduced pressure to give diene **2e**, that was purified by chromatography on silica gel using the appropriate mixture of eluents.

3. Optimization

Table S1. *Optimization for the hydroarylation of dienes using anilines.*

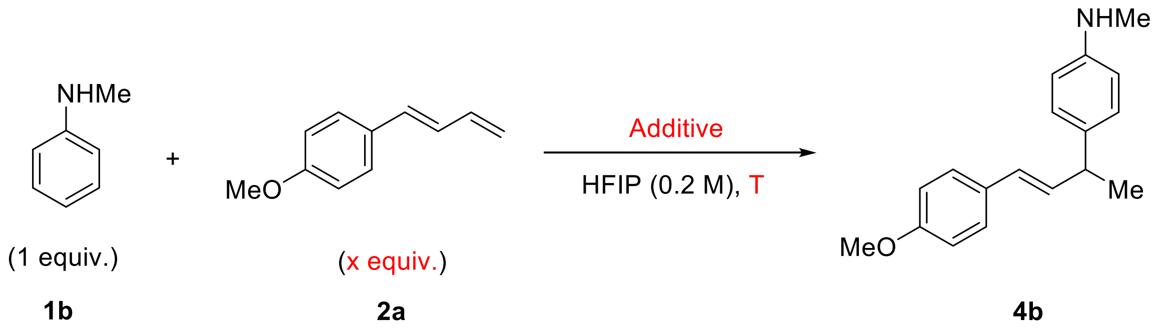

Entry	Temperature (ºC)	2a (equiv.)	Additives	Isolated Yield, 4b (%)
**1**	25	1	-	27
**2**	25	2	-	39
**3**	80	1	-	57
**4**	80	2	-	71
**5**	80	1	1.0 equiv. NaOAc	30
**6**	80	2	1.0 equiv. NaOAc	68

Table S2. *Optimization for the trifluoromethylarylation of dienes using anilines.*

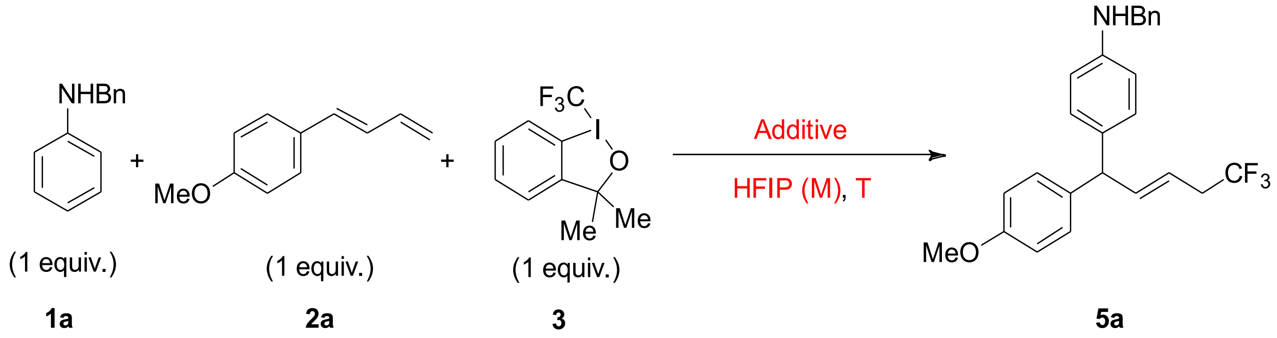

Entry	Temperature (ºC)	Solvent (M)	Additives	Isolated Yield, 5a (%)
**1**	25	HFIP (0.2M)	-	47
**2**	40	HFIP (0.4M)	-	55
**3**	80	DCM (0.2M)	5 mol % TfOH	0

DCM: dichloromethane

4. General procedure for the hydroarylation of dienes using anilines in HFIP.

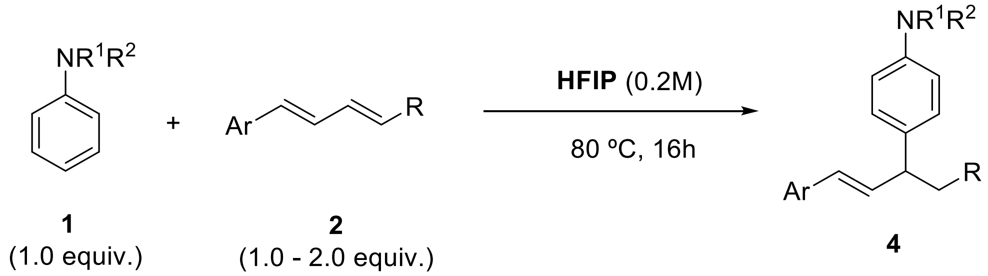

To a 10 mL oven-dried vial equipped with a stirrer bar was added the corresponding aniline **1** (1.0 equiv.), diene **2** (1.0 - 2.0 equiv.) and HFIP (5.0 mL/mmol). The reaction vessel was sealed with a septum cap and purged with Ar. Following this, the vial was heated to 80 ºC, and stirred overnight, unless other temperature stated. The mixture was cooled to rt, monitored by TLC and the solvent was evaporated under reduced pressure. The crude reaction was purified by chromatography on silica gel using the appropriate mixture of eluents to give the corresponding product **4**.

(*E*)-*N*-Benzyl-4-[4-(4-methoxyphenyl)but-3-en-2-yl]aniline, 4a.

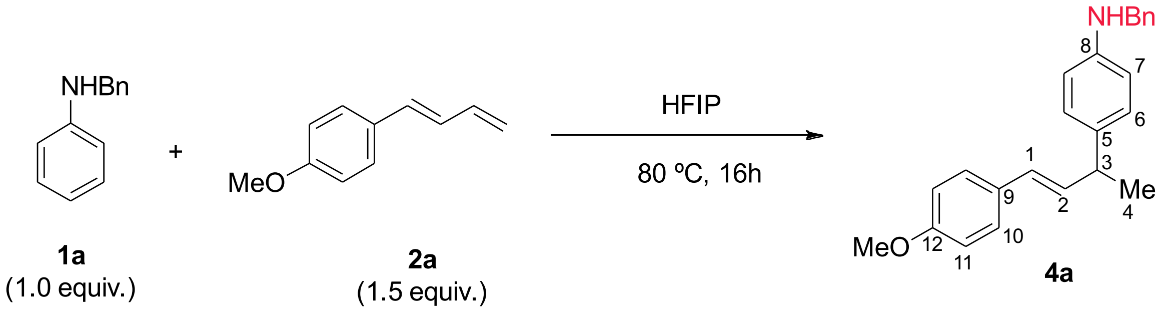

From aniline **1a** (36.7 mg, 0.2 mmol) and diene **2a** (48.1 mg, 0.3 mmol), in 1.0 mL of HFIP, following the general procedure, aniline **4a** was obtained. Chromatographic purification (gradient elution: 0:100 → 15:85 Et_2O – hexane) gave aniline **4a** (42.6 mg, 62%) as a colourless oil.

Data for **4a**: **R*f*** 0.30 (30% Et_2O – hexane). **^{1}H NMR (400 MHz, $CDCl_3$)** δ 7.48– 7.33 (5H, m, Ar), 7.30 (2H, d, *J* = 8.7 Hz, 10-H), 7.10 (2H, d, *J* = 8.4 Hz, 6-H), 6.84 (2H, d, *J* = 8.8 Hz, 11-H), 6.63 (2H, d, *J* = 8.5 Hz, 7-H), 6.34 (1H, d, *J* = 17.0 Hz, 1-H), 6.23 (1H, dd, *J* = 15.9 and 6.7 Hz, 2-H), 4.33 (2H, s, CH_2 Bn), 3.81 (3H, s, OMe), 3.53 (1H, quint, *J* = 6.9 Hz, 3-H), 1.42 (3H, d, *J* = 7.0 Hz, Me). **^{13}C NMR (101 MHz, $CDCl_3$)** δ 158.8 (1C, C Ar), 146.7 (1C, C Ar), 139.7 (1C, C Ar), 135.0 (1C, C Ar), 134.1 (1C, C-2), 130.8 (1C, C Ar), 128.7 (2C, CH Ar), 128.2 (2C, C-6), 127.7 (2C, CH Ar), 127.4 (1C, CH Ar), 127.33 (1C, C-1), 127.32 (2C, C-10), 114.0 (2C, C-11), 113.1 (2C, C-7), 55.4 (1C, OMe), 48.7 (1C, CH_2 Bn), 41.8 (1C, C-3), 21.5 (1C, C-4). **HRMS** (ESI): calculated for $C_{24}H_{26}NO$ $[M+H]^+$ requires *m/z* 344.2009, found 344.2004.

(*E*)-4-[4-(4-Methoxyphenyl)but-3-en-2-yl]-*N*-methylaniline, 4b.

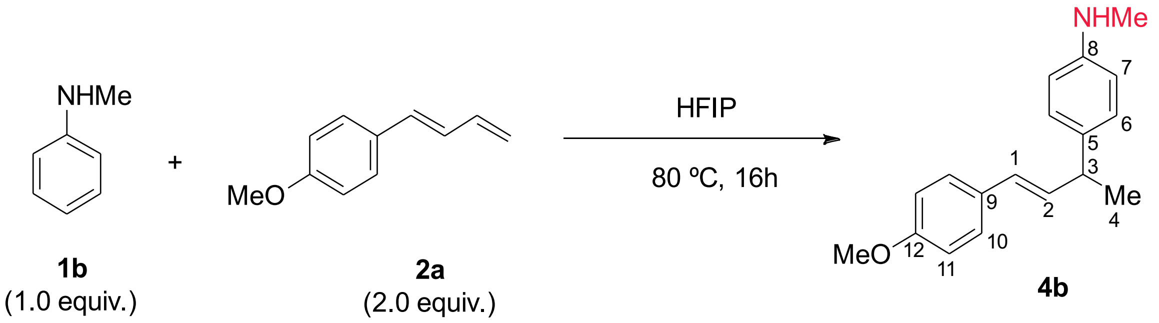

From aniline **1b** (21.4 mg, 0.2 mmol) and diene **2a** (64.1 mg, 0.4 mmol), in 1.0 mL of HFIP, following the general procedure, aniline **4b** was obtained. Chromatographic purification (gradient elution: 20:100 → 20:80 Et_2O – hexane) gave aniline **4b** (41.2 mg, 77%) as a yellow oil.

Data for **4b**: **R*f*** 0.30 (20% Et_2O – hexane). **^{1}H NMR (400 MHz, $CDCl_3$)** δ 7.29 (2H, d, *J* = 8.7 Hz, 10-H), 7.11 (2H, d, *J* = 8.5 Hz, 6-H), 6.83 (2H, d, *J* = 8.7 Hz, 11-H), 6.60 (2H, d, *J* = 8.5 Hz, 7-H), 6.34 (1H, d, *J* = 16.0 Hz, 1-H), 6.23 (1H, dd, *J* = 15.9 and 6.5 Hz, 2-H), 3.80 (3H, s, OMe), 3.53 (1H, quint, *J* = 6.9 Hz, 3-H), 2.83 (3H, s, NMe), 1.42 (3H, d, *J* = 7.0 Hz, Me). **^{13}C NMR (101 MHz, $CDCl_3$)** δ 158.8 (1C, C Ar), 147.9 (1C, C Ar), 134.7 (1C, C Ar), 134.2 (1C, C-2), 130.8 (1C, C Ar), 128.1 (2C, C-6), 127.34 (1C, C-1), 127.31 (2C, C-10), 114.0 (2C, C-11), 112.7 (2C, C-7), 55.4 (1C, OMe), 41.7 (1C, C-3), 31.1 (1C, NMe), 21.5 (1C, C-4). **HRMS** (ESI): calculated for $C_{18}H_{22}NO$ $[M+H]^+$ requires *m/z* 268.1696, found 268.1697.

(*E*)-4-[4-(4-Methoxyphenyl)but-3-en-2-yl]-*N*,*N*-dimethylaniline, 4c.

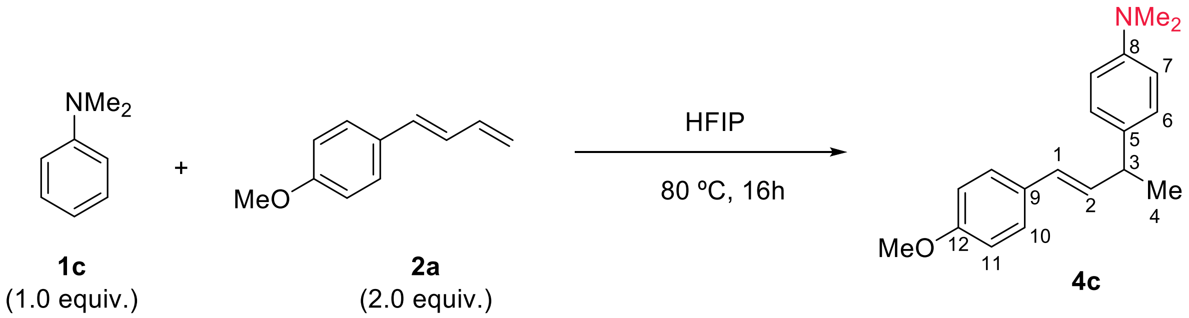

From aniline **1c** (24.2 mg, 0.2 mmol) and diene **2a** (64.1 mg, 0.4 mmol), in 1.0 mL of HFIP, following the general procedure, aniline **4c** was obtained. Chromatographic purification (gradient elution: 0:100 → 15:85 Et_2O – hexane) gave aniline **4c** (45.0 mg, 80%) as a colourless oil.

Data for **4c**: **R$_f$** 0.50 (5% Et_2O – hexane). **^{1}H NMR (400 MHz, $CDCl_3$)** δ 7.32 (2H, d, *J*= 8.8 Hz, 10-H), 7.19 (2H, d, *J* = 8.7 Hz, 6-H), 6.86 (2H, d, *J* = 8.8 Hz, 11-H), 6.76 (2H, d, *J* = 8.8 Hz, 7-H), 6.38 (1H, d, *J* = 15.9 Hz, 1-H), 6.27 (1H, dd, *J* = 17.3 and 6.7 Hz, 2-H), 3.82 (3H, s, OMe), 3.58 (1H, quint, *J* = 6.9 Hz, 3-H), 2.96 (6H, s, NMe_2), 1.47 (3H, d, *J* = 7.1 Hz, Me). **^{13}C NMR (101 MHz,**

CDCl₃) δ 158.8 (1C, C Ar), 149.4 (1C, C Ar), 134.11 (1C, C-2), 134.07 (1C, C Ar), 130.8 (1C, C Ar), 128.0 (2C, C-6), 127.34 (1C, C-1), 127.30 (2C, C-10), 114.0 (2C, C-11), 113.1 (2C, C-7), 55.4 (1C, OMe), 41.6 (1C, C-3), 41.0 (2C, NMe_2), 21.5 (1C, C-4). **HRMS** (ESI): calculated for $C_{19}H_{24}NO$ $[M+H]^+$ requires *m/z* 282.1852, found 282.1852.

(*E*)-4-[4-(4-methoxyphenyl)but-3-en-2-yl]aniline, 4d.

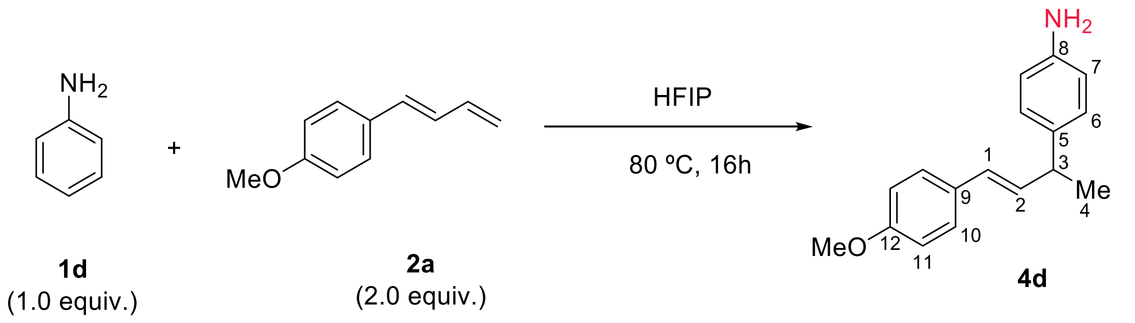

From aniline **1d** (18.6 mg, 0.2 mmol) and diene **2a** (64.1 mg, 0.4 mmol), in 1.0 mL of HFIP, following the general procedure, aniline **4d** was obtained. Chromatographic purification (gradient elution: 20:100 → 50:50 Et_2O – hexane) gave aniline **4d** (36 mg, 71%) as a yellow oil.

Data for **4d**: **R_f** 0.10 (50% Et_2O – hexane). **^{1}H NMR (400 MHz, $CDCl_3$)** δ 7.29 (2H, d, *J* = 8.8 Hz, 10-H), 7.07 (2H, d, *J* = 8.3 Hz, 6-H), 6.84 (2H, d, *J* = 8.8 Hz, 11-H), 6.66 (2H, d, *J* = 8.4 Hz, 7-H), 6.33 (1H, d, *J* = 15.9 Hz, 1-H), 6.23 (1H, ddd, *J* = 15.9, 6.6 and 0.9 Hz, 2-H), 3.80 (3H, s, OMe), 3.58 (2H, br s, NH_2), 3.53 (1H, t, *J* = 6.9 Hz, 3-H), 1.42 (3H, d, *J* = 7.0 Hz, Me). **^{13}C NMR (101 MHz, $CDCl_3$)** δ 158.8 (1C, C Ar), 144.7 (1C, C Ar), 136.1 (1C, C Ar), 134.0 (1C, C-2), 130.7 (1C, C Ar), 128.2 (2C, C-6), 127.5 (1C, C-1), 127.3 (2C, C-10), 115.4 (2C, C-7), 114.0 (2C, C-11), 55.4 (1C, OMe), 41.8 (1C, C-3), 21.5 (1C, C-4). **HRMS** (ESI): calculated for $C_{17}H_{20}NO$ $[M+H]^+$ requires *m/z* 254.1539, found 254.1540.

(*E*)-2-Chloro-4-[4-(4-methoxyphenyl)but-3-en-2-yl]-*N*-methylaniline, 4e.

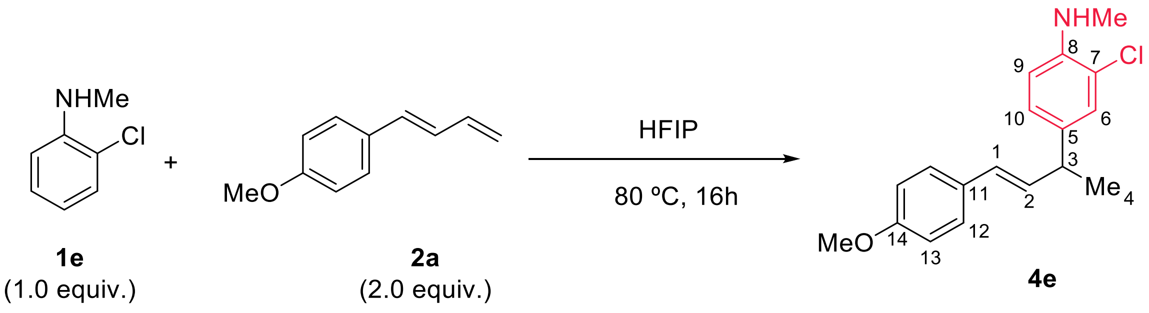

From aniline **1e** (28.3 mg, 0.2 mmol) and diene **2a** (64.1 mg, 0.4 mmol), in 1.0 mL of HFIP, following the general procedure, aniline **4e** was obtained. Chromatographic purification (gradient elution: 0:100 → 20:80 Et_2O – hexane) gave aniline **4e** (36 mg, 60%) as an orange oil.

Data for **4e**: **R*f*** 0.40 (30% Et_2O – hexane). **^{1}H NMR (400 MHz, $CDCl_3$)** δ 7.29 (2H, d, *J* = 8.7 Hz, 12-H), 7.17 (1H, d, *J* = 2.1 Hz, 6-H), 7.07 (1H, dd, *J* = 8.3 and 2.1 Hz, 10-H), 6.84 (2H, d, *J* = 8.7 Hz, 13-H), 6.62 (1H, d, *J* = 8.3 Hz, 9-H), 6.33 (1H, dd, *J* = 15.8 and 1.4 Hz, 1-H), 6.19 (1H, dd, *J* = 15.9 and 6.7 Hz, 2-H), 4.24 (1H, br s, NH), 3.80 (3H, s, OMe), 3.51 (1H, quint, *J* = 6.9 Hz, 3-H), 2.89 (3H, s, NMe), 1.41 (3H, d, *J* = 7.0 Hz, Me).**^{13}C NMR (101 MHz, $CDCl_3$)** δ 158.9 (1C, C Ar), 143.5 (1C, C Ar), 135.0 (1C, C Ar), 133.4 (1C, C-2), 130.5 (1C, C Ar), 127.9 (1C, C-6), 127.8 (1C, C-1), 127.4 (2C, C-12), 126.8 (1C, C-10), 119.2 (1C, C Ar), 114.1(2C, C-13), 110.9 (1C, C-9), 55.4 (1C, OMe), 41.5 (1C, C-3), 30.7 (1C, NMe), 21.4 (1C, C-4). **HRMS** (ESI): calculated for $C_{18}H_{20}NO$ $[M+H]^+$ requires *m/z* 302.1306, found 302.1305.

(*E*)-2-Bromo-4-[4-(4-methoxyphenyl)but-3-en-2-yl]-*N*-methylaniline, 4f.

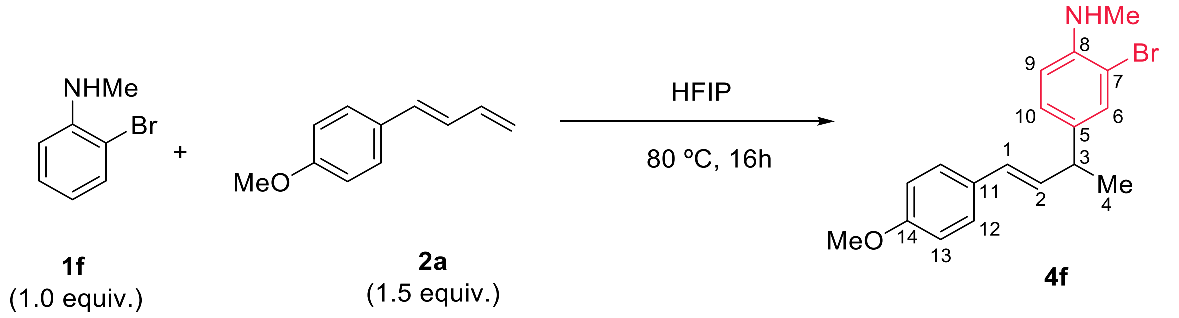

From aniline **1f** (37.2 mg, 0.2 mmol) and diene **2a** (48.1 mg, 0.3 mmol), in 1.0 mL of HFIP, following the general procedure, aniline **4f** was obtained. Chromatographic purification (gradient elution: 0:100 → 20:80 Et_2O – hexane) gave aniline **4f** (34.6 mg, 50%) as a colourless oil.

Data for **4f**: **R*f*** 0.30 (30% Et_2O – hexane). **^{1}H NMR (400 MHz, $CDCl_3$)** δ 7.35 (1H, d, *J* = 2.1 Hz, 6-H), 7.30 (2H, d, *J* = 8.7 Hz, 12-H), 7.13 (1H, dd, *J* = 8.3 and 2.1 Hz, 10-H), 6.85 (2H, d, *J* = 8.8 Hz, 13-H), 6.62 (1H, d, *J* = 8.3 Hz, 9-H), 6.35 (1H, dd, *J* = 15.9 and 1.4 Hz, 1-H), 6.20 (1H, dd, *J* = 15.8 and 6.7 Hz, 2-H), 3.81 (3H, s, OMe), 3.52 (1H, quint, *J* = 6.9 Hz, 3-H), 2.89 (3H, s, NMe), 1.42 (3H, d, *J* = 7.0 Hz, Me). **^{13}C NMR (101 MHz, $CDCl_3$)** δ 158.9 (1C, C Ar), 144.3 (1C, C Ar), 135.6 (1C, C Ar), 133.3 (1C, C-2), 131.1 (1C, C-6), 130.5 (1C, C Ar), 127.8 (1C, C-1), 127.5 (1C, C-10), 127.4 (2C, C-12), 114.0 (2C, C-13), 111.0 (1C, C-9), 109.9 (1C, C Ar), 55.4 (1C, OMe), 41.5 (1C, C-3), 31.0 (1C, NMe), 21.4 (1C, C-4). **HRMS** (ESI): calculated for $C_{18}H_{21}BrNO$ $[M+H]^+$ requires *m/z* 346.0806 and 348.0781, found 346.0776 and 348.0756.

(*E*)-4-[4-(4-Methoxyphenyl)but-3-en-2-yl]-*N*,2-dimethylaniline, 4g.

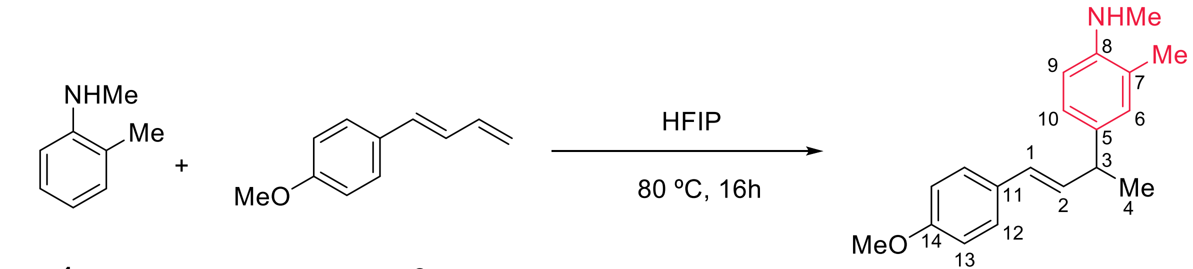

From aniline **1g** (24.2 mg, 0.2 mmol) and diene **2a** (48.1 mg, 0.3 mmol), in 1.0 mL of HFIP, following the general procedure, aniline **4g** was obtained. Chromatographic purification (gradient elution: 0:100 → 20:80 Et_2O – hexane) gave aniline **4g** (42.7 mg, 76%) as a colourless oil.

Data for **4g**: **R*f*** 0.30 (30% Et_2O – hexane). **^{1}H NMR (400 MHz, $CDCl_3$)** δ 7.32 (2H, d, *J* =8.7 Hz, 12-H), 7.10 (1H, d, *J* = 8.2 Hz, 10-H), 7.00 (1H, s, 6-H), 6.86 (2H, d, *J* = 8.7 Hz, 13-H), 6.61 (1H, d, *J* = 8.2, 9-H), 6.38 (1H, d, *J* = 15.9 Hz, 1-H), 6.27 (1H, dd, *J* = 15.9 and 6.6 Hz, 2-H), 3.82 (3H, s, OMe), 3.55 (1H, quint, *J* = 6.9 Hz, 3-H), 3.50 (1H, br s, NH), 2.91 (3H, s, NMe), 2.16 (3H, s, Me), 1.46 (3H, d, *J* = 7.1 Hz, Me). **^{13}C NMR (101 MHz, $CDCl_3$)** δ 158.8 (1C, C Ar), 145.8 (1C, C Ar), 134.28 (1C, C Ar), 134.27 (1C, C-2), 130.8 (1C, C Ar), 129.2 (1C, C-6), 127.3 (2C, C-12), 127.2 (1C, C-1), 125.8 (1C, C-10), 122.2 (1C, C Ar), 114.0 (2C, C-13), 109.4 (1C, C-9), 55.4 (1C, OMe), 41.8 (1C, C-3), 31.1 (1C, NMe), 21.6 (1C, C-4), 17.6 (1C, Me). **HRMS** (ESI): calculated for $C_{19}H_{24}NO$ $[M+H]^+$ requires *m/z* 282.1852, found 282.1851.

(*E*)-*N*,2-Dibenzyl-4-[4-(4-methoxyphenyl)but-3-en-2-yl]aniline, 4h.

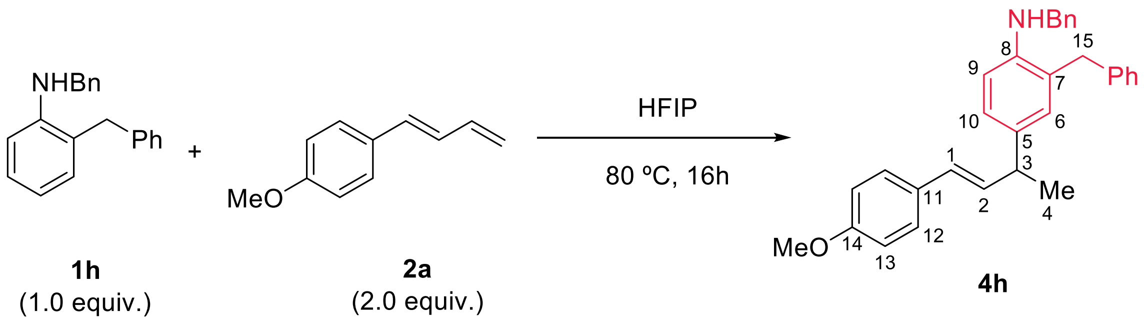

From aniline **1h** (54.7 mg, 0.2 mmol) and diene **2a** (64.1 mg, 0.4 mmol), in 1.0 mL of HFIP, following the general procedure, aniline **4h** was obtained. Chromatographic purification (gradient elution: 0:100 → 20:80 Et_2O – hexane) gave aniline **4h** (57.2 mg, 66%) as a yellow oil.

Data for **4h**: **R*f*** 0.50 (30% Et_2O – hexane). **^{1}H NMR (400 MHz, $CDCl_3$)** δ 7.34 – 7.17 (10H, m, Ar), 7.11 (1H, d, *J* = 8.0 Hz, Ar), 7.11 (1H, d, *J* = 7.6 Hz, Ar), 7.07 (1H, dd, *J* = 8.2 and 2.2 Hz, 10-H), 7.03 (1H, d, *J* = 2.2 Hz, 6-H), 6.85 (2H, d, *J* = 8.8 Hz, 13-H), 6.62 (1H, d, *J* = 8.2 Hz, 9-H),

6.35 (1H, d, J = 15.9 Hz, 1-H), 6.25 (1H, dd, J = 15.9 and 6.6 Hz, 2-H), 4.24 (2H, s, CH_2 Bn), 3.95 (2H, s, 15-H_2), 3.81 (3H, s, OMe), 3.55 (1H, quint, J = 6.8 Hz, 3-H), 1.44 (3H, d, J = 7.0 Hz, 4-H). **^{13}C NMR (101 MHz, $CDCl_3$)** δ 158.8 (1C, C Ar), 144.3 (1C, C Ar), 139.5 (1C, C Ar), 139.4 (1C, C Ar), 134.9 (1C, C Ar), 134.2 (1C, C-2), 130.8 (1C, C Ar), 130.0 (1C, C-6), 128.8 (2C, CH Ar), 128.7 (2C, CH Ar), 128.6 (2C, CH Ar), 127.43 (1C, CH Ar), 127.38 (2C, CH Ar), 127.3 (2C, CH Ar), 127.2 (1C, C-1), 126.5 (2C, CH Ar and C-10), 125.0 (1C, C Ar) 114.0 (2C, C-13), 111.4 (1C, C-9) 55.4 (1C, OMe), 48.5 (1C, CH_2 Bn), 41.8 (1C, C-3), 38.6 (1C, C-15), 21.6 (1C, C-4). **HRMS** (ESI): calculated for $C_{31}H_{32}NO$ $[M+H]^+$ requires *m/z* 434.2478, found 434.2458.

(*E*)-*N*-Benzyl-5-[4-(4-methoxyphenyl)but-3-en-2-yl]-(1,1'-biphenyl)-2-amine, 4i.

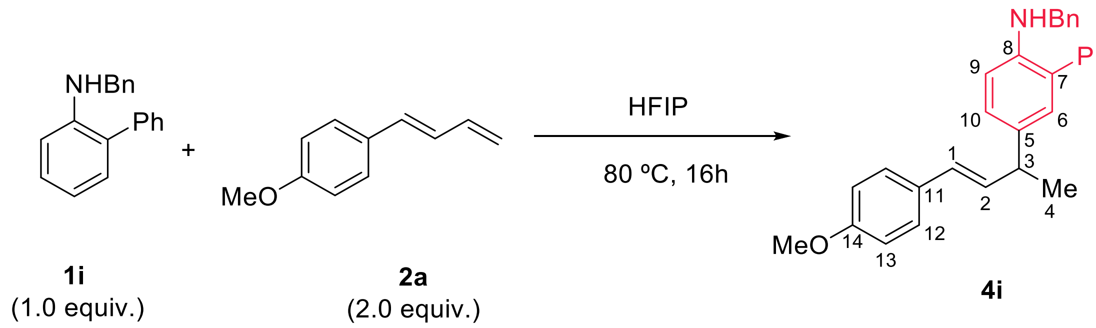

From aniline **1i** (51.9 mg, 0.2 mmol) and diene **2a** (64.1 mg, 0.4 mmol), in 1.0 mL of HFIP, following the general procedure, aniline **4i** was obtained. Chromatographic purification (gradient elution: 0:100 → 20:80 Et_2O – hexane) gave aniline **4i** (72.9 mg, 84%) as a colourless oil.

Data for **4i**: **R*f*** 0.30 (30% Et_2O – hexane). **^{1}H NMR (400 MHz, $CDCl_3$)** δ 7.50 – 7.39 (4H, m, Ar), 7.35 – 7.20 (6H, m, Ar), 7.28 (2H, d, J = 8.7 Hz, 12-H), 7.09 (1H, dd, J = 8.4 and 2.3 Hz, 10-H), 7.02 (1H, d, J = 2.2 Hz, 6-H), 6.81 (2H, d, J = 8.8 Hz, 13-H), 6.66 (1H, d, J = 8.3 Hz, 9-H), 6.34 (1H, d, J = 16.0 Hz, 1-H), 6.22 (1H, dd, J = 15.8 and 6.8 Hz, 2-H), 4.30 (2H, s, CH_2 Bn), 3.78 (3H, s, OMe), 3.54 (1H, quint, J = 6.9 Hz, 3-H), 1.42 (3H, d, J = 7.0 Hz, Me). **^{13}C NMR (101 MHz, $CDCl_3$)** δ 158.8 (1C, C Ar), 143.0 (1C, C Ar), 139.6 (1C, C Ar), 139.5 (1C, C Ar), 135.0 (1C, C Ar), 133.9 (1C, C-2), 130.7 (1C, C Ar), 129.5 (2C, CH Ar), 129.4 (1C, C-6), 129.1 (2C, CH Ar), 128.7 (2C, CH Ar), 128.2 (1C, C Ar), 127.48 (1C, CH Ar), 127.47 (1C, CH Ar), 127.39 (1C, C-1), 127.34 (2C, CH Ar), 127.33 (2C, CH Ar), 127.2 (1C, C-10), 114.0 (2C, C-12), 111.4 (1C, C-9), 55.4 (1C, OMe), 48.8 (1C, CH_2 Bn), 41.9 (1C, C-3), 21.5 (1C, C-4). **HRMS** (ESI): calculated for $C_{30}H_{30}NO$ $[M+H]^+$ requires *m/z* 420.2322, found 420.2299.

(*E*)-3-Chloro-4-[4-(4-methoxyphenyl)but-3-en-2-yl]-*N*-methylaniline, 4j.

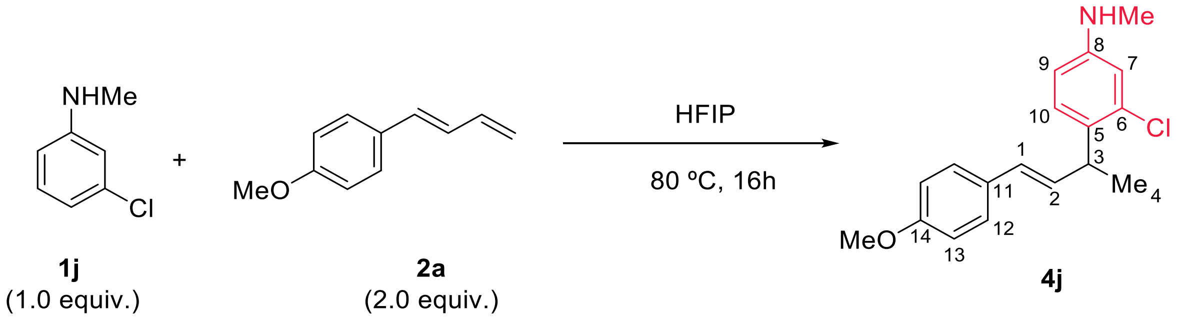

From aniline **1j** (28.3 mg, 0.2 mmol) and diene **2a** (64.1 mg, 0.4 mmol), in 1.0 mL of HFIP, following the general procedure, aniline **4j** was obtained. Chromatographic purification (gradient elution: 0:100 → 10:90 Et_2O – hexane) gave aniline **4j** (28 mg, 46%) as a colourless oil.

Data for **4j**: **R_f** 0.20 (5% Et_2O – hexane). **^{1}H NMR (400 MHz, $CDCl_3$)** δ 7.29 (2H, d, *J* = 8.7 Hz, 12-H), 7.07 (1H, d, *J* = 8.4 Hz, 10-H), 6.83 (2H, d, *J* = 8.8 Hz, 13-H), 6.62 (1H, d, *J* = 2.5 Hz, 7-H), 6.49 (1H, dd, *J* = 8.4 and 2.5 Hz, 9-H), 6.35 (1H, dd, *J* = 15.9 and 1.5 Hz, 1-H), 6.22 (1H, dd, *J* = 16.0 and 6.0 Hz, 2-H), 4.08 – 3.98 (1H, m, 3-H), 3.80 (3H, s, OMe), 2.81 (3H, s, NMe), 1.39 (3H, d, *J* = 7.0 Hz, Me). **^{13}C NMR (101 MHz, $CDCl_3$)** δ 158.9 (1C, C Ar), 148.6 (1C, C Ar), 134.3 (1C, C Ar), 132.5 (1C, C-2), 131.4 (1C, C Ar), 130.7 (1C, C Ar), 128.7 (1C, C-1), 127.9 (1C, C-10), 127.4 (2C, C-12), 114.0 (2C, C-13), 112.7 (1C, C-7), 111.9 (1C, C-9), 55.4 (1C, OMe), 37.6 (1C, C-3), 30.9 (1C, NMe), 20.4 (1C, C-4). **HRMS** (ESI): calculated for $C_{18}H_{21}ClNO$ $[M+H]^+$ requires *m/z* 302.1306, found 302.1303.

(*E*)-*N*-Benzyl-3-bromo-4-[4-(4-methoxyphenyl)but-3-en-2-yl]aniline, 4k.

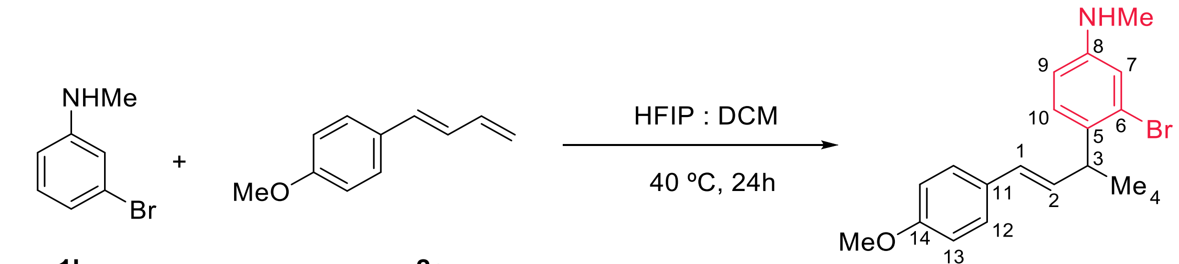

From aniline **1k** (37.2 mg, 0.2 mmol) and diene **2a** (32.0 mg, 0.2 mmol), in 1.0 mL of HFIP: DCM (4:1), following the general procedure (at 40 °C for 24h), aniline **4k** was obtained. Chromatographic purification (gradient elution: 10:90 → 20:80 Et_2O – hexane) gave aniline **4k** (45.1 mg, 65%) as a colourless oil.

Data for **4k**: **R_f** 0.20 (30% Et_2O – hexane). **^{1}H NMR (400 MHz, $CDCl_3$)** δ 7.31 (2H, d, *J* = 8.8 Hz, 12-H), 7.07 (1H, d, *J* = 8.5 Hz, 10-H), 6.85 (2H, d, *J* = 8.7 Hz, 13-H), 6.82 (1H, d, *J* = 2.5

Hz, 7-H), 6.54 (1H, dd, *J* = 8.5 and 2.5 Hz, 9-H), 6.38 1H, (1H, dd, *J* = 16.0 and 1.5 Hz, 1-H), 6.23 (1H, dd, *J* = 16.0 and 5.9 Hz, 2-H), 4.14 – 3.96 (1H, m, 3-H), 3.81 (3H, s, OMe), 3.68 (1H, s, NH), 2.81 (3H, s, NMe), 1.40 (3H, d, *J* = 7.1 Hz, Me). **^{13}C NMR (101 MHz, $CDCl_3$)** δ 158.9 (1C, C Ar), 148.7 (1C, C Ar), 133.0 (1C, C Ar), 132.5 (1C, C-2), 130.7 (1C, C Ar), 128.6 (1C, C-10), 127.9 (1C, C-1), 127.3 (2C, C-12), 125.0 (1C, C Ar), 115.9 (1C, C-7), 114.0 (2C, C-13), 112.6 (1C, C-9), 55.4 (1C, OMe), 40.1 (1C, C-3), 30.8 (1C, NMe), 20.5 (1C, C-4). **HRMS** (ESI): calculated for $C_{18}H_{21}BrNO$ $[M+H]^+$ requires *m/z* 346.0801 and 348.0782, found 346.0797 and 348.0771.

Methyl (*E*)-2-{5-(benzylamino)-2-[4-(4-methoxyphenyl)but-3-en-2-yl]phenyl}acetate, 4l.

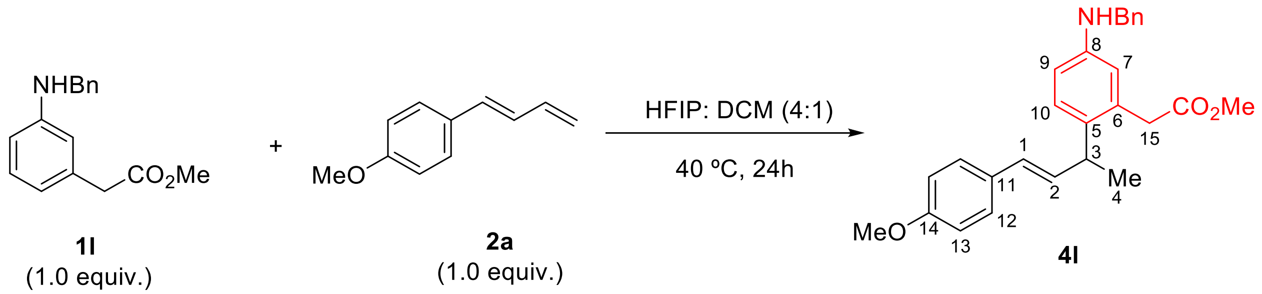

1l (1.0 equiv.) **2a** (1.0 equiv.) **4l**

From aniline **1l** (51.1 mg, 0.2 mmol) and diene **2a** (32.0 mg, 0.2 mmol), in 1.0. mL of HFIP: DCM (4:1) at 40 ºC for 24h, following the general procedure, aniline **4l** was obtained. Chromatographic purification (gradient elution: 0:100 → 50:50 Et_2O – hexane) gave aniline **4l** (19.2 mg, 23%) as a yellowish oil.

Data for **4l**: **R*f*** 0.10 (30% Et_2O – hexane). **^{1}H NMR (400 MHz, $CDCl_3$)** δ 7.42 – 7.23 (5H, m, Ar), 7.26 (2H, d, *J* = 8.8 Hz, 12-H), 7.11 (1H, d, *J* = 8.4 Hz, 10-H), 6.82 (2H, d, *J* = 8.7 Hz, 13-H), 6.62 (1H, dd, *J* = 8.4 and 2.6 Hz, 9-H), 6.57 (1H, d, *J* = 2.6 Hz, 7-H), 6.25 (1H, d, *J* = 16.1 Hz, 1-H), 6.17 (1H, d, *J* = 16.0 and 5.8 Hz, 2-H), 4.31 (2H, s, CH_2 Bn), 3.79 (3H, s, OMe), 3.76 – 3.71 (1H, m, 3-H) 3.68 (1H, d, *J* = 15.6 Hz, 15-H_a), 3.61 (3H, s, CO_2Me), 3.61 (1H, d, *J* = 15.5 Hz, 15-H_b), 1.39 (3H, d, *J* = 6.9 Hz, Me). **^{13}C NMR (101 MHz, $CDCl_3$)** δ 172.3 (1C, C=O), 158.9 (1C, C Ar), 145.8 (1C, C Ar), 139.0 (1C, C Ar), 133.9 (1C, C Ar), 133.4 (1C, C-2), 132.9 (1C, C Ar), 130.6 (1C, C Ar), 128.8 (2C, CH Ar), 128.2 (1C, C-10), 128.0 (2C, CH Ar), 127.6 (1C, CH Ar), 127.5 (1C, C-1), 127.3 (2C, C-12), 115.9 (1C, C-7), 114.0 (2C, C-13), 113.1 (1C, C-9), 55.4 (1C, OMe), 52.2 (1C, CO_2Me), 49.1 (1C, CH_2 Bn), 38.9 (1C, C-3), 37.4 (1C, C-15), 21.2 (1C, C-4). **HRMS** (ESI): calculated for $C_{27}H_{30}NO$ $[M+H]^+$ requires *m/z* 416.2220, found 416.2208.

(*E*)-*N*-Benzyl-3-ethynyl-4-[4-(4-methoxyphenyl)but-3-en-2-yl]aniline, 4m.

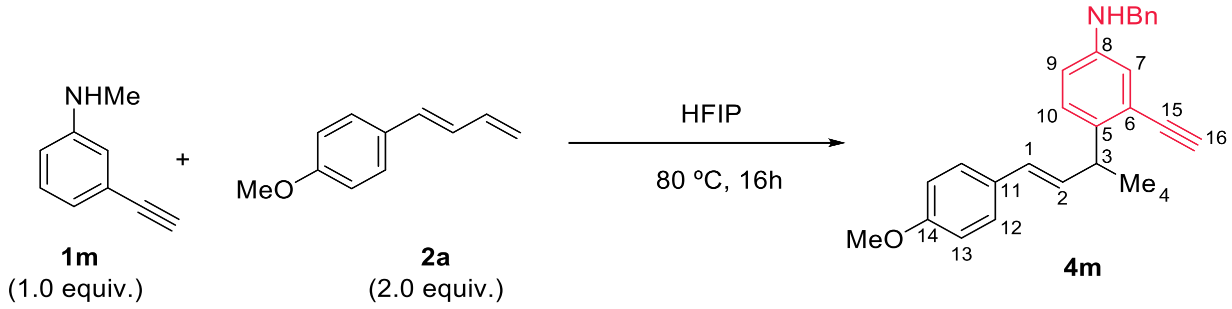

From aniline **1m** (44.7 mg, 0.2 mmol) and diene **2a** (64.1 mg, 0.4 mmol), in 1.0 mL of HFIP, following the general procedure, aniline **4m** was obtained. Chromatographic purification (gradient elution: 0:100 → 20:80 Et_2O – hexane) gave aniline **4m** (39.0 mg, 53%) as a colourless oil.

Data for **4m**: **R*f*** 0.30 (30% Et_2O – hexane). **^{1}H NMR (400 MHz, $CDCl_3$)** δ 7.41 – 7.29 (5H, m, Ar), 7.28 (2H, d, *J* = 8.7 Hz, 12-H), 7.07 (1H, d, *J* = 8.5 Hz, 10-H), 6.84 (1H, s, 7-H), 6.83 (2H, d, *J* = 8.8 Hz, 13-H), 6.66 (1H, dd, *J* = 8.5 and 2.7 Hz, 9-H), 6.36 (1H, dd, *J* = 15.8 and 1.4 Hz, 1-H), 6.25 (1H, dd, *J* = 16.0 and 6.0 Hz, 2-H), 4.30 (2H, s, CH_2 Bn), 4.11 (1H, quint, *J* = 7.2 Hz, 3-H), 3.79 (3H, s, OMe), 3.22 (1H, s, 16-H), 1.40 (3H, d, *J* = 7.0 Hz, Me). **^{13}C NMR (101 MHz, $CDCl_3$)** δ 158.9 (1C, C Ar), 145.4 (1C, C Ar), 138.7 (1C, C Ar), 138.5 (1C, C Ar), 132.9 (1C, C-2), 130.8 (1C, C Ar), 128.8 (2C, CH Ar), 127.9 (2C, CH Ar), 127.7 (1C, CH Ar), 127.6 (1C, C-1), 127.44 (1C, C-10), 127.35 (2C, C-12), 121.8 (1C, C Ar), 117.3 (1C, C-7), 115.3 (1C, C-9), 114.0 (2C, C-13), 82.8 (1C, C-15), 80.6 (1C, C-16), 55.4 (1C, OMe), 49.0 (1C, CH_2 Bn), 38.9 (1C, C-3), 20.9 (1C, C-4). **HRMS** (ESI): calculated for $C_{26}H_{26}F_3NO$ $[M+H]^+$ requires *m/z* 368.2009, found 368.2008.

(*E*)-5-Amino-2-[4-(4-methoxyphenyl)but-3-en-2-yl]phenol, 4n.

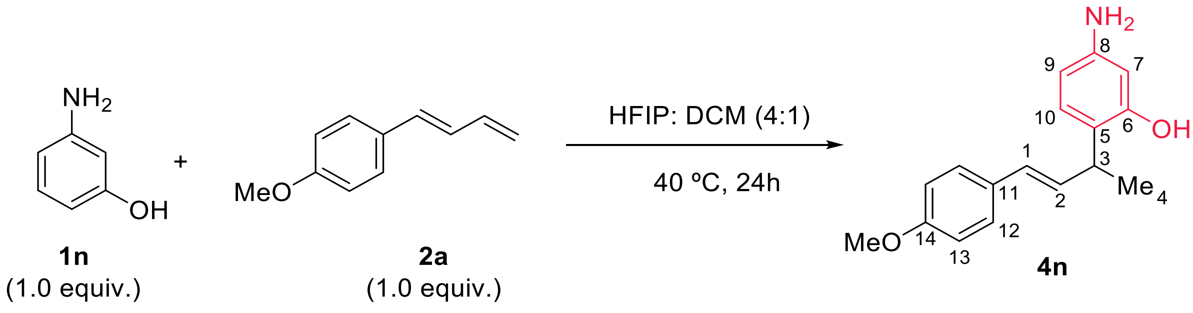

From aniline **1n** (87.3 mg, 0.8 mmol) and diene **2a** (128 mg, 0.8 mmol), in 4.0 mL of HFIP : DCM (4:1), following the general procedure (at 40 ºC for 24h), aniline **4n** was obtained. Chromatographic purification (gradient elution: 20:100 → 40:60 EtOAc – hexane) gave aniline **4n** (96.7 mg.0 mg, 45%) as a yellow oil.

Data for **4n**: **R*f*** 0.15 (50% EtOAc – hexane). **^{1}H NMR (400 MHz, CD_3OD)** 7.24 (2H, d, *J* = 8.8 Hz, 12-H), 6.86 (1H, d, *J* = 7.8 Hz, 10-H), 6.80 (2H, d, *J* = 8.8 Hz, 13-H), 6.33 – 6.17 (4H, m, 1-

H, 2-H, 7-H and 10-H), 3.89 – 3.81 (1H, m, 3-H), 3.74 (3H, s, OMe), 1.33 (3H, d, J = 7.0 Hz, Me).^{13}C **NMR (101 MHz, CD_3OD)** δ 160.1 (1C, C Ar), 156.1 (1C, C Ar), 147.2 (1C, C Ar), 134.6 (1C, C-1), 132.3 (1C, C Ar), 129.0 (1C, C-10), 128.14 (1C, C-2), 128.05 (2C, C-12), 124.0 (1C, C Ar), 114.8 (2C, C-13), 108.8 (1C, C-9), 104.3 (1C, C-7), 55.6 (1C, OMe), 36.0 (1C, C-3), 20.9 (1C, Me).

(*E*)-4-[1-(4-Methoxyphenyl)pent-1-en-3-yl]-*N*-methylaniline, 4o.

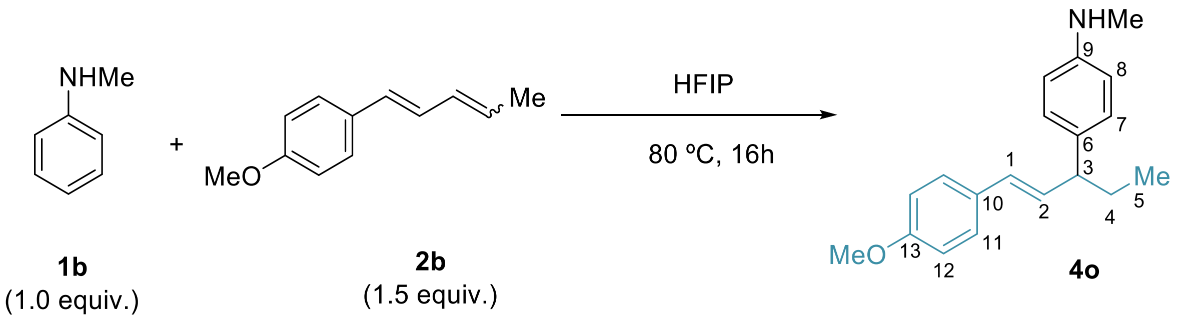

From aniline **1b** (21.4 mg, 0.2 mmol) and diene **2b** (52.3 mg, 0.3 mmol), in 1.0 mL of HFIP, following the general procedure, aniline **4o** was obtained. Chromatographic purification (0:100 → 20:80 Et_2O – hexane) gave aniline **4o** (41.1 mg, 73%, rr 16:1) as a colourless oil.

Data for **4o**: **R*f*** 0.20 (30% Et_2O – hexane). **^{1}H NMR (400 MHz, $CDCl_3$)** δ 7.30 (2H, d, J = 8.7 Hz, 11-H), 7.10 (2H, d, J = 8.5 Hz, 7-H), 6.85 (2H, d, J = 8.8 Hz, 12-H), 6.62 (2H, d, J = 8.5 Hz, 8-H), 6.34 (1H, d, J = 15.8 Hz, 1-H), 6.20 (1H, dd, J = 15.8 and 7.7 Hz, 2-H), 3.81 (3H, s, OMe), 3.21 (1H, q, J = 7.5 Hz, 3-H), 2.84 (3H, s, NMe), 1.80 (2H, m, 4-H_2), 0.93 (3H, t, J = 7.3 Hz, 5-H_3). **^{13}C NMR (101 MHz, $CDCl_3$)** δ 158.8 (1C, C Ar), 147.6 (1C, C Ar), 133.7 (1C, C Ar), 133.1 (1C, C-2), 130.8 (1C, C Ar), 128.5 (2C, C-7), 128.2 (1C, C-1), 127.3 (2C, C-11), 114.0 (2C, C-12), 112.8 (2C, C-8), 55.4 (1C, OMe), 50.1 (1C, C-3), 31.1 (1C, NMe), 29.0 (1C, C-4), 12.5 (1C, C-5). **HRMS** (ESI): calculated for $C_{19}H_{24}NO$ $[M+H]^+$ requires *m/z* 282.1852, found 282.1847.

(*E*)-4-[4-(2-Methoxyphenyl)but-3-en-2-yl]-*N*-methylaniline, 4p.

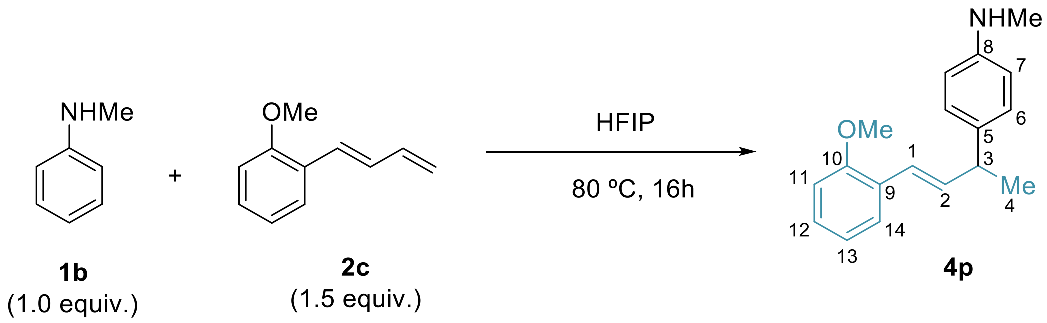

From aniline **1b** (21.4 mg, 0.2 mmol) and diene **2c** (48.1 mg, 0.3 mmol), in 1.0 mL of HFIP, following the general procedure, aniline **4p** was obtained. Chromatographic purification (0:100 → 20:80 Et_2O – hexane) gave aniline **4p** (43.3 mg, 82%) as a colourless oil.

Data for **4p**: **R*f*** 0.20 (30% Et_2O – hexane). **^{1}H NMR (400 MHz, $CDCl_3$)** δ 7.45 (1H, dd, *J* = 7.6 and 1.7 Hz, 14-H), 7.20 (1H, ddd, *J* = 8.2, 7.4 and 1.7 Hz, 12-H), 7.15 (2H, d, *J* = 8.4 Hz, 6-H), 6.92 (1H, dd, *J* = 7.6 and 1.2 Hz, 13-H), 6.87 (1H, dd, *J* = 8.2 and 1.2 Hz, 11-H), 6.79 (1H, dd, *J* = 16.1 and 1.4 Hz, 1-H), 6.63 (2H, d, *J* = 8.6 Hz, 7-H), 6.38 (1H, dd, *J* = 16.0 and 7.1 Hz, 2-H), 3.86 (3H, s, OMe), 3.60 (1H, quintd, *J* = 6.9 and 1.4 Hz, 3-H), 2.85 (3H, s, NMe), 1.46 (3H, d, *J* = 7.0 Hz, Me). **^{13}C NMR (101 MHz, $CDCl_3$)** δ 156.5 (1C, C Ar), 147.5 (1C, C Ar), 136.6 (1C, C-2), 135.2 (1C, C Ar), 128.1 (2C, C-6), 128.0 (1C, C-12), 126.9 (1C, C Ar), 126.5 (1C, C-14), 122.5 (1C, C-1), 120.7 (1C, C-13), 113.0 (2C, C-7), 110.9 (1C, C-11), 55.6 (1C, OMe), 42.2 (1C, C-3), 31.3 (1C, NMe), 21.5 (1C, C-4). **HRMS** (ESI): calculated for $C_{18}H_{22}NO$ $[M+H]^+$ requires *m/z* 268.1696, found 268.1693

(*E*)-*N*-Methyl-4-[4-(*p*-tolyl)but-3-en-2-yl]aniline, 4q.

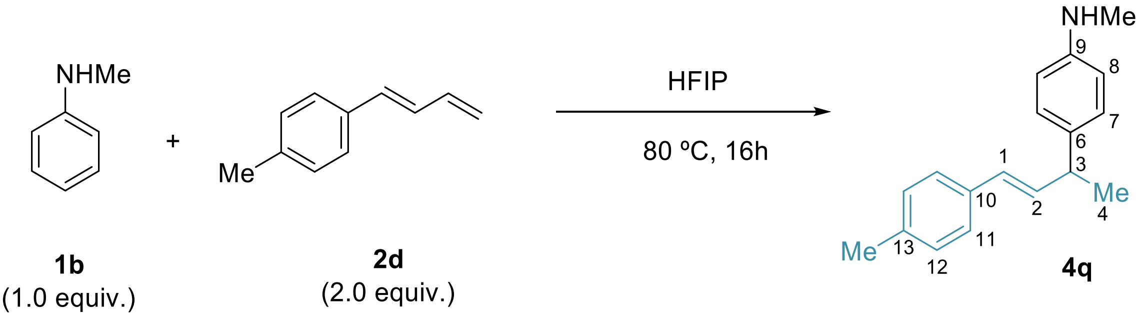

1b (1.0 equiv.) **2d** (2.0 equiv.)

From aniline **1b** (21.4 mg, 0.2 mmol) and diene **2d** (57.7 mg, 0.4 mmol), in 1.0 mL of HFIP, following the general procedure, aniline **4q** was obtained. Chromatographic purification (gradient elution: 0:100 → 20:80 Et_2O – hexane) gave aniline **4q** (23 mg, 45%, rr 16:1) as a colourless oil.

Data for **4q**: **R*f*** 0.20 (30% Et_2O – hexane). **^{1}H NMR (400 MHz, $CDCl_3$)** δ 7.26 (2H, d, *J* = 8.2 Hz, 11-H), 7.22 – 7.02 (4H, m, Ar), 6.64 (2H, d, *J* = 8.5 Hz, 12-H), 6.37 (1H, d, *J* = 15.9 Hz, 1-H), 6.32 (1H, dd, *J* = 15.9 and 5.6 Hz, 2-H), 3.64 – 3.48 (1H, m, 3-H), 2.85 (3H, s, NMe), 2.33 (3H, s, Me), 1.43 (3H, d, *J* = 7.1 Hz, Me). **^{13}C NMR (101 MHz, $CDCl_3$)** δ 146.6 (1C, C Ar), 136.7 (1C, C Ar), 135.9 (1C, C Ar), 135.1 (1C, C Ar), 135.0 (1C, C-2), 129.3 (2C, CH Ar), 128.3 (2C, CH Ar), 128.0 (1C, C-1), 126.1 (2C, C-11), 113.7 (2C, C-12), 41.8 (1C, NMe), 31.8 (1C, C-3), 21.5 (1C, C-4), 21.3 (1C, Me). **HRMS** (ESI): calculated for $C_{18}H_{22}N$ $[M+H]^+$ requires *m/z* 252.1747, found 252.1744.

(*E*)-4-{4-[(1,1'-biphenyl)-4-yl]but-3-en-2-yl}-*N*-methylaniline, 4r.

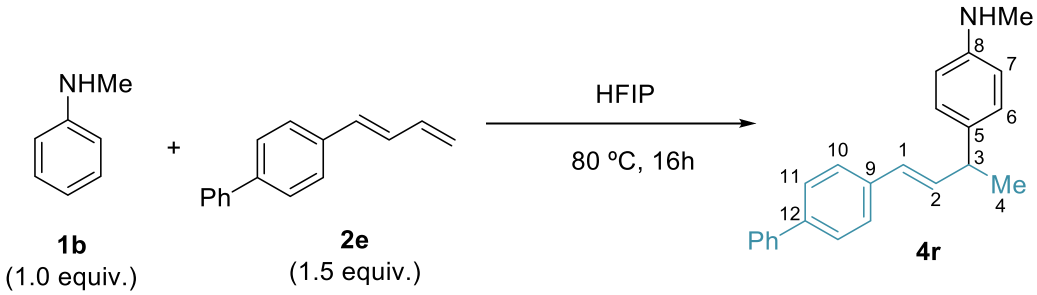

From aniline **1b** (21.4 mg, 0.2 mmol) and diene **2e** (61.9 mg, 0.3 mmol), in 1.0 mL of HFIP, following the general procedure, aniline **4r** was obtained. Chromatographic purification (0:100 → 12:88 Et_2O – hexane) gave aniline **4r** (16.6 mg, 27%, rr 3:1) as a colourless oil.

Data for **4r**: **R_f** 0.15 (20% Et_2O – hexane). **^{1}H NMR (400 MHz, $CDCl_3$)** δ 7.71 – 7.49 (4H, m, Ar), 7.47 – 7.40 (4H, m, Ar), 7.39 – 7.31 (1H, m, Ar), 7.13 (2H, d, *J* = 8.5 Hz, 6-H), 6.62 (2H, d, *J* = 8.7 Hz, 7-H), 6.47 – 6.26 (2H, m, 1-H and 2-H), 3.69 – 3.50 (1H, m, 3-H), 2.85 (3H, s, NMe), 1.46 (3H, d, *J* = 7.0 Hz, 4-H_3). **^{13}C NMR (101 MHz, $CDCl_3$)** 147.9 (1C, C Ar), 141.0 (1C, C Ar), 139.7 (1C, C Ar), 137.0 (1C, C Ar), 136.5 (1C, C-2), 134.4 (1C, C Ar), 128.9 (2C, CH Ar), 128.2 (2C, CH Ar), 127.5 (1C, C-1), 127.3 (2C, CH Ar), 127.2 (1C, CH Ar), 127.0 (2C, CH Ar), 126.7 (2C, CH Ar), 112.7 (2C, CH Ar), 41.8 (1C, C-3), 31.1 (1C, NMe), 21.4 (1C, C-4).

(*E*)-*N*-Methyl-4-(4-(thiophen-2-yl)but-3-en-2-yl)aniline, 4s.

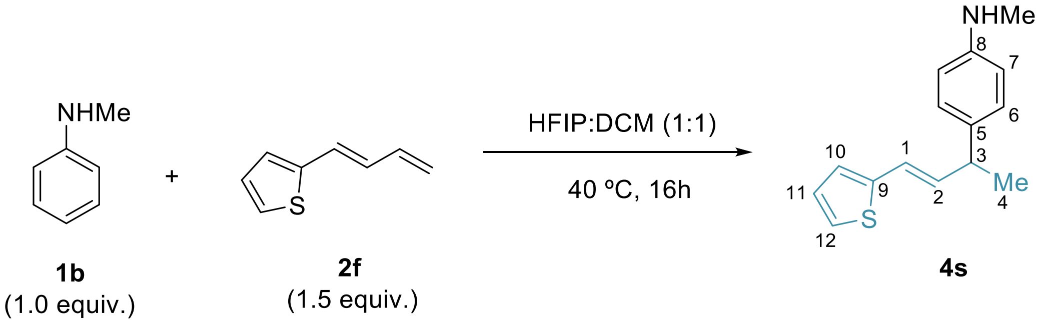

From aniline **1b** (21.4 mg, 0.2 mmol) and diene **2f** (40.9 mg, 0.3 mmol), in 1.0 mL of HFIP: DCM (1:1) at 40 °C, following the general procedure, aniline **4s** was obtained. Chromatographic purification (0:100 → 20:80 Et_2O – hexane) gave aniline **4s** (25.1 mg, 52%, rr 13:1) as yellow oil.

Data for **4s**: **R_f** 0.20 (20% Et_2O – hexane). **^{1}H NMR (400 MHz, $CDCl_3$)** δ 7.05 – 6.95 (3H, m, 6-H and 12-H), 6.83 (dd, *J* = 5.1 and 3.5 Hz, 11-H), 6.79 (1H, d, *J* = 3.5 Hz, 10-H), 6.50 (2H, d, *J* = 8.4 Hz, 7-H), 6.40 (1H, dd, *J* = 15.8 and 1.5 Hz, 1-H), 6.14 (1H, dd, *J* = 15.7 and 6.6 Hz, 2-H), 3.52 (1H, s, NH), 3.50 – 3.32 (1H, m, 3-H), 2.74 (3H, s, NMe), 1.32 (3H, d, *J* = 7.0 Hz, 4-H_3).**^{13}C NMR (101 MHz, $CDCl_3$)** δ 147.9 (1C, C Ar), 143.3 (1C, C Ar), 136.2 (1C, C-2), 134.0 (1C, C Ar), 128.2

(2C, C-6), 127.3 (1C, C-11), 124.6 (1C, C-10), 123.3 (1C, C-12), 121.4 (1C, C-1), 112.7(2C, C-7), 41.6 (1C,-3), 31.1 (1C, NMe), 21.3 (C-4).

(*E*)-*N*-Methyl-4-(4-phenylpent-3-en-2-yl)aniline, 4t.

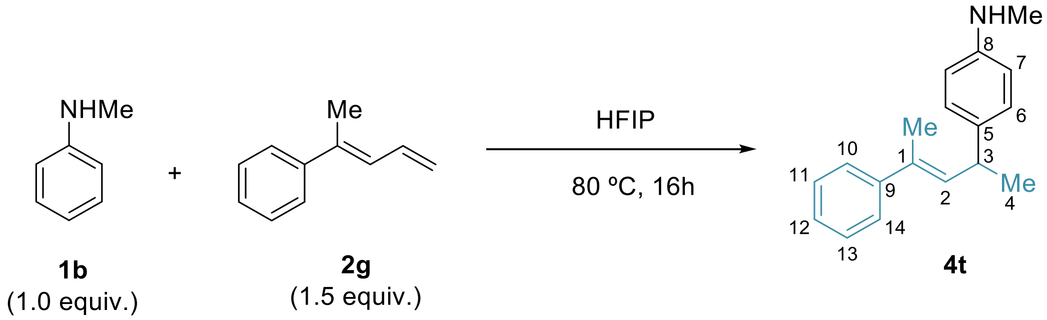

From aniline **1b** (21.4 mg, 0.2 mmol) and diene **2g** (43.3 mg, 0.3 mmol), in 1.0 mL of HFIP, following the general procedure, aniline **4t** was obtained. Chromatographic purification (0:100 → 12:88 Et_2O – hexane) gave aniline **4t** (25.2 mg, 50%) as a colourless oil.

Data for **4t**: **R*f*** 0.20 (20% Et_2O – hexane). **^{1}H NMR (400 MHz, $CDCl_3$)** δ 7.42 (2H, d, *J* = 7.2 Hz, Ar), 7.32 (2H, t, *J* = 7.6 Hz, Ar), 7.26 – 7.20 (1H, m, Ar), 7.15 (2H, d, *J* = 8.4 Hz, 6-H), 6.61 (2H, d, *J* = 8.5 Hz, 7-H), 5.92 (1H, dd, *J* = 9.3 and 1.4 Hz, 2-H), 3.79 (1H, dq, *J* = 9.3 and 6.9 Hz, 3-H), 3.62 (1H, s, NH), 2.84 (3H, s, NMe), 2.12 (3H, d, *J* = 1.4 Hz, 1-Me), 1.41 (3H, d, *J* = 6.9 Hz, 4-H_3). **^{13}C NMR (101 MHz, $CDCl_3$)** δ 147.7 (1C, C Ar), 144.1 (1C, C Ar), 135.5 (1C, C Ar), 134.3 (1C, C-2), 133.2 (1C, C Ar), 128.2 (2C, CH Ar), 127.8 (2C, C-6), 126.7 (1C, CH Ar), 125.9 (2C, CH Ar), 112.8 (2C, C-7), 37.8 (1C, C-3), 31.1 (1C, NMe), 22.6 (1C, C-4), 16.1 (1C, Me).

***N*-Benzyl-3-bromo-4-(4,4-diphenylbut-3-en-2-yl)aniline, 4u.**

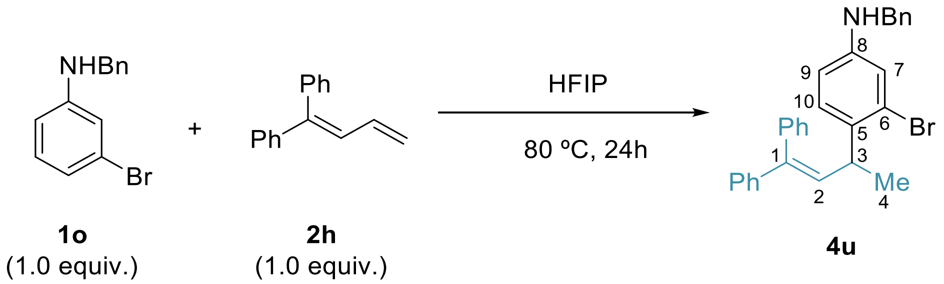

From aniline **1o** (210 mg, 0.8 mmol) and diene **2h** (165 mg, 0.8 mmol), in 4.0 mL of HFIP, following the general procedure, aniline **4u** was obtained. Chromatographic purification (gradient elution: 0:100 → 10:90 Et_2O – hexane) gave aniline **4u** (270 mg, 72%) as a yellow oil.

Data for **4u**: **R*f*** 0.15 (5% Et_2O – hexane). **^{1}H NMR (400 MHz, $CDCl_3$)** δ 7.29 – 7.18 (8H, m, Ar), 7.17 – 7.10 (5H, m, Ar), 7.08 – 7.03 (3H, m, Ar), 6.72 (1H, d, *J* = 2.5 Hz, 7-H), 6.47 (1H, dd, *J* = 8.5 and 2.5 Hz, 9-H), 6.13 (1H, d, *J* = 9.9 Hz, 2-H), 4.18 (2H, s, CH_2 Bn), 3.97 – 3.73 (2H, m, 1-H), 1.22 (3H, d, *J* = 6.8 Hz, Me). **^{13}C NMR (101 MHz, $CDCl_3$)** δ 147.3 (1C, C Ar), 142.9 (1C, C

Ar), 140.9 (1C, C Ar), 140.1 (1C, C-1), 139.1 (1C, C Ar), 134.6 (1C, C Ar), 133.6 (1C, C-2), 130.0 (2C, CH Ar), 128.8 (2C, CH Ar), 128.5 (1C, CH Ar), 128.22 (2C, CH Ar), 128.17 (2C, CH Ar), 127.7 (2C, CH Ar), 127.6 (2C, CH Ar), 127.5 (1C, CH Ar), 127.13 (1C, CH Ar), 127.09 (1C, CH Ar), 124.4 (1C, C Ar), 116.7 (1C, C-7), 112.8 (1C, C-9), 48.4 (1C, CH_2 Bn), 38.0 (1C, C-3), 23.1 (1C, Me). **HRMS** (ESI): calculated for $C_{29}H_{27}BrN$ $[M+H]^+$ requires *m/z* 468.1321 and 470.1305, found 468.1315 and 470.1303.

***N*-Methyl-1',4',5',6'-tetrahydro-[1,1':3',1''-terphenyl]-4-amine, 4v.**

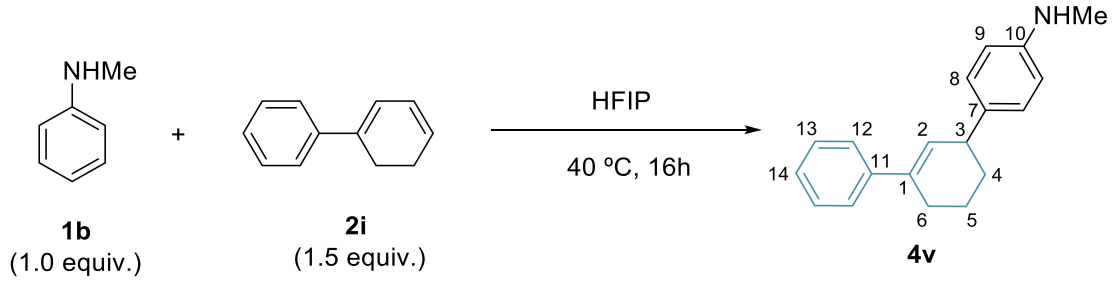

From aniline **1b** (21.4 mg, 0.2 mmol) and diene **2i** (46.9 mg, 0.3 mmol), in 1.0 mL of HFIP, following the general procedure, aniline **4v** was obtained. Chromatographic purification (0:100 → 20:80 Et_2O – hexane) gave aniline **4v** (11.7 mg, 22%) as a colourless oil.

Data for **4v**: $\mathbf{R_f}$ 0.20 (20% Et_2O – hexane). **^{1}H NMR (400 MHz, $CDCl_3$)** δ 7.46 (2H, dd, *J* = 8.3, 1.3 Hz, Ar), 7.33 (2H, t, *J* = 7.5 Hz, Ar), 7.26 – 7.21 (1H, m, Ar), 7.09 (2H, d, *J* = 8.5 Hz, 8-H), 6.60 (2H, d, *J* = 8.5 Hz, 9-H), 6.17 (1H, dt, *J* = 3.2 and 1.7 Hz, 2-H), 3.65 (1H, s, NH), 3.55 – 3.45 (1H, m, 3-H), 2.84 (3H, s, NMe), 2.50 (2H, m, 4-H_2), 2.11 – 1.99 (1H, m, 6-H_A), 1.99 – 1.87 (1H, m, 5-H_A), 1.82 – 1.68 (1H, m, 5-H_B), 1.65 – 1.52 (1H, m, 6-H_B). **^{13}C NMR (101 MHz, $CDCl_3$)** δ 147.9 (1C, C Ar), 142.5 (1C, C Ar), 137.2 (1C, C Ar), 135.6 (1C, C Ar), 128.74 (2C, C-8), 128.67 (1C, C-2), 128.4 (2C, CH Ar), 126.9 (1C, CH Ar), 125.3 (2C, CH Ar), 112.6 (2C, C-9), 41.9 (1C, C-3), 32.5 (1C, C-6), 31.1 (1C, NMe), 27.5 (1C, C-4), 21.9 (1C, C-5).

5. General procedure for the trifluoromethylarylation of dienes using anilines in HFIP.

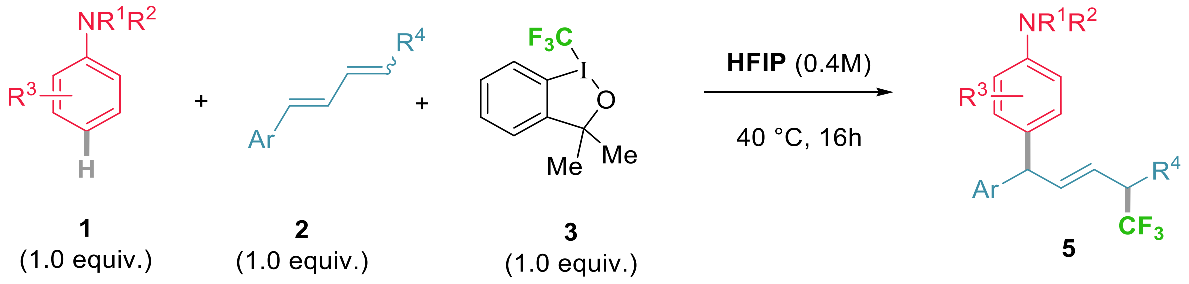

Aniline **1** (1.0 equiv.) and alkene **2** (1.0 equiv.) were placed in an oven-dry 10.0 mL vial and hexafluoroisopropanol (0.4 M) was added, followed by trifluoromethyl reagent **3** (1.0 equiv.). The vial was sealed, purged with Argon and set in a preheated heating block overnight at 40 ºC, unless

other conditions stated. The reaction mixture was cooled down, the solvent was evaporated under reduced pressure and the crude was purified by chromatography on silica gel using the appropriate mixture of eluents to give the corresponding product, **5**.

(*E*)-*N*-Benzyl-4-[5,5,5-trifluoro-1-(4-methoxyphenyl)pent-2-en-1-yl]aniline, 5a.

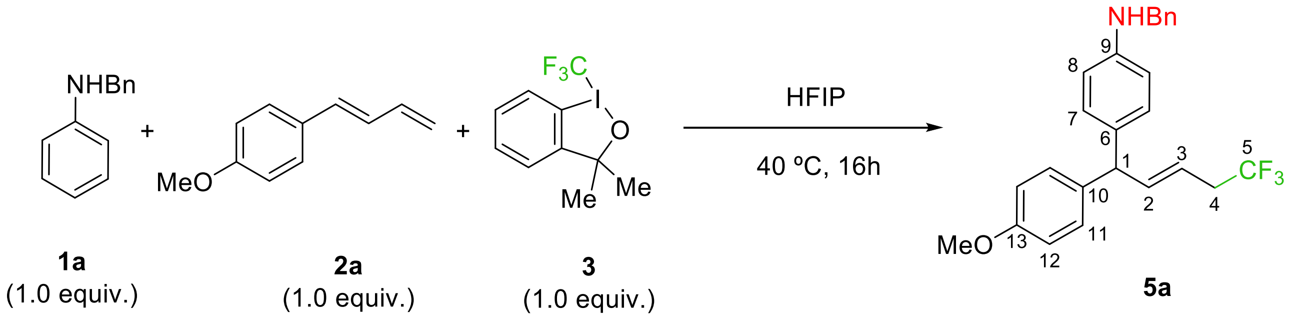

From aniline **1a** (36.6 mg, 0.2 mmol), alkene **2a** (32.0 mg, 0.2 mmol) and trifluoromethyl reagent **3** (66.0 mg, 0.2 mmol), in 0.5 mL of HFIP, following the general procedure, aniline **5a** was obtained. Chromatographic purification (gradient elution: 0:100 → 15:85 Et_2O – hexane) gave **5a** (44.9 mg, 55%), as a colourless oil.

Data for **5a**: $\boldsymbol{R_f}$ 0.40 (30% Et_2O – hexane). **^{1}H NMR (400 MHz, $CDCl_3$)** δ 7.39 – 7.26 (5H m, Ar), 7.06 (2H, d, J = 8.6 Hz, 11-H), 6.96 (2H, d, J = 8.5 Hz, 7-H), 6.83 (2H, d, J = 8.7 Hz, 12-H), 6.65 (2H, d, J = 8.5 Hz, 8-H), 6.12 (1H, dd, J = 15.4 and 7.3 Hz, 2-H), 5.33 (1H, dtd, J = 15.4, 7.1 and 1.3 Hz, 3-H), 4.60 (1H, d, J = 7.3 Hz, 1-H), 4.30 (2H, s, CH_2 Bn), 3.78 (3H, s, OMe), 2.84 (2H, qd, J = 10.6 and 7.2 Hz, 4-H_2). **^{13}C NMR (101 MHz, $CDCl_3$)** δ 158.2 (1C, C Ar), 146.1 (1C, C Ar), 141.2 (1C, C-2), 139.0 (1C, C Ar), 135.7 (1C, C Ar), 132.9 (1C, C Ar), 129.5 (2C, CH Ar), 129.4 (2C, CH Ar), 128.8 (2C, CH Ar), 127.9 (2C, CH Ar), 127.5 (1C, CH Ar), 126.0 (1C, q, J = 276.7 Hz, CF_3), 118.86 (1C, C-3), 113.9 (2C, CH Ar), 113.6 (2C, CH Ar), 55.4 (1C, OMe), 52.3 (1C, C-1), 49.0 (1C, CH_2 Bn), 37.4 (1C, q, J = 29.6 Hz, C-4). **^{19}F NMR (376 MHz, $CDCl_3$)** δ –66.4 (3F, CF_3). **HRMS** (EI): calculated for $C_{25}H_{24}F_3NO$ $[M+H]^+$ requires *m/z* 412.1883, found 412.1885.

(*E*)-*N*-Benzyl-2-methyl-4-[5,5,5-trifluoro-1-(4-methoxyphenyl)pent-2-en-1-yl]aniline, 5b.

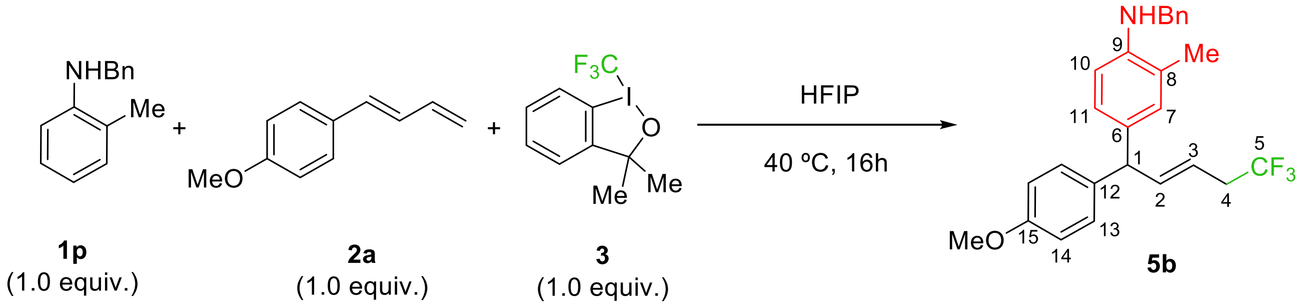

From aniline **1p** (39.5 mg, 0.2 mmol), alkene **2a** (32.0 mg, 0.2 mmol) and trifluoromethyl reagent **3** (66.0 mg, 0.2 mmol), in 0.5 mL of HFIP, following the general procedure, aniline **5b** was

obtained. Chromatographic purification (gradient elution: 0:100 → 10:90 Et_2O – hexane) gave **5b** (49.1 mg, 55%, rr 11:1), as a colourless oil.

Data for **5b**: R_f 0.45 (30% Et_2O – hexane). **^{1}H NMR (400 MHz, CDCl$_3$)** δ 7.47 – 7.30 (5H, m, Ar), 7.11 (2H, d, J = 8.4 Hz, 13-H), 6.91 (1H, d, J = 7.8 Hz, 11-H), 6.90 (1H, s, 7-H), 6.86 (2H, d, J = 8.8 Hz, 14-H), 6.60 (1H, d, J = 8.1 Hz, 10-H), 6.16 (1H, dd, J = 15.4 and 7.5 Hz, 2-H), 5.37 (1H, dtd, J = 15.4, 7.2 and 1.4 Hz, 3-H), 4.62 (1H, d, J = 7.4 Hz, 1-H), 4.38 (2H, s, CH_2 Bn), 3.81 (3H, s, OMe), 3.58 (1H, s, NH), 2.86 (2H, qd, J = 10.7 and 7.2 Hz, 4-H_2), 2.16 (3H, s, Me). **^{13}C NMR (101 MHz, CDCl$_3$)** δ 158.2 (1C, C Ar), 144.8 (1C, C Ar), 141.4 (1C, C-2), 139.6 (1C, C Ar), 135.9 (1C, C Ar), 131.9 (1C, C Ar), 130.4 (1C, CH Ar), 129.5 (2C, C-13), 128.8 (2C, CH Ar), 127.7 (2C, CH Ar), 127.4 (1C, CH Ar), 127.1 (1C, CH Ar), 126.2 (1C, q, J = 276.0 Hz, CF_3), 122.3 (1C, C Ar), 118.7 (1C, q, J = 3.4 Hz, C-3), 113.9 (2C, C-14), 110.1 (1C, C-10), 55.4 (1C, OMe), 52.3 (1C, C-1), 48.6 (1C, CH_2 Bn), 37.4 (1C, q, J = 29.6 Hz, C-4), 17.8 (1C, Me). **^{19}F NMR (376 MHz, CDCl$_3$)** δ –66.3 (3F, CF_3). **HRMS** (ESI): calculated for $C_{26}H_{27}F_3NO$ $[M+H]^+$ requires m/z 426.2039, found 426.2032.

(*E*)-6-[5,5,5-Trifluoro-1-(4-methoxyphenyl)pent-2-en-1-yl]-1,2,3,4-tetrahydroquinoline, 5c.

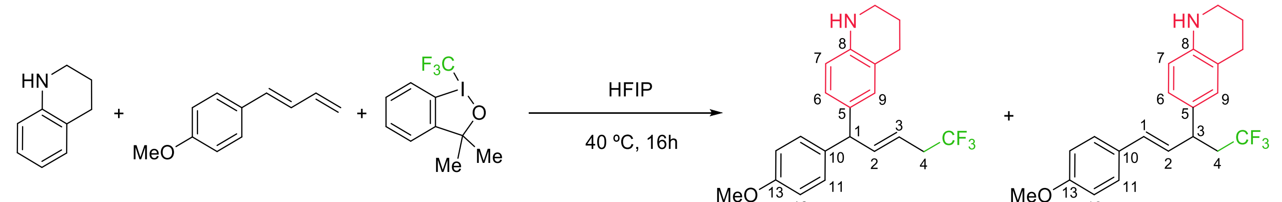

From aniline **1q** (26.6 mg, 0.2 mmol), alkene **2a** (32.1 mg, 0.2 mmol) and trifluoromethyl reagent **3** (66.0 mg, 0.2 mmol), in 0.5 mL of HFIP, following the general procedure, aniline **5c** was obtained. Chromatographic purification (gradient elution: 0:100 → 5:95 Et_2O – hexane) gave **5c** (37.9 mg, 55%, rr 3:1), as a colourless oil.

Data for **5c** (major regioisomer, from the mixture): R_f 0.30 (5% Et_2O – hexane). **^{1}H NMR (400 MHz, CDCl$_3$)** δ 7.06 (2H, d, J = 8.5 Hz, 11-H), , 6.88 – 6.80 (3H, m, Ar), 6.76 – 6.71 (1H, m, Ar), 6.48 – 6.39 (1H, m, Ar), 6.12 (1H, dd, J = 15.3 and 7.5 Hz, 2-H), 5.34 (1H, dtd, J = 15.5, 7.2 and 1.4 Hz, 3-H) 4.55 (1H, d, J = 7.4 Hz, 1-H), 3.79 (3H, s, OMe), 3.38 – 3.13 (2H, m, CH_2 tetrahydroquinoline), 2.93 – 2.79 (2H, m, 4-H_2), 2.78 – 2.68 (2H, m, CH_2 tetrahydroquinoline), 2.04–1.86 (2H, m, CH_2 tetrahydroquinoline). **^{13}C NMR (101 MHz, CDCl$_3$)** δ 158.1 (1C, C Ar), 143.4 (1C, C Ar), 141.4 (1C, C-2), 135.9 (1C, C Ar), 131.7 (1C, C Ar), 129.6 (1C, CH Ar), 129.5 (2C, CH Ar), 128.5 (1C, CH Ar), 126.17 (1C, q, J = 274.9 Hz, CF_3), 121.6 (1C, C Ar), 118.7 (1C, q, J = 3.5 Hz, C-3), 114.4 (1C, CH Ar), 113.9 (2C, CH Ar), 55.37 (1C, OMe), 52.4 (1C, C-1), 42.2 (1C, NCH_2), 37.4

(1C, q, J = 29.6 Hz, C-4), 27.2 (1C, CH_2 tetrahydroquinoline), 22.34 (1C, CH_2 tetrahydroquinoline). **^{19}F NMR (376 MHz, $CDCl_3$)** δ –66.4 (3F, CF_3). **HRMS** (ESI): calculated for $C_{21}H_{23}F_3NO$ $[M+H]^+$ requires m/z 362.1726, found 362.1723.

Partial data for **5c** (minor regioisomer): The NMR signals overlapped with major isomer, except for: **^{1}H NMR (400 MHz, $CDCl_3$)** δ 7.27 (2H, d, J = 8.9 Hz, 11-H), 6.35 (1H, d, J = 15.8 Hz, 1-H), 3.80 (3H, s, OMe), 3.68 (1H, q, J = 7.5 Hz, 3-H), 2.65 – 2.44 (1H, m, 4-H_2). **^{13}C NMR (101 MHz, $CDCl_3$)** δ 159.1 (1C, C Ar), 143.8 (1C, C Ar), 130.8 (1C, C Ar), 130.2 (1C, C Ar), 130.1 (1C, C-3), 129.35 (1C, C-2), 128.09 (1C, q, J = 276.3 Hz, CF_3), 127.5 (2C, C-12), 126.8 (1C, CH Ar), 121.9 (1C, C Ar), 125.7 (1C, CH Ar), 114.6 (1C, CH Ar), 114.0 (2C, CH Ar), 55.42 (1C, OMe), 42.3 (1C, q, J = 2.6 Hz, C-3), 42.1 (1C, NCH_2), 40.2 (1C, q, J = 26.7 Hz, C-4), 22.28 (1C, CH_2 tetrahydroquinoline). **^{19}F NMR (376 MHz, $CDCl_3$)** δ –63.4 (3F, CF_3).

(*E*)-*N*-Benzyl-5-[5,5,5-trifluoro-1-(4-methoxyphenyl)pent-2-en-1-yl]-(1,1'-biphenyl)-2-amine, 5d.

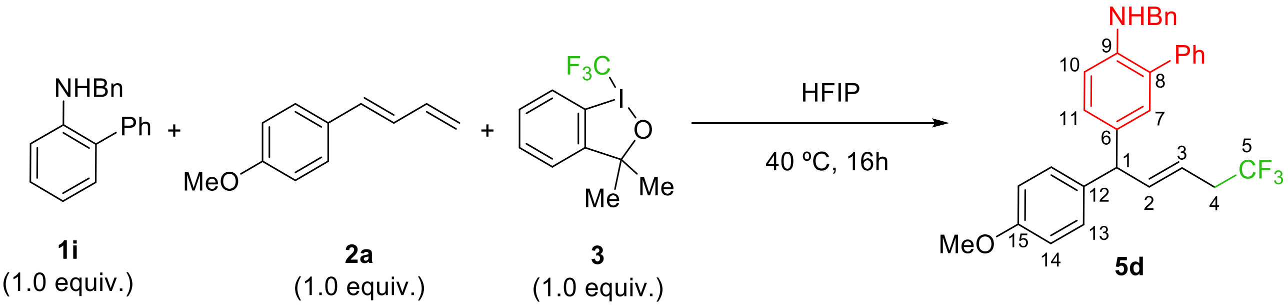

1i (1.0 equiv.) **2a** (1.0 equiv.) **3** (1.0 equiv.) **5d**

From aniline **1i** (51.9 mg, 0.2 mmol), alkene **2a** (32.0 mg, 0.2 mmol) and trifluoromethyl reagent **3** (66.0 mg, 0.2 mmol), in 0.5 mL of HFIP, following the general procedure, aniline **5d** was obtained. Chromatographic purification (gradient elution: 0:100 → 10:90 Et_2O – hexane) gave **5d** (42.0 mg, 43%), as a colourless oil.

Data for **5d**: ***R_f*** 0.50 (30% Et_2O – hexane). **^{1}H NMR (400 MHz, $CDCl_3$)** δ 7.53 – 7.41 (5H, m, Ar), 7.39 – 7.24 (5H, m, Ar), 7.12 (2H, d, J = 8.7 Hz, 13-H), 6.98 (1H, dd, J = 8.3 and 2.3 Hz, 11-H), 6.94 (1H, d, J = 2.2 Hz, 7-H), 6.85 (2H, d, J = 8.7 Hz, 14-H), 6.63 (1H, d, J = 8.3 Hz, 10-H), 6.16 (1H, dd, J = 15.3 and 7.4 Hz, 2-H), 5.39 (1H, dtd, J = 15.5, 7.2 and 1.4 Hz, 3-H), 4.64 (1H, d, J = 7.5 Hz, 1-H), 4.38 (1H, NH), 4.32 (2H, s, CH_2 Bn), 3.80 (3H, s, OMe), 2.85 (2H, qd, J = 10.8 and 7.3 Hz, 4-H_2). **^{13}C NMR (101 MHz, $CDCl_3$)** δ 158.2 (1C, C Ar), 143.6 (1C, C Ar), 141.2 (1C, C Ar), 139.7 (1C, C-2), 139.5 (1C, C Ar), 135.7 (1C, C Ar), 131.2 (1C, C Ar), 130.5 (1C, C-7), 129.52 (2C, CH Ar), 129.49 (2C, CH Ar), 129.1 (2C, CH Ar), 128.7 (2C, CH Ar), 128.6 (1C, C-11), 127.8 (1C, C Ar), 127.4 (1C, CH Ar), 127.24 (2C, CH Ar), 127.20 (1C, CH Ar), 126.2 (1C, q, J = 276.6 Hz, CF_3), 118.9 (1C, q, J = 3.5 Hz, C-3), 113.9 (2C, C-14), 110.9 (1C, C-10), 55.4 (1C, OMe), 52.4 (1C,

C-1), 48.5 (1C, CH_2 Bn), 37.4 (1C, q, J = 29.6 Hz, C-4). **^{19}F NMR (376 MHz, $CDCl_3$)** δ –66.3 (3F, CF_3). **HRMS** (ESI): calculated for $C_{31}H_{29}F_3NO$ $[M+H]^+$ requires *m/z* 488.2196, found 488.2192.

(*E*)-*N*-Benzyl-3-fluoro-4-[5,5,5-trifluoro-1-(4-methoxyphenyl)pent-2-en-1-yl]aniline, 5e.

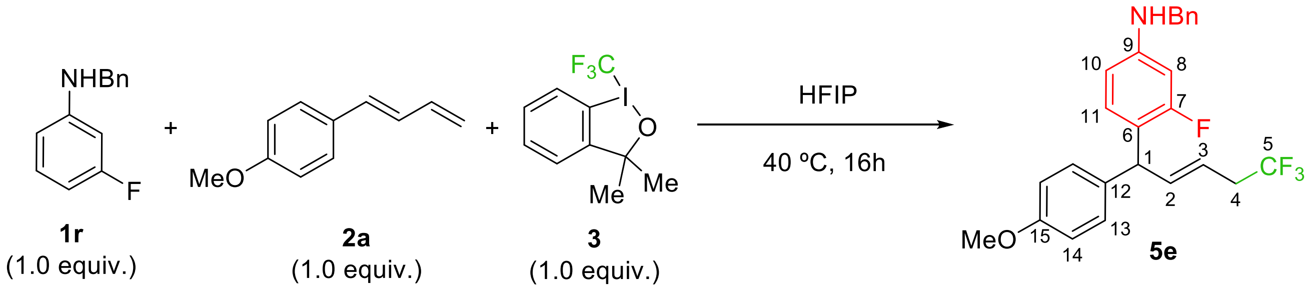

From aniline **1r** (40.3 mg, 0.2 mmol), diene **2a** (32.0 mg, 0.2 mmol) and trifluoromethyl reagent **3** (66.0 mg, 0.2 mmol), in 0.5 mL of HFIP, following the general procedure, aniline **5e** was obtained. Chromatographic purification (gradient elution: 0:100 → 20:80 Et_2O – hexane) gave **5e** (28.6 mg, 33%), as a colourless oil.

Data for **5e**: ***R_f*** 0.15 (15% Et_2O – hexane). **^{1}H NMR (400 MHz, $CDCl_3$)** δ 7.46 – 7.26 (5H, m, Ar), 7.10 (2H, d, J = 8.6 Hz, 13-H), 6.88 (1H, t, J = 8.7 Hz, 11-H), 6.85 (2H, d, J = 8.7 Hz, 14-H), 6.39 (1H, dd, J = 8.3 and 2.4 Hz, 10-H), 6.35 (1H, dd, J = 12.4 and 2.3 Hz, 8-H), 6.13 (1H, dd, J = 15.4 and 6.9 Hz, 2-H), 5.33 (1H, dtd, J = 15.6, 7.2 and 1.5 Hz, 3-H), 4.89 (1H, d, J = 6.8 Hz, 1-H), 4.30 (2H, s, CH_2 Bn), 3.79 (3H, s, OMe), 2.92 – 2.77 (2H, m, 4-H_2). **^{13}C NMR (101 MHz, $CDCl_3$)** δ 161.5 (1C, d, J = 244.5 Hz, C Ar), 158.3 (1C, C Ar), 148.3 (1C, d, J = 10.8 Hz C Ar), 139.8 (1C, C-2), 138.7 (1C, C Ar), 134.5 (1C, C Ar), 130.3 (1C, d, J = 6.5 Hz, C-11), 129.4 (2C, C-13), 128.9 (2C, CH Ar), 127.7 (2C, CH Ar), 127.6 (1C, CH Ar), 126.1 (1C, q, J = 276.7 Hz, CF_3), 119.4 (1C, q, J = 3.7 Hz, C-3), 118.9 (1C, d, J = 15.2 Hz, C Ar), 113.9 (2C, C-14), 109.2 (1C, C-10), 100.2 (1C, d, J = 26.4 Hz, C-8), 55.4 (1C, OMe), 48.7 (1C, CH_2 Bn), 45.4 (1C, d, J = 2.3 Hz. C-1), 37.4 (1C, q, J = 29.6 Hz, C-4). **^{19}F NMR (376 MHz, $CDCl_3$)** δ –66.4 (3F, CF_3), –116.7 (1F, Ar-F). **HRMS** (ESI): calculated for $C_{25}H_{24}F_3NO$ $[M+H]^+$ requires *m/z* 430.1789, found 430.1805.

(*E*)-*N*-Benzyl-3-bromo-4-(5,5,5-trifluoro-1-(4-methoxyphenyl)pent-2-en-1-yl)aniline, 5f.

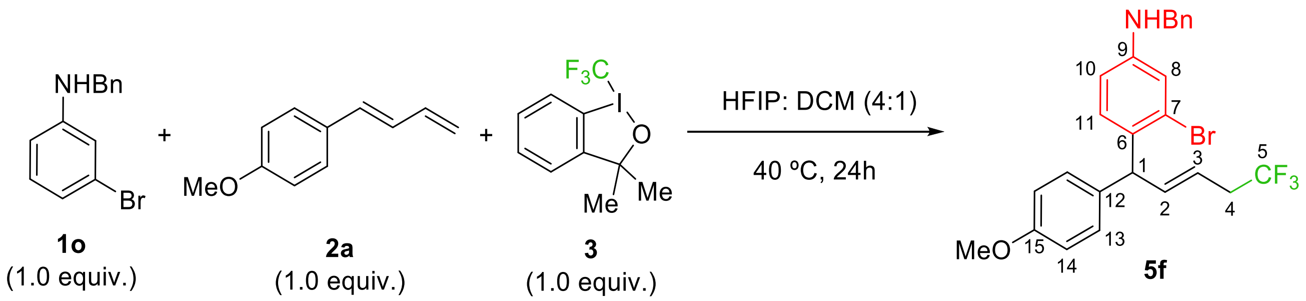

From aniline **1o** (262 mg, 1.0 mmol), alkene **2a** (160 mg, 1.0 mmol) and trifluoromethyl reagent **3** (66.0 mg, 0.2 mmol), in 2.5 mL of HFIP: DCM (4:1), following the general procedure (at 20 ºC for 24h), aniline **5f** was obtained. Chromatographic purification (gradient elution: 10:90 → 20:80 Et_2O – hexane) gave **5f** (300 mg, 61%), as a colourless oil.

Data for **5f**: ***R**$_f$* 0.10 (10% Et_2O – hexane). **^{1}H NMR (400 MHz, $CDCl_3$)** δ 7.41 – 7.26 (5H, m, Ar), 7.07 (2H, d, *J* = 8.6 Hz, 13-H), 6.89 (1H, d, *J* = 8.4 Hz, 11-H), 6.87 (1H, d, *J* = 2.5 Hz, 8-H), 6.84 (2H, d, *J* = 8.8 Hz, 14-H), 6.53 (1H, dd, *J* = 8.4 and 2.6 Hz, 10-H), 6.08 (1H, dd, *J* = 15.4 and 6.4 Hz, 2-H), 5.26 (1H, dtd, *J* = 15.6, 7.2 and 1.6 Hz, 3-H), 5.07 (1H, d, *J* = 6.4 Hz, 1-H), 4.29 (2H, s, CH_2 Bn), 3.79 (3H, s, OMe), 2.92 – 2.77 (2H, m, 4-H_2). **^{13}C NMR (101 MHz, $CDCl_3$)** δ 158.3 (1C, C-15), 147.8 (1C, C Ar), 140.2 (1C, C-2), 138.9 (1C, C Ar), 134.4 (1C, C Ar), 130.7 (1C, C Ar), 130.5 (1C, C-11), 129.8 (2C, C-13), 128.9 (2C, CH Ar), 127.7 (2C, CH Ar), 127.6 (1C, CH Ar), 126.1 (1C, q, *J* = 276.6 Hz, CF_3), 125.7 (1C, C Ar), 119.7 (1C, q, *J* = 3.6 Hz, C-3), 116.7 (1C, C-8), 113.9 (2C, C-14), 112.5 (1C, C-10), 55.4 (1C, OMe), 50.9 (1C, C-1), 48.4 (1C, CH_2 Bn), 37.4 (1C, q, *J* = 29.7 Hz, C-4). **^{19}F NMR (376 MHz, $CDCl_3$)** δ –66.3 (3F, CF_3).

(*E*)-*N*-Benzyl-3-methyl-4-(5,5,5-trifluoro-1-(4-methoxyphenyl)pent-2-en-1-yl)aniline, 5g.

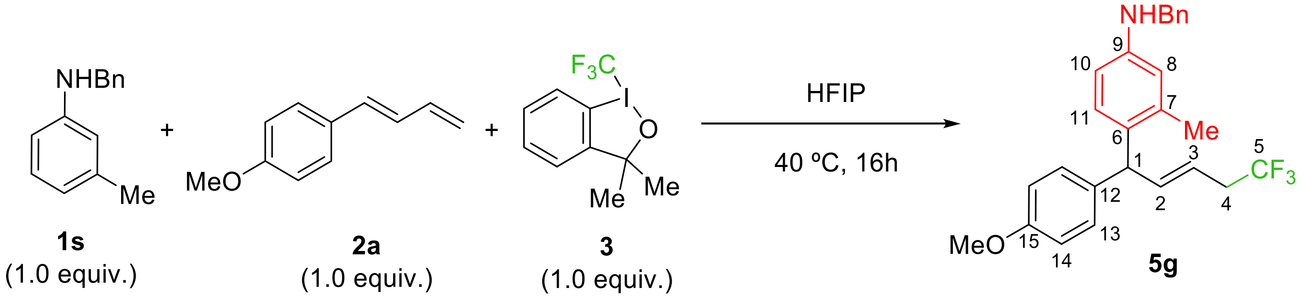

From aniline **1s** (39.5 mg, 0.2 mmol), alkene **2a** (32.1 mg, 0.2 mmol) and trifluoromethyl reagent **3** (66.0 mg, 0.2 mmol), in 0.5 mL of HFIP, following the general procedure, aniline **5g** was obtained. Chromatographic purification (gradient elution: 0:100 → 20:80 Et_2O – hexane) gave **5g** (30.6 mg, 36%, rr 6:1), as a colourless oil.

Data for **5g**: ***R**$_f$* 0.10 (10% Et_2O – hexane). **^{1}H NMR (400 MHz, $CDCl_3$)** δ 7.42 – 7.27 (5H, m, Ar), 7.02 (2H, d, *J* = 8.7 Hz, 13-H), 6.88 (1H, d, *J* = 8.1 Hz, 11-H), 6.82 (2H, dd, *J* = 8.5 Hz, 14-H), 6.56 – 6.48 (2H, m, 8-H and 10-H), 6.11 (1H, dd, *J* = 15.4 and 6.5 Hz, 2-H), 5.20 (1H, dtd, *J* = 15.6, 7.2 and 1.6 Hz, 3-H), 4.77 (1H, d, *J* = 6.5 Hz, 1-H), 4.31 (2H, s, CH_2 Bn), 3.79 (3H, s, OMe), 2.84 (2H, qd, *J* = 10.7 and 7.1 Hz, 4-H_2), 2.16 (3H, s, Me). **^{13}C NMR (101 MHz, $CDCl_3$)** δ 158.1 (1C, C-15), 145.9 (1C, C Ar), 141.1 (1C, C-2), 139.0 (1C, C Ar), 137.5 (1C, C Ar), 134.9 (1C, C Ar), 131.4 (1C, C Ar), 129.8 (2C, CH Ar), 129.3 (1C, C-11), 128.8 (2C, CH Ar), 128.0 (2C, CH Ar), 127.5 (1C, CH Ar), 126.1 (1C, q, *J* = 276.6 Hz, CF_3), 119.0 (1C, q, *J* = 3.5 Hz, C-3), 116.1 (1C, C-8), 113.9

(2C, C-14), 111.2 (1C, C-10), 55.4 (1C, OMe), 49.2 (1C, CH_2 Bn), 48.7 (1C, C-1), 37.4 (1C, q, *J* = 29.7 Hz, C-4), 20.1 (1C, Me). **^{19}F NMR (376 MHz, $CDCl_3$)** δ –66.4 (3F, CF_3). **HRMS** (ESI): calculated for $C_{26}H_{27}F_3NO$ $[M+H]^+$ requires *m/z* 426.2039, found 426.2038.

(*E*)-*N*-Benzyl-5-chloro-2-methoxy-4-[5,5,5-trifluoro-1-(4-methoxyphenyl)pent-2-en-1-yl]aniline, 5h.

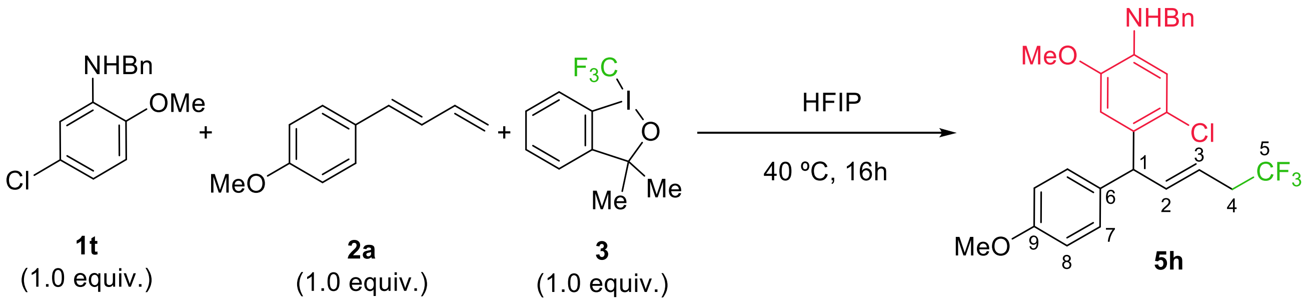

From aniline **1t** (49.5 mg, 0.2 mmol), alkene **2a** (32.1 mg, 0.2 mmol) and trifluoromethyl reagent **3** (66.0 mg, 0.2 mmol), in 0.5 mL of HFIP, following the general procedure, aniline **5h** was obtained. Chromatographic purification (gradient elution: 0:100 → 20:80 Et_2O – hexane) gave **5h** (43.5 mg, 46%), as a colourless oil.

Data for **5h**: ***R_f*** 0.35 (30% Et_2O – hexane). **^{1}H NMR (400 MHz, $CDCl_3$)** δ 7.43 – 7.27 (5H, m, Ar), 7.09 (2H, d, *J* = 8.5 Hz, 7-H), 6.86 (2H, d, *J* = 8.7 Hz, 8-H), 6.60, (1H, s, Ar) 6.56 (1H, s, Ar), 6.13 (1H, dd, *J* = 15.4 and 6.4 Hz, 2-H), 5.31 (1H, dtd, *J* = 15.6, 7.2 and 1.6 Hz, 3-H), 5.12 (1H, d, *J* = 6.3 Hz, 1-H), 4.61 (1H, br s, NH), 4.31 (2H, s, CH_2 Bn), 3.80 (3H, s, OMe), 3.76 (3H, s, OMe), 2.87 (2H, m, 4-H_2). **^{13}C NMR (101 MHz, $CDCl_3$)** δ 158.2 (1C, C Ar), 145.7 (1C, C Ar), 140.4 (1C, C-2), 139.0 (1C, C Ar), 137.8 (1C, C Ar), 134.3 (1C, C Ar), 129.6 (2C, C-7), 128.8 (2C, CH Ar), 127.7 (2C, CH Ar), 127.49 (1C, CH Ar), 127.48 (1C, C Ar), 126.2 (1C, q, *J* = 276.6 Hz, CF_3), 126.0 (1C, C Ar), 119.8 (1C, q, *J* = 3.5 Hz, C-3), 113.9 (2C, C-8), 110.64 (1C, CH Ar), 110.56 (1C, CH Ar), 55.7(1C, OMe), 55.3 (1C, OMe), 48.7 (1C, C-1), 48. (1C, CH_2 Bn) 1, 37.4 (1C, q, *J* = 29.7 Hz, C-4). **^{19}F NMR (376 MHz, $CDCl_3$)** δ –66.3 (3F, CF_3). **HRMS** (ESI): calculated for $C_{26}H_{26}ClF_3NO_2$ $[M+H]^+$ requires *m/z* 476.1599, found 476.1598.

(*E*)-*N*-Benzyl-3-fluoro-2-methyl-4-[5,5,5-trifluoro-1-(4-methoxyphenyl)pent-2-en-1-yl]aniline, 5i.

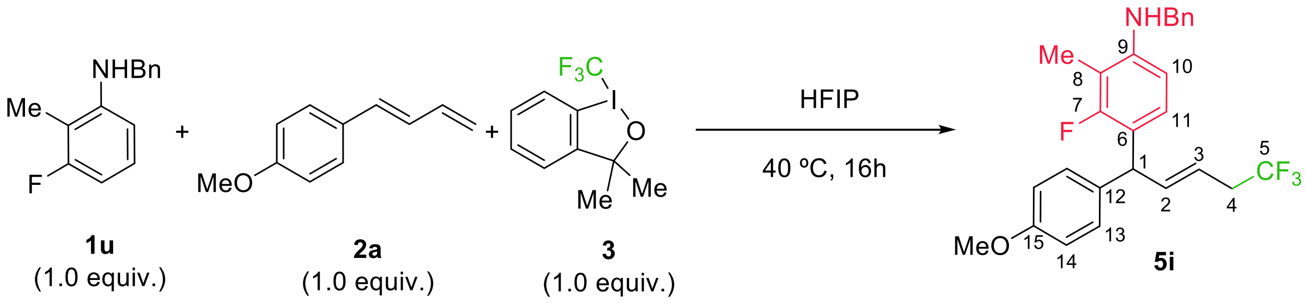

From aniline **1u** (43.1 mg, 0.2 mmol), alkene **2a** (32.0 mg, 0.2 mmol) and trifluoromethyl reagent **3** (66.0 mg, 0.2 mmol), in 0.5 mL of HFIP, following the general procedure, aniline **5i** was obtained. Chromatographic purification (gradient elution: 0:100 → 25:75 Et_2O – hexane) gave **5i** (39.8 mg, 45%), as a colourless oil.

Data for **5i**: R_f 0.25 (30% Et_2O – hexane). **^{1}H NMR (400 MHz, $CDCl_3$)** 7.41 – 7.27 (5H, m, Ar), 7.11 (2H, d, *J* = 8.6 Hz, 13-H), 6.85 (2H, *J* = 8.6 Hz, 14-H), 6.82 (1H, d, *J* = 8.4 Hz, 11-H), 6.39 (1H, d, *J* = 8.5 Hz, 10-H), 6.16 (1H, dd, *J* = 15.4 and 6.9 Hz, 2-H), 5.34 (1H, dt, *J* = 15.2 and 7.3 Hz, 3-H), 4.92 (1H, d, *J* = 6.9 Hz, 1-H), 4.37 (2H, s, CH_2 Bn), 3.80 (3H, s, OMe), 2.86 (2H, qd, *J* = 10.7 and 7.0 Hz, 4-H_2), 2.07 (3H, d, *J* = 1.8 Hz, Me). **^{13}C NMR (101 MHz, $CDCl_3$)** δ 159.0 (1C, d, *J* = 242.1 Hz, C-7), 158.2 (1C, C-15), 146.5 (1C, d, *J* = 7.0 Hz, C-9), 140.1 (1C, C-2), 139.3 (1C, C Ar), 134.7 (1C, C Ar), 129.4 (2C, C-13), 128.9 (2C, CH Ar), 127.7 (2C, CH Ar), 127.5 (1C, CH Ar), 127.1 (1C, d, *J* = 6.6 Hz, C-11), 126.1 (1C, q, *J* = 277.3 Hz, CF_3), 119.2 (1C, q, *J* = 3.7 Hz, C-3), 118.9 (1C, d, *J* = 17.0 Hz, C-6), 113.9 (2C, C-14), 109.0 (1C, d, *J* = 19.6 Hz, C-8), 105.6 (1C, d, *J* = 2.8 Hz, C-10), 55.3 (1C, OMe), 48.7 (1C, CH_2 Bn), 45.9 (1C, d, *J* = 2.9 Hz, C-1), 37.4 (1C, q, *J* = 29.5 Hz, C-4), 8.6 (1C, d, *J* = 6.9 Hz, Me). **^{19}F NMR (376 MHz, $CDCl_3$)** δ –66.3 (3F, CF_3), –121.4 (1F, Ar F). **HRMS** (ESI): calculated for $C_{26}H_{26}F_4NO$ $[M+H]^+$ requires *m/z* 444.1945, found 444.1946.

(*E*)-*N*-Benzyl-4-[5,5,5-trifluoro-1-(4-methoxyphenyl)-4-methylpent-2-en-1-yl]aniline, 5j.

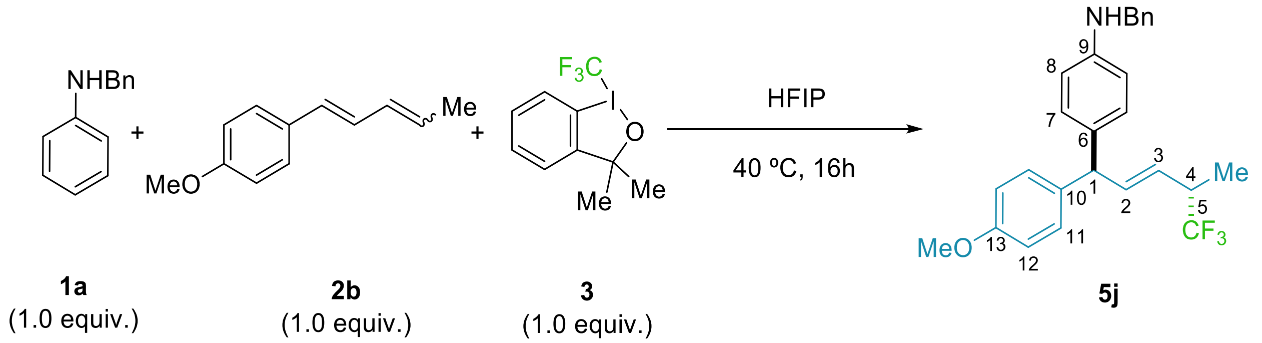

From aniline **1a** (36.7 mg, 0.20 mmol), alkene **2b** (34.8 mg, 0.20 mmol) and trifluoromethyl reagent **3** (66.0 mg, 0.20 mmol), in 0.5 mL of HFIP, following the general procedure, aniline **5j** was obtained as an inseparable *anti:syn* 60:40 mixture. Chromatographic purification (gradient elution: 0:100 → 5:95 Et_2O – hexane) gave **5j** (63.6 mg, 75%, dr 60:40), as a colourless oil.

Data for **5j** (major isomer, from the mixture): ***R**$_f$* 0.45 (30% Et_2O – hexane). **^{1}H NMR (400 MHz, $CDCl_3$)** δ 7.41 – 7.26 (5H, m, Ar), 7.07 (2H, d, *J* = 8.6 Hz, 11-H), 6.96 (2H, d, *J* = 8.4 Hz, 7-H), 6.84 (2H, d, *J* = 8.8 Hz, 12-H), 6.64 (2H, d, *J* = 8.5 Hz, 8-H), 6.08 (1H, dd, *J* = 15.4 and 7.4 Hz, 2-H), 5.33 (1H, dd, *J* = 15.4 and 7.9 Hz, 3-H), 4.59 (1H, d, *J* = 7.3 Hz, 1-H), 4.31 (2H, s, CH_2 Bn), 3.79 (3H, s, OMe), 2.91 (1H, m, 4-H), 1.22 (3H, d, *J* = 7.0 Hz, Me).**^{13}C NMR (101 MHz, $CDCl_3$)** δ 158.2 (1C, C-13), 145.8 (1C, C Ar), 138.9 (1C, C-2), 138.4 (1C, C Ar), 135.9 (1C, C Ar), 133.4 (1C, C Ar) 129.5 (2C, CH Ar), 129.4 (2C, CH Ar), 128.8 (2C, CH Ar), 128.0 (2C, CH Ar), 127.5 (1C, CH Ar), 127.4 (1C, q, *J* = 278.8 Hz, CF_3), 125.9 (1C, C-3), 113.9 (2C, CH Ar), 113.8 (2C, CH Ar), 55.4 (1C, OMe), 52.2 (1C, C-1), 49.2 (1C, CH_2 Bn), 41.6 (1C, q, *J* = 27.4 Hz, C-4), 13.6 (1C, Me).**^{19}F NMR (376 MHz, $CDCl_3$)** δ –72.7 (3F, CF_3). **HRMS** (ESI): calculated for $C_{26}H_{26}F_3NO$ $[M+H]^+$ requires m/z 426.2039, found m/z 426.2040.

Partial data for **5j** (minor isomer, from the mixture): The NMR signals overlapped with major isomer, except for: **^{1}H NMR (400 MHz, $CDCl_3$)** δ 7.06 (2H, d, *J* = 8.5 Hz, 11-H), 6.97 (2H, d, *J* = 8.4 Hz, 7-H), 6.83 (2H, d, *J* = 8.8 Hz, 12-H), 6.64 (2H, d, *J* = 8.4 Hz, 6-H).

(*E*)-7-[4-(benzylamino)phenyl]-7-(4-methoxyphenyl)-4-(trifluoromethyl)hept-5-enoic acid, 5k.

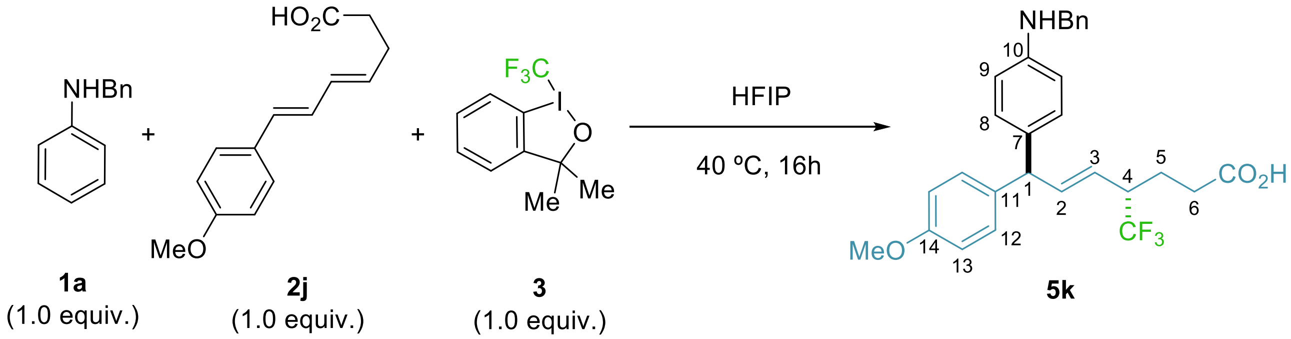

From aniline **1a** (36.6 mg, 0.2 mmol), alkene **2j** (46.5 mg, 0.2 mmol) and trifluoromethyl reagent **3** (66.0 mg, 0.2 mmol), in 0.5 mL of HFIP, following the general procedure, aniline **5k** was obtained as an inseparable *anti:syn* 60:40 mixture. Chromatographic purification (gradient elution: 20:80 → 50:50 Et_2O – hexane– 2% AcOH) gave **5k** (22.5 mg, 40%, dr 60:40), as a colourless oil.

Data for **5k** (major isomer, from the mixture): ***R**$_f$* 0.10 (28% Et_2O – hexane – 2% AcOH). **^{1}H NMR (400 MHz, $CDCl_3$)** δ 7.40 – 7.26 (5H, m, Ar), 7.07 (2H, d, *J* = 8.7 Hz, 12-H), 6.93 (2H, d, *J* = 8.5 Hz, 8-H), 6.84 (2H, d, *J* = 8.6 Hz, 13-H), 6.59 (2H, d, *J* = 8.5 Hz, 9-H), 6.14 (1H, dd, *J* = 15.4

and 7.0 Hz, 2-H), 5.18 – 5.10 (1H, m, 3-H), 4.62 (1H, d, J = 6.9 Hz, 1-H), 4.30 (2H, s, CH_2 Bn), 3.79 (3H, s, OMe), 2.95 – 2.80 (1H, m, 4-H), 2.47 – 2.28 (2H, m, 6-H_2), 2.16 – 2.02 (1H, m, 5-H_a), 1.80 – 1.67 (1H, m, 5-H_b). **^{13}C NMR (101 MHz, $CDCl_3$)** δ 177.9 (1C, C=O), 158.2 (1C, C Ar), 146.8 (1C, C Ar), 141.7 (1C, C-2), 139.6 (1C, C Ar), 135.6 (1C, C Ar), 132.1 (1C, C Ar), 129.5 (2C, C-12), 129.4 (2C, C-8), 128.8 (2C, CH Ar), 127.7 (2C, CH Ar), 127.4 (1C, CH Ar), 123.4 (1C, C-3), 114.0 (2C, C-13), 113.1 (2C, C-9), 55.4 (1C, OMe), 52.2 (1C, C-1), 48.7 (1C, CH_2 Bn), 46.8 (1C, q, J = 26.5 Hz, C-4), 30.7 (1C, C-6), 22.9 (1C, C-5). **^{19}F NMR (376 MHz, $CDCl_3$)** δ –71.0 (3F, CF_3). **HRMS** (ESI): calculated for $C_{28}H_{29}F_3NO_3$ $[M+H]^+$ requires *m/z* 484.2094, found *m/z* 484.2086.

Partial data for **5k** (minor isomer, from the mixture): The NMR signals overlapped with major isomer, except for: **^{1}H NMR (400 MHz, $CDCl_3$)** δ 7.05 (2H, d, J = 8.6 Hz, 12-H), 6.96 (2H, d, J = 8.6 Hz, 8-H), 6.83 (2H, d, J = 8.8 Hz, 13-H), 6.60 (2H, d, J = 8.6 Hz, 9-H), 4.31 (2H, s, CH_2 Bn), 3.78 (3H, s, OMe). **^{13}C NMR (101 MHz, $CDCl_3$)** δ 158.2 (1C, C Ar), 146. (1C, C Ar) 135.57 (1C, C Ar), 132.6 (1C, C Ar), 129.52 (2C, C-12), 129.4 (2C, C-8), 113.9 (2C, C-13), 113.1 (2C, C-9).

(*E*)-*N*-Benzyl-4-[5,5,5-trifluoro-1-(4-methoxyphenyl)-4,4-dimethylpent-2-en-1-yl]aniline, 5l.

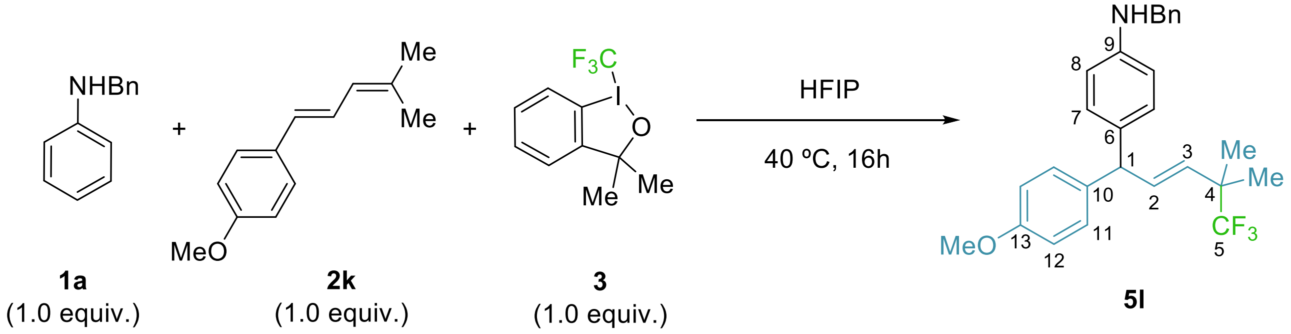

From aniline **1a** (36.7 mg, 0.20 mmol), alkene **2k** (37.7 mg, 0.20 mmol) and trifluoromethyl reagent **3** (66.0 mg, 0.20 mmol), in 0.5 mL of HFIP, following the general procedure, aniline **5l** was obtained. Chromatographic purification (gradient elution: 0:100 → 5:95 Et_2O – hexane) gave **5l** (44.5 mg, 52%), as a colourless oil.

Data for **5l**: ***R_f*** 0.40 (30% Et_2O – hexane). **^{1}H NMR (400 MHz, $CDCl_3$)** δ 7.43 – 7.28 (5H, m, Ar), 7.07 (2H, d, J = 8.7 Hz, 11-H), 6.96 (2H, d, J = 8.5 Hz, 7-H), 6.85 (2H, d, J = 8.7 Hz, 12-H), 6.62 (2H, d, J = 8.5 Hz, 8-H), 6.06 (1H, dd, J = 15.7 and 7.5 Hz, 2-H), 5.49 (1H, d, J = 15.8 Hz, 3-H), 4.60 (1H, d, J = 7.5 Hz, 1-H), 4.32 (2H, s, CH_2 Bn), 3.80 (3H, s, OMe), 1.25 (6H, s, 2 x Me). **^{13}C NMR (101 MHz, $CDCl_3$)** δ 158.1 (1C, C-13), 146.4 (1C, C Ar), 139.4 (1C, C Ar), 136.1 (1C, C Ar), 135.2 (1C, C-2), 133.0 (1C, C Ar), 131.1 (1C, q, J = 1.9 Hz, C-3), 129.5 (2C, CH Ar), 129.4 (2C, CH Ar), 128.8 (2C, CH Ar), 128.6 (1C, q, J = 282.0 Hz, CF_3), 127.8 (2C, CH Ar), 127.4 (1C, CH Ar), 113.9 (2C, CH Ar), 113.3 (2C, CH Ar), 55.4 (1C, OMe), 52.2 (1C, C-1), 48.9 (1C, CH_2 Bn), 42.4

(1C, q, $J = 25.3$ Hz, C-4), 21.4 (2C, 2 x Me). **^{19}F NMR (376 MHz, $CDCl_3$)** δ –77.9 (3F, CF_3). **HRMS** (ESI): calculated for $C_{27}H_{28}F_3NO$ $[M+H]^+$ requires *m/z* 440.2196, found *m/z* 440.2185.

(*E*)-*N*-Benzyl-4-{1-(4-methoxyphenyl)-3-[1-(trifluoromethyl)cyclobutyl]allyl}aniline, 5m.

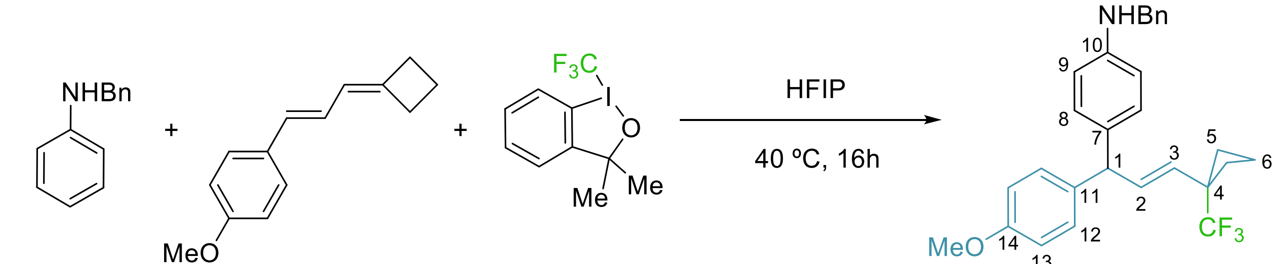

1a (1.0 equiv.) **2l** (1.0 equiv.) **3** (1.0 equiv.) **5m**

From aniline **1a** (36.6 mg, 0.2 mmol), alkene **2l** (40.1 mg, 0.2 mmol) and trifluoromethyl reagent **3** (66.0 mg, 0.2 mmol), in 0.5 mL of HFIP, following the general procedure, aniline **5m** was obtained. Chromatographic purification (gradient elution: 0:100 → 25:75 Et_2O – hexane) gave **5m** (33%), as a colourless oil.

Data for **5m**: ***R$_f$*** 0.25 (30% Et_2O – hexane). **^{1}H NMR (400 MHz, $CDCl_3$)** δ 7.41 – 7.25 (5H, m, Ar), 7.08 (2H, d, $J = 8.6$ Hz, 12-H), 6.96 (2H, d, $J = 8.5$ Hz, 8-H), 6.84 (2H, d, $J = 8.7$ Hz, 13-H), 6.60 (2H, d, $J = 8.6$ Hz, 9-H), 6.14 (1H, dd, $J = 15.6$ and 7.3 Hz, 2-H), 5.49 (1H, dd, $J = 15.6$ and 1.4 Hz, 3-H), 4.64 (1H, d, $J = 7.3$ Hz, 1-H), 4.31 (2H, s, CH_2 Bn), 3.79 (3H, s, OMe), 2.46 – 2.34 (2H, m, 5-H$_a$), 2.15 – 2.04 (2H, m, 5-H$_b$), 2.03 – 1.85 (2H, m, 6-H$_2$). **^{13}C NMR (101 MHz, $CDCl_3$)** δ 158.1 (1C, C-14), 146.7 (1C, C Ar), 139.6 (1C, C Ar), 136.3 (1C, C Ar), 136.1 (1C, C-2), 132.7 (1C, C Ar), 129.6 (2C, CH Ar), 129.4 (2C, CH Ar), 128.9 (1C, q, $J = 2.6$ Hz, C-3), 128.8 (2C, CH Ar), 127.9 (1C, q, $J = 277.0$ Hz, CF_3), 127.7 (2C, CH Ar), 127.4 (1C, CH Ar), 113.9 (2C, CH Ar), 113.1 (2C, CH Ar), 55.4 (1C, OMe), 52.1 (1C, C-1), , 48.7 (1C, CH_2 Bn), 47.1 (1C, q, $J = 27.4$ Hz, C-4), 27.0 – 26.8 (2C, m, C-5), 15.3 (1C, C-6). **^{19}F NMR (376 MHz, $CDCl_3$)** δ –77.8 (3F, CF_3). **HRMS** (ESI): calculated for $C_{28}H_{29}F_3NO$ $[M+H]^+$ requires *m/z* 452.2196, found *m/z* 452.2189.

(*E*)-*N*-Benzyl-4-[5,5,5-trifluoro-1-(2-methoxyphenyl)pent-2-en-1-yl]aniline, 5n.

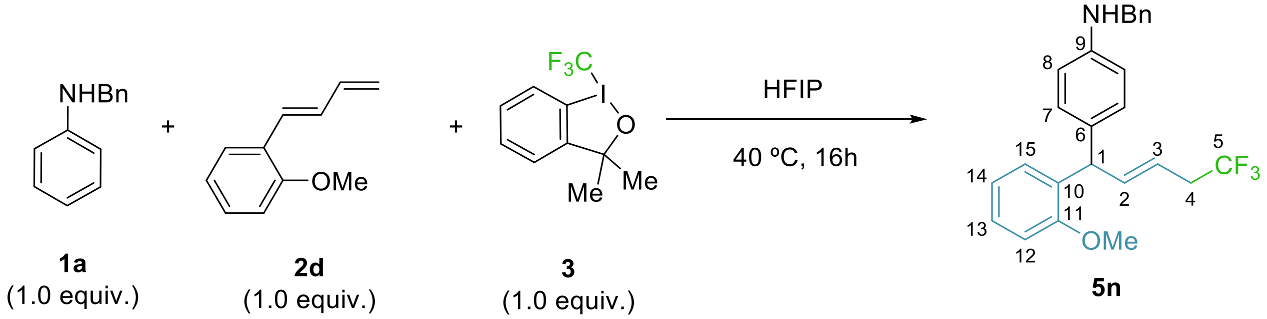

1a (1.0 equiv.) **2d** (1.0 equiv.) **3** (1.0 equiv.) **5n**

From aniline **1a** (36.7 mg, 0.2 mmol), alkene **2d** (32.1 mg, 0.2 mmol) and trifluoromethyl reagent **3** (66.0 mg, 0.2 mmol), in 0.5 mL of HFIP, following the general procedure, aniline **5n** was obtained. Chromatographic purification (gradient elution: 0:100 → 15:85 Et_2O – hexane) gave **5n** (29.0 mg, 35%), as a colourless oil.

Data for **5n**: ***R****f* 0.15 (15% Et_2O – hexane). **^{1}H NMR (400 MHz, CDCl$_3$)** δ 7.40 – 7.26 (5H, m, Ar), 7.20 (1H, ddd, *J* = 8.1, 7.4 and 1.8 Hz, 13-H), 7.06 (1H, dd, *J* = 7.6 and 1.8 Hz, 15-H), 6.97 (2H, d, *J* = 8.4 Hz, 7-H), 6.90 (1H, td, *J* = 7.5 and 1.2 Hz, 14-H), 6.86 (1H, dd, *J* = 8.2 and 1.2 Hz, 12-H), 6.59 (2H, d, *J* = 8.5 Hz, 8-H), 6.13 (1H, dd, *J* = 15.4 and 6.8 Hz, 2-H), 5.33 – 5.21 (1H, m, 3-H), 5.07 (1H, d, *J* = 6.8 Hz, 1-H), 4.30 (2H, s, CH_2 Bn), 3.77 (·H, s, OMe), 2.82 (2H, qd, *J* = 10.7 and 7.0 Hz, 4-H_2). **^{13}C NMR (101 MHz, CDCl$_3$)** δ 157.0 (1C, C-11), 146.5 (1C, C Ar), 140.7 (1C, C-2), 139.5 (1C, C Ar), 132.05 (1C, C Ar), 131.99 (1C, C Ar) 129.5 (2C, C-7), 129.3 (1C, C-15), 128.8 (2C, CH Ar), 127.8 (2C, CH Ar), 127.6 (1C, C-13), 127.4 (1C, CH Ar), 126.2 (1C, q, *J* = 276.5 Hz, CF_3), 120.6 (1C, C-14), 118.7 (1C, q, *J* = 3.5 Hz, C-3), 113.1 (2C, C-8), 110.9 (1C, C-12), 55.7 (1C, OMe), 48.9 (1C, CH_2 Bn), 45.8 (1C, C-1), 37.5 (1C, q, *J* = 29.5 Hz, C-4). **^{19}F NMR (376 MHz, CDCl$_3$)** δ –66.4 (3F, CF_3). **HRMS** (ESI): calculated for $C_{25}H_{25}F_3NO$ $[M+H]^+$ requires *m/z* 412.1883, found *m/z* 412.1878.

(*E*)-*N*-Benzyl-4-[1-(2,3-dihydrobenzofuran-5-yl)-5,5,5-trifluoropent-2-en-1-yl]aniline, 5o.

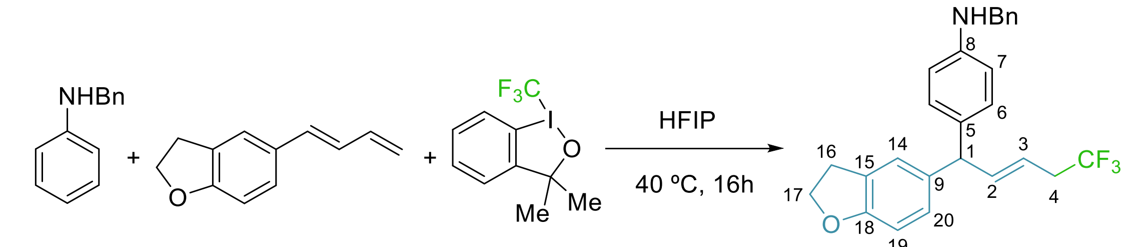

1a (1.0 equiv.) **2m** (1.0 equiv.) **3** (1.0 equiv.) **5o**

From aniline **1a** (36.7 mg, 0.2 mmol) and diene **2m** (46.9 mg, 0.2 mmol), in 0.5 mL of HFIP, following the general procedure, aniline **5o** was obtained. Chromatographic purification (0:100 → 20:80 Et_2O – hexane) gave aniline **5o** (31.7 mg, 37%) as a colourless oil.

Data for **5o**: **R***f* 0.20 (20% Et_2O – hexane). **^{1}H NMR (500 MHz, CDCl$_3$)** δ 7.40 – 7.31 (4H, m, Ar), 7.30 – 7.26 (1H, m, Ar), 7.01 – 6.92 (3H, m, 6-H and 14-H), 6.90 (1H, dd, *J* = 8.3 and 1.9 Hz, 20-H), 6.71 (1H, d, *J* = 8.2 Hz, 19-H), 6.60 (2H, d, *J* = 8.6 Hz, 7-H), 6.12 (1H, dd, *J* = 15.3 and 7.3 Hz, 2-H), 5.34 (1H, dtd, *J* = 15.5, 7.1 and 1.4 Hz, 3-H), 4.58 (1H, d, *J* = 7.4 Hz, 1-H), 4.54 (2H, t, *J* = 8.7 Hz, 17-H_2), 4.31 (1H, s, CH_2 Bn), 4.01 (1H, s, NH), 3.16 (2H, t, *J* = 8.7 Hz, 16-H), 2.84 (1H, qd, *J* = 10.8 and 7.1 Hz, 4-H_2).**^{13}C NMR (125 MHz, CDCl$_3$)** δ 168.1 (1C, C Ar), 158.7 (1C, C Ar), 146.8 (1C, C Ar), 141.4 (1C, C-2), 139.6 (1C, C Ar), 135.8 (1C, C Ar), 132.5 (1C, C Ar), 129.4 (2C,

C-6), 128.8 (2C, CH Ar), 128.1 (1C, C-20), 127.7 (2C, CH Ar), 127.4 (1C, CH Ar), 125.1 (1C, C-14), 118.77 (1C, q, J = 3.9 Hz, C-3), 113.0 (2C, C-7), 109.1 (1C, C-19), 71.4 (1C, C-17), 52.5 (1C, C-1), 48.7 (1C, CH_2 Bn), 37.4 (1C, q, J = 29.7 Hz, C-4), 29.9 (1C, C-16). **^{19}F NMR (376 MHz, $CDCl_3$)** δ –71.0 (3F, CF_3)

(*E*)-*N*-Benzyl-4-[1-(2,3-dihydrobenzofuran-5-yl)-5,5,5-trifluoropent-2-en-1-yl]aniline, 5p.

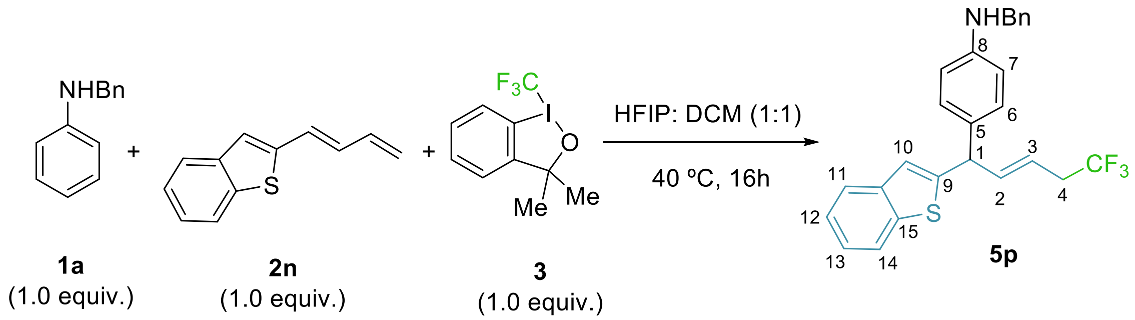

From aniline **1a** (36.7 mg, 0.2 mmol) and diene **2n** (37.3 mg, 0.2 mmol), in 0.5 mL of HFIP: DCM (1:1) at 40 ºC, following the general procedure, aniline **5p** was obtained. Chromatographic purification (0:100 → 12:88 Et_2O – hexane) gave aniline **5p** (29.2 mg, 33%, rr 4:1) as a colourless oil.

Data for **5p**: **R_f** 0.20 (20% Et_2O – hexane). **^{1}H NMR (400 MHz, $CDCl_3$)** δ 7.75 (1H, d, J = 7.8 Hz, Ar), 7.68 (1H, d, J = 7.7 Hz, Ar), 7.49 – 7.31 (7H, m, Ar), 7.10 (2H, d, J = 8.5 Hz, 6-H), 7.00 (1H, s, 10-H), 6.65 (2H, d, J = 8.5 Hz, 7-H), 6.22 (1H, dd, J = 15.3 and 7.5 Hz, 2-H), 5.56 (1H, ddd, J = 14.8, 7.7 and 6.5 Hz, 3-H), 4.90 (1H, d, J = 7.6 Hz, 1-H), 4.35 (2H, s, CH_2 Bn), 3.02 – 2.74 (2H, m, 4-H_2). **^{13}C NMR (101 MHz, $CDCl_3$)** δ 148.9 (1C, C Ar), 147.5 (1C, C Ar), 139.99 (1C, C Ar), 139.98 (1C, C Ar) 139.6 (1C, C-2), 139.4 (1C, C Ar), 130.9 (1C, C Ar), 129.2 (2C, CH Ar), 128.8 (2C, CH Ar), 127.7 (2C, CH Ar), 127.5 (1C, CH Ar), 124.3 (1C, CH Ar), 123.9 (1C, CH Ar), 123.3 (1C, CH Ar), 122.3 (1C, CH Ar), 121.6 (1C, C-10), 119.8 (1C, q, J = 3.5 Hz, C-3), 113.0 (2C, CH Ar), 49.4 (1C, C-1), 48.6 (1C, CH_2 Bn), 37.4 (1C, q, J = 29.6 Hz, C-4).

(*E*)-*N*-Benzyl-4-(5,5,5-trifluoro-3-methyl-1-(thiophen-2-yl)pent-2-en-1-yl)aniline, 5q.

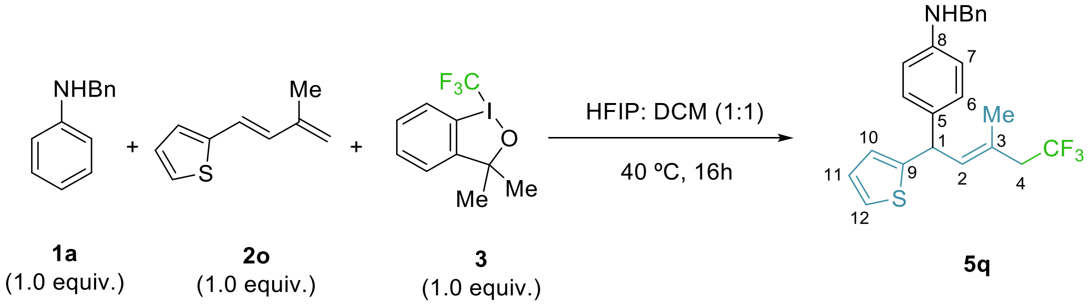

From aniline **1a** (36.7 mg, 0.2 mmol), diene **2o** (30.0 mg, 0.2 mmol) and trifluoromethyl reagent **3** (66.0 mg, 0.2 mmol), in 0.5 mL of HFIP: DCM (1:1) at 40 ºC, following the general

procedure, aniline **5q** was obtained. Chromatographic purification (0:100 → 10:90 Et_2O – hexane) gave aniline **5q** (34.3 mg, 43%, *E*:*Z* 8:1) as a yellow oil.

Data for **5q**: **R*f*** 0.40 (20% Et_2O – hexane). **^{1}H NMR (400 MHz, $CDCl_3$)** δ 7.41 – 7.27 (5H, m, Ar), 7.17 (1H, dd, *J* = 5.1 and 1.2 Hz, 12-H), 7.07 (2H, d, *J* = 8.5 Hz, 6-H), 6.93 (1H, dd, *J* = 5.1 and 3.5 Hz, 11-H), 6.77 (1H, d, *J* = 3.5 Hz, 10-H), 6.61 (2H, d, *J* = 8.6 Hz, 7-H), 5.88 (1H, d, *J* = 9.5 Hz, 2-H), 5.02 (1H, d, *J* = 9.5 Hz, 1-H), 4.32 (2H, s, CH_2 Bn), 4.03 (2H, s, NH), 2.82 (2H, q, *J* = 10.9 Hz, 4-H_2), 1.86 (3H, s, Me).**^{13}C NMR (101 MHz, $CDCl_3$)** δ 149.1 (1C, C Ar), 147.2 (1C, C Ar), 139.5 (1C, C Ar), 135.6 (1C, C-2), 132.7 (1C, C Ar), 128.80 (2C, CH Ar), 128.78 (2C, CH Ar), 127.7 (2C, CH Ar), 127.4 (1C, CH Ar), 126.8 (1C, C-11), 126.4 (1C, q, *J* = 278.0 Hz, CF_3), 125.5 (1C, q, *J* = 2.7 Hz, C-3), 124.5 (1C, C-10), 124.1 (1C, C-12), 113.1 (2C, CH Ar), 48.6 (1C, CH_2 Bn), 44.4 (1C, C-1), 43.62 (1C, q, *J* = 28.6 Hz, C-4), 17.0 (1C, Me).

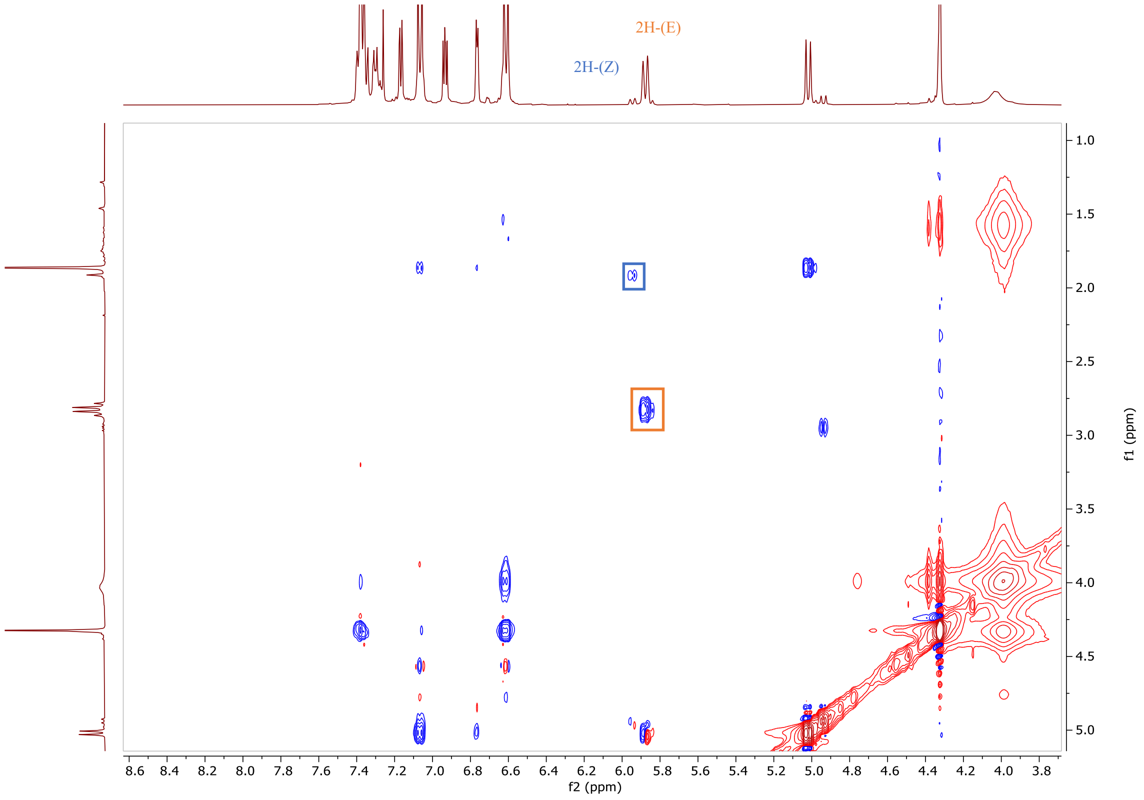

(*E*)-4-[5,5,5-trifluoro-1-(4-methoxyphenyl)pent-2-en-1-yl]aniline, 5r.

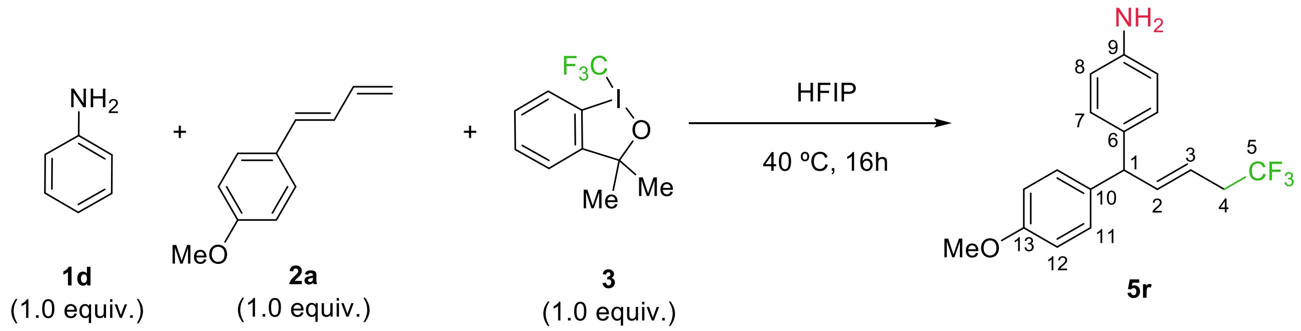

From aniline **1d** (46.6 mg, 0.5 mmol), alkene **2a** (80.1 mg, 0.5 mmol) and trifluoromethyl reagent **3** (165.0 mg, 0.5 mmol), in 1.25 mL of HFIP, following the general procedure (at 25ºC), aniline **5r** was obtained. Chromatographic purification (gradient elution: 0:100 → 50:50 EtOAc – hexane) gave **5r** (30.0 mg, 19%), as an orange oil.

Data for **5r**: ***Rf*** 0.30 (40% EtOAc – hexane). **^{1}H NMR (400 MHz, CDCl$_3$)** δ 7.25 (2H, d, *J* = 8.6 Hz, 11-H), 7.09 (2H, d, *J* = 8.4 Hz, 12-H), 7.04 (2H, d, *J* = 8.7 Hz, 7-H), 6.83 (2H, d, *J* = 8.7 Hz, 8-H), 6.11 (1H, dd, *J* = 15.3 and 7.3 Hz, 2-H), 5.34 (1H, dtd, *J* = 15.6, 7.2 and 1.4 Hz, 3-H), 4.66 (1H, d, *J* = 7.3 Hz, 1-H), 3.78 (3H, s, OMe), 2.84 (2H, qd, *J* = 10.7 and 6.9 Hz, 4-H$_2$). **^{13}C NMR (101 MHz, CDCl$_3$)** δ 158.4 (1C, C-13), 140.5 (1C, C-2), 139.4 (1C, C Ar), 136.4 (1C, C Ar), 134.9 (1C, C Ar), 129.5 (2C, CH Ar), 129.4 (2C, CH Ar), 126.1 (1C, q, *J* = 276.3 Hz, CF$_3$), 121.5 (2C, C-11), 119.7 (1C, q, *J* = 3.7 Hz, C-3), 114.1 (2C, C-8), 55.4 (1C, OMe), 52.5 (1C, C-1), 37.4 (1C, q, *J* = 29.6 Hz, C-4). **^{19}F NMR (376 MHz, CDCl$_3$)** δ –66.4 (3F, CF$_3$).

Unreactive or low yielding dienes towards both the hydroarylation and the trifluoromethyl.

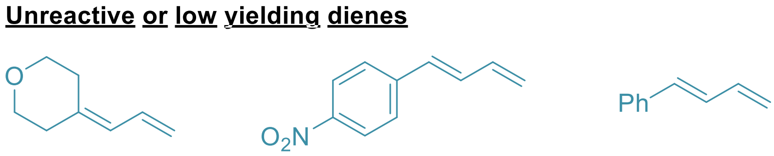

6. Synthesis of sulfonamides and carbamates.

6.1. General procedure for the synthesis of sulfonamides or carbamates

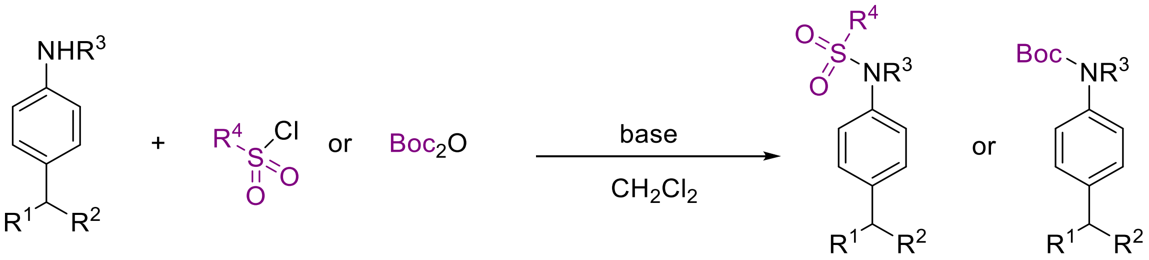

To an oven-dried vial/flask a solution of aniline in dry CH$_2$Cl$_2$ (0.1 – 0.3 M), or the stated solvent, was added, followed by the corresponding base (0 – 5 equiv.) and sulfonyl chloride (1.2 – 1.5 equiv.) or Boc$_2$O (1.2 – 2.4 equiv., 0.4 M solution of dry CH$_2$Cl$_2$) at 0 ºC. The reaction mixture was let to warm up to rt and stirred overnight. The reaction mixture was diluted with CH$_2$Cl$_2$, extracted with water and the organic layers dried over Na$_2$SO$_4$. The resulting crude was purified by column chromatography using the appropriate mixture of eluents.

***N*-Benzyl-2,4-dinitro-*N*-{4-[(*E*)-5,5,5-trifluoro-1-(4-methoxyphenyl)-4-methylpent-2-en-1-yl]phenyl}benzenesulfonamide, 6.**

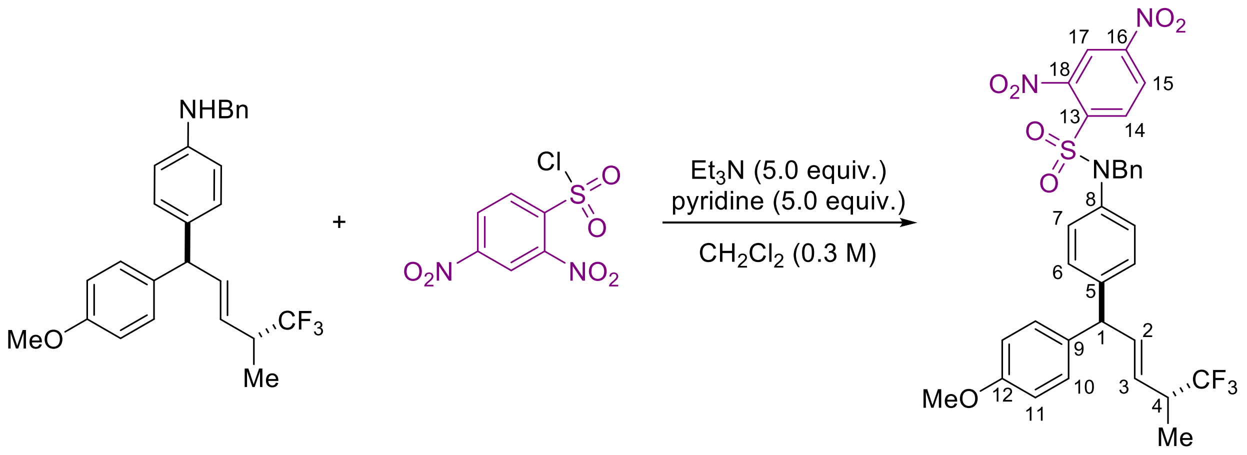

5j (dr 60:40) **6** (dr 70: 30)

From aniline **5j** (134.5 mg, 0.316 mmol), triethylamine (220 µL, 1.580 mmol, 5.0 equiv.), pyridine (128 µL, 1.580 mmol, 5.0 equiv.) and 2,4-dinitrosulfonyl chloride (126.4 mg, 0.474 mmol, 1.5 equiv.), in 1.0 mL of dry CH_2Cl_2, following the general procedure, sulfonamide **6** was obtained. Chromatographic purification (gradient elution: 0:100 → 40:70 Et_2O – hexane) gave **6** (96.9 mg, 47%), as a yellow foam, that was recrystalized using THF – pentane, to give white crystal needles that were analysed using X-Ray diffraction.

Data for **6** (major diastereoisomer, from the mixture): ***R*$_f$** 0.20 (40% Et_2O – hexane). **m.p.** (THF – pentane): 89 ºC. **^{1}H NMR (400 MHz, $CDCl_3$)** δ 8.48 (1H, d, *J* = 2.2 Hz, 17-H), 8.32 – 8.19 (1H, m, 15-H), 7.72 (1H, d, *J* = 8.7 Hz, 14-H), 7.30 – 7.18 (5H, m, Ar), 7.04 – 6.92 (6H, m, Ar), 6.86 – 6.79 (2H, m, Ar), 6.17 – 5.89 (1H, m, 2-H), 5.21 (1H, ddd, *J* = 15.4, 7.9 and 1.4 Hz, 3-H), 4.94 (2H, s, CH_2 Bn), 4.71 – 4.57 (1H, m, 1-H), 3.80 (3H, s, OMe), 3.04 – 2.76 (1H, m, 4-H), 1.20 (3H, d, *J* = 7.1 Hz, Me). **^{13}C NMR (101 MHz, $CDCl_3$)** δ 158.6 (1C C Ar), 149.8 (1C, C Ar), 148.2 (1C, C Ar), 144.5 (1C, C Ar), 137.9 (1C, C Ar), 137.3 (1C, C-2), 135.6 (1C, C Ar), 135.3 (1C, C Ar), 134.2 (1C, C Ar), 133.9 (1C, CH Ar), 129.72 (2C, CH Ar), 129.70 (2C, CH Ar), 129.5 (2C, CH Ar), 128.9 (2C, CH Ar), 128.7 (2C, CH Ar), 128.2 (1C, CH Ar), 127.3 – 127.0 (1C, m, C-3), 125.5 (1C, CH Ar), 119.6 (1C CH Ar), 114.2 (2C, CH Ar), 57.1 (1C, CH_2 Bn), 55.4 (1C, OMe), 52.4 (1C, C-1), 42.2 – 40.8 (1C, m, C-4), 13.8 – 13.4 (1C, m, Me). **^{19}F NMR (376 MHz, $CDCl_3$)** δ –72.72 (3F, CF_3).

Data for **6** (minor diastereoisomer, from the mixture): The NMR signals overlapped with major isomer, except for: **^{1}H NMR (400 MHz, $CDCl_3$)** δ 7.73 (1H, d, *J* = 8.7 Hz, 14-H), 1.21 (d, *J* = 7.1 Hz, 1H). **^{13}C NMR (101 MHz, $CDCl_3$)** δ 137.2 (1C, C-2), 135.4 (1C, C Ar), 134.1 (1C, C Ar), 129.8 (2C, CH Ar), 129.74 (2C, CH Ar), 129.6 (2C, CH Ar), 119.6 (1C, CH Ar), 114.1 (2C, CH Ar), 52.3 (1C, C-1). **^{19}F NMR (376 MHz, $CDCl_3$)** δ –72.71 (3F, CF_3).

(*E*)-*N*-{4-[4-(4-Methoxyphenyl)but-3-en-2-yl]phenyl}-*N*,4-dimethylbenzenesulfonamide, 4w.

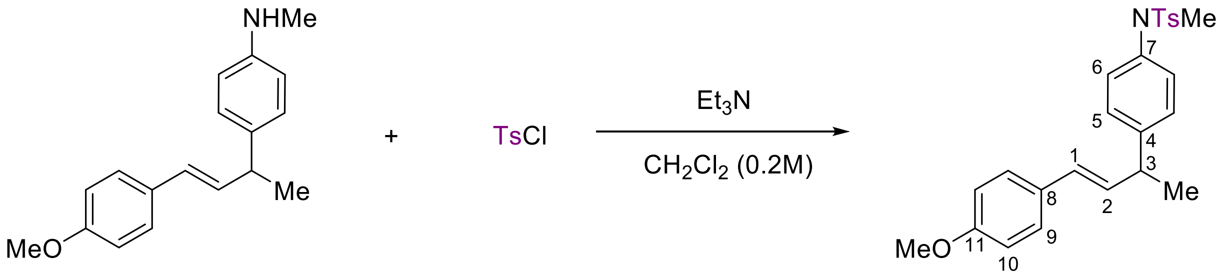

4b **4w**

From aniline **4b** (500.0 mg, 1.87 mmol), triethylamine (391 µL, 2.80 mmol, 1.5 equiv.) and *p*ara-toluenesulfonyl chloride (427.8 mg, 2.24 mmol, 1.2 equiv.) in 9.5 mL of dry CH_2Cl_2, following the general procedure, sulfonamide **4w** was obtained. Chromatographic purification (gradient elution: 0:100 → 30:70 Et_2O – hexane) gave **4w** (540 mg, 68%), as a colourless oil.

Data for **4w**: ***R**f* 0.20 (30% Et_2O – hexane). **^{1}H NMR (300 MHz, $CDCl_3$)** δ 7.48 (2H, d, *J* = 8.2 Hz, CH Ar), 7.35 – 7.17 (5H, m, Ar), 7.18 – 6.97 (3H, m, CH Ar), 6.86 (2H, d, *J* = 8.7 Hz, CH Ar), 6.38 (1H, d, *J* = 16.0 Hz, 1-H), 6.22 (1H, dd, *J* = 15.9 and 6.7 Hz, 2-H), 3.80 (3H, s, OMe), 3.63 (1H, quint, *J* = 7.0 Hz, 3-H), 3.16 (3H, s, NMe), 2.43 (3H, s, Me Ts), 1.46 (3H, d, *J* = 7.0 Hz, Me).

(*E*)-*N*-{3-Hydroxy-4-[4-(4-methoxyphenyl)but-3-en-2-yl]phenyl}-4-methylbenzenesulfonamide, 4x.

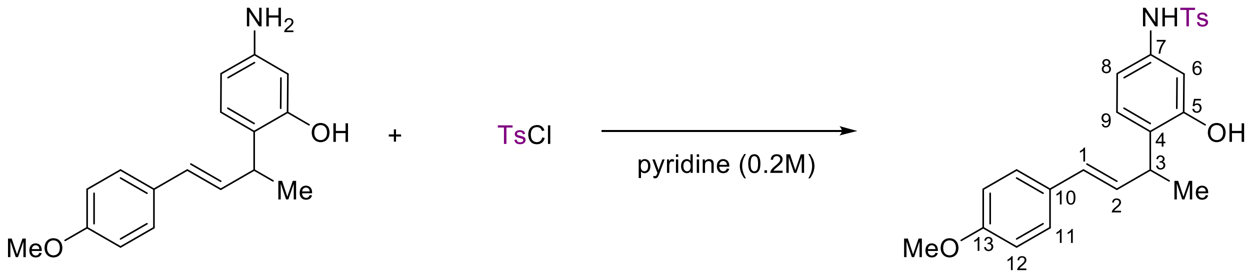

4n **4x**

From aniline **4n** (41.5 mg, 0.154 mmol) and *p*ara-toluenesulfonyl chloride (35.2 mg, 0.185 mmol, 1.2 equiv.) in 0.770 mL of pyridine, following the general procedure, sulfonamide **4x** was obtained. Chromatographic purification (gradient elution: 0:100 → 35:65 EtOAc – hexane) gave **4x** (62.5 mg, 96%), as a colourless oil.

Data for **4x**: ***R**f* 0.45 (50% EtOAc – hexane). **^{1}H NMR (300 MHz, $CDCl_3$)** δ 7.69 (2H, d, *J* = 8.2 Hz, Ts), 7.23 (2H, d, *J* = 8.6 Hz, 11-H), 7.17 (2H, d, *J* = 8.0 Hz, Ts), 6.99 (1H, d, *J* = 8.2 Hz, 9-H), 6.81 (2H, d, *J* = 8.7 Hz, 12-H), 6.74 (1H, d, *J* = 2.4 Hz, 6-H), 6.61 – 6.52 (1H, m, 8-H), 6.34 (1H, d, *J* = 16.1 Hz, 1-H), 6.18 (1H, dd, *J* = 16.0 and 6.3 Hz, 2-H), 3.89 – 3.70 (4H, m, OMe and 3-H), 2.32 (3H, s, Me Ts), 1.36 (3H, d, *J* = 7.0 Hz, Me).

(*E*)-*N*-Benzyl-4-methyl-N-{4-[5,5,5-trifluoro-1-(4-methoxyphenyl)pent-2-en-1-yl]phenyl}benzenesulfonamide, 5s.

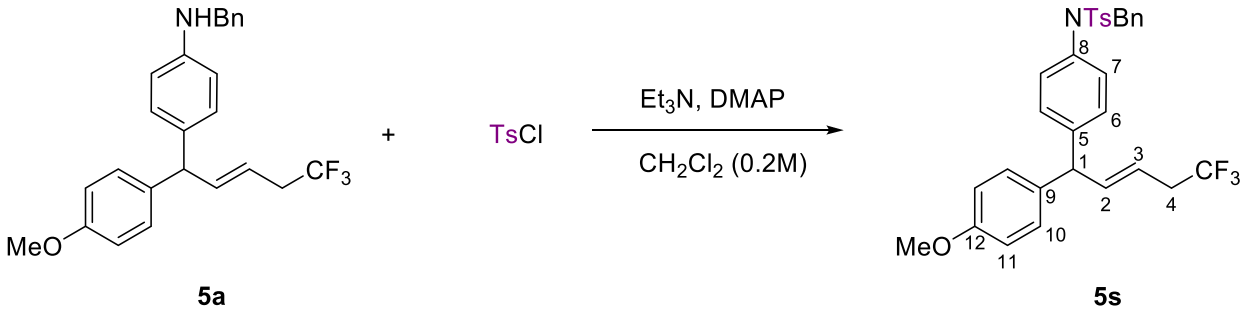

From aniline **5a** (200.0 mg, 0.486 mmol), triethylamine (102 μL, 0.729 mmol, 1.5 equiv.), dimethylaminopyridine (5.9 mg, 0.049 mmol, 0.1 equiv.) and *p*ara-toluenesulfonyl chloride (111.0 mg, 0.583 mmol, 1.2 equiv.) in 2.4 mL of dry CH_2Cl_2, following the general procedure, sulfonamide **5s** was obtained. Chromatographic purification (gradient elution: 0:100 → 30:70 Et_2O – hexane) gave **5s** (263.2 mg, 96%), as a colourless oil.

Data for **5s**: ***R**$_f$* 0.20 (30% Et_2O – hexane). **^{1}H NMR (300 MHz, $CDCl_3$)** δ 7.56 (2H, d, *J* = 8.2 Hz, Ts), 7.28 (2H, d, *J* = 8.4 Hz, Ts), 7.25 – 7.20 (5H, m, Ar), 7.07 – 6.90 (6H, m, Ar), 6.84 (1H, d, *J* = 8.7 Hz, Ar), 6.09 (1H, dd, *J* = 15.3 and 7.2 Hz, 2-H), 5.25 (1H, dtd, *J* = 15.5, 7.1 and 1.4 Hz, 3-H), 4.72 (2H, s, CH_2 Bn), 4.64 (1H, d, *J* = 7.1 Hz, 1-H), 3.80 (3H, s, OMe), 2.84 (2H, qd, *J* = 10.6 and 6.9 Hz, 4-H_2), 2.45 (3H, s, Me Ts).

(*E*)-*N*-Benzyl-*N*-{3-bromo-4-[5,5,5-trifluoro-1-(4-methoxyphenyl)pent-2-en-1-yl]phenyl}-4-methylbenzenesulfonamide, 5t.

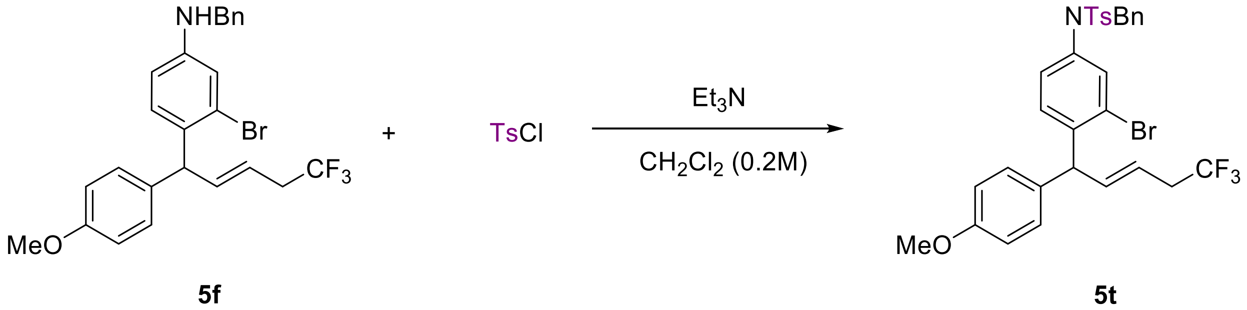

From aniline **5f** (154.4 mg, 0.315 mmol), triethylamine (65.8 μL, 0.472 mmol, 1.5 equiv.) and *p*ara-toluenesulfonyl chloride (72.0 mg, 0.378 mmol, 1.2 equiv.) in 1.6 mL of dry CH_2Cl_2, following the general procedure, sulfonamide **5t** was obtained. Chromatographic purification (gradient elution: 0:100 → 30:70 Et_2O – hexane) gave **5t** (135.5 mg, 67%), as a colourless oil.

Data for **5t**: ***R**$_f$* 0.20 (30% Et_2O – hexane). **^{1}H NMR (300 MHz, $CDCl_3$)** δ 7.57 (2H, d, *J* = 8.2 Hz, Ts), 7.31 (2H, d, *J* = 8.0 Hz, Ts), 7.24 (6H, m, Ar), 7.06 – 6.90 (4H, m, Ar), 6.85 (2H, d, *J* = 8.7

Hz, Ar), 6.05 (1H, dd, J = 15.4 and 6.3 Hz, 2-H), 5.22 – 5.13 (1H, m, 3-H), 5.13 – 5.05 (1H, m, 1-H), 4.70 (2H, s, CH_2 Bn), 3.79 (3H, s, OMe), 3.00 – 2.73 (2H, m, 4-H_2), 2.46 (3H, s, Me).

***tert*-Butyl (*E*)-{3-hydroxy-4-[4-(4-methoxyphenyl)but-3-en-2-yl]phenyl}carbamate, 4y.**

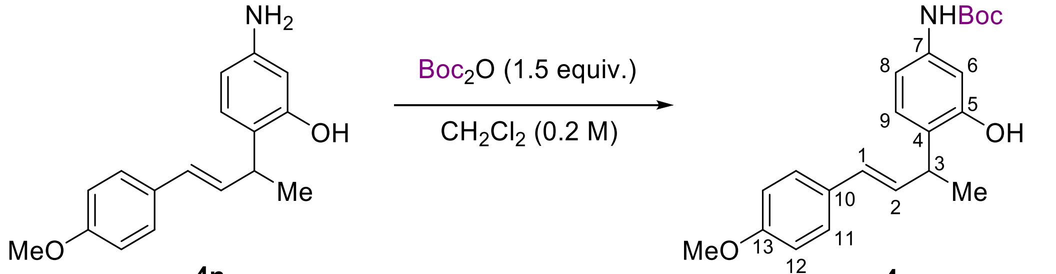

From aniline **4n** (150 mg, 0.557 mmol) and Boc_2O (182 mg, 0.835 mmol, 1.5 equiv.) in 0.5 mL of dry CH_2Cl_2, following the general procedure, carbamate **4y** was obtained. Chromatographic purification (gradient elution: 10:90 → 20:80 EtOAc – hexane) gave **4y** (144.1 mg, 70%), as a colourless oil.

Data for **4y**: R_f 0.4 (40% EtOAc – hexane). **^{1}H NMR (300 MHz, $CDCl_3$)** δ 7.29 (2H, d, J = 8.7 Hz, 11-H), 7.14 (1H, s, OH), 7.07 (1H, d, J = 8.3 Hz, 9-H), 6.84 (2H, d, J = 8.7 Hz, 12-H), 6.72 (1H, dd, J = 8.2 and 2.2 Hz, 8-H), 6.62 (1H, s, 6-H), 6.41 (1H, d, J = 16.1 Hz, 1-H), 6.28 (1H, dd, J = 16.0 and 6.0 Hz, 2-H), 6.16 (1H, s, NH), 3.96 – 3.84 (1H, m, 3-H), 3.79 (3H, s, OMe), 1.52 (9H, s, ^{t}Bu Boc), 1.42 (3H, d, J = 7.0 Hz, Me).

***tert*-Butyl (*E*)-{4-[5,5,5-trifluoro-1-(4-methoxyphenyl)pent-2-en-1-yl]phenyl}carbamate, 5u.**

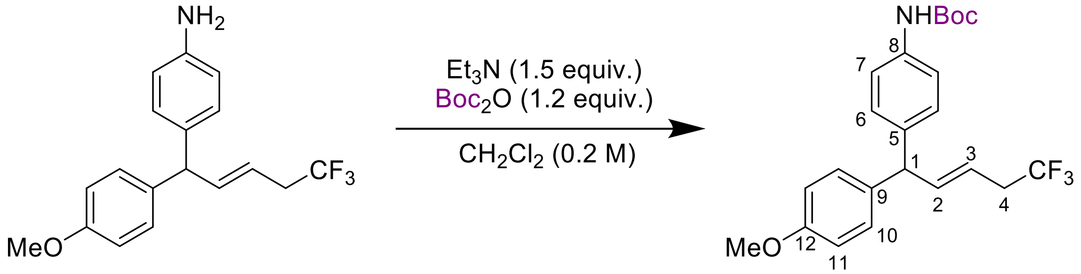

From aniline **5r** (30.0 mg, 0.093 mmol), triethylamine (20 μL, 0.15 mmol, 3.0 equiv.) and Boc_2O (24 mg, 0.11 mmol, 1.2 equiv.) in 0.5 mL of dry CH_2Cl_2, following the general procedure, carbamate **5u** was obtained. Chromatographic purification (gradient elution: 0:100 → 40:60 EtOAc – hexane) gave **5u** (18.7 mg, 48%), as a yellow oil.

Data for **5u**: R_f 0.50 (40% EtOAc – hexane). **^{1}H NMR (400 MHz, $CDCl_3$)** δ 7.29 (2H, d, J = 8.3 Hz, CH Ar), 7.07 (2H, d, J = 8.3 Hz, CH Ar), 7.05 (2H, d, J = 8.5 Hz, CH Ar), 6.83 (2H, d, J = 8.7 Hz, CH Ar), 6.43 (1H, s, NH), 6.12 (1H, dd, J = 15.4 and 7.3 Hz, 2-H), 5.34 (1H, dtd, J = 15.4,

7.1 and 1.3 Hz, 3-H), 4.66 (1H, d, J = 7.3 Hz, 1-H), 3.78 (3H, s, OMe), 2.84 (2H, qd, J = 10.7 and 7.0 Hz, 4-H_2), 1.51 (9H, s, ^{t}Bu Boc). **^{13}C NMR (101 MHz, $CDCl_3$)** δ 158.2 (1C, C-12), 152.8 (1C, C=O), 140.6 (1C, C-2), 137.8 (1C, C Ar), 136.7 (1C, C Ar), 135.03 (1C, C Ar), 129.4 (2C, CH Ar), 129.0 (2C, CH Ar), 125.9 (1C, q, J = 276.2 Hz, CF_3), 119.3 (1C, q, J = 3.7 Hz, C-3), 118.7 (2C, CH Ar), 113.9 (2C, CH Ar), 80.5 (1C, C Boc), 55.3 (1C, OMe), 52.3 (1C, C-1), 37.3 (1C, q, J = 29.7 Hz, C-4), 28.3 (3C, ^{t}Bu Boc). **^{19}F NMR (376 MHz, $CDCl_3$)** δ –66.4 (3F, CF_3).

7. Functionalization of hydroarylation products 4.

7.1. Alkene reduction: synthesis of 4-[4-(4-Methoxyphenyl)butan-2-yl]-*N*-methylaniline, 7.

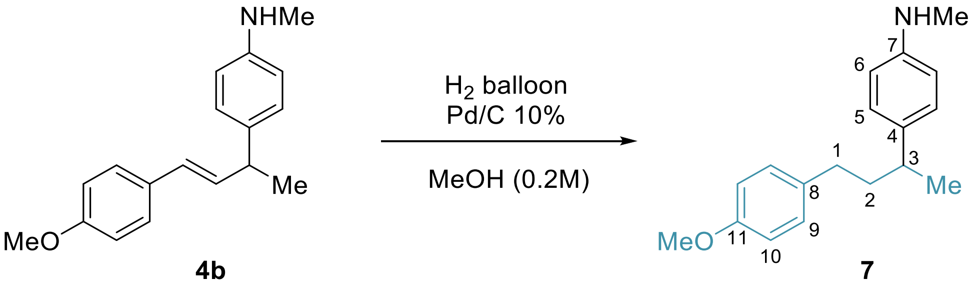

To an oven-dried vial, aniline **4b** (26.7 mg, 0.1 mmol, 1 equiv.) and 10% Pd/C (1.1 mg, 4 wt %) were added. Then, MeOH (0.2 M) was added and the reaction mixture was kept under hydrogen for 16h. The reaction mixture was diluted with dichloromethane, filtered through a pad of celite and evaporated under reduced pressure. The residue was purified by column chromatography (gradient elution: 0:100 → 15:85 Et_2O – hexane) to give aniline **7** (28.2 mg, 93%).

Data for **7**: **R*f*** 0.20 (30% Et_2O – hexane). **^{1}H NMR (400 MHz, $CDCl_3$)** δ 7.06 (2H, d, J = 8.6 Hz, 9-H), 7.06 (2H, d, J = 8.6 Hz, 10-H), 6.82 (2H, d, J = 8.5 Hz, 5-H), 6.64 (1H, d, J = 8.5 Hz, 6-H), 3.79 (3H, s, OMe), 2.85 (3H, s, NMe), 2.63 (1H, h, J = 7.0 Hz, 3-H), 2.47 (1H, t, J = 7.9 Hz, 1-H), 1.92 – 1.77 (2H, m, 2-H_2), 1.24 (3H, d, J = 6.9 Hz, 4-H). **^{13}C NMR (101 MHz, $CDCl_3$)** δ 157.7 (1C, C Ar), 147.1 (1C, C Ar), 136.9 (1C, C Ar), 135.0 (1C, C Ar), 129.4 (2C, C-6), 127.9 (2C, C-10), 113.8 (2C, C-11), 113.1 (2C, C-8), 55.4 (1C, OMe), 40.5 (1C, C-3), 38.6 (1C, C-1), 33.2 (1C, C-2), 31.4 (1C, NMe), 22.9 (1C, Me). **HRMS** (ESI): calculated for $C_{18}H_{24}NO$ $[M+H]^+$ requires *m/z* 270.1852, found 270.1843.

7.2. Alkene oxidation: synthesis of *N*,4-Dimethyl-*N*-[4-(4-oxobutan-2-yl)phenyl]benzenesulfonamide, 8.

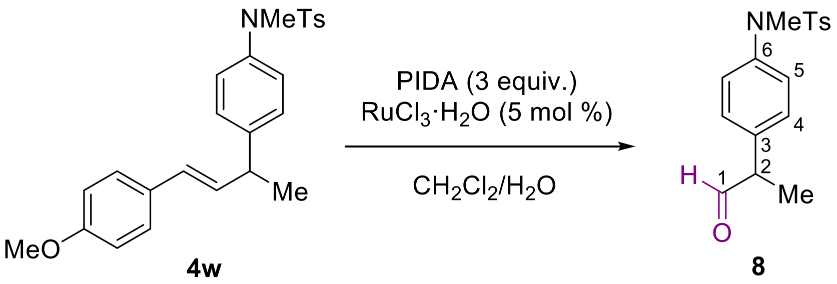

To a stirring solution of aniline **4w** (19.2 mg, 0.046 mmol, 1 equiv.) in 5.0 mL/mmol of a 4:1 mixture of CH_2Cl_2: H_2O, PIDA (3.0 equiv.) was added in one portion. The mixture was stirred at that temperature for 15 min and $RuCl_3{\cdot}H_2O$ (0.5 mg, 5 mol %) was added in one portion. The mixture was warmed up to room temperature. The reaction was monitored by TLC until completion, diluted with CH_2Cl_2 and H_2O, and extracted with CH_2Cl_2. The combined organic layers were dried using Na_2SO_4, filtered and the solvent was evaporated under reduced pressure. The residue was purified by column chromatography (gradient elution: 0:100 → 30:70 Et_2O – hexane) to give aniline **8** (5.5 mg, 40%).

Data for **8**: **R*f*** 0.20 (30% Et_2O – hexane). **^{1}H NMR (400 MHz, $CDCl_3$)** δ 9.66 (1H, d, *J* = 1.4 Hz, CHO), 7.43 (2H, d, *J* = 8.3 Hz, Ts), 7.24 (2H, d, *J* = 8.6 Hz, Ts), 7.15 (2H, d, *J* = 8.6 Hz, Ar), 7.11 (2H, d, *J* = 8.8 Hz, Ar), 3.63 (1H, qd, *J* = 7.1 and 1.5 Hz, 2-H), 3.14 (3H, s, NMe), 2.42 (3H, s, Me Ts), 1.43 (3H, d, *J* = 7.1 Hz, Me). **^{13}C NMR (101 MHz, $CDCl_3$)** δ 200.8 (1C, C=O), 143.8 (1C, C Ar), 141.1 (1C, C Ar), 136.8 (1C, C Ar), 133.6 (1C, C Ar), 129.5 (2C, CH Ar), 128.9 (2C, CH Ar), 128.0 (2C, CH Ar), 127.3 (2C, CH Ar), 52.6 (1C, C-2), 38.1 (NMe), 21.7 (1C, Me Ts), 14.7 (1C, C-4). **HRMS** (ESI): calculated for $C_{17}H_{19}NNaO_3S$ $[M+Na]^+$ requires *m/z* 340.0978, found 340.0975.

7.3. Alkene hydroetherification: synthesis of *N*-[2-(4-Methoxyphenyl)-4-methylchroman-7-yl]-4-methylbenzenesulfonamide, 9.

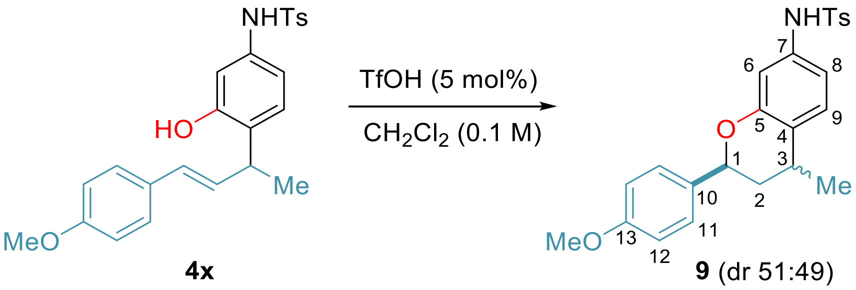

To a solution of aniline **4x** (17 mg, 0.040 mmol, 1.0 equiv.) in CH_2Cl_2 (0.1 M), TfOH (0.122 µL, 5 mol %), was added and the reaction mixture was stirred at 20 ºC for 8 h. Then, the mixture was quenched with solid $NaHCO_3$ and filtered off through a wool pad. The solvent was evaporated under

reduced pressure and the residue was purified by column chromatography (gradient elution: 20:80 → 40:60 EtOAc – hexane) to give product **9** (7.6 mg, 66%, dr 51:49) as colourless oil.

Data for **9** (diastereoisomer 1, from the mixture): **R*f*** 0.20 (40% EtOAc – hexane). **^{1}H NMR (400 MHz, CDCl$_3$)** δ 7.69 (2H, d, *J* = 8.3 Hz, Ts), 7.37 – 7.27 (2H, m, 11-H), 7.24 (2H, d, *J* = 8.1 Hz, Ts), 7.00 (1H, d, *J* = 8.2 Hz, 9-H), 6.92 (2H, d, *J* = 8.7 Hz, 12-H), 6.68 – 6.64 (1H, m, 8-H), 6.57 (1H, d, *J* = 2.3 Hz, 6-H), 6.42 (1H, s, NH), 5.01 (1H, dd, *J* = 10.7 and 2.3 Hz, 1-H), 3.82 (3H, s, OMe), 2.95 – 2.82 (1H, m, 3-H) 2.39 (3H, s, Me Ts), 2.24 – 2.04 (1H, m, 2-H$_A$), 1.85 (1H, dt, *J* = 13.8 and 2.6 Hz, 2-H$_B$), 1.34 (3H, d, *J* = 7.2 Hz, Me). **^{13}C NMR (101 MHz, CDCl$_3$)** δ 159.6 (1C, C Ar), 155.2 (1C, C Ar), 143.9 (1C, C Ar), 136.6 (1C, C Ar), 135.7 (1C, C Ar), 133.6 (1C, C Ar), 130.0 (1C, C-9), 129.8 (2C, CH Ts), 127.5 (2C, C-11), 127.4 (2C, CH Ts), 124.5 (1C, C Ar), 114.1 (2C, C-12), 113.8 (1C, C-8), 109.9 (1C, C-6), 73.5 (1C, C-1), 55.5 (1C, OMe), 36.7 (1C, C-2), 28.3 (1C, C-3), 23.9 (1C, Me), 21.7 (1C, Me Ts). **HRMS** (ESI): calculated for $C_{24}H_{25}NNaO_4S$ $[M+Na]^+$ requires m/z 446.1397, found 446.1384.

Data for **9** (diastereoisomer 2, from the mixture): The NMR signals overlapped, except for: **^{1}H NMR (400 MHz, CDCl$_3$)** δ 7.11 (1H, dd, *J* = 8.3 and 0.7 Hz, CH Ar), 6.91 (2H, d, *J* = 8.7 Hz, 12-H), 6.54 (1H, d, *J* = 2.3 Hz, 6-H), 4.96 (1H, dd, *J* = 11.6 and 1.9 Hz, 1-H), 3.82 (3H, s, OMe), 3.17 – 2.99 (1H, m, 3-H), 1.75 (1H, dt, *J* = 13.6 and 11.8 Hz, 2-H$_B$), 1.30 (3H, d, *J* = 6.8 Hz, Me). **^{13}C NMR (101 MHz, CDCl$_3$)** δ 159.5 (1C, C Ar), 155.6 (1C, C Ar), 135.6 (1C, C Ar), 128.0 (1C, C-9), 127.6 (2C, C-11), 124.5 (1C, C Ar), 114.1 (2C, C-12), 113.7 (1C, C-8), 109.7 (1C, C-6), 78.1 (1C, C-1), 39.6 (1C, C-2), 29.9 (1C, C-3), 20.2 (1C, Me).

7.4. Alkene iodoetherification, synthesis of *N*-(3-iodo-2-(4-methoxyphenyl)-4-methylchroman-7-yl)-4-methylbenzenesulfonamide, 10.

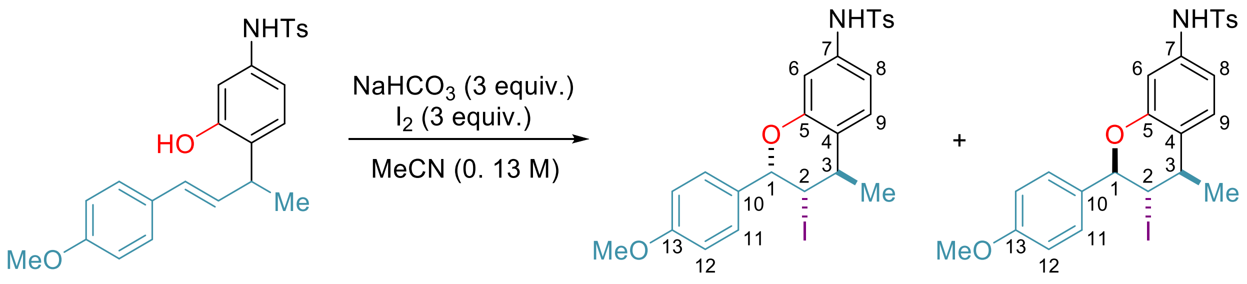

4x **10** (dr 2:1)

Following a reported procedure[5] under unoptimized conditions, to an oven-dried 10 mL vial, aniline **4x** (17 mg, 0.04 mmol, 1.0 equiv.), $NaHCO_3$ (10.1 mg, 0.12 mmol, 3.0 equiv.) and dry MeCN (0.13 M) were added. While stirring the reaction mixture at 0ºC, I_2 (30.6 mg, 0.120 mmol, 3.0 equiv.) was added and the reaction mixture was allowed to stir at 25 ºC for 2 h. The reaction mixture was monitored by TLC until completion. Then, the mixture was quenched with a saturated aqueous

$Na_2S_2O_3$ solution and extracted with EtOAc. The combined organic phase was dried over anhydrous Na_2SO_4 and the solvent evaporated under reduced pressure. The residue was purified by column chromatography (gradient elution: 0:100 → 35:65 EtOAc – hexane) to give product **10** (13.1 mg, 60%, dr 2:1) as yellow oil.

Data for *cis-trans*-**10** (major diastereoisomer, from the mixture): **R*f*** 0.25 (40% EtOAc – hexane). **^{1}H NMR (400 MHz, $CDCl_3$)** δ 7.73 (2H, d, *J* = 8.0 Hz, Ts), 7.32 (1H, d, *J* = 8.6 Hz, 9-H), 7.30 – 7.27 (2H, m, Ts), 7.19 (2H, d, *J* = 8.8 Hz, 11-H), 6.89 (2H, d, *J* = 8.7 Hz, 12-H), 6.70 (1H, dd, *J* = 8.3 and 2.3 Hz, 8-H), 6.64 (1H, d, *J* = 2.2 Hz, 6-H), 5.51 (1H, d, *J* = 5.8 Hz, 1-H), 4.67 (1H, dd, *J* = 5.8 and 4.0 Hz, 2-H), 3.83 (3H, s, OMe), 2.64 – 2.53 (1H, m, 3-H), 2.43 (3H, s, Me Ts), 1.37 (3H, d, *J* = 6.7 Hz, Me). **^{13}C NMR (101 MHz, $CDCl_3$)** δ 159.7 (1C, C Ar), 153.1 (1C, C Ar), 144.0 (1C, C Ar), 136.7 (1C, C Ar), 136.4 (1C, C Ar), 132.6 (1C, C Ar), 129.8 (2C, CH Ts) , 129.0 (1C, C-9), 127.5 (2C, C-11), 127.5 (2C, CH Ts), 121.9 (1C, C Ar), 114.1 (2C, C-12), 114.0 (1C, C-8), 109.2 (1C, C-6), 80.5 (1C, C-1), 55.5 (1C, OMe), 37.9 (1C, C-2), 32.5 (1C, C-3), 22.1 (1C, Me), 21.7 (1C, Me Ts). **NOESY-2D (400 MHz, $CDCl_3$)**: between 1-H and 2-H, between 1-H and Me, between 2-H and 3-H, between 2-H and Me. **HRMS** (ESI): calculated for $C_{24}H_{24}INNaO_4S$ $[M+Na]^+$ requires m/z 572.0363, found 572.0346.

Partial data for *trans-trans*-**10** (minor diastereoisomer, from the mixture): The NMR signals overlapped with major isomer, except for: **^{1}H NMR (400 MHz, $CDCl_3$)** δ 7.72 (2H, d, *J* = 8.2 Hz, Ts), 7.12 (1H, d, *J* = 8.5 Hz, 9-H), 6.97 (2H, d, *J* = 8.2 Hz, Ar), 6.95 (2H, d, *J* = 8.7 Hz, Ar), 6.73 (1H, dd, *J* = 8.4 and 2.3 Hz, 8-H), 6.55 (1H, d, *J* = 2.3 Hz, 6-H), 5.04 (1H, d, *J* = 10.8 Hz, 1-H), 4.25 (1H, t, *J* = 10.5 Hz, 2-H), 3.86 (3H, s, OMe), 3.55 – 3.43 (1H, m, 3-H), 2.42 (3H, s, Me Ts), 1.56 (3H, d, *J* = 6.7 Hz, Me). **^{13}C NMR (101 MHz, $CDCl_3$)** δ 160.2 (1C, C Ar), 155.0 (1C, C Ar), 144.0 (1C, C Ar), 136.3 (1C, C Ar), 136.2 (1C, C Ar), 132.3 (1C, C Ar), 129.9 (2C, CH Ts), 128.4 (1C, C-9), 128.3 (2C, C-11), 127.4 (2C, CH Ts), 121.8 (1C, C Ar), 114.5 (1C, C-8), 113.9 (2C, C-12), 109.4 (1C, C-6), 83.1 (1C, C-1), 41.7 (1C, C-3), 40.3 (1C, C-2), 21.7 (1C, Me), 21.3 (1C, Me). **NOESY-2D (400 MHz, $CDCl_3$)**: between 1-H and 3-H, between 2-H and Me.

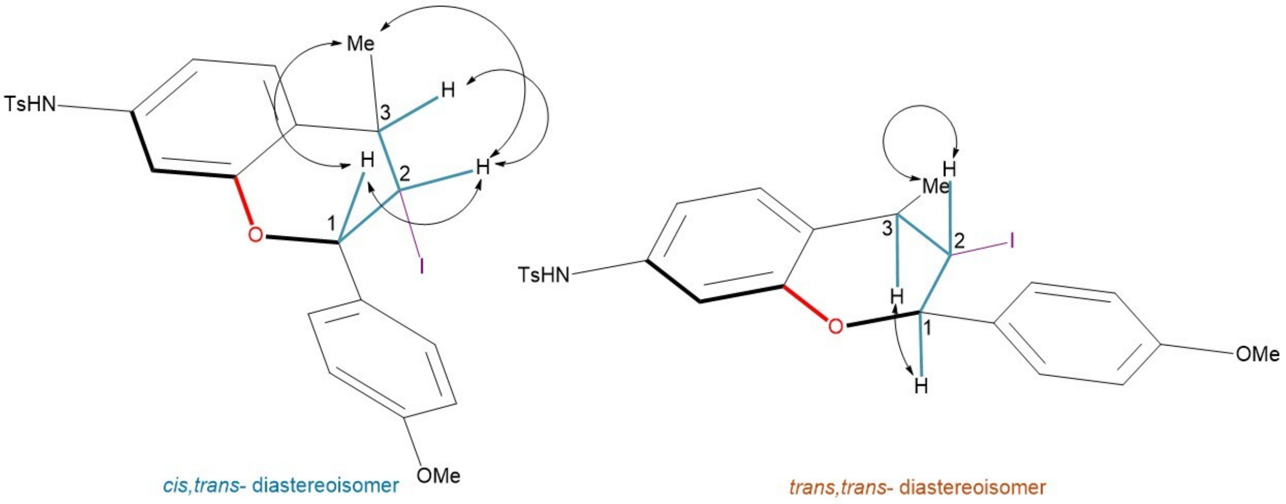

The ^{1}H NMR spectrum of **10** (cis,trans) presents 1-H as a doblet (J = 5.8 Hz) that has COSY cross-peaks with 2-H and Me, while 2-H is a doblet of doblets (J = 5.6 and 5.5 Hz) that has COSY cross-peaks with 1-H and Me. Moreover 3-H appears as a multiplet that has COSY cross-peaks with Me.

The ^{1}H NMR spectrum of **10** (trans,trans) presents 1-H as a doblet (J = 10.8 Hz) that has COSY cross-peaks with 2-H and 3-H, while 2-H is a triplet (J = 10.5 Hz) that has COSY cross-peaks with 3-H and Me. Moreover 3-H appears as a multiplet that has COSY cross-peaks with Me.

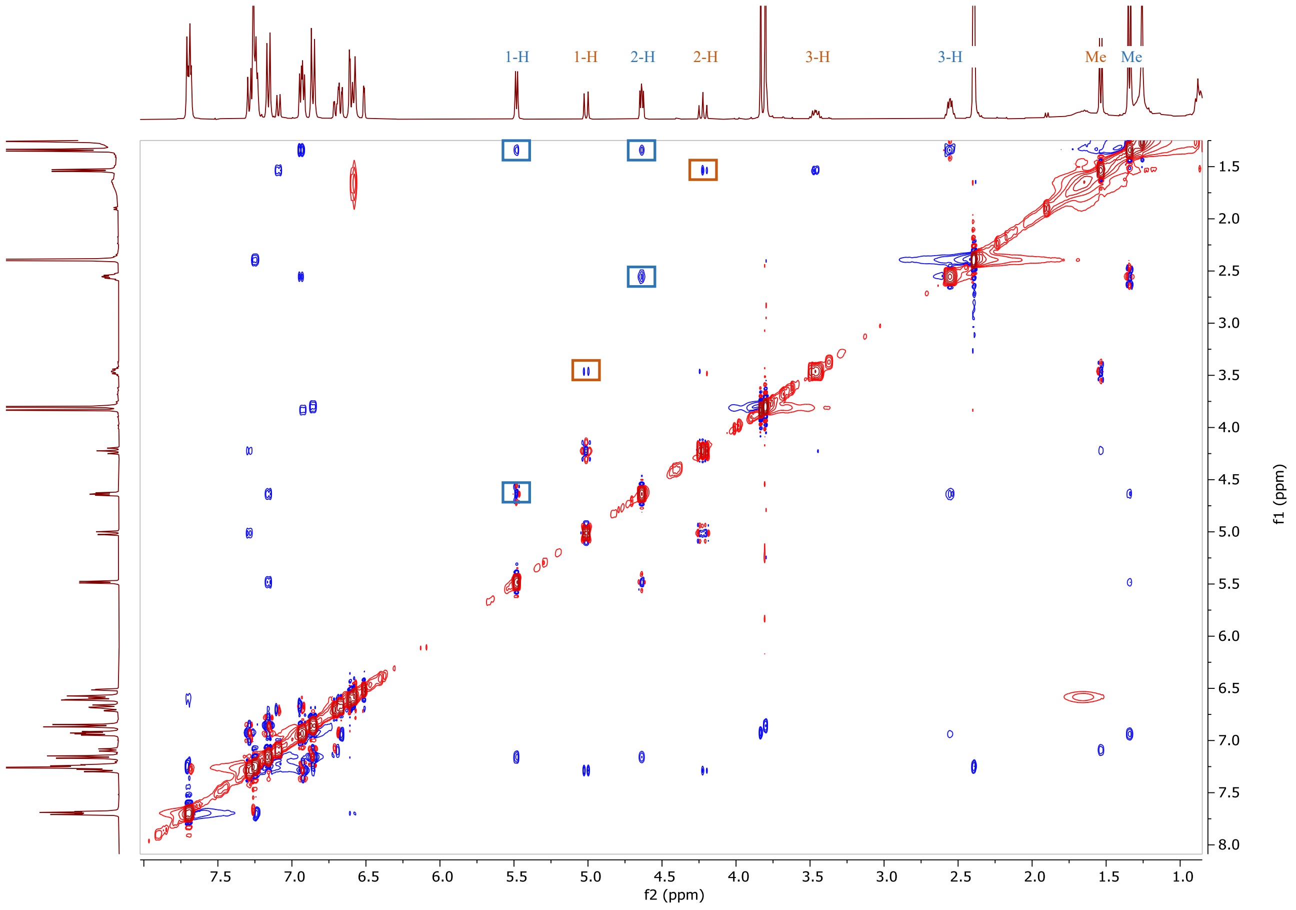

7.5. Palladium-catalyzed cyclization: synthesis of *tert*-Butyl (2-[4-methoxybenzyl]-3-methylbenzofuran-6-yl)carbamate, 11.

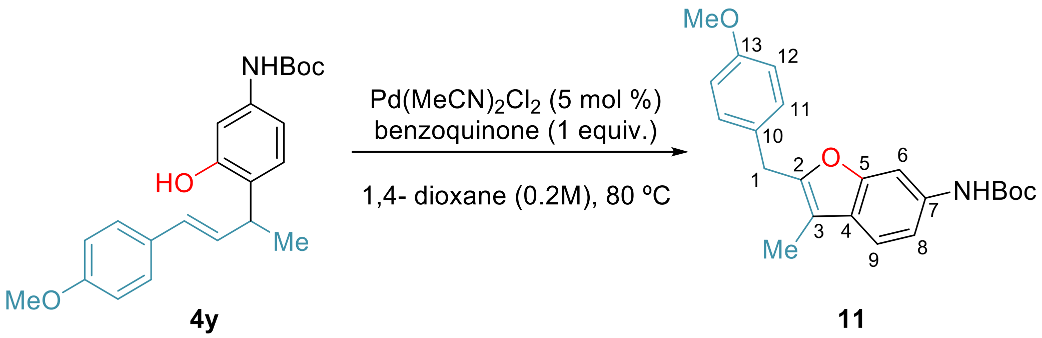

Following an adapted reported procedure[6] under unoptimized conditions, to an oven-dried 10 mL vial, aniline **4y** (18.6 mg, 0.050 mmol, 1.0 equiv.), $Pd(MeCN)_2Cl_2$ (0.6 mg, 5 mol %) and benzoquinone (7 mg, 0.050 mmol, 1.0 equiv.) were added. Three vacuum-argon cycles were performed and dry 1,4 dioxane (0.2 M) was added under argon atmosphere. The reaction mixture was stirred at 80 ºC for 2 h and monitored by TLC until completion. Then, the mixture was cooled to room temperature and filtered through Celite. The filtrate was diluted with H_2O and extracted with ethyl acetate. The combined organic phase was dried over anhydrous Na_2SO_4 and the solvent evaporated under reduced pressure. The residue was purified by column chromatography (gradient elution: 0:100 → 30:70 Et_2O – hexane) to give product **11** (6.9 mg, 40%) as colourless oil.

Data for **11**: **R_f** 0.30 (40% Et_2O – hexane). **^{1}H NMR (400 MHz, $CDCl_3$)** δ 7.57 (1H, s, 6-H), 7.30 (1H, d, J = 8.3 Hz, 9-H), 7.15 (2H, d, J = 8.6 Hz, 11-H), 7.05 (1H, dd, J = 8.2 and 1.9 Hz, 8-H), 6.82 (2H, d, J = 8.6 Hz, 12-H), 6.50 (1H, s, NH), 3.99 (2H, s, 1-H_2), 3.77 (3H, s, OMe), 2.17 (3H, s, Me), 1.52 (9H, s, ^{t}Bu Boc). **^{13}C NMR (101 MHz, $CDCl_3$)** δ 158.4 (1C, C-13), 154.6 (1C, C=O), 153.0 (1C, C-5), 152.5 (1C, C-2), 134.8 (1C, C-7), 130.3 (1C, C-10), 129.6 (2C, C-11), 126.2 (1C, C-4), 118.8 (1C, C-9), 114.1 (3C, C-8 and C-12), 110.4 (1C, C-3), 102.1 (1C, C-6), 80.6 (1C, C Boc), 55.4 (1C, OMe), 31.8 (1C, C-1), 28.5 (3C, ^{t}Bu Boc), 8.1 (1C, Me). **HRMS** (ESI): calculated for $C_{22}H_{25}NNaO_4$ $[M+Na]^+$ requires m/z 390.1676, found 390.1671.

8. Functionalization of trifluoromethylarylation products, 5.

8.1. Borylation: synthesis of (*E*)-*N*-Benzyl-4-methyl-*N*-{3-[4,4,5,5-tetramethyl-1,3,2-dioxaborolan-2-yl]-4-[5,5,5-trifluoro-1-(4-methoxyphenyl)pent-2-en-1-yl]phenyl}benzenesulfonamide, 12.

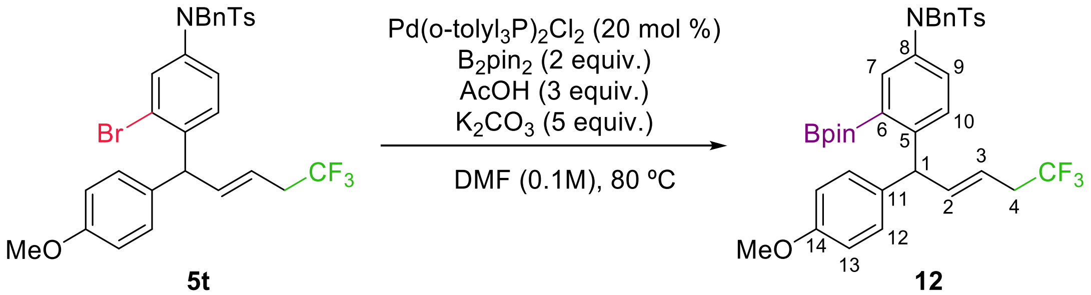

5t **12**

Under unoptimized conditions, to an oven-dried 10 mL vial, aniline **5t** (26.0 mg, 0.040 mmol, 1.0 equiv.), B_2pin_2 (20 mg, 0.080 mmol, 2.0 equiv.), and K_2CO_3 (28 mg, 0.20 mmol, 5.0. equiv.) were added. Three vacuum-argon cycles were performed and dry DMF (0.1 M) was added under argon atmosphere. While stirring the reaction mixture at room temperature, AcOH (6.9 µL, 0.12 mmol, 3.0 equiv.) was added and the reaction mixture was warmed to 80 ºC and stirred for 16 h. Then, the mixture was diluted with EtOAc and extracted with H_2O three times. The solvent was evaporated under reduced pressure and the residue was purified by column chromatography (gradient elution: 10:90 → 30:70 Et_2O – hexane) to give the product **12** (18.9 mg, 68%) as colourless oil.

Data for **12**: $\mathbf{R_f}$ 0.10 (30% Et_2O – hexane).**^{1}H NMR (400 MHz, CDCl$_3$)** δ 7.54 (2H, d, *J* = 8.3 Hz, Ts), 7.31 (1H, d, *J* = 2.4 Hz, 7-H), 7.28 – 7.18 (7H, m, Ar), 7.02 – 6.92 (4H, m, Ar), 6.79 (2H, d, *J* = 8.7 Hz, 13-H), 6.07 (1H, dd, *J* = 15.4 and 6.6 Hz, 2-H), 5.60 (1H, d, *J* = 6.7 Hz, 1-H), 5.11 (1H, dtd, *J* = 15.7, 7.1 and 1.5 Hz, 3-H), 4.74 (1H, d, *J* = 14.5 Hz, CH_A Bn), 4.66 (1H, d, *J* = 14.4 Hz, CH_B Bn), 3.77 (3H, s, OMe), 2.80 (2H, qd, *J* = 10.7 and 7.0 Hz, 4-H_2), 2.43 (3H, s, Me), 1.22 (12H, s, 4 x Me Bpin). **^{13}C NMR (101 MHz, CDCl$_3$)** δ 158.1 (1C, C-14), 149.3 (1C, C Ar), 143.4 (1C, C Ar), 141.7 (1C, C-2), 137.0 (1C, C Ar), 136.4 (1C, C Ar), 136.1 (1C, C-7), 136.0 (1C, C Ar), 135.6 (1C, C Ar), 132.2 (1C, C Ar), 130.0 (2C, CH Ar), 129.6 (1C, CH Ar), 129.5 (2C, CH Ar), 129.0 (1C, CH Ar), 128.7 (2C, CH Ar), 128.4 (2C, CH Ar), 128.0 (2C, CH Ts), 127.6 (1C, CH Ar), 126.1 (1C, q, *J* = 276.7 Hz, CF_3), 119.3 (1C, q, *J* = 3.8 Hz, C-3), 113.7 (2C, C-13), 83.8 (2C, 2 x C Bpin), 55.4 (1C, OMe), 55.1 (1C, CH_2 Bn), 49.7 (1C, C-1), 37.5 (1C, q, *J* = 29.7 Hz, C-4), 24.90 (2C, 2 x Me Bpin) 24.86 (2C, 2 x Me Bpin), 21.7 (1C, Me Ts). **^{19}F NMR (376 MHz, CDCl$_3$)** δ –67.3 (3F, CF_3). **HRMS** (ESI): calculated for $C_{38}H_{42}BF_3NO_5S$ $[M+H]^+$ requires m/z 692.2830, found 692.2800.

8.2. Suzuki-Miyaura cross-coupling: synthesis of (*E*)-*N*-Benzyl-*N*-{4'-methoxy-6-[5,5,5-trifluoro-1-(4-methoxyphenyl)pent-2-en-1-yl]-[1,1'-biphenyl]-3-yl}-4-methylbenzenesulfonamide, 13.

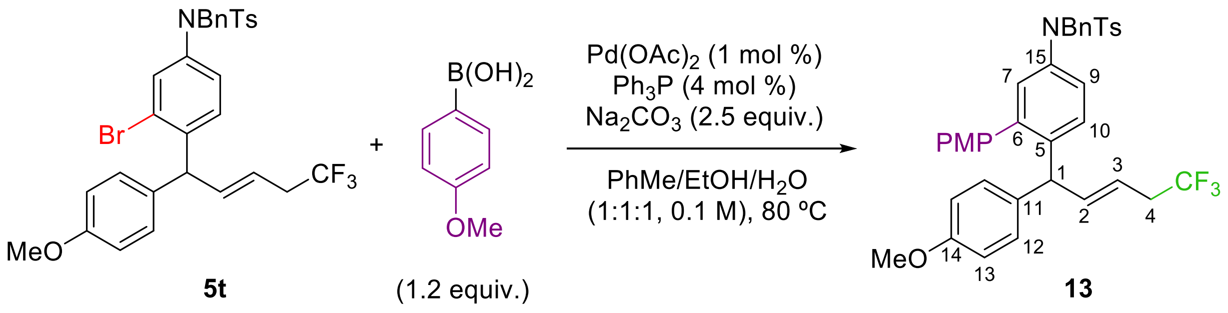

Following a reported procedure[7] under unoptimized conditions, to an oven-dried 10 mL vial, $Pd(OAc)_2$ (0.11 mg, 1 mol %), Ph_3P (0.52 mg, 4 mol %), aniline **5t** (32.0 mg, 0.050 mmol, 1.0 equiv.), (4-methoxyphenyl)boronic acid (9.1 mg, 0.060 mmol, 1.2 equiv.) and Na_2CO_3 (13 mg, 0.13 mmol, 2.5 equiv.) were added. Three vacuum-argon cycles were performed and dry and degassed Toluene:EtOH:H_2O (1:1:1, 0.1 M) was added under argon atmosphere. The reaction mixture was warmed to 80 ºC and stirred for 16 h. Then, the mixture was diluted with EtOAc and extracted with H_2O three times. The solvent was evaporated under reduced pressure and the residue was purified by column chromatography (gradient elution: 0:100 → 30:70 Et_2O – hexane) to give product **13** (16.1 mg, 48%) as colourless oil.

Data for **13**: **R*f*** 0.10 (30% Et_2O – hexane). **^{1}H NMR (400 MHz, $CDCl_3$)** δ 7.62 (2H, d, *J* = 8.3 Hz, Ts), 7.36 – 7.31 (2H, m, Ar), 7.30 – 7.26 (5H, m, Ar), 7.09 – 7.03 (2H, m, Ar), 6.96 (2H, d, *J* = 8.6 Hz, 12-H), 6.89 (4H, d, *J* = 7.4 Hz, Ar), 6.83 (2H, d, *J* = 8.7 Hz, 13-H), 6.79 – 6.76 (1H, m, Ar), 6.04 (1H, dd, *J* = 15.4 and 6.5 Hz, 2-H), 5.20 – 4.95 (1H, m, 3-H), 4.79 (1H, d, *J* = 14.3 Hz, CH_A Bn), 4.77 (1H, d, *J* = 7.3 Hz, 1-H), 4.73 (1H, d, *J* = 14.4 Hz, CH_B Bn), 3.89 (3H, s, OMe), 3.84 (3H, s, OMe), 2.86 (2H, qd, *J* = 10.7 and 7.1 Hz, 4-H_2), 2.49 (3H, s, Me Ts). **^{13}C NMR (101 MHz, $CDCl_3$)** δ 159.0 (1C, C Ar), 158.2 (1C, C Ar), 143.7 (1C, C Ar), 142.4 (1C, C Ar), 140.9 (1C, C Ar), 140.3 (1C, C-2), 137.1 (1C, C Ar), 136.2 (1C, C Ar), 135.8 (1C, C Ar), 134.9 (1C, C Ar), 132.8 (1C, C Ar), 130.4 (1C, CH Ar), 130.2 (2C, C-12), 129.58 (2C, CH), 129.56 (2C, CH Ar), 129.54 (1C, CH Ar), 128.7 (2C, CH Ar), 128.44 (2C, CH Ar), 128.40 (1C, CH Ar), 127.9 (2C, CH Ar), 127.7 (1C, CH Ar), 126.0 (1C, q, *J* = 276.7 Hz, CF_3), 119.8 (1C, q, *J* = 3.5 Hz, C-3), 113.8 (2C, C-13), 113.5 (2C, CH), 55.43 (1C, OMe), 55.37 (1C, OMe), 54.9 (1C, CH_2 Bn), 48.5 (1C, C-1), 37.4 (1C, q, *J* = 29.7 Hz, C-4), 21.7 (1C, Me). **^{19}F NMR (376 MHz, $CDCl_3$)** δ –67.3 (3F, CF_3). **HRMS** (ESI): calculated for $C_{39}H_{37}F_3NO_4S$ $[M+H]^+$ requires m/z 672.2390, found 672.2361.

8.3. Heck reaction: synthesis of ethyl (*E*)-3-{5-[(*N*-benzyl-4-methylphenyl)sulfonamido]-2-[(*E*)-5,5,5-trifluoro-1-(4-methoxyphenyl)pent-2-en-1-yl]phenyl}acrylate, 14.

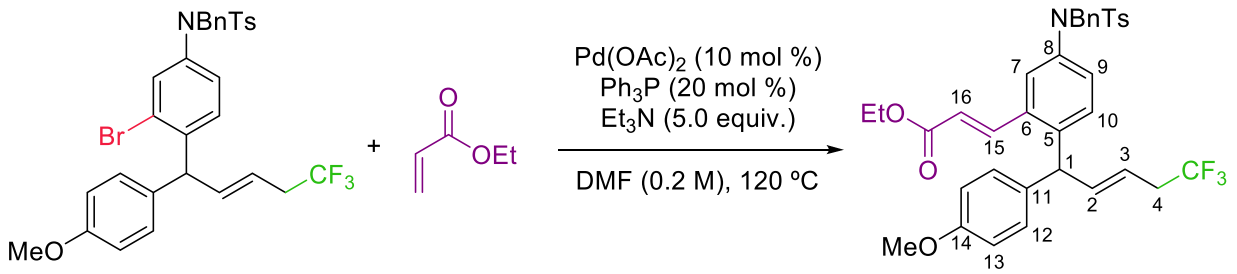

5t (3.0 equiv.) **14**

Under unoptimized conditions, to an oven-dried 10 mL vial, $Pd(OAc)_2$ (0.5 mg, 5 mol %), Ph_3P (1.5 mg, 10 mol %) and aniline **5t** (32.0 mg, 0.050 mmol, 1.0. equiv.) were added. Three vacuum-argon cycles were performed and dry and degassed DMF (0.2 M) was added under argon atmosphere. While stirring the reaction mixture at room temperature, ethyl acrylate (16.2 µL, 0.15 mmol, 3.0 equiv.) and Et_3N (35.6 µL, 0.255 mmol, 5.0 equiv.) were added and the reaction mixture was warmed to 120 ºC and stirred for 16 h. Then, the mixture was diluted with EtOAc and extracted with H_2O three times. The solvent was evaporated under reduced pressure and the residue was purified by column chromatography (gradient elution: 0:100 → 35:65 Et_2O – hexane) to give product **14** (11.5 mg, 35%) as colourless oil.

Data for **14**: $\mathbf{R}_f$ 0.10 (30% Et_2O – hexane). **^{1}H NMR (400 MHz, $CDCl_3$)** δ 7.86 (1H, d, J = 15.7 Hz, 15-H), 7.61 (2H, d, J = 8.3 Hz, Ts), 7.36 (2H, d, J = 8.0 Hz, Ts), 7.33 (1H, s, 7-H), 7.31 – 7.24 (5H, m, Ar), 7.05 – 6.99 (4H, m, Ar), 6.89 (2H, d, J = 8.7 Hz, 12-H), 6.12 (1H, dd, J = 15.5 and 6.3 Hz, 2-H), 5.93 (1H, d, J = 15.7 Hz, 16-H), 5.23 – 5.06 (1H, m, 3-H), 5.01 (1H, d, J = 6.2 Hz, 1-H), 4.80 (1H, d, J = 14.2 Hz, CH_a Bn), 4.74 (1H, d, J = 14.2 Hz, CH_b Bn), 4.29 (2H, q, J = 7.1 Hz, CH_2 OEt), 3.86 (3H, s, OMe), 2.89 (2H, qd, J = 10.6 and 7.0 Hz, 4-H_2), 2.53 (3H, s, Me Ts), 1.38 (3H, t, J = 7.1 Hz, CH_3 OEt). **^{13}C NMR (101 MHz, $CDCl_3$)** δ 166.5 (1C, C=O), 158.6 (1C, C-14), 144.0 (1C, C Ar), 141.4 (1C, C Ar), 141.3 (1C, C-15), 139.6 (1C, C-2), 138.1 (1C, C Ar), 135.9 (1C, C Ar), 135.5 (1C, C Ar), 134.4 (1C, C Ar), 133.2 (1C, C Ar), 130.7 (1C, CH Ar), 129.9 (2C, CH Ts), 129.8 (2C, C-12), 129.7 (1C, CH Ar), 128.7 (2C, CH Ar), 128.6 (2C, CH Ar), 127.90 (2C, CH Ar), 127.85 (1C, CH Ar), 127.2 (1C, CH Ar), 125.93 (1C, q, J = 276.7 Hz, CF_3) 121.4 (1C, C-16), 120.8 (1C, q, J = 3.4 Hz, C-3), 114.2 (2C, C-13), 60.7 (1C, CH_2 OEt), 55.4 (1C, OMe), 54.9 (1C, CH_2 Bn), 48.7 (1C, C-1), 37.4 (1C, q, J = 29.5 Hz, C-4), 21.7 (1C, Me Ts), 14.4 (1C, CH_3 OEt). **^{19}F NMR (376 MHz, $CDCl_3$)** δ –67.3 (3F, CF_3). **HRMS** (ESI): calculated for $C_{37}H_{37}F_3NO_5S$ $[M+H]^+$ requires m/z 664.2339, found 664.2339.

8.4. *ortho*-Benzoylation: synthesis of *tert*-Butyl (*E*)-{2-benzoyl-4-[5,5,5-trifluoro-1-(4-methoxyphenyl)pent-2-en-1-yl]phenyl}carbamate, 15.

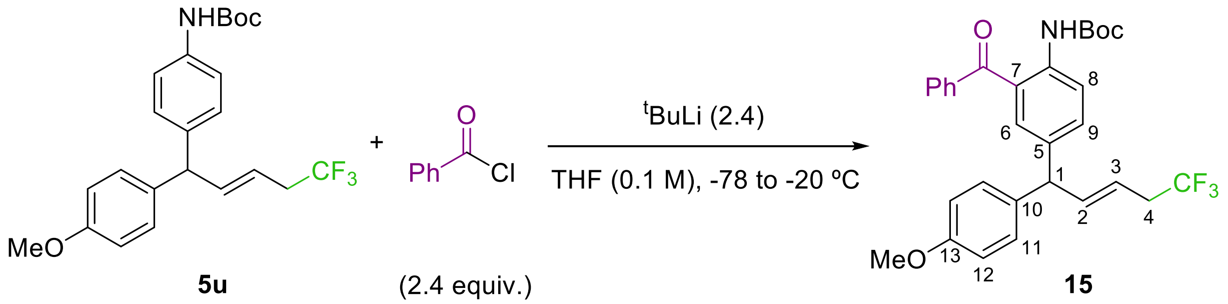

Following an adapted procedure,[8] to an oven-dried 10 mL vial, aniline **5u** (9.4 mg, 0.022 mmol, 1.0 equiv.) was added. Three vacuum-argon cycles were performed and dry THF (0.1 M) was added under argon atmosphere. While stirring the reaction mixture at –78 ºC, ^{t}BuLi (31.3 µL, 0.053 mmol, 2.4 equiv.) was added dropwise, the reaction mixture was stirred for 15 min and stirred at –20 ºC for 2h. Then, benzoyl chloride (6.2 µL, 0.053 mmol, 2.4 equiv.) was added and the reaction mixture allowed to stir at room temperature until total conversion was observed by TLC monitoring. When total conversion was observed, the mixture was diluted with EtOAc and extracted with H_2O three times. The solvent was evaporated under reduced pressure and the residue was purified by column chromatography (gradient elution: 0:100 → 20:80 EtOAc – hexane) to give product **15** (8.6 mg, 74%) as colourless oil.

Data for **15**: $\mathbf{R_{\mathit{f}}}$ 0.40 (30% EtOAc – hexane). **^{1}H NMR (400 MHz, CDCl$_3$)** δ 7.71 (2H, d, *J* = 7.1 Hz, Ph), 7.53 (1H, t, *J* = 7.2 Hz, Ph), 7.44 (2H, t, *J* = 7.4 Hz, Ph), 7.20 (3H, s, Ar), 7.07 (2H, d, *J* = 8.6 Hz, 11-H), 6.85 (2H, d, *J* = 8.7 Hz, 12-H), 6.14 (1H, dd, *J* = 15.4 and 7.5 Hz, 2-H), 5.37 (1H, dtd, *J* = 15.5, 7.2 and 1.3 Hz, 3-H), 4.73 (1H, d, *J* = 7.4 Hz, 1-H), 3.79 (3H, s, OMe), 2.93 – 2.77 (2H, m, 4-H_2), 1.21 (9H, s, ^{t}Bu Boc). **^{13}C NMR (101 MHz, CDCl$_3$)** δ 173.0 (1C, C=O Ph), 158.5 (1C, C-13), 153.5 (1C, C=O Boc), 142.8 (1C, C-5), 140.4 (1C, C-2), 137.5 (1C, C-7), 137.2 (1C, C Ph), 134.6 (1C, C-10), 131.9 (1C, CH Ph), 129.7 (2C, C-11), 129.4 (2C, C Ar and CH Ar), 128.4 (2C, CH Ar), 128.3 (2C, CH Ph), 128.0 (2C, CH Ph), 126.1 (1C, q, *J* = 276.6 Hz, CF_3), 119.8 (1C, q, *J* = 3.7 Hz, C-3), 114.1 (2C, C-12), 83.7 (1C, C Boc), 55.4 (1C, OMe), 52.7 (1C, C-1), 37.4 (1C, q, *J* = 29.7 Hz, C-4), 27.6 (3C, ^{t}Bu Boc). **^{19}F NMR (376 MHz, CDCl$_3$)** δ –66.4 (3F, CF_3). **HRMS** (ESI): calculated for $C_{30}H_{31}F_3NO_4$ $[M+H]^+$ requires m/z 548.2019, found 548.2014.

8.5. Elimination: synthesis (*E*)-*N*-benzyl-*N*-(4-(5,5-difluoro-1-(4-methoxyphenyl)penta-2,4-dien-1-yl)phenyl)-4-methylbenzenesulfonamide, 16.

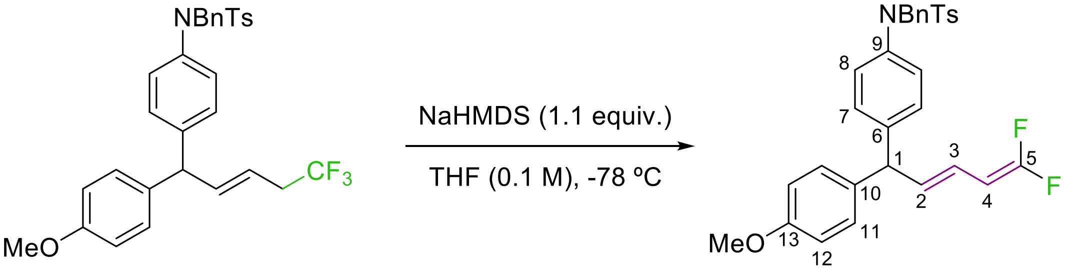

5s **16**

Following an adapted reported procedure[9] under unoptimized conditions, to an oven-dried 10 mL vial, aniline **5s** (17.0 mg, 0.030 mmol, 1 equiv.) was added and three vacuum-argon cycles were performed. THF (0.1 M), was added and the reaction mixture stirred at -78 ºC. After 5 minutes, NaHMDS (33 µL, 0.033 mmol, 1.1 equiv.) in THF (1 M) was added reaction and the reaction was stirred at -78 ºC for 1 h and monitored by TLC until completion. Then, the reaction mixture was quenched at -78 ºC with aqueous NH_4Cl and let it warm to room temperature. The mixture was diluted with H_2O and extracted with ethyl acetate. The combined organic phase was dried over anhydrous Na_2SO_4 and the solvent evaporated under reduced pressure. The residue was purified by column chromatography (gradient elution: 0:100 → 10:90 Et_2O – hexane) to give the product **16** (7.3 mg, 45%) as colourless oil.

Data for **16**: **R_f** 0.25 (30% Et_2O – hexane). **^{1}H NMR (400 MHz, $CDCl_3$)** δ 7.54 (2H, d, *J* = 8.0 Hz, Ts), 7.31 – 7.25 (2H, m, Ar), 7.24 – 7.18 (5H, m, Ar), 6.99 (2H, d, *J* = 8.7 Hz, 11-H), 6.97 (2H, d, *J* = 8.3 Hz, 7-H), 6.90 (2H, d, *J* = 8.2 Hz, 8-H), 6.83 (2H, d, *J* = 8.5 Hz, 12-H), 5.95 (1H, dd, *J* = 15.4 and 7.4 Hz, 2-H), 5.75 (1H, dd, *J* = 15.4 and 10.8 Hz, 3-H), 4.98 (1H, dd, *J* = 24.4 and 10.5 Hz, 4-H), 4.69 (2H, s, CH_2 Bn), 4.63 (1H, d, *J* = 7.4 Hz, 1-H), 3.79 (3H, s, OMe), 2.44 (3H, s, Me Ts). **^{13}C NMR (101 MHz, $CDCl_3$)** δ 158.4 (1C, C-13), 154.9 (1C, dd, *J* = 296.8 and 296.2 Hz, C-5) 143.6 (1C, C Ar), 143.0 (1C, C Ar), 137.5 (1C, C Ar), 136.2 (1C, C Ar), 135.8 (1C, C Ar), 135.0 (1C, dd, *J* = 11.1 and 2.9 Hz, C-2), 134.8 (1C, C Ar), , 129.63 (2C, CH Ar), 129.61 (2C, CH Ar), 129.04 (2C, CH Ar), 128.96 (2C, CH Ar), 128.6 (2C, CH Ar), 128.5 (2C, CH Ar), 127.9 (2C, CH Ar), 127.7 (1C, CH Ar), 120.5 (1C, d, *J* = 4.7 Hz, C-3), 114.0 (2C, C-12), 81.9 (1H, dd, *J* = 27.1 and 16.9 Hz, C-4), 55.4 (1C, OMe), 54.9 (1C, CH_2 Bn), 52.8 (1C, C-1), 21.7 (1C, Me Ts). **^{19}F NMR (376 MHz, $CDCl_3$)** δ –86.2 (1F, d, *J* = 30.0 Hz, 5-F_{trans}), –88.3 (1F, d, *J* = 31.3 Hz, 5-F_{cis}). **HRMS** (ESI): calculated for $C_{32}H_{29}F_2NNaO_3S$ $[M+Na]^+$ requires *m/z* 568.1728, found 568.1720.

9. Mechanistic experiments

· Hofmann-Martius rearrangement

Intermediate **S-4d** was synthesised according to the following procedure:

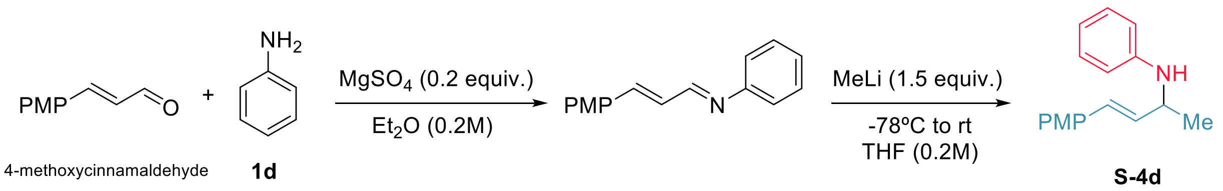

Imine formation: To a solution containing aniline (250 mg, 1.45 mmol, 1.0 equiv.) and magnesium sulphate (34.9 mg, 0.29 mmol, 0.20 equiv.) in dry diethyl ether (0.2M), *trans*-4-methoxycinnamaldehyde was added (253.2 mg, 1.45 mmol, 1.0 equiv.). The reaction mixture was stirred at 20 ºC and monitored by TLC until complete conversion. After total conversion, the reaction mixture was filtered through a paper filter and evaporated under reduced pressure to dryness. The imine was used without further purification for the next step.

Methylation: To an oven-dry round bottom flask, previous imine (1.45 mmol) was added in dry THF (0.2M). The reaction mixture was stirred at –78 ºC. After 5 minutes, MeLi (1.5 equiv.) was added dropwise, and the reaction mixture was stirred overnight at 20 ºC. The reaction mixture was monitored by TLC until complete conversion. The reaction mixture was quenched with saturated aqueous NH_4Cl at 0 ºC and extracted with ethyl acetate. The organic phase was dry over Na_2SO_4 and purified by chromatographic column to yield product **S-4d** (332.2 mg, 79 %) as an orange solid.

Hofmann-Martius rearrangement: From aniline **S-4d** (50.7 mg, 0.2 mmol) in 1 mL of HFIP, following the general procedure, aniline **4a** (50.2 mg, 99%) was obtained without further purification.

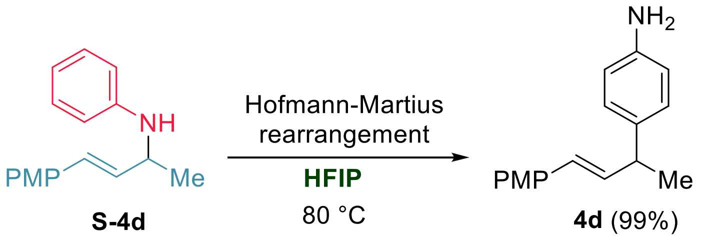

· Deuteration experiments

(*E*)-4-[4-(4-Methoxyphenyl)but-3-en-2-yl-1-d]-*N*-methylbenzen-2,6-d2-amine, 4b-d$_3$.

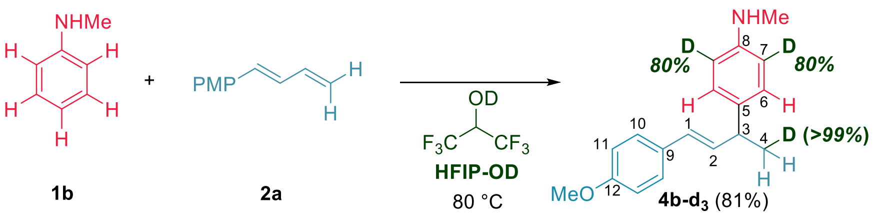

From aniline **1b** (5.4 mg, 0.05 mmol) and diene **2a** (12.0 mg, 0.075 mmol), in 0.25 mL of HFIP-OD, following the general procedure, aniline **4b-d3** was obtained. Chromatographic purification (0:100 → 20:80 Et_2O – hexane) gave aniline **4b-d3** (11.0 mg, 81%) as a colourless oil.

Data for **4b-d3**: $\mathbf{R_f}$ 0.15 (30% Et_2O – hexane). **^{1}H NMR (400 MHz, CDCl$_3$)** δ 7.28 (2H, d, *J* = 8.7 Hz, 10-H), 7.12 – 7.06 (2H, m, Ar), 6.82 (2H, d, *J* = 8.8 Hz, 11-H), 6.59 (0.43H, d, *J* = 8.8 Hz, Ar), 6.33 (1H, d, *J* = 15.9 Hz, 1-H), 6.22 (1H, dd, *J* = 15.9 and 6.5 Hz, 2-H), 3.79 (3H, s, OMe), 3.52 (1H, q, *J* = 6.8 Hz, 3-H), 2.83 (3H, s, NMe), 1.40 (2H, t, *J* = 6.9 Hz, 4-H_2).**^{13}C NMR (101 MHz, CDCl$_3$)** 158.9 (1C, C Ar), 145.75 (1C, C Ar), 136.8 (1C, C Ar), 133.8 (1C, C-2), 130.7 (1C, C Ar), 128.2 (2C, C-6), 127.6 (2C, C-10), 127.3 (1C, C-1), 114.0 (4C, C-11 and C-7), 55.4 (1C, OMe), 41.8 (1C, C-3), 32.2 (1C, NMe), 21.2 (1C, t, *J* = 19.5 Hz, C-4). **^{2}H (77 MHz, CHCl$_3$)** δ 6.6 (2D, s, 7-D), 1.4 (1D, s, 4-D).

(*E*)-4-[4-(4-Methoxyphenyl)but-3-en-2-yl]benzen-2,3,5,6-d4-amine, 4d-d$_4$.

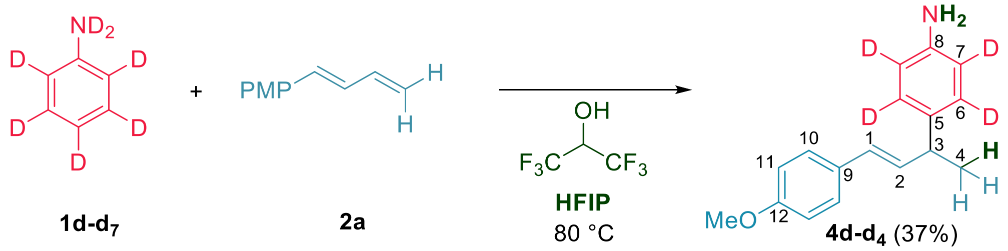

From aniline **1d-d$_7$** (22.0 mg, 0.22 mmol) and diene **2a** (52.9 mg, 0.33 mmol), in 1.1 mL of HFIP, following the general procedure and after the kinetic experiment, aniline **4d-d$_4$** was obtained. Chromatographic purification (0:100 → 50:50 Et_2O – hexane) gave aniline **4d-d$_4$** (21.1 mg, 37%) as a colourless oil.

Data for **4d-d$_4$**: $\mathbf{R_f}$ 0.1 (50% Et_2O – hexane). **^{1}H NMR (400 MHz, CDCl$_3$)** 7.29 (2H, d, *J* = 8.7 Hz, 6-H), 6.84 (2H, d, *J* = 8.8 Hz, 7-H), 6.33 (1H, d, *J* = 15.9 Hz, 1-H), 6.22 (1H, dd, *J* = 15.8 and 6.6 Hz, 2-H), 3.80 (3H, s, OMe), 3.59 (1H, s, NH), 3.57 – 3.47 (1H, m, 3-H), 1.42 (3H, d, *J* = 7.0 Hz, 4-H_2).**^{13}C NMR (101 MHz, CDCl$_3$)** δ 158.8 (1C, C Ar), 144.5 (1C, C Ar), 136.0 (1C, C Ar), 134.0 (1C, C-2), 130.7 (1C, C Ar), 127.81 (2C, t, *J* = 24.0 Hz, C-6), 127.5 (1C, C-1), 127.3 (2C, C-10), 115.1 (2C, t, *J* = 25.2 Hz, C-7), 114.0 (2C, C-11), 55.4 (1C, OMe), 41.7 (1C, C-3), 21.5 (1C, C-4). **^{2}H (77 MHz, CHCl$_3$)** δ 7.1 (2D, s, 6-D), 6.7 (2D, s, 7-D).

· Kinetic experiments and Kinetic Isotope Effect (KIE)

Kinetic experiments were performed one round and carrying out the reaction following the general protocol. Aliquots were taken at 0, 10, 30, 50, 80, 120, 180 and 220 minutes, diluted with a solution of $CDCl_3$ containing benzoic acid as internal standard and ^1H NMR experiments were recorded. The integrals were measured over the most clean and isolated signal of each component of the mixture. Concentration of each aliquot was corrected to the concentration in the reaction mixture.

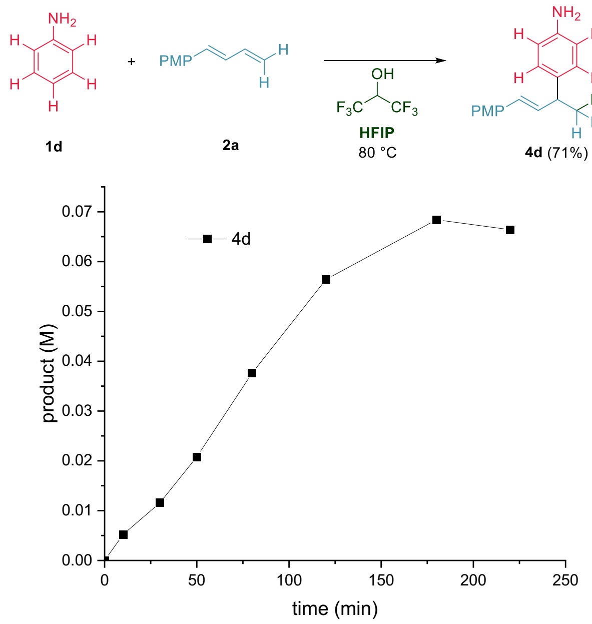

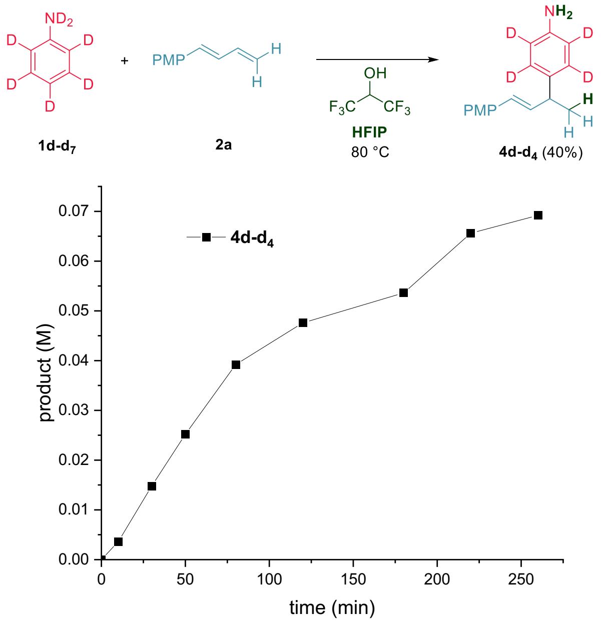

Figure S1. Kinetic experiment using **1d** in HFIP-OH. **Figure S2.** Kinetic experiment using **1d-d₇** in HFIP-OH.

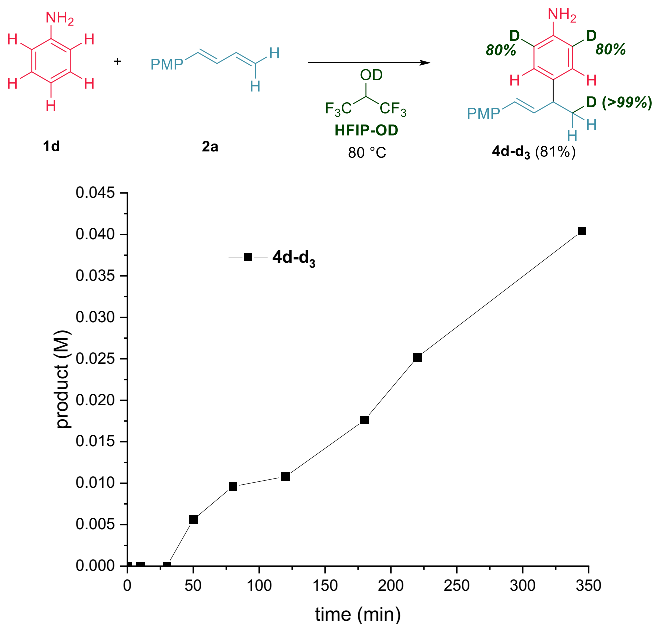

Figure S3. Kinetic experiment using **1d** in **HFIP-OD**.

K_{1d}/K_{1d-d7}

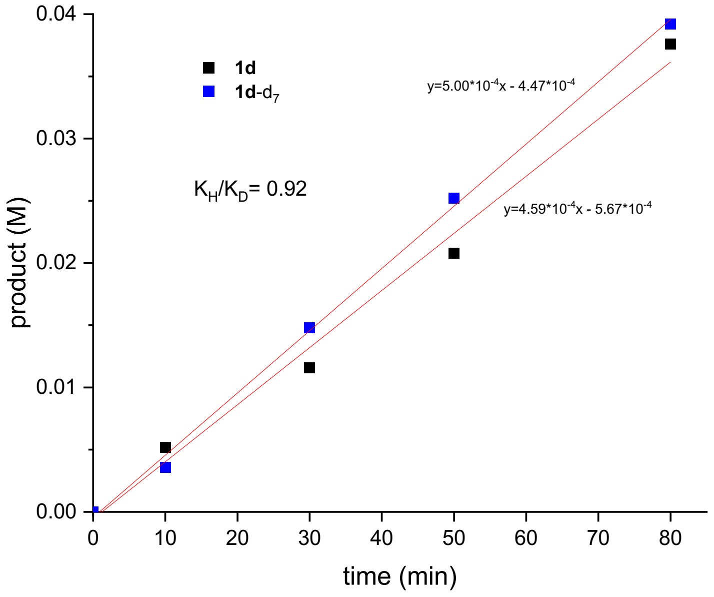

Figure S4. Kinetic isotope effect experiment for **1d** and **1d-d$_7$**.

Comparing deuterated aniline (**1d-*d$_7$***) vs. non-deuterated aniline (**1d**) shows an inverse secondary KIE (=0.92). This is in agreement with similar results for aryl nitration via electrophilic aromatic substitution, where the σ-/π-complex formation or the elecrophile addition (Wheland intermediate with de-aromatization) is the RDS.

The KIE for a Friedel-Crafts reaction might vary from a small primary KIE value to a secondary inverse KIE. Those values are usually explained depending on which is the rate-determining step (RDS), that is different for acylation, alkylation or nitration. An inverse secondary KIE is observed for alkylation and nitrations, where it has been proposed that the rate-determing step is not the rearomatization of the Wheland intermediate, but the formation of relative stable σ-/π-complex or the Wheland intermediate. [10]-[13]

$K_{HFIP}/K_{HFIP\text{-}OD}$

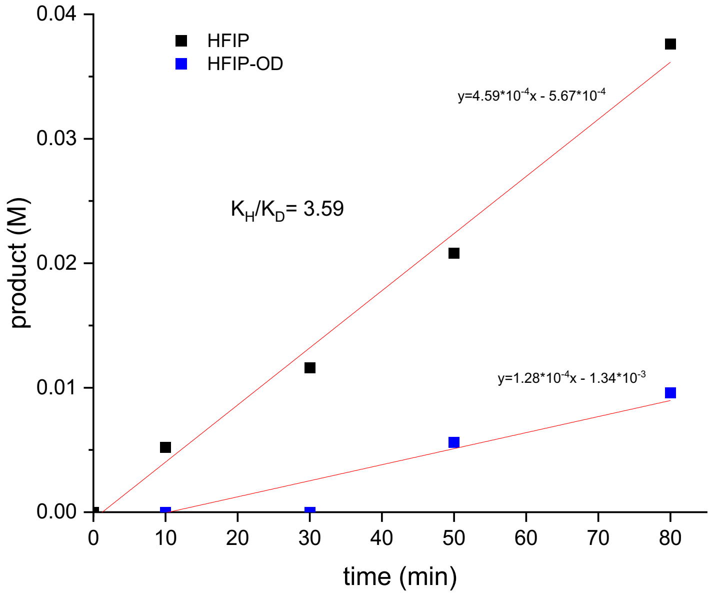

Figure S5. Kinetic isotope effect experiment for **HFIP** and **HFIP-OD**.

Comparing deuterated solvent (HFIP-OD) vs. non-deuterated solvent (HFIP-OH) shows a strong KIE (= 3.59). This result might indicate that there is probably a rapid pre-equilibrium between aniline-HFIP, followed by the RDS (σ-/π-complex formation or the elecrophile addition). But we have to be extremely careful, because the medium has changed and therefore the properties are different. [14]

10. Computational details

All the calculations reported in this paper were performed with the Gaussian 09 suite of programs.[15] Electron correlation was partially taken into account using the hybrid functional usually denoted as B3LYP[16] in conjunction with the D3 dispersion correction suggested by Grimme et al.[17] using the standard double-ζ quality def2-SVP[18] basis set for all atoms including solvent effects (solvent = HFIP, e = 16.7) with the Polarization Continuum Model (PCM) method.[19] Geometries were fully optimized in solution without any geometry or symmetry constraints. Reactants, intermediates, and products were characterized by frequency calculations,[20] and have positive definite Hessian matrices. Transition structures (TS's) show only one negative eigenvalue in their diagonalized force constant matrices, and their associated eigenvectors were confirmed to correspond to the motion along the reaction coordinate under consideration using the Intrinsic Reaction Coordinate (IRC) method.[21] Energy refinements were carried out by means of single-point calculations at the accurate M06-2X[22] level using the much larger triple-ζ basis set def2-TZVPP.[18] This level is denoted PCM(HFIP)-M06-2X/def2-TZVPP//PCM(HFIP)-B3LYP–D3/def2-SVP.

Activation Strain Model of Reactivity

Within the ASM method,[23] also known as the distortion/interaction model,[23c] the potential energy surface $\Delta E(\zeta)$ is decomposed along the reaction coordinate, ζ, into two contributions, namely the strain $\Delta E_{strain}(\zeta)$ associated with the deformation (or distortion) required by the individual reactants during the process and the interaction $\Delta E_{int}(\zeta)$ between these increasingly deformed reactants:

$$\Delta E(\zeta) = \Delta E_{strain}(\zeta) + \Delta E_{int}(\zeta)$$

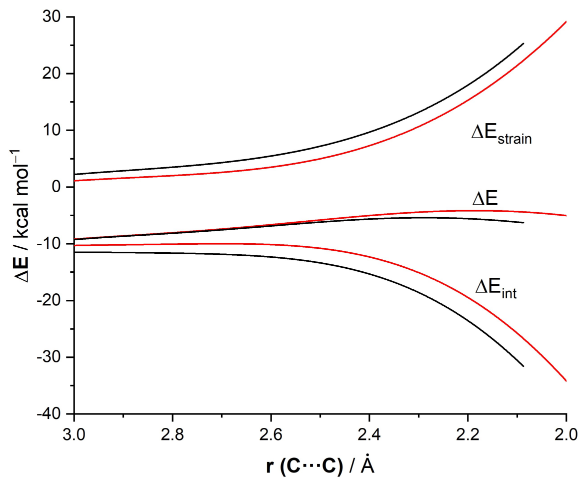

Figure S6. Comparative activation strain analyses for both pathways involved in the **1b** + **AC-b** reaction (black lines: 1,2-addition; red lines: 1,4-addition) projected onto the C···C bond-forming distance. All data have been computed at the PCM(HFIP)-M06-2X/def2-TZVPP//PCM(HFIP)-B3LYP-D3/def2-SVP level.

Cartesian coordinates (in Å) and total energies (in a.u.) of all the stationary points discussed in the text. All calculations have been performed at the PCM(HFIP)-M06-2X/def2-TZVPP//PCM(HFIP)-B3LYP–D3/def2-SVP level.

1b, E = -326.890695

C	2.847580000	0.362186000	0.058555000
H	2.838786000	1.098942000	-0.763881000
H	3.815061000	-0.157195000	0.027489000
H	2.790875000	0.927707000	1.009937000
C	0.450474000	-0.280308000	-0.034212000
C	0.004078000	1.062533000	-0.029742000
H	0.724850000	1.881013000	-0.045808000
C	-1.363195000	1.355360000	-0.006140000
H	-1.678641000	2.402878000	-0.004990000
C	-2.322740000	0.338983000	0.020959000
C	-1.887347000	-0.994523000	0.024081000
H	-2.618133000	-1.808096000	0.048256000
C	-0.529798000	-1.304073000	-0.002488000
H	-0.207262000	-2.349670000	-0.001645000
N	1.786709000	-0.606002000	-0.085876000
H	2.021789000	-1.571891000	0.104057000
H	-3.388605000	0.577375000	0.041633000

AC-a (R = H), E = -541.27556

C	1.202636000	0.691179000	0.178123000
H	1.406225000	1.767851000	0.186044000
C	-0.148394000	0.316842000	0.107941000
C	-1.143473000	1.343198000	0.048566000
H	-0.824896000	2.388232000	0.061583000
C	-2.487316000	1.048326000	-0.024043000
H	-3.218542000	1.854104000	-0.066648000
C	-2.896911000	-0.310854000	-0.040664000
C	-1.924923000	-1.351660000	0.018493000
H	-2.279253000	-2.383251000	0.003594000
C	-0.590437000	-1.048305000	0.090161000
H	0.135893000	-1.860531000	0.133138000
C	-5.228822000	0.236944000	-0.177288000
H	-6.146000000	-0.359356000	-0.237538000
H	-5.135927000	0.868159000	-1.074225000
H	-5.252277000	0.864793000	0.726604000
C	2.332413000	-0.159085000	0.238587000
H	2.204731000	-1.244735000	0.237832000
C	3.595127000	0.362588000	0.297477000
H	3.707015000	1.454891000	0.289273000
C	4.849715000	-0.426229000	0.361931000
H	4.631195000	-1.505271000	0.352752000
C	5.839110000	-0.052932000	-0.758173000
H	5.418560000	-0.284430000	-1.748689000
H	6.772861000	-0.621925000	-0.638745000
H	6.086517000	1.019991000	-0.732068000

H	5.324454000	-0.197824000	1.336606000
O	-4.152864000	-0.707597000	-0.109522000

AC-b (R = CF3), E = -878.367666

C	0.322510000	-0.606644000	-0.392890000
H	0.113931000	-1.646033000	-0.669413000
C	1.666344000	-0.268082000	-0.211888000
C	2.661199000	-1.284672000	-0.395931000
H	2.340610000	-2.290756000	-0.676136000
C	4.001308000	-1.025888000	-0.227771000
H	4.731295000	-1.820061000	-0.375141000
C	4.411880000	0.284790000	0.140141000
C	3.442009000	1.315479000	0.326645000
H	3.799087000	2.307393000	0.606728000
C	2.110532000	1.049211000	0.156310000
H	1.385894000	1.850298000	0.304931000
C	6.742788000	-0.291498000	0.181448000
H	7.655306000	0.272914000	0.402253000
H	6.781914000	-0.675446000	-0.849122000
H	6.636410000	-1.123005000	0.894468000
C	-0.809192000	0.243915000	-0.252990000
H	-0.674751000	1.294759000	0.015856000
C	-2.065388000	-0.240884000	-0.444995000
H	-2.198501000	-1.295693000	-0.713030000
C	-3.310223000	0.584166000	-0.368904000
H	-3.098349000	1.546214000	0.121583000
C	-3.877806000	0.836549000	-1.781872000
H	-3.148312000	1.393211000	-2.384986000
H	-4.800959000	1.428717000	-1.707653000
H	-4.106843000	-0.112500000	-2.288044000
C	-4.349279000	-0.113762000	0.505908000
F	-4.696519000	-1.314121000	0.002814000
F	-3.880007000	-0.322742000	1.748516000
F	-5.467861000	0.618647000	0.611714000
O	5.662833000	0.642233000	0.331382000

RC-12 (R = H), E = -868.181757

C	-1.569593000	2.463398000	-0.052818000
H	-1.414479000	3.411512000	0.493632000
H	-1.851904000	1.689486000	0.675520000
H	-2.414184000	2.591843000	-0.741383000
C	0.829450000	1.830075000	-0.281138000
C	1.021025000	1.662576000	1.117761000
H	0.175953000	1.761880000	1.799096000
C	2.291944000	1.397403000	1.630046000
H	2.414578000	1.279623000	2.709602000
C	3.393908000	1.262914000	0.783005000
C	3.213132000	1.436031000	-0.604246000
H	4.068789000	1.349084000	-1.278597000
C	1.961283000	1.709905000	-1.130989000

H	1.833670000	1.837199000	-2.209191000
C	0.460002000	-1.220335000	0.608084000
H	0.683615000	-1.290192000	1.677009000
C	-0.904278000	-1.030022000	0.270813000
C	-1.873715000	-1.034478000	1.312560000
H	-1.542551000	-1.184902000	2.342862000
C	-3.219470000	-0.852861000	1.056811000
H	-3.931928000	-0.866505000	1.880179000
C	-3.649533000	-0.642916000	-0.277022000
C	-2.702233000	-0.633146000	-1.329455000
H	-3.063610000	-0.462573000	-2.344620000
C	-1.362548000	-0.817666000	-1.064458000
H	-0.651305000	-0.793690000	-1.890004000
C	-5.954690000	-0.430817000	0.345884000
H	-6.886487000	-0.251840000	-0.203380000
H	-5.798542000	0.378316000	1.077367000
H	-6.017088000	-1.398476000	0.868976000
C	1.539423000	-1.423862000	-0.270663000
H	1.375415000	-1.393754000	-1.350579000
C	2.804296000	-1.671194000	0.200000000
H	2.960569000	-1.694873000	1.284718000
C	3.960972000	-2.049900000	-0.657710000
H	3.789035000	-1.719268000	-1.695092000
C	5.334480000	-1.604960000	-0.147884000
H	5.445651000	-0.512654000	-0.202727000
H	6.133041000	-2.057539000	-0.753998000
H	5.489028000	-1.910179000	0.899366000
H	3.944345000	-3.159621000	-0.692731000
N	-0.401532000	2.074591000	-0.815637000
O	-4.916039000	-0.443497000	-0.633660000
H	-0.430870000	2.259397000	-1.811269000
H	4.385176000	1.062698000	1.192491000

RC-12 (R = CF3), E = -1205.273478

C	-3.006528000	2.297402000	-0.556691000
H	-3.163707000	3.345563000	-0.244691000
H	-3.131217000	1.652758000	0.324564000
H	-3.787980000	2.026401000	-1.277540000
C	-0.514519000	2.356589000	-0.579072000
C	-0.402445000	2.540211000	0.827471000
H	-1.293703000	2.511450000	1.454042000
C	0.840243000	2.795148000	1.408822000
H	0.900250000	2.943901000	2.489664000
C	1.999000000	2.843177000	0.631443000
C	1.896520000	2.664827000	-0.763814000
H	2.794065000	2.717821000	-1.385137000
C	0.671486000	2.418942000	-1.360801000
H	0.604103000	2.279269000	-2.442724000
C	-0.126961000	-0.387369000	0.946095000
H	0.014073000	-0.178611000	2.010530000
C	-1.444392000	-0.689020000	0.536719000

C	-2.468966000	-0.768473000	1.525436000
H	-2.208053000	-0.593008000	2.571709000
C	-3.776297000	-1.060306000	1.195516000
H	-4.531670000	-1.117307000	1.977843000
C	-4.117330000	-1.276521000	-0.165457000
C	-3.116651000	-1.199815000	-1.165770000
H	-3.412804000	-1.365752000	-2.202476000
C	-1.814221000	-0.909568000	-0.826991000
H	-1.061958000	-0.850430000	-1.613162000
C	-6.433543000	-1.660360000	0.324778000
H	-7.316713000	-1.891411000	-0.281981000
H	-6.592605000	-0.710809000	0.859698000
H	-6.260856000	-2.472869000	1.048179000
C	1.044203000	-0.430512000	0.153301000
H	0.966648000	-0.665688000	-0.910010000
C	2.276358000	-0.199207000	0.692676000
H	2.359689000	0.022378000	1.760983000
C	3.560593000	-0.338195000	-0.081851000
H	3.403813000	0.046407000	-1.103049000
C	4.744037000	0.377013000	0.576668000
H	4.537096000	1.451071000	0.660275000
H	5.657243000	0.243957000	-0.018577000
H	4.922760000	-0.022679000	1.586156000
C	3.888028000	-1.822280000	-0.281281000
F	4.058110000	-2.456628000	0.893449000
F	2.910483000	-2.465536000	-0.946126000
F	5.017867000	-1.978595000	-0.990616000
N	-1.711772000	2.103691000	-1.179228000
O	-5.343248000	-1.553167000	-0.593045000
H	-1.703474000	2.063771000	-2.191920000
H	2.963552000	3.060047000	1.093023000

TS-12 (R = H), E = -868.176272 (i = -297 cm-1)

C	-1.453738000	2.545497000	0.380623000
H	-1.249971000	3.447247000	0.980308000
H	-1.616119000	1.693585000	1.056184000
H	-2.372217000	2.705317000	-0.195953000
C	0.872587000	1.907047000	-0.217306000
C	1.273817000	1.742872000	1.154175000
H	0.591744000	2.023220000	1.956252000
C	2.514075000	1.238977000	1.443752000
H	2.813370000	1.106186000	2.486177000
C	3.383601000	0.771759000	0.406110000
C	3.073412000	1.174687000	-0.942077000
H	3.803045000	0.992246000	-1.734234000
C	1.845639000	1.684619000	-1.252782000
H	1.578130000	1.915711000	-2.286380000
C	0.433626000	-1.152028000	0.658164000
H	0.625447000	-1.142868000	1.736468000
C	-0.962328000	-1.026945000	0.280829000
C	-1.953360000	-1.080142000	1.282897000

H	-1.653539000	-1.256589000	2.319208000
C	-3.304765000	-0.906226000	0.994461000
H	-4.034244000	-0.955095000	1.802059000
C	-3.704543000	-0.661741000	-0.333391000
C	-2.727395000	-0.606698000	-1.350797000
H	-3.056292000	-0.408868000	-2.372703000
C	-1.387654000	-0.784643000	-1.049399000
H	-0.653837000	-0.714849000	-1.854348000
C	-6.022617000	-0.491547000	0.236958000
H	-6.952892000	-0.304037000	-0.313757000
H	-5.889251000	0.293053000	1.001229000
H	-6.087202000	-1.474537000	0.733497000
C	1.514390000	-1.275332000	-0.167995000
H	1.371702000	-1.314446000	-1.251613000
C	2.869304000	-1.248705000	0.307347000
H	2.996264000	-1.413354000	1.383244000
C	3.936994000	-1.892141000	-0.550166000
H	3.816170000	-1.574605000	-1.598919000
C	5.385862000	-1.729668000	-0.093612000
H	5.764111000	-0.710253000	-0.264906000
H	6.040604000	-2.415754000	-0.651392000
H	5.494411000	-1.957702000	0.979144000
H	3.681704000	-2.969359000	-0.539389000
N	-0.368440000	2.271724000	-0.547479000
O	-4.976297000	-0.463029000	-0.724398000
H	-0.572242000	2.376159000	-1.536477000
H	4.429110000	0.603895000	0.667068000

TS-12 (R = CF3), E = -1205.267913 (i = 268 cm-1)

C	-2.897868000	2.493287000	0.092529000
H	-3.018891000	3.486867000	0.553600000
H	-2.831778000	1.735147000	0.884463000
H	-3.782154000	2.275033000	-0.517440000
C	-0.452707000	2.487663000	-0.362153000
C	-0.103003000	2.552744000	1.033286000
H	-0.883672000	2.688414000	1.781155000
C	1.209734000	2.457690000	1.409458000
H	1.474400000	2.497420000	2.468570000
C	2.234253000	2.191898000	0.446226000
C	1.904588000	2.385709000	-0.942686000
H	2.702437000	2.373608000	-1.688796000
C	0.602253000	2.484091000	-1.340109000
H	0.340805000	2.551036000	-2.398602000
C	-0.096851000	-0.462920000	0.802384000
H	0.053205000	-0.332905000	1.879225000
C	-1.454664000	-0.755896000	0.397876000
C	-2.433338000	-0.985099000	1.389544000
H	-2.136850000	-0.977018000	2.441601000
C	-3.768227000	-1.210558000	1.068696000
H	-4.488966000	-1.380337000	1.867602000

C	-4.167093000	-1.207533000	-0.283010000
C	-3.201598000	-0.986817000	-1.289965000
H	-3.531650000	-0.984172000	-2.330376000
C	-1.876938000	-0.765645000	-0.956633000
H	-1.157754000	-0.580810000	-1.756461000
C	-6.468424000	-1.609546000	0.239083000
H	-7.391170000	-1.731809000	-0.341532000
H	-6.577670000	-0.746520000	0.917527000
H	-6.290642000	-2.520805000	0.834457000
C	1.004855000	-0.343313000	-0.004131000
H	0.907123000	-0.501144000	-1.080589000
C	2.281518000	0.071804000	0.481012000
H	2.426532000	0.024300000	1.564918000
C	3.522239000	-0.317989000	-0.317120000
H	3.320621000	-0.182525000	-1.391615000
C	4.814110000	0.416453000	0.063778000
H	4.800208000	1.451987000	-0.297919000
H	5.681696000	-0.075207000	-0.395101000
H	4.955923000	0.423543000	1.154780000
C	3.742682000	-1.830370000	-0.154675000
F	3.954874000	-2.161173000	1.133800000
F	2.695035000	-2.553683000	-0.586812000
F	4.813049000	-2.243647000	-0.856161000
N	-1.723895000	2.449199000	-0.765022000
O	-5.427549000	-1.396176000	-0.706240000
H	-1.889326000	2.422535000	-1.766464000
H	3.265482000	2.340978000	0.764272000

INT-12 (R = H), E = -868.192174

C	1.868012000	4.456841000	0.422828000
H	0.882536000	4.167885000	0.816810000
H	1.766776000	5.395334000	-0.132963000
H	2.561177000	4.613301000	1.262539000
C	2.623269000	2.175632000	-0.180290000
C	2.395659000	1.664427000	1.154005000
H	2.042421000	2.334185000	1.937051000
C	2.614044000	0.356706000	1.419394000
H	2.425468000	-0.023570000	2.427129000
C	3.073747000	-0.626480000	0.404747000
C	3.356484000	-0.020220000	-0.923502000
H	3.750899000	-0.671672000	-1.706552000
C	3.137814000	1.283221000	-1.200593000
H	3.346725000	1.689952000	-2.192816000
C	-0.385326000	-1.336453000	0.635724000
H	-0.284730000	-1.671967000	1.675735000
C	-1.732632000	-0.881737000	0.264899000
C	-2.754632000	-0.894672000	1.230078000
H	-2.529167000	-1.247384000	2.240551000
C	-4.056012000	-0.470567000	0.943402000
H	-4.811602000	-0.500738000	1.728117000
C	-4.367636000	-0.014868000	-0.346941000

C	-3.356447000	0.005256000	-1.329458000
H	-3.613656000	0.360979000	-2.329500000
C	-2.070373000	-0.418776000	-1.027956000
H	-1.313163000	-0.390890000	-1.814586000
C	-6.656947000	0.417791000	0.192875000
H	-7.536976000	0.799326000	-0.341014000
H	-6.446094000	1.074830000	1.054964000
H	-6.873474000	-0.599611000	0.563346000
C	0.710840000	-1.377804000	-0.141754000
H	0.661304000	-1.047270000	-1.185884000
C	2.062869000	-1.847332000	0.324059000
H	1.976830000	-2.220447000	1.358545000
C	2.588784000	-3.004519000	-0.550000000
H	2.594710000	-2.699098000	-1.610585000
C	3.960158000	-3.550580000	-0.151596000
H	4.770944000	-2.828539000	-0.341546000
H	4.197971000	-4.458764000	-0.726638000
H	3.988782000	-3.814197000	0.918752000
H	1.839082000	-3.810050000	-0.480019000
N	2.380271000	3.434788000	-0.484326000
O	-5.588784000	0.418332000	-0.738869000
H	2.560982000	3.724910000	-1.443310000
H	4.020589000	-1.061869000	0.783585000

INT-12 (R = CF3), E = -1205.286803

C	-0.415856000	5.422955000	0.249347000
H	-1.250728000	4.825596000	0.644113000
H	-0.820235000	6.247868000	-0.347060000
H	0.161171000	5.838493000	1.088495000
C	1.100529000	3.527894000	-0.254060000
C	1.047525000	3.019699000	1.100141000
H	0.466015000	3.547434000	1.854938000
C	1.711219000	1.886865000	1.418935000
H	1.654868000	1.501913000	2.440730000
C	2.528142000	1.105006000	0.450897000
C	2.578386000	1.710612000	-0.909087000
H	3.186594000	1.213890000	-1.668609000
C	1.908063000	2.833918000	-1.239431000
H	1.962348000	3.244858000	-2.250147000
C	-0.423563000	-0.673238000	0.724294000
H	-0.244300000	-0.818672000	1.796951000
C	-1.836919000	-0.711740000	0.327137000
C	-2.822943000	-0.915719000	1.308014000
H	-2.519036000	-1.043968000	2.350783000
C	-4.185313000	-0.962043000	0.996479000
H	-4.909007000	-1.124034000	1.794906000
C	-4.596906000	-0.801415000	-0.335728000
C	-3.622654000	-0.595541000	-1.334277000
H	-3.957873000	-0.473514000	-2.366485000
C	-2.275150000	-0.552006000	-1.007835000
H	-1.548885000	-0.392144000	-1.807740000

C	-6.917827000	-1.040684000	0.190477000
H	-7.861021000	-1.027897000	-0.370980000
H	-6.942460000	-0.243257000	0.953844000
H	-6.812674000	-2.016677000	0.695871000
C	0.644373000	-0.484451000	-0.068739000
H	0.517010000	-0.338247000	-1.146557000
C	2.062551000	-0.406112000	0.435453000
H	2.091384000	-0.734838000	1.486480000
C	3.049274000	-1.317206000	-0.347657000
H	2.939194000	-1.140083000	-1.430099000
C	4.517610000	-1.122028000	0.052950000
H	4.888271000	-0.134973000	-0.254110000
H	5.153736000	-1.872125000	-0.434724000
H	4.642587000	-1.219038000	1.142553000
C	2.666085000	-2.785515000	-0.161304000
F	2.624819000	-3.128147000	1.143385000
F	1.465471000	-3.081747000	-0.689302000
F	3.555995000	-3.604514000	-0.755767000
N	0.438565000	4.608956000	-0.610007000
O	-5.883286000	-0.827795000	-0.755026000
H	0.523571000	4.906181000	-1.580218000
H	3.559480000	1.107089000	0.856430000

RC-14 (R = H), E = -868.181817

C	-3.496096000	1.966554000	0.048628000
H	-3.467283000	1.451802000	1.022017000
H	-4.425422000	1.680394000	-0.460614000
H	-3.524601000	3.054217000	0.244773000
C	-1.065160000	1.798387000	-0.431136000
C	-0.690790000	2.163001000	0.887751000
H	-1.453994000	2.336658000	1.646008000
C	0.654842000	2.280423000	1.228007000
H	0.922696000	2.544492000	2.254237000
C	1.660760000	2.048054000	0.281918000
C	1.297322000	1.743046000	-1.039808000
H	2.071447000	1.579004000	-1.793505000
C	-0.037557000	1.612126000	-1.395415000
H	-0.311659000	1.350904000	-2.420382000
C	1.587255000	-0.969687000	0.813278000
H	1.771016000	-0.703792000	1.859078000
C	0.245826000	-1.125581000	0.436808000
C	-0.777191000	-0.942198000	1.419624000
H	-0.484674000	-0.672785000	2.436343000
C	-2.113119000	-1.118144000	1.125984000
H	-2.859398000	-0.987478000	1.907880000
C	-2.494110000	-1.436073000	-0.200748000
C	-1.500655000	-1.601708000	-1.204203000
H	-1.830253000	-1.846129000	-2.214920000
C	-0.172488000	-1.448292000	-0.897744000
H	0.570666000	-1.580621000	-1.683856000
C	-4.826630000	-1.579884000	0.342180000

H	-5.733304000	-1.799837000	-0.233030000
H	-4.913707000	-0.580960000	0.794669000
H	-4.695410000	-2.337807000	1.130303000
C	2.740195000	-1.184964000	0.004887000
H	2.616580000	-1.450067000	-1.048729000
C	3.997520000	-1.067271000	0.511627000
H	4.109945000	-0.785364000	1.566938000
C	5.258250000	-1.244972000	-0.259559000
H	5.037865000	-1.582309000	-1.284709000
C	6.100663000	0.044563000	-0.284765000
H	5.559034000	0.858736000	-0.791211000
H	7.045336000	-0.127409000	-0.822485000
H	6.345116000	0.381179000	0.735400000
H	5.850717000	-2.042478000	0.226941000
N	-2.370812000	1.584242000	-0.777133000
O	-3.746550000	-1.627517000	-0.594171000
H	-2.558908000	1.438602000	-1.761930000
H	2.713252000	2.127217000	0.560206000

RC-14 (R = CF3), E = -1205.275717

C	4.935123000	-1.498116000	0.237262000
H	4.793081000	-0.931790000	1.170890000
H	5.822441000	-1.100168000	-0.271624000
H	5.129711000	-2.551768000	0.508319000
C	2.527896000	-1.770100000	-0.314521000
C	2.168998000	-2.106805000	1.016992000
H	2.920377000	-2.099547000	1.806128000
C	0.851141000	-2.431121000	1.326415000
H	0.590183000	-2.669761000	2.360462000
C	-0.141494000	-2.434582000	0.337706000
C	0.217120000	-2.162617000	-0.993057000
H	-0.543220000	-2.186448000	-1.777552000
C	1.522609000	-1.826786000	-1.319234000
H	1.790326000	-1.593244000	-2.352393000
C	-0.575086000	0.540848000	0.689493000
H	-0.751296000	0.321998000	1.747082000
C	0.724261000	0.913720000	0.332931000
C	1.732309000	0.985338000	1.349142000
H	1.457230000	0.743961000	2.377705000
C	3.024291000	1.373661000	1.070136000
H	3.755654000	1.435628000	1.874552000
C	3.387407000	1.661268000	-0.270113000
C	2.413059000	1.581453000	-1.303444000
H	2.727663000	1.812810000	-2.321943000
C	1.124561000	1.213633000	-1.014622000
H	0.397325000	1.158175000	-1.824213000
C	5.640131000	2.252250000	0.307289000
H	6.513947000	2.580287000	-0.267010000
H	5.879868000	1.319929000	0.839642000
H	5.355817000	3.033637000	1.028826000
C	-1.718550000	0.481751000	-0.168322000

H	-1.599261000	0.691908000	-1.234521000
C	-2.946019000	0.160801000	0.306396000
H	-3.069709000	-0.061835000	1.372638000
C	-4.163435000	-0.009227000	-0.550062000
H	-4.004692000	0.464824000	-1.531021000
C	-4.481136000	-1.505500000	-0.745708000
H	-3.640939000	-2.001009000	-1.250822000
H	-5.383811000	-1.623261000	-1.362408000
H	-4.649127000	-1.999053000	0.222904000
C	-5.353932000	0.712245000	0.071060000
F	-5.661609000	0.212603000	1.284862000
F	-5.103743000	2.023980000	0.233411000
F	-6.451005000	0.602661000	-0.696323000
N	3.789890000	-1.359435000	-0.638715000
O	4.597350000	2.043478000	-0.650259000
H	3.987836000	-1.249706000	-1.626583000
H	-1.175747000	-2.673240000	0.592034000

TS-14 (R = H), E = -868.174426 (i = -370 cm-1)

C	3.540680000	1.994588000	-0.093749000
H	3.425540000	1.336790000	-0.967288000
H	4.442513000	1.695801000	0.453409000
H	3.663910000	3.032597000	-0.441997000
C	1.118584000	1.984146000	0.439933000
C	0.734437000	2.252368000	-0.922026000
H	1.490537000	2.525989000	-1.656894000
C	-0.576175000	2.135128000	-1.284527000
H	-0.869821000	2.307412000	-2.322726000
C	-1.565960000	1.653387000	-0.350696000
C	-1.216483000	1.728084000	1.050804000
H	-1.996984000	1.578882000	1.800069000
C	0.084881000	1.842791000	1.435406000
H	0.369960000	1.793423000	2.488532000
C	-1.566962000	-0.309415000	-0.683111000
H	-1.752509000	-0.239249000	-1.760022000
C	-0.224769000	-0.813906000	-0.365798000
C	0.769158000	-0.834445000	-1.366185000
H	0.510417000	-0.518907000	-2.379517000
C	2.076326000	-1.241389000	-1.106199000
H	2.804193000	-1.246834000	-1.916689000
C	2.436813000	-1.619579000	0.200155000
C	1.455876000	-1.619612000	1.213824000
H	1.749197000	-1.927346000	2.219051000
C	0.158093000	-1.223115000	0.935131000
H	-0.569394000	-1.219455000	1.747267000
C	4.720175000	-2.026341000	-0.390371000
H	5.621326000	-2.353147000	0.143347000
H	4.899616000	-1.028636000	-0.825488000
H	4.499570000	-2.741159000	-1.201004000
C	-2.743720000	-0.790120000	0.060749000
H	-2.617576000	-1.030271000	1.121088000

C	-3.956238000	-0.939427000	-0.501856000
H	-4.081542000	-0.677189000	-1.561710000
C	-5.187044000	-1.417156000	0.206523000
H	-4.936702000	-1.714708000	1.238333000
C	-6.302949000	-0.360010000	0.215807000
H	-5.983429000	0.546237000	0.755026000
H	-7.207342000	-0.750272000	0.708225000
H	-6.578610000	-0.061907000	-0.809077000
H	-5.561055000	-2.323459000	-0.304467000
N	2.395892000	1.868074000	0.795408000
O	3.675171000	-1.992831000	0.572384000
H	2.590235000	1.689609000	1.776294000
H	-2.613001000	1.816057000	-0.613590000

TS-14 (R = CF3), E = -1205.271076 (i = -352 cm-1)

C	-5.026378000	-1.255234000	-0.323269000
H	-4.677999000	-0.657437000	-1.177366000
H	-5.828306000	-0.705452000	0.183295000
H	-5.429156000	-2.210544000	-0.696608000
C	-2.723439000	-1.911901000	0.320425000
C	-2.345636000	-2.217475000	-1.035524000
H	-3.103715000	-2.249248000	-1.817062000
C	-1.032041000	-2.441602000	-1.332390000
H	-0.734121000	-2.642287000	-2.364115000
C	-0.009072000	-2.287703000	-0.328295000
C	-0.443748000	-2.320867000	1.048369000
H	0.302680000	-2.422669000	1.839175000
C	-1.749111000	-2.095080000	1.367600000
H	-2.071628000	-2.017608000	2.408107000
C	0.501336000	-0.341095000	-0.560793000
H	0.705714000	-0.415688000	-1.633412000
C	-0.678434000	0.466063000	-0.251271000
C	-1.583532000	0.801853000	-1.281887000
H	-1.365896000	0.486737000	-2.304914000
C	-2.746894000	1.527263000	-1.039305000
H	-3.406765000	1.770473000	-1.871069000
C	-3.058556000	1.917050000	0.277029000
C	-2.165704000	1.596892000	1.322368000
H	-2.418031000	1.916285000	2.334953000
C	-1.005688000	0.887796000	1.062485000
H	-0.345962000	0.648069000	1.896883000
C	-5.097613000	2.996899000	-0.364581000
H	-5.892193000	3.540666000	0.161220000
H	-5.537016000	2.129478000	-0.884718000
H	-4.628155000	3.665789000	-1.105264000
C	1.727918000	-0.217261000	0.243098000
H	1.624436000	-0.023798000	1.314924000
C	2.956849000	-0.322157000	-0.286979000
H	3.071402000	-0.523309000	-1.359180000
C	4.235775000	-0.205400000	0.495983000
H	4.012291000	0.091767000	1.532763000

C	5.024921000	-1.525361000	0.494185000
H	4.424142000	-2.319614000	0.958503000
H	5.961775000	-1.417564000	1.059758000
H	5.271446000	-1.830308000	-0.534104000
C	5.089252000	0.923773000	-0.070500000
F	5.431347000	0.695929000	-1.355853000
F	4.440055000	2.101527000	-0.036555000
F	6.232639000	1.078622000	0.621778000
N	-3.942638000	-1.471472000	0.622944000
O	-4.166299000	2.590851000	0.630827000
H	-4.142262000	-1.290561000	1.602570000
H	0.978570000	-2.693114000	-0.556388000

INT-14 (R = H), E = -868.186195

C	3.589395000	-3.461522000	0.082819000
H	3.930479000	-2.561123000	0.614595000
H	4.393638000	-3.812860000	-0.572790000
H	3.358226000	-4.248723000	0.815689000
C	1.269837000	-2.682840000	-0.315720000
C	1.032117000	-2.421541000	1.087970000
H	1.797809000	-2.671229000	1.821295000
C	-0.136933000	-1.867087000	1.480429000
H	-0.304216000	-1.665505000	2.541863000
C	-1.208447000	-1.457977000	0.536815000
C	-0.947596000	-1.858889000	-0.869621000
H	-1.733203000	-1.664702000	-1.603481000
C	0.211454000	-2.423095000	-1.271219000
H	0.377334000	-2.683883000	-2.319005000
C	-1.497334000	0.112361000	0.694776000
H	-1.805174000	0.224782000	1.745036000
C	-0.230170000	0.927601000	0.489866000
C	0.337986000	1.126891000	-0.775459000
H	-0.144617000	0.716516000	-1.664981000
C	1.526283000	1.846482000	-0.945298000
H	1.928409000	1.975990000	-1.949818000
C	2.178307000	2.389528000	0.173679000
C	1.616164000	2.203075000	1.449420000
H	2.127501000	2.635080000	2.312186000
C	0.434416000	1.484030000	1.597963000
H	0.017924000	1.350656000	2.600374000
C	3.950919000	3.331481000	-1.128175000
H	4.857526000	3.916133000	-0.924657000
H	3.296410000	3.907540000	-1.805820000
H	4.236201000	2.387083000	-1.624546000
C	-2.650012000	0.549205000	-0.173625000
H	-2.447537000	0.683765000	-1.242502000
C	-3.883821000	0.789337000	0.288005000
H	-4.082134000	0.653271000	1.360840000
C	-5.058366000	1.216425000	-0.544786000
H	-4.739704000	1.377430000	-1.588483000
C	-6.213723000	0.205701000	-0.496209000

H	-5.901952000	-0.769991000	-0.903069000
H	-7.075153000	0.561330000	-1.083356000
H	-6.556747000	0.042285000	0.538929000
H	-5.423592000	2.191593000	-0.172652000
N	2.422212000	-3.161016000	-0.740332000
O	3.332092000	3.098131000	0.124992000
H	2.515040000	-3.328767000	-1.740128000
H	-2.157549000	-1.929604000	0.857082000

INT-14 (R = CF3), E = -1205.282019

C	6.058517000	-2.757369000	0.300982000
H	6.282708000	-1.785147000	0.764381000
H	6.929809000	-3.085218000	-0.276567000
H	5.855412000	-3.495372000	1.090696000
C	3.689493000	-2.308632000	-0.278595000
C	3.329411000	-1.990556000	1.086424000
H	4.091263000	-2.006870000	1.864913000
C	2.051716000	-1.663813000	1.390390000
H	1.790540000	-1.420983000	2.424019000
C	0.944037000	-1.667138000	0.395200000
C	1.399558000	-1.914830000	-1.001922000
H	0.651490000	-1.875909000	-1.796445000
C	2.673868000	-2.241424000	-1.309869000
H	2.963387000	-2.455492000	-2.341091000
C	-0.097007000	-0.513953000	0.597252000
H	-0.394846000	-0.587769000	1.656764000
C	0.427810000	0.903481000	0.371777000
C	1.718408000	1.211086000	-0.067403000
H	2.434950000	0.420133000	-0.284659000
C	2.143448000	2.535572000	-0.245568000
H	3.160776000	2.721096000	-0.589095000
C	1.261650000	3.592448000	0.018492000
C	-0.044481000	3.297876000	0.456291000
H	-0.726282000	4.127506000	0.654958000
C	-0.447927000	1.979960000	0.624883000
H	-1.471718000	1.773923000	0.948068000
C	2.859546000	5.274072000	-0.561643000
H	2.876799000	6.371121000	-0.597866000
H	3.072682000	4.877395000	-1.569953000
H	3.644014000	4.924742000	0.132720000
C	-1.340647000	-0.745144000	-0.230372000
H	-1.252534000	-0.568093000	-1.308679000
C	-2.524376000	-1.088838000	0.285556000
H	-2.621448000	-1.241717000	1.367664000

11. References

[1] B. M. Trost; Z. Huang, *Angew. Chem. Int. Ed.* **2019**, *58*, 6396. *Angew. Chem.* **2019**, *58*, 6396.

[2] W. Huang, J. Bai, Y. Guo, Q. Chong, F. Meng, *Angew. Chem. Int. Ed.* **2023**, 62, e202219257; *Angew. Chem.* **2023**, *135*, e202219257.

[3] Cong, X.; Hao, N.; Mishra, A.; Zhuo, Q.; An, K.; Nishiura, M.; Hou, Z. *J. Am. Chem. Soc.* **2024**, *146*, 10187–10198

[4] Landge V. G.; Shrestha, K. K.; Grant, A. J.; Grant,;Young, M. C. *Org. Lett.* **2020**, *22*, 9745–9750.

[5] X.-X. Chen, H. Luo, Y.-W. Chen, Y. Liu, Z.-T. He, *Angew. Chemie Int. Ed.* **2023**, *62*, e202307628, *Angew. Chem. Int. Ed.* **2023**, *62*, e202307628; *Angew. Chem.* **2019**, *135*, e202307628.

[6] G. Wang, L. Gao, H. Chen, X. Liu, J. Cao, S. Chen, X. Cheng, S. Li, *Angew. Chem. Int. Ed.* **2019**, *58*, 1694–1699; *Angew. Chem.* **2019**, *131*, 1708–1713.

[7] M. Du, X. Wang, J. Zhang, P. Liu, C.-T. Li, *J. Org. Chem.* **2023**, *88*, 9496–9504.

[8] I. S. Cho, L. Gong, J. M. Muchowski, *J. Org. Chem.* **1991**, *56*, 7288–7291.

[9] R. Shimizu, H. Egami, Y. Hamashima, M. Sodeoka, *Angew. Chemie Int. Ed.* **2012**, *51*, 4577–4580; *Angew. Chem.* **2012**, 124, 4655–4658.

[10] Olah, G. A.; Kuhn, S. J.; Flood, S. H. *J. Am. Chem. Soc.* **1961**, *8*, 4571–4580. DOI: ja01483a017.

[11] Olah, G. A.; Kuhn, S. J.; Flood, S. H. *J. Am. Chem. Soc.* **1962**, *84*, 1688–1695. DOI: ja00868a039.

[12] Nakane, R.; Kurihara, O.; Takematsu, A. *J. Org. Chem.* **1971**, *36*, 2753–2756. DOI: jo00818a006.

[13] Brunen S.; Mitschke B.; Leutzsach M.; and List, B. *J. Am. Chem. Soc.* **2023**, *145*, 15708-15713. DOI: 10.1021/jacs.3c05148

[14] Melander L., *Isotope Effects on Reaction Rates*; The Ronald Press Company, 1960.

[15] Gaussian 09, Revision D.01, Frisch, M. J.; Trucks, G. W.; Schlegel, H. B.; Scuseria, G. E.; Robb, M. A.; Cheeseman, J. R.; Scalmani, G.; Barone, V.; Mennucci, B.; Petersson, G. A.; Nakatsuji, H.; Caricato, M.; Li, X.; Hratchian, H. P.; Izmaylov, A. F.; Bloino, J.; Zheng, G.; Sonnenberg, J. L.; Hada, M.; Ehara, M.; Toyota, K.; Fukuda, R.; Hasegawa, J.; Ishida, M.; Nakajima, T.; Honda, Y.; Kitao, O.; Nakai, H.; Vreven, T.; Montgomery, J. A., Jr.; Peralta, J. E.; Ogliaro, F.; Bearpark, M.; Heyd, J. J.; Brothers, E.; Kudin, K. N.; Staroverov, V. N.; Kobayashi, R.; Normand, J.; Raghavachari, K.; Rendell, A.; Burant, J. C.; Iyengar, S. S.; Tomasi, J.; Cossi, M.; Rega, N.; Millam, J. M.; Klene, M.; Knox, J. E.; Cross, J. B.; Bakken, V.; Adamo, C.; Jaramillo, J.; Gomperts, R.; Stratmann, R. E.; Yazyev, O.; Austin, A. J.; Cammi, R.; Pomelli, C.; Ochterski, J. W.; Martin, R. L.; Morokuma, K.; Zakrzewski, V. G.; Voth, G. A.; Salvador, P.; Dannenberg, J. J.; Dapprich, S.; Daniels, A. D.; Farkas, Ö.; Foresman, J. B.; Ortiz, J. V.; Cioslowski, J.; Fox, D. J. Gaussian, Inc., Wallingford CT, 2009.

[16] a) A. D. Becke, *J. Chem. Phys*. **1993**, *98*, 5648; b) C. Lee, W. Yang, R. G. Parr, R. G. *Phys. Rev. B* **1998**, *37*, 785; c) S. H. Vosko, L. Wilk, M. Nusair, *Can. J. Phys*. **1980**, *58*, 1200.

[17] S. Grimme, J. Antony, S. Ehrlich, H. Krieg, *J. Chem. Phys*. **2010**, *132*, 154104.

[18] F. Weigend, R. Ahlrichs, *Phys. Chem. Chem. Phys*. **2005**, *7*, 3297

[19] a) S. Miertuš, E. Scrocco, J. Tomasi, *Chem. Phys*. **1981**, *55*, 117; b) J. L. Pascual-Ahuir, E. Silla, I. Tuñón, *J. Comput. Chem*. **1994**, *15*, 1127; c) V. Barone, M. Cossi, *J. Phys. Chem. A* **1998**, *102*, 1995.

[20] J. W. McIver, A. K.; Komornicki, *J. Am. Chem. Soc.* **1972**, *94*, 2625.

[21] C. González, H. B. Schlegel, *J. Phys. Chem*. **1990**, *94*, 5523.

[22] Y. Zhao, D. G. Truhlar, *Theor. Chem. Acc.* **2008**, *120*, 215.

[23] a) I. Fernández, F. M. Bickelhaupt, *Chem. Soc. Rev*. **2014**, *43*, 4953–4967; b) L. P. Wolters, F. M. Bickelhaupt, *WIREs Comput. Mol. Sci.* **2015**, *5*, 324–343; c) F. M. Bickelhaupt, K. N. Houk, *Angew. Chem. Int. Ed.* **2017**, *56*,10070–10086; *Angew. Chem*. **2017**, *129*, 10204-10221. See also; d) I. Fernández, in Discovering the Future of Molecular Sciences (Ed.: B.Pignataro), Wiley-VCH, Weinheim, 2014, pp. 165–187

12. X-Ray diffraction analysis

Crystal structure report for compound 6

Compound **6** was crystallized using a mixture of THF :Pentane (compound **6** was dissolved in THF and exposed to pentane vapours) in order to obtain appropriate crystals for X-ray analysis.

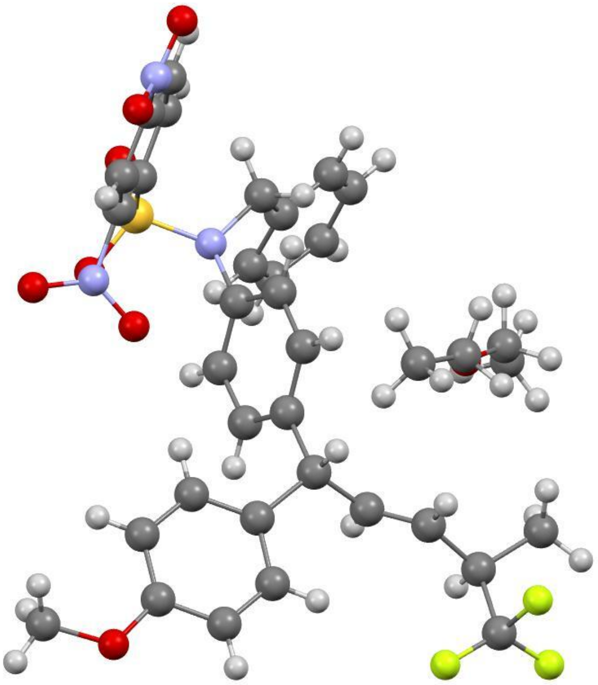

Figure S7. Ball and stick plot of compound **6** with non-hydrogen atoms labelled. Compound **6** collection details are gathered in the following tables:

Table S3. Sample and crystal data for 6.

**Identification code**	03628
**Chemical formula**	$C_{33}H_{30.50}F_3N_3O_{7.25}S$
**Formula weight**	674.16 g/mol
**Temperature**	250(2) K
**Wavelength**	0.71073 Å
**Crystal size**	0.014 x 0.031 x 0.214 mm
**Crystal habit**	clear colourless ribbon
**Crystal system**	monoclinic

**Space group**	C 1 2/c 1	
**Unit cell dimensions**	a = 31.226(3) Å	α = 90°
	b = 5.8199(5) Å	β = 106.344(3)°
	c = 38.948(3) Å	γ = 90°
**Volume**	6792.1(10) $Å^3$	
**Z**	8	
**Density (calculated)**	1.319 g/cm^3	
**Absorption coefficient**	0.162 mm^{-1}	
**F(000)**	2804	

Table S4. Data collection and structure refinement for 6.

**Theta range for data collection**	2.63 to 25.08°	
**Index ranges**	-36<=h<=36, -6<=k<=6, -46<=l<=46	
**Reflections collected**	42831	
**Independent reflections**	5989 [R(int) = 0.2402]	
**Coverage of independent reflections**	99.5%	
**Absorption correction**	Multi-Scan	
**Max. and min. transmission**	0.9980 and 0.9660	
**Structure solution technique**	direct methods	
**Structure solution program**	XT, VERSION 2018/2	
**Refinement method**	Full-matrix least-squares on F^2	
**Refinement program**	SHELXL-2019/1 (Sheldrick, 2019)	
**Function minimized**	$\Sigma w(F_o^2 - F_c^2)^2$	
**Data / restraints / parameters**	5989 / 395 / 470	
**Goodness-of-fit on F^2**	1.013	
**Final R indices**	2357 data; I>2σ(I)	R1 = 0.0916, wR2 = 0.2240
	all data	R1 = 0.2418, wR2 = 0.3020

**Weighting scheme**	$w=1/[\sigma^2(F_o^2)+(0.1394P)^2+5.6177P]$ where $P=(F_o^2+2F_c^2)/3$
**Largest diff. peak and hole**	0.578 and -0.417 eÅ^{-3}
**R.M.S. deviation from mean**	0.085 eÅ^{-3}

Table S5. Atomic coordinates and equivalent isotropic atomic displacement parameters (Å^2) for 6.

U(eq) is defined as one third of the trace of the orthogonalized U_{ij} tensor.

	**x/a**	**y/b**	**z/c**	**U(eq)**
C1	0.7845(2)	0.4652(11)	0.33641(16)	0.0370(15)
C2	0.8026(2)	0.5044(11)	0.37288(17)	0.0400(15)
C3	0.8380(2)	0.3811(12)	0.39355(18)	0.0462(17)
C4	0.8546(2)	0.2097(12)	0.37730(19)	0.0463(17)
C5	0.8377(2)	0.1619(12)	0.34092(19)	0.0488(17)
C6	0.8028(2)	0.2918(11)	0.32025(18)	0.0431(17)
C7	0.6842(2)	0.2925(12)	0.27575(17)	0.0505(19)
C8	0.6428(2)	0.3422(12)	0.24608(17)	0.0448(16)
C9	0.6191(3)	0.5473(14)	0.24293(19)	0.060(2)
C10	0.5808(3)	0.5780(16)	0.2140(2)	0.074(2)
C11	0.5664(3)	0.4149(17)	0.1889(2)	0.077(2)
C12	0.5908(3)	0.2159(17)	0.1917(2)	0.082(3)
C13	0.6286(3)	0.1779(14)	0.2201(2)	0.065(2)
C14	0.6777(2)	0.4483(11)	0.33402(16)	0.0391(16)
C15	0.6874(2)	0.2563(12)	0.35512(17)	0.0468(17)
C16	0.6716(3)	0.2323(13)	0.38430(19)	0.0540(19)
C17	0.6448(3)	0.3959(13)	0.39276(19)	0.0568(19)
C18	0.6341(2)	0.5899(13)	0.37088(19)	0.0570(19)
C19	0.6502(2)	0.6181(12)	0.34154(18)	0.0494(18)
C20	0.6283(3)	0.3642(15)	0.4258(2)	0.068(2)

	x/a	y/b	z/c	U(eq)
C21	0.6419(2)	0.5603(12)	0.45304(18)	0.0497(18)
C22	0.6786(2)	0.6965(12)	0.45512(18)	0.0477(18)
C23	0.6924(2)	0.8619(12)	0.48128(16)	0.0461(17)
C24	0.6701(2)	0.8909(12)	0.50677(17)	0.0497(18)
C25	0.6338(3)	0.7520(14)	0.50595(19)	0.059(2)
C26	0.6200(3)	0.5907(13)	0.47925(18)	0.058(2)
C27	0.7143(3)	0.2135(14)	0.5332(2)	0.065(2)
C28	0.5816(3)	0.3097(16)	0.4164(2)	0.072(2)
C29	0.5583(3)	0.1482(15)	0.4234(2)	0.067(2)
C30	0.5104(3)	0.1116(19)	0.4162(3)	0.094(3)
C31	0.4946(3)	0.9033(19)	0.3939(3)	0.096(3)
F1A	0.5042(4)	0.7138(18)	0.4142(3)	0.154(4)
F2A	0.5100(4)	0.874(2)	0.3675(3)	0.136(4)
F3A	0.4508(3)	0.9060(16)	0.3848(3)	0.117(3)
C32	0.4944(4)	0.104(3)	0.4504(3)	0.120(4)
F1B	0.5126(6)	0.893(4)	0.4638(5)	0.120(6)
F2B	0.5097(9)	0.279(5)	0.4708(7)	0.153(8)
F3B	0.4502(6)	0.052(4)	0.4390(6)	0.128(7)
N1	0.69506(17)	0.4748(9)	0.30359(13)	0.0395(13)
N2	0.7846(2)	0.6829(10)	0.39241(15)	0.0490(15)
N3	0.8905(2)	0.0626(12)	0.39960(19)	0.0613(17)
O1	0.73604(16)	0.8311(7)	0.32672(12)	0.0514(13)
O2	0.74822(16)	0.6350(9)	0.27393(12)	0.0585(14)
O3	0.74901(17)	0.6484(9)	0.39802(13)	0.0598(14)
O4	0.80904(18)	0.8465(9)	0.40373(14)	0.0685(16)
O5	0.9035(2)	0.1081(11)	0.43118(16)	0.0834(18)

	x/a	y/b	z/c	U(eq)
O6	0.9038(2)	0.9034(12)	0.38531(17)	0.097(2)
O7	0.67972(18)	0.0513(9)	0.53333(12)	0.0656(15)
S1	0.73985(6)	0.6238(3)	0.30814(5)	0.0447(5)
O8	0.500000	0.105(5)	0.250000	0.140(9)
C33	0.5279(7)	0.030(5)	0.2792(5)	0.133(8)
C34	0.5602(6)	0.116(4)	0.3063(4)	0.092(6)

Table S6. Bond lengths (Å) for 6.

C1-C2	1.392(8)	C1-C6	1.394(9)
C1-S1	1.773(7)	C2-C3	1.371(9)
C2-N2	1.489(9)	C3-C4	1.361(9)
C4-C5	1.394(9)	C4-N3	1.481(9)
C5-C6	1.384(9)	C7-N1	1.486(8)
C7-C8	1.501(10)	C8-C13	1.373(10)
C8-C9	1.391(10)	C9-C10	1.405(10)
C10-C11	1.345(11)	C11-C12	1.373(12)
C12-C13	1.388(11)	C14-C15	1.369(9)
C14-C19	1.393(9)	C14-N1	1.444(8)
C15-C16	1.366(9)	C16-C17	1.367(10)
C17-C18	1.397(10)	C17-C20	1.528(10)
C18-C19	1.382(9)	C20-C28	1.436(11)
C20-C21	1.534(10)	C21-C22	1.377(9)
C21-C26	1.391(9)	C22-C23	1.380(9)
C23-C24	1.374(9)	C24-O7	1.363(8)
C24-C25	1.386(10)	C25-C26	1.377(10)
C27-O7	1.436(9)	C28-C29	1.266(10)
C29-C30	1.455(11)	C30-C31	1.493(13)

C30-C32	1.549(13)	C31-F2A	1.261(11)
C31-F3A	1.315(10)	C31-F1A	1.341(11)
C32-F2B	1.300(17)	C32-F3B	1.358(15)
C32-F1B	1.391(15)	N1-S1	1.612(6)
N2-O3	1.208(7)	N2-O4	1.223(7)
N3-O5	1.211(8)	N3-O6	1.213(8)
O1-S1	1.429(5)	O2-S1	1.430(4)
O8-C33	1.30(2)	O8-C33#1	1.30(2)
C33-C34	1.335(16)		

Symmetry transformations used to generate equivalent atoms:

#1 -x+1, y, -z+1/2

Table S7. Bond angles (°) for 6.

C2-C1-C6	118.6(6)	C2-C1-S1	124.8(5)
C6-C1-S1	116.6(5)	C3-C2-C1	122.8(6)
C3-C2-N2	115.0(6)	C1-C2-N2	122.2(6)
C4-C3-C2	117.4(6)	C3-C4-C5	122.3(7)
C3-C4-N3	118.4(7)	C5-C4-N3	119.2(6)
C6-C5-C4	119.6(6)	C5-C6-C1	119.3(6)
N1-C7-C8	113.2(6)	C13-C8-C9	118.6(7)
C13-C8-C7	117.6(7)	C9-C8-C7	123.7(6)
C8-C9-C10	119.4(7)	C11-C10-C9	121.8(9)
C10-C11-C12	118.3(9)	C11-C12-C13	121.7(9)
C8-C13-C12	120.2(8)	C15-C14-C19	119.8(6)
C15-C14-N1	120.6(6)	C19-C14-N1	119.5(6)
C16-C15-C14	120.8(7)	C15-C16-C17	121.1(7)
C16-C17-C18	118.3(7)	C16-C17-C20	119.6(7)

C18-C17-C20	122.1(7)	C19-C18-C17	121.3(7)
C18-C19-C14	118.6(7)	C28-C20-C17	111.8(7)
C28-C20-C21	113.9(7)	C17-C20-C21	113.5(6)
C22-C21-C26	117.2(7)	C22-C21-C20	122.3(6)
C26-C21-C20	120.3(6)	C21-C22-C23	121.9(6)
C24-C23-C22	120.1(7)	O7-C24-C23	125.7(7)
O7-C24-C25	114.9(6)	C23-C24-C25	119.4(7)
C26-C25-C24	119.7(7)	C25-C26-C21	121.8(7)
C29-C28-C20	135.5(10)	C28-C29-C30	133.5(10)
C29-C30-C31	112.6(9)	C29-C30-C32	113.7(9)
C31-C30-C32	110.1(10)	F2A-C31-F3A	113.0(11)
F2A-C31-F1A	107.3(12)	F3A-C31-F1A	102.1(10)
F2A-C31-C30	116.5(10)	F3A-C31-C30	107.1(10)
F1A-C31-C30	109.9(10)	F2B-C32-F3B	123.(2)
F2B-C32-F1B	115.(2)	F3B-C32-F1B	101.3(16)
F2B-C32-C30	110.4(15)	F3B-C32-C30	105.6(13)
F1B-C32-C30	98.7(12)	C14-N1-C7	117.5(5)
C14-N1-S1	118.8(4)	C7-N1-S1	118.4(4)
O3-N2-O4	125.3(6)	O3-N2-C2	118.4(6)
O4-N2-C2	116.1(6)	O5-N3-O6	124.9(7)
O5-N3-C4	116.8(7)	O6-N3-C4	118.2(7)
C24-O7-C27	116.9(5)	O1-S1-O2	119.8(3)
O1-S1-N1	108.5(3)	O2-S1-N1	107.6(3)
O1-S1-C1	106.7(3)	O2-S1-C1	106.5(3)
N1-S1-C1	107.1(3)	C33-O8-C33#1	141.(4)
O8-C33-C34	138.(3)		

Symmetry transformations used to generate equivalent atoms:

#1 -x+1, y, -z+1/2

Table S8. Torsion angles (°) for 6.

C6-C1-C2-C3	0.6(10)	S1-C1-C2-C3	-178.3(5)
C6-C1-C2-N2	-178.8(6)	S1-C1-C2-N2	2.4(9)
C1-C2-C3-C4	-1.9(10)	N2-C2-C3-C4	177.5(6)
C2-C3-C4-C5	1.6(10)	C2-C3-C4-N3	-175.4(6)
C3-C4-C5-C6	0.0(10)	N3-C4-C5-C6	177.0(6)
C4-C5-C6-C1	-1.4(10)	C2-C1-C6-C5	1.0(10)
S1-C1-C6-C5	-180.0(5)	N1-C7-C8-C13	-176.4(6)
N1-C7-C8-C9	5.8(10)	C13-C8-C9-C10	1.9(11)
C7-C8-C9-C10	179.6(7)	C8-C9-C10-C11	-0.6(13)
C9-C10-C11-C12	-1.3(14)	C10-C11-C12-C13	2.0(14)
C9-C8-C13-C12	-1.2(11)	C7-C8-C13-C12	-179.0(7)
C11-C12-C13-C8	-0.8(13)	C19-C14-C15-C16	-2.3(10)
N1-C14-C15-C16	178.8(6)	C14-C15-C16-C17	1.8(11)
C15-C16-C17-C18	-0.4(11)	C15-C16-C17-C20	-179.4(7)
C16-C17-C18-C19	-0.4(11)	C20-C17-C18-C19	178.6(7)
C17-C18-C19-C14	-0.1(11)	C15-C14-C19-C18	1.4(10)
N1-C14-C19-C18	-179.7(6)	C16-C17-C20-C28	-107.8(9)
C18-C17-C20-C28	73.2(10)	C16-C17-C20-C21	121.6(8)
C18-C17-C20-C21	-57.4(10)	C28-C20-C21-C22	-153.9(8)
C17-C20-C21-C22	-24.4(11)	C28-C20-C21-C26	33.0(11)
C17-C20-C21-C26	162.5(7)	C26-C21-C22-C23	-2.1(11)
C20-C21-C22-C23	-175.4(7)	C21-C22-C23-C24	1.6(11)
C22-C23-C24-O7	-178.2(7)	C22-C23-C24-C25	0.2(11)

O7-C24-C25-C26	177.2(7)	C23-C24-C25-C26	-1.4(12)
C24-C25-C26-C21	0.8(12)	C22-C21-C26-C25	0.9(12)
C20-C21-C26-C25	174.4(8)	C17-C20-C28-C29	123.6(10)
C21-C20-C28-C29	-106.1(11)	C20-C28-C29-C30	172.4(9)
C28-C29-C30-C31	119.3(12)	C28-C29-C30-C32	-114.7(12)
C29-C30-C31-F2A	-43.5(15)	C32-C30-C31-F2A	-171.4(12)
C29-C30-C31-F3A	-171.1(9)	C32-C30-C31-F3A	61.0(12)
C29-C30-C31-F1A	78.7(12)	C32-C30-C31-F1A	-49.2(13)
C29-C30-C32-F2B	50.(2)	C31-C30-C32-F2B	177.0(18)
C29-C30-C32-F3B	-175.5(13)	C31-C30-C32-F3B	-48.2(17)
C29-C30-C32-F1B	-71.1(15)	C31-C30-C32-F1B	56.2(15)
C15-C14-N1-C7	57.2(8)	C19-C14-N1-C7	-121.7(7)
C15-C14-N1-S1	-96.8(7)	C19-C14-N1-S1	84.3(7)
C8-C7-N1-C14	92.0(7)	C8-C7-N1-S1	-113.8(6)
C3-C2-N2-O3	-107.2(7)	C1-C2-N2-O3	72.3(8)
C3-C2-N2-O4	67.8(8)	C1-C2-N2-O4	-112.8(7)
C3-C4-N3-O5	-1.5(10)	C5-C4-N3-O5	-178.6(7)
C3-C4-N3-O6	176.6(7)	C5-C4-N3-O6	-0.4(10)
C23-C24-O7-C27	4.3(11)	C25-C24-O7-C27	-174.2(7)
C14-N1-S1-O1	-45.5(5)	C7-N1-S1-O1	160.7(5)
C14-N1-S1-O2	-176.5(5)	C7-N1-S1-O2	29.7(5)
C14-N1-S1-C1	69.3(5)	C7-N1-S1-C1	-84.5(5)
C2-C1-S1-O1	17.0(6)	C6-C1-S1-O1	-161.9(5)
C2-C1-S1-O2	146.0(5)	C6-C1-S1-O2	-32.9(6)
C2-C1-S1-N1	-99.0(6)	C6-C1-S1-N1	82.1(5)
C33#1-O8-C33-C34	169.(3)		

Symmetry transformations used to generate equivalent atoms:

#1 -x+1, y, -z+1/2

Table S9. Anisotropic atomic displacement parameters (Å^2) for 6.

The anisotropic atomic displacement factor exponent takes the form: $-2\pi^2[h^2 a^{*2} U_{11} + ... + 2 h k a^* b^* U_{12}]$

	U_{11}	U_{22}	U_{33}	U_{23}	U_{13}	U_{12}
C1	0.051(4)	0.027(3)	0.038(3)	0.004(2)	0.022(3)	-0.001(3)
C2	0.052(4)	0.034(3)	0.043(3)	0.001(3)	0.028(3)	-0.004(3)
C3	0.053(4)	0.045(4)	0.045(4)	0.002(3)	0.021(3)	-0.003(3)
C4	0.048(4)	0.044(4)	0.051(3)	0.008(3)	0.021(3)	0.003(3)
C5	0.056(4)	0.040(4)	0.058(4)	0.006(3)	0.029(3)	0.007(3)
C6	0.055(4)	0.036(4)	0.044(4)	0.000(3)	0.022(3)	0.005(3)
C7	0.071(4)	0.047(4)	0.034(3)	-0.006(3)	0.016(3)	0.011(3)
C8	0.053(4)	0.049(4)	0.037(3)	0.001(3)	0.021(3)	-0.005(3)
C9	0.068(5)	0.058(5)	0.049(4)	-0.001(3)	0.006(3)	0.011(4)
C10	0.071(5)	0.079(6)	0.064(5)	0.010(4)	0.008(4)	0.008(4)
C11	0.071(5)	0.088(6)	0.065(5)	0.007(4)	0.007(4)	-0.019(4)
C12	0.090(6)	0.081(6)	0.064(5)	-0.008(4)	0.004(4)	-0.021(4)
C13	0.085(5)	0.057(5)	0.051(4)	-0.008(3)	0.013(4)	-0.007(4)
C14	0.045(4)	0.044(4)	0.031(3)	-0.007(3)	0.016(3)	-0.003(3)
C15	0.063(5)	0.038(4)	0.043(4)	-0.004(3)	0.022(3)	-0.005(3)
C16	0.077(5)	0.047(4)	0.043(4)	-0.014(3)	0.026(3)	-0.018(3)
C17	0.070(5)	0.055(4)	0.054(4)	-0.014(3)	0.032(3)	-0.025(3)
C18	0.061(5)	0.058(5)	0.062(4)	-0.017(3)	0.032(4)	-0.004(4)
C19	0.057(4)	0.047(4)	0.050(4)	-0.003(3)	0.026(3)	0.006(3)
C20	0.083(5)	0.070(5)	0.064(4)	-0.019(4)	0.041(4)	-0.027(4)
C21	0.063(4)	0.051(4)	0.043(3)	-0.004(3)	0.028(3)	-0.009(3)
C22	0.059(4)	0.047(4)	0.046(4)	-0.003(3)	0.030(3)	-0.006(3)

	U_{11}	U_{22}	U_{33}	U_{23}	U_{13}	U_{12}
C23	0.061(4)	0.042(4)	0.039(3)	0.004(3)	0.019(3)	-0.005(3)
C24	0.070(4)	0.051(4)	0.033(3)	0.001(3)	0.023(3)	-0.002(3)
C25	0.077(5)	0.070(5)	0.041(4)	-0.009(3)	0.033(4)	-0.013(4)
C26	0.068(5)	0.067(5)	0.051(4)	-0.014(3)	0.035(4)	-0.016(4)
C27	0.089(6)	0.057(5)	0.055(5)	-0.015(4)	0.029(4)	-0.012(4)
C28	0.075(5)	0.089(6)	0.062(5)	-0.018(4)	0.037(4)	-0.029(4)
C29	0.071(5)	0.066(5)	0.072(5)	-0.007(4)	0.035(4)	-0.010(4)
C30	0.078(5)	0.108(6)	0.100(6)	-0.003(5)	0.033(4)	-0.021(4)
C31	0.075(5)	0.094(6)	0.112(7)	-0.005(5)	0.016(5)	-0.021(5)
F1A	0.146(8)	0.120(7)	0.201(9)	0.036(6)	0.058(7)	0.031(6)
F2A	0.121(7)	0.164(9)	0.127(7)	-0.053(6)	0.043(5)	-0.057(6)
F3A	0.082(5)	0.115(7)	0.145(7)	0.016(5)	0.015(4)	-0.010(4)
C32	0.100(7)	0.165(10)	0.108(7)	0.011(6)	0.053(6)	0.003(7)
F1B	0.088(11)	0.176(12)	0.119(12)	0.030(9)	0.064(9)	0.031(9)
F2B	0.129(15)	0.197(14)	0.160(13)	-0.025(10)	0.082(11)	-0.014(10)
F3B	0.093(8)	0.136(15)	0.168(14)	0.042(11)	0.058(8)	0.015(8)
N1	0.050(3)	0.041(3)	0.031(3)	-0.006(2)	0.018(2)	0.005(2)
N2	0.065(4)	0.040(3)	0.048(3)	-0.004(3)	0.025(3)	-0.007(3)
N3	0.059(4)	0.066(4)	0.063(4)	0.015(3)	0.023(3)	0.010(3)
O1	0.073(3)	0.031(3)	0.059(3)	0.002(2)	0.033(3)	0.005(2)
O2	0.078(4)	0.065(3)	0.043(3)	0.017(2)	0.034(2)	0.013(3)
O3	0.062(3)	0.062(4)	0.064(3)	-0.016(3)	0.032(3)	-0.011(3)
O4	0.086(4)	0.043(3)	0.087(4)	-0.023(3)	0.041(3)	-0.020(3)
O5	0.082(4)	0.093(5)	0.067(3)	0.011(3)	0.007(3)	0.016(3)
O6	0.107(5)	0.093(5)	0.093(4)	0.005(4)	0.030(4)	0.052(4)
O7	0.088(4)	0.068(4)	0.047(3)	-0.019(3)	0.030(3)	-0.018(3)

	U_{11}	U_{22}	U_{33}	U_{23}	U_{13}	U_{12}
S1	0.0633(13)	0.0368(11)	0.0413(10)	0.0069(8)	0.0264(9)	0.0071(9)
O8	0.080(12)	0.26(3)	0.079(11)	0.000000	0.024(9)	0.000000
C33	0.088(13)	0.23(2)	0.084(11)	-0.016(11)	0.020(8)	-0.005(12)
C34	0.071(11)	0.138(18)	0.067(9)	0.029(10)	0.017(8)	-0.011(11)

Table S10. Hydrogen atomic coordinates and isotropic atomic displacement parameters (Å^2) for 6.

	x/a	y/b	z/c	U(eq)
H3	0.8502	0.4138	0.4180	0.055000
H5	0.8499	0.0422	0.3305	0.059000
H6	0.7915	0.2633	0.2956	0.052000
H7A	0.6804	0.1463	0.2870	0.061000
H7B	0.7094	0.2753	0.2656	0.061000
H9	0.6287	0.6644	0.2600	0.072000
H10	0.5647	0.7162	0.2121	0.089000
H11	0.5404	0.4366	0.1700	0.092000
H12	0.5817	0.1026	0.1739	0.099000
H13	0.6444	0.0393	0.2215	0.078000
H15	0.7050	0.1396	0.3494	0.056000
H16	0.6794	0.1012	0.3988	0.065000
H18	0.6156	0.7035	0.3762	0.068000
H19	0.6428	0.7491	0.3270	0.059000
H20	0.6438	0.2259	0.4381	0.082000
H22	0.6947	0.6762	0.4382	0.057000
H23	0.7172	0.9550	0.4817	0.055000
H25	0.6186	0.7679	0.5235	0.071000
H26	0.5951	0.4986	0.4787	0.070000

	x/a	y/b	z/c	U(eq)
H27A	0.7156	1.3301	0.5512	0.098000
H27B	0.7080	1.2855	0.5098	0.098000
H27C	0.7428	1.1344	0.5384	0.098000
H28	0.5640	0.4210	0.4013	0.086000
H29	0.5755	0.0262	0.4360	0.080000
H30	0.4956	0.2452	0.4021	0.112000
H31A	0.4840	-0.0525	0.3689	0.106000
H31B	0.4706	-0.1685	0.4012	0.115000
H31C	0.5191	-0.2046	0.3970	0.115000
H32A	0.4696	-0.0027	0.4468	0.143000
H32B	0.4848	0.2558	0.4553	0.143000
H32C	0.5187	0.0532	0.4705	0.143000
H33A	0.5430	-0.0951	0.2705	0.160000
H33B	0.5082	-0.0454	0.2914	0.160000
H34A	0.5887	0.0990	0.3012	0.139000
H34B	0.5608	0.0329	0.3281	0.139000
H34C	0.5545	0.2771	0.3094	0.139000

Table S11. Hydrogen bond distances (Å) and angles (°) for 6.

	Donor-H	Acceptor-H	Donor-Acceptor	Angle
C3-H3···O7#3	0.94	2.35	3.075(8)	134.0
C7-H7B···O2#2	0.98	2.43	3.366(8)	158.5
C15-H15···O1#1	0.94	2.33	3.256(8)	168.7

Symmetry transformations used to generate equivalent atoms:

#1 -x+1, y, -z+1/2

13. NMR Spectra

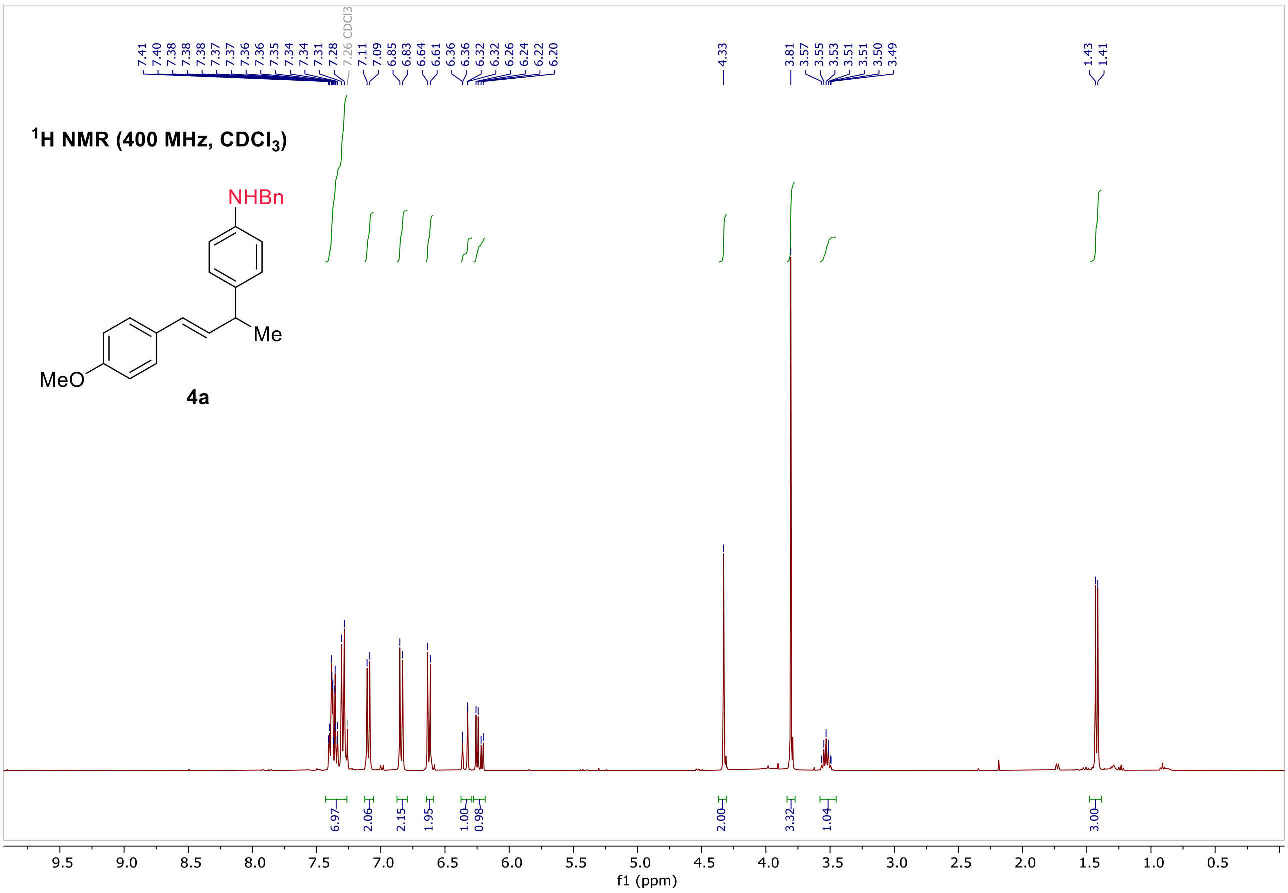

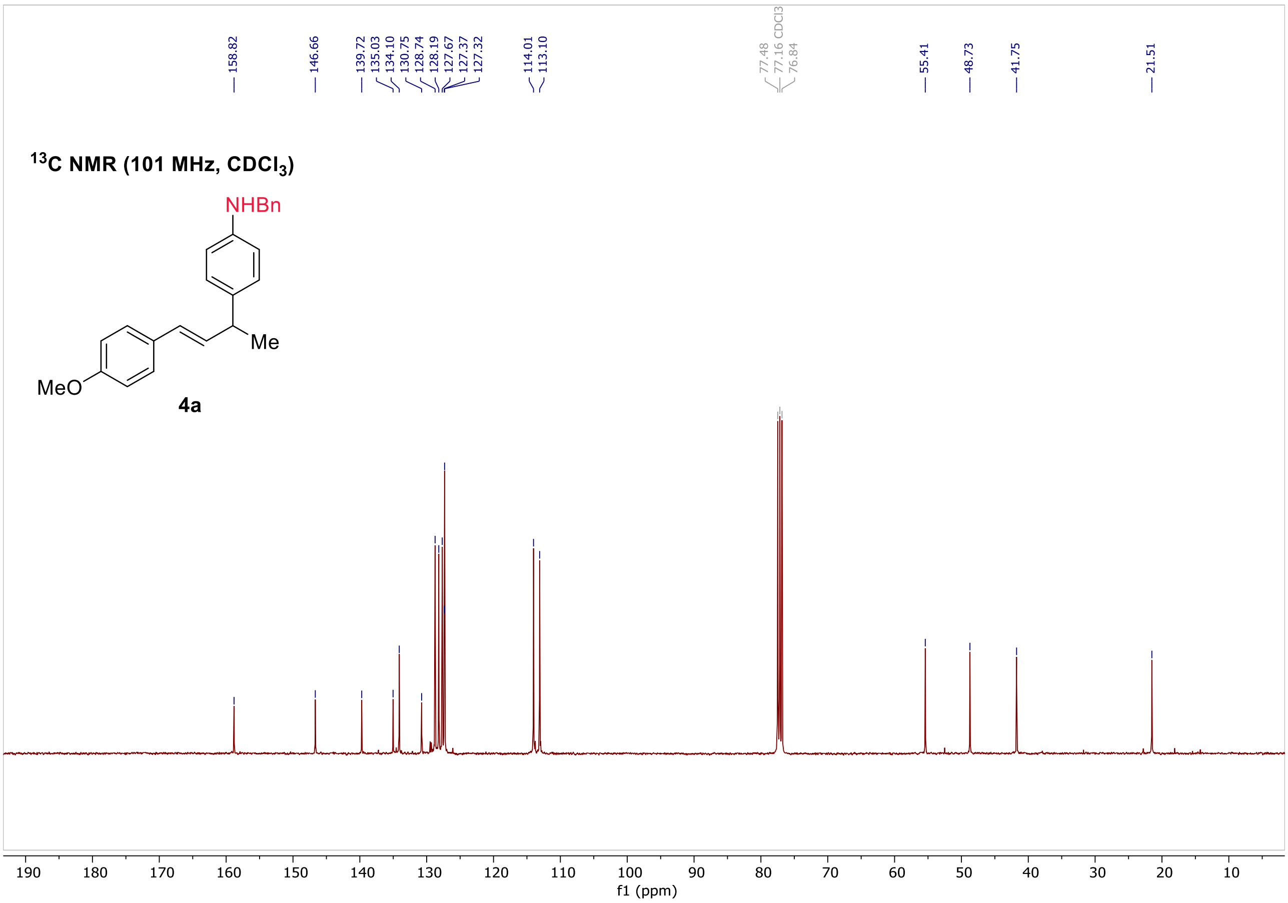

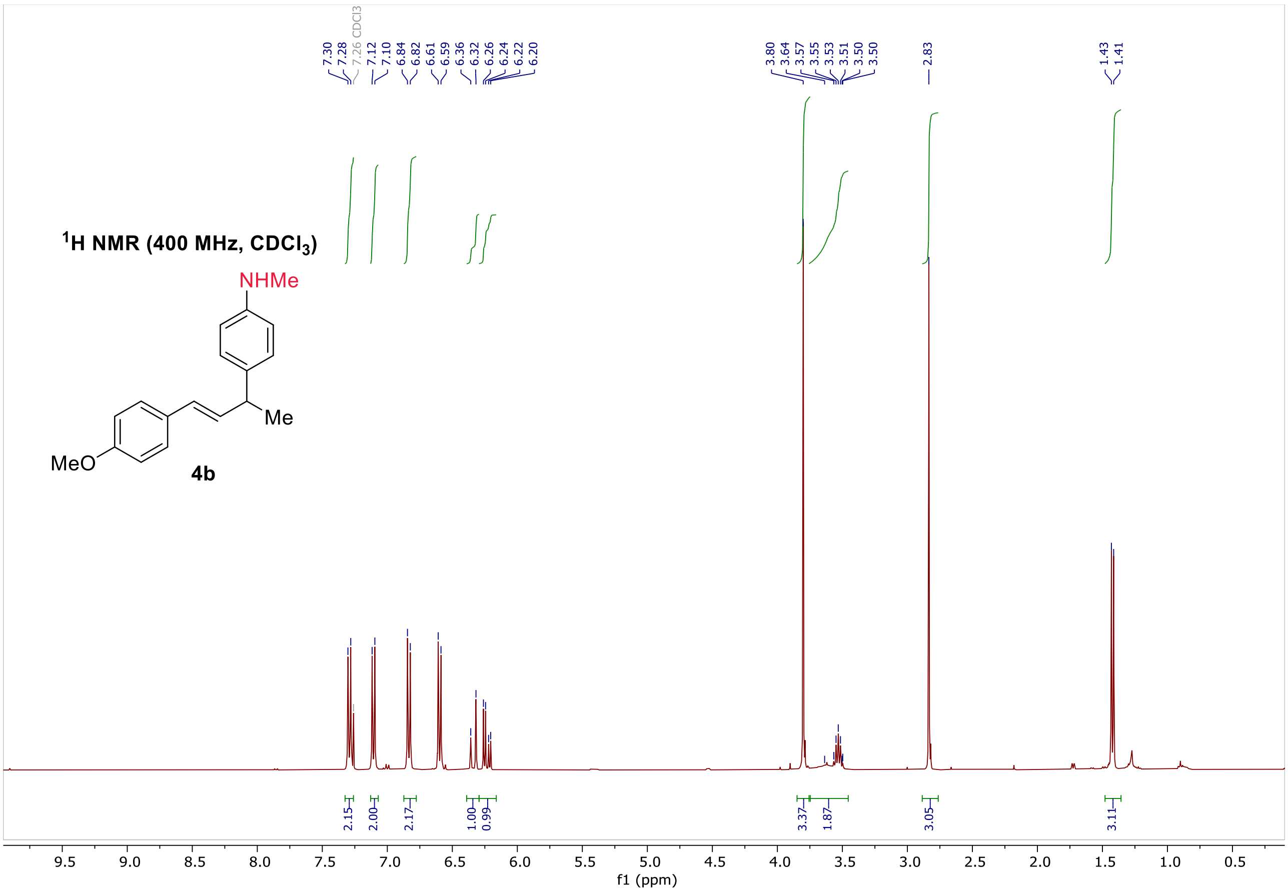
1H NMR (400 MHz, CDCl3)
NHMe
Me
MeO
4b
f1 (ppm)

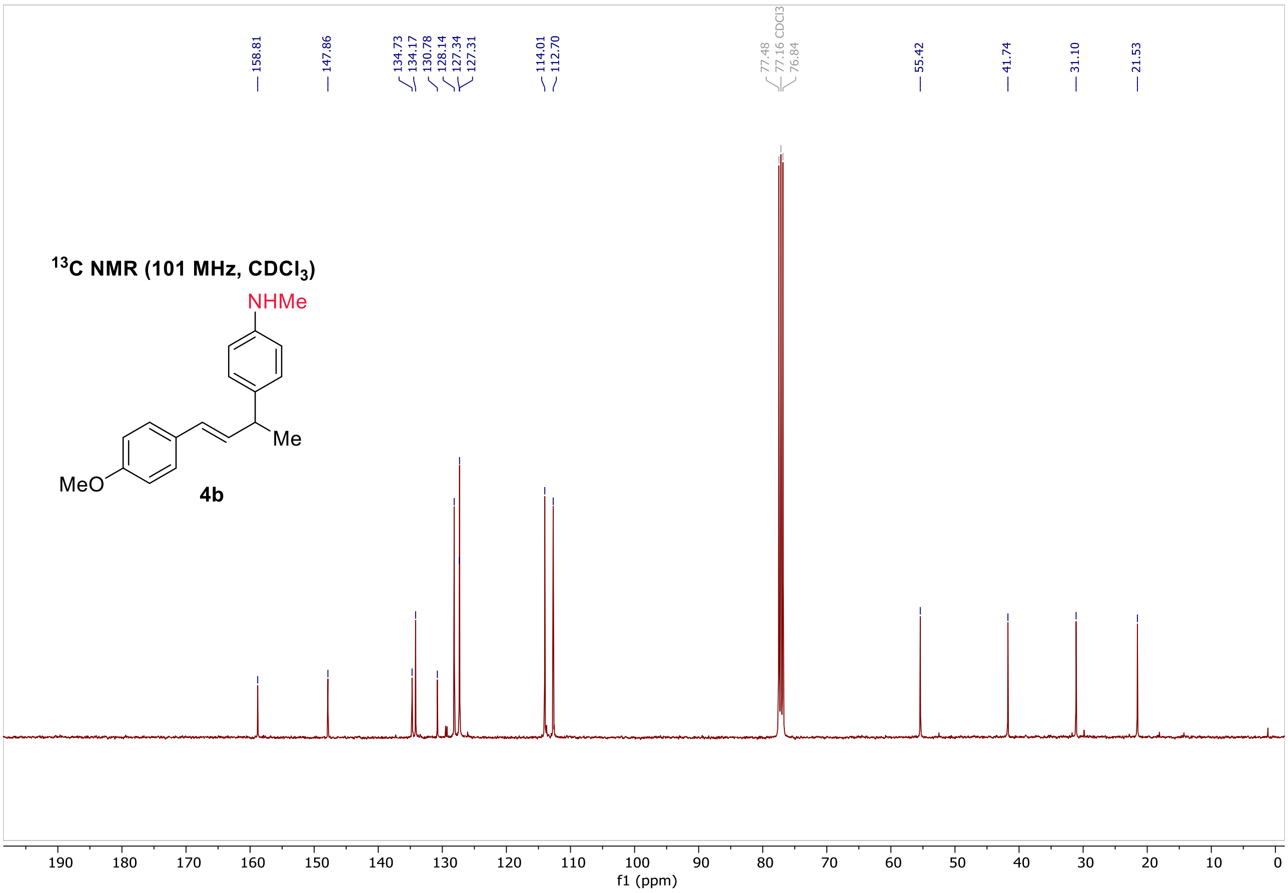
13C NMR (101 MHz, CDCl3)
NHMe
Me
MeO
4b
f1 (ppm)

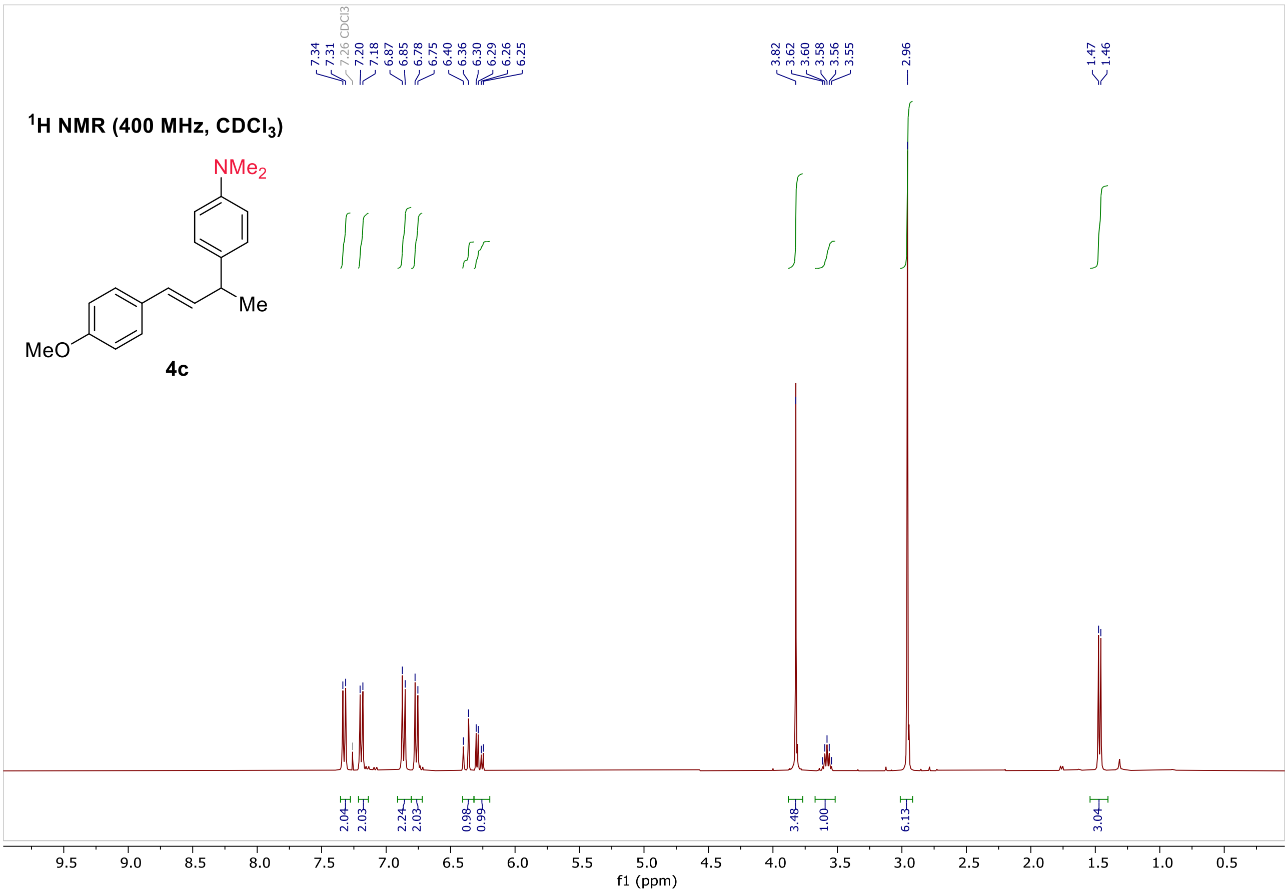
1H NMR (400 MHz, CDCl3)
NMe2
Me
MeO
4c
7.34 7.31 7.26 CDCl3 7.20 7.18 6.87 6.85 6.78 6.75 6.40 6.36 6.30 6.29 6.26 6.25
3.82 3.62 3.60 3.58 3.56 3.55
2.96
1.47 1.46
2.04 2.03 2.24 2.03 0.98 0.99 3.48 1.00 6.13 3.04
f1 (ppm)

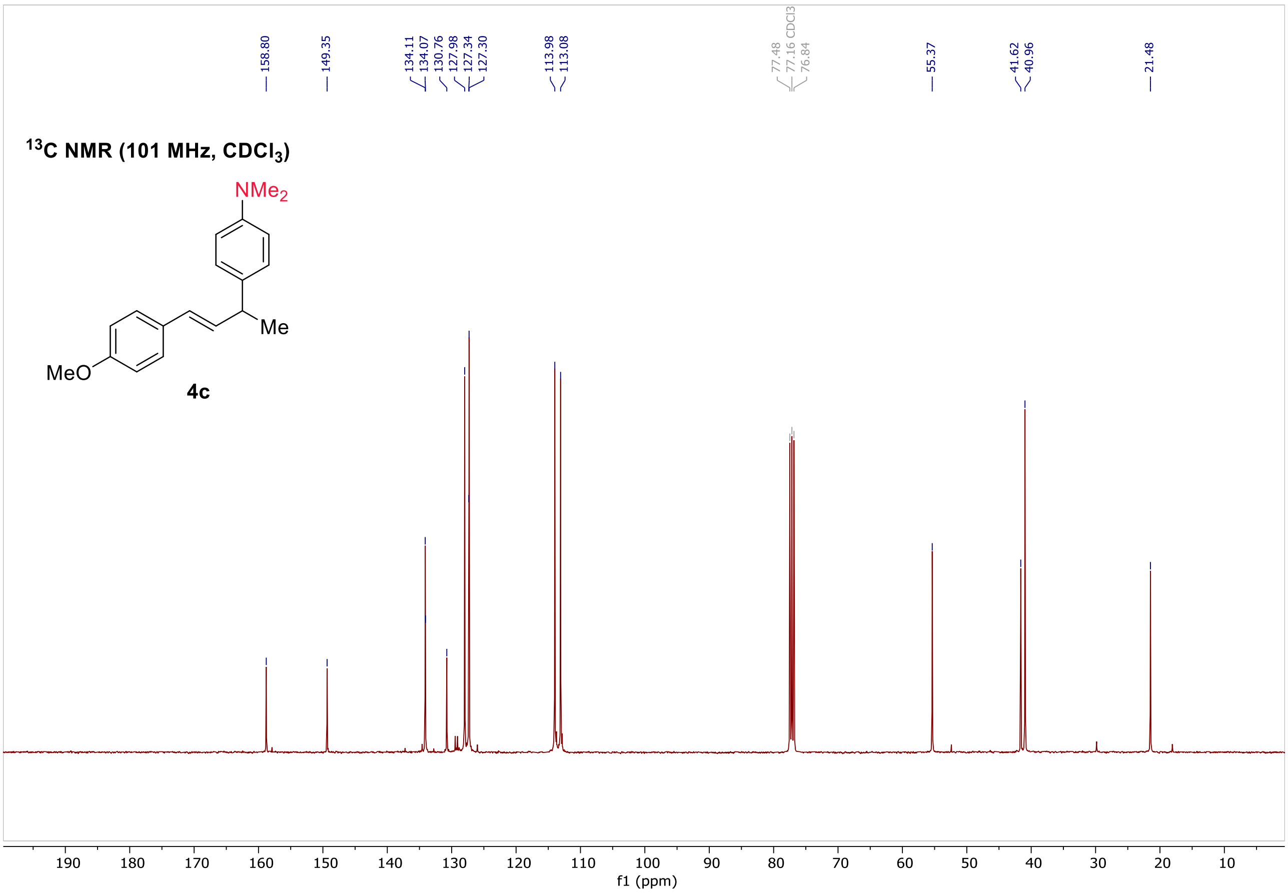
13C NMR (101 MHz, CDCl3)
NMe2
Me
MeO
4c
158.80
149.35
134.11 134.07 130.76 127.98 127.34 127.30
113.98 113.08
77.48 77.16 CDCl3 76.84
55.37
41.62 40.96
21.48
f1 (ppm)

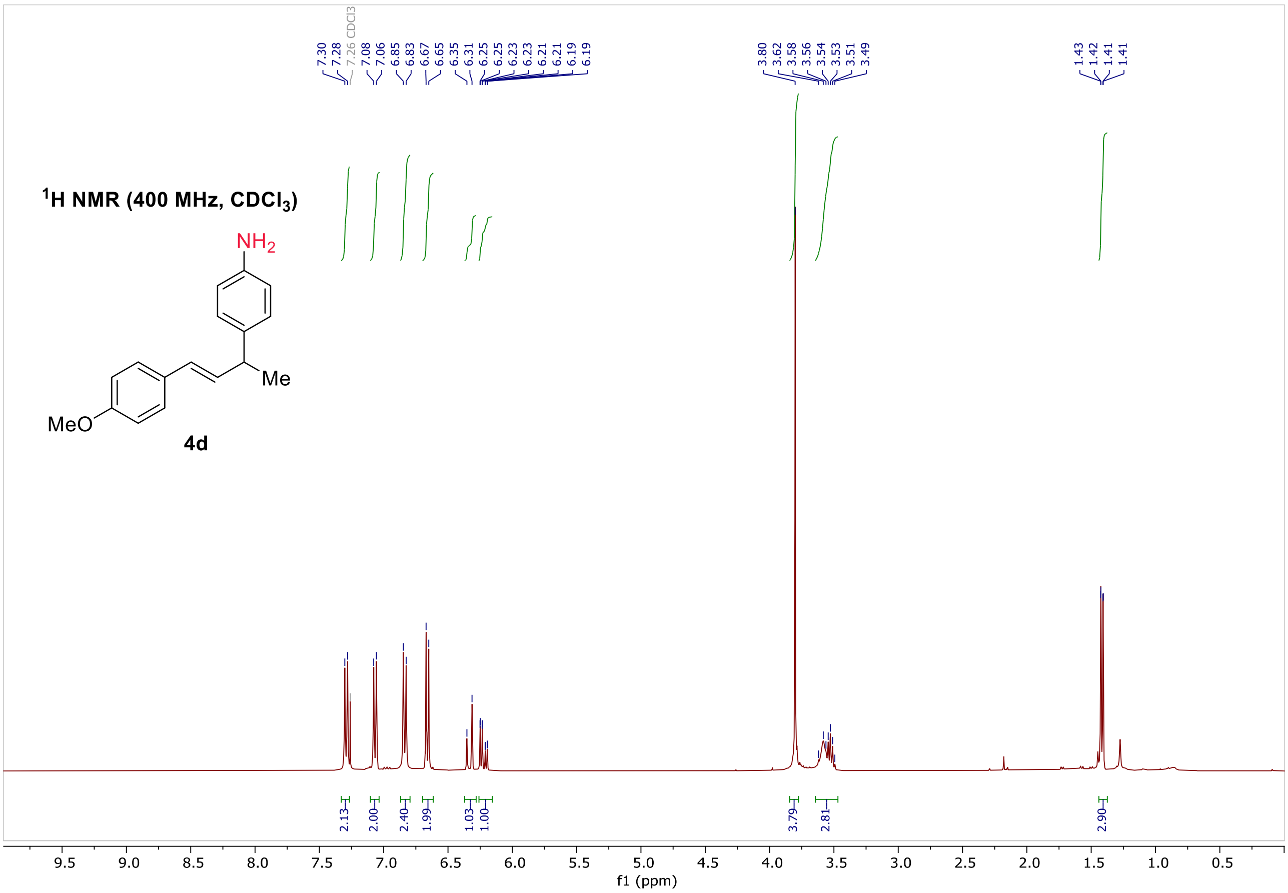
7.30
7.28
7.26 CDCl3
7.08
7.06
6.85
6.83
6.67
6.65
6.35
6.31
6.25
6.25
6.23
6.23
6.21
6.21
6.19
6.19
3.80
3.62
3.58
3.56
3.54
3.53
3.51
3.49
1.43
1.42
1.41
1.41
1H NMR (400 MHz, CDCl3)
NH2
Me
MeO
4d
2.13
2.00
2.40
1.99
1.03
1.00
3.79
2.81
2.90
f1 (ppm)

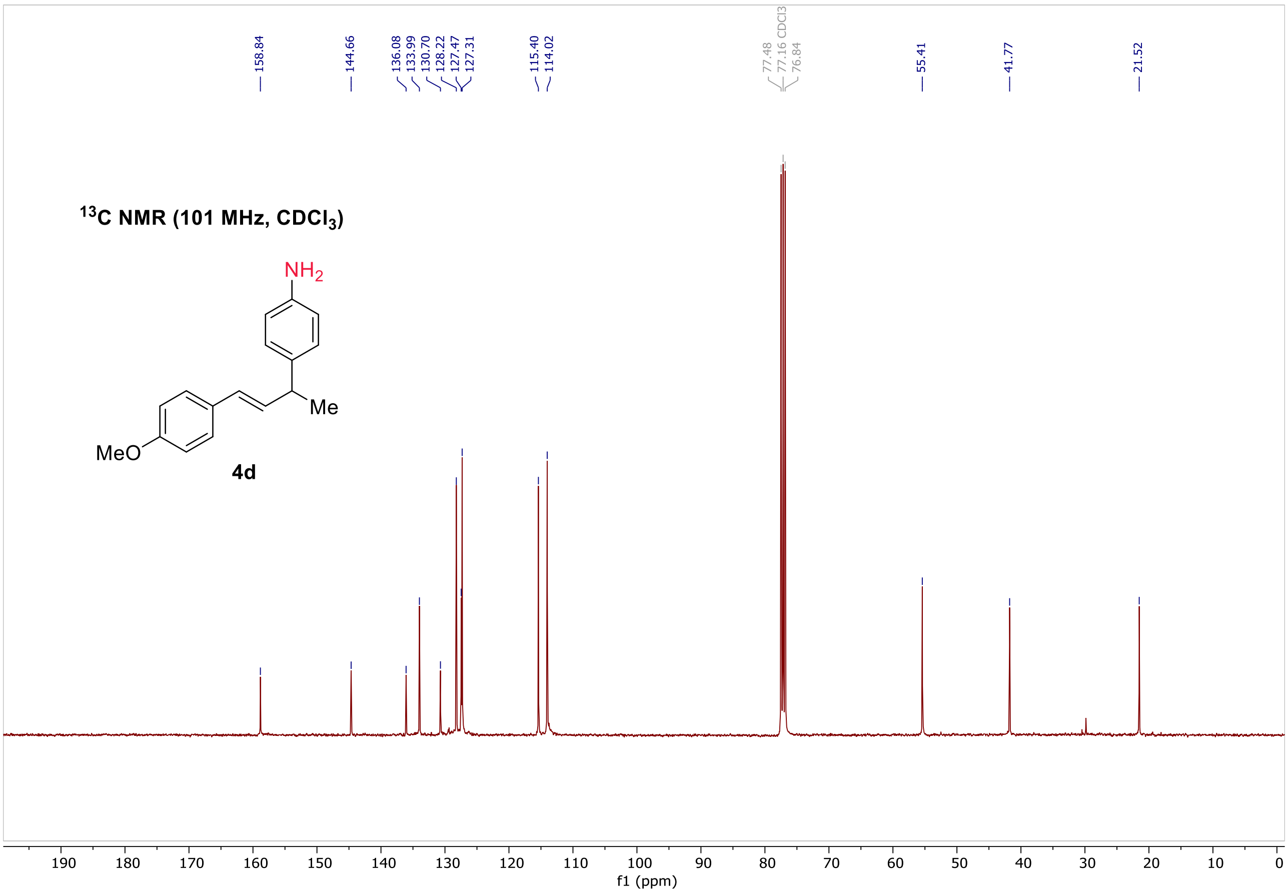
158.84
144.66
136.08
133.99
130.70
128.22
127.47
127.31
115.40
114.02
77.48
77.16 CDCl3
76.84
55.41
41.77
21.52
13C NMR (101 MHz, CDCl3)
NH2
Me
MeO
4d
f1 (ppm)

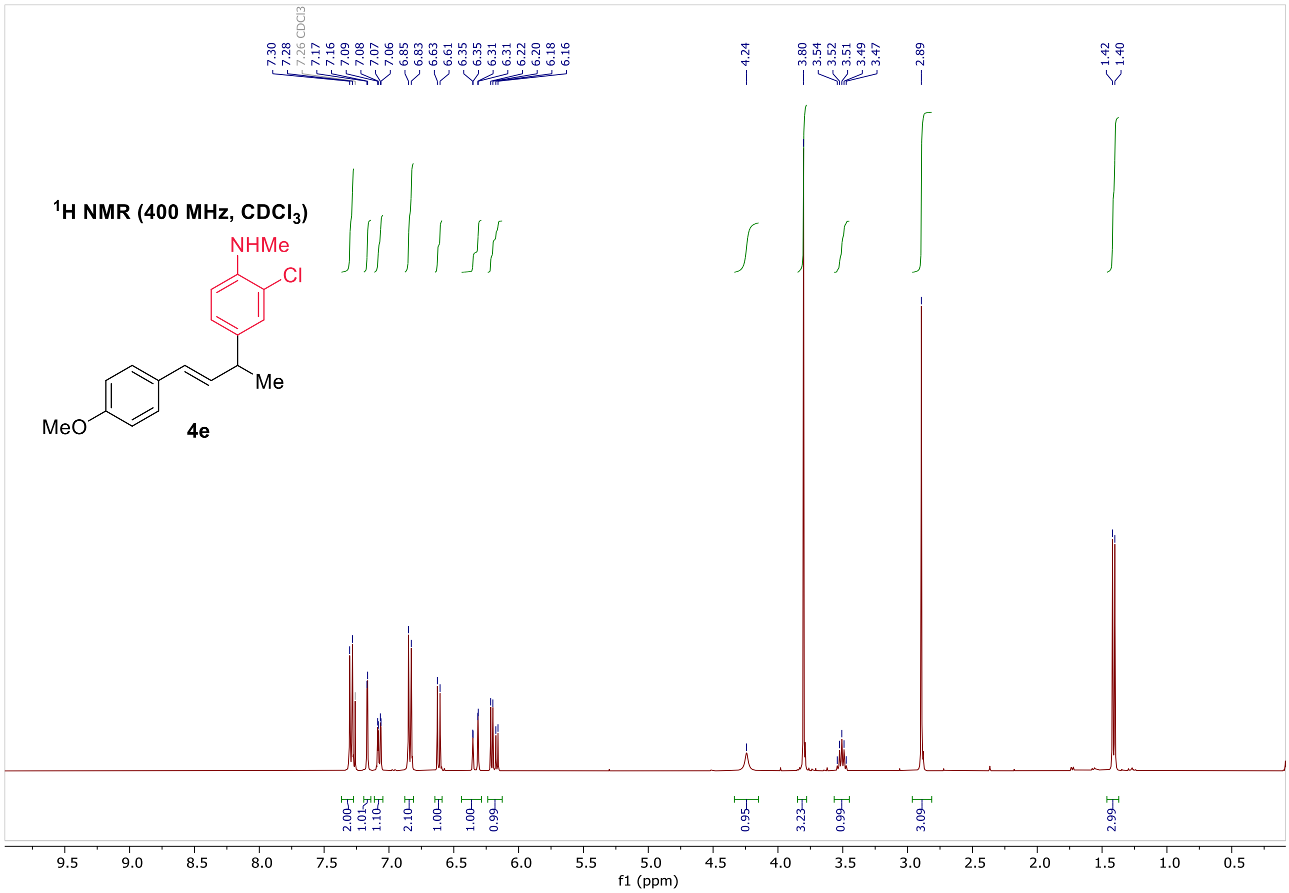

7.30
7.28
7.26 CDCl3
7.17
7.16
7.09
7.08
7.07
7.06
6.85
6.83
6.63
6.61
6.35
6.35
6.31
6.31
6.22
6.20
6.18
6.16
4.24
3.80
3.54
3.52
3.51
3.49
3.47
2.89
1.42
1.40
1H NMR (400 MHz, CDCl3)
NHMe
Cl
Me
MeO
4e
2.00
1.01
1.10
2.10
1.00
1.00
0.99
0.95
3.23
0.99
3.09
2.99
f1 (ppm)

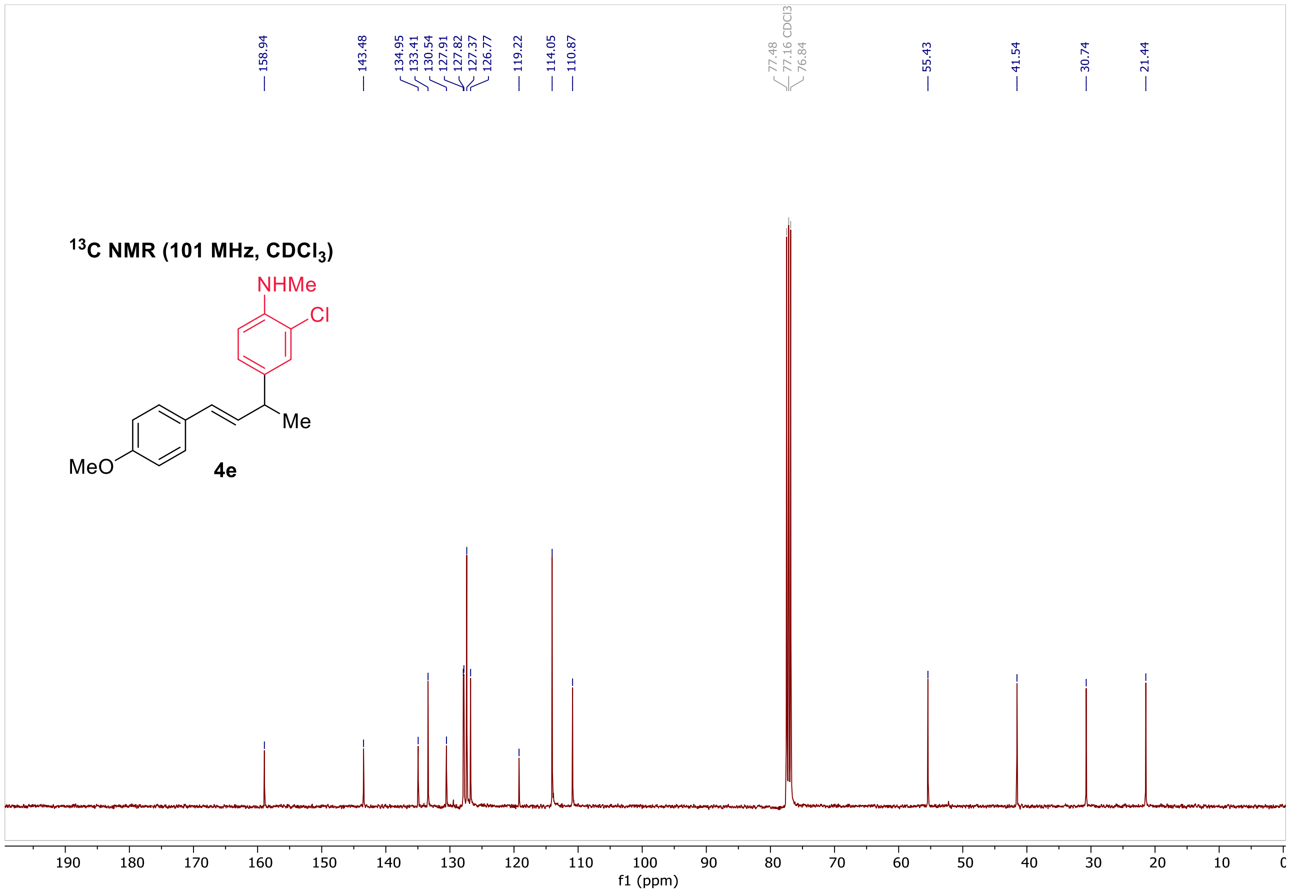

158.94
143.48
134.95
133.41
130.54
127.91
127.82
127.37
126.77
119.22
114.05
110.87
77.48
77.16 CDCl3
76.84
55.43
41.54
30.74
21.44
13C NMR (101 MHz, CDCl3)
NHMe
Cl
Me
MeO
4e
f1 (ppm)

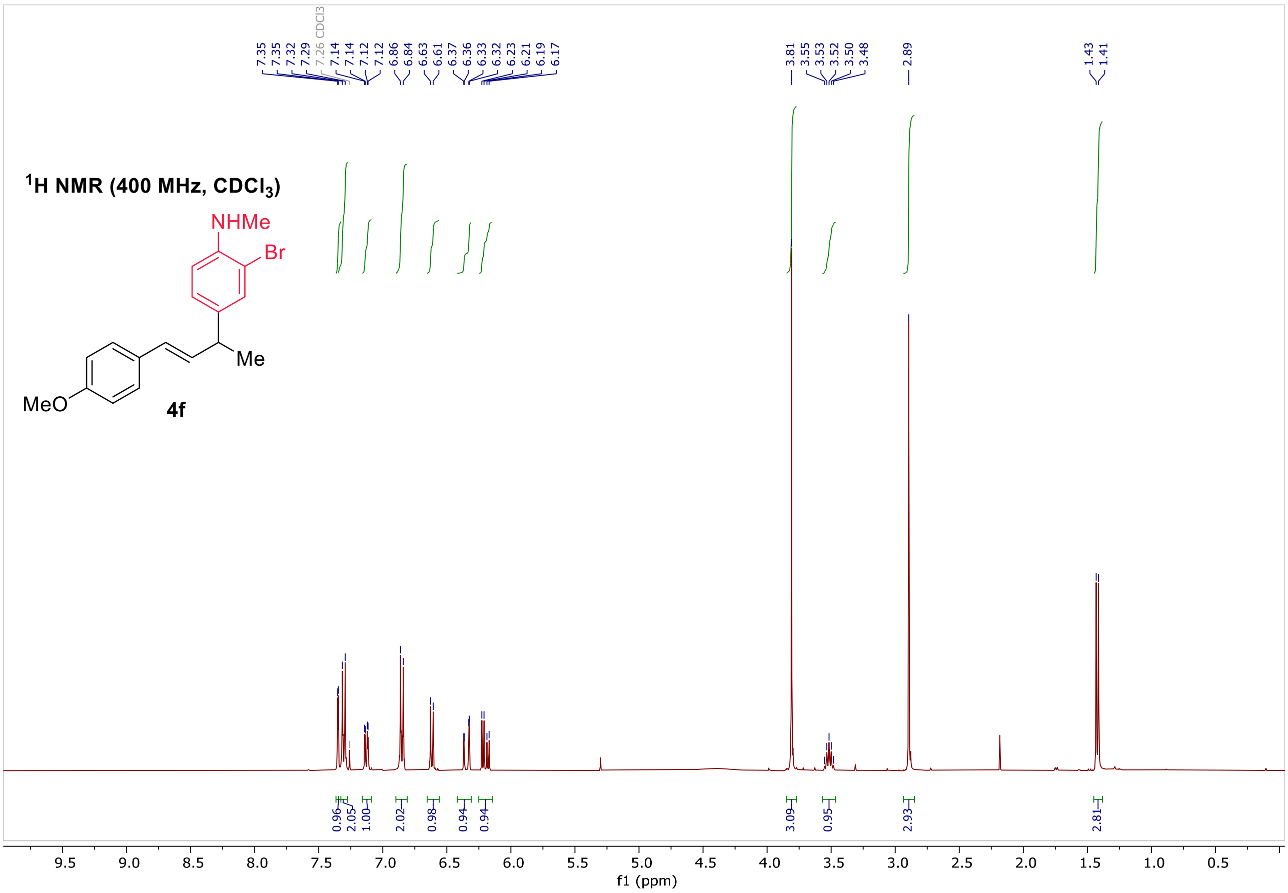
7.35
7.35
7.32
7.29
7.26 CDCl3
7.14
7.14
7.12
7.12
6.86
6.84
6.63
6.61
6.37
6.36
6.33
6.32
6.23
6.21
6.19
6.17
3.81
3.55
3.53
3.52
3.50
3.48
2.89
1.43
1.41
1H NMR (400 MHz, CDCl3)
NHMe
Br
Me
MeO
4f
0.96
2.05
1.00
2.02
0.98
0.94
0.94
3.09
0.95
2.93
2.81
9.5
9.0
8.5
8.0
7.5
7.0
6.5
6.0
5.5
5.0
4.5
4.0
3.5
3.0
2.5
2.0
1.5
1.0
0.5
f1 (ppm)

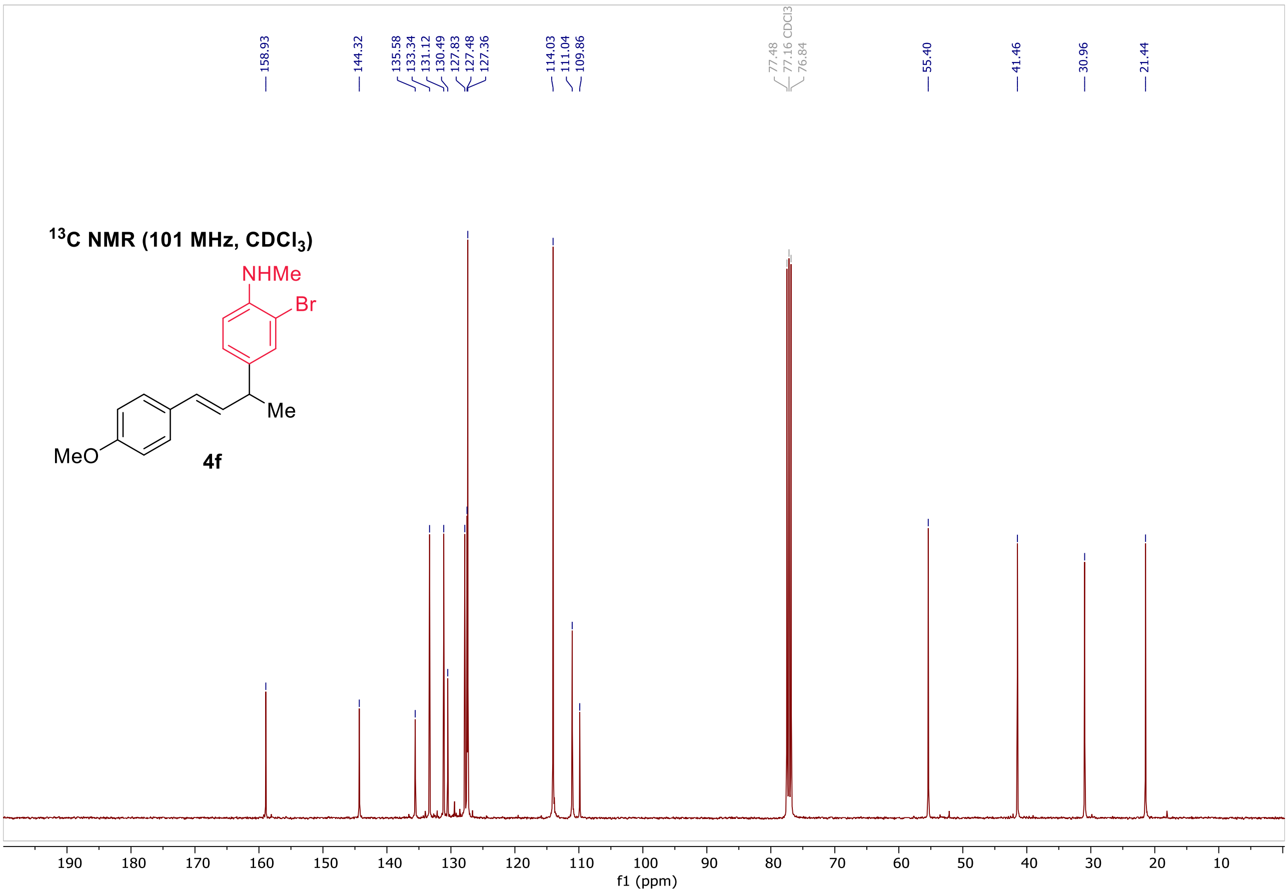
158.93
144.32
135.58
133.34
131.12
130.49
127.83
127.48
127.36
114.03
111.04
109.86
77.48
77.16 CDCl3
76.84
55.40
41.46
30.96
21.44
13C NMR (101 MHz, CDCl3)
NHMe
Br
Me
MeO
4f
190
180
170
160
150
140
130
120
110
100
90
80
70
60
50
40
30
20
10
f1 (ppm)

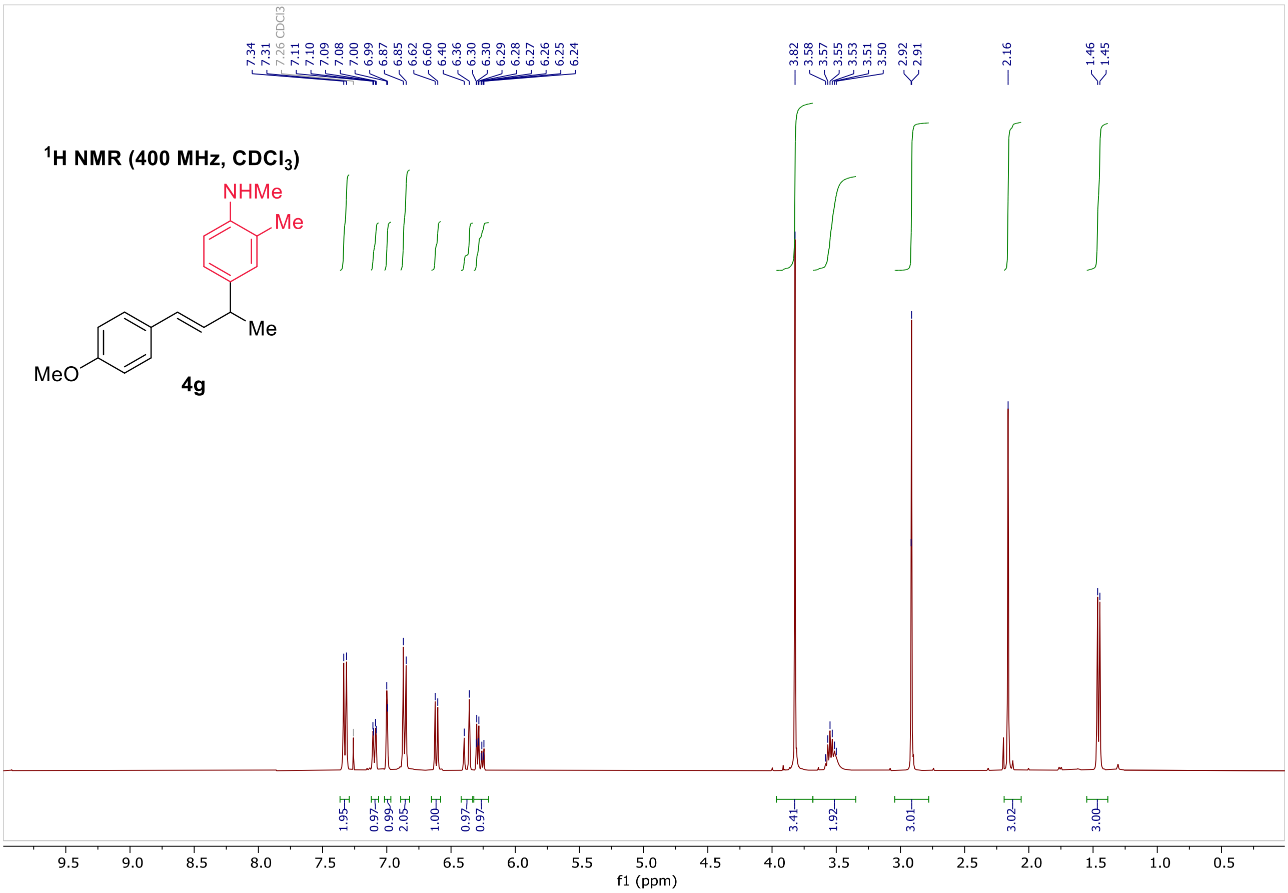
1H NMR (400 MHz, CDCl3)
NHMe
Me
Me
MeO
4g
7.34 7.31 7.26 CDCl3 7.11 7.10 7.09 7.08 7.00 6.99 6.87 6.85 6.62 6.60 6.40 6.36 6.30 6.30 6.29 6.28 6.27 6.26 6.25 6.24
3.82 3.58 3.57 3.55 3.53 3.51 3.50 2.92 2.91
2.16
1.46 1.45
1.95 0.97 0.99 2.05 1.00 0.97 0.97 3.41 1.92 3.01 3.02 3.00
9.5 9.0 8.5 8.0 7.5 7.0 6.5 6.0 5.5 5.0 4.5 4.0 3.5 3.0 2.5 2.0 1.5 1.0 0.5
f1 (ppm)

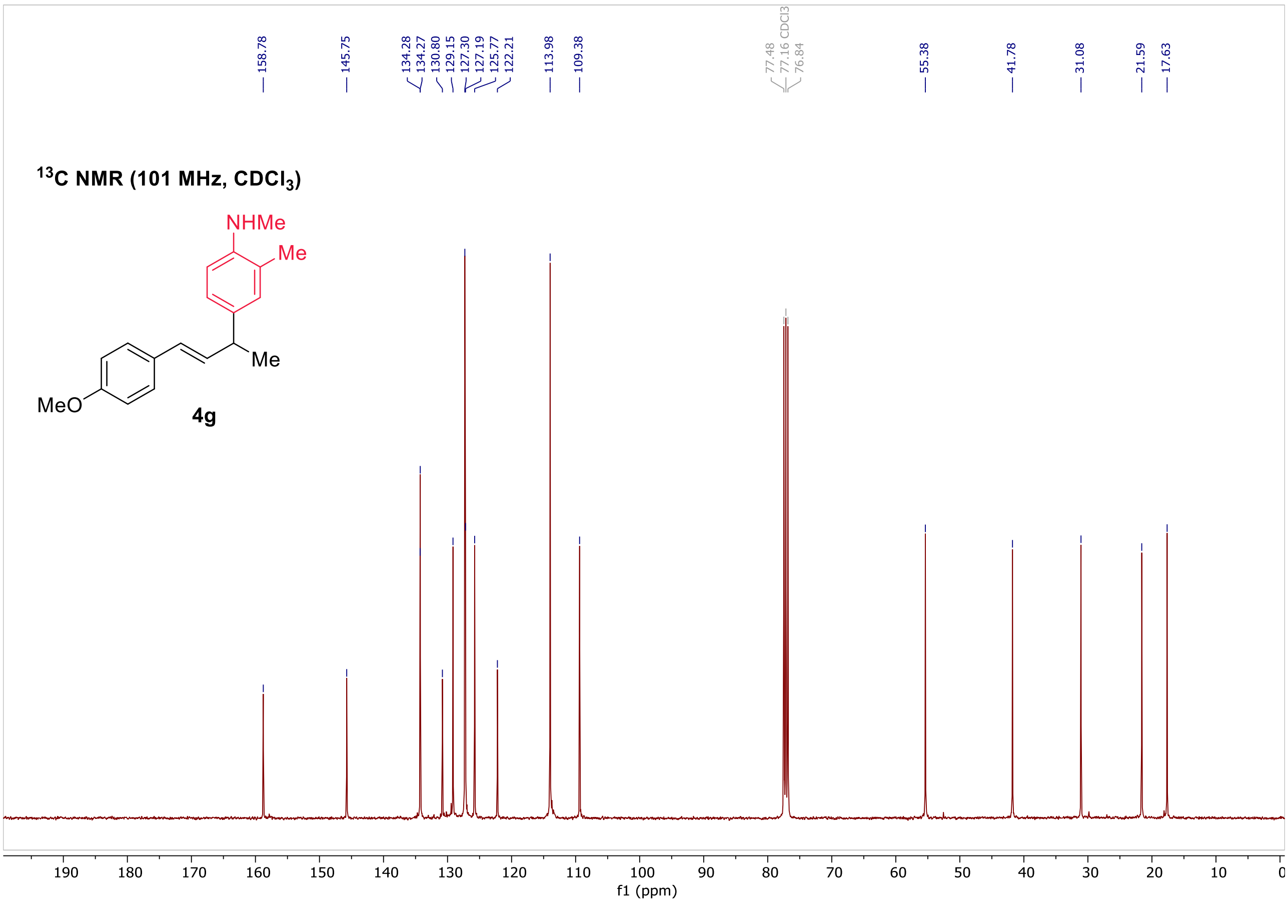
13C NMR (101 MHz, CDCl3)
NHMe
Me
Me
MeO
4g
158.78
145.75
134.28 134.27 130.80 129.15 127.30 127.19 125.77 122.21
113.98 109.38
77.48 77.16 CDCl3 76.84
55.38
41.78
31.08
21.59 17.63
190 180 170 160 150 140 130 120 110 100 90 80 70 60 50 40 30 20 10 0
f1 (ppm)

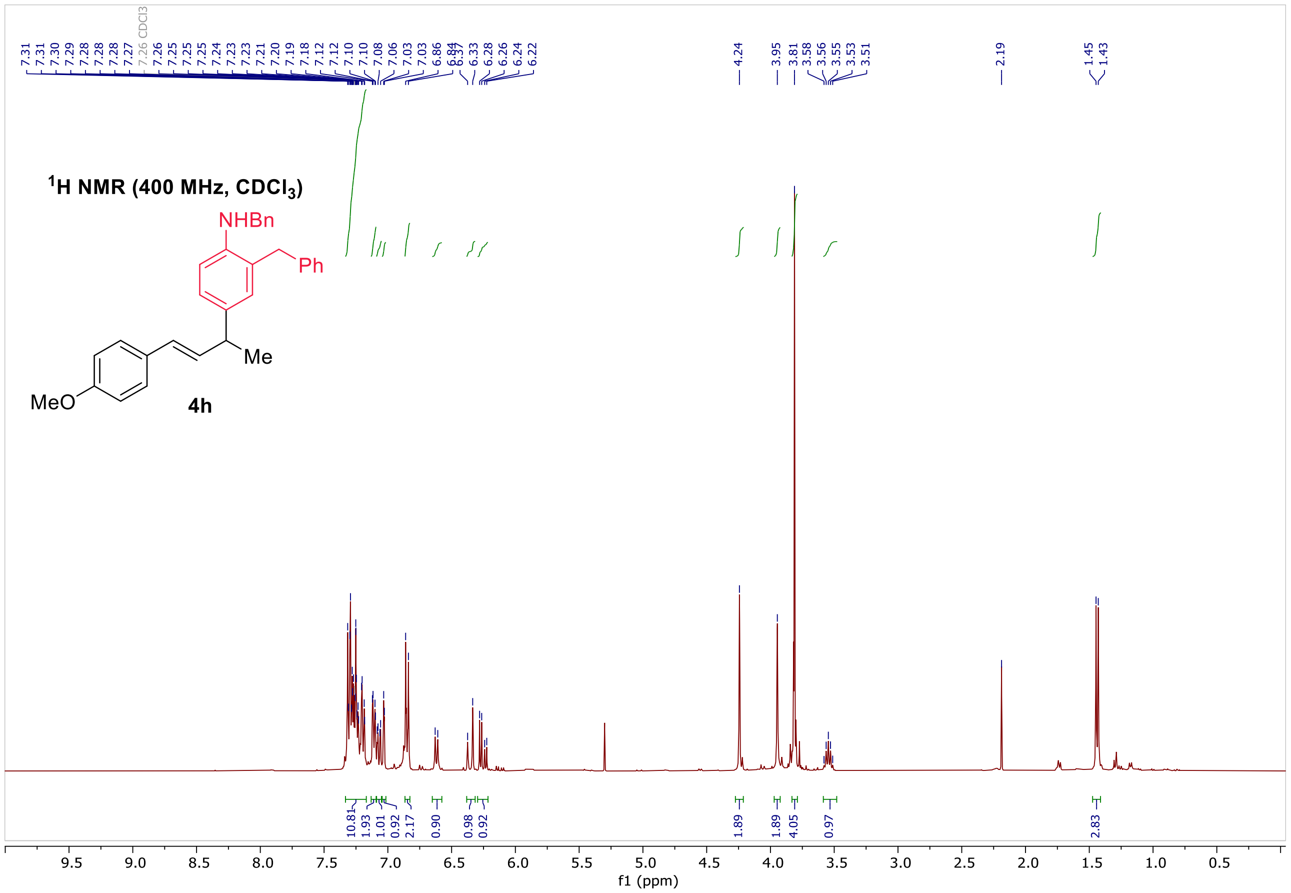
1H NMR (400 MHz, CDCl3)
NHBn
Ph
Me
MeO
4h
7.31 7.31 7.30 7.29 7.28 7.28 7.28 7.27 7.26 CDCl3 7.26 7.25 7.25 7.25 7.24 7.23 7.23 7.21 7.20 7.19 7.18 7.12 7.12 7.10 7.10 7.08 7.06 7.03 7.03 6.86 6.84 6.37 6.33 6.28 6.26 6.24 6.22
4.24 3.95 3.81 3.58 3.56 3.55 3.53 3.51 2.19 1.45 1.43
10.81 1.93 1.01 0.92 2.17 0.90 0.98 0.92 1.89 1.89 4.05 0.97 2.83
9.5 9.0 8.5 8.0 7.5 7.0 6.5 6.0 5.5 5.0 4.5 4.0 3.5 3.0 2.5 2.0 1.5 1.0 0.5
f1 (ppm)

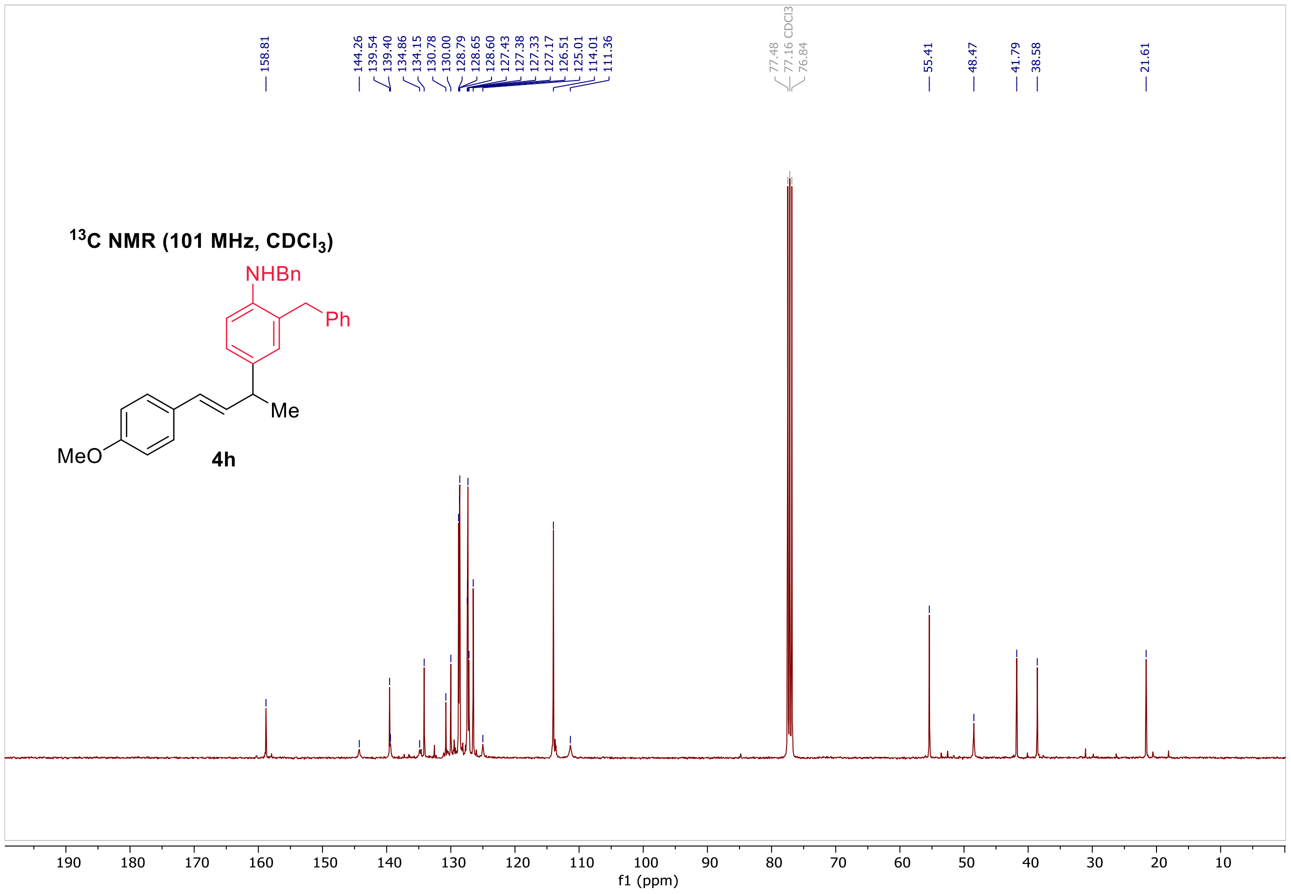
13C NMR (101 MHz, CDCl3)
NHBn
Ph
Me
MeO
4h
158.81 144.26 139.54 139.40 134.86 134.15 130.78 130.00 128.79 128.65 128.60 127.43 127.38 127.33 127.17 126.51 125.01 114.01 111.36
77.48 77.16 CDCl3 76.84
55.41 48.47 41.79 38.58 21.61
190 180 170 160 150 140 130 120 110 100 90 80 70 60 50 40 30 20 10
f1 (ppm)

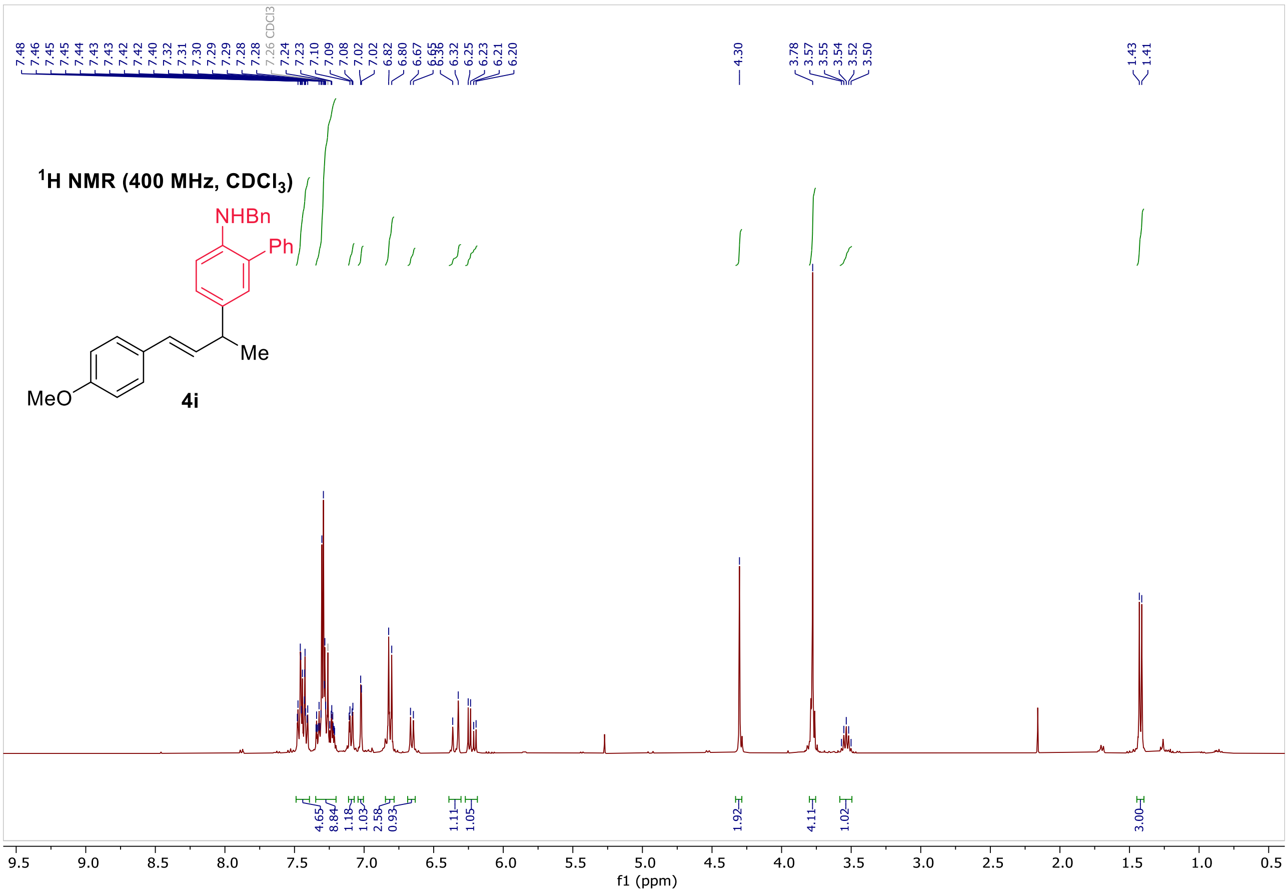

7.48 7.46 7.45 7.45 7.44 7.43 7.43 7.42 7.42 7.40 7.32 7.31 7.30 7.29 7.29 7.28 7.28 7.26 CDCl3 7.24 7.23 7.10 7.09 7.08 7.02 7.02 6.82 6.80 6.67 6.65 6.36 6.32 6.25 6.23 6.21 6.20
4.30
3.78 3.57 3.55 3.54 3.52 3.50
1.43 1.41
1H NMR (400 MHz, CDCl3)
NHBn
Ph
Me
MeO
4i
4.65 8.84 1.18 1.03 2.58 0.93 1.11 1.05 1.92 4.11 1.02 3.00
9.5 9.0 8.5 8.0 7.5 7.0 6.5 6.0 5.5 5.0 4.5 4.0 3.5 3.0 2.5 2.0 1.5 1.0 0.5
f1 (ppm)

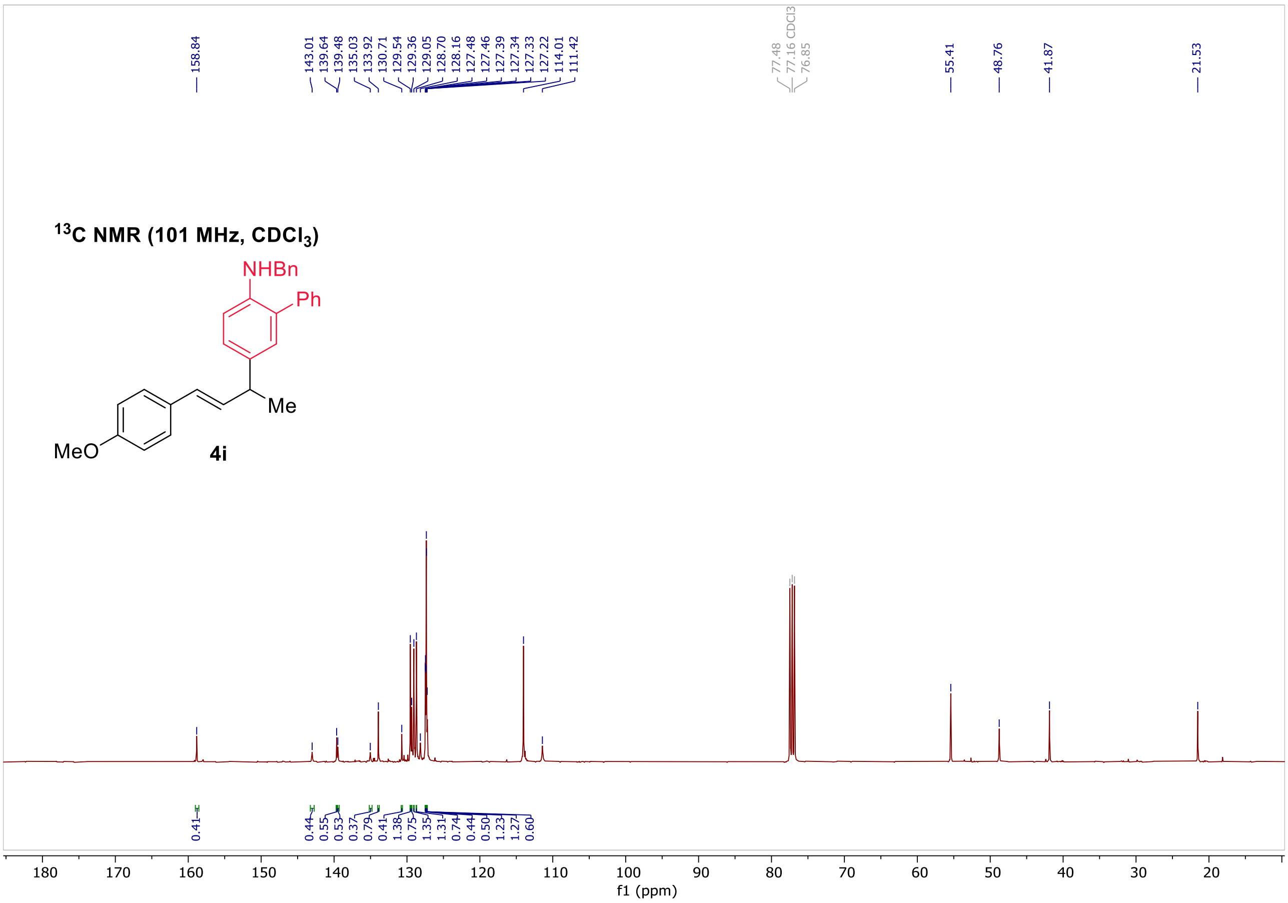

158.84
143.01 139.64 139.48 135.03 133.92 130.71 129.54 129.36 129.05 128.70 128.16 127.48 127.46 127.39 127.34 127.33 127.22 114.01 111.42
77.48 77.16 CDCl3 76.85
55.41
48.76
41.87
21.53
13C NMR (101 MHz, CDCl3)
NHBn
Ph
Me
MeO
4i
0.41 0.44 0.55 0.53 0.37 0.79 0.41 1.38 0.75 1.35 1.31 0.74 0.44 0.50 1.23 1.27 0.60
180 170 160 150 140 130 120 110 100 90 80 70 60 50 40 30 20
f1 (ppm)

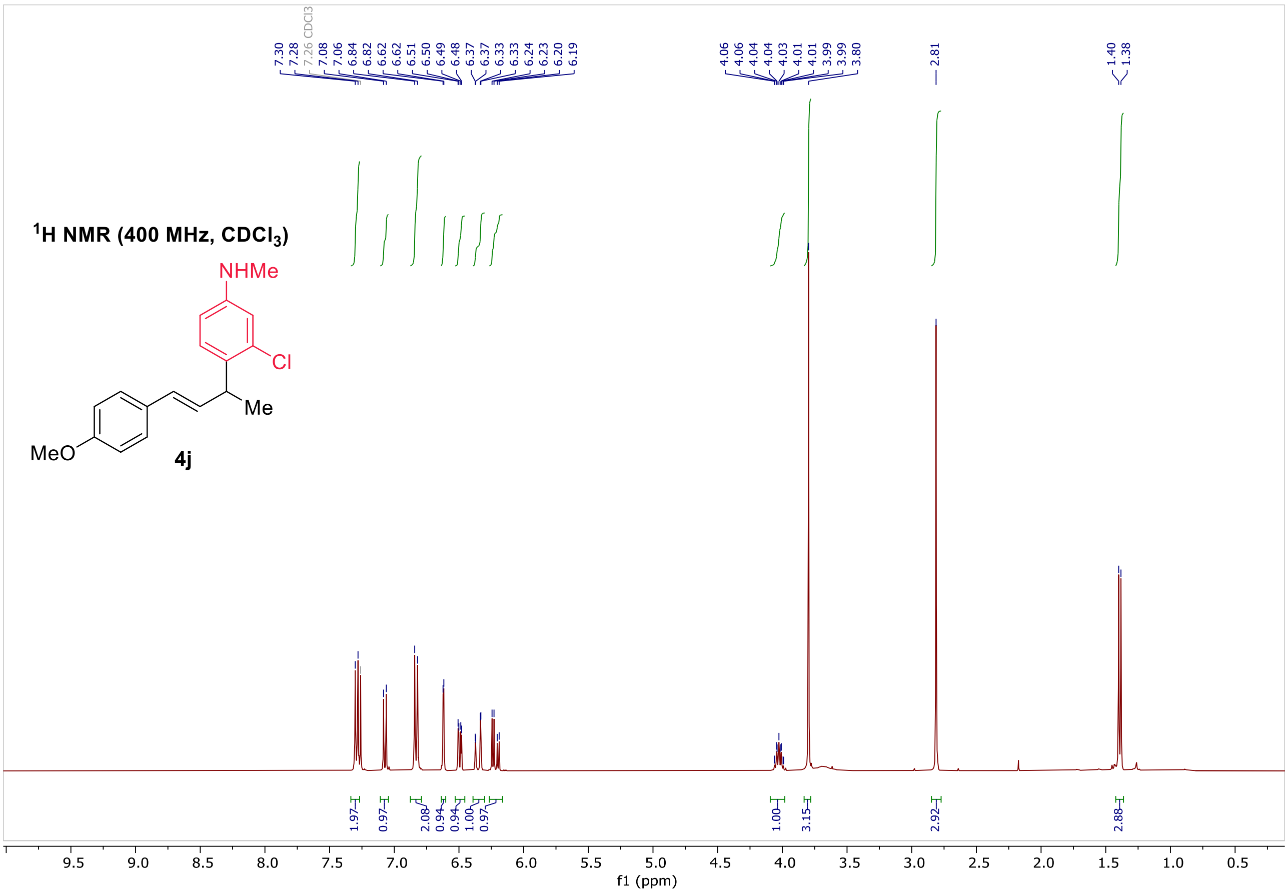
7.30 7.28 7.26 CDCl3 7.08 7.06 6.84 6.82 6.62 6.62 6.51 6.50 6.49 6.48 6.37 6.37 6.33 6.33 6.24 6.23 6.20 6.19
4.06 4.06 4.04 4.04 4.03 4.01 4.01 3.99 3.99 3.80
2.81
1.40 1.38
1H NMR (400 MHz, CDCl3)
NHMe
Cl
Me
MeO
4j
1.97 0.97 2.08 0.94 0.94 1.00 0.97 1.00 3.15 2.92 2.88
9.5 9.0 8.5 8.0 7.5 7.0 6.5 6.0 5.5 5.0 4.5 4.0 3.5 3.0 2.5 2.0 1.5 1.0 0.5
f1 (ppm)

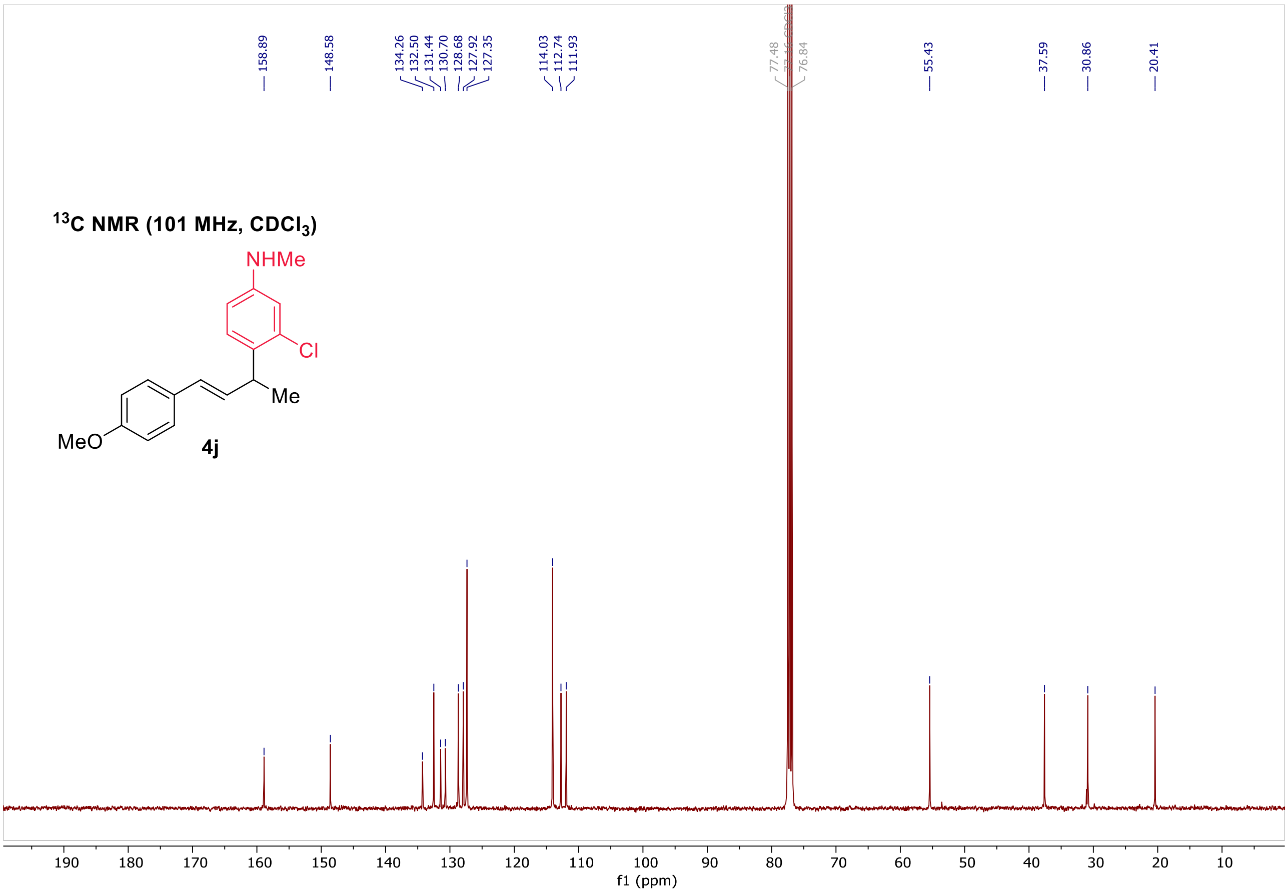
158.89
148.58
134.26 132.50 131.44 130.70 128.68 127.92 127.35
114.03 112.74 111.93
77.48 77.16 CDCl3 76.84
55.43
37.59
30.86
20.41
13C NMR (101 MHz, CDCl3)
NHMe
Cl
Me
MeO
4j
190 180 170 160 150 140 130 120 110 100 90 80 70 60 50 40 30 20 10
f1 (ppm)

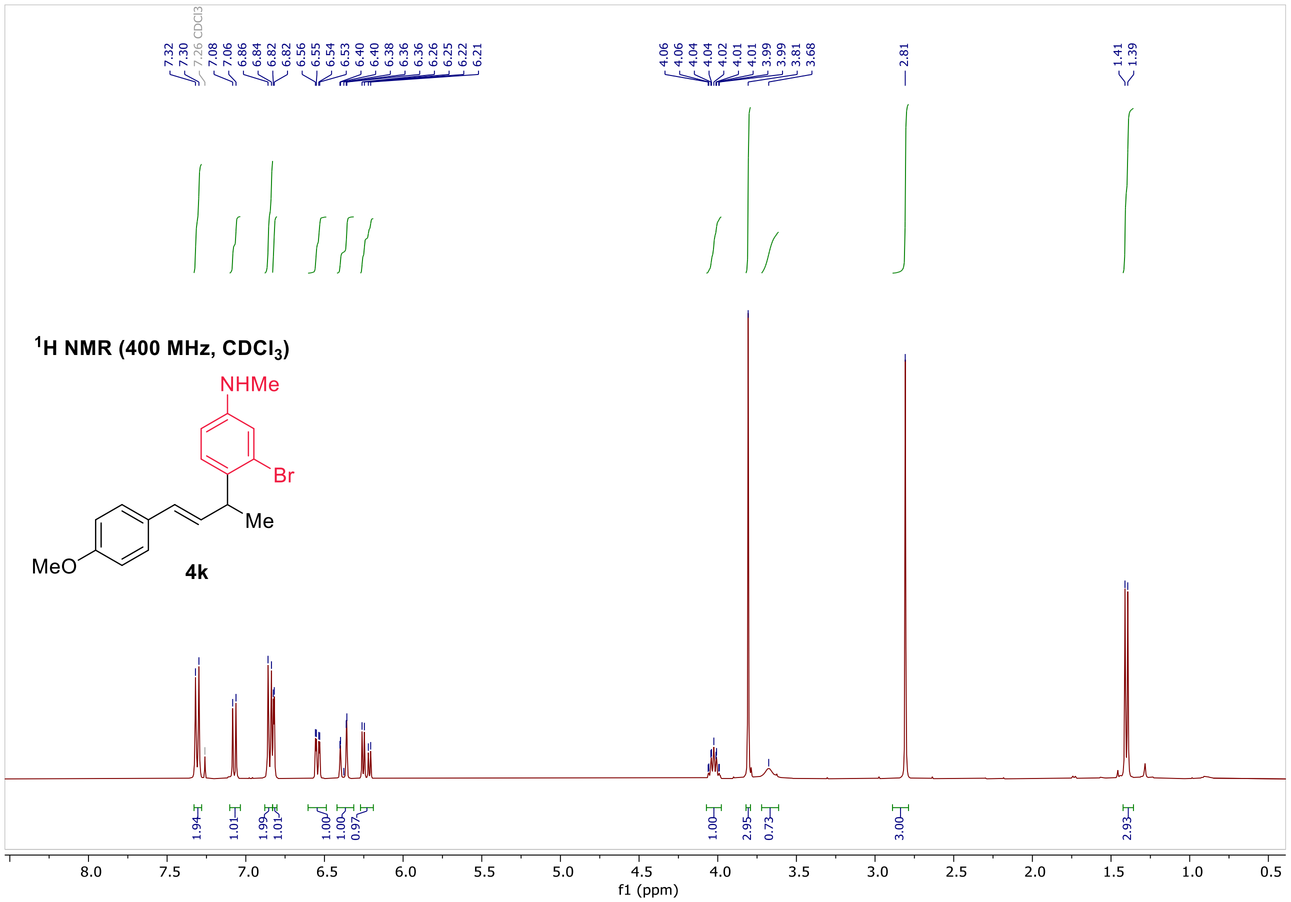

1H NMR (400 MHz, CDCl3)
NHMe
Br
Me
MeO
4k
f1 (ppm)

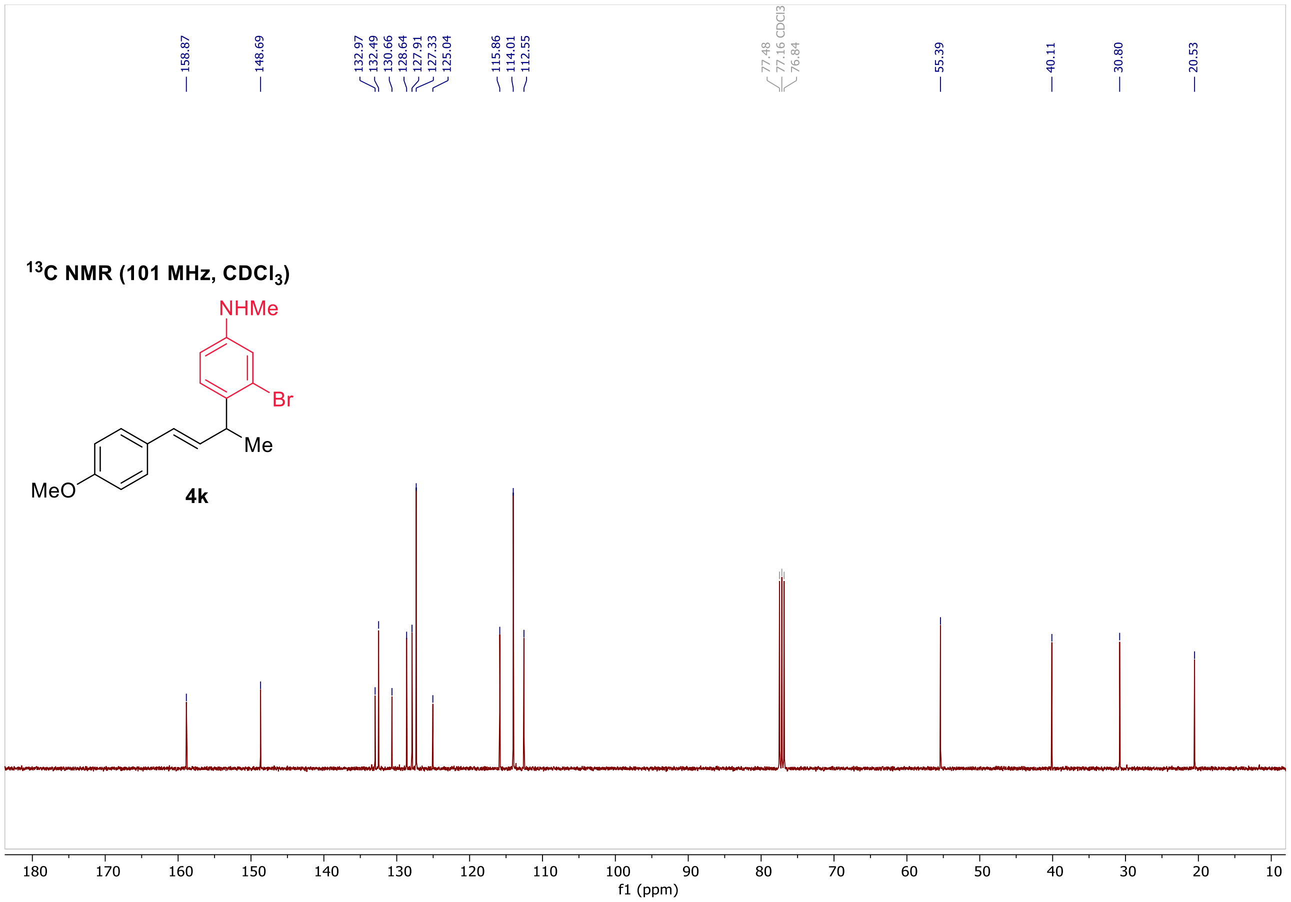

13C NMR (101 MHz, CDCl3)
NHMe
Br
Me
MeO
4k
158.87
148.69
132.97
132.49
130.66
128.64
127.91
127.33
125.04
115.86
114.01
112.55
77.48
77.16 CDCl3
76.84
55.39
40.11
30.80
20.53
f1 (ppm)

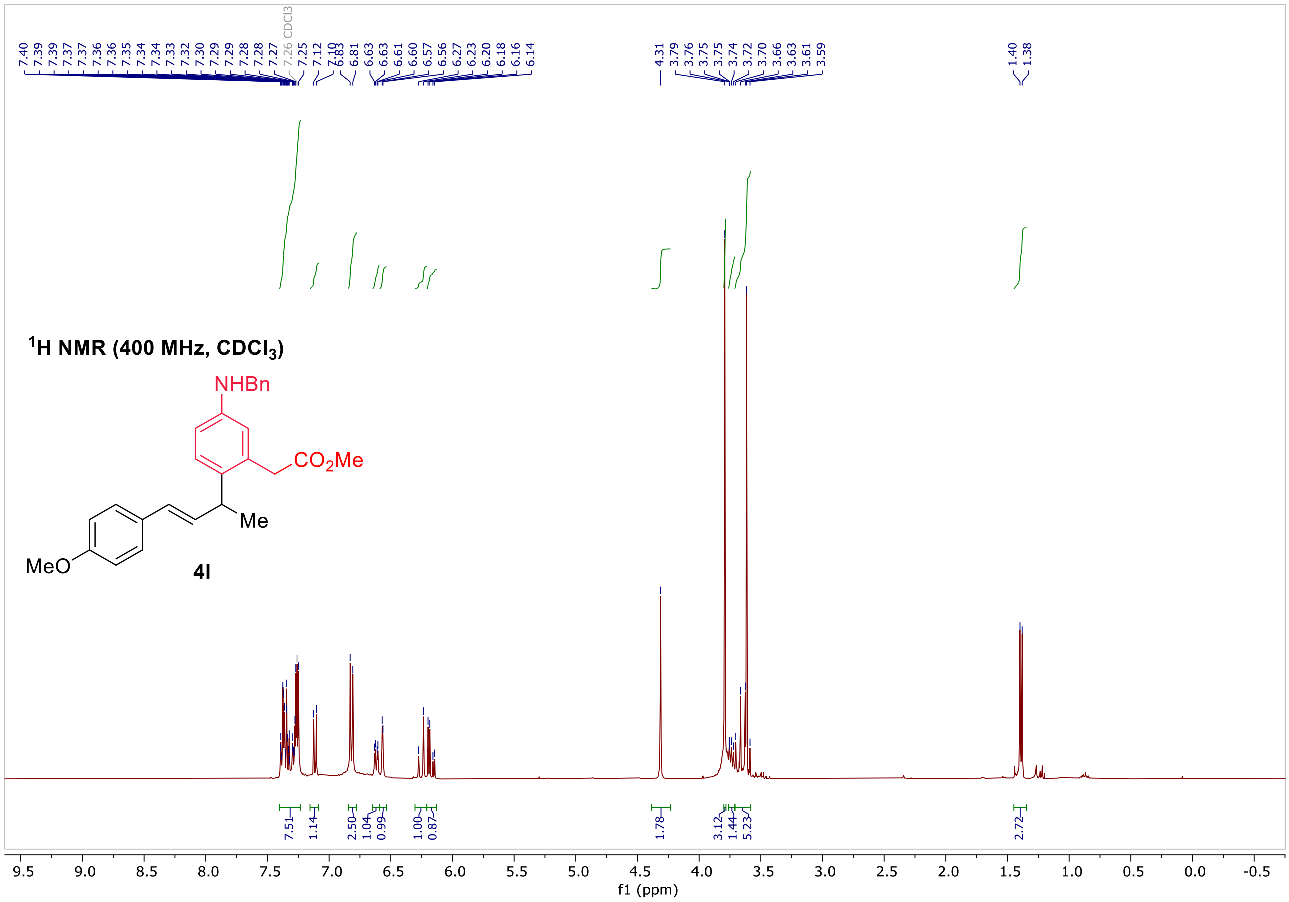

1H NMR (400 MHz, CDCl3)
NHBn
CO2Me
Me
MeO
4l
f1 (ppm)

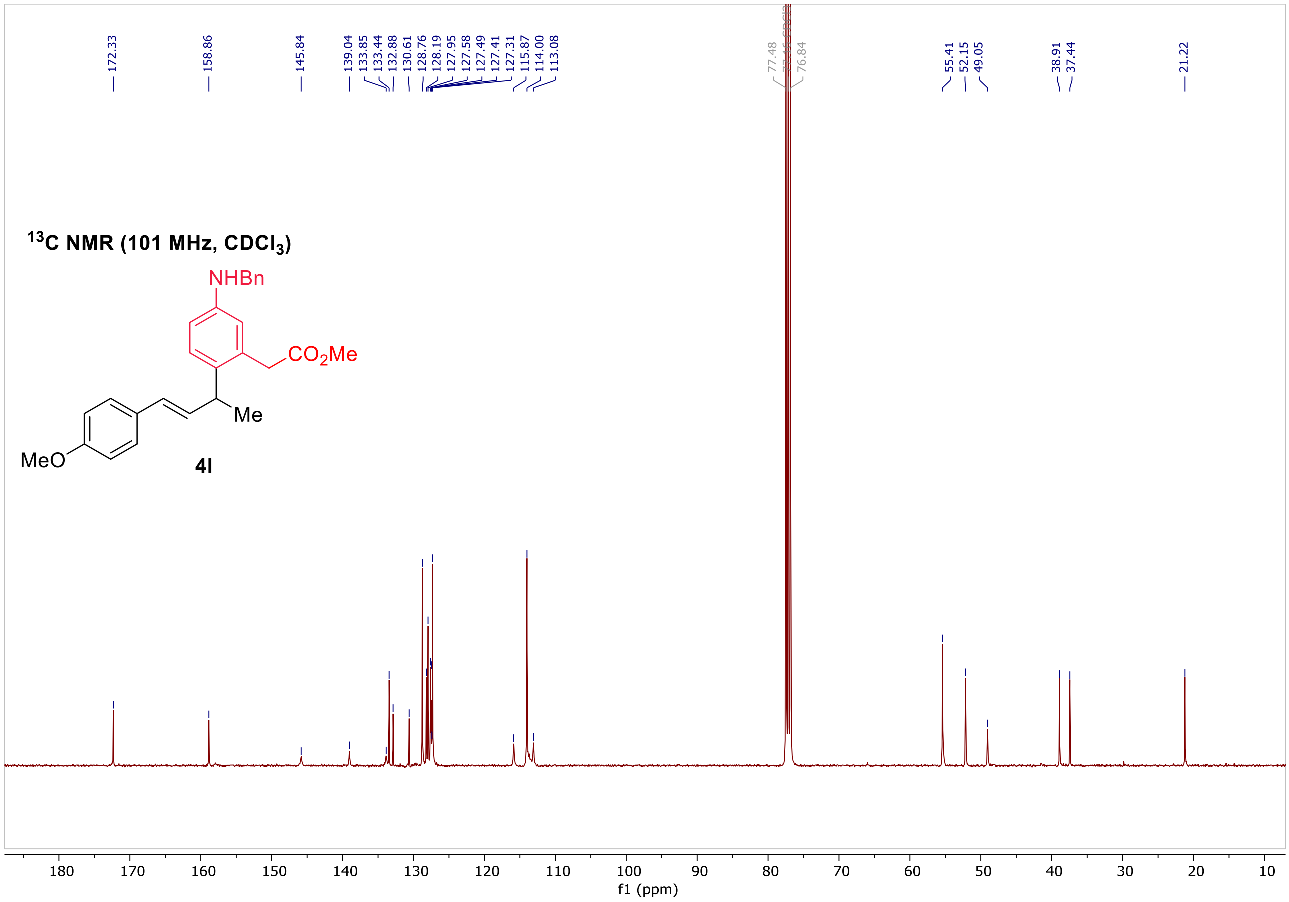

13C NMR (101 MHz, CDCl3)
NHBn
CO2Me
Me
MeO
4l
f1 (ppm)

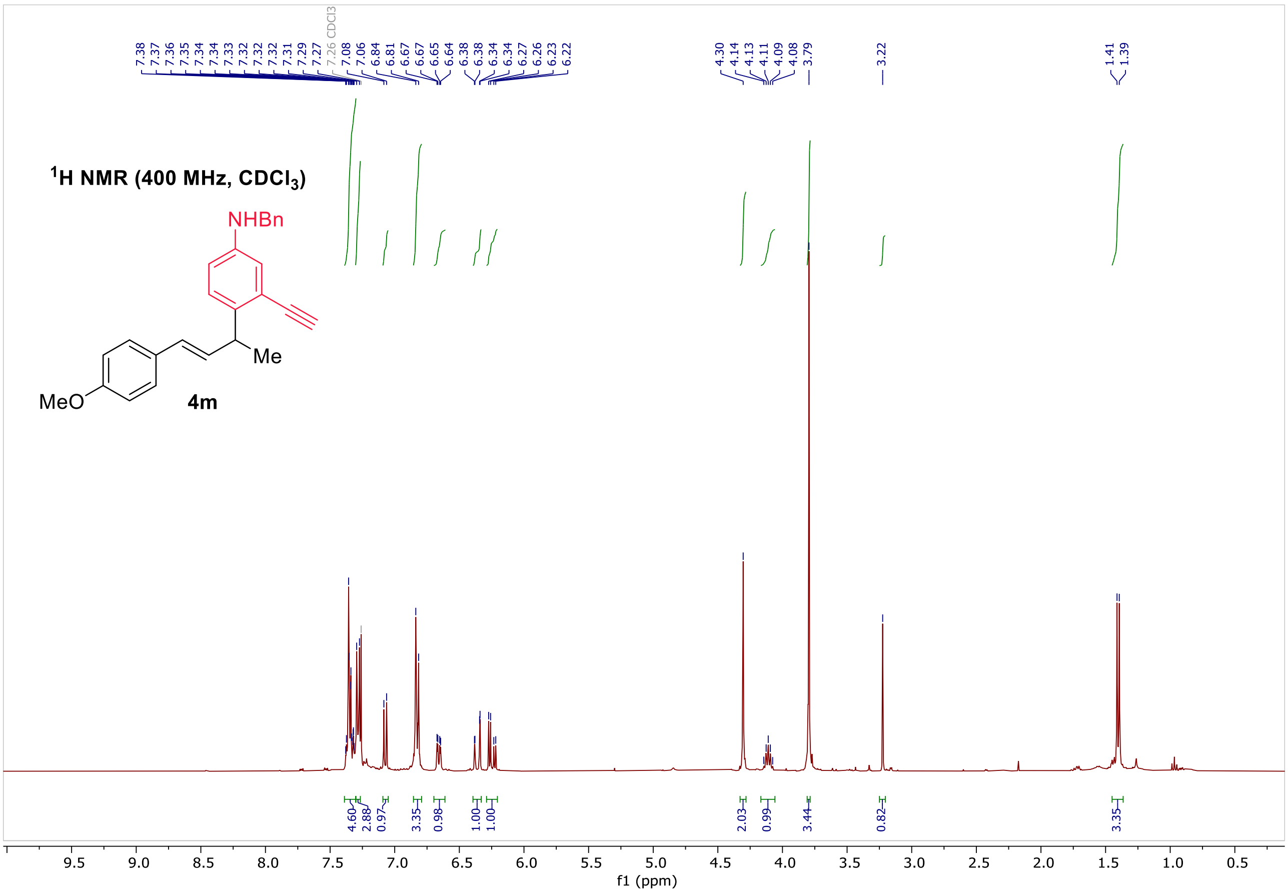

7.38
7.37
7.36
7.35
7.34
7.34
7.33
7.32
7.32
7.32
7.31
7.29
7.27
7.26 CDCl3
7.08
7.06
6.84
6.81
6.67
6.67
6.65
6.64
6.38
6.38
6.34
6.34
6.27
6.26
6.23
6.22
4.30
4.14
4.13
4.11
4.09
4.08
3.79
3.22
1.41
1.39
^{1}H NMR (400 MHz, CDCl$_3$)
NHBn
Me
MeO
4m
4.60
2.88
0.97
3.35
0.98
1.00
1.00
2.03
0.99
3.44
0.82
3.35
9.5
9.0
8.5
8.0
7.5
7.0
6.5
6.0
5.5
5.0
4.5
4.0
3.5
3.0
2.5
2.0
1.5
1.0
0.5
f1 (ppm)

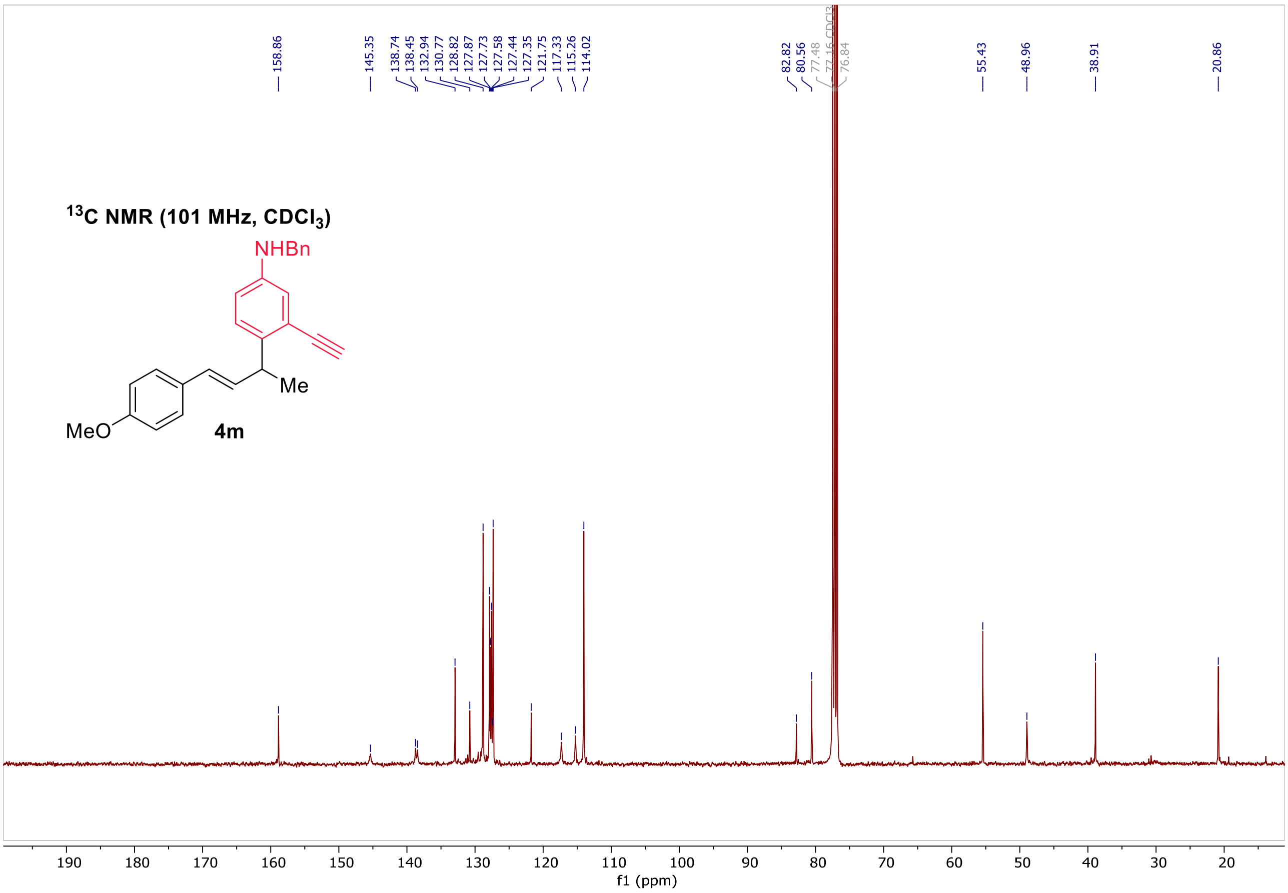

158.86
145.35
138.74
138.45
132.94
130.77
128.82
127.87
127.73
127.58
127.44
127.35
121.75
117.33
115.26
114.02
82.82
80.56
77.48
77.16 CDCl3
76.84
55.43
48.96
38.91
20.86
^{13}C NMR (101 MHz, CDCl$_3$)
NHBn
Me
MeO
4m
190
180
170
160
150
140
130
120
110
100
90
80
70
60
50
40
30
20
f1 (ppm)

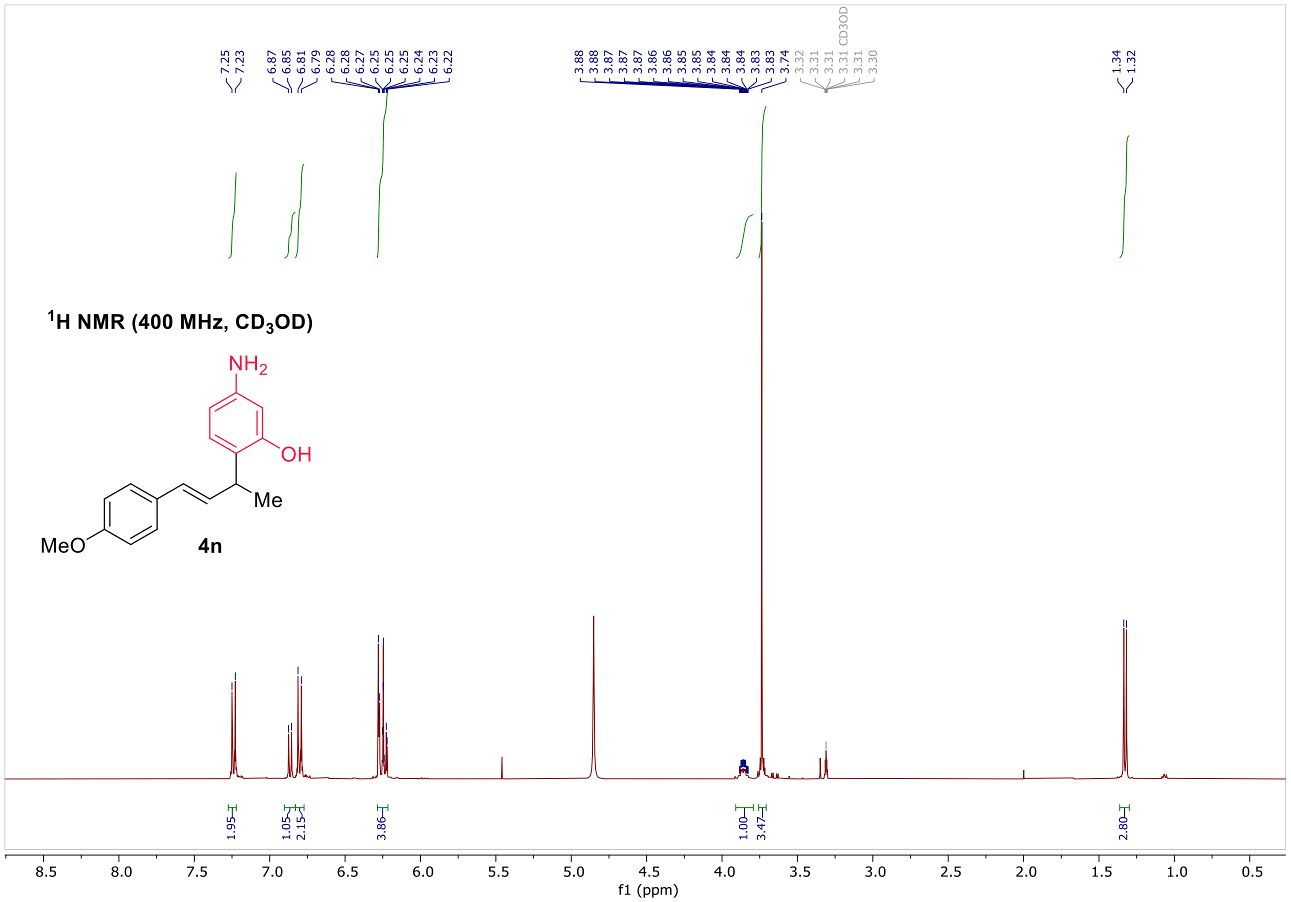

1H NMR (400 MHz, CD3OD)
NH2
OH
Me
MeO
4n
f1 (ppm)

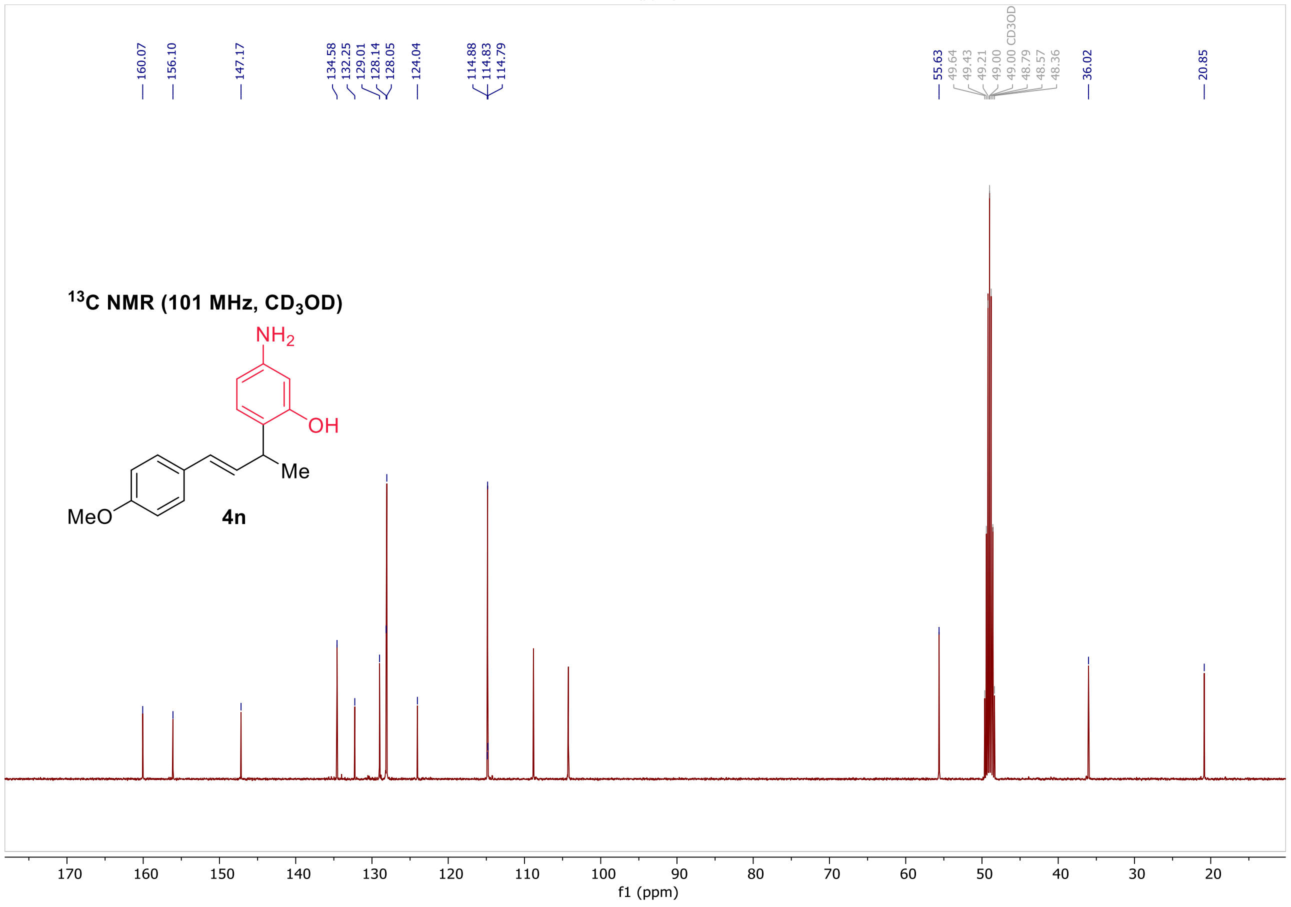

13C NMR (101 MHz, CD3OD)
NH2
OH
Me
MeO
4n
f1 (ppm)

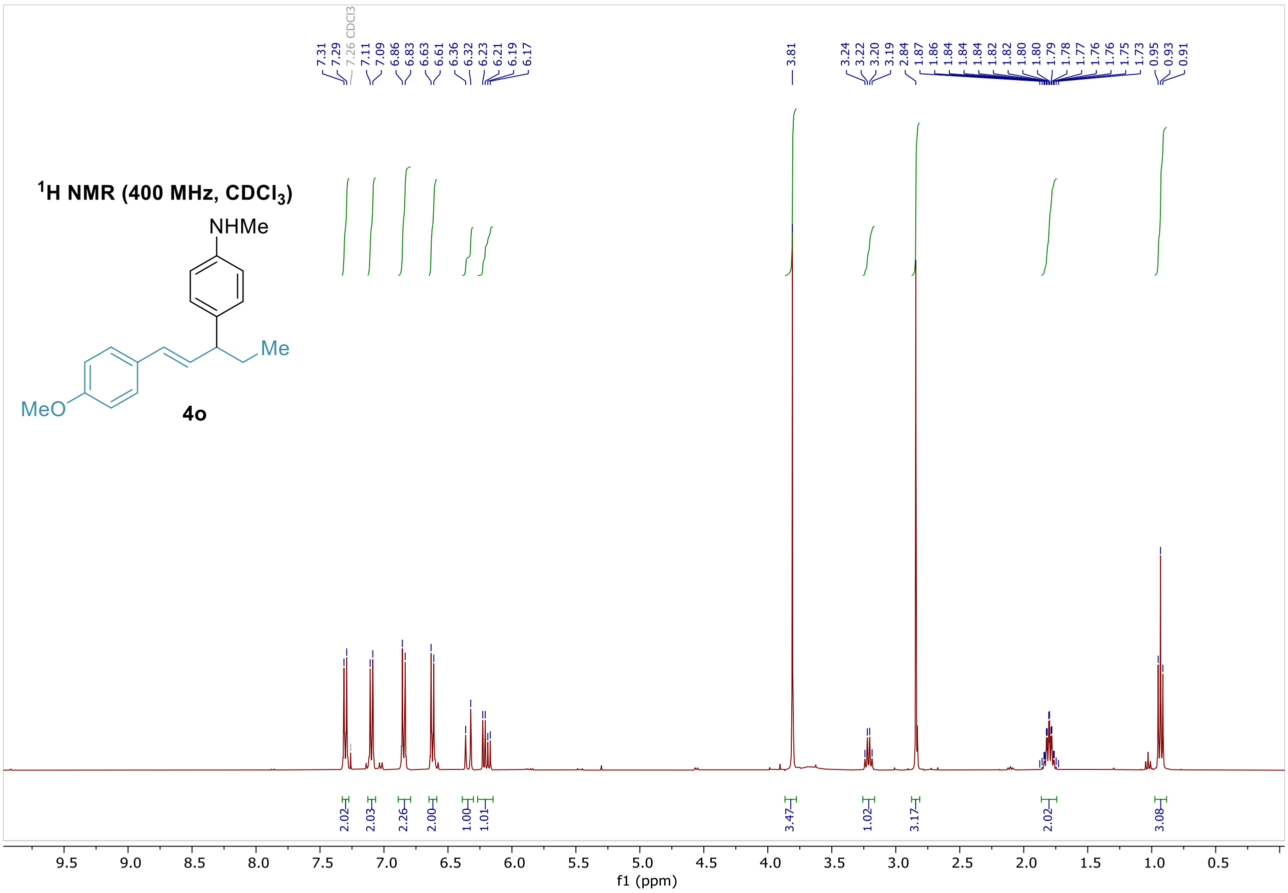

1H NMR (400 MHz, CDCl3)
NHMe
Me
MeO
4o
7.31 7.29 7.26 CDCl3 7.11 7.09 6.86 6.83 6.63 6.61 6.36 6.32 6.23 6.21 6.19 6.17
3.81
3.24 3.22 3.20 3.19 2.84 1.87 1.86 1.84 1.84 1.84 1.82 1.82 1.80 1.80 1.79 1.78 1.77 1.76 1.76 1.75 1.73 0.95 0.93 0.91
2.02 2.03 2.26 2.00 1.00 1.01 3.47 1.02 3.17 2.02 3.08
9.5 9.0 8.5 8.0 7.5 7.0 6.5 6.0 5.5 5.0 4.5 4.0 3.5 3.0 2.5 2.0 1.5 1.0 0.5
f1 (ppm)

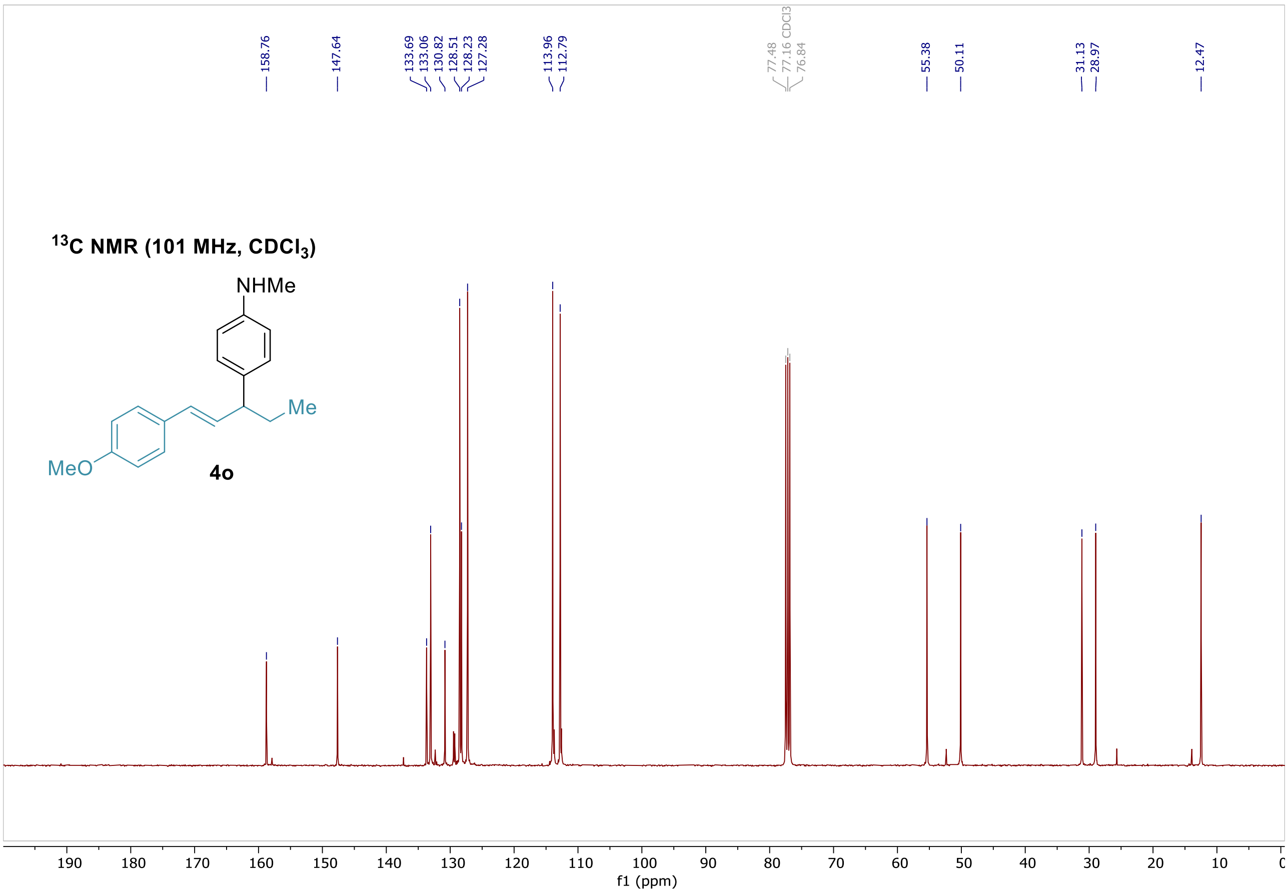

13C NMR (101 MHz, CDCl3)
NHMe
Me
MeO
4o
158.76
147.64
133.69 133.06 130.82 128.51 128.23 127.28
113.96 112.79
77.48 77.16 CDCl3 76.84
55.38
50.11
31.13 28.97
12.47
190 180 170 160 150 140 130 120 110 100 90 80 70 60 50 40 30 20 10 0
f1 (ppm)

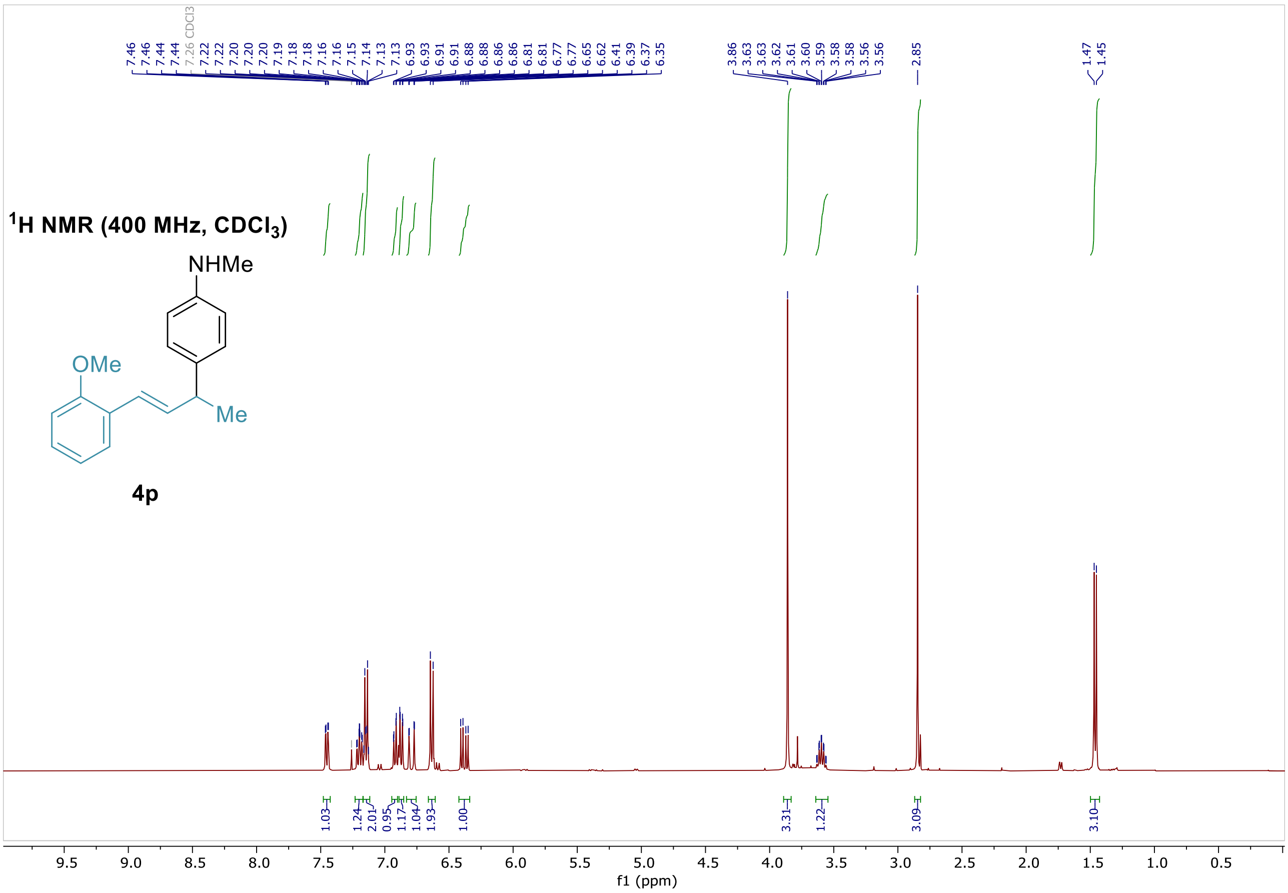
1H NMR (400 MHz, CDCl3)
NHMe
OMe
Me
4p
7.46 7.46 7.44 7.44 7.26 CDCl3 7.22 7.22 7.20 7.20 7.20 7.19 7.18 7.16 7.16 7.15 7.14 7.13 7.13 6.93 6.93 6.91 6.91 6.88 6.88 6.86 6.86 6.81 6.81 6.77 6.77 6.65 6.62 6.41 6.39 6.37 6.35
3.86 3.63 3.63 3.62 3.61 3.60 3.59 3.58 3.58 3.56 3.56
2.85
1.47 1.45
1.03 1.24 2.01 0.95 1.17 1.04 1.93 1.00 3.31 1.22 3.09 3.10
f1 (ppm)

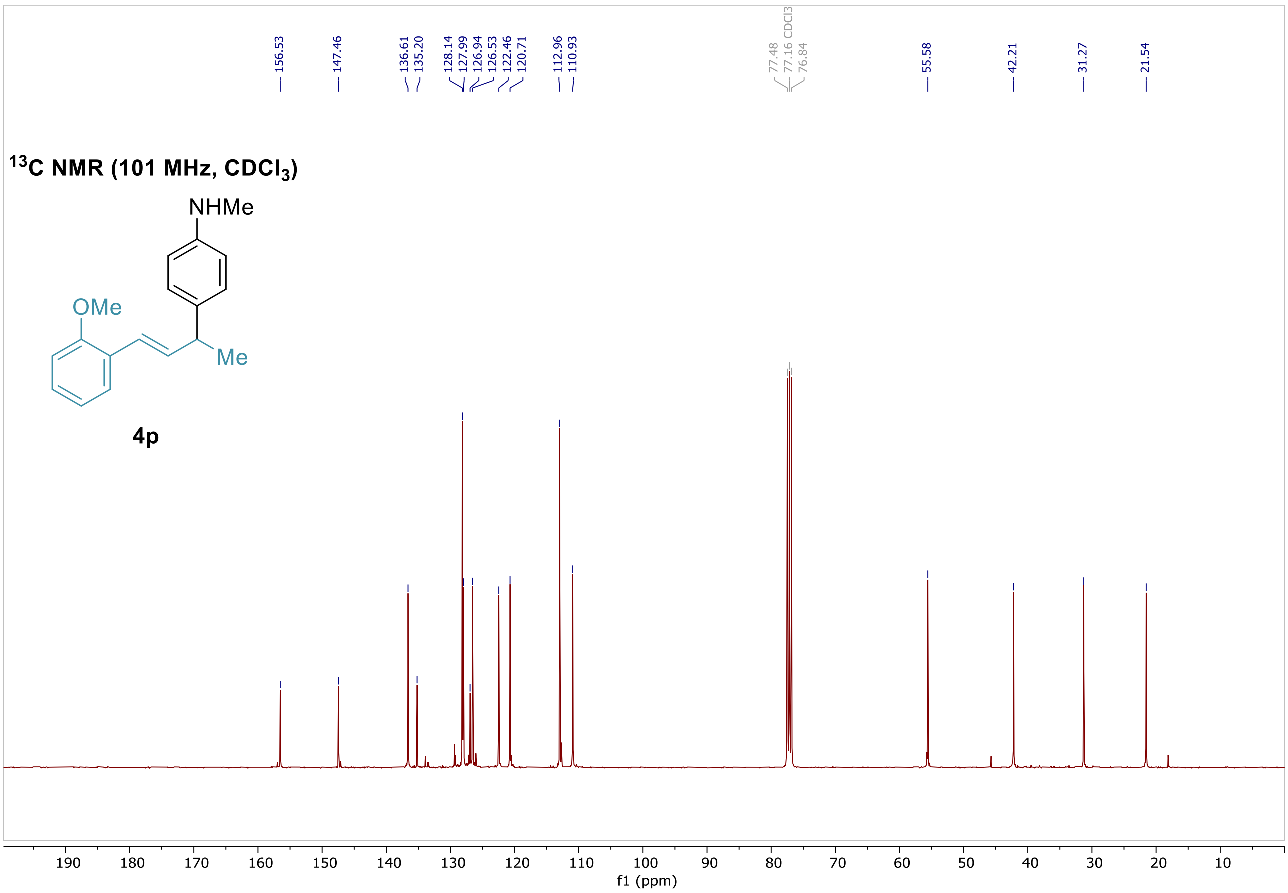
13C NMR (101 MHz, CDCl3)
NHMe
OMe
Me
4p
156.53 147.46 136.61 135.20 128.14 127.99 126.94 126.53 122.46 120.71 112.96 110.93
77.48 77.16 CDCl3 76.84
55.58 42.21 31.27 21.54
f1 (ppm)

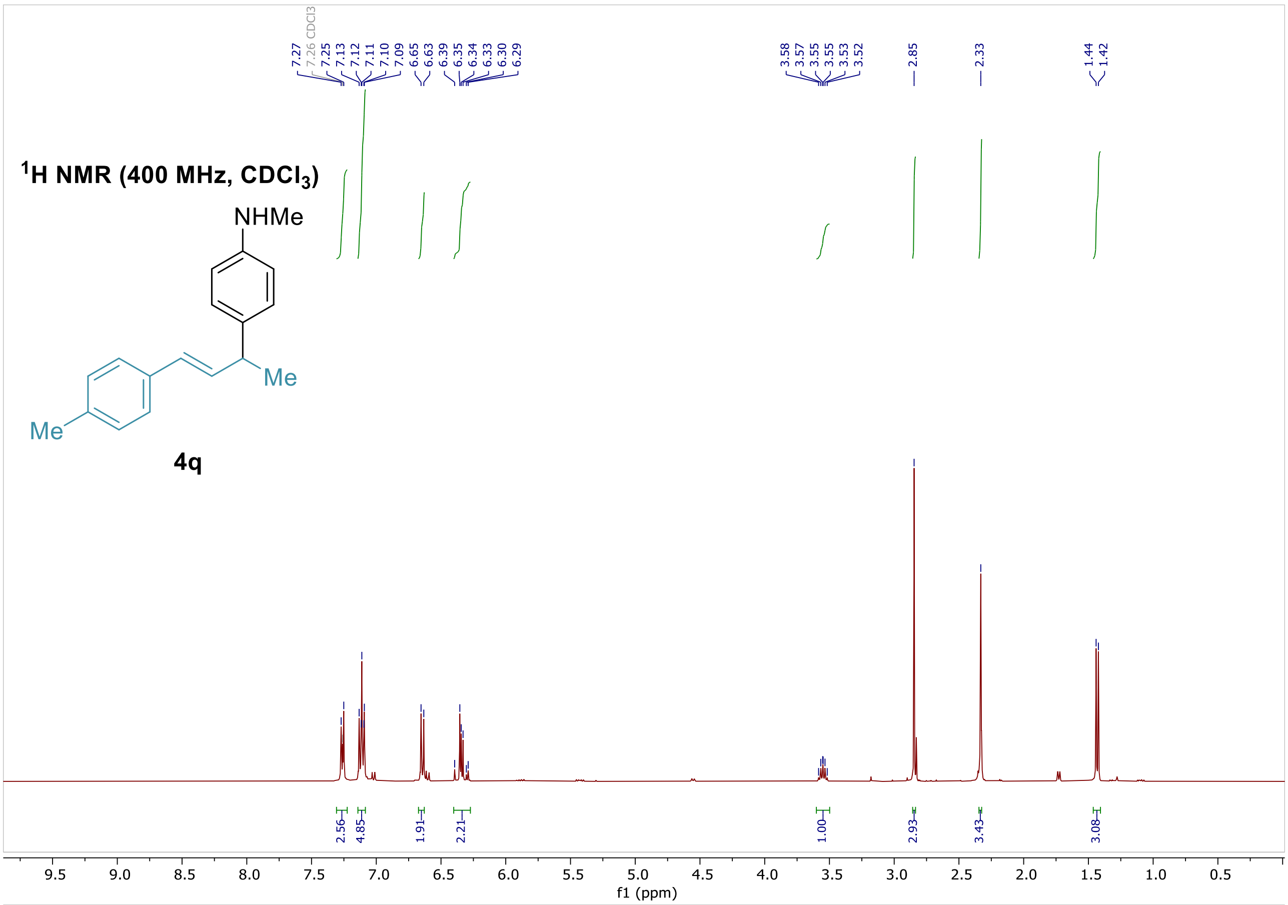

1H NMR (400 MHz, CDCl3)
NHMe
Me
Me
4q
7.27
7.26 CDCl3
7.25
7.13
7.12
7.11
7.10
7.09
6.65
6.63
6.39
6.35
6.34
6.33
6.30
6.29
3.58
3.57
3.55
3.55
3.53
3.52
2.85
2.33
1.44
1.42
2.56
4.85
1.91
2.21
1.00
2.93
3.43
3.08
9.5 9.0 8.5 8.0 7.5 7.0 6.5 6.0 5.5 5.0 4.5 4.0 3.5 3.0 2.5 2.0 1.5 1.0 0.5
f1 (ppm)

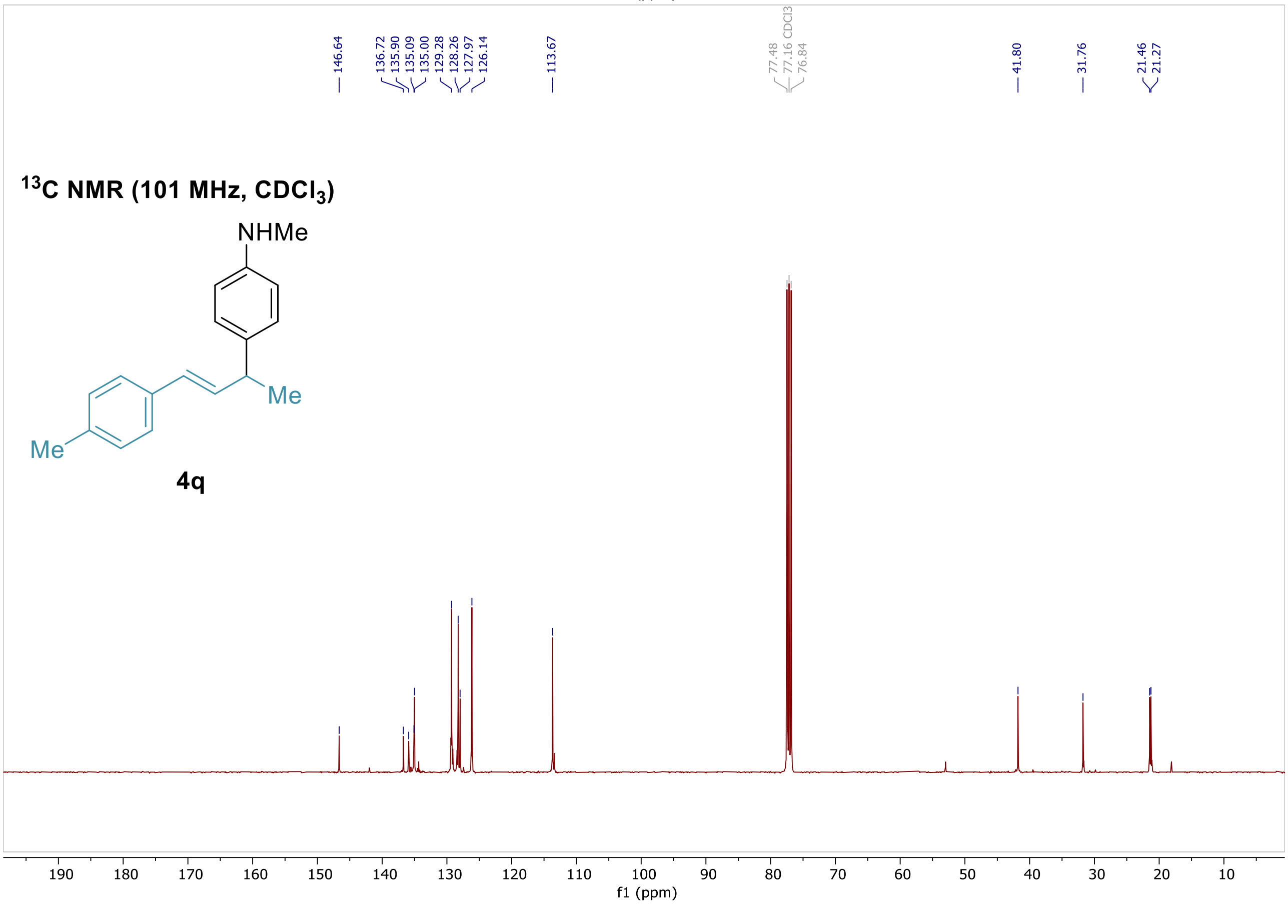

13C NMR (101 MHz, CDCl3)
NHMe
Me
Me
4q
146.64
136.72
135.90
135.09
135.00
129.28
128.26
127.97
126.14
113.67
77.48
77.16 CDCl3
76.84
41.80
31.76
21.46
21.27
190 180 170 160 150 140 130 120 110 100 90 80 70 60 50 40 30 20 10
f1 (ppm)

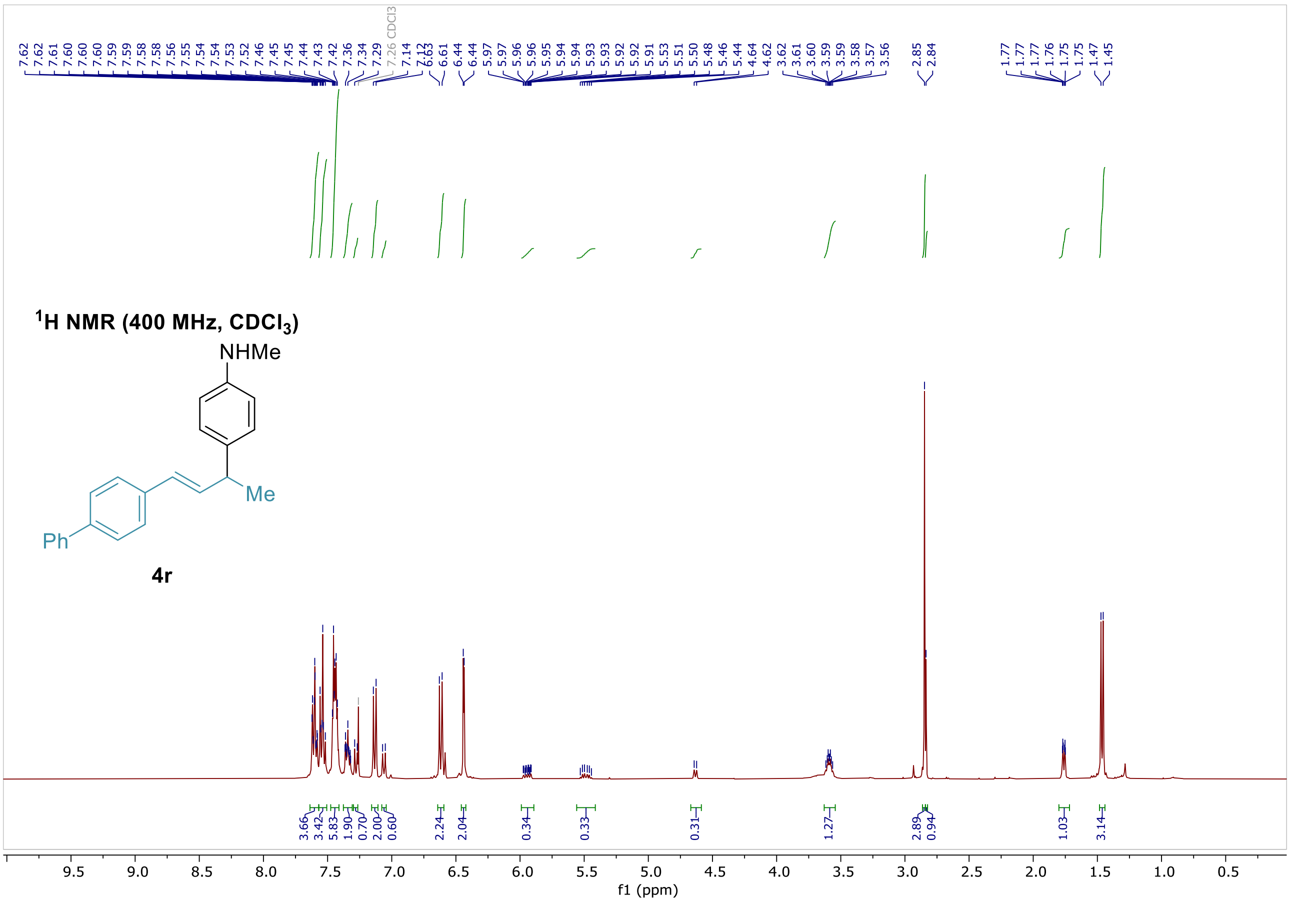

^{1}H NMR (400 MHz, $CDCl_3$)
NHMe
Me
Ph
4r
f1 (ppm)

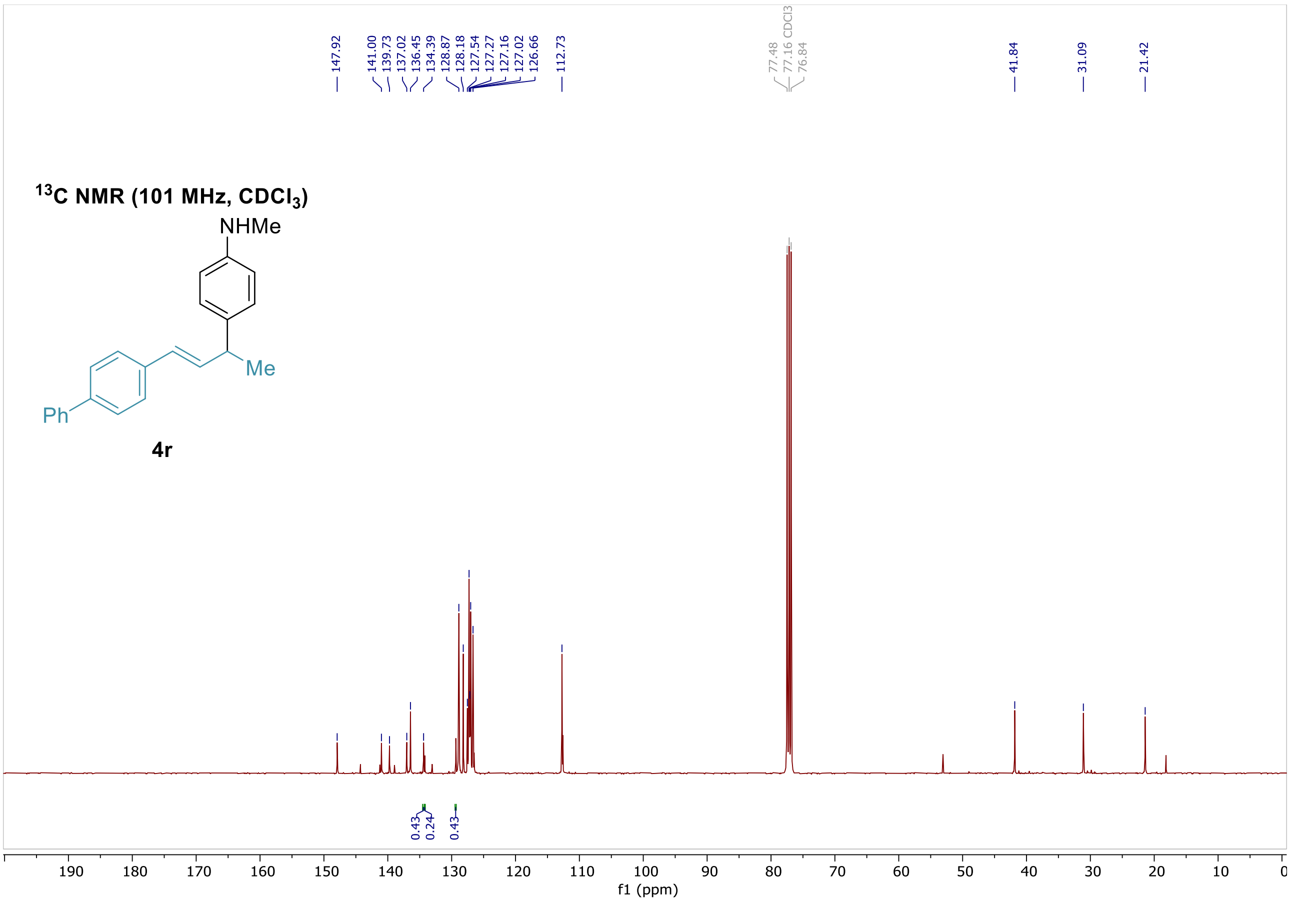

^{13}C NMR (101 MHz, $CDCl_3$)
NHMe
Me
Ph
4r
f1 (ppm)

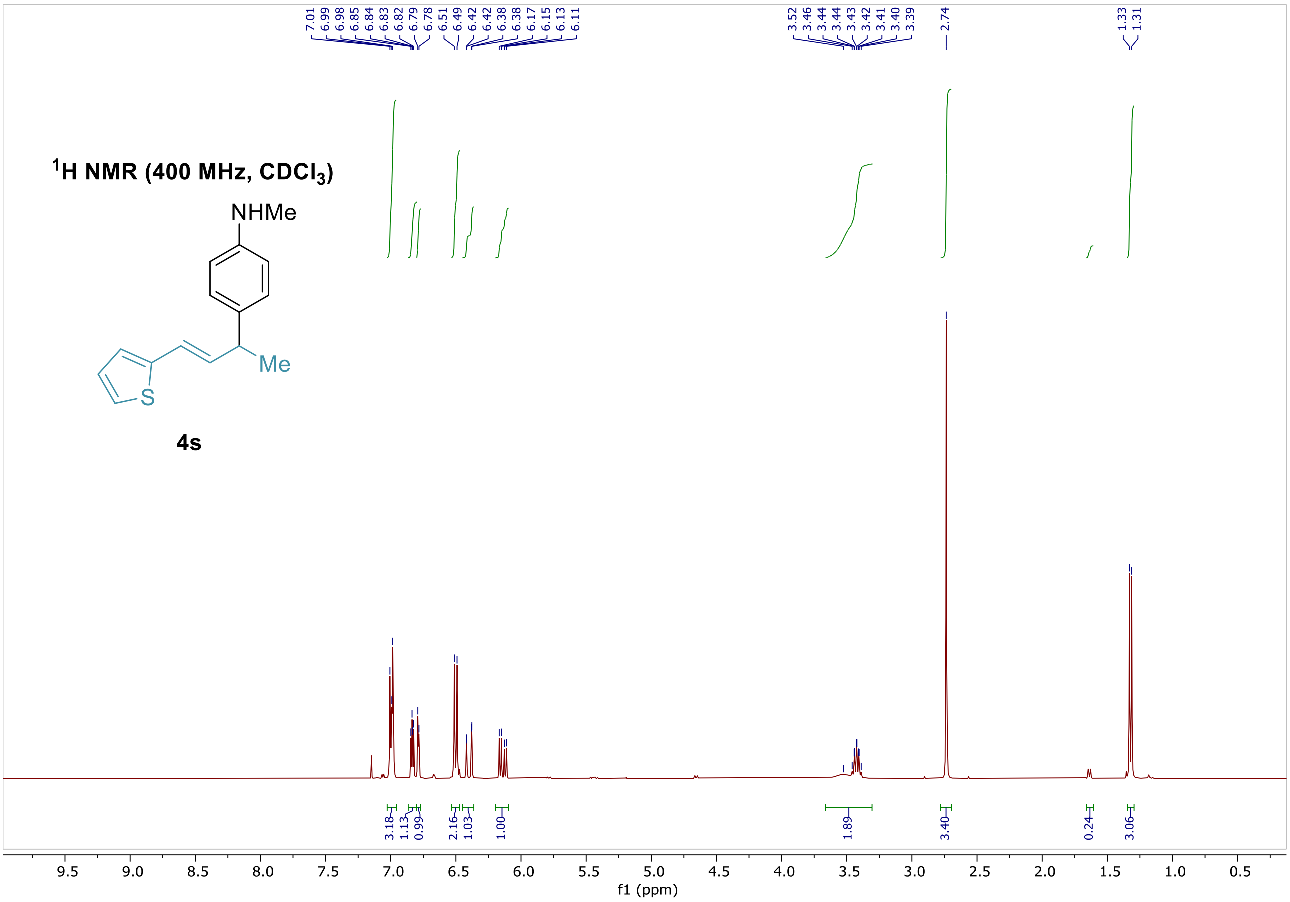
7.01 6.99 6.98 6.85 6.84 6.83 6.82 6.79 6.78 6.51 6.49 6.42 6.42 6.38 6.38 6.17 6.15 6.13 6.11
3.52 3.46 3.44 3.44 3.43 3.42 3.41 3.40 3.39
2.74
1.33 1.31
1H NMR (400 MHz, CDCl3)
NHMe
Me
S
4s
3.18 1.13 0.99 2.16 1.03 1.00 1.89 3.40 0.24 3.06
9.5 9.0 8.5 8.0 7.5 7.0 6.5 6.0 5.5 5.0 4.5 4.0 3.5 3.0 2.5 2.0 1.5 1.0 0.5
f1 (ppm)

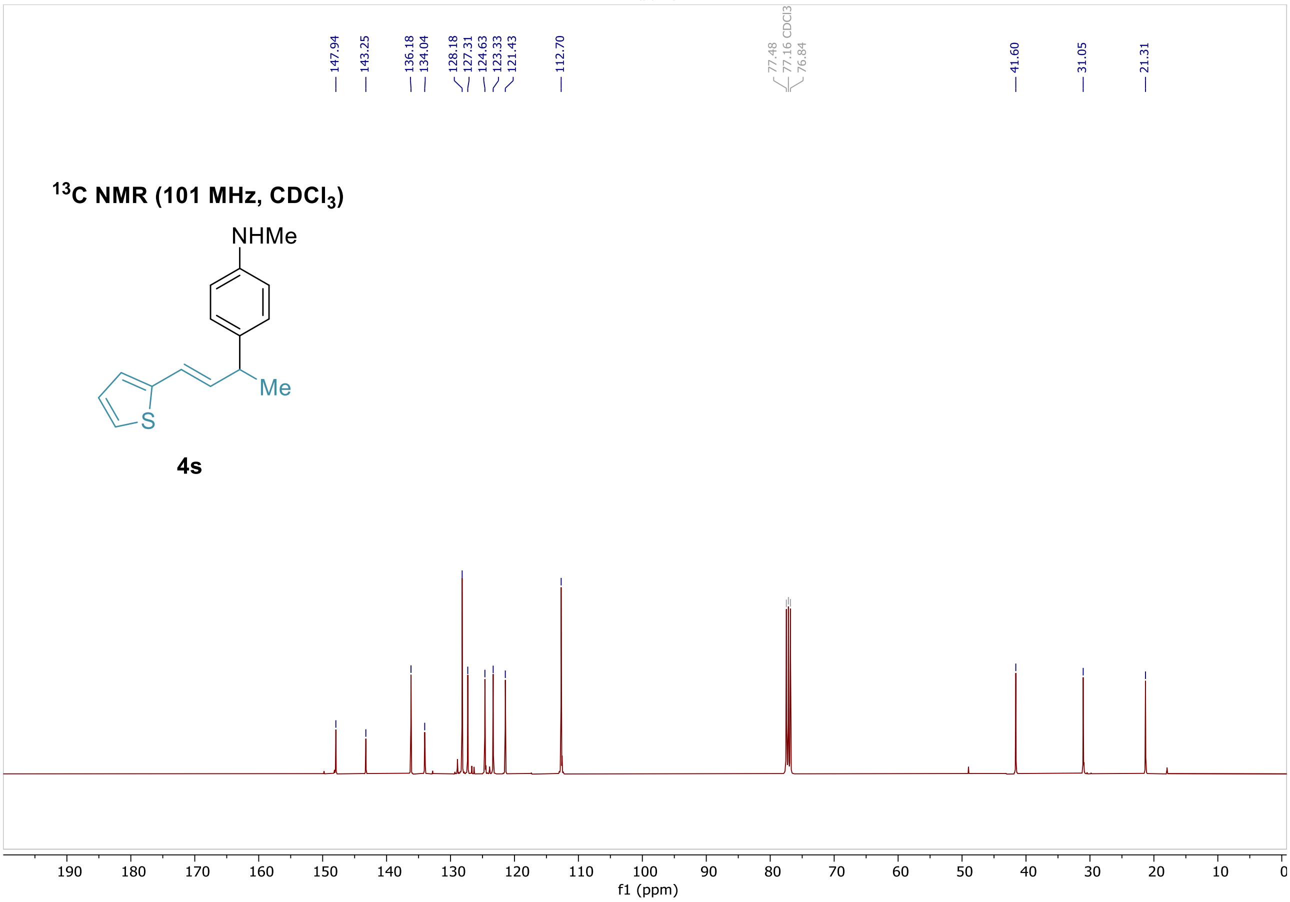
147.94
143.25
136.18 134.04
128.18 127.31 124.63 123.33 121.43
112.70
77.48 77.16 CDCl3 76.84
41.60
31.05
21.31
13C NMR (101 MHz, CDCl3)
NHMe
Me
S
4s
190 180 170 160 150 140 130 120 110 100 90 80 70 60 50 40 30 20 10 0
f1 (ppm)

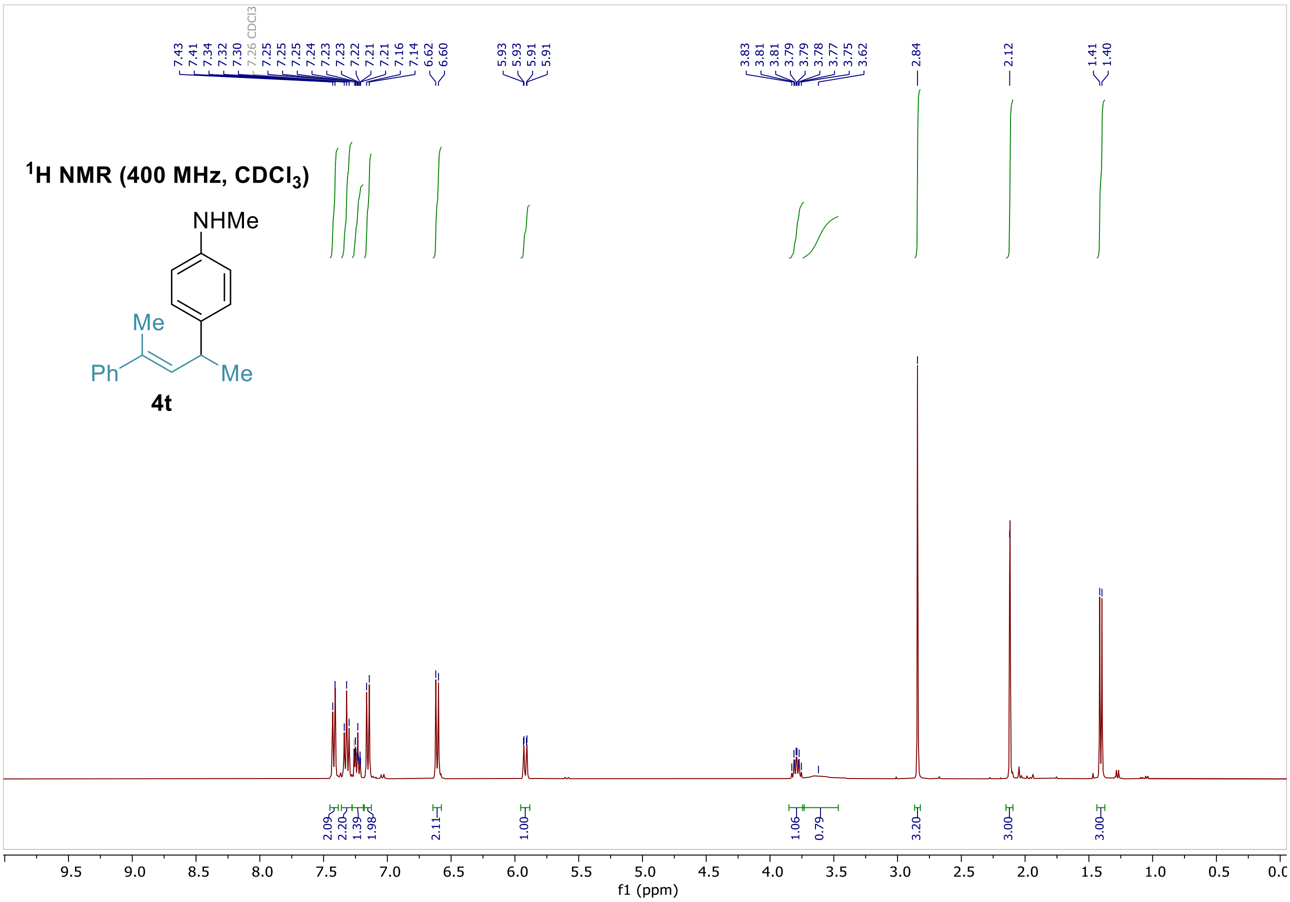

7.43
7.41
7.34
7.32
7.30
7.26 CDCl3
7.25
7.25
7.25
7.24
7.23
7.23
7.22
7.21
7.21
7.16
7.14
6.62
6.60
5.93
5.93
5.91
5.91
3.83
3.81
3.81
3.79
3.79
3.78
3.77
3.75
3.62
2.84
2.12
1.41
1.40
1H NMR (400 MHz, CDCl3)
NHMe
Me
Ph
Me
4t
2.09
2.20
1.39
1.98
2.11
1.00
1.06
0.79
3.20
3.00
3.00
9.5
9.0
8.5
8.0
7.5
7.0
6.5
6.0
5.5
5.0
4.5
4.0
3.5
3.0
2.5
2.0
1.5
1.0
0.5
f1 (ppm)

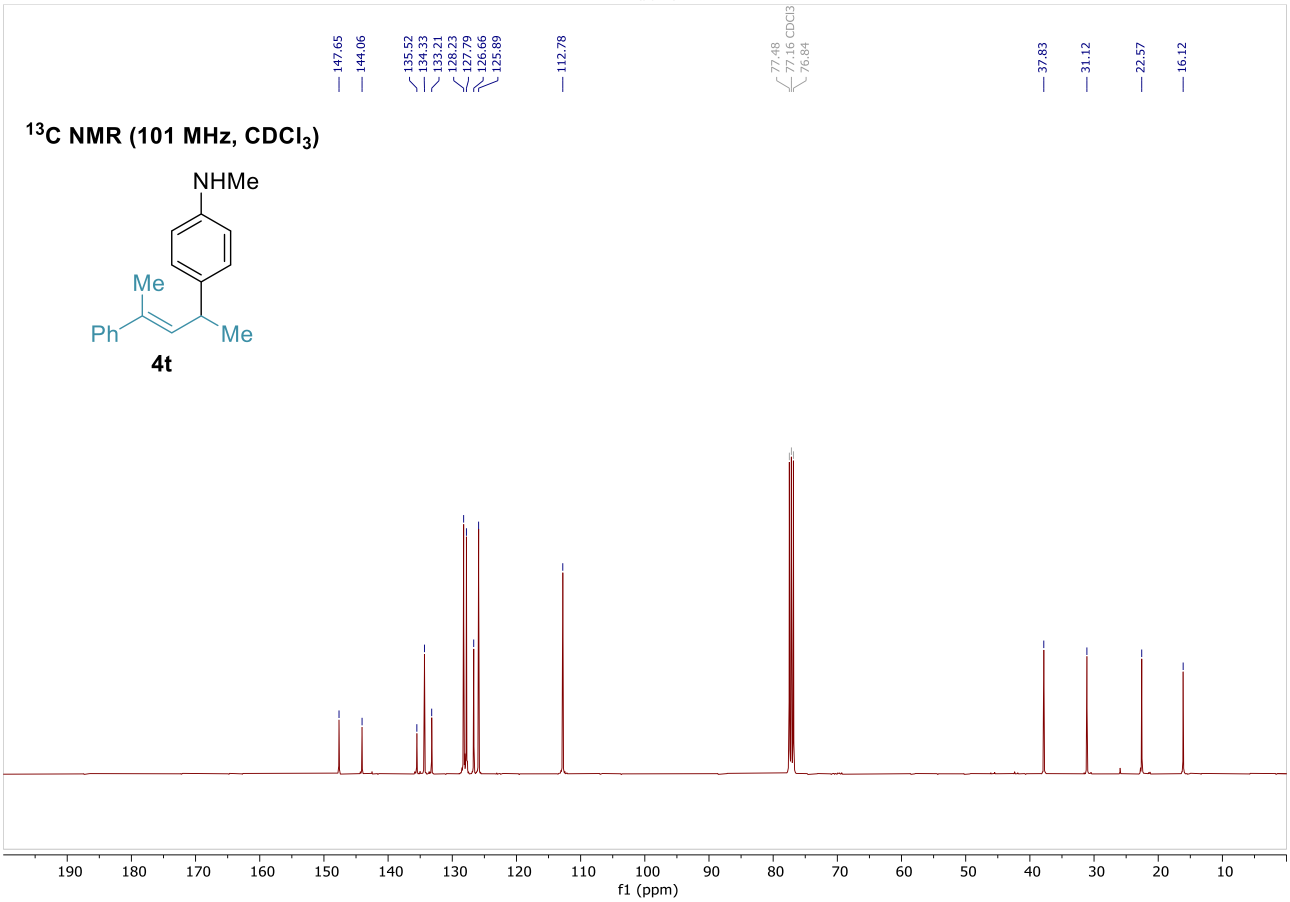

147.65
144.06
135.52
134.33
133.21
128.23
127.79
126.66
125.89
112.78
77.48
77.16 CDCl3
76.84
37.83
31.12
22.57
16.12
13C NMR (101 MHz, CDCl3)
NHMe
Me
Ph
Me
4t
190
180
170
160
150
140
130
120
110
100
90
80
70
60
50
40
30
20
10
f1 (ppm)

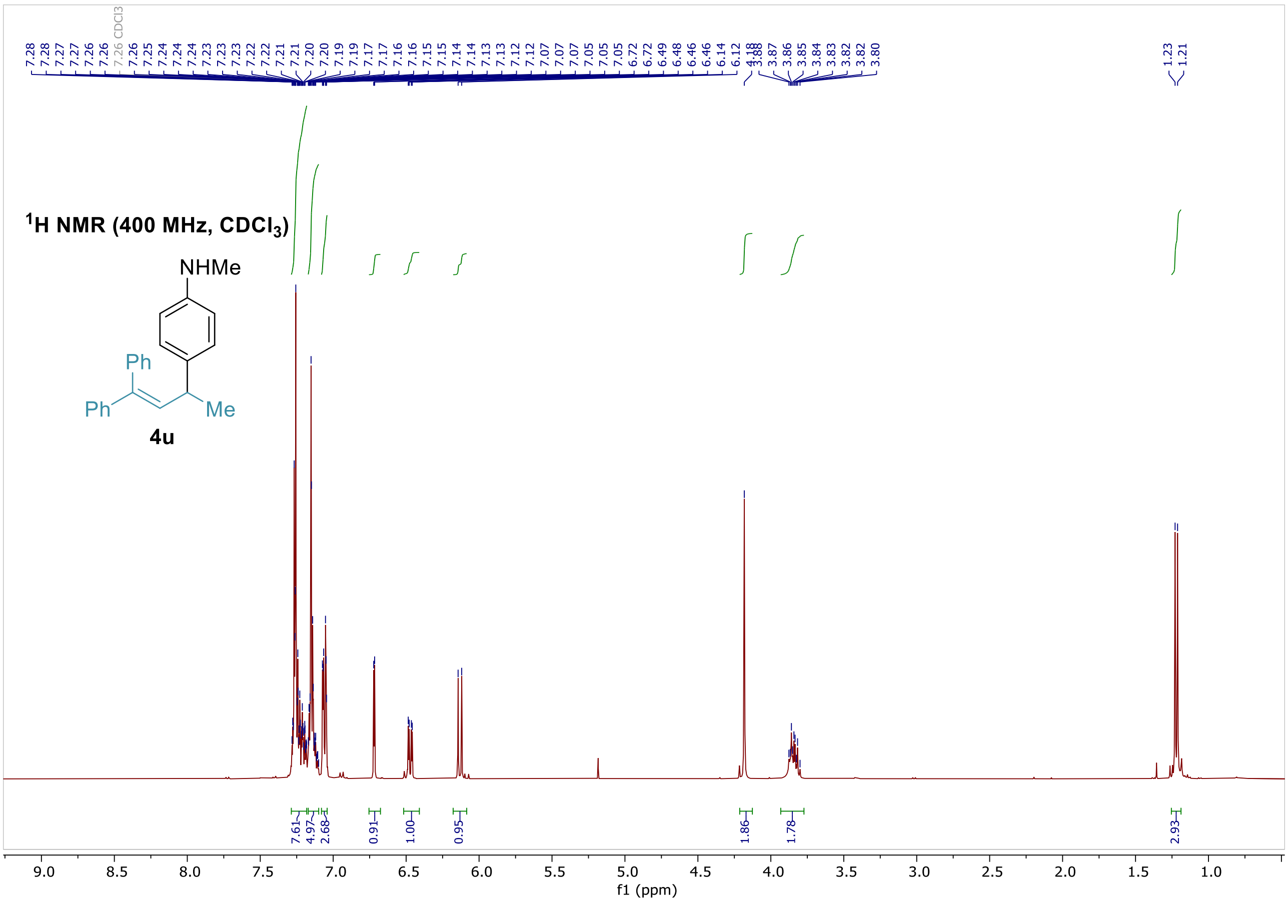
1H NMR (400 MHz, CDCl3)
NHMe
Ph
Ph
Me
4u
7.28 7.28 7.27 7.26 7.26 7.26 CDCl3 7.26 7.25 7.24 7.24 7.24 7.23 7.23 7.23 7.22 7.22 7.21 7.21 7.20 7.20 7.19 7.19 7.17 7.17 7.16 7.16 7.15 7.15 7.14 7.14 7.13 7.13 7.12 7.12 7.07 7.07 7.07 7.05 7.05 6.72 6.72 6.49 6.48 6.46 6.46 6.14 6.12 4.18 3.88 3.87 3.86 3.85 3.84 3.83 3.82 3.82 3.80 1.23 1.21
7.61 4.97 2.68 0.91 1.00 0.95 1.86 1.78 2.93
9.0 8.5 8.0 7.5 7.0 6.5 6.0 5.5 5.0 4.5 4.0 3.5 3.0 2.5 2.0 1.5 1.0
f1 (ppm)

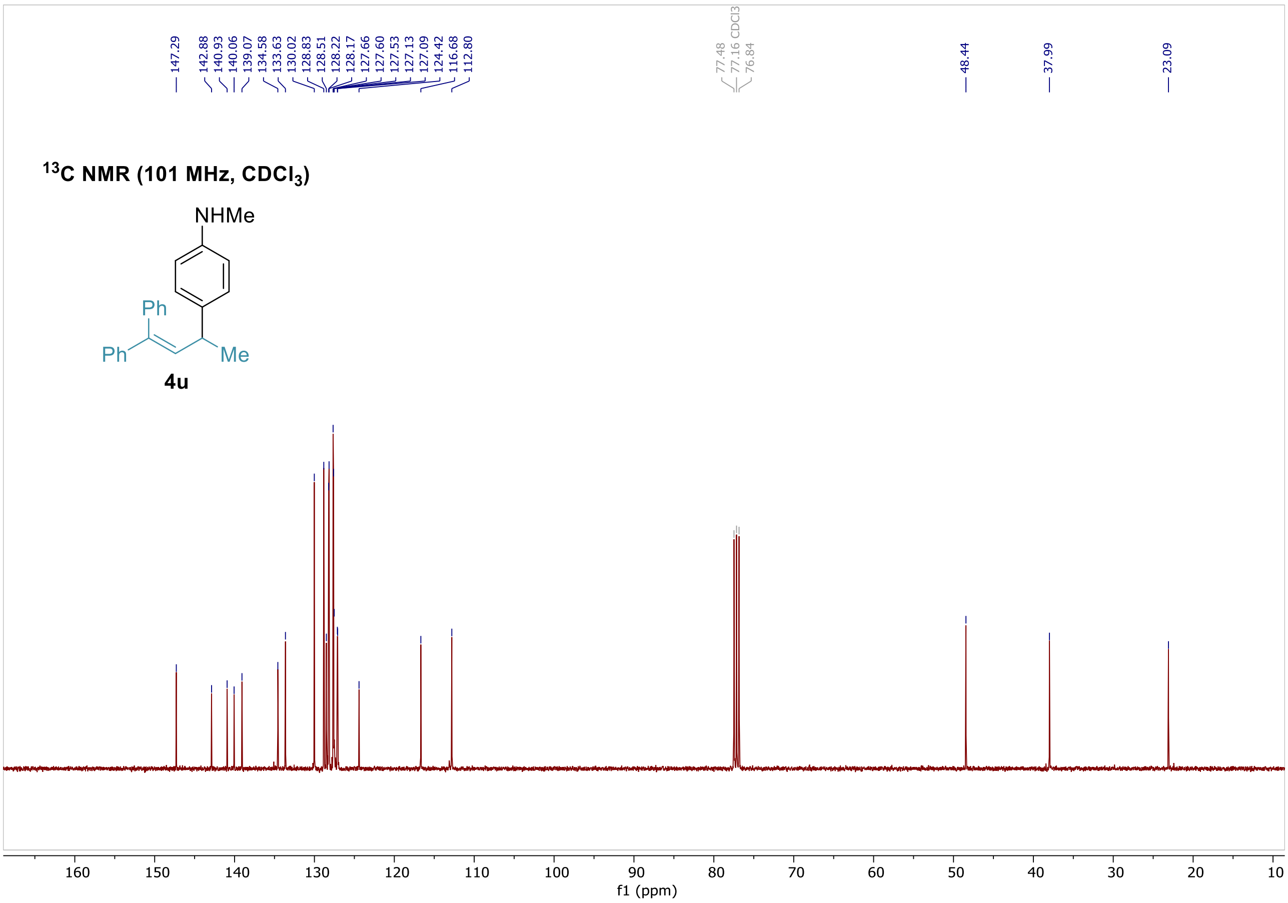
13C NMR (101 MHz, CDCl3)
NHMe
Ph
Ph
Me
4u
147.29 142.88 140.93 140.06 139.07 134.58 133.63 130.02 128.83 128.51 128.22 128.17 127.66 127.60 127.53 127.13 127.09 124.42 116.68 112.80 77.48 77.16 CDCl3 76.84 48.44 37.99 23.09
160 150 140 130 120 110 100 90 80 70 60 50 40 30 20 10
f1 (ppm)

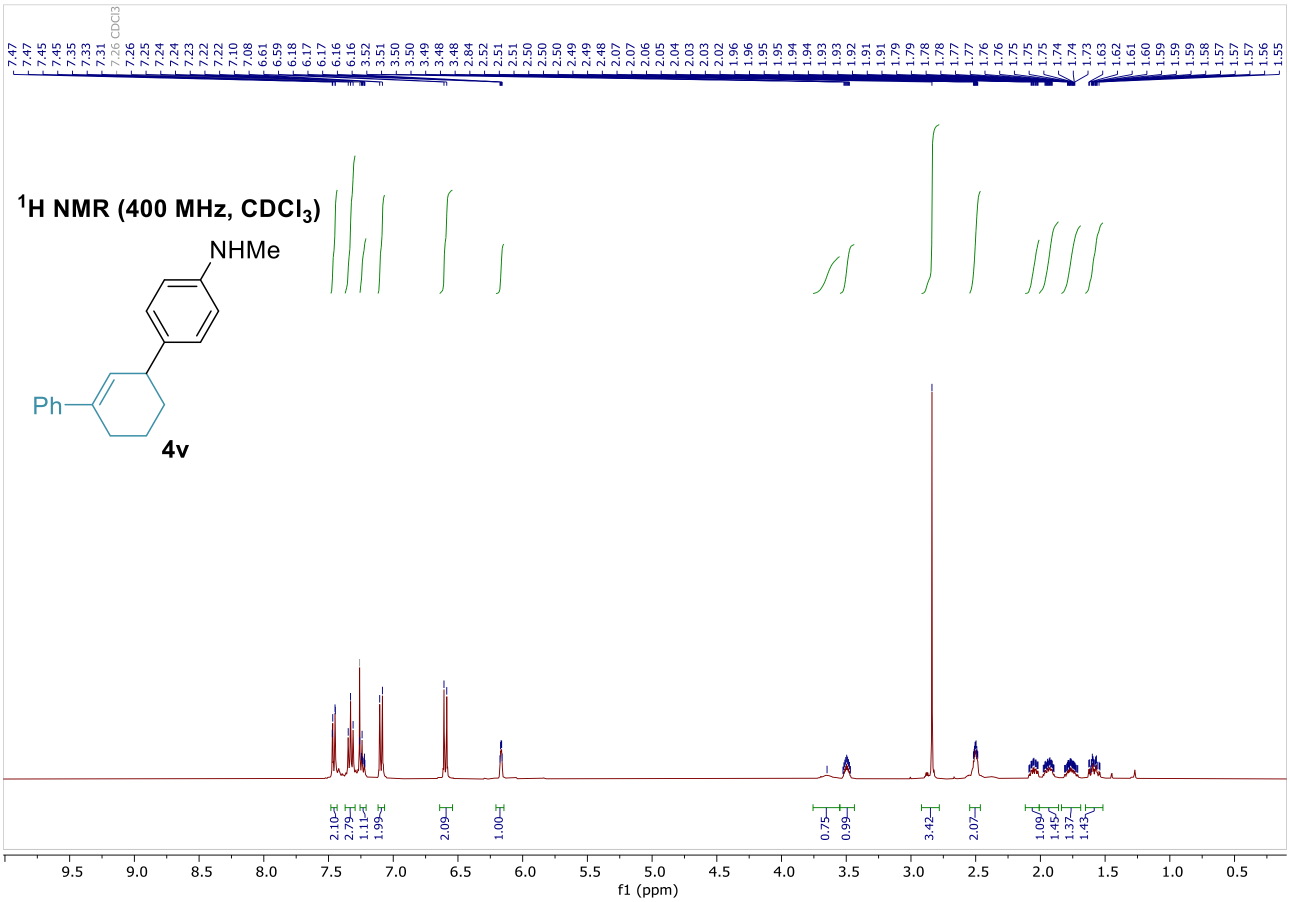
7.47 7.47 7.45 7.45 7.35 7.33 7.31 7.26 CDCl3 7.26 7.25 7.24 7.24 7.23 7.22 7.22 7.10 7.08 6.61 6.59 6.18 6.17 6.17 6.16 6.16 3.52 3.51 3.50 3.50 3.49 3.48 3.48 2.84 2.52 2.51 2.51 2.50 2.50 2.49 2.49 2.48 2.07 2.06 2.05 2.04 2.03 2.03 2.02 1.96 1.95 1.95 1.94 1.94 1.93 1.93 1.92 1.91 1.91 1.91 1.79 1.79 1.78 1.78 1.77 1.77 1.76 1.76 1.75 1.75 1.75 1.74 1.74 1.73 1.63 1.62 1.61 1.60 1.59 1.59 1.59 1.58 1.57 1.57 1.57 1.56 1.55
^{1}H NMR (400 MHz, CDCl$_3$)
NHMe
Ph
4v
2.10 2.79 1.11 1.99 2.09 1.00 0.75 0.99 3.42 2.07 1.09 1.45 1.37 1.43
9.5 9.0 8.5 8.0 7.5 7.0 6.5 6.0 5.5 5.0 4.5 4.0 3.5 3.0 2.5 2.0 1.5 1.0 0.5
f1 (ppm)

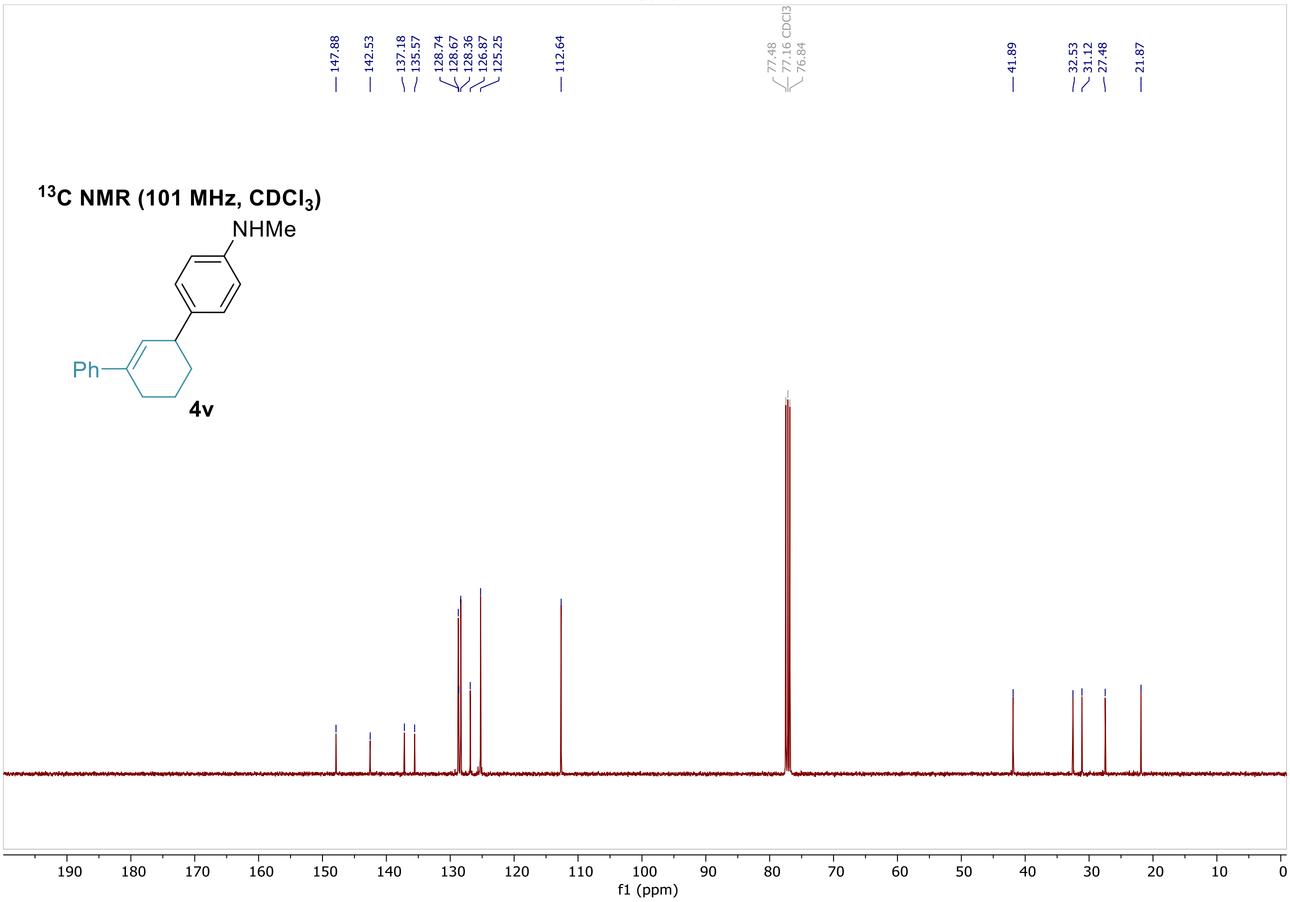
147.88 142.53 137.18 135.57 128.74 128.67 128.36 126.87 125.25 112.64 77.48 77.16 CDCl3 76.84 41.89 32.53 31.12 27.48 21.87
^{13}C NMR (101 MHz, CDCl$_3$)
NHMe
Ph
4v
190 180 170 160 150 140 130 120 110 100 90 80 70 60 50 40 30 20 10 0
f1 (ppm)

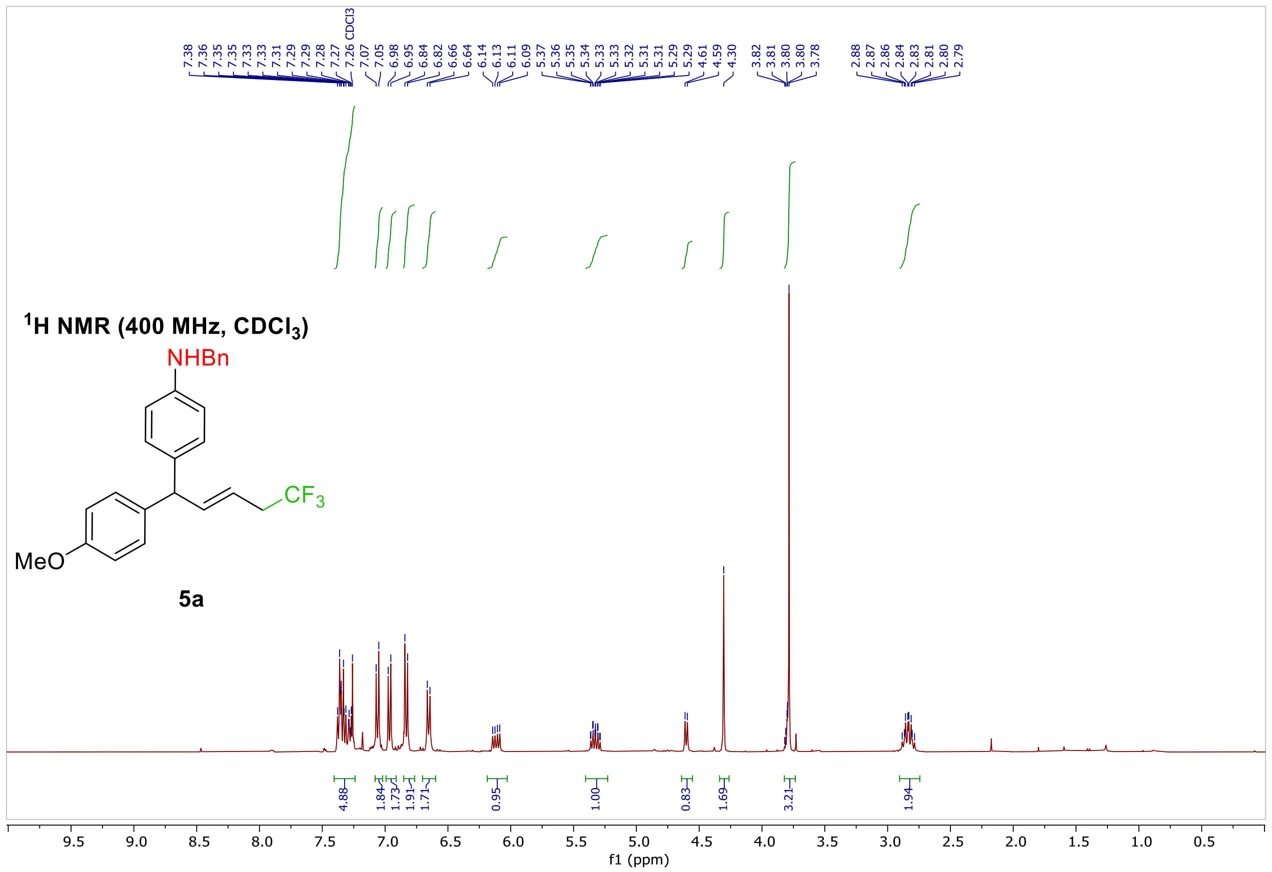
7.38 7.36 7.35 7.35 7.33 7.33 7.31 7.29 7.29 7.28 7.27 7.26 CDCl3 7.07 7.05 6.98 6.95 6.84 6.82 6.66 6.64 6.14 6.13 6.11 6.09 5.37 5.36 5.35 5.34 5.33 5.33 5.32 5.31 5.31 5.29 5.29 4.61 4.59 4.30 3.82 3.81 3.80 3.80 3.78 2.88 2.87 2.86 2.84 2.83 2.81 2.80 2.79
1H NMR (400 MHz, CDCl3)
NHBn
CF3
MeO
5a
4.88 1.84 1.73 1.91 1.71 0.95 1.00 0.83 1.69 3.21 1.94
f1 (ppm)

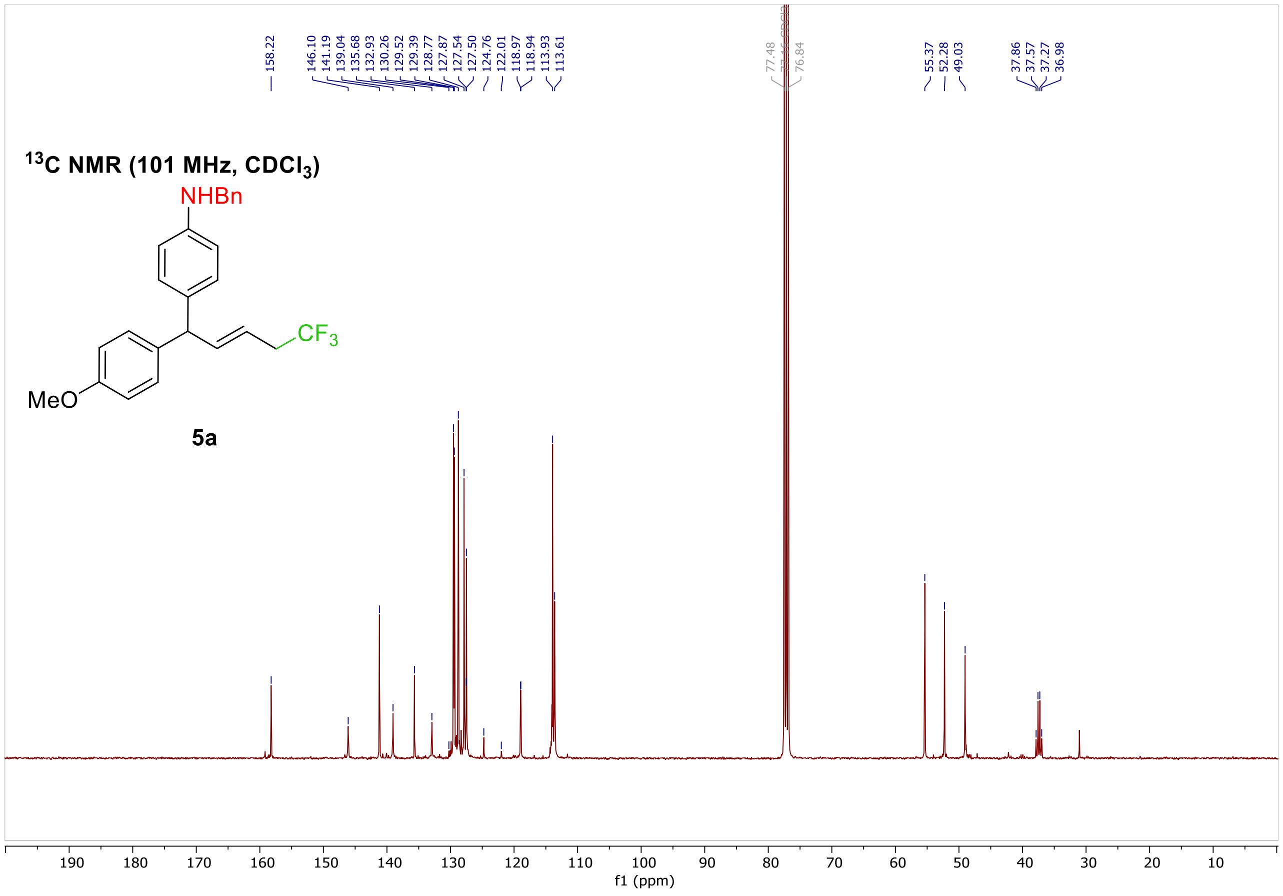
158.22 146.10 141.19 139.04 135.68 132.93 130.26 129.52 129.39 128.77 127.87 127.54 127.50 124.76 122.01 118.97 118.94 113.93 113.61 77.48 76.84 55.37 52.28 49.03 37.86 37.57 37.27 36.98
13C NMR (101 MHz, CDCl3)
NHBn
CF3
MeO
5a
f1 (ppm)

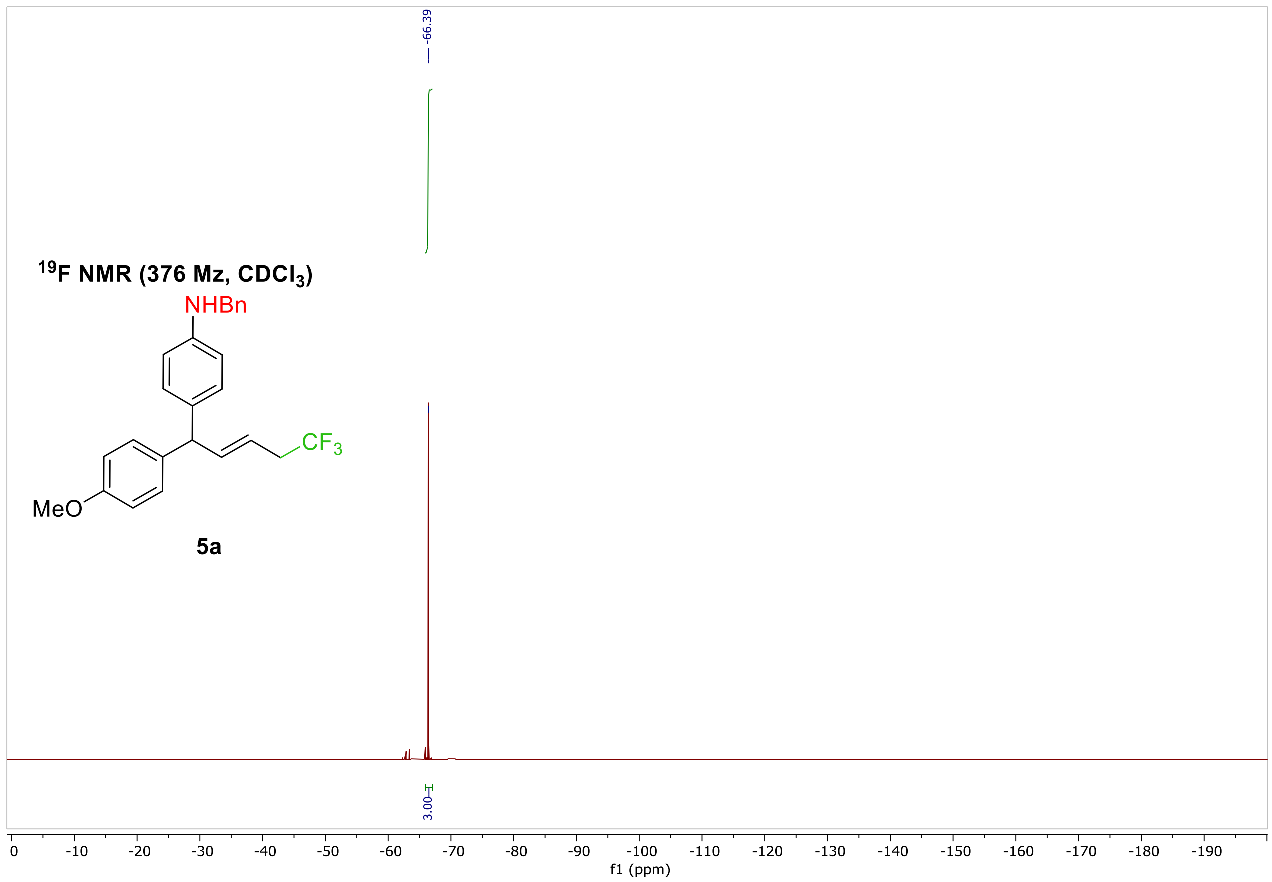

-66.39
^{19}F NMR (376 Mz, CDCl$_3$)
NHBn
CF$_3$
MeO
5a
3.00
f1 (ppm)

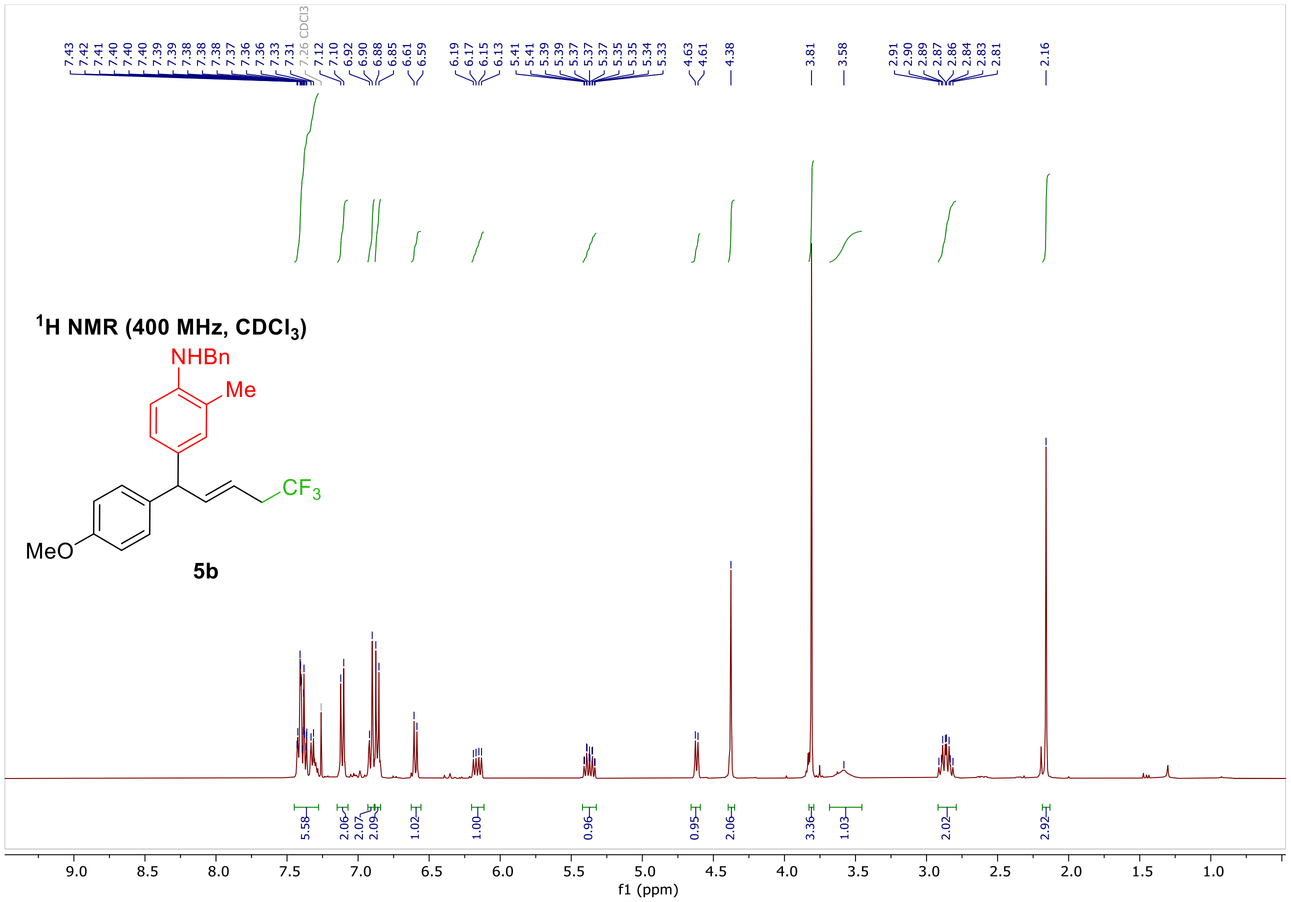

7.43 7.42 7.41 7.40 7.40 7.40 7.39 7.39 7.38 7.38 7.38 7.37 7.36 7.36 7.33 7.31 7.26 CDCl3 7.12 7.10 6.92 6.90 6.88 6.85 6.61 6.59 6.19 6.17 6.15 6.13 5.41 5.41 5.39 5.39 5.37 5.37 5.37 5.35 5.35 5.34 5.33 4.63 4.61 4.38 3.81 3.58 2.91 2.90 2.89 2.87 2.86 2.84 2.83 2.81 2.16
^{1}H NMR (400 MHz, CDCl$_3$)
NHBn
Me
CF$_3$
MeO
5b
5.58 2.06 2.07 2.09 1.02 1.00 0.96 0.95 2.06 3.36 1.03 2.02 2.92
f1 (ppm)

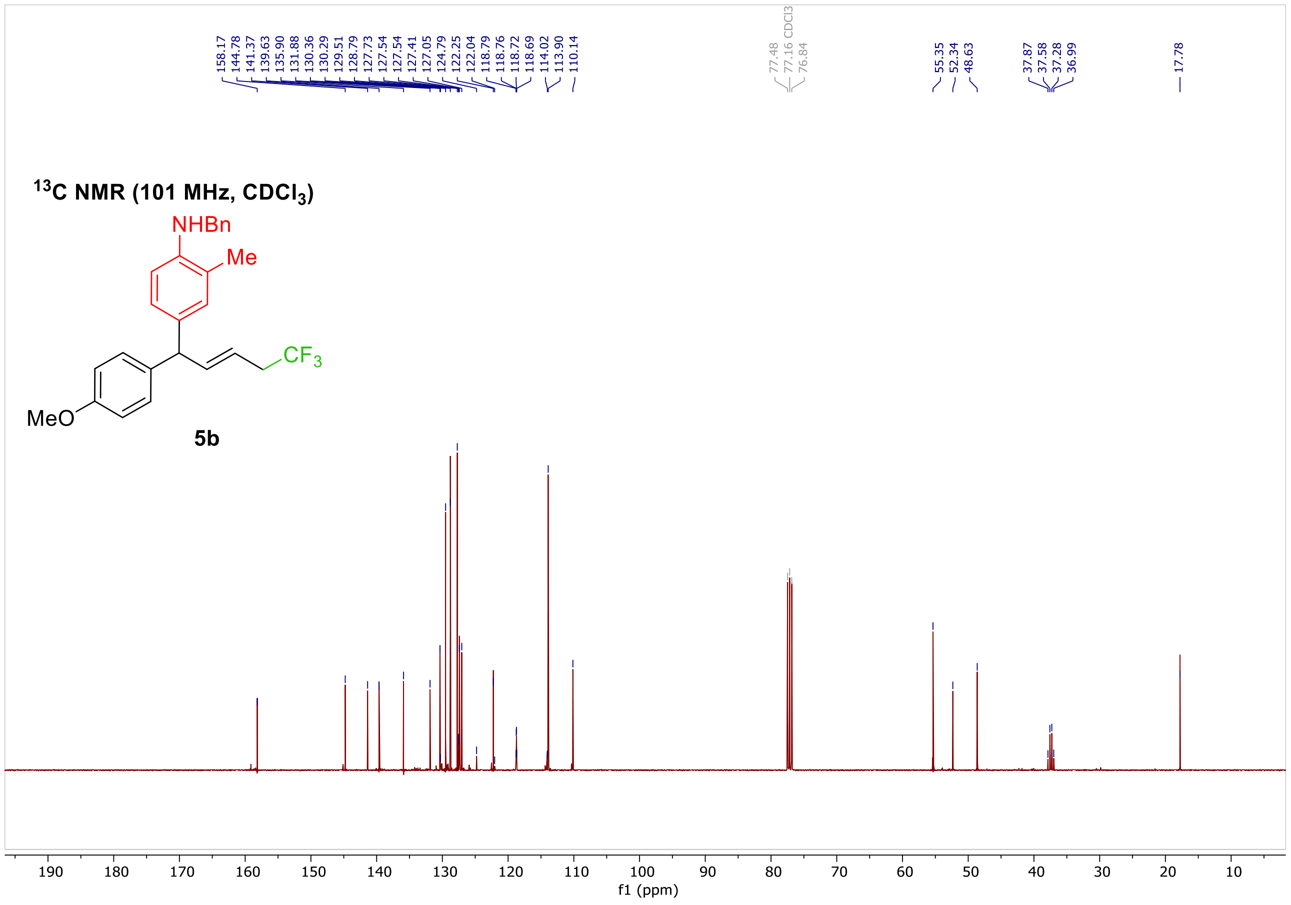
158.17
144.78
141.37
139.63
135.90
131.88
130.36
130.29
129.51
128.79
127.73
127.54
127.54
127.41
127.05
124.79
122.25
122.04
118.79
118.76
118.72
118.69
114.02
113.90
110.14
77.48
77.16 CDCl3
76.84
55.35
52.34
48.63
37.87
37.58
37.28
36.99
17.78
13C NMR (101 MHz, CDCl3)
NHBn
Me
CF3
MeO
5b
f1 (ppm)

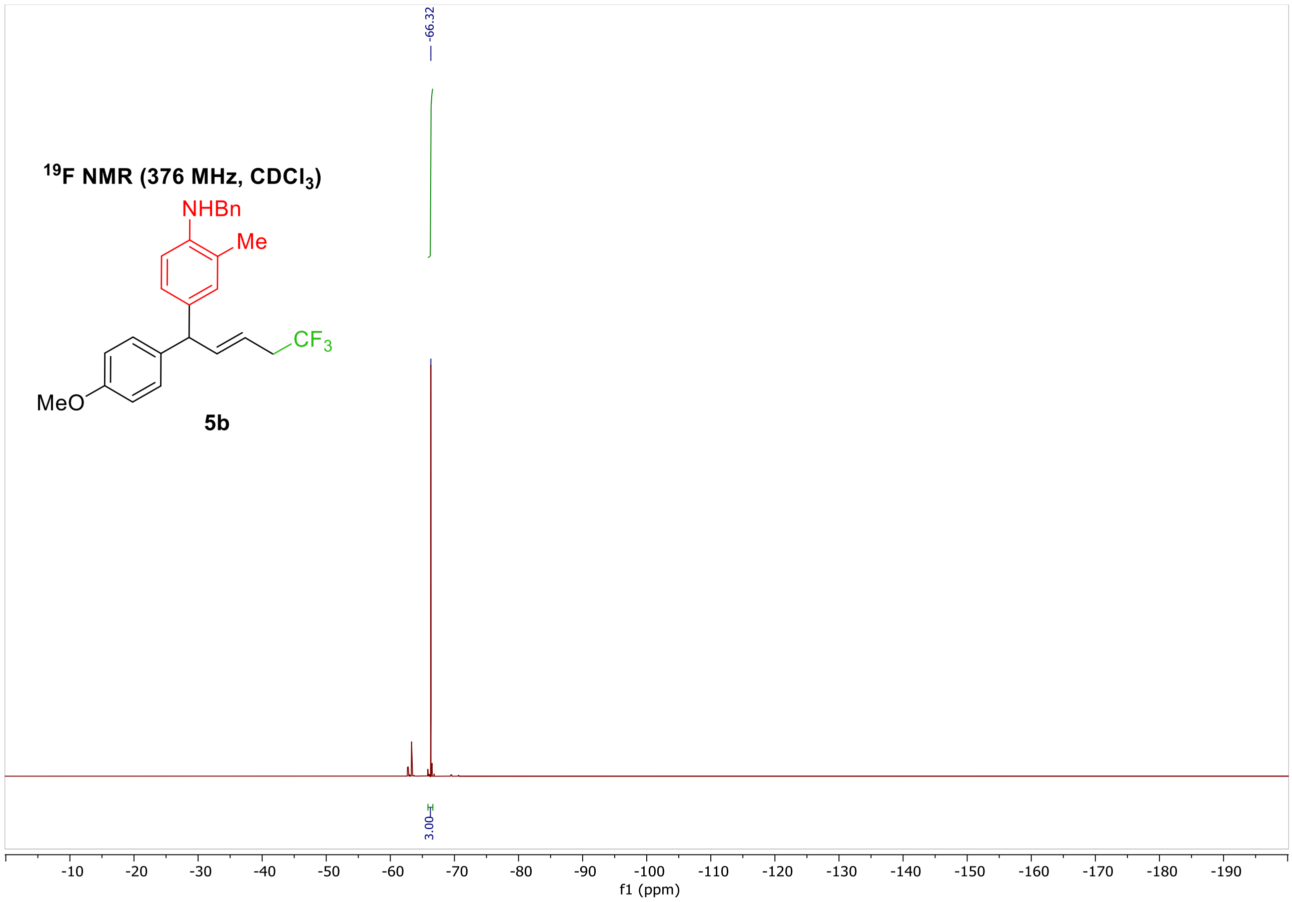
-66.32
19F NMR (376 MHz, CDCl3)
NHBn
Me
CF3
MeO
5b
3.00
f1 (ppm)

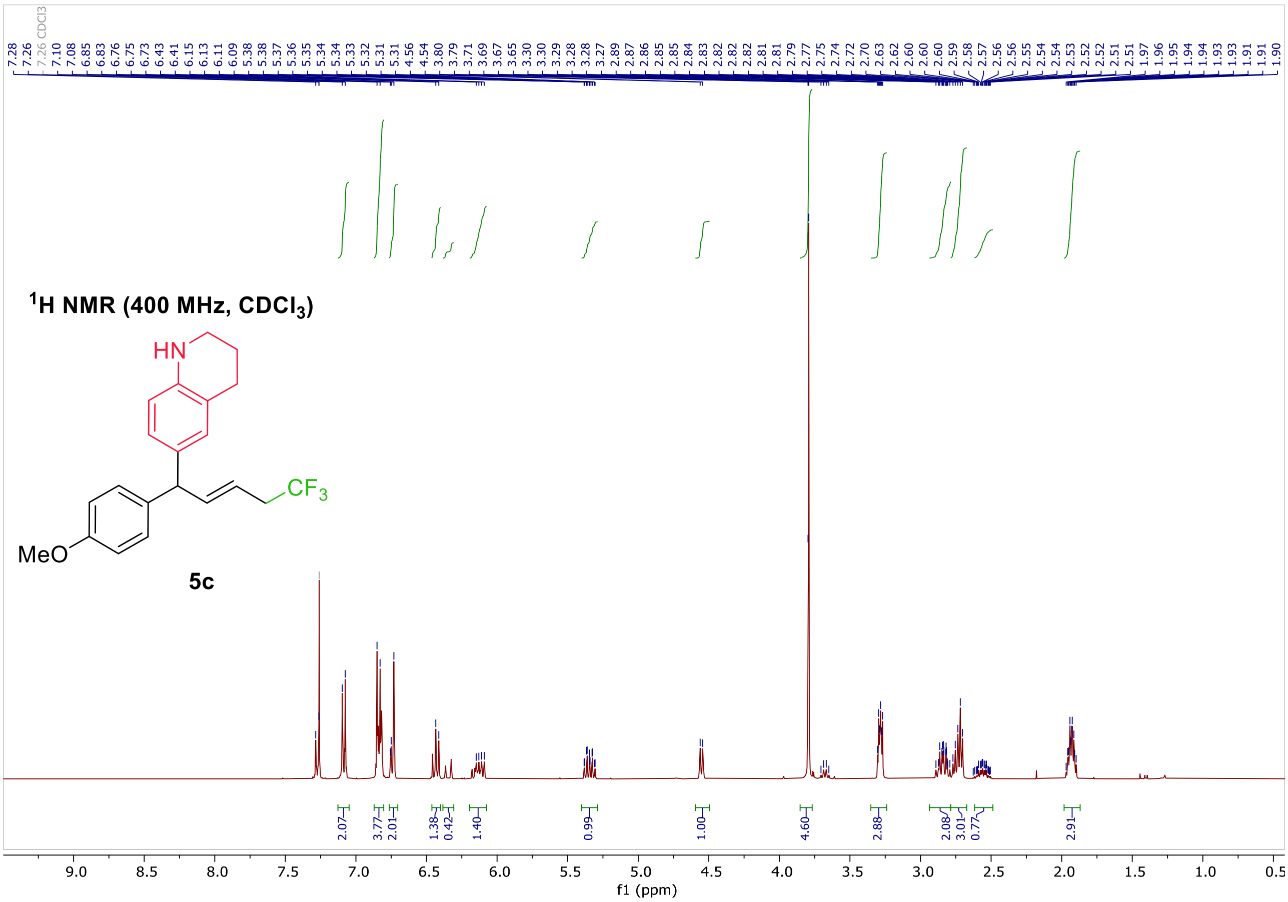
1H NMR (400 MHz, CDCl3)
HN
CF3
MeO
5c
f1 (ppm)

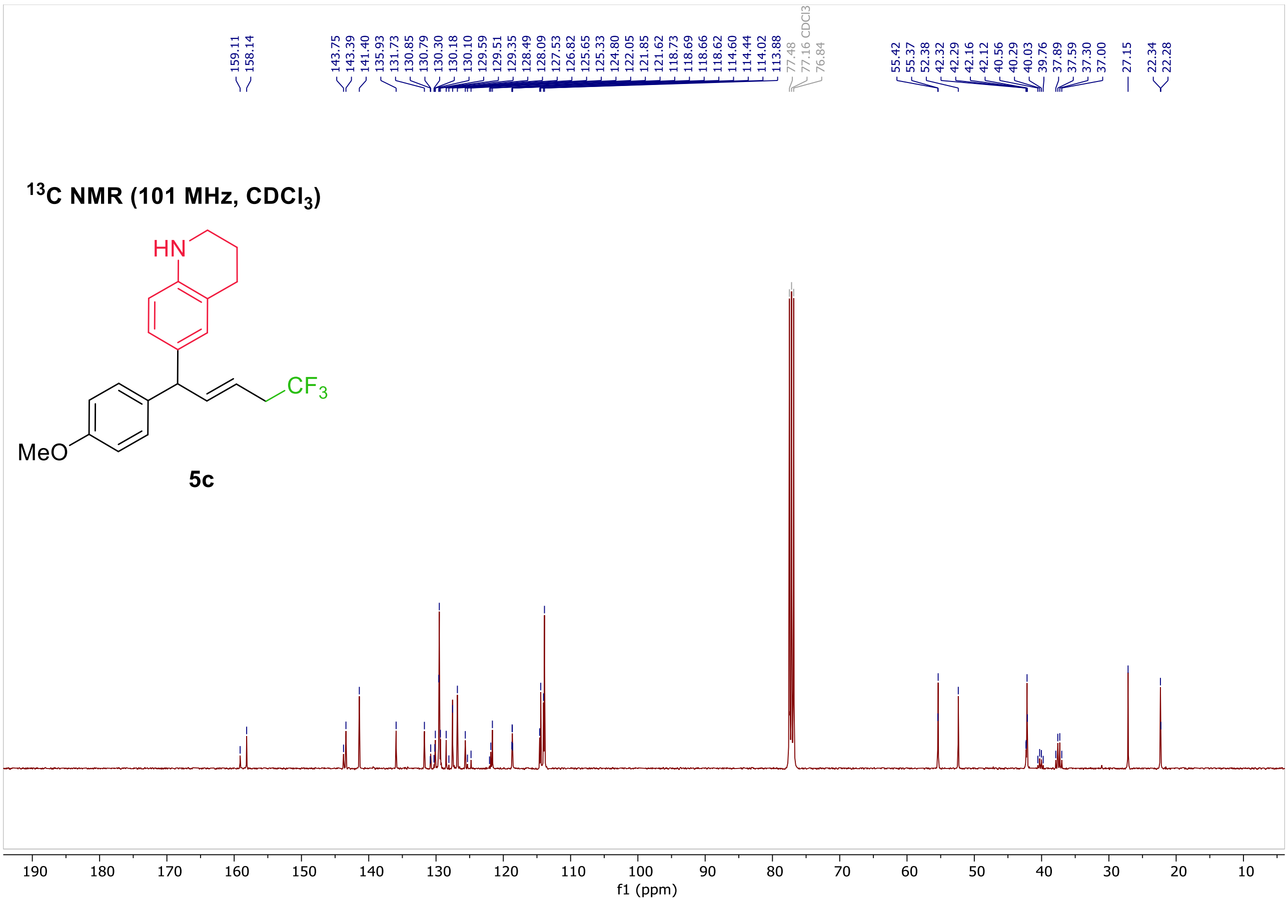
13C NMR (101 MHz, CDCl3)
HN
CF3
MeO
5c
f1 (ppm)

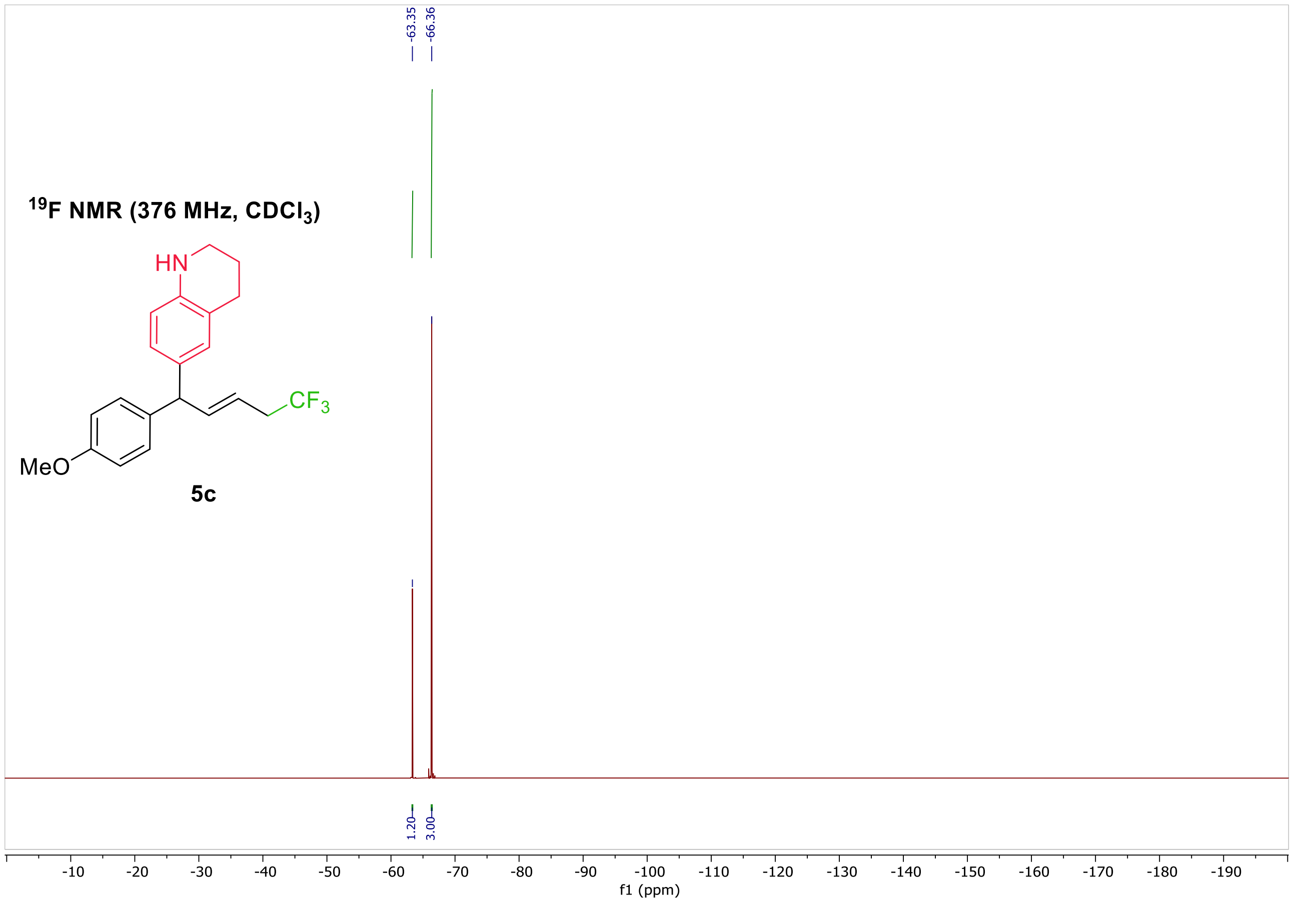
-63.35
-66.36
19F NMR (376 MHz, CDCl3)
HN
CF3
MeO
5c
1.20
3.00
f1 (ppm)

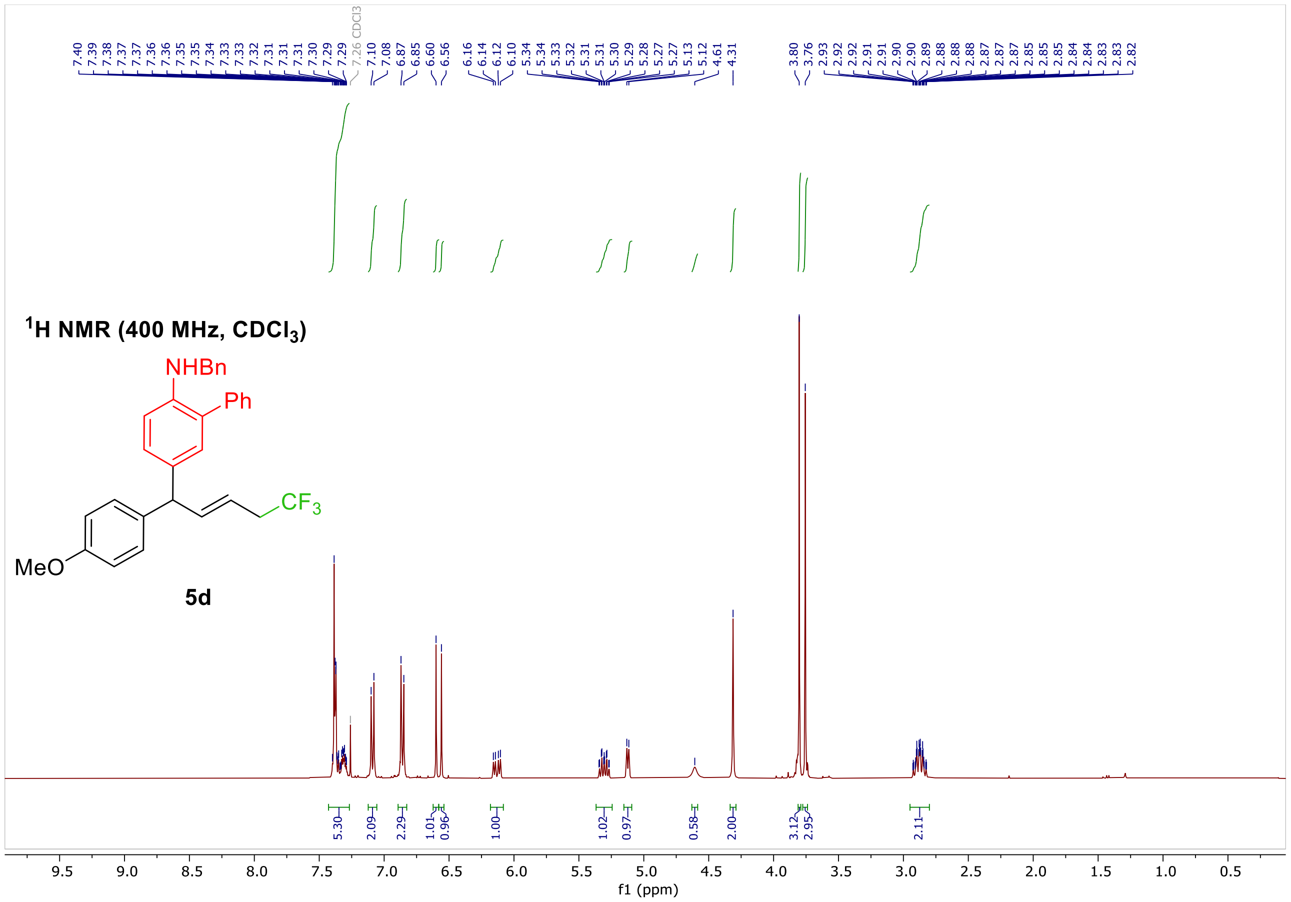
7.40 7.39 7.38 7.37 7.37 7.36 7.36 7.35 7.35 7.34 7.33 7.33 7.32 7.31 7.31 7.31 7.30 7.29 7.29 7.26 CDCl3 7.10 7.08 6.87 6.85 6.60 6.56 6.16 6.14 6.12 6.10 5.34 5.34 5.33 5.32 5.31 5.30 5.29 5.28 5.27 5.27 5.13 5.12 4.61 4.31 3.80 3.76 2.93 2.92 2.92 2.91 2.91 2.90 2.90 2.89 2.88 2.88 2.88 2.87 2.87 2.87 2.85 2.85 2.85 2.84 2.84 2.83 2.83 2.82
1H NMR (400 MHz, CDCl3)
NHBn
Ph
CF3
MeO
5d
5.30 2.09 2.29 1.01 0.96 1.00 1.02 0.97 0.58 2.00 3.12 2.95 2.11
f1 (ppm)

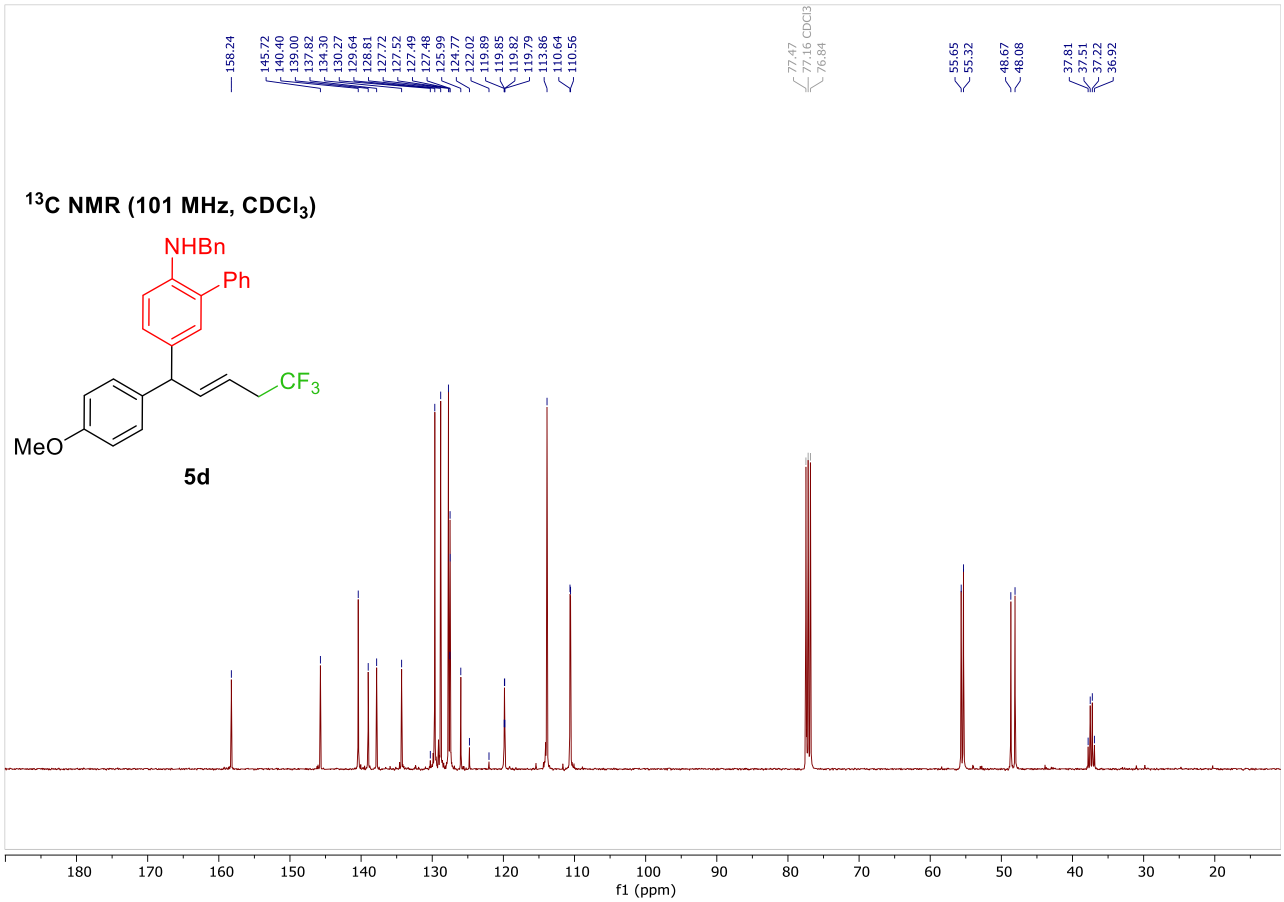

158.24
145.72
140.40
139.00
137.82
134.30
130.27
129.64
128.81
127.72
127.52
127.49
127.48
125.99
124.77
122.02
119.89
119.85
119.82
119.79
113.86
110.64
110.56
77.47
77.16 CDCl3
76.84
55.65
55.32
48.67
48.08
37.81
37.51
37.22
36.92
13C NMR (101 MHz, CDCl3)
NHBn
Ph
CF3
MeO
5d
f1 (ppm)

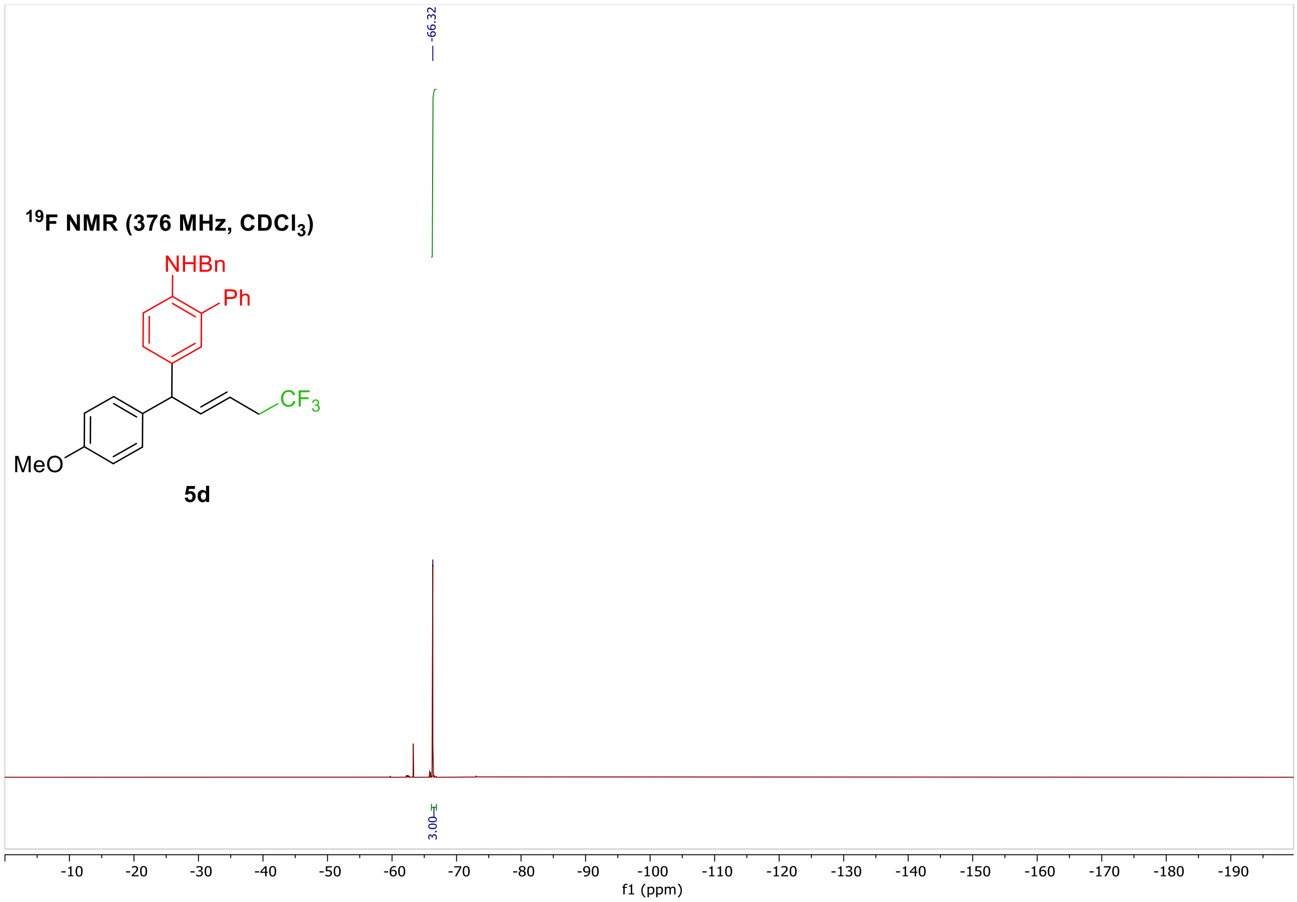

-66.32
19F NMR (376 MHz, CDCl3)
NHBn
Ph
CF3
MeO
5d
3.00
f1 (ppm)

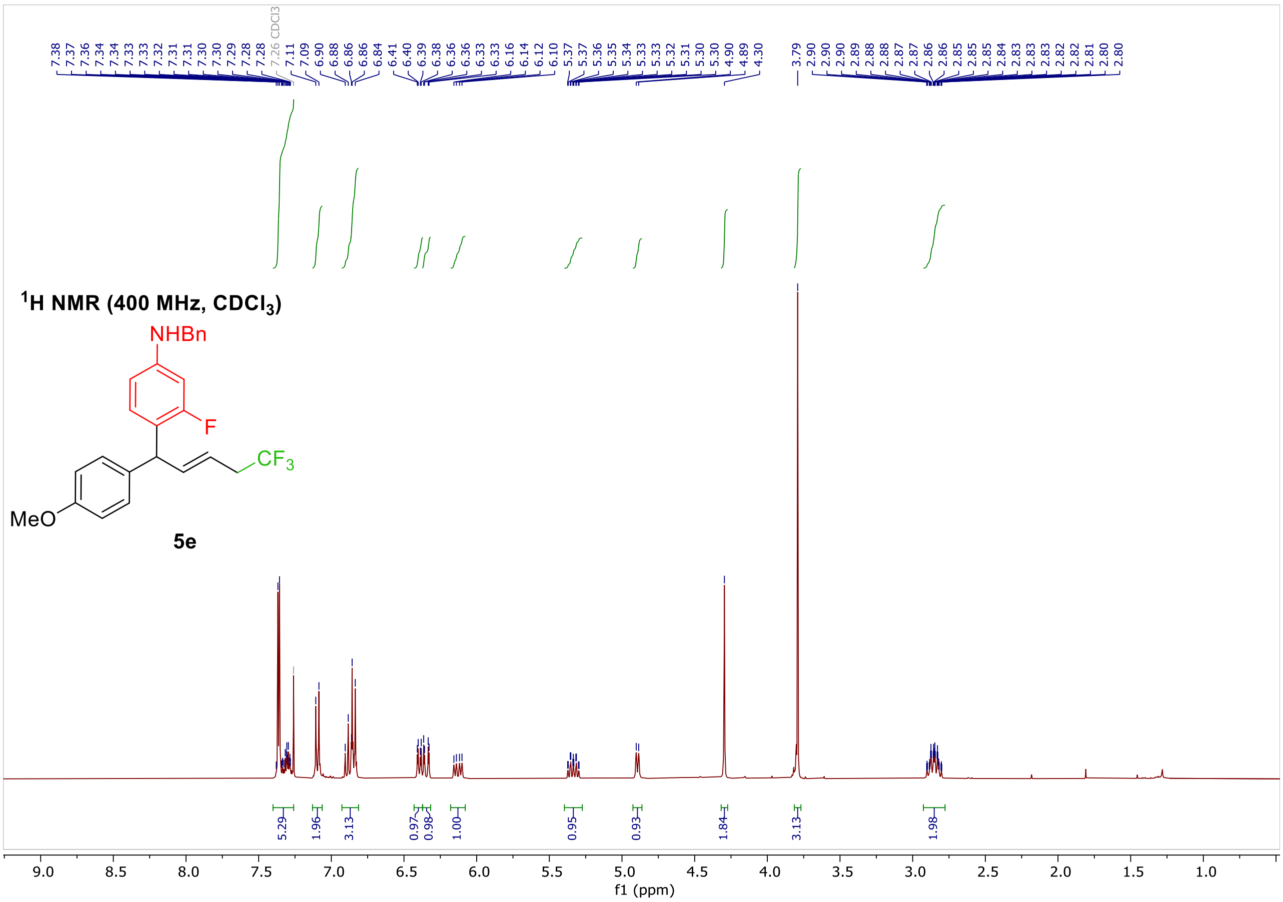

7.38 7.37 7.36 7.34 7.34 7.33 7.33 7.32 7.31 7.31 7.30 7.30 7.29 7.28 7.28 7.26 CDCl3 7.11 7.09 6.90 6.88 6.86 6.86 6.84 6.41 6.40 6.39 6.38 6.36 6.36 6.33 6.33 6.16 6.14 6.12 6.10 5.37 5.37 5.36 5.35 5.34 5.33 5.33 5.32 5.31 5.30 4.90 4.89 4.30 3.79 2.90 2.90 2.89 2.88 2.88 2.87 2.87 2.86 2.86 2.85 2.85 2.85 2.84 2.83 2.83 2.83 2.82 2.82 2.81 2.80 2.80
¹H NMR (400 MHz, CDCl₃)
NHBn
F
CF₃
MeO
5e
5.29 1.96 3.13 0.97 0.98 1.00 0.95 0.93 1.84 3.13 1.98
9.0 8.5 8.0 7.5 7.0 6.5 6.0 5.5 5.0 4.5 4.0 3.5 3.0 2.5 2.0 1.5 1.0
f1 (ppm)

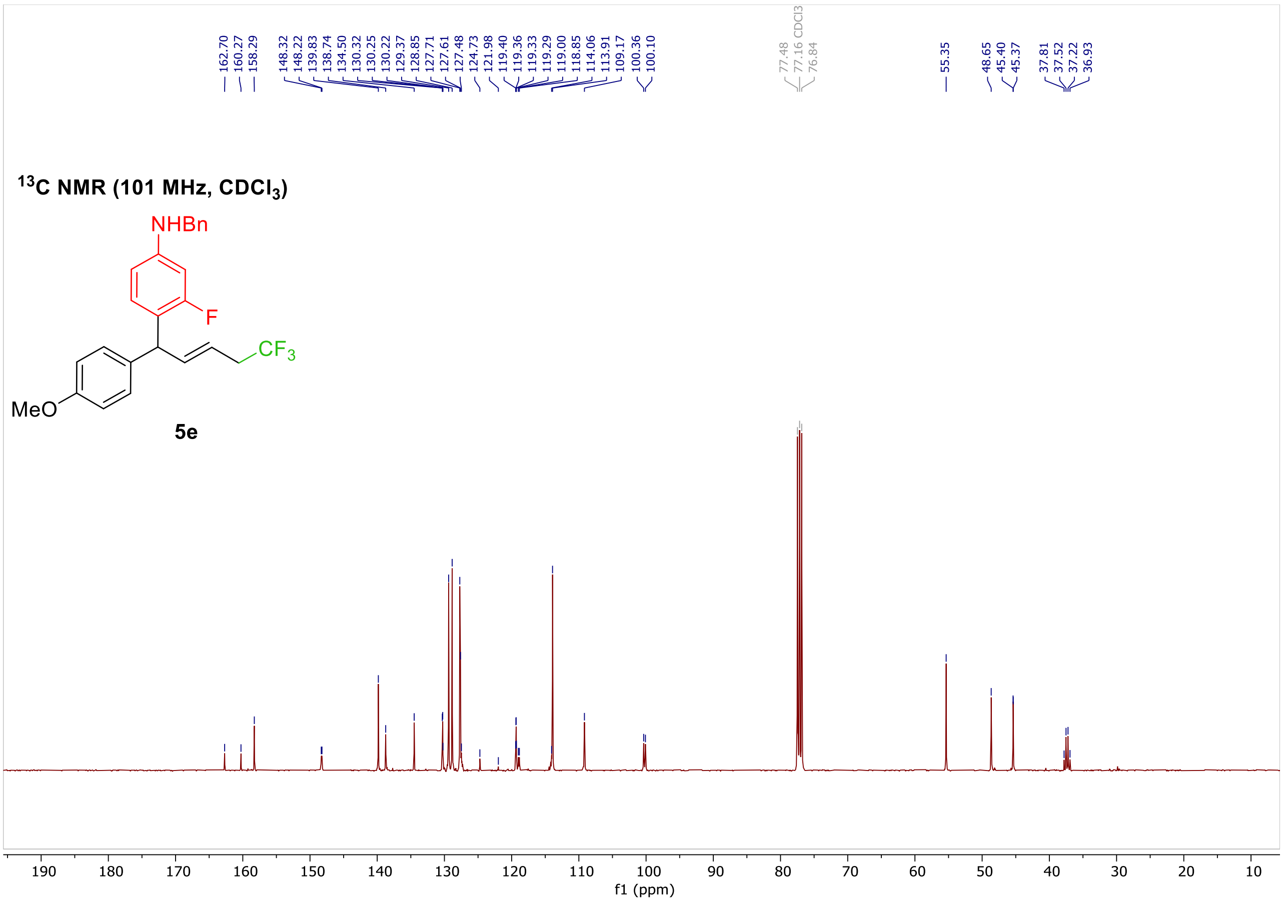

162.70 160.27 158.29 148.32 148.22 139.83 138.74 134.50 130.32 130.25 130.22 129.37 128.85 127.71 127.61 127.48 124.73 121.98 119.40 119.36 119.33 119.29 119.00 118.85 114.06 113.91 109.17 100.36 100.10 77.48 77.16 CDCl3 76.84 55.35 48.65 45.40 45.37 37.81 37.52 37.22 36.93
¹³C NMR (101 MHz, CDCl₃)
NHBn
F
CF₃
MeO
5e
190 180 170 160 150 140 130 120 110 100 90 80 70 60 50 40 30 20 10
f1 (ppm)

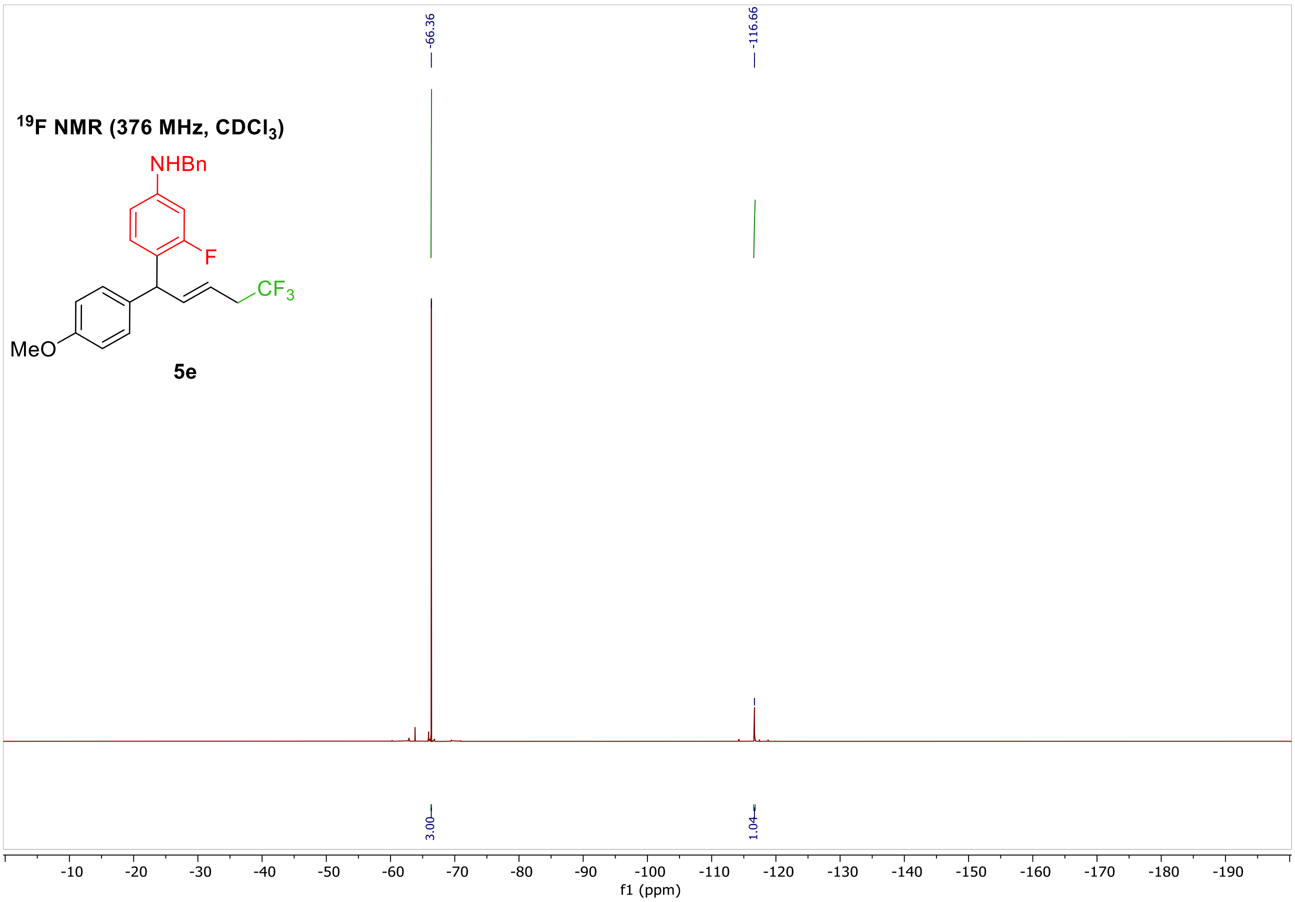
19F NMR (376 MHz, CDCl3)
NHBn
F
CF3
MeO
5e
-66.36
-116.66
3.00
1.04
f1 (ppm)

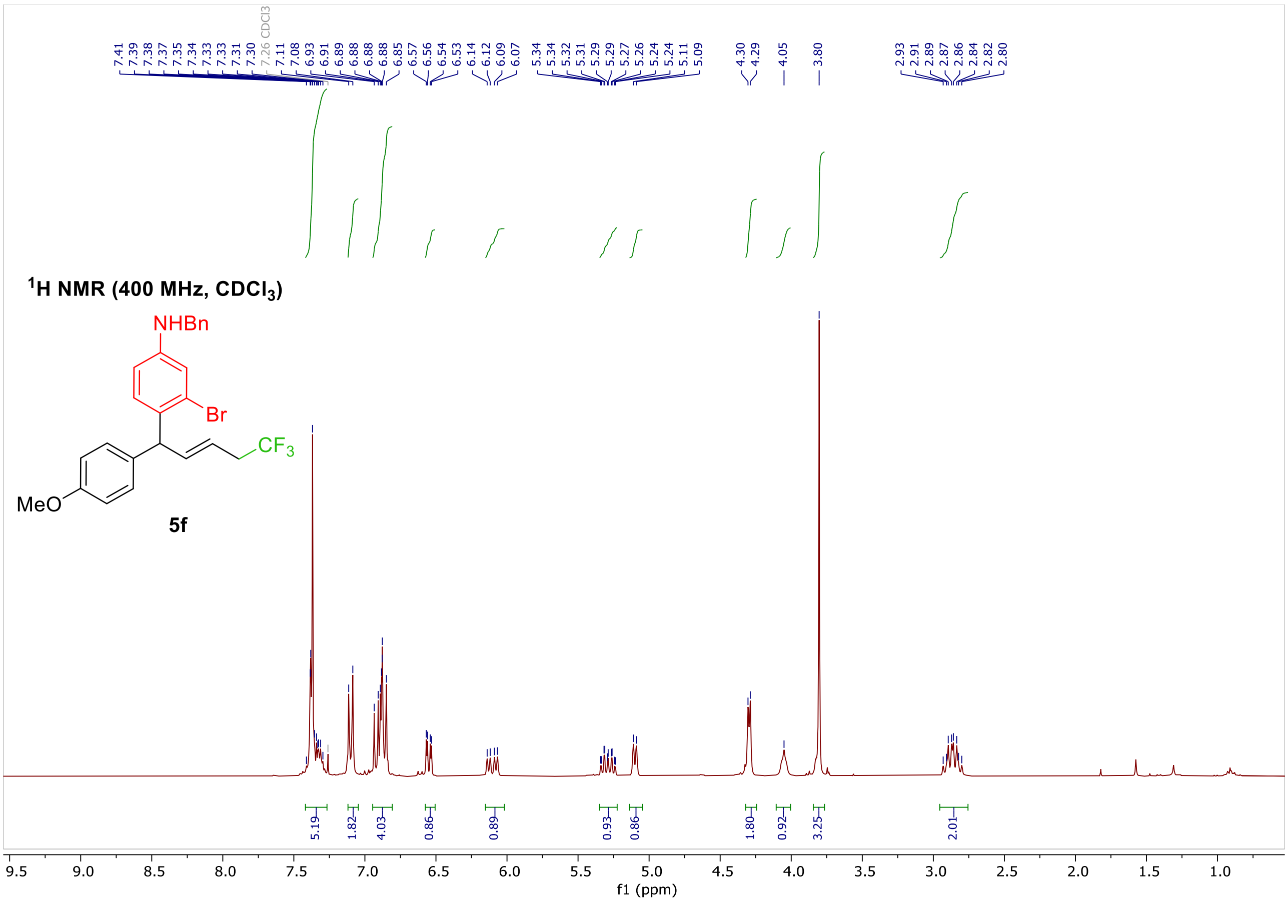
1H NMR (400 MHz, CDCl3)
NHBn
Br
CF3
MeO
5f
7.26 CDCl3
f1 (ppm)

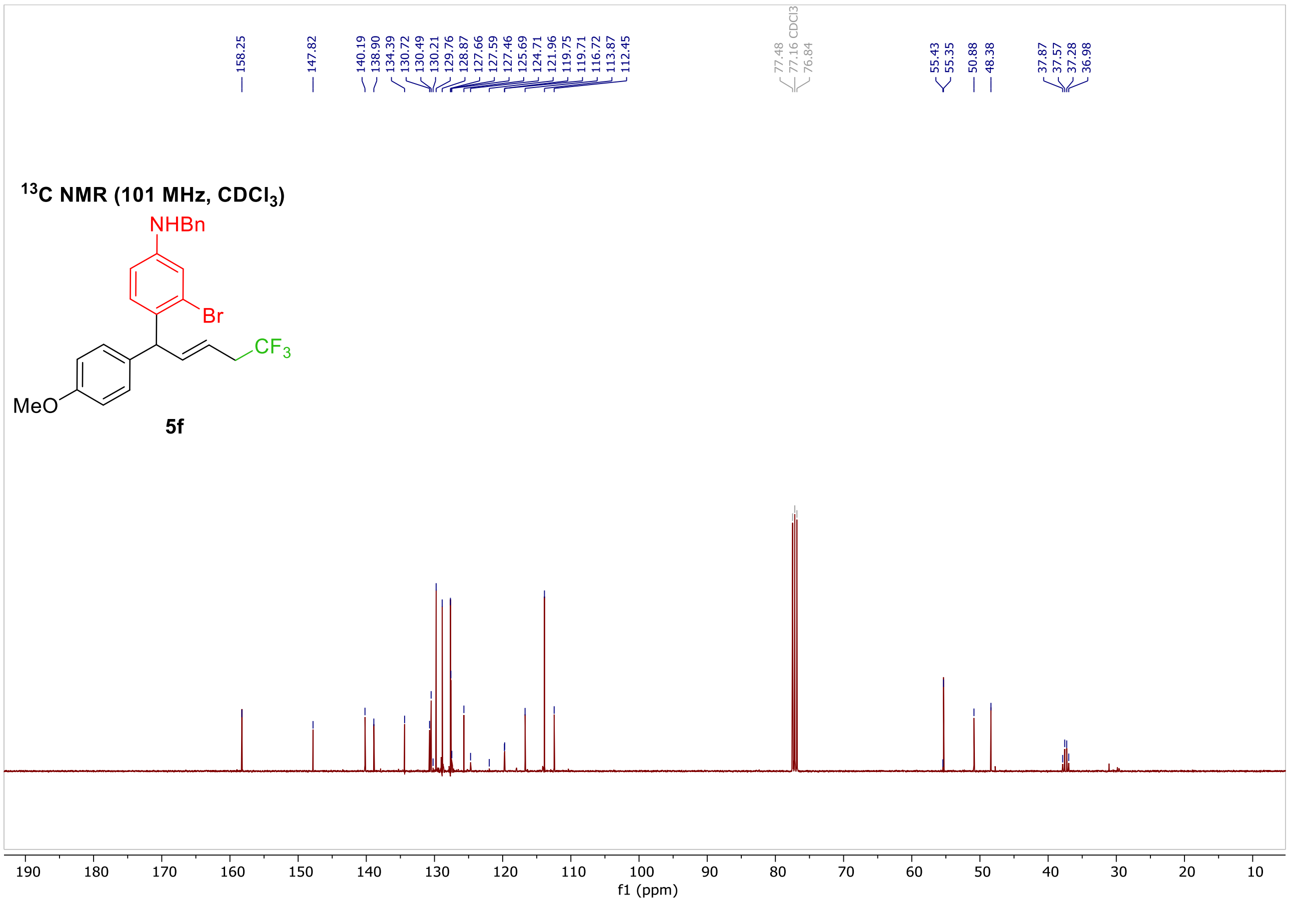
158.25
147.82
140.19
138.90
134.39
130.72
130.49
130.21
129.76
128.87
127.66
127.59
127.46
125.69
124.71
121.96
119.75
119.71
116.72
113.87
112.45
77.48
77.16 CDCl3
76.84
55.43
55.35
50.88
48.38
37.87
37.57
37.28
36.98
^{13}C NMR (101 MHz, $CDCl_3$)
NHBn
Br
CF_3
MeO
5f
f1 (ppm)

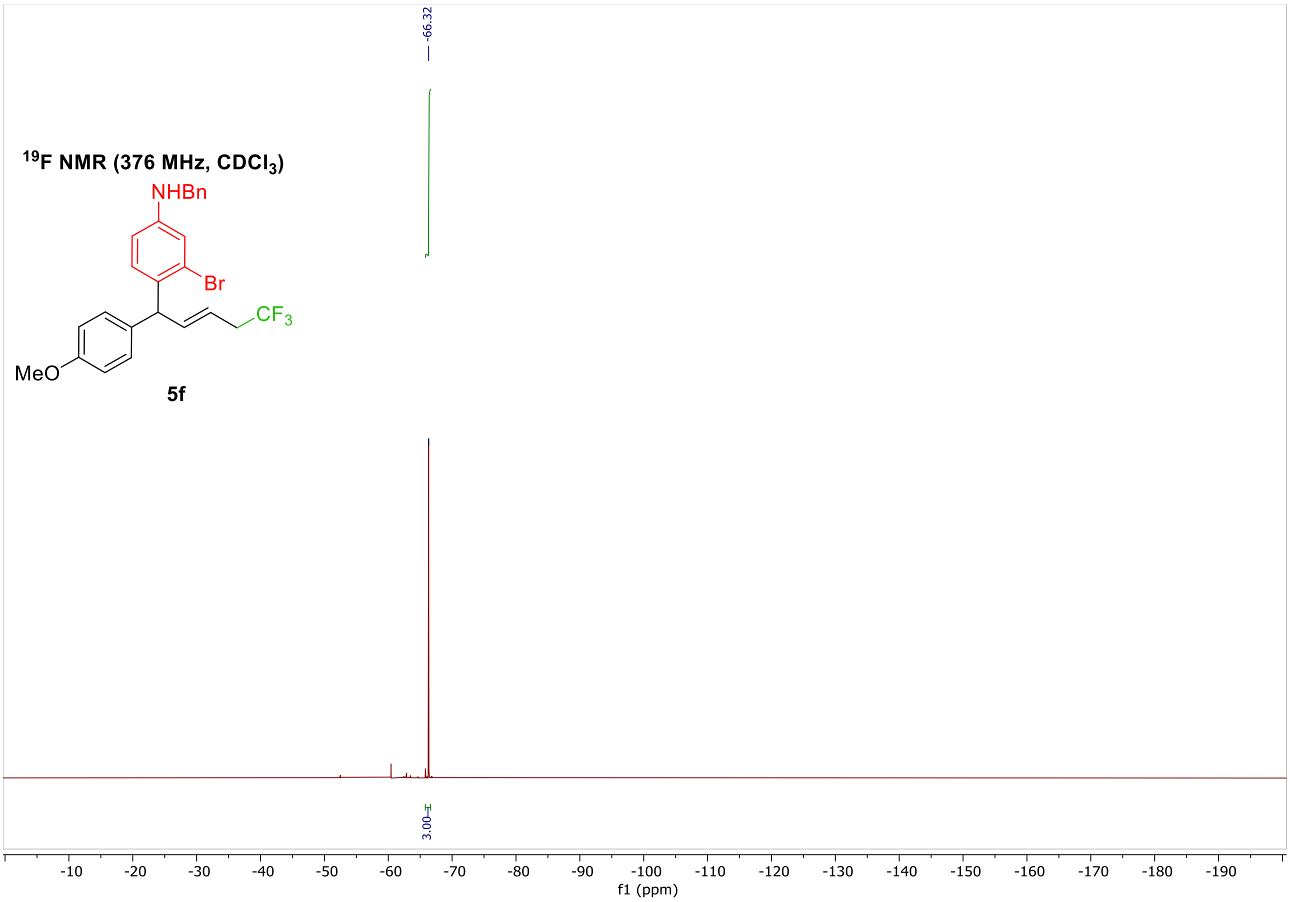
-66.32
^{19}F NMR (376 MHz, $CDCl_3$)
NHBn
Br
CF_3
MeO
5f
3.00
f1 (ppm)

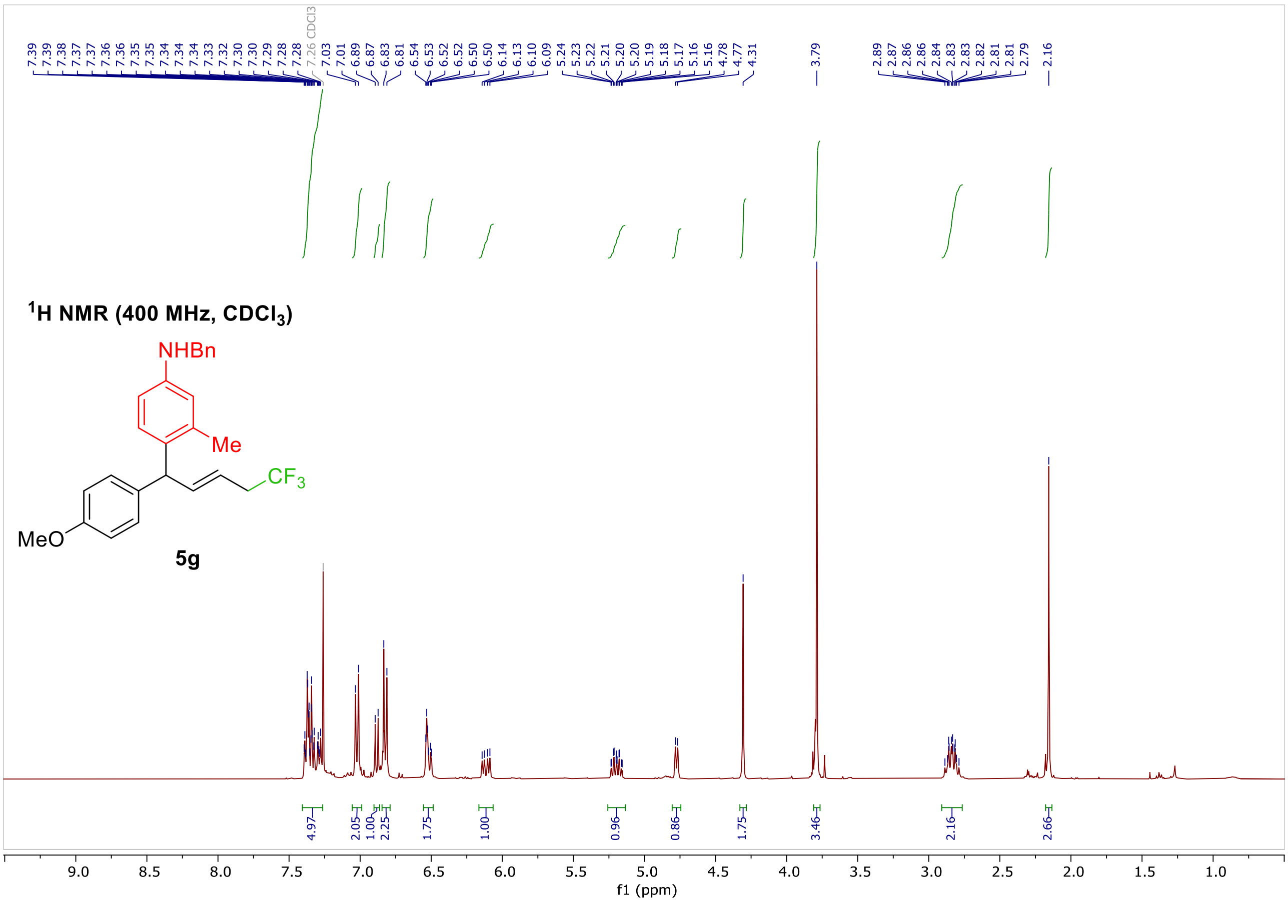

7.39 7.39 7.38 7.37 7.37 7.36 7.36 7.35 7.35 7.34 7.34 7.34 7.33 7.32 7.30 7.30 7.29 7.28 7.28 7.26 CDCl3 7.03 7.01 6.89 6.87 6.83 6.81 6.54 6.53 6.52 6.52 6.50 6.50 6.14 6.13 6.10 6.09 5.24 5.23 5.22 5.21 5.20 5.20 5.19 5.18 5.17 5.16 5.16 4.78 4.77 4.31 3.79 2.89 2.87 2.86 2.86 2.84 2.83 2.83 2.82 2.81 2.81 2.79 2.16
^{1}H NMR (400 MHz, CDCl$_3$)
NHBn
Me
CF$_3$
MeO
5g
4.97 2.05 1.00 2.25 1.75 1.00 0.96 0.86 1.75 3.46 2.16 2.66
9.0 8.5 8.0 7.5 7.0 6.5 6.0 5.5 5.0 4.5 4.0 3.5 3.0 2.5 2.0 1.5 1.0
f1 (ppm)

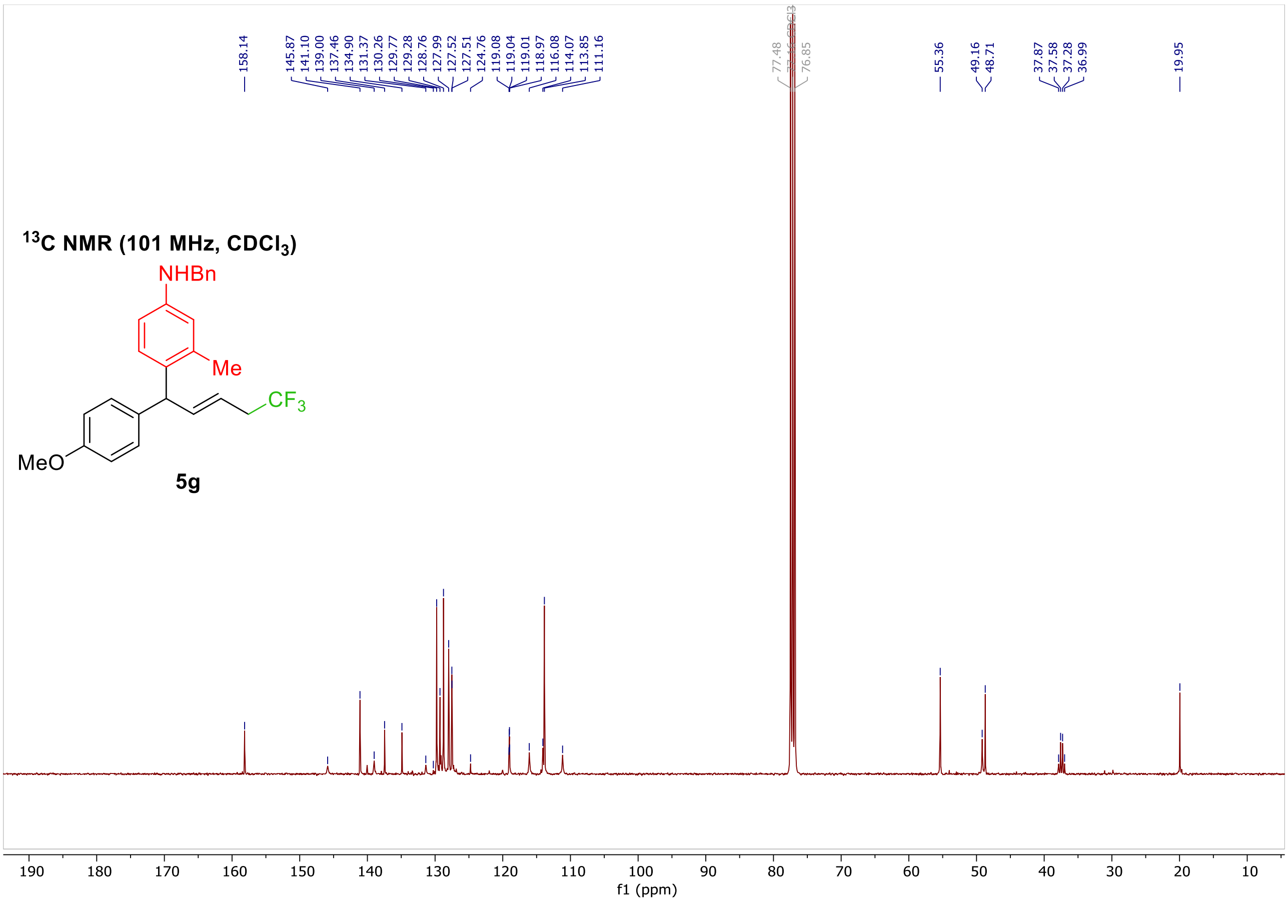

158.14 145.87 141.10 139.00 137.46 134.90 131.37 130.26 129.77 129.28 128.76 127.99 127.52 127.51 124.76 119.08 119.04 119.01 118.97 116.08 114.07 113.85 111.16 77.48 77.16 CDCl3 76.85 55.36 49.16 48.71 37.87 37.58 37.28 36.99 19.95
^{13}C NMR (101 MHz, CDCl$_3$)
NHBn
Me
CF$_3$
MeO
5g
190 180 170 160 150 140 130 120 110 100 90 80 70 60 50 40 30 20 10
f1 (ppm)

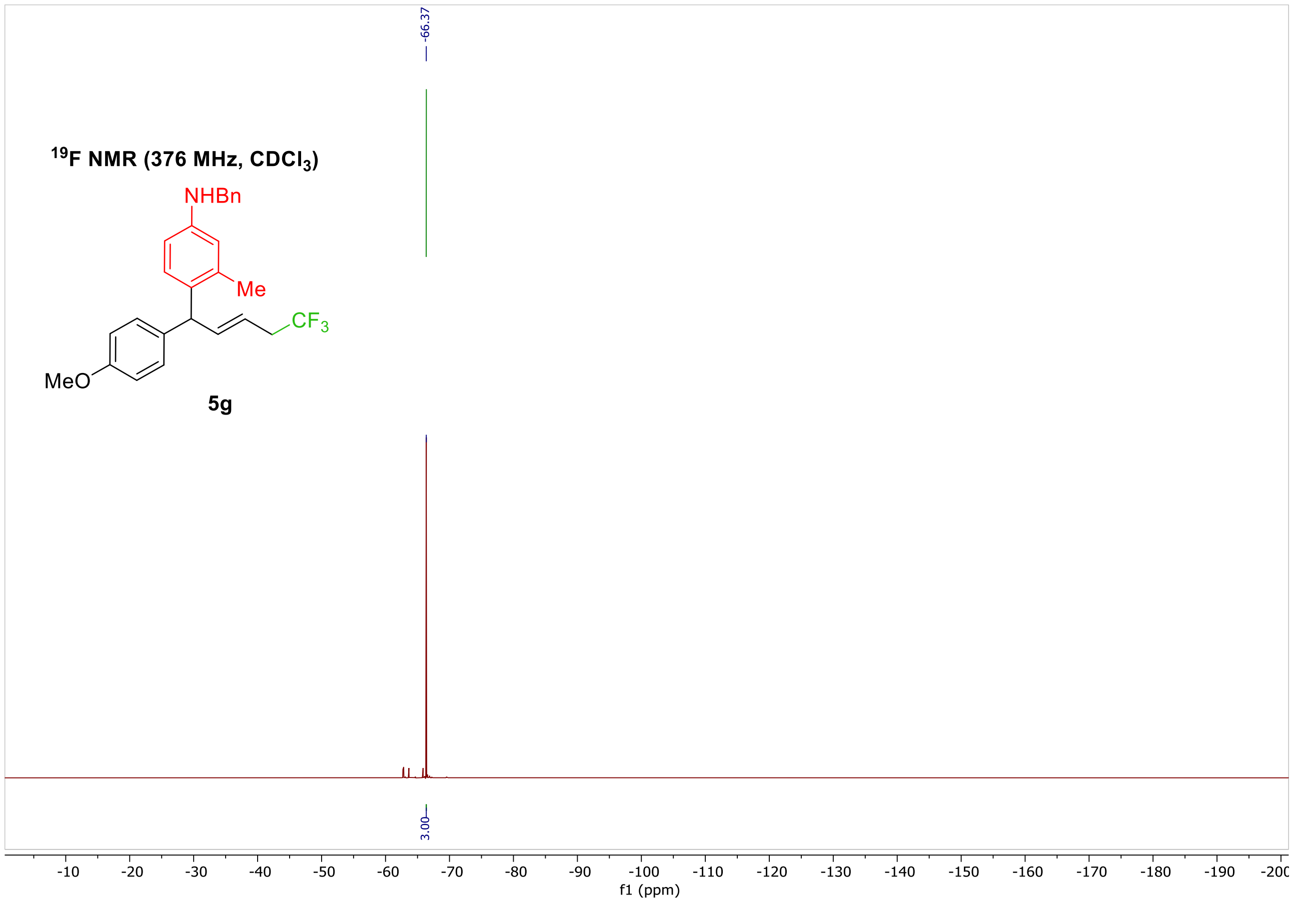

19F NMR (376 MHz, CDCl3)
NHBn
Me
CF3
MeO
5g
-66.37
3.00
f1 (ppm)

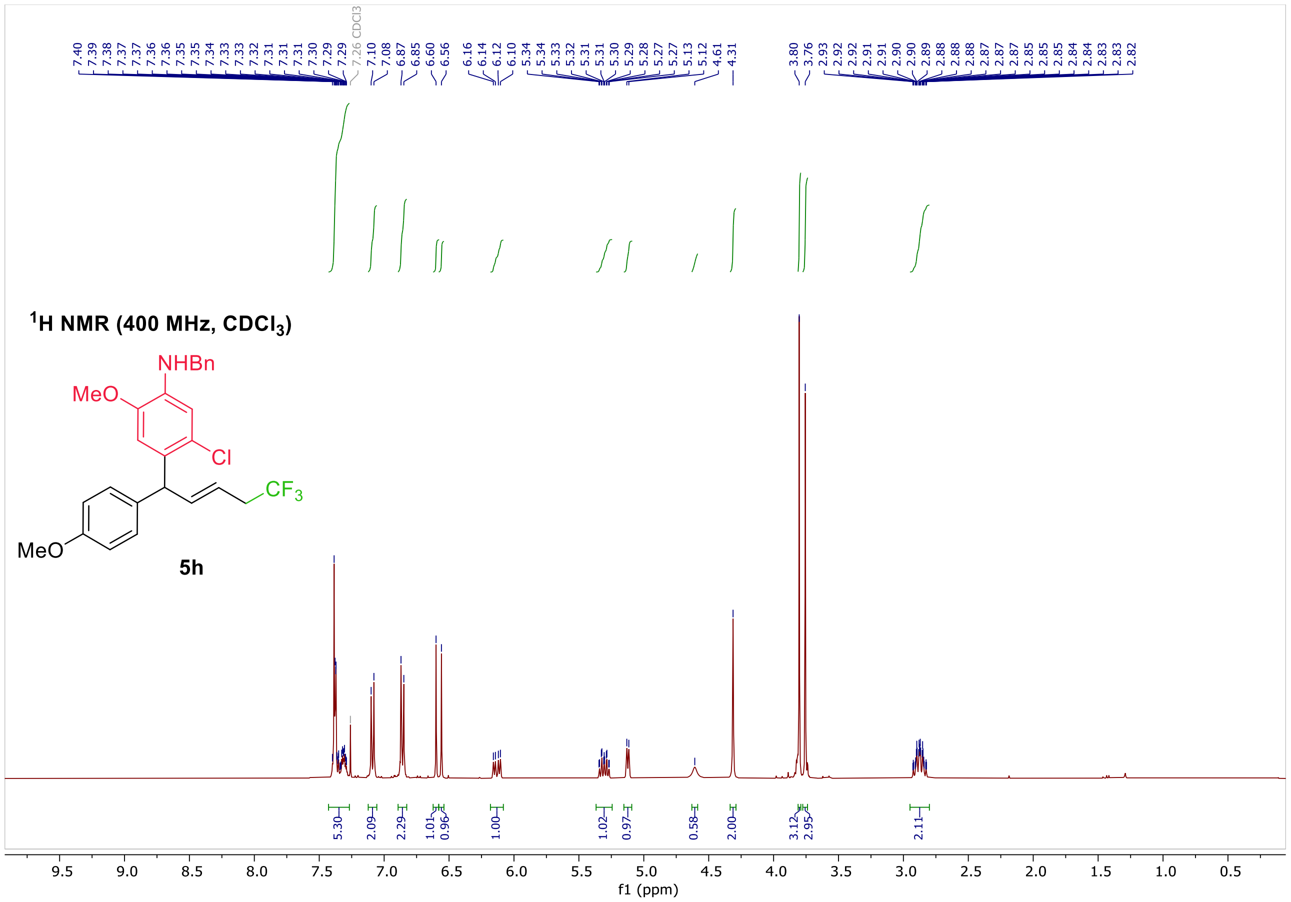

1H NMR (400 MHz, CDCl3)
NHBn
MeO
Cl
CF3
MeO
5h
7.26 CDCl3
f1 (ppm)

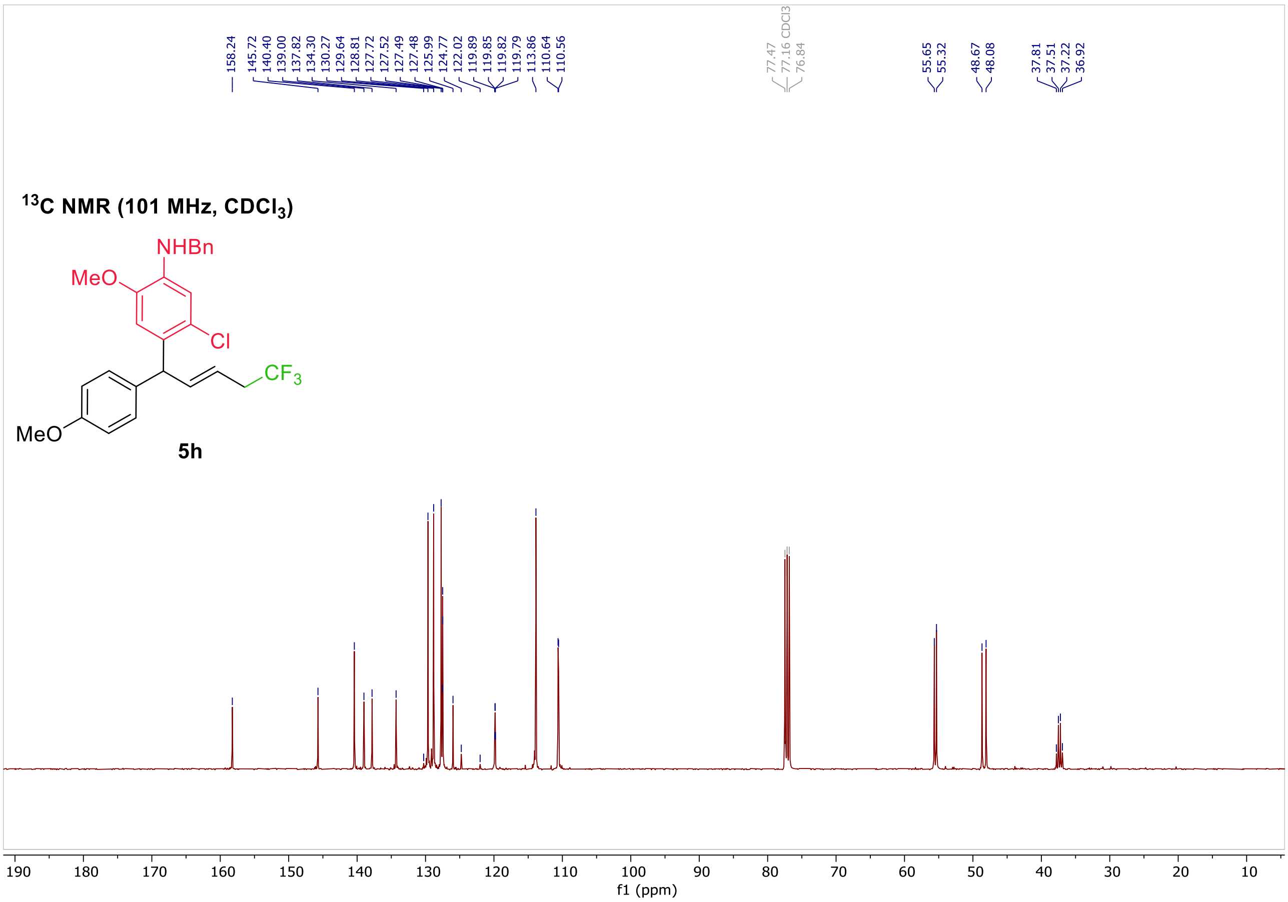

158.24
145.72
140.40
139.00
137.82
134.30
130.27
129.64
128.81
127.72
127.52
127.49
127.48
125.99
124.77
122.02
119.89
119.85
119.82
119.79
113.86
110.64
110.56
77.47
77.16 CDCl3
76.84
55.65
55.32
48.67
48.08
37.81
37.51
37.22
36.92
13C NMR (101 MHz, CDCl3)
NHBn
MeO
Cl
CF3
MeO
5h
f1 (ppm)

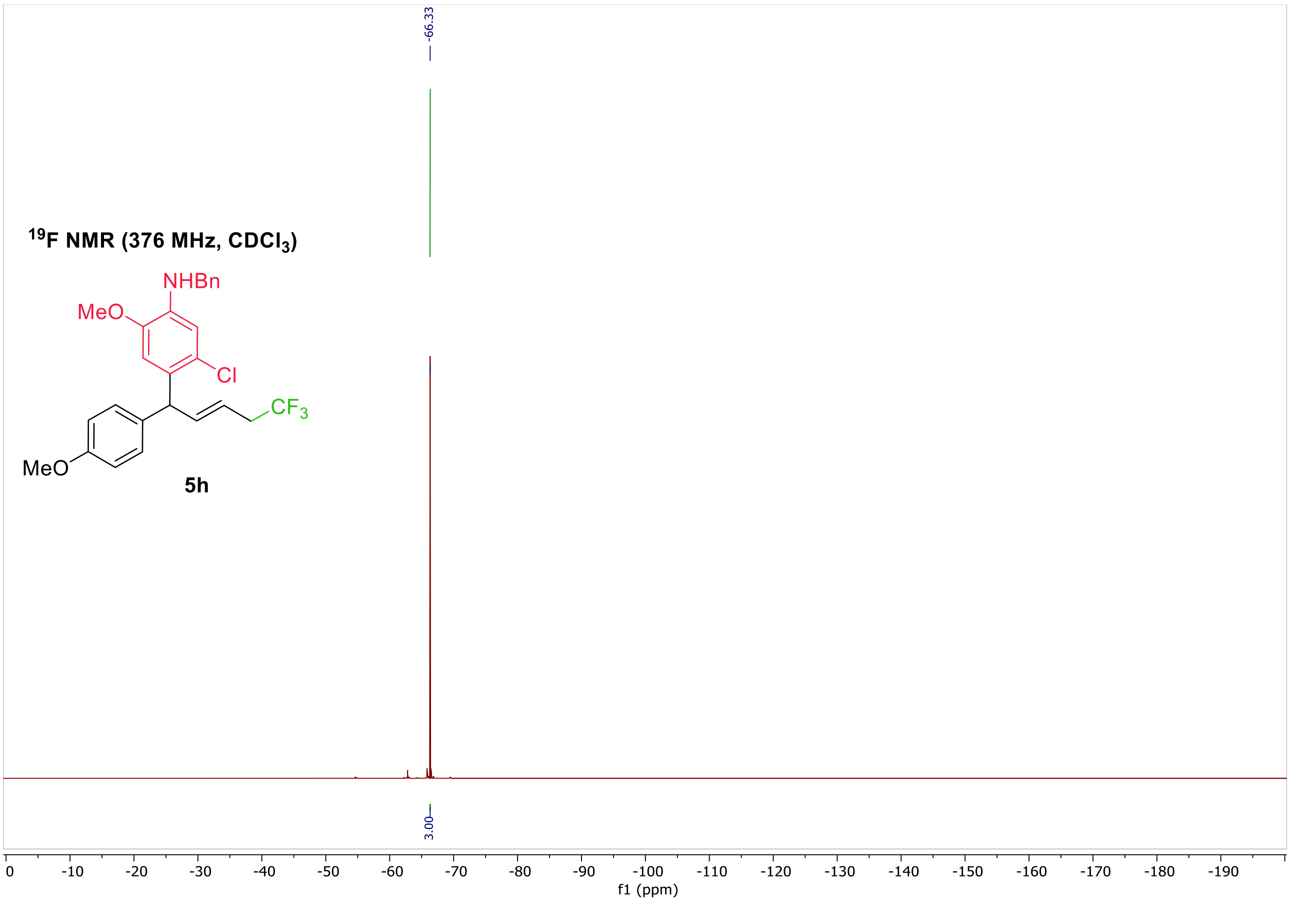

-66.33
19F NMR (376 MHz, CDCl3)
NHBn
MeO
Cl
CF3
MeO
5h
3.00
f1 (ppm)

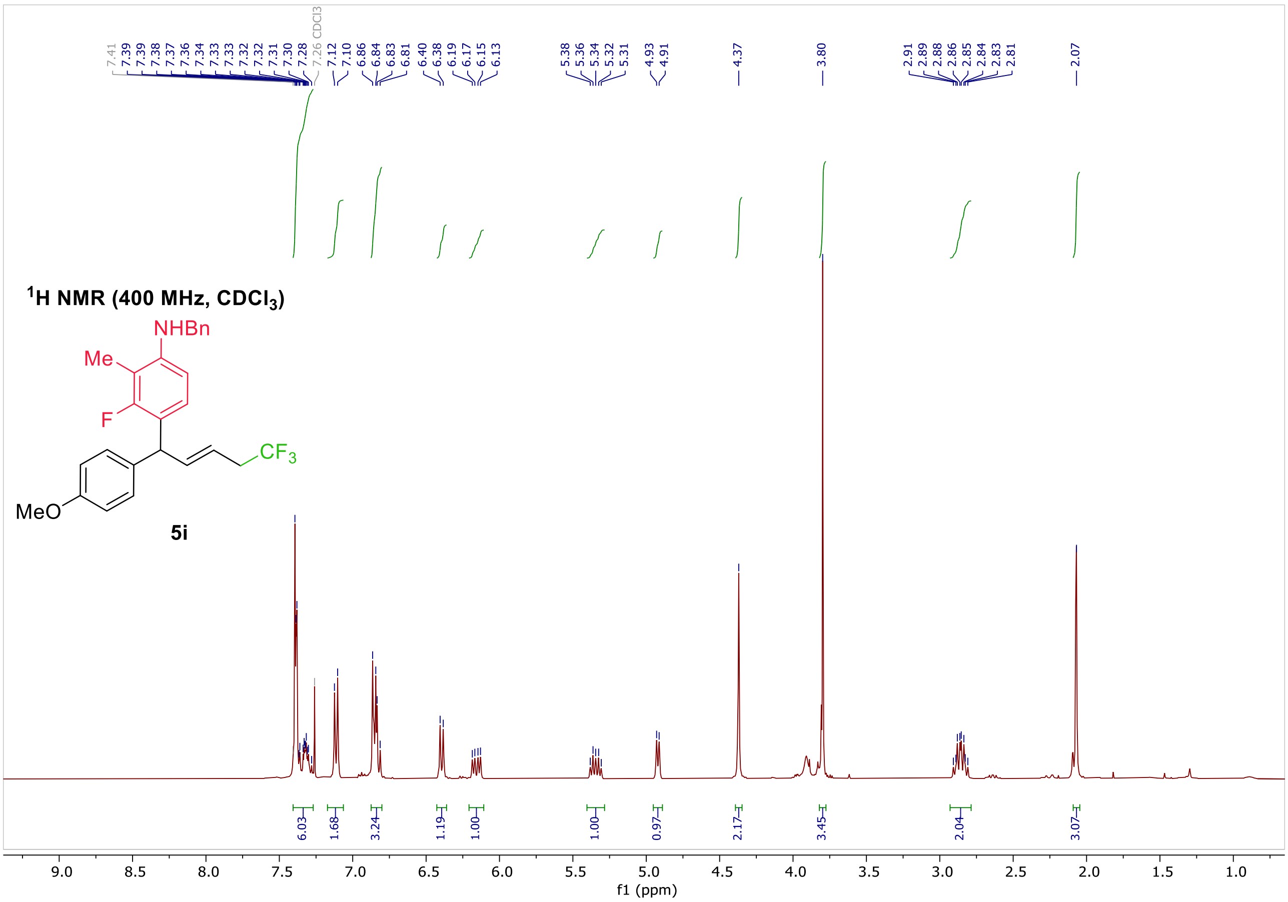

¹H NMR (400 MHz, CDCl3)
NHBn
Me
F
CF3
MeO
5i
7.41
7.39
7.39
7.38
7.37
7.36
7.34
7.33
7.33
7.32
7.32
7.31
7.30
7.28
7.26 CDCl3
7.12
7.10
6.86
6.84
6.83
6.81
6.40
6.38
6.19
6.17
6.15
6.13
5.38
5.36
5.34
5.32
5.31
4.93
4.91
4.37
3.80
2.91
2.89
2.88
2.86
2.85
2.84
2.83
2.81
2.07
6.03
1.68
3.24
1.19
1.00
1.00
0.97
2.17
3.45
2.04
3.07
9.0
8.5
8.0
7.5
7.0
6.5
6.0
5.5
5.0
4.5
4.0
3.5
3.0
2.5
2.0
1.5
1.0
f1 (ppm)

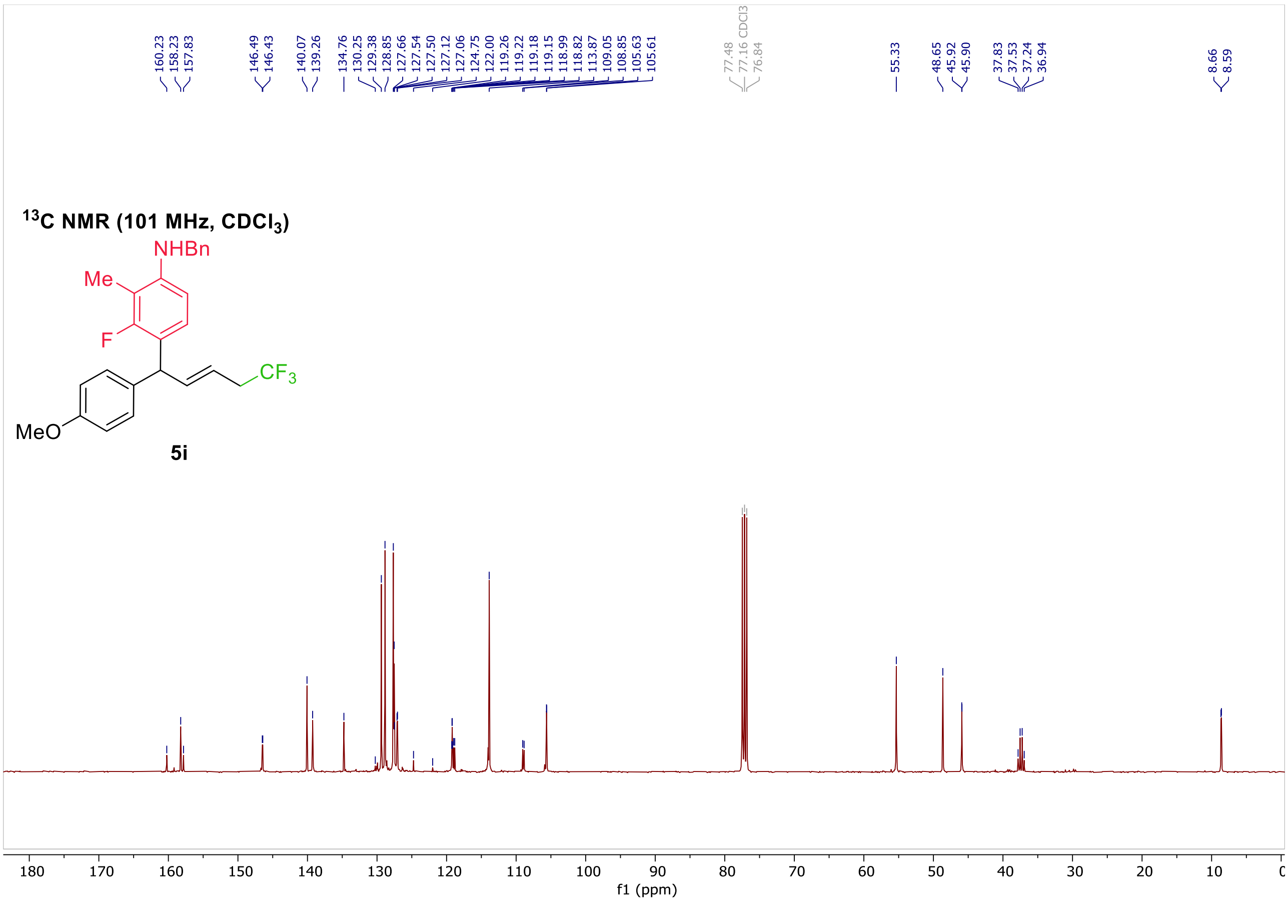

¹³C NMR (101 MHz, CDCl3)
NHBn
Me
F
CF3
MeO
5i
160.23
158.23
157.83
146.49
146.43
140.07
139.26
134.76
130.25
129.38
128.85
127.66
127.54
127.50
127.12
127.06
124.75
122.00
119.26
119.22
119.18
119.15
118.99
118.82
113.87
109.05
108.85
105.63
105.61
77.48
77.16 CDCl3
76.84
55.33
48.65
45.92
45.90
37.83
37.53
37.24
36.94
8.66
8.59
180
170
160
150
140
130
120
110
100
90
80
70
60
50
40
30
20
10
0
f1 (ppm)

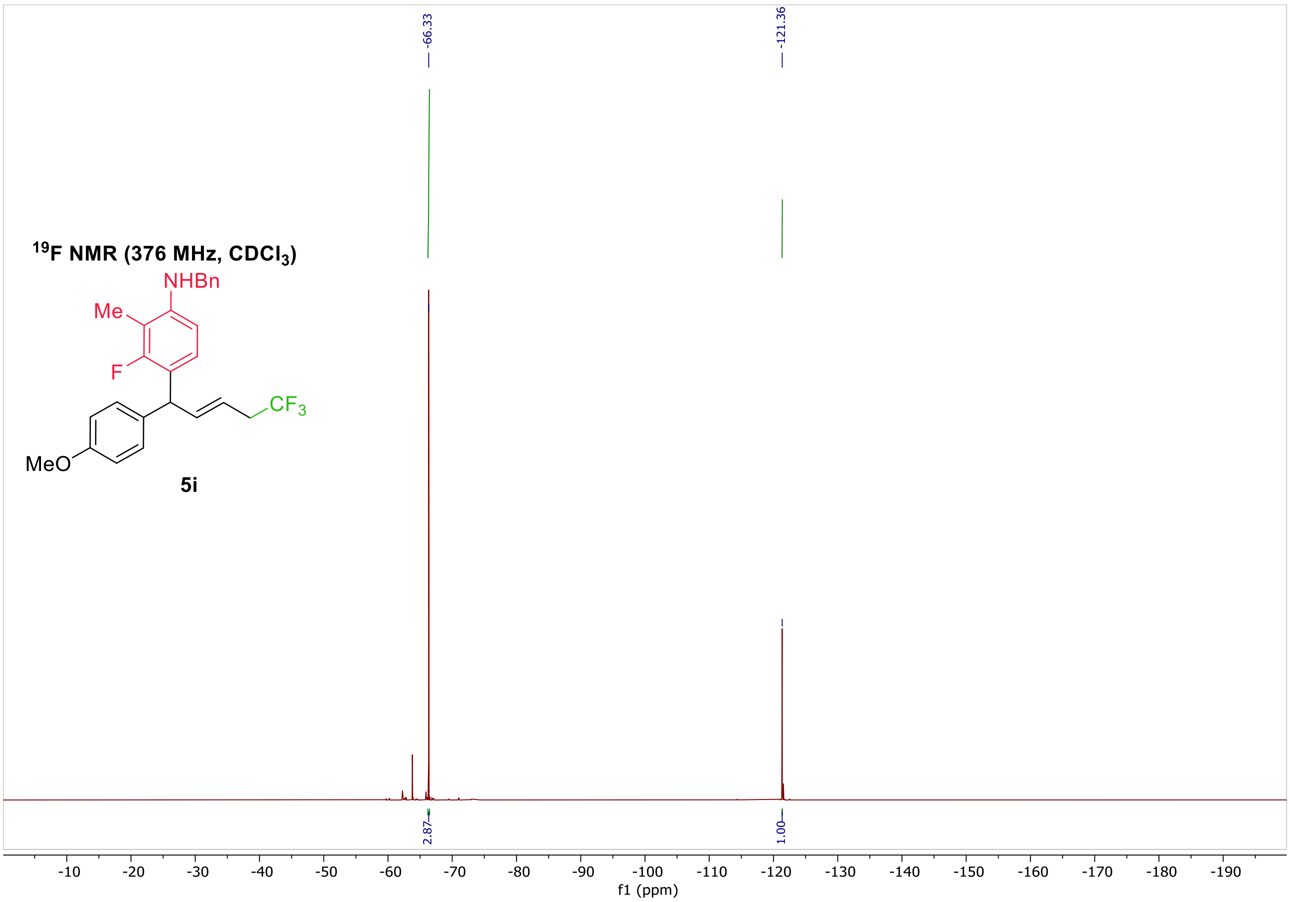

19F NMR (376 MHz, CDCl3)
NHBn
Me
F
CF3
MeO
5i
-66.33
-121.36
2.87
1.00
f1 (ppm)

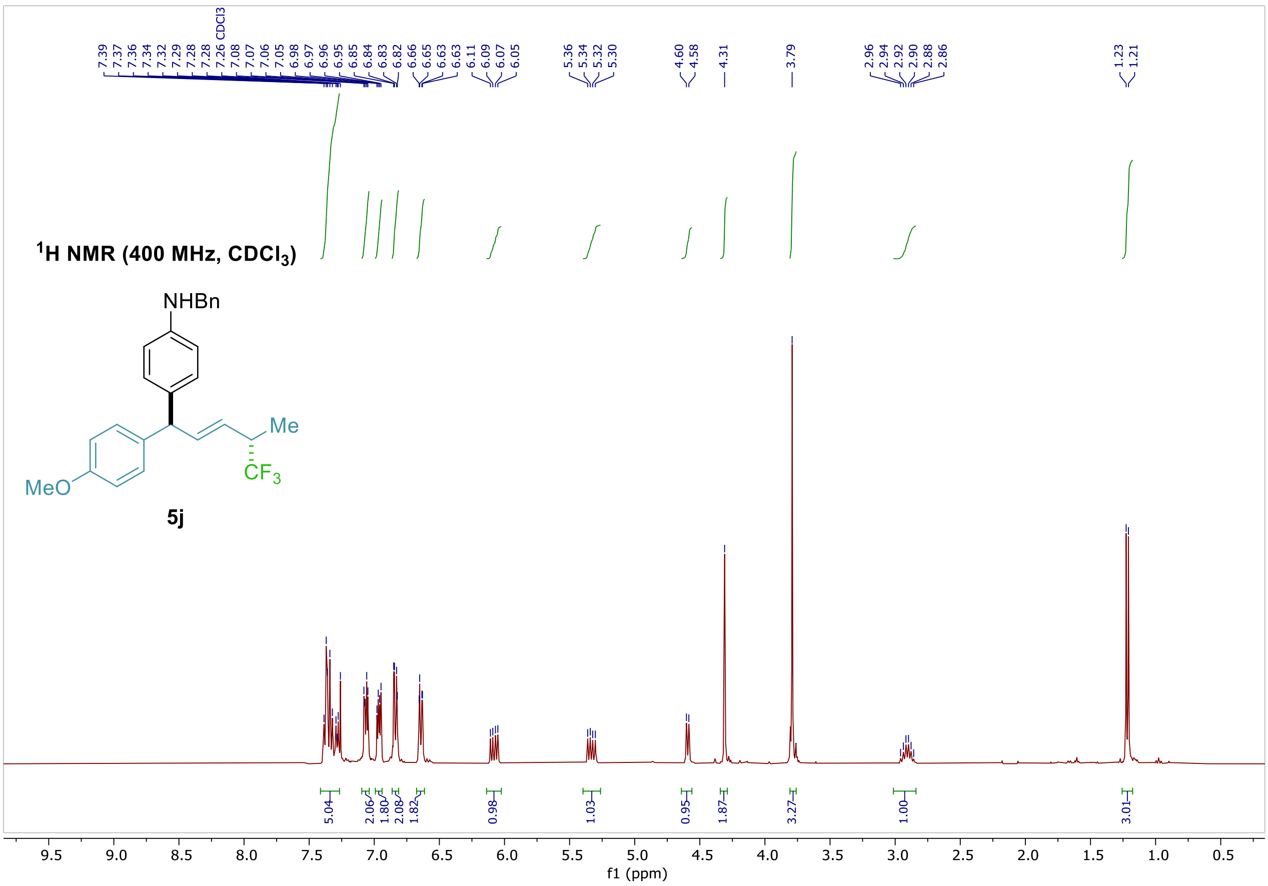

1H NMR (400 MHz, CDCl3)
NHBn
Me
CF3
MeO
5j
7.39 7.37 7.36 7.34 7.32 7.29 7.28 7.26 CDCl3 7.08 7.07 7.06 7.05 6.98 6.97 6.96 6.95 6.85 6.84 6.83 6.82 6.66 6.65 6.63 6.63 6.11 6.09 6.07 6.05 5.36 5.34 5.32 5.30 4.60 4.58 4.31 3.79 2.96 2.94 2.92 2.90 2.88 2.86 1.23 1.21
5.04 2.06 1.80 2.08 1.82 0.98 1.03 0.95 1.87 3.27 1.00 3.01
f1 (ppm)

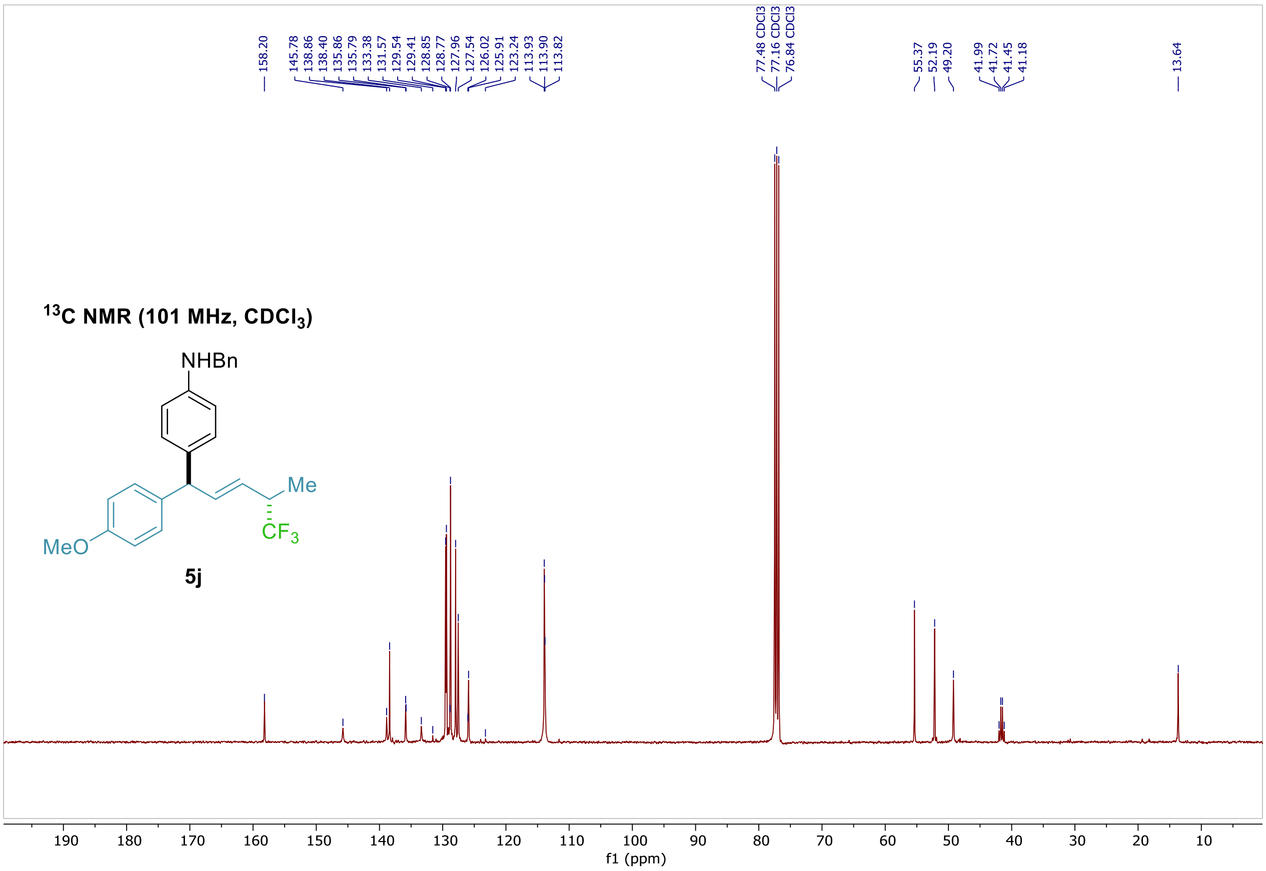
158.20
145.78
138.86
138.40
135.86
135.79
133.38
131.57
129.54
129.41
128.85
128.77
127.96
127.54
126.02
125.91
123.24
113.93
113.90
113.82
77.48 CDCl3
77.16 CDCl3
76.84 CDCl3
55.37
52.19
49.20
41.99
41.72
41.45
41.18
13.64
13C NMR (101 MHz, CDCl3)
NHBn
Me
MeO
CF3
5j
f1 (ppm)

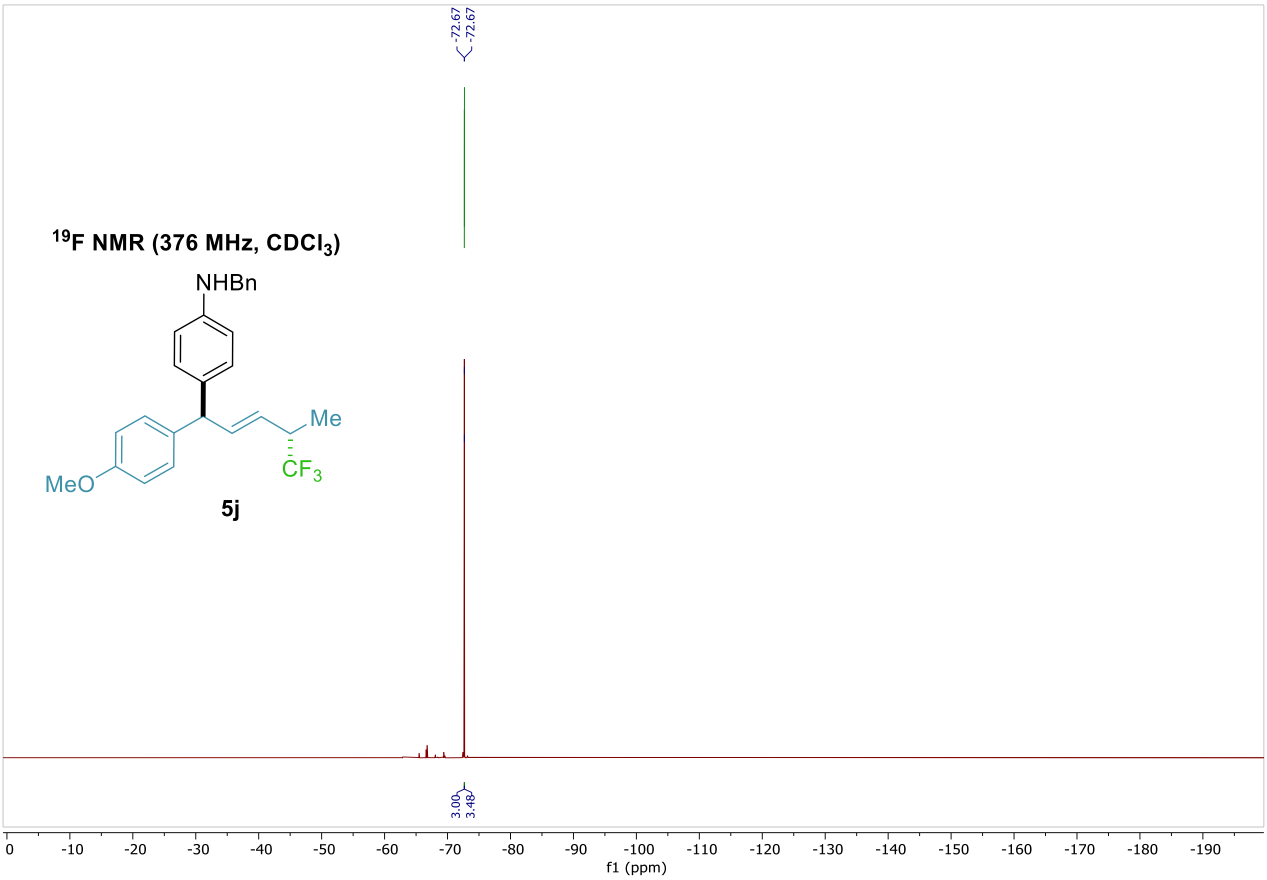
-72.67
-72.67
19F NMR (376 MHz, CDCl3)
NHBn
Me
MeO
CF3
5j
3.00
3.48
f1 (ppm)

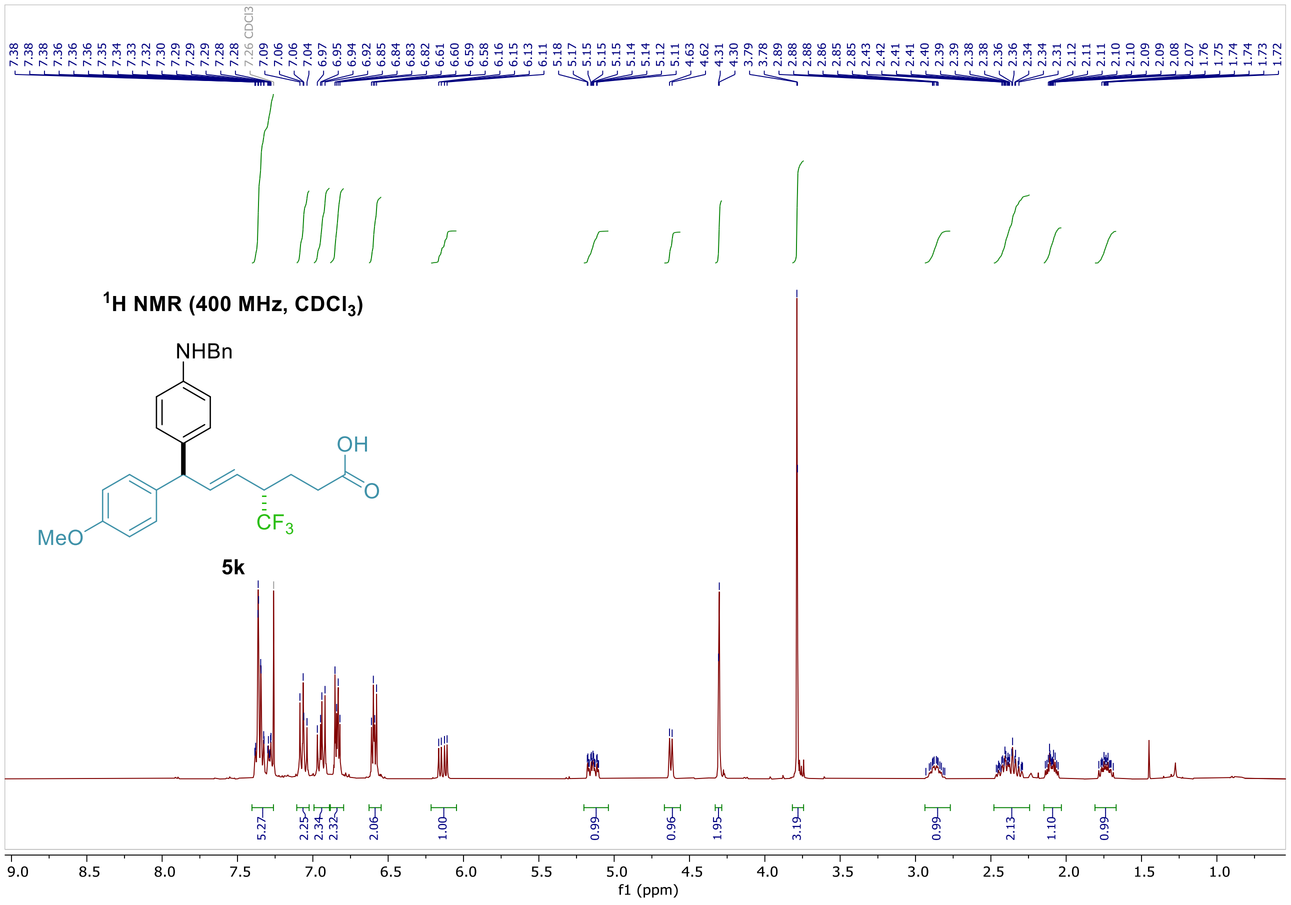

1H NMR (400 MHz, CDCl3)
NHBn
OH
O
MeO
CF3
5k
f1 (ppm)

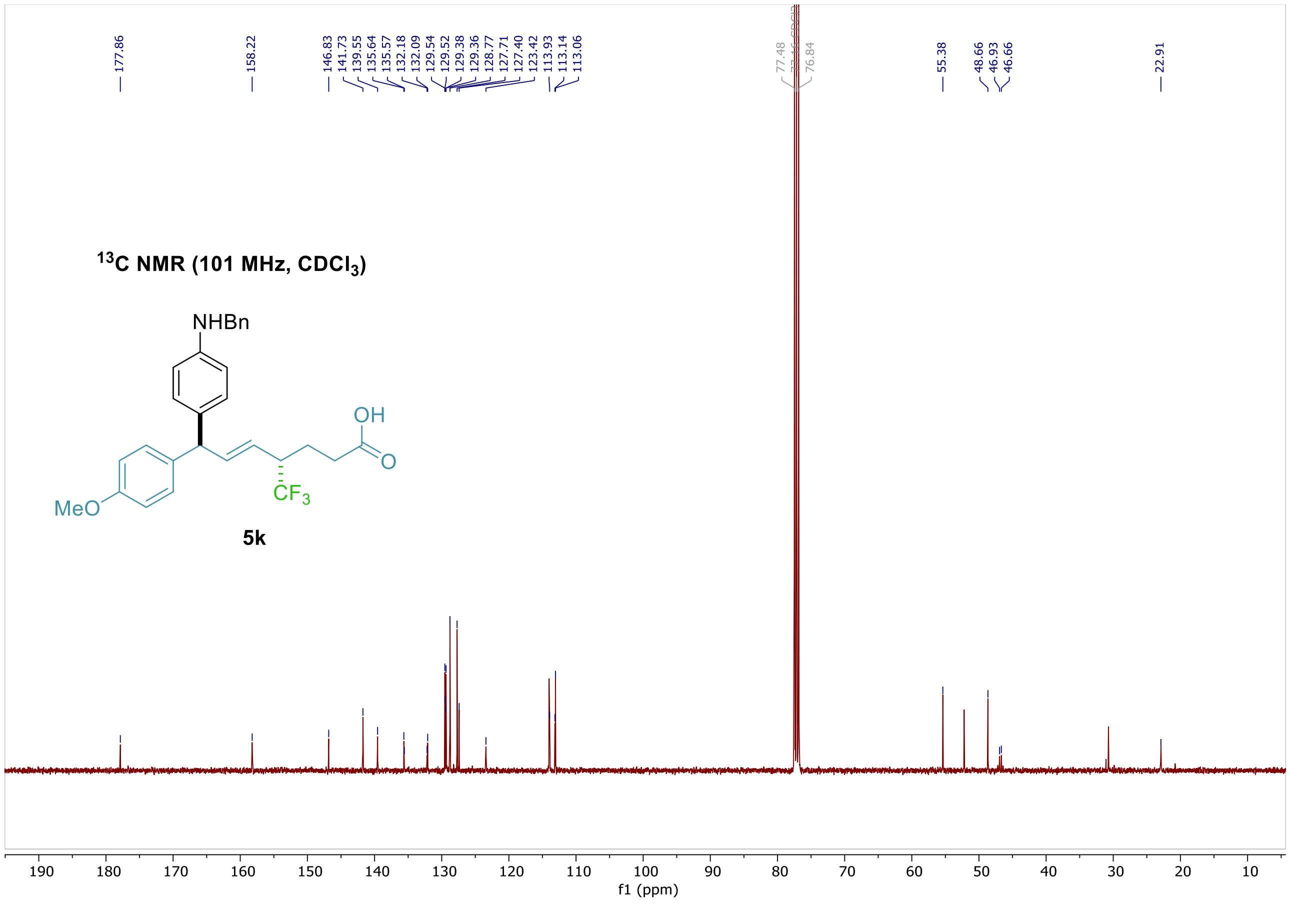

177.86
158.22
55.38
22.91
13C NMR (101 MHz, CDCl3)
NHBn
OH
O
MeO
CF3
5k
f1 (ppm)

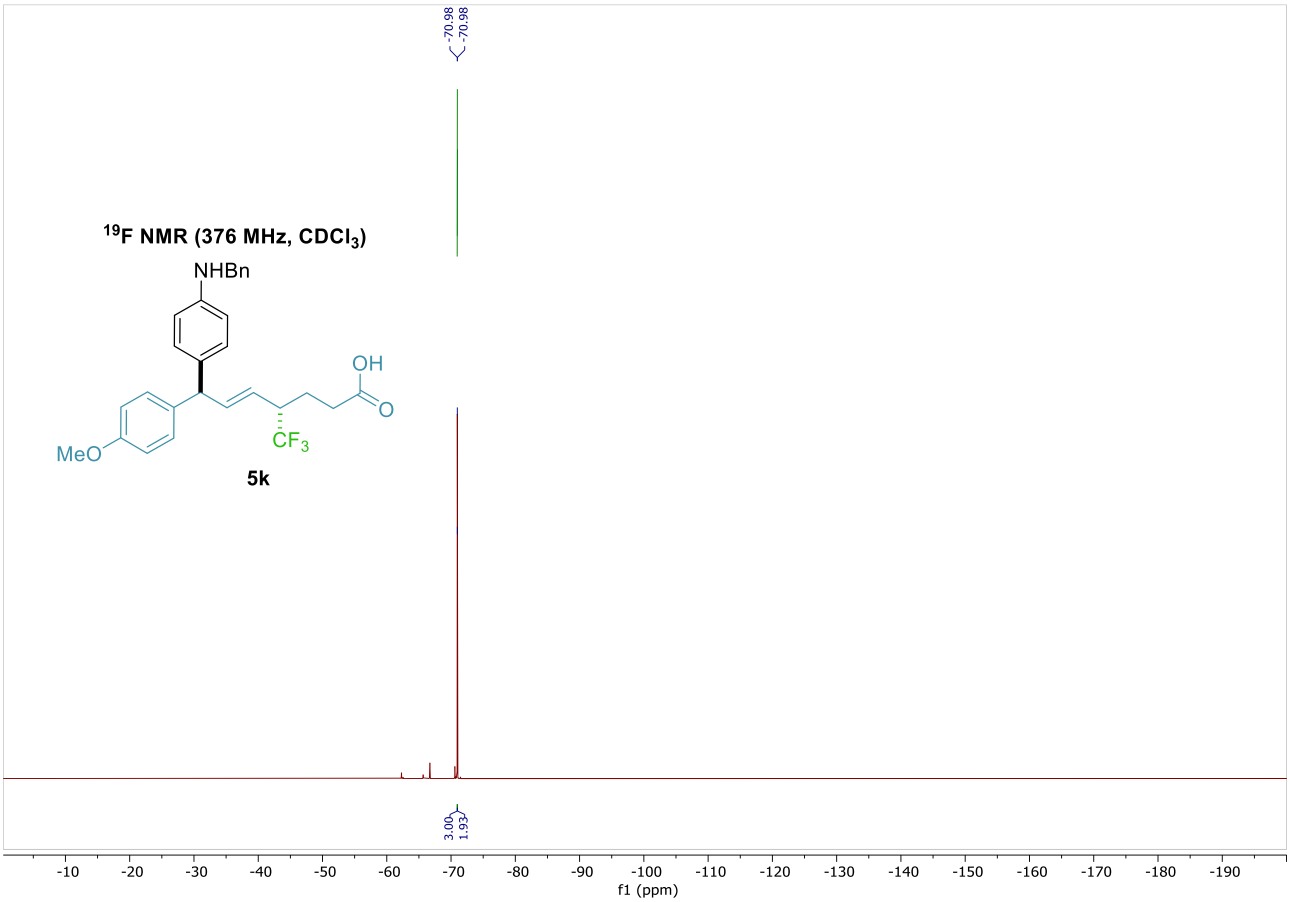
-70.98
-70.98
19F NMR (376 MHz, CDCl3)
NHBn
OH
O
MeO
CF3
5k
3.00
1.93
-10 -20 -30 -40 -50 -60 -70 -80 -90 -100 -110 -120 -130 -140 -150 -160 -170 -180 -190
f1 (ppm)

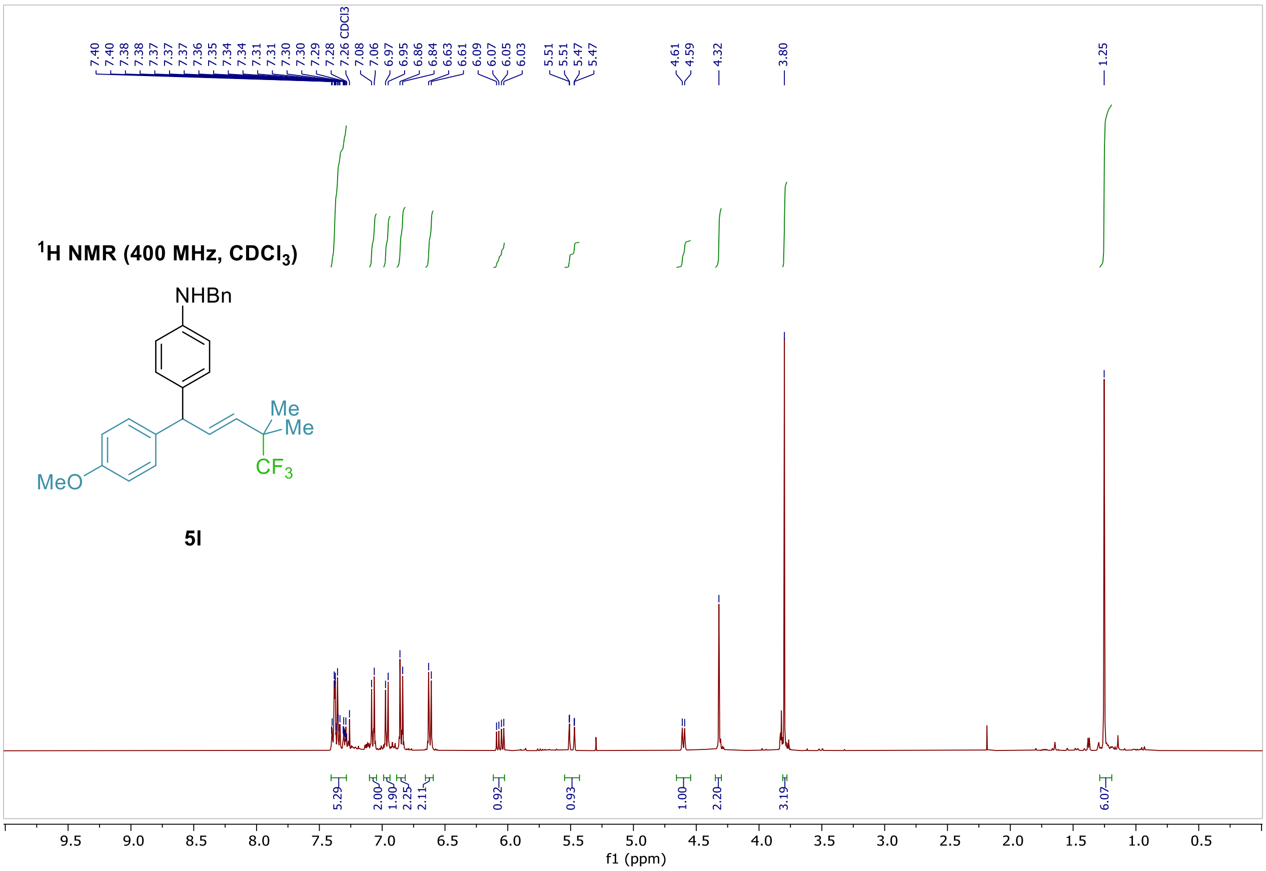
7.40 7.40 7.38 7.38 7.37 7.37 7.37 7.36 7.35 7.34 7.34 7.31 7.31 7.30 7.30 7.29 7.28 7.26 CDCl3 7.08 7.06 6.97 6.95 6.86 6.84 6.63 6.61 6.09 6.07 6.05 6.03 5.51 5.51 5.47 5.47 4.61 4.59 4.32 3.80 1.25
1H NMR (400 MHz, CDCl3)
NHBn
Me
Me
MeO
CF3
5l
5.29 2.00 1.90 2.25 2.11 0.92 0.93 1.00 2.20 3.19 6.07
9.5 9.0 8.5 8.0 7.5 7.0 6.5 6.0 5.5 5.0 4.5 4.0 3.5 3.0 2.5 2.0 1.5 1.0 0.5
f1 (ppm)

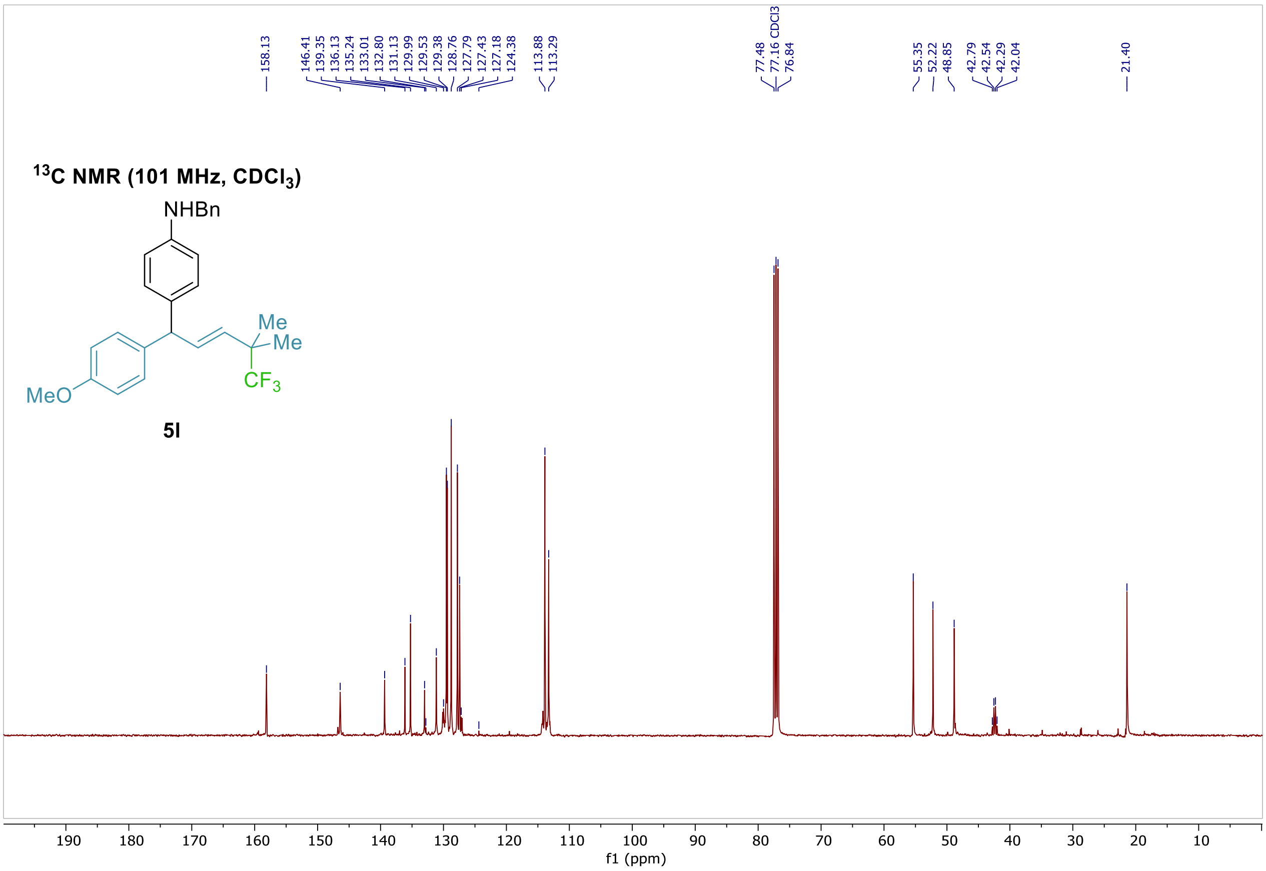
158.13
146.41
139.35
136.13
135.24
133.01
132.80
131.13
129.99
129.53
129.38
128.76
127.79
127.43
127.18
124.38
113.88
113.29
77.48
77.16 CDCl3
76.84
55.35
52.22
48.85
42.79
42.54
42.29
42.04
21.40
13C NMR (101 MHz, CDCl3)
NHBn
Me
Me
CF3
MeO
5l
f1 (ppm)

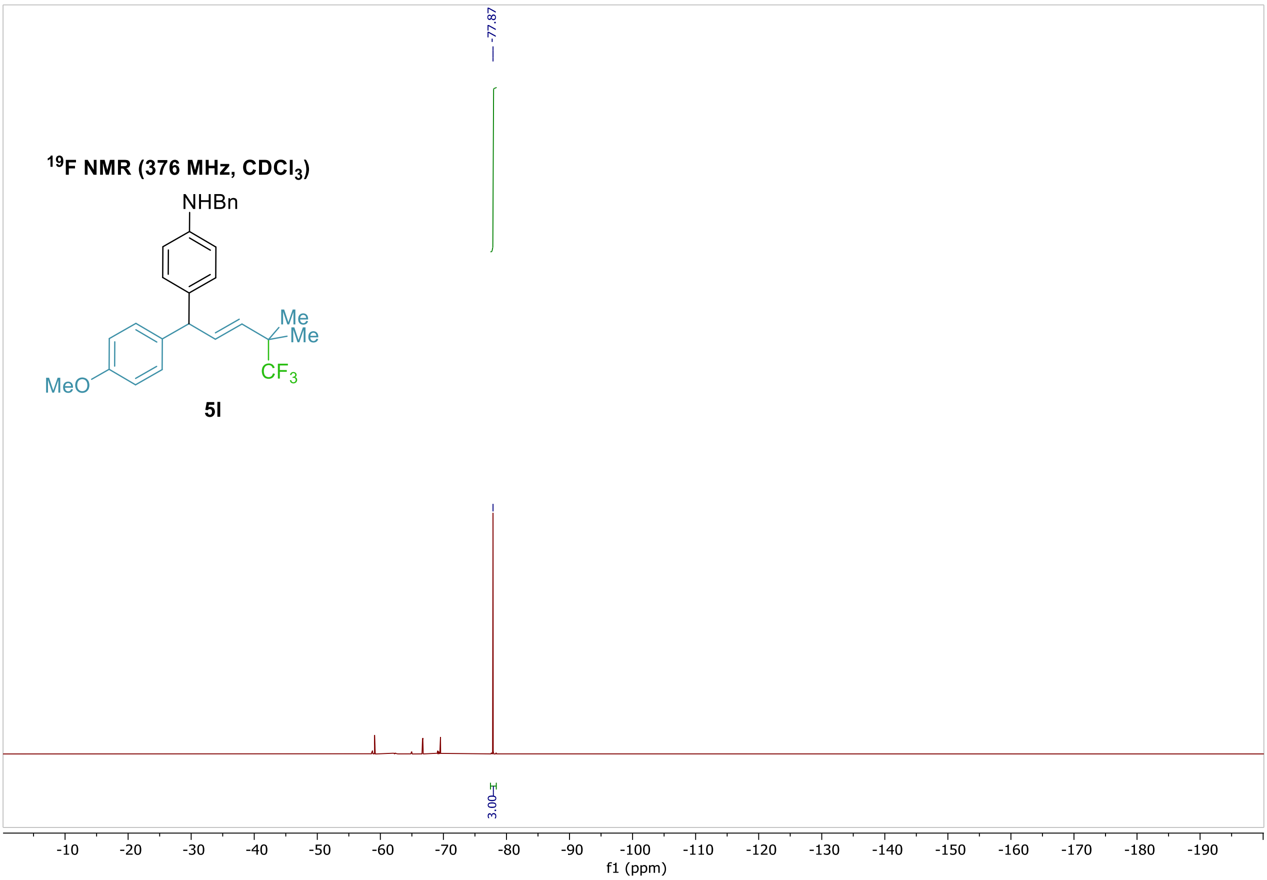
-77.87
19F NMR (376 MHz, CDCl3)
NHBn
Me
Me
CF3
MeO
5l
3.00
f1 (ppm)

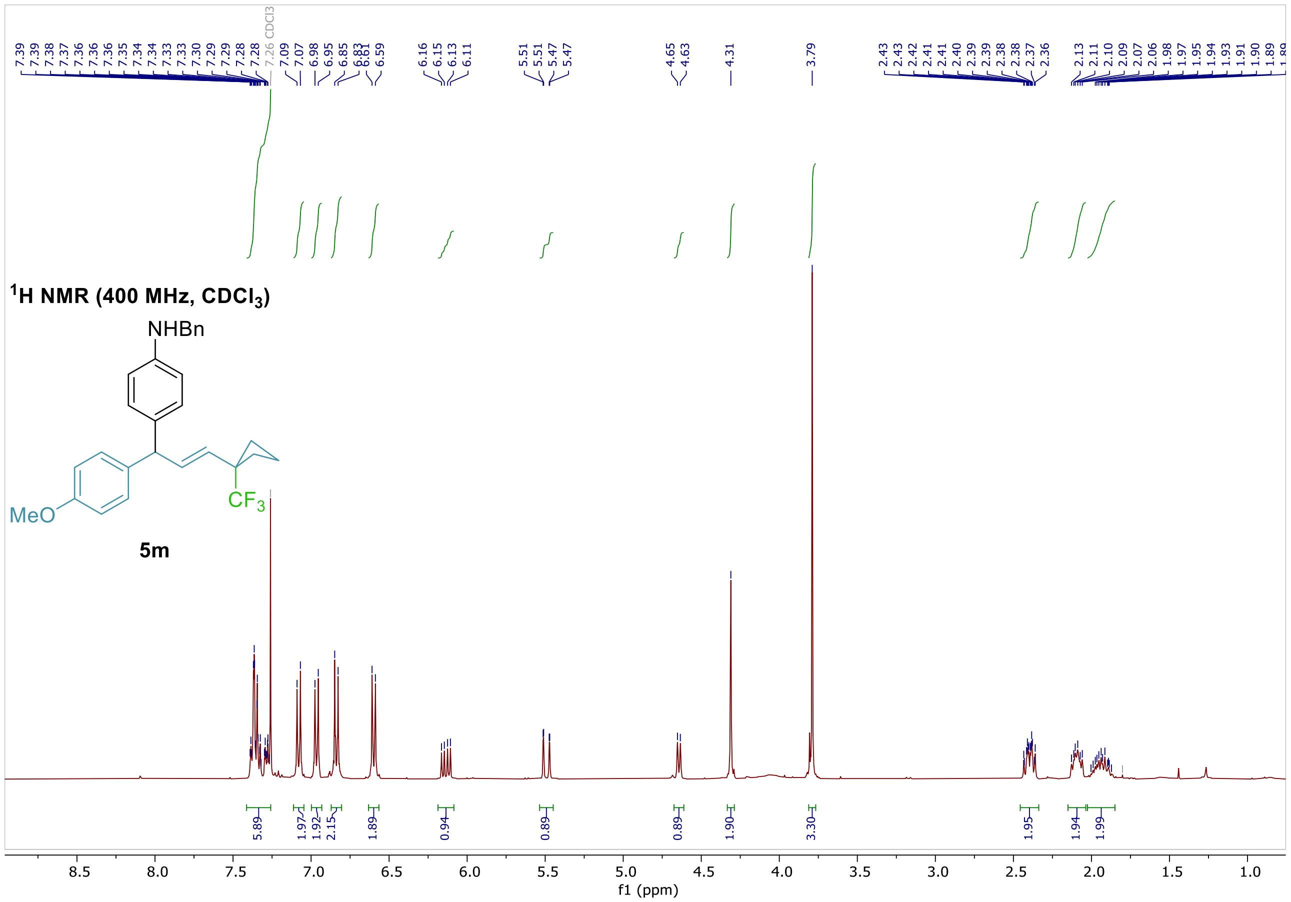
1H NMR (400 MHz, CDCl3)
NHBn
MeO
CF3
5m
7.26 CDCl3
5.89
1.97
1.92
2.15
1.89
0.94
0.89
0.89
1.90
3.30
1.95
1.94
1.99
f1 (ppm)

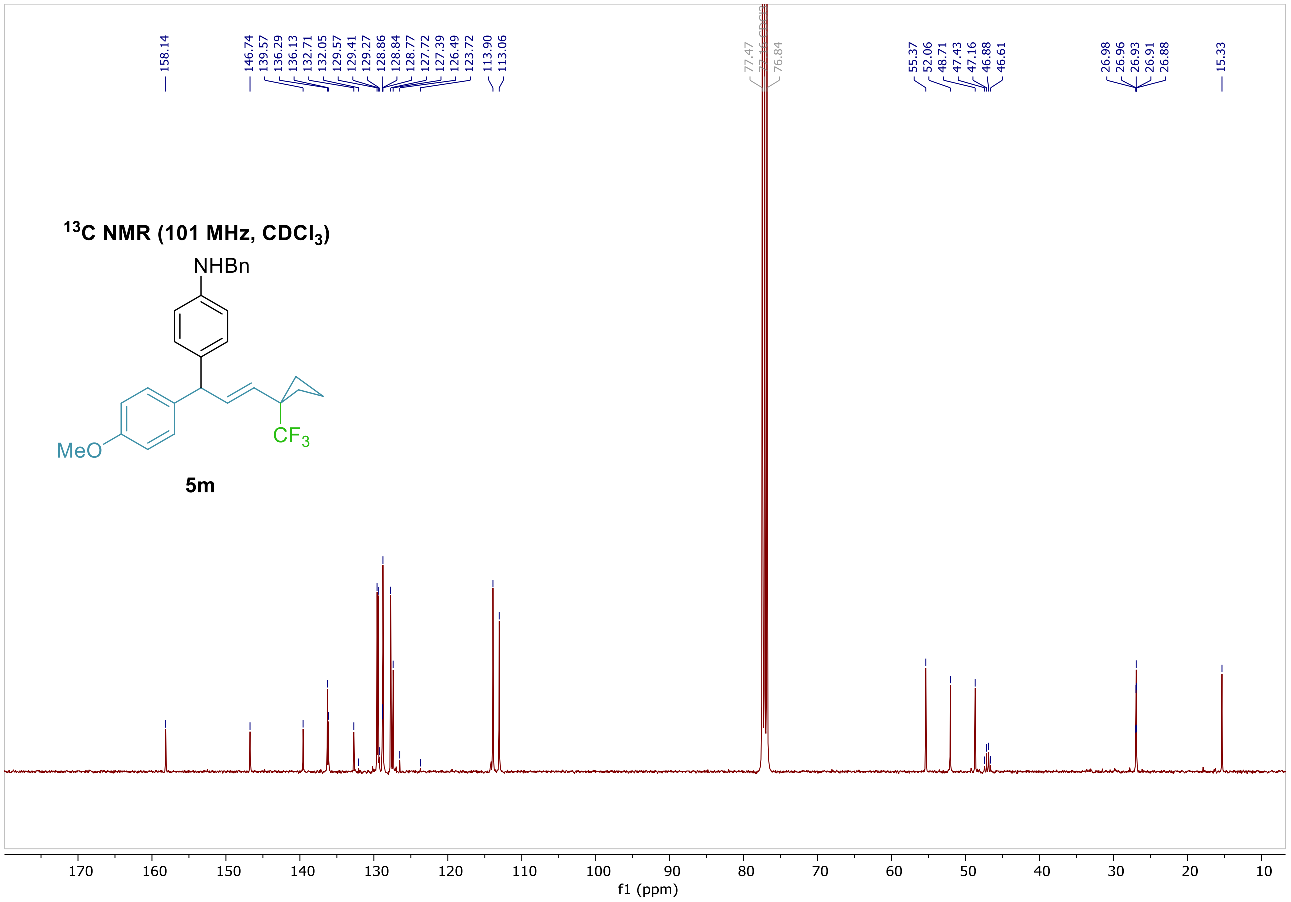
13C NMR (101 MHz, CDCl3)
NHBn
MeO
CF3
5m
158.14
146.74
139.57
136.29
136.13
132.71
132.05
129.57
129.41
129.27
128.86
128.84
128.77
127.72
127.39
126.49
123.72
113.90
113.06
77.47
76.84
55.37
52.06
48.71
47.43
47.16
46.88
46.61
26.98
26.96
26.93
26.91
26.88
15.33
f1 (ppm)

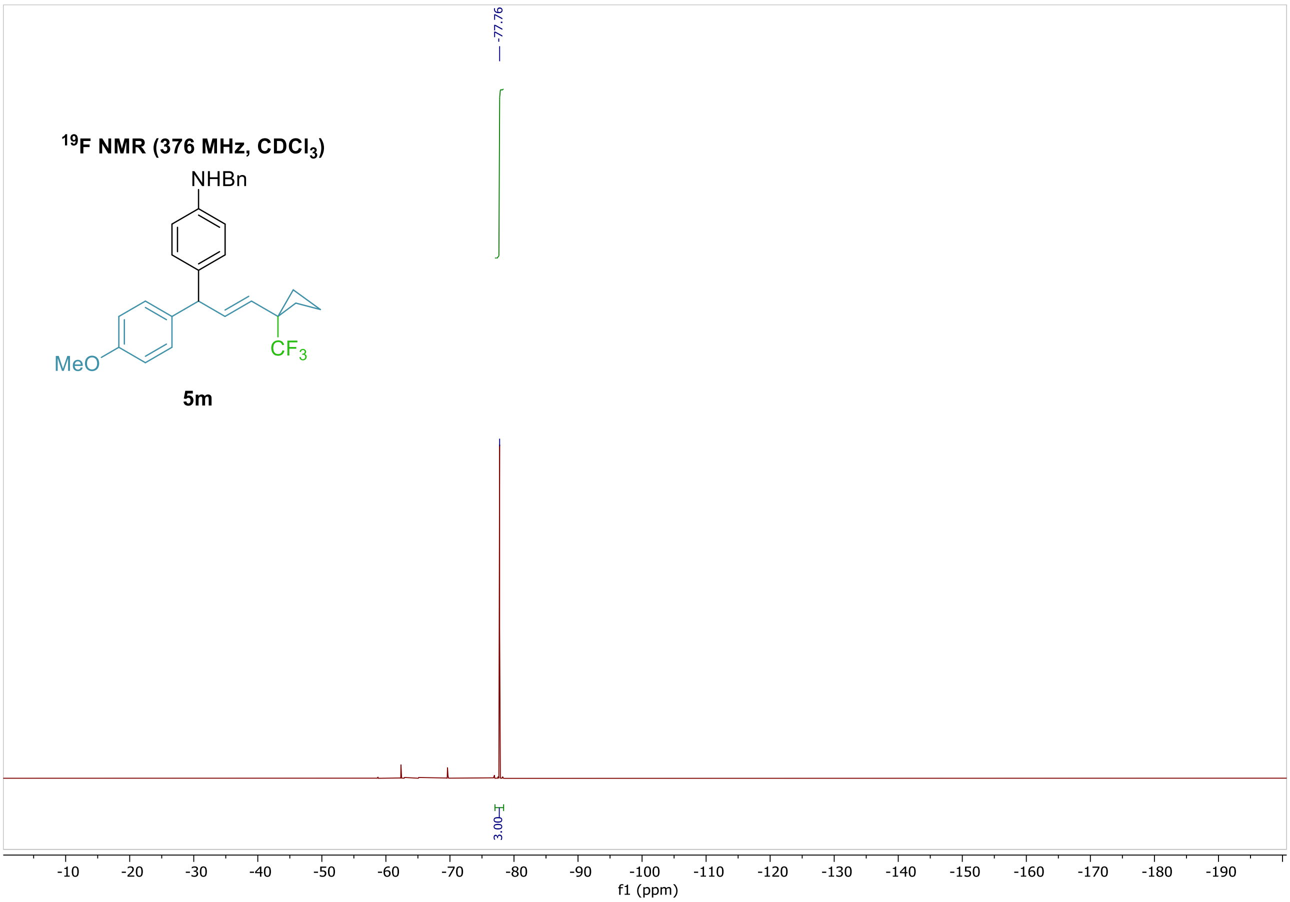

19F NMR (376 MHz, CDCl3)
NHBn
MeO
CF3
5m
-77.76
3.00
f1 (ppm)

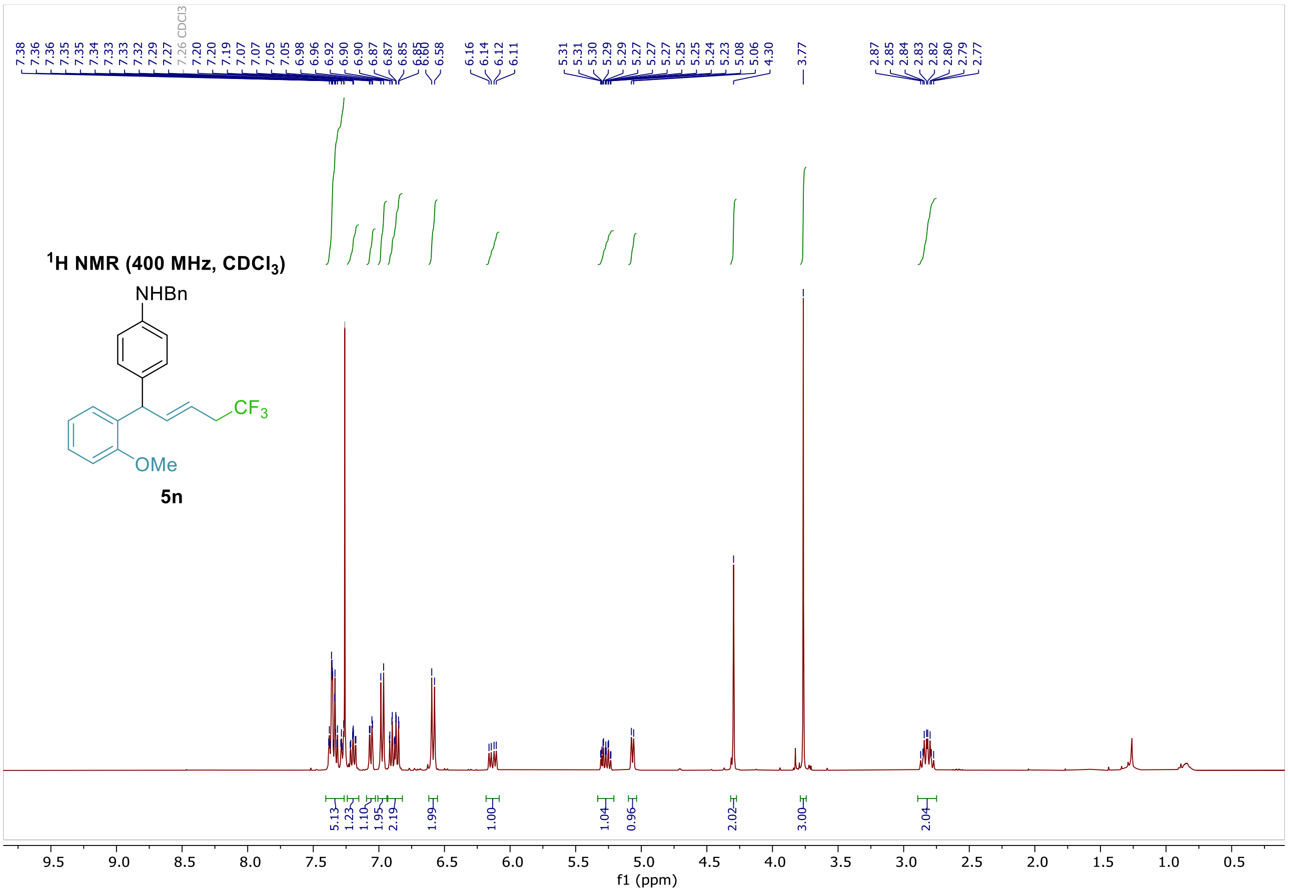

1H NMR (400 MHz, CDCl3)
NHBn
CF3
OMe
5n
7.38 7.36 7.35 7.35 7.34 7.33 7.33 7.32 7.29 7.27 7.26 CDCl3 7.20 7.20 7.19 7.07 7.07 7.05 7.05 6.98 6.96 6.92 6.90 6.90 6.87 6.87 6.85 6.85 6.60 6.58 6.16 6.14 6.12 6.11 5.31 5.31 5.30 5.29 5.29 5.27 5.27 5.27 5.25 5.25 5.24 5.23 5.08 5.06 4.30 3.77 2.87 2.85 2.84 2.83 2.82 2.80 2.79 2.77
5.13 1.23 1.10 1.95 2.19 1.99 1.00 1.04 0.96 2.02 3.00 2.04
f1 (ppm)

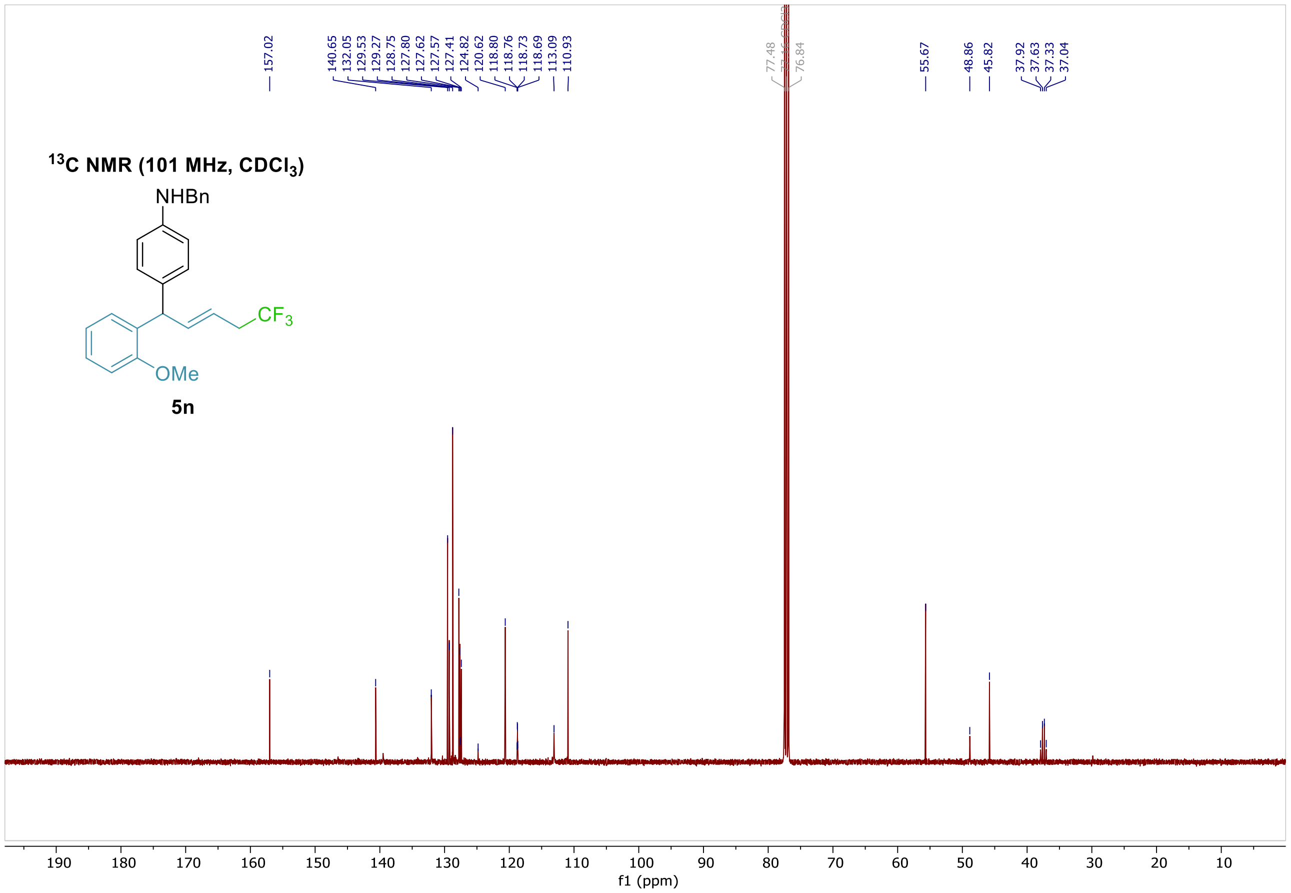

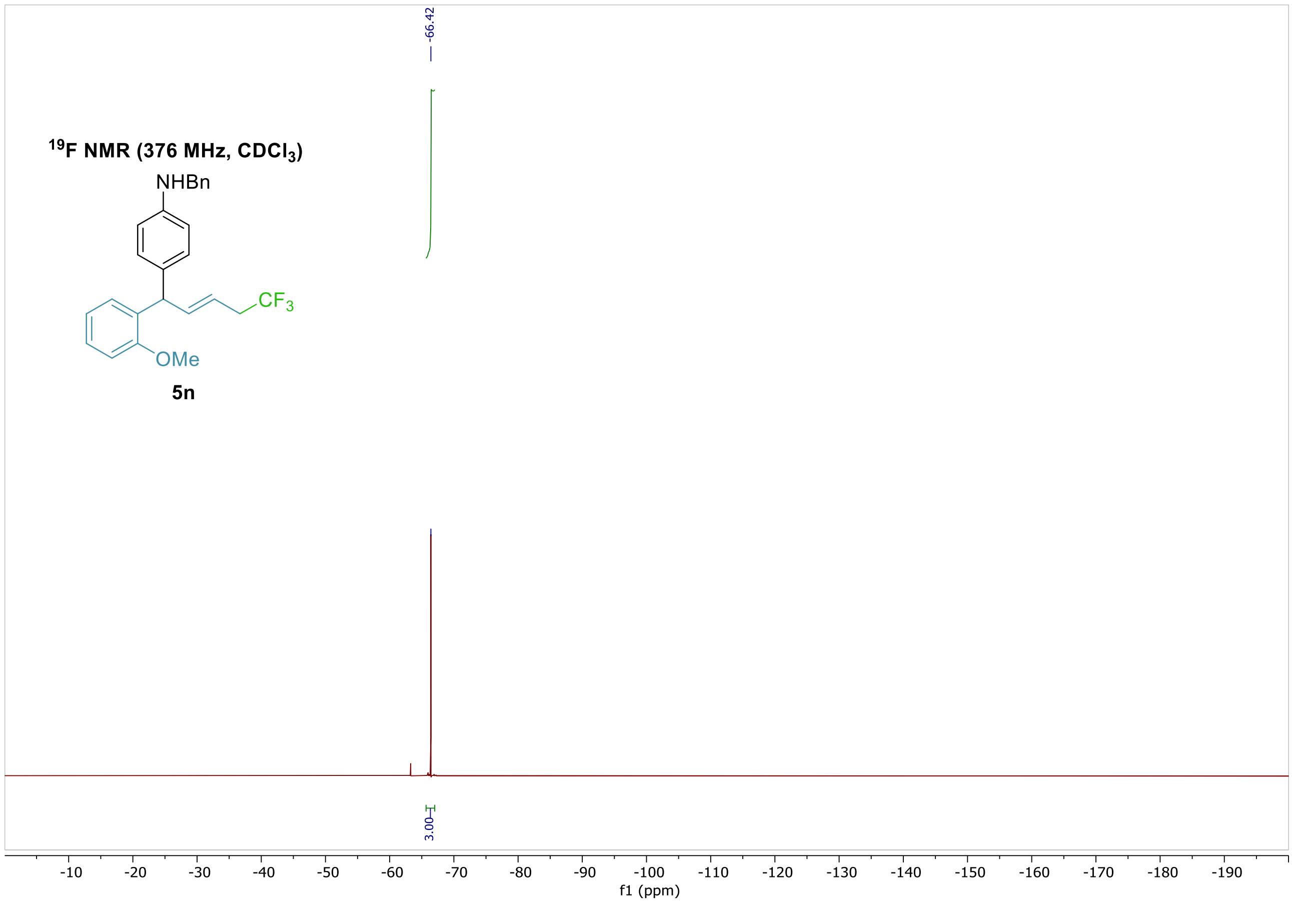

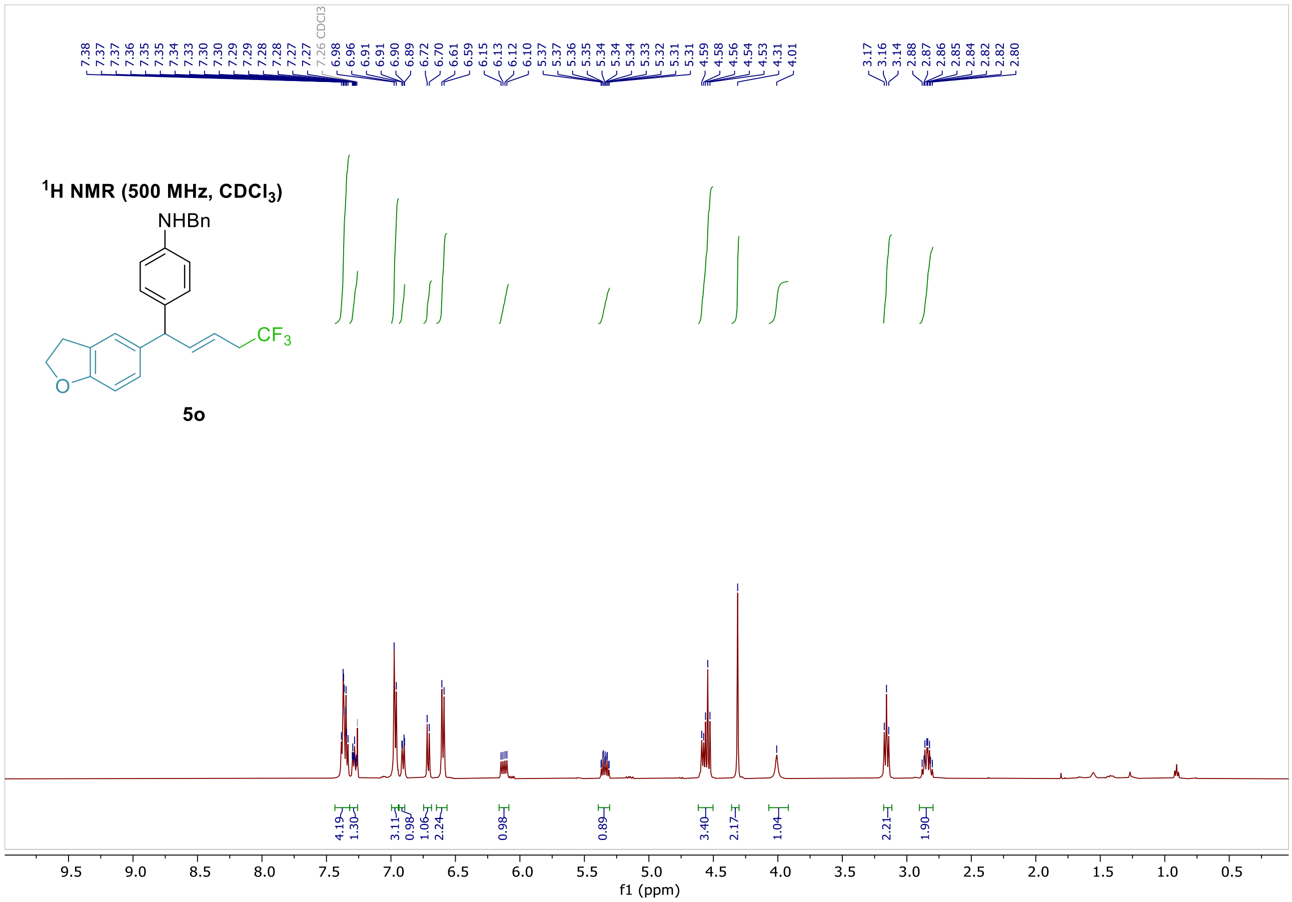
1H NMR (500 MHz, CDCl3)
NHBn
CF3
5o
f1 (ppm)

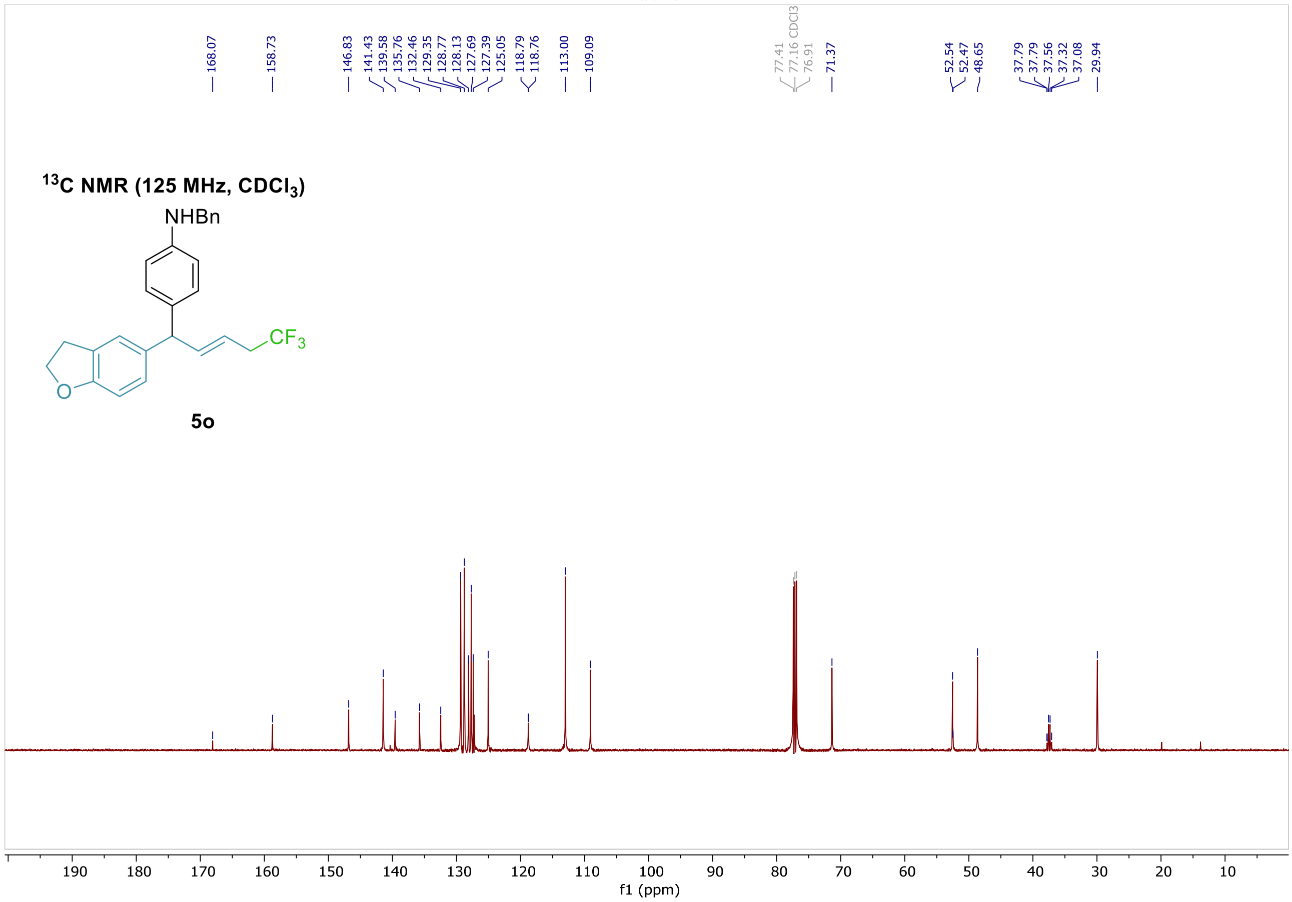
13C NMR (125 MHz, CDCl3)
NHBn
CF3
5o
f1 (ppm)

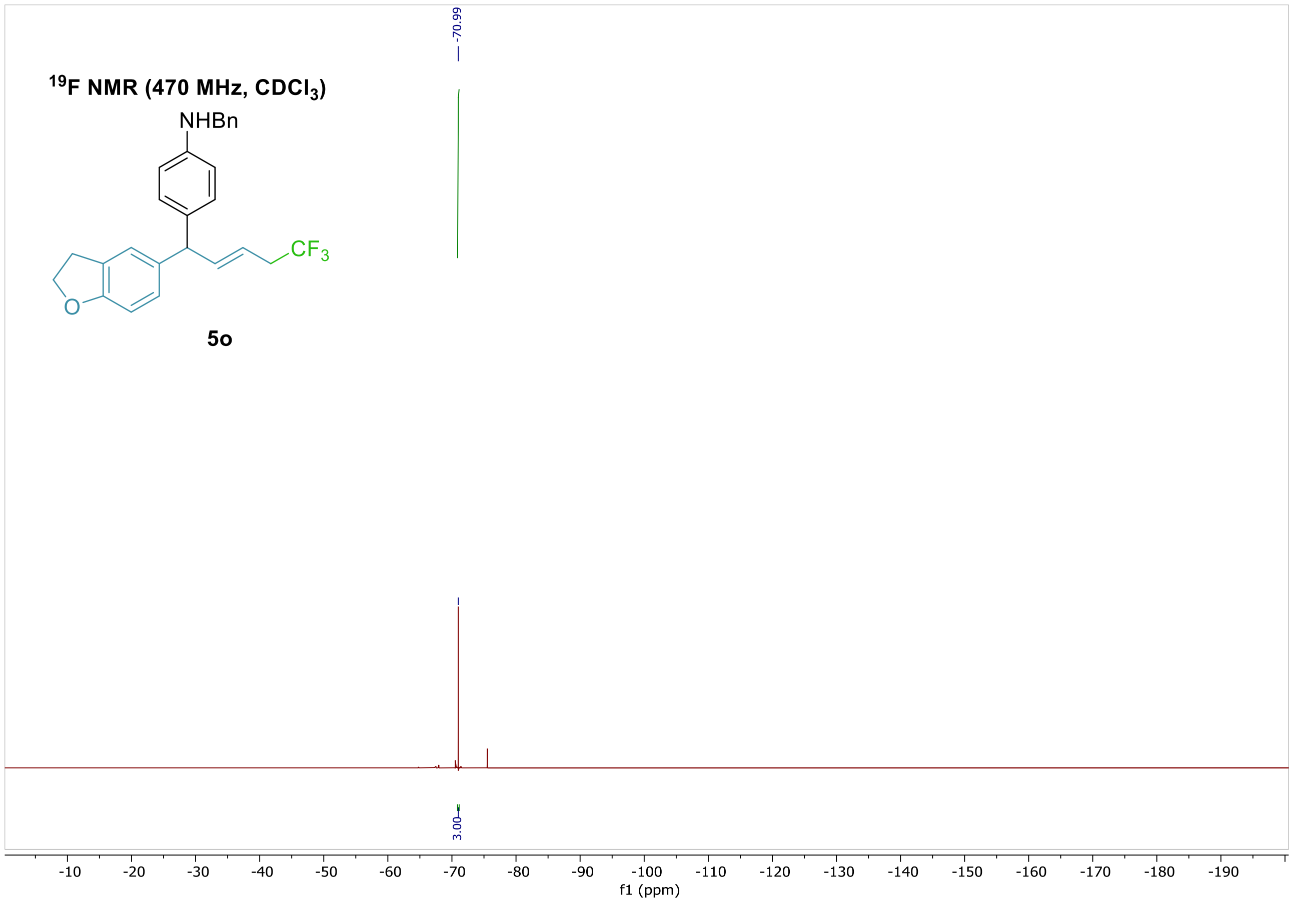

-70.99
19F NMR (470 MHz, CDCl3)
NHBn
CF3
O
5o
3.00
f1 (ppm)

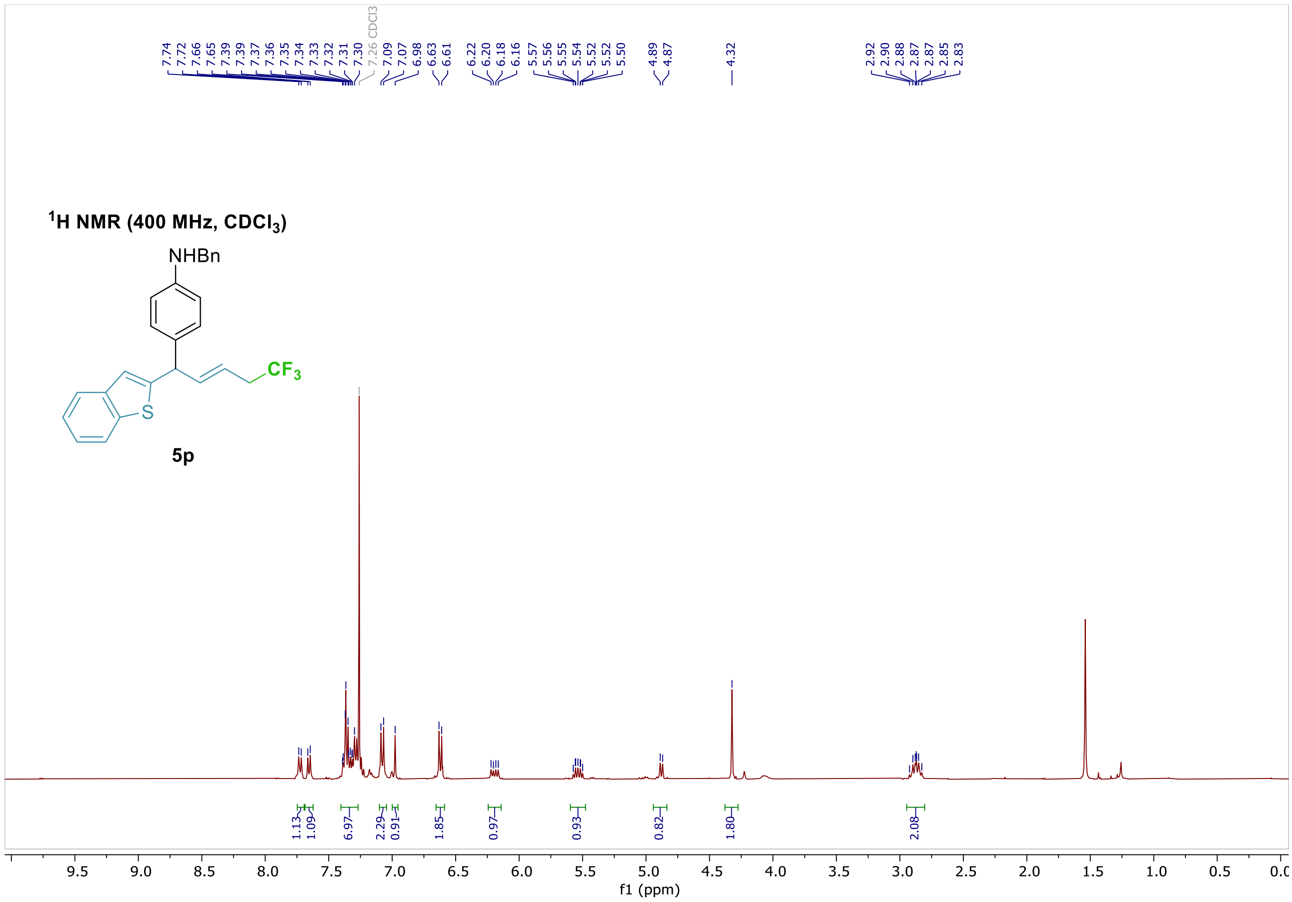

7.74
7.72
7.66
7.65
7.39
7.39
7.37
7.36
7.35
7.34
7.33
7.32
7.31
7.30
7.26 CDCl3
7.09
7.07
6.98
6.63
6.61
6.22
6.20
6.18
6.16
5.57
5.56
5.55
5.54
5.52
5.52
5.50
4.89
4.87
4.32
2.92
2.90
2.88
2.87
2.87
2.85
2.83
1H NMR (400 MHz, CDCl3)
NHBn
CF3
S
5p
1.13
1.09
6.97
2.29
0.91
1.85
0.97
0.93
0.82
1.80
2.08
f1 (ppm)

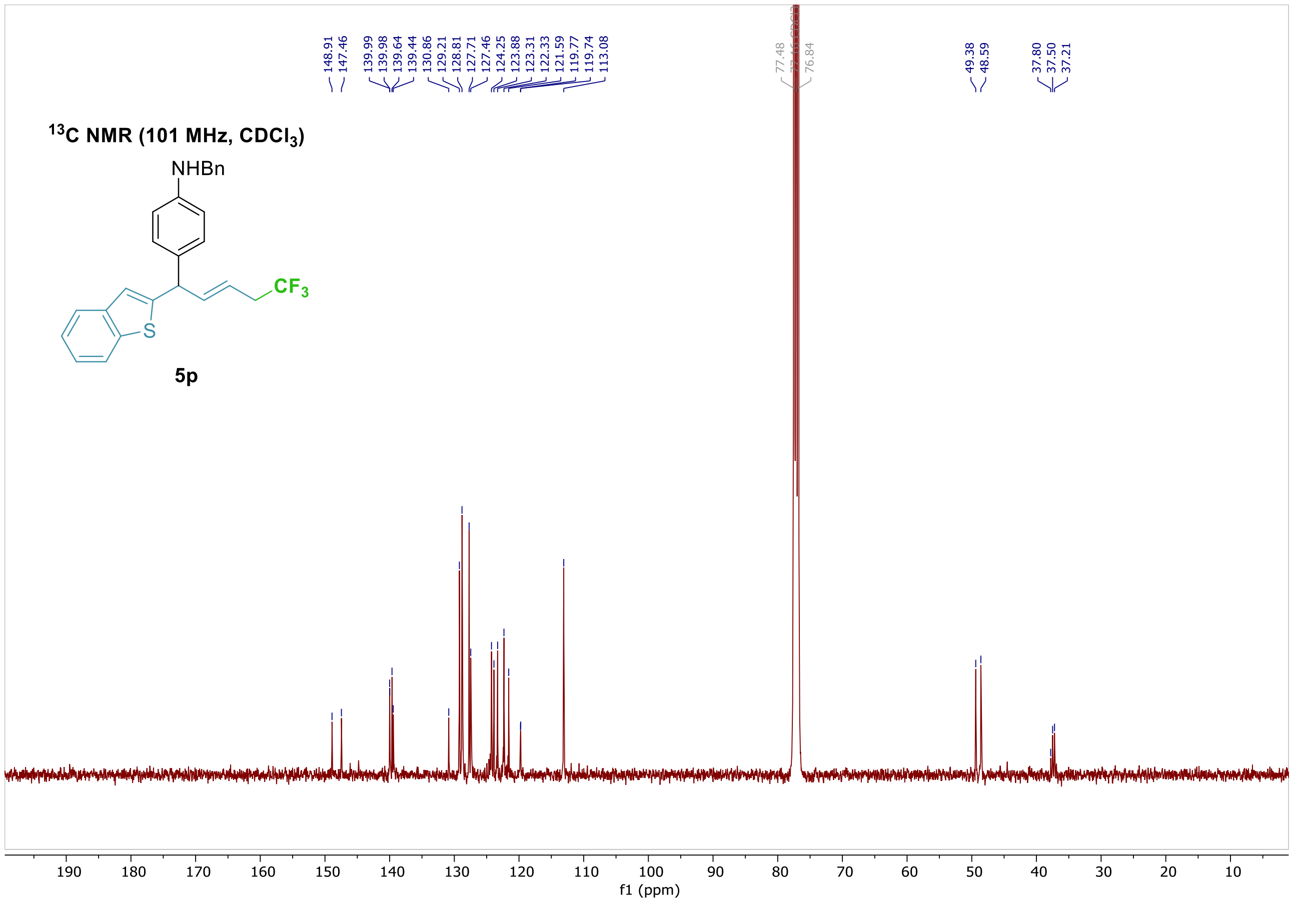

13C NMR (101 MHz, CDCl3)
NHBn
CF3
S
5p
148.91
147.46
139.99
139.98
139.64
139.44
130.86
129.21
128.81
127.71
127.46
124.25
123.88
123.31
122.33
121.59
119.77
119.74
113.08
77.48
76.84
49.38
48.59
37.80
37.50
37.21
f1 (ppm)

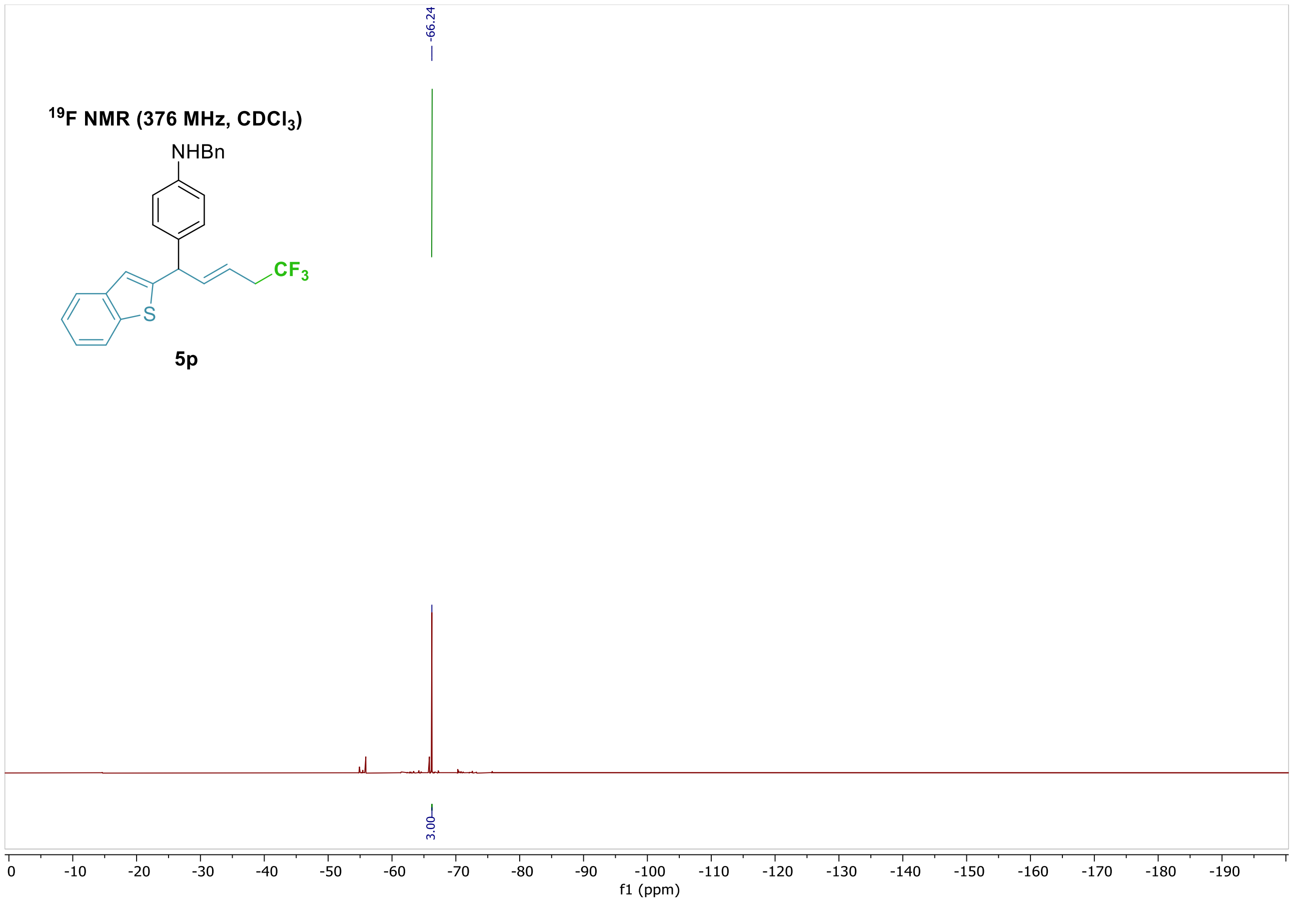

19F NMR (376 MHz, CDCl3)
NHBn
CF3
S
5p
-66.24
3.00
f1 (ppm)

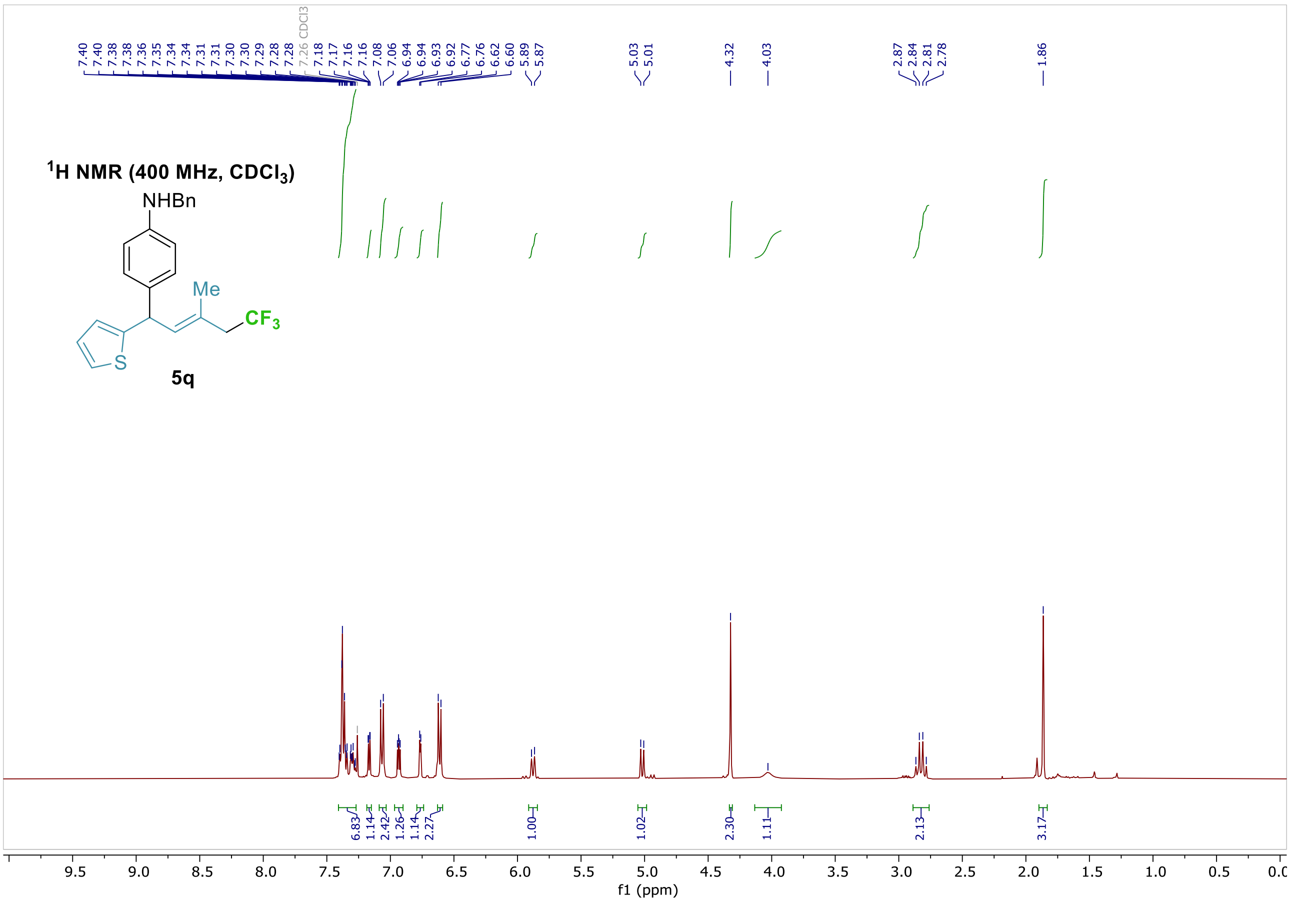

7.40 7.40 7.38 7.38 7.36 7.35 7.34 7.34 7.31 7.31 7.30 7.30 7.29 7.28 7.28 7.26 CDCl3 7.18 7.17 7.16 7.16 7.08 7.06 6.94 6.94 6.93 6.92 6.77 6.76 6.62 6.60 5.89 5.87 5.03 5.01 4.32 4.03 2.87 2.84 2.81 2.78 1.86
^{1}H NMR (400 MHz, $CDCl_3$)
NHBn
Me
CF_3
S
5q
6.83 1.14 2.42 1.26 1.14 2.27 1.00 1.02 2.30 1.11 2.13 3.17
9.5 9.0 8.5 8.0 7.5 7.0 6.5 6.0 5.5 5.0 4.5 4.0 3.5 3.0 2.5 2.0 1.5 1.0 0.5
f1 (ppm)

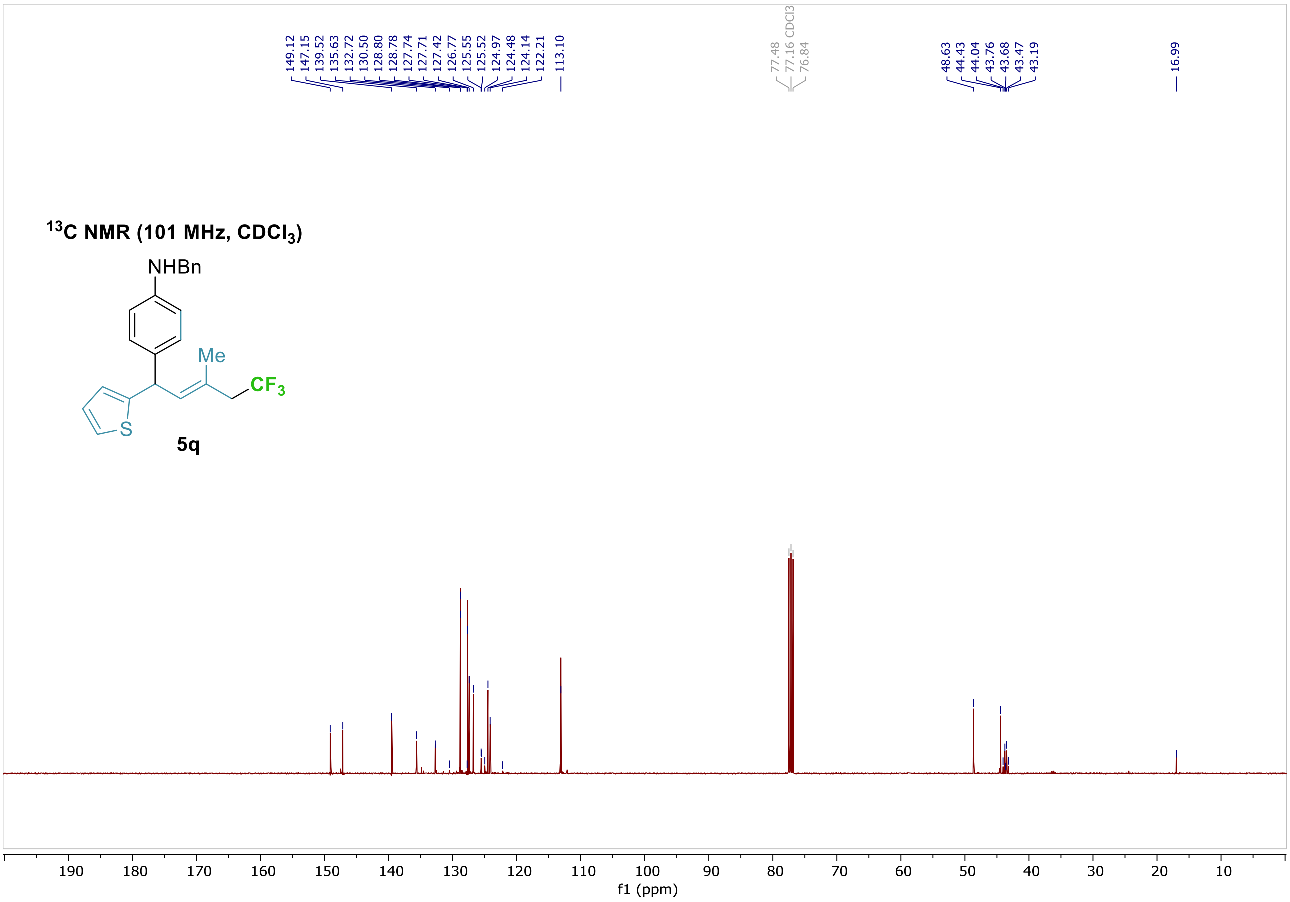

149.12 147.15 139.52 135.63 132.72 130.50 128.80 128.78 127.74 127.71 127.42 126.77 125.55 125.52 124.97 124.48 124.14 122.21 113.10 77.48 77.16 CDCl3 76.84 48.63 44.43 44.04 43.76 43.68 43.47 43.19 16.99
^{13}C NMR (101 MHz, $CDCl_3$)
NHBn
Me
CF_3
S
5q
190 180 170 160 150 140 130 120 110 100 90 80 70 60 50 40 30 20 10
f1 (ppm)

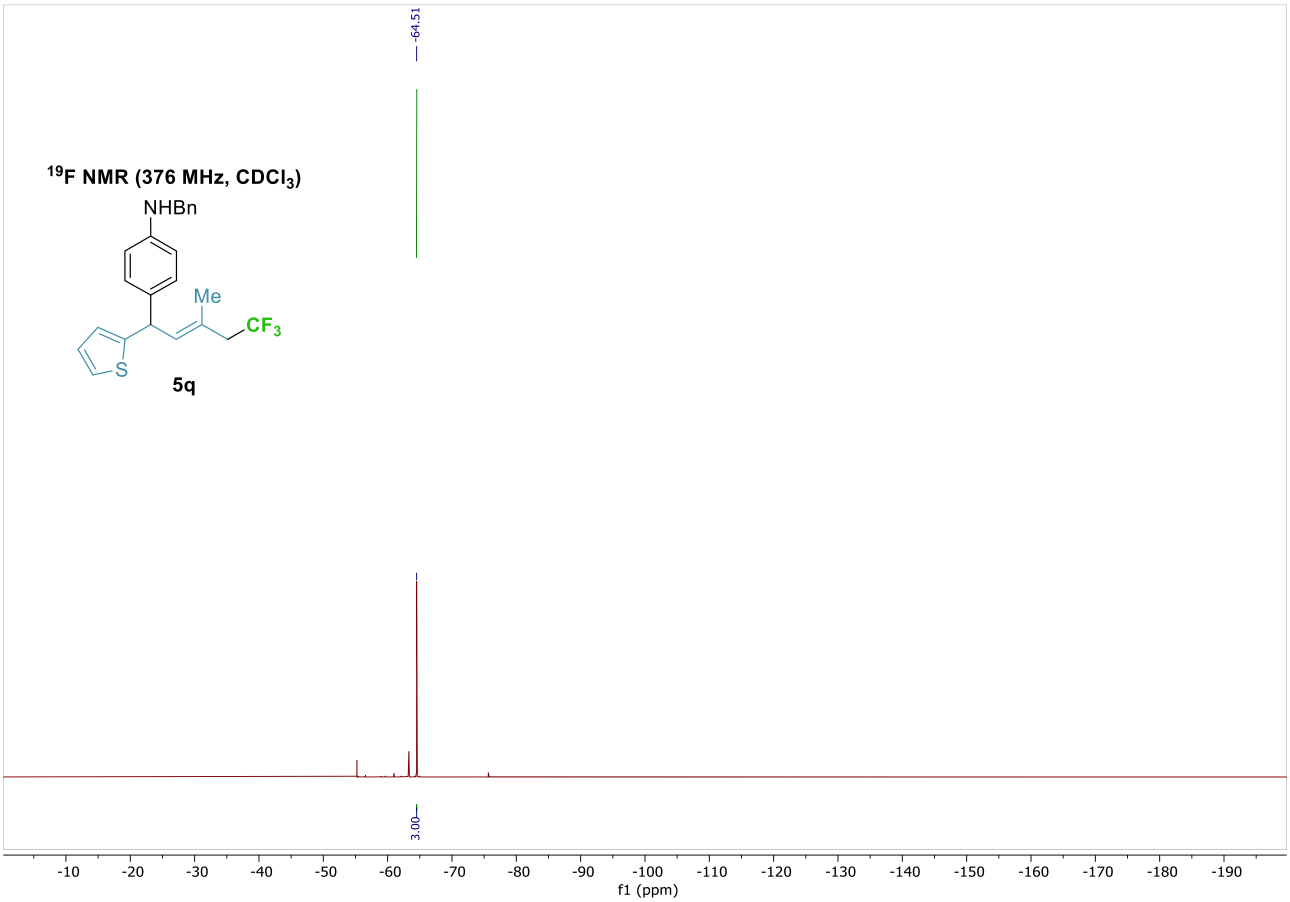
19F NMR (376 MHz, CDCl3)
NHBn
Me
CF3
S
5q
-64.51
3.00
f1 (ppm)

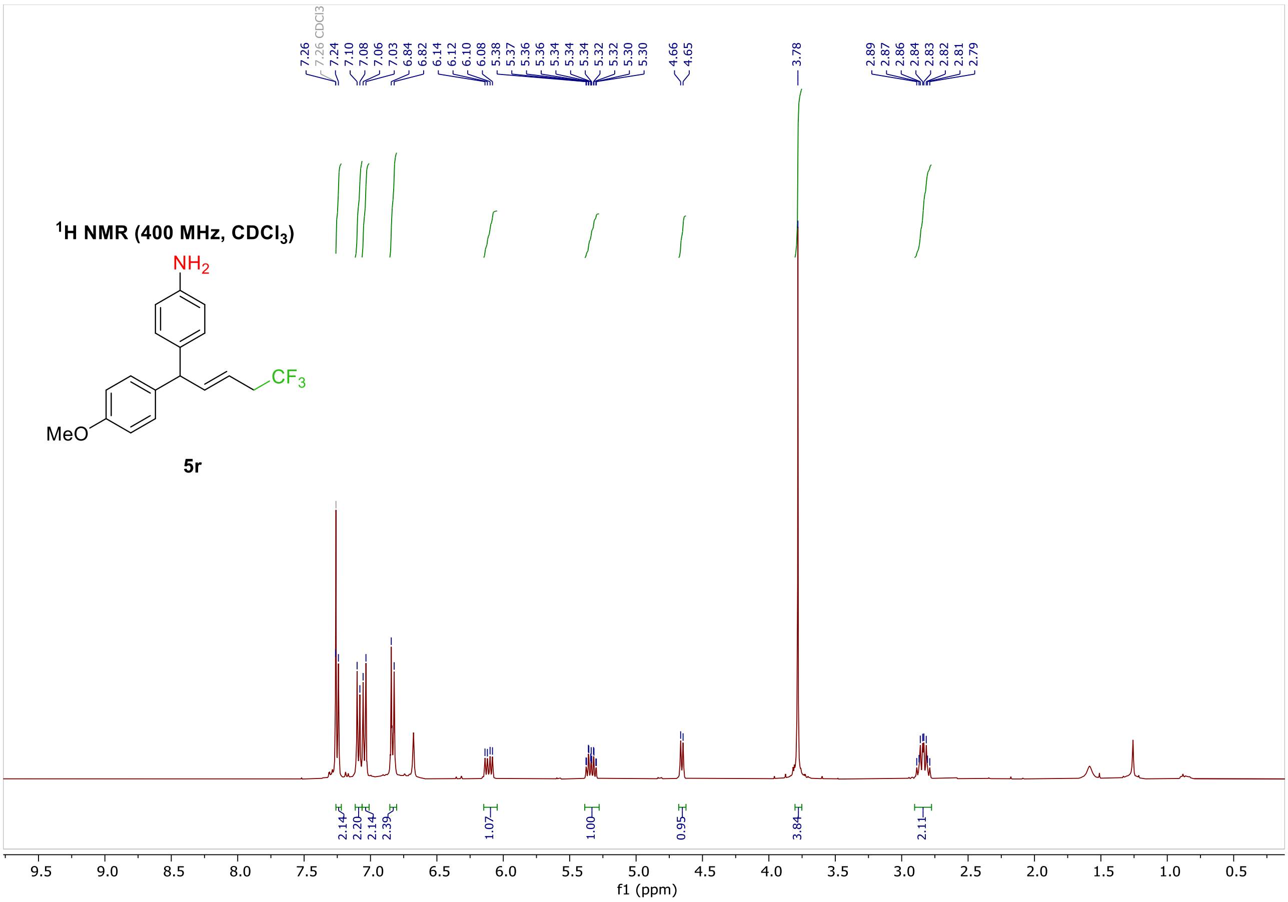
1H NMR (400 MHz, CDCl3)
NH2
CF3
MeO
5r
f1 (ppm)

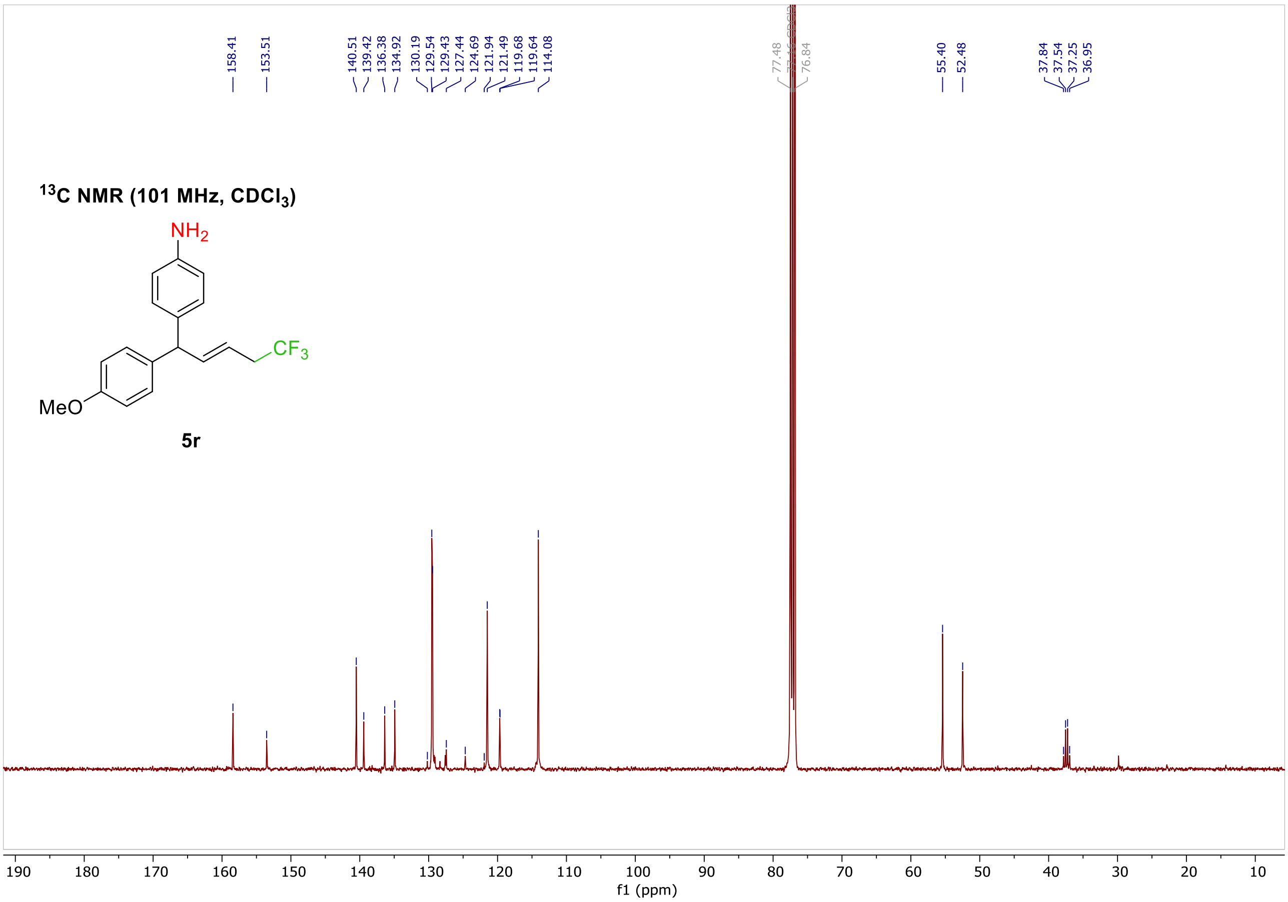

158.41
153.51
140.51
139.42
136.38
134.92
130.19
129.54
129.43
127.44
124.69
121.94
121.49
119.68
119.64
114.08
77.48
76.84
55.40
52.48
37.84
37.54
37.25
36.95
^{13}C NMR (101 MHz, $CDCl_3$)
NH_2
CF_3
MeO
5r
190
180
170
160
150
140
130
120
110
100
90
80
70
60
50
40
30
20
10
f1 (ppm)

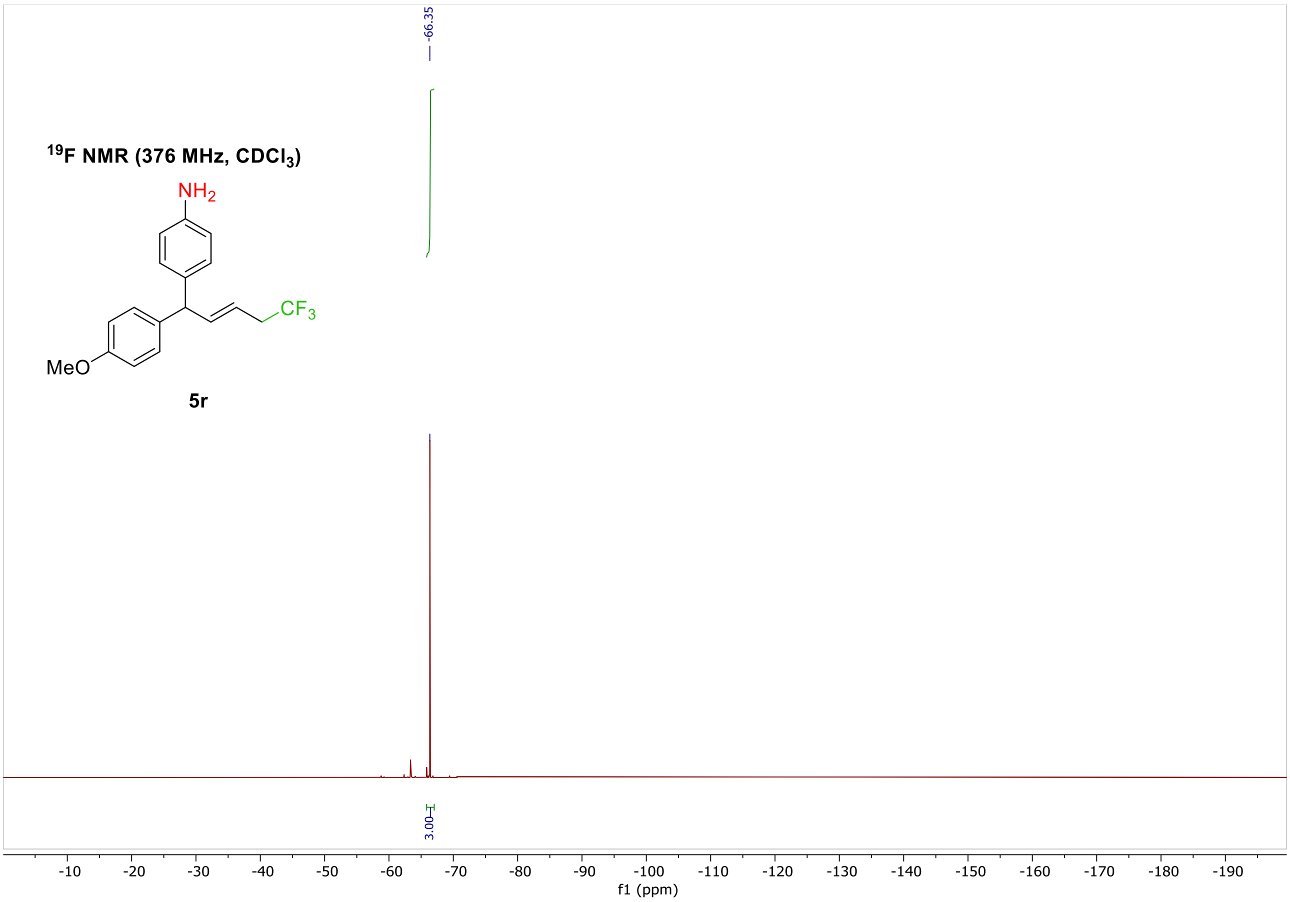

-66.35
^{19}F NMR (376 MHz, $CDCl_3$)
NH_2
CF_3
MeO
5r
3.00
-10
-20
-30
-40
-50
-60
-70
-80
-90
-100
-110
-120
-130
-140
-150
-160
-170
-180
-190
f1 (ppm)

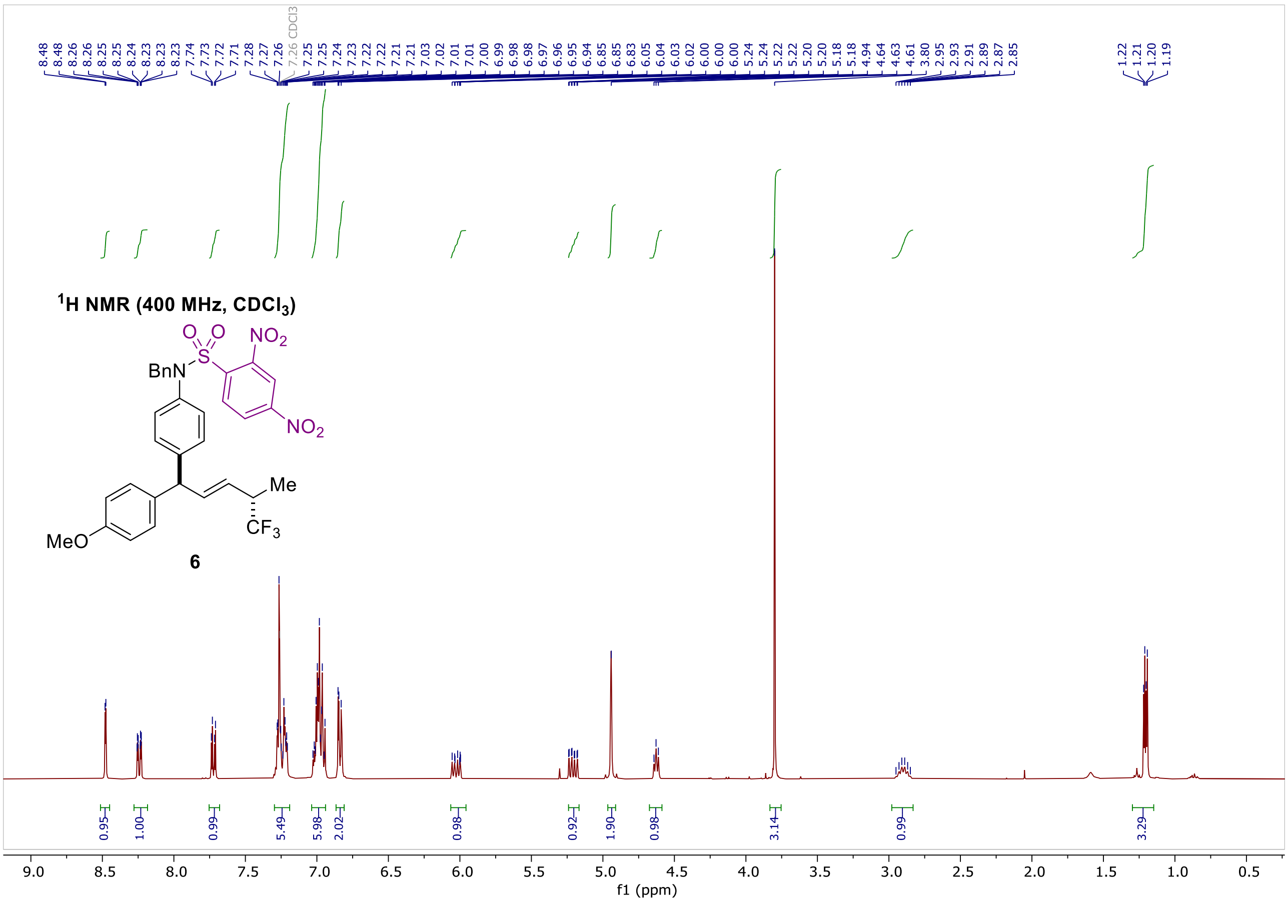

^{1}H NMR (400 MHz, $CDCl_3$)
BnN
NO_2
NO_2
Me
CF_3
MeO
6
f1 (ppm)

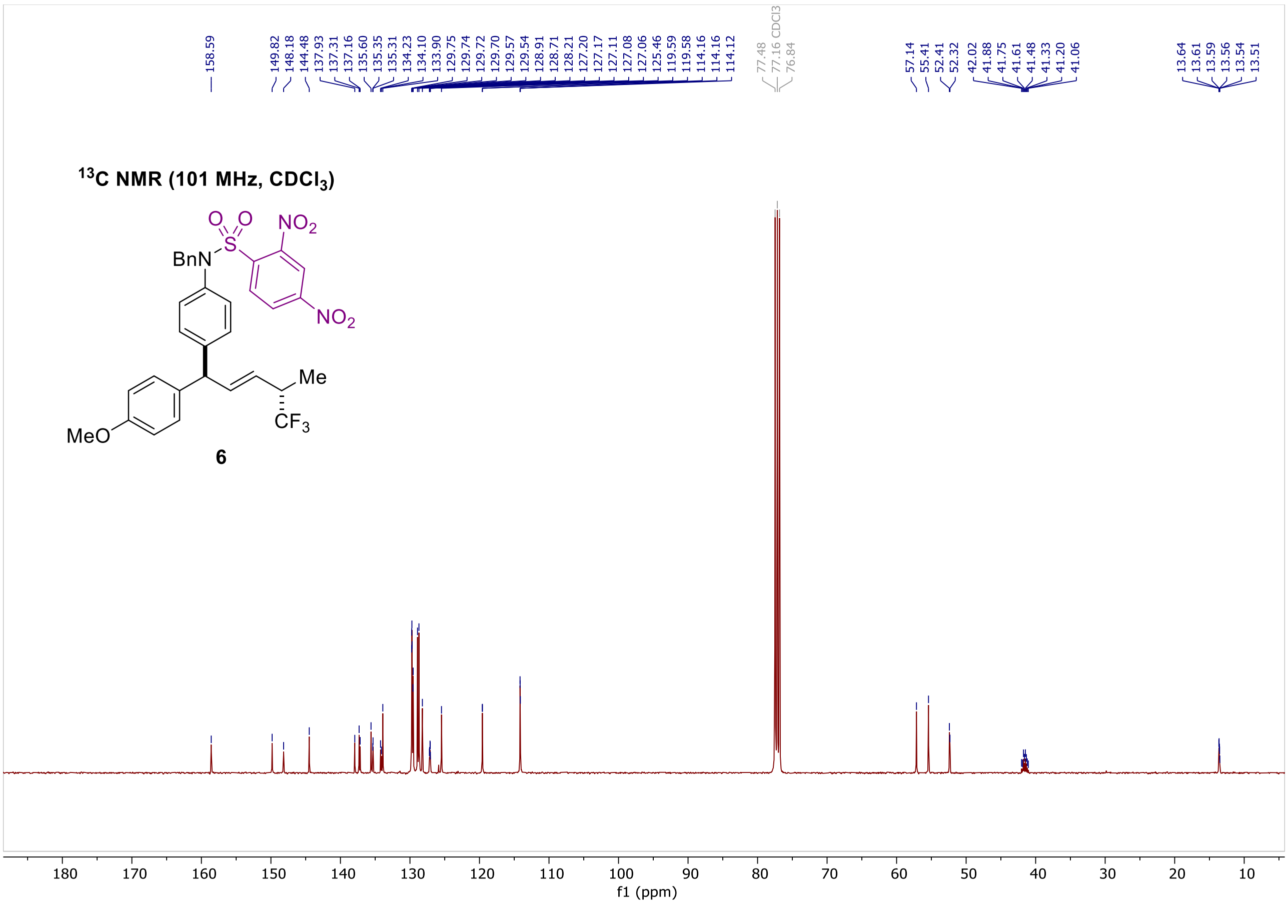

^{13}C NMR (101 MHz, $CDCl_3$)
BnN
NO_2
NO_2
Me
CF_3
MeO
6
f1 (ppm)

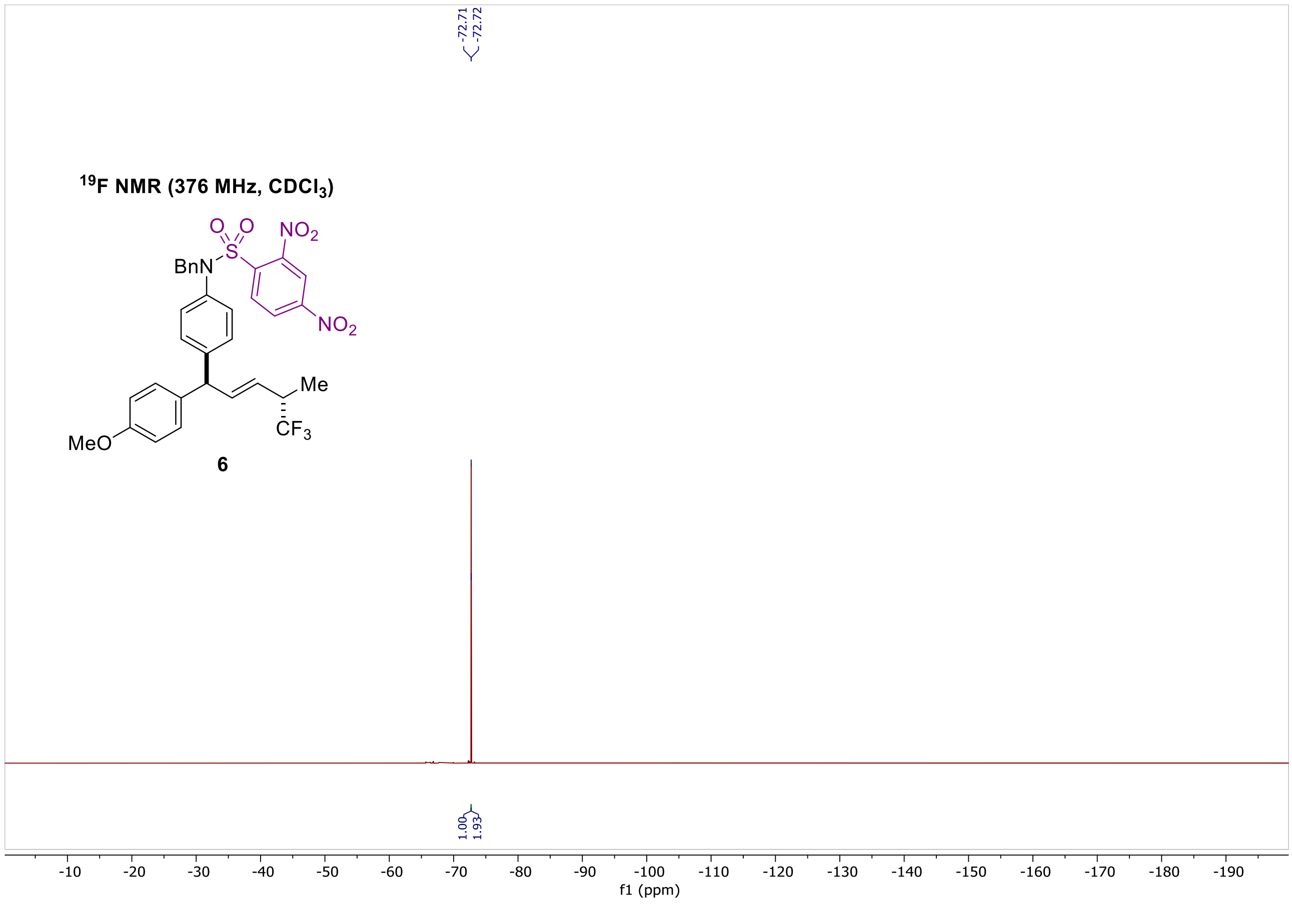

19F NMR (376 MHz, CDCl3)
6
-72.71
-72.72
f1 (ppm)

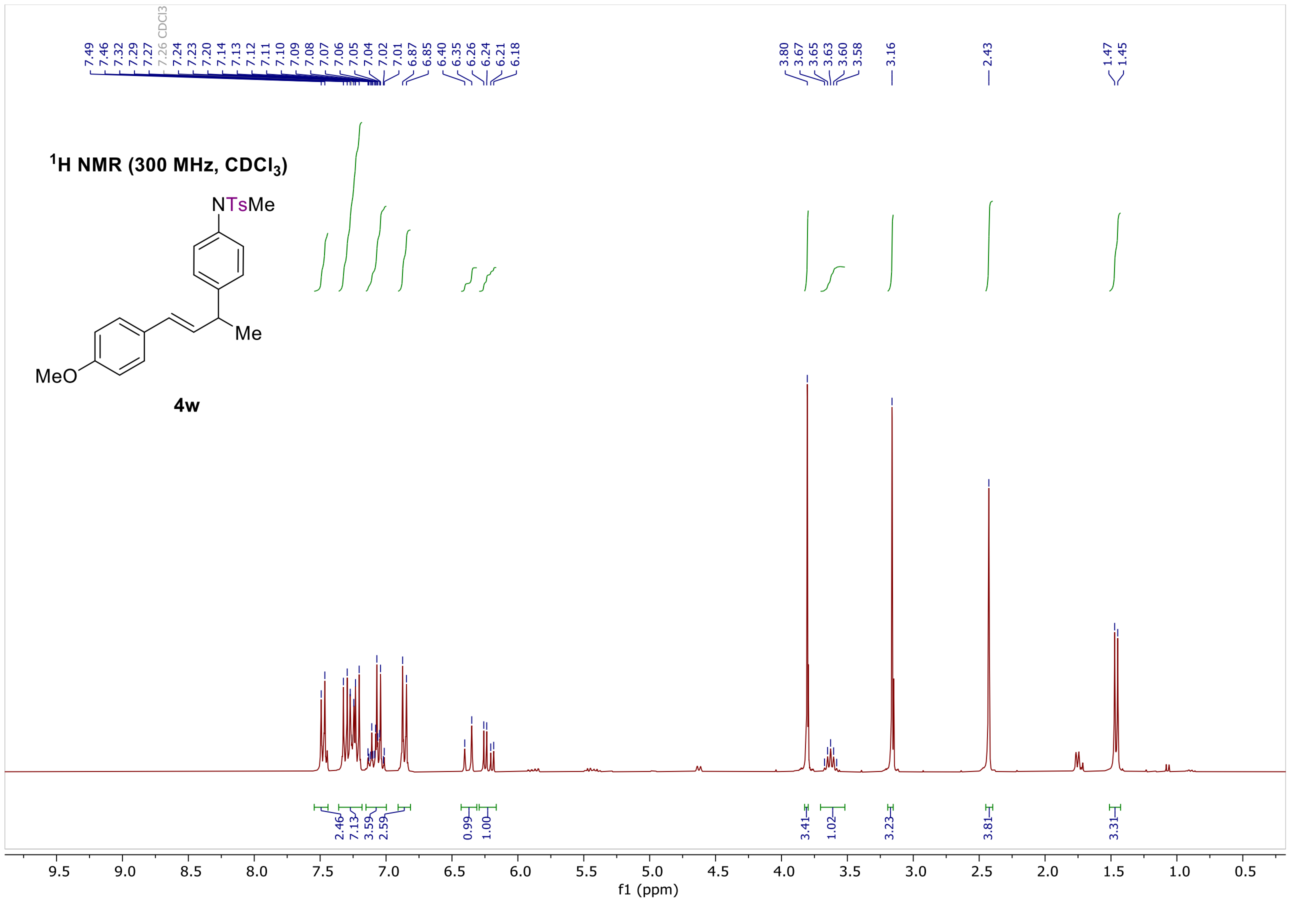

1H NMR (300 MHz, CDCl3)
4w
f1 (ppm)

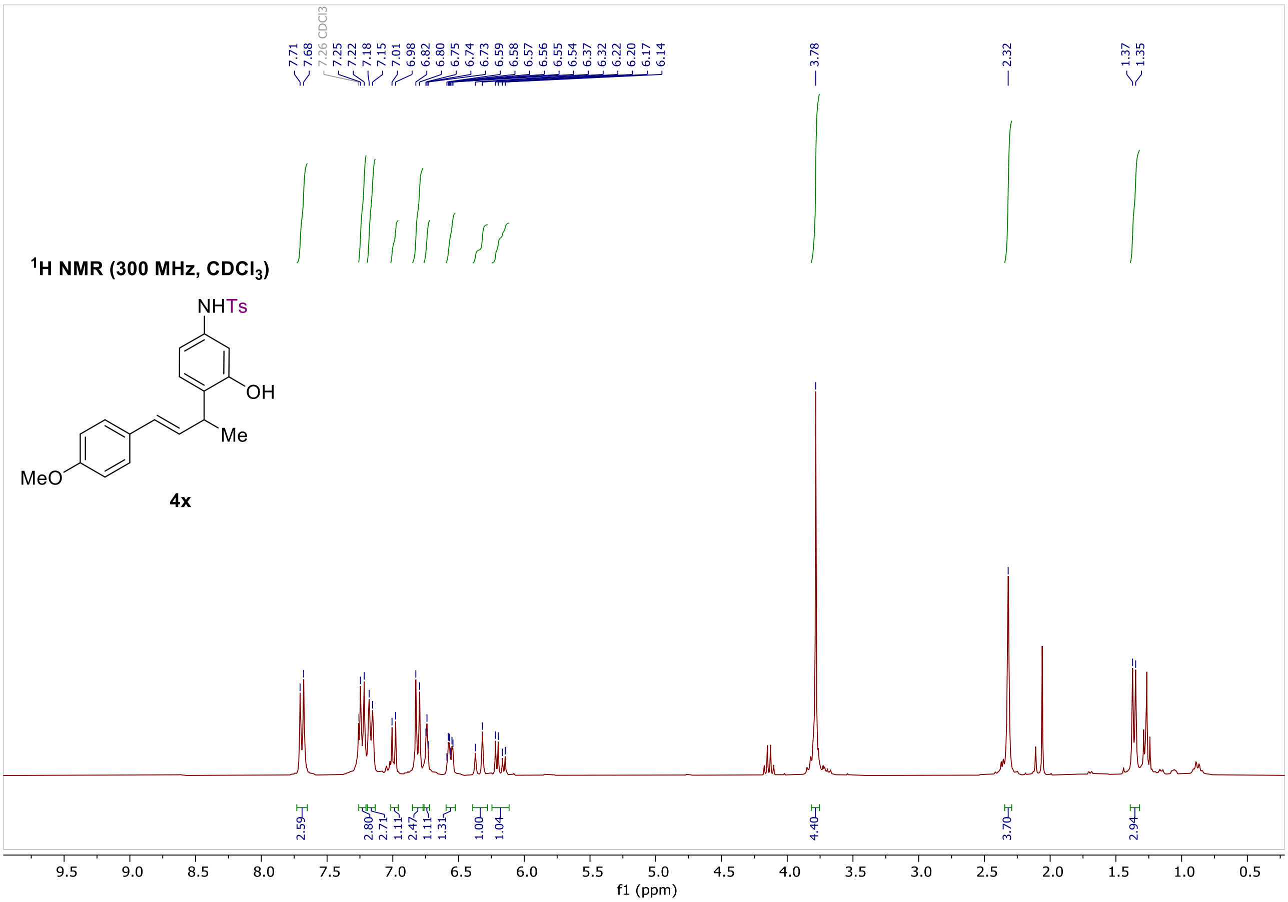
^{1}H NMR (300 MHz, CDCl$_3$)
NHTs
OH
Me
MeO
4x

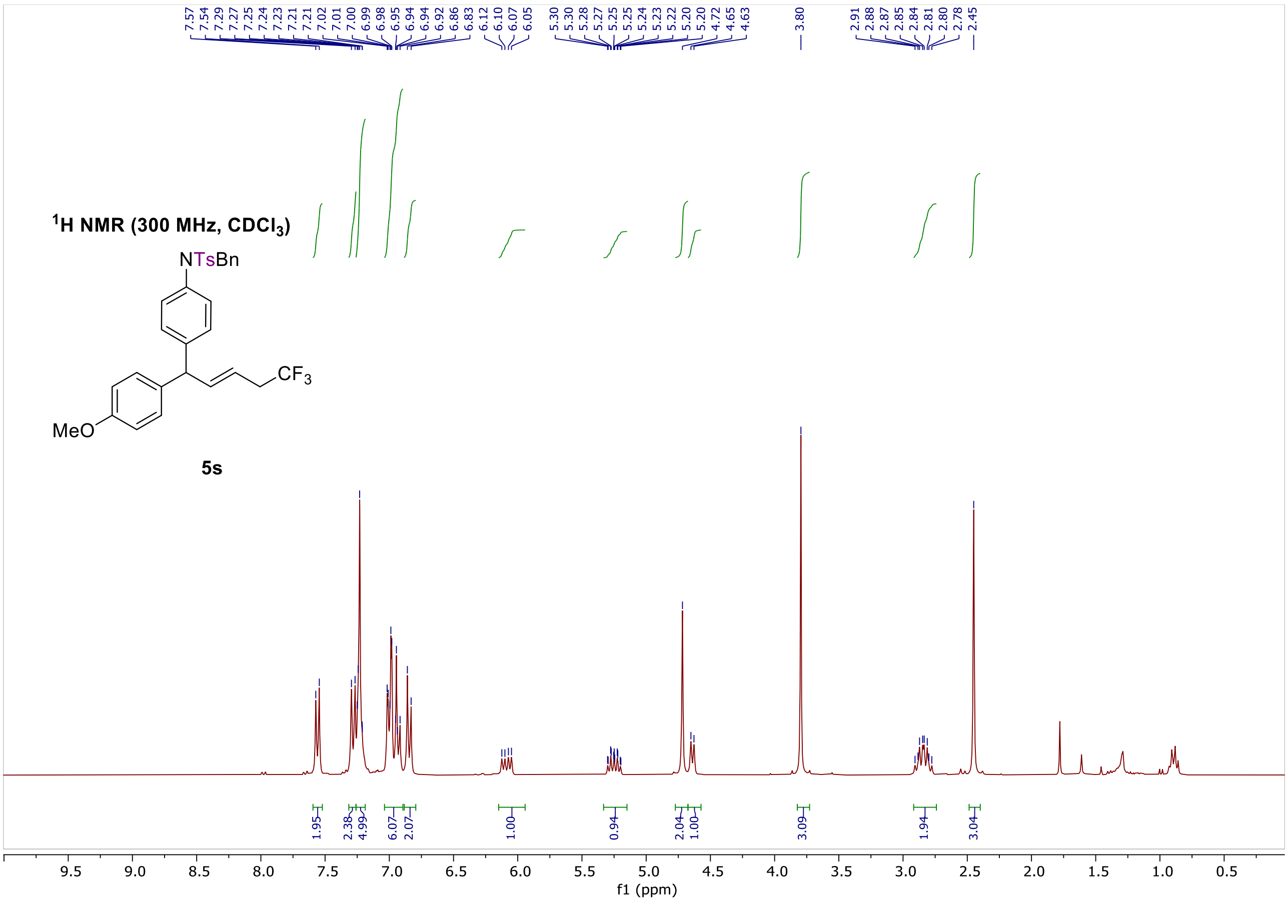
^{1}H NMR (300 MHz, CDCl$_3$)
NTsBn
CF$_3$
MeO
5s

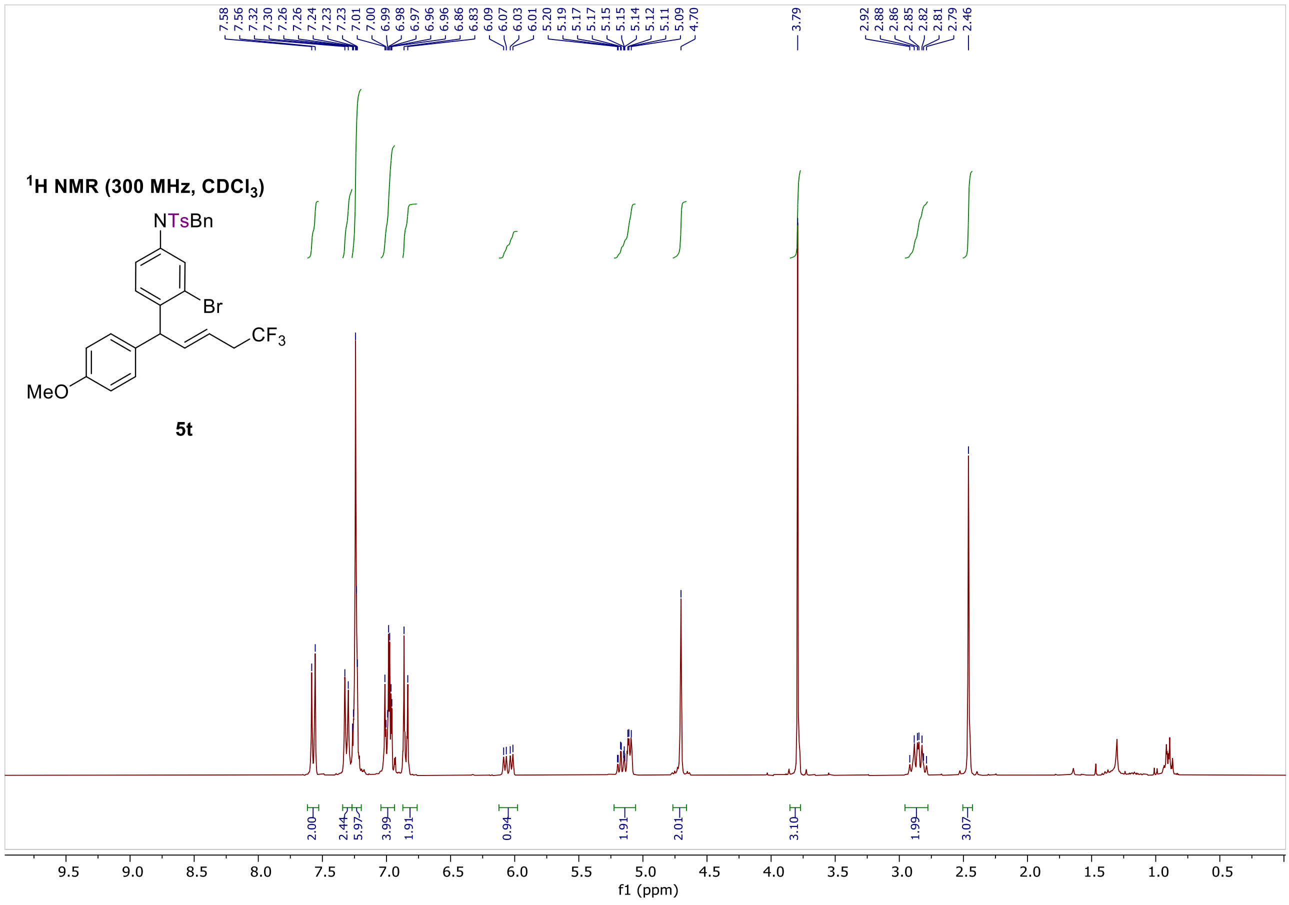

1H NMR (300 MHz, CDCl3)
NTsBn
Br
CF3
MeO
5t
f1 (ppm)

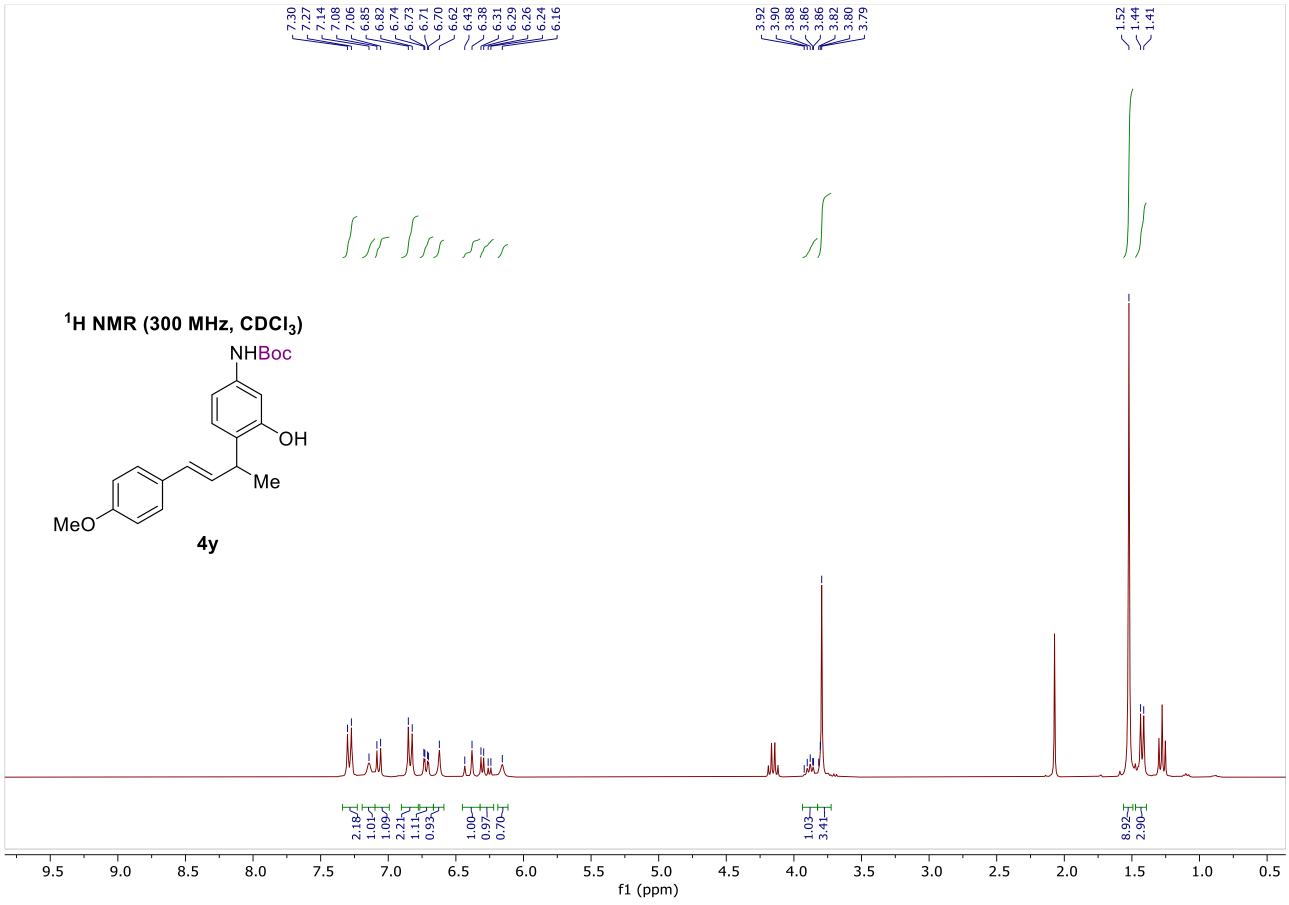

1H NMR (300 MHz, CDCl3)
NHBoc
OH
Me
MeO
4y
f1 (ppm)

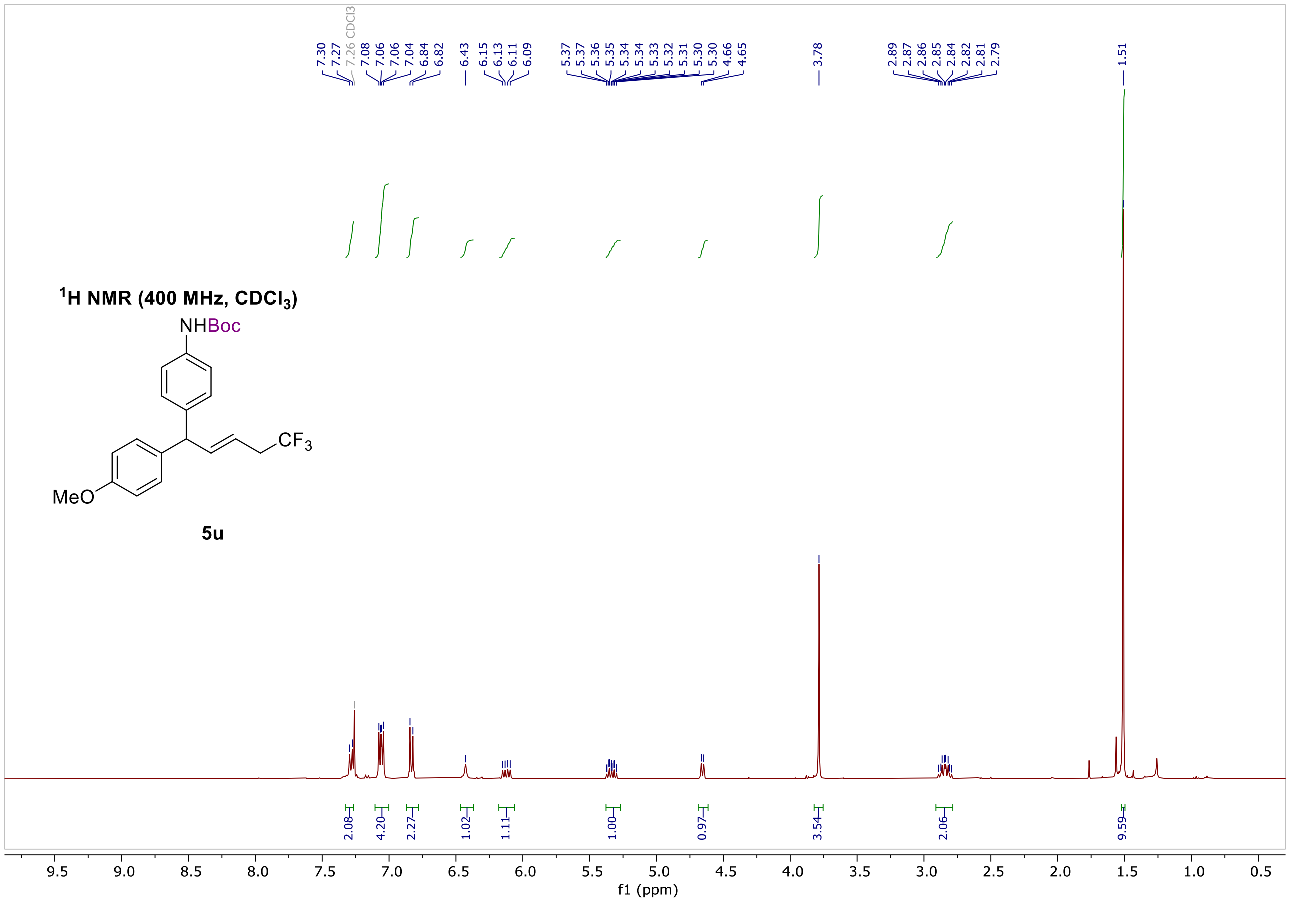

1H NMR (400 MHz, CDCl3)
NHBoc
CF3
MeO
5u
7.30
7.27
7.26 CDCl3
7.08
7.06
7.06
7.04
6.84
6.82
6.43
6.15
6.13
6.11
6.09
5.37
5.37
5.36
5.35
5.34
5.34
5.33
5.32
5.31
5.30
5.30
4.66
4.65
3.78
2.89
2.87
2.86
2.85
2.84
2.82
2.81
2.79
1.51
2.08
4.20
2.27
1.02
1.11
1.00
0.97
3.54
2.06
9.59
f1 (ppm)

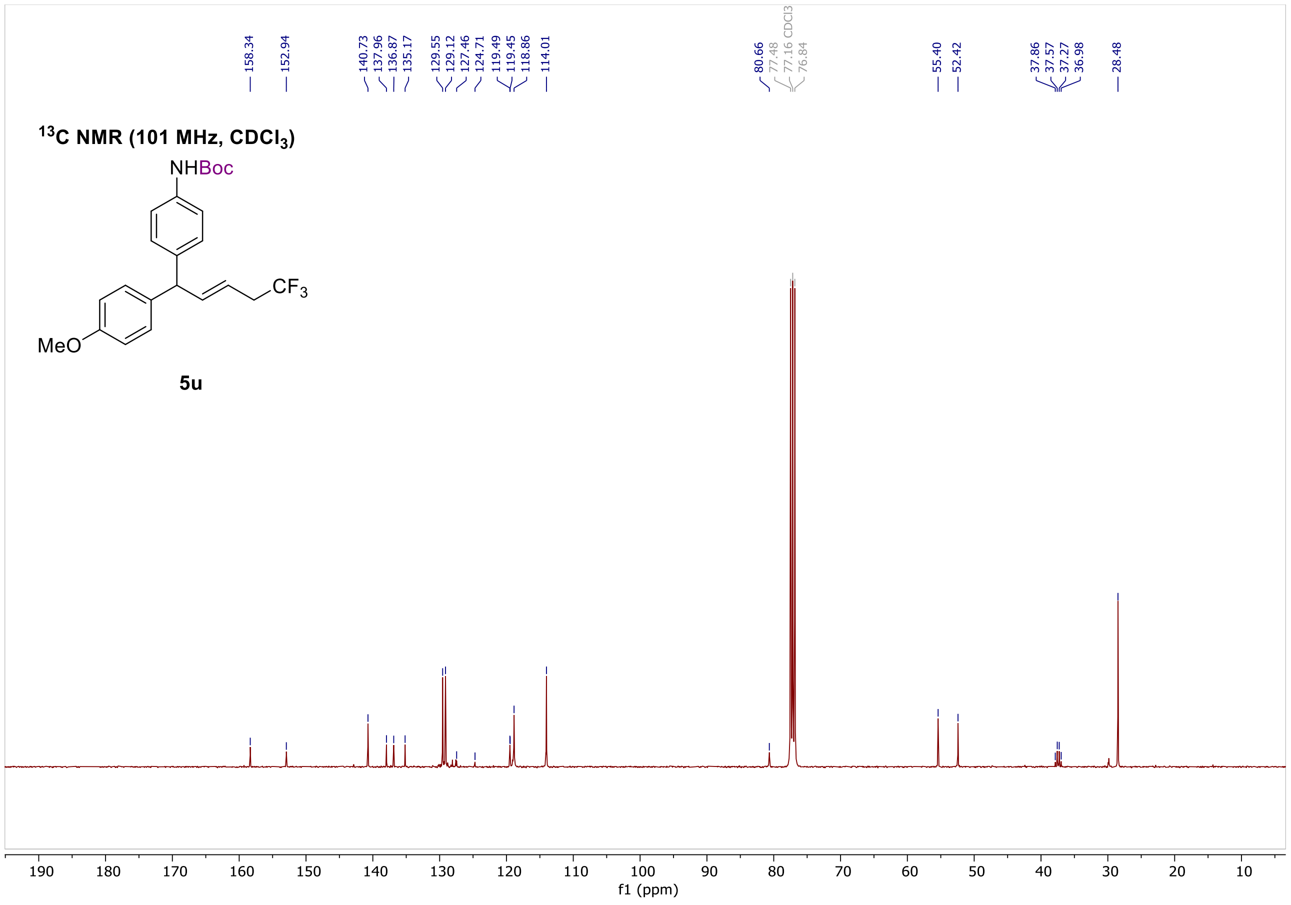

13C NMR (101 MHz, CDCl3)
NHBoc
CF3
MeO
5u
158.34
152.94
140.73
137.96
136.87
135.17
129.55
129.12
127.46
124.71
119.49
119.45
118.86
114.01
80.66
77.48
77.16 CDCl3
76.84
55.40
52.42
37.86
37.57
37.27
36.98
28.48
f1 (ppm)

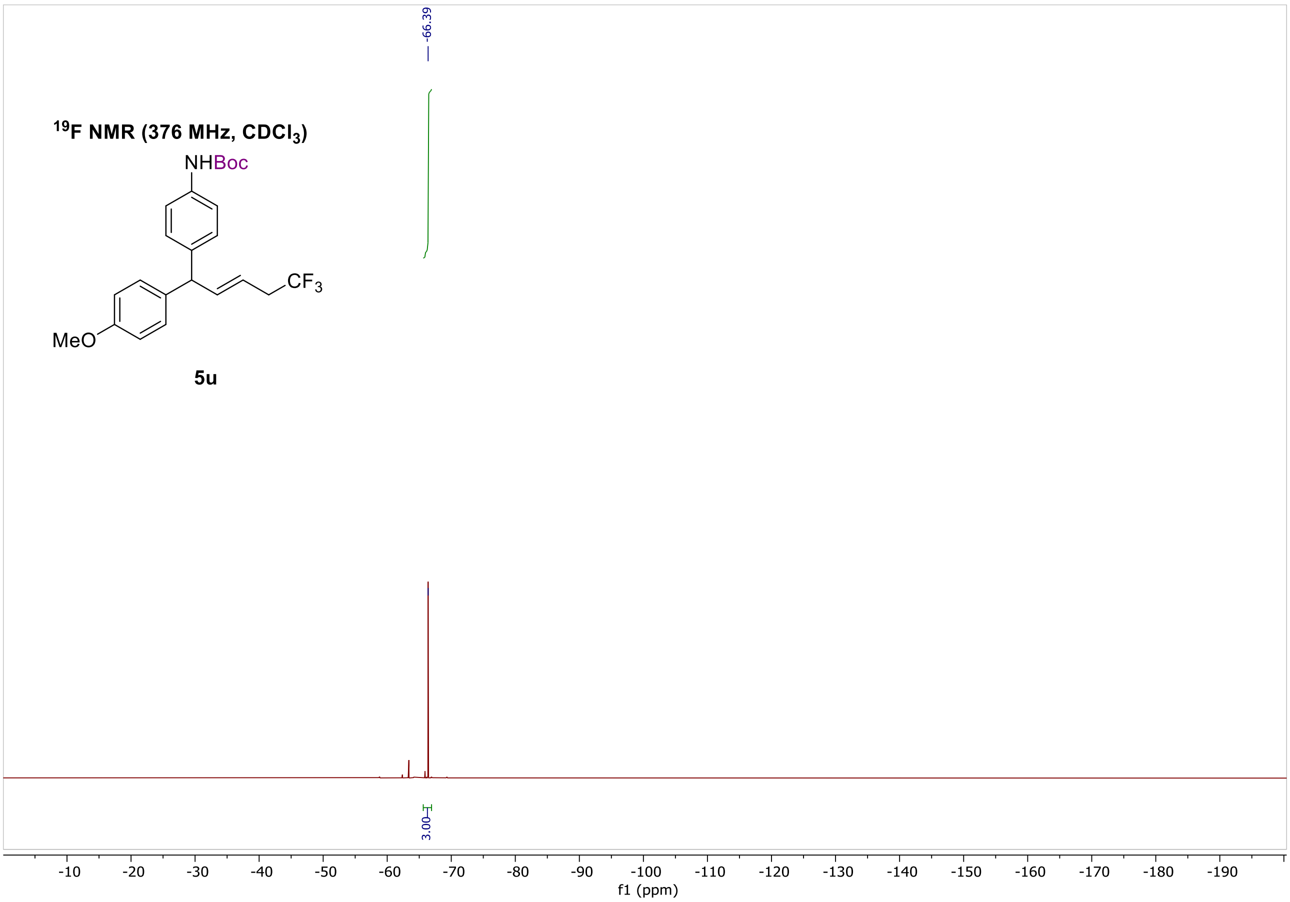

-66.39
19F NMR (376 MHz, CDCl3)
NHBoc
CF3
MeO
5u
3.00
-10 -20 -30 -40 -50 -60 -70 -80 -90 -100 -110 -120 -130 -140 -150 -160 -170 -180 -190
f1 (ppm)

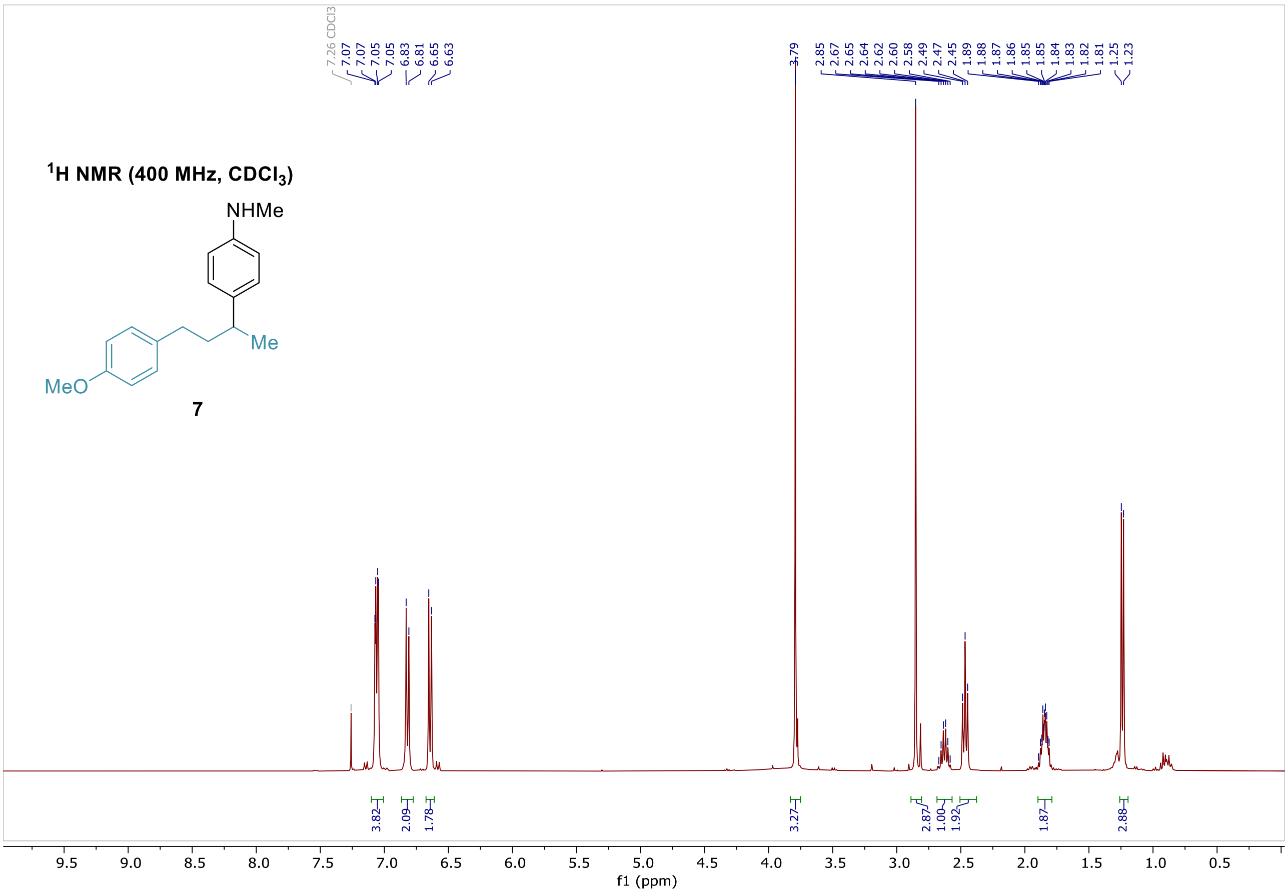

7.26 CDCl3
7.07 7.07 7.05 7.05 6.83 6.81 6.65 6.63
3.79
2.85 2.67 2.65 2.64 2.62 2.60 2.58 2.49 2.47 2.45 1.89 1.88 1.87 1.86 1.85 1.84 1.83 1.82 1.81 1.25 1.23
1H NMR (400 MHz, CDCl3)
NHMe
Me
MeO
7
3.82 2.09 1.78 3.27 2.87 1.00 1.92 1.87 2.88
9.5 9.0 8.5 8.0 7.5 7.0 6.5 6.0 5.5 5.0 4.5 4.0 3.5 3.0 2.5 2.0 1.5 1.0 0.5
f1 (ppm)

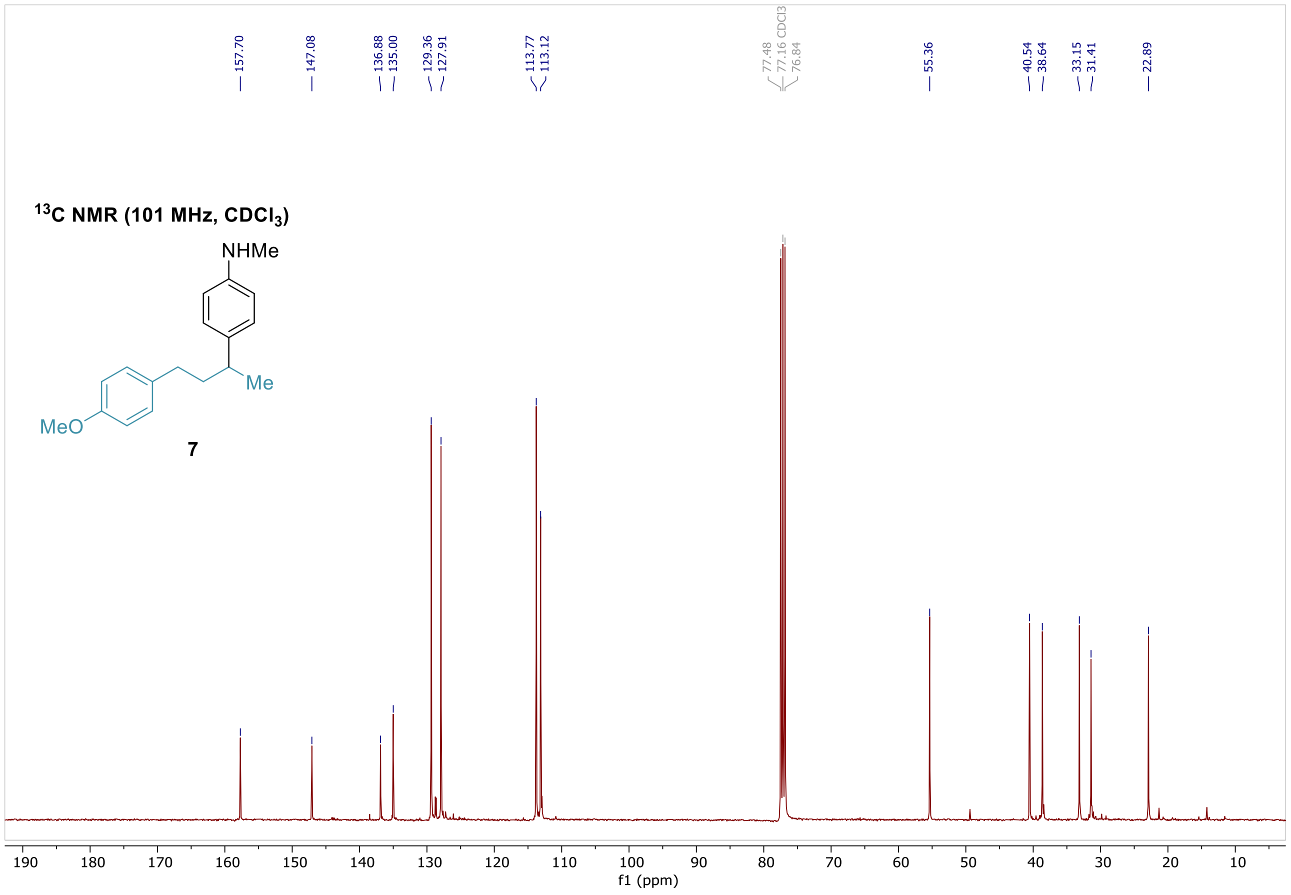

157.70
147.08
136.88
135.00
129.36
127.91
113.77
113.12
77.48
77.16 CDCl3
76.84
55.36
40.54
38.64
33.15
31.41
22.89
^{13}C NMR (101 MHz, CDCl$_3$)
NHMe
Me
MeO
7
f1 (ppm)

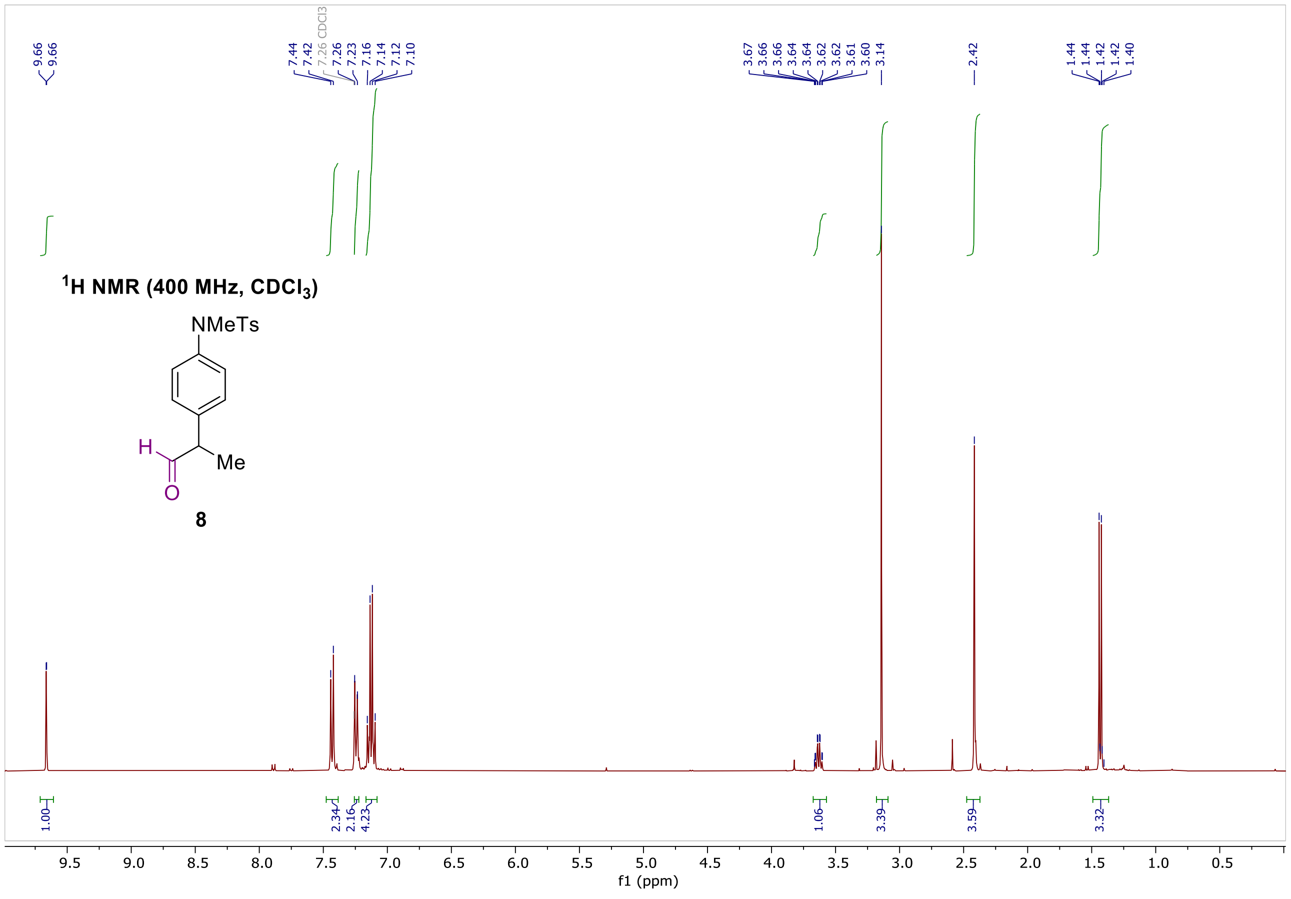

9.66
9.66
7.44
7.42
7.26 CDCl3
7.26
7.23
7.16
7.14
7.12
7.10
3.67
3.66
3.66
3.64
3.62
3.62
3.61
3.60
3.14
2.42
1.44
1.44
1.42
1.42
1.40
^{1}H NMR (400 MHz, CDCl$_3$)
NMeTs
H
Me
O
8
1.00
2.34
2.16
4.23
1.06
3.39
3.59
3.32
f1 (ppm)

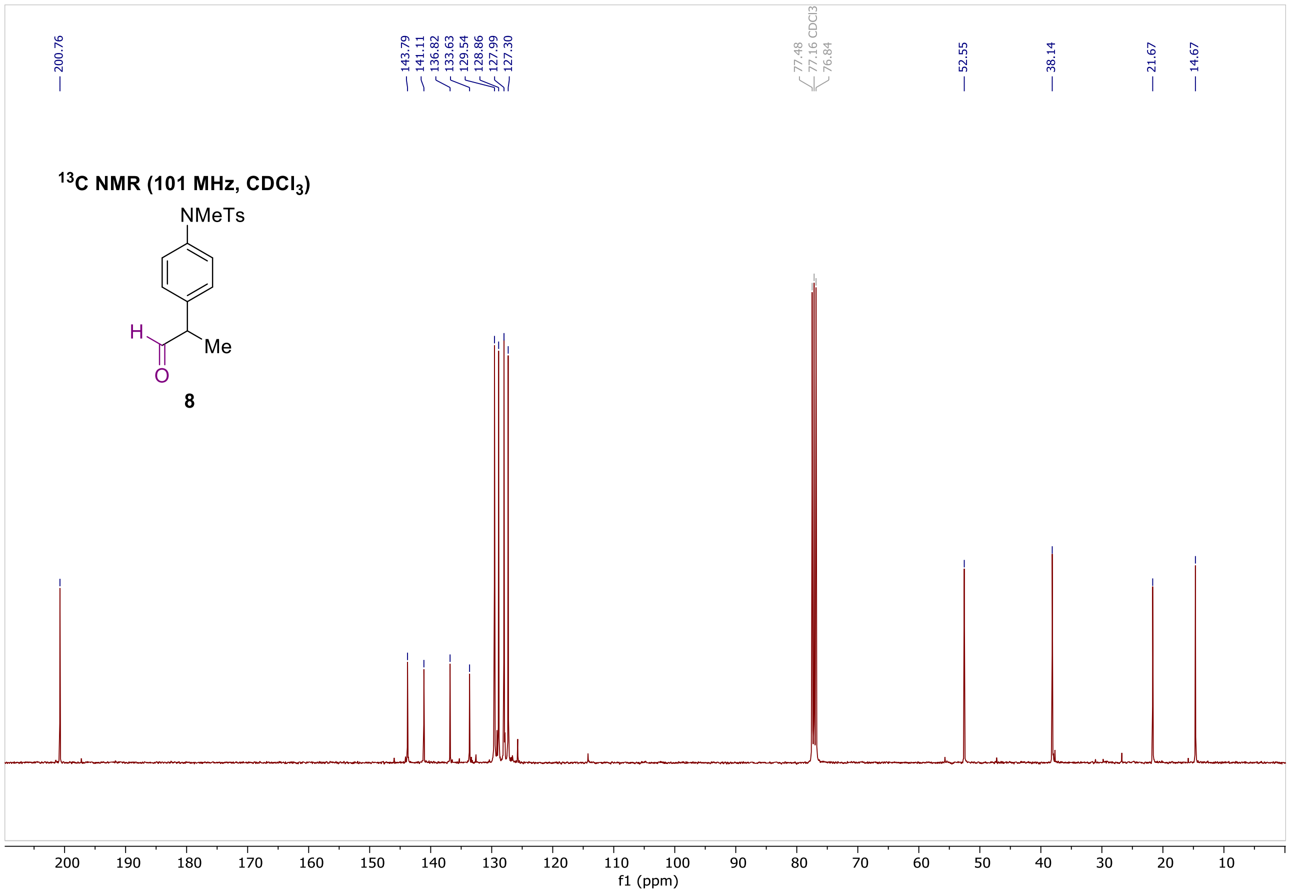

^{13}C NMR (101 MHz, CDCl$_3$)
NMeTs
H
O
Me
8
f1 (ppm)

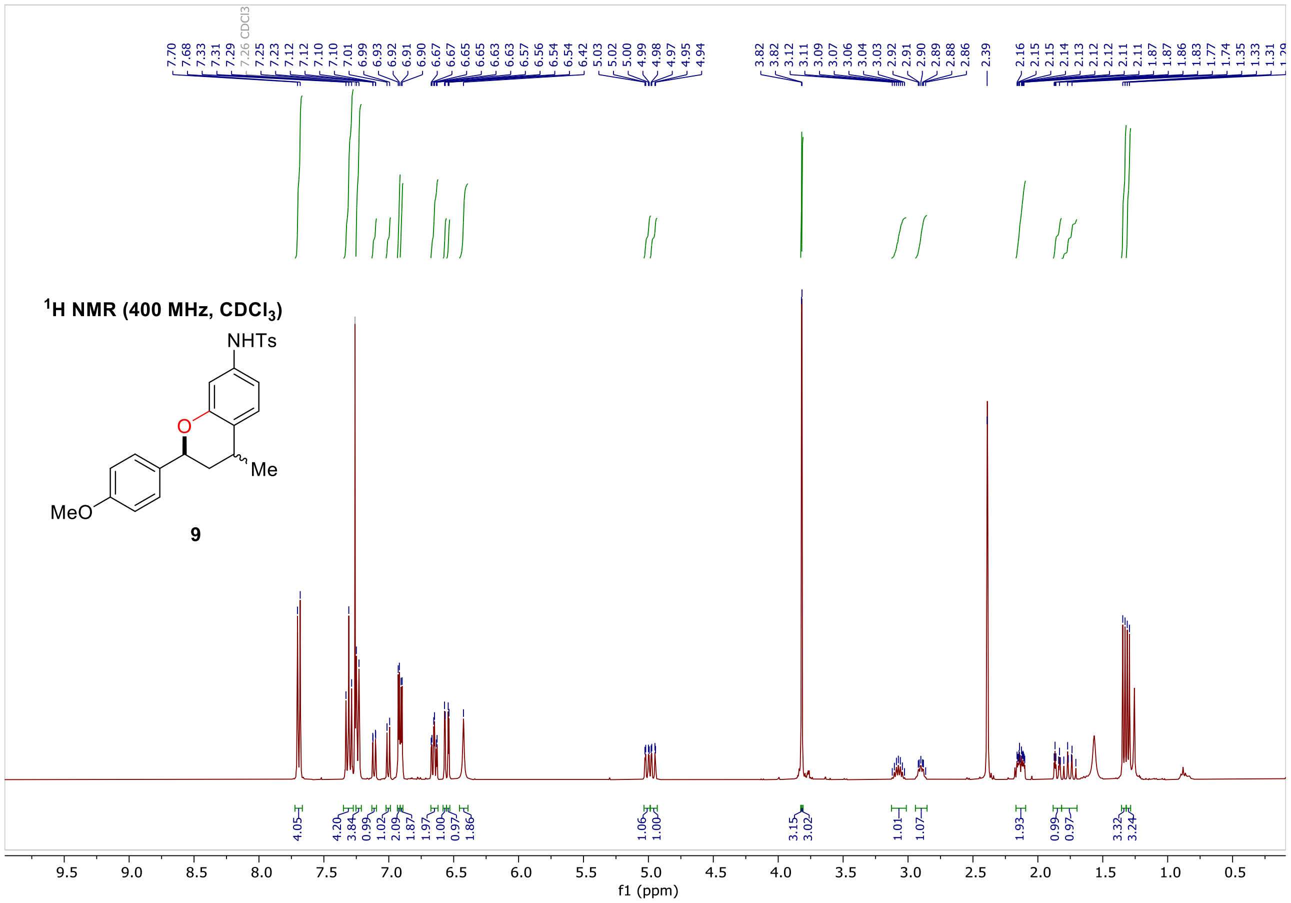

^{1}H NMR (400 MHz, CDCl$_3$)
NHTs
O
Me
MeO
9
f1 (ppm)

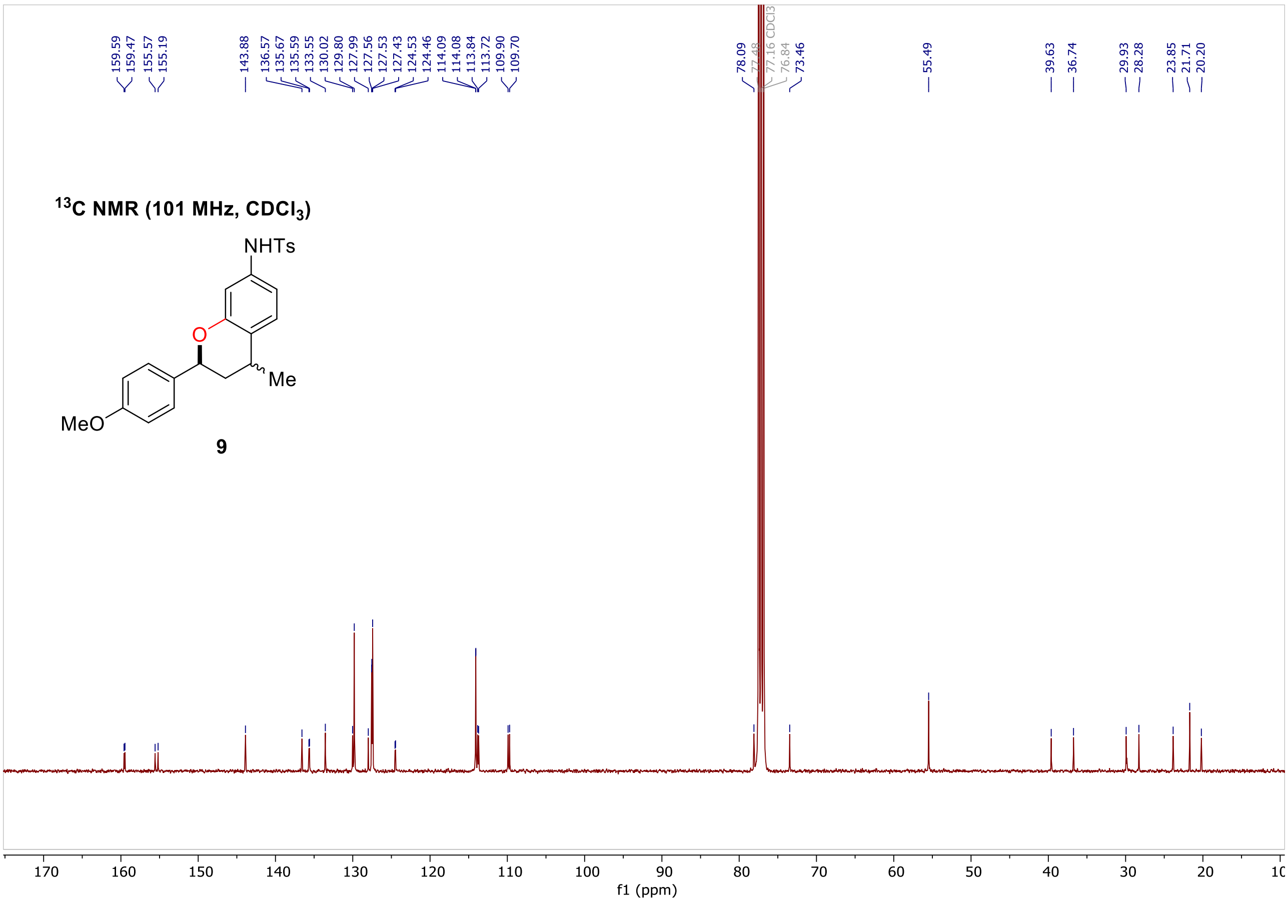
13C NMR (101 MHz, CDCl3)
NHTs
MeO
Me
9
159.59
159.47
155.57
155.19
143.88
136.57
135.67
135.59
133.55
130.02
129.80
127.99
127.56
127.53
127.43
124.53
124.46
114.09
114.08
113.84
113.72
109.90
109.70
78.09
77.48
77.16 CDCl3
76.84
73.46
55.49
39.63
36.74
29.93
28.28
23.85
21.71
20.20
f1 (ppm)

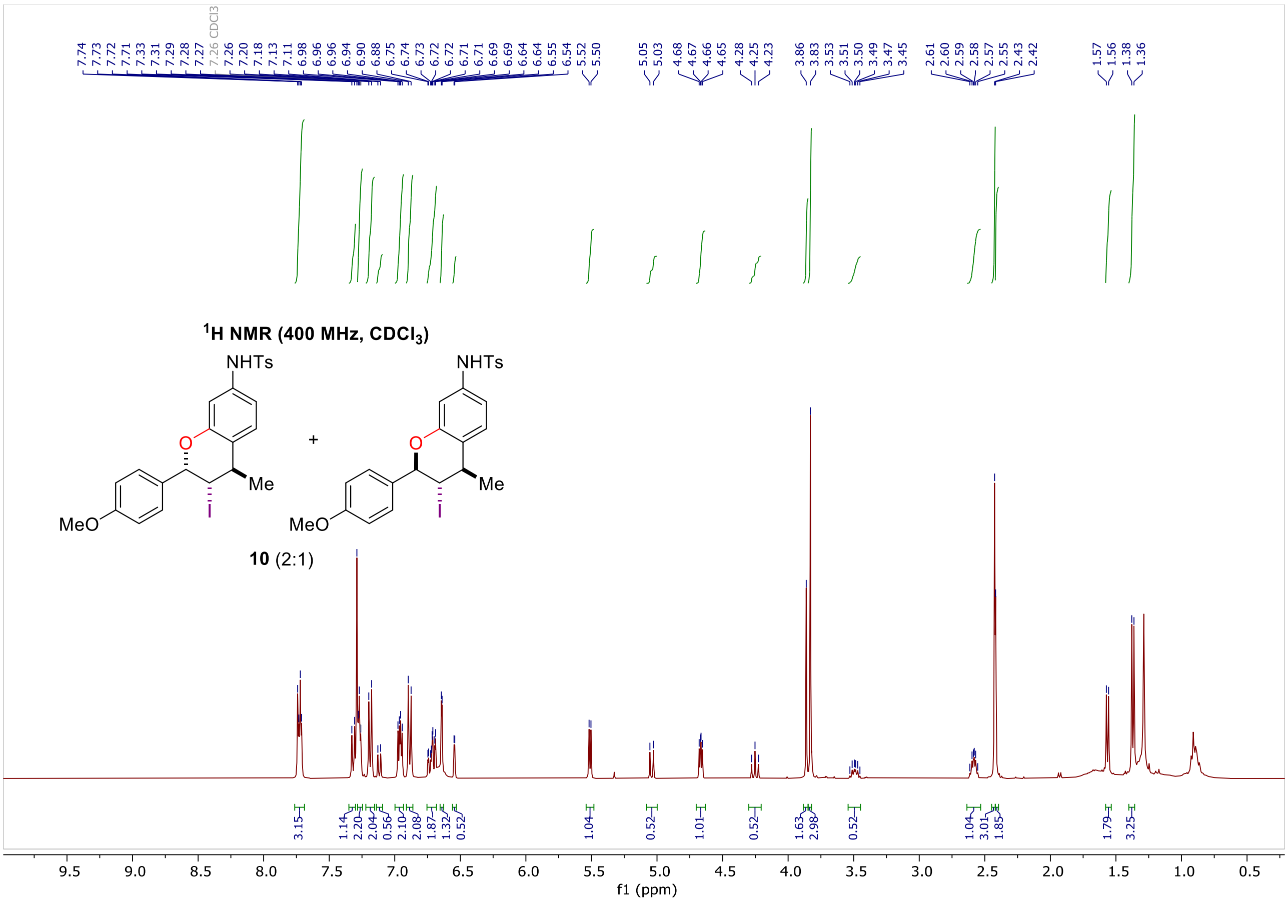
1H NMR (400 MHz, CDCl3)
NHTs
MeO
Me
I
+
10 (2:1)
7.74
7.73
7.72
7.71
7.33
7.31
7.29
7.28
7.27
7.26 CDCl3
7.26
7.20
7.18
7.13
7.11
6.98
6.96
6.96
6.94
6.90
6.88
6.75
6.74
6.73
6.72
6.72
6.71
6.71
6.69
6.64
6.64
6.55
6.54
5.52
5.50
5.05
5.03
4.68
4.67
4.66
4.65
4.28
4.25
4.23
3.86
3.83
3.53
3.51
3.50
3.49
3.47
3.45
2.61
2.60
2.59
2.58
2.57
2.55
2.43
2.42
1.57
1.56
1.38
1.36
3.15
1.14
2.20
2.04
0.56
2.10
2.08
1.87
1.32
0.52
1.04
0.52
1.01
0.52
1.63
2.98
0.52
1.04
3.01
1.85
1.79
3.25
f1 (ppm)

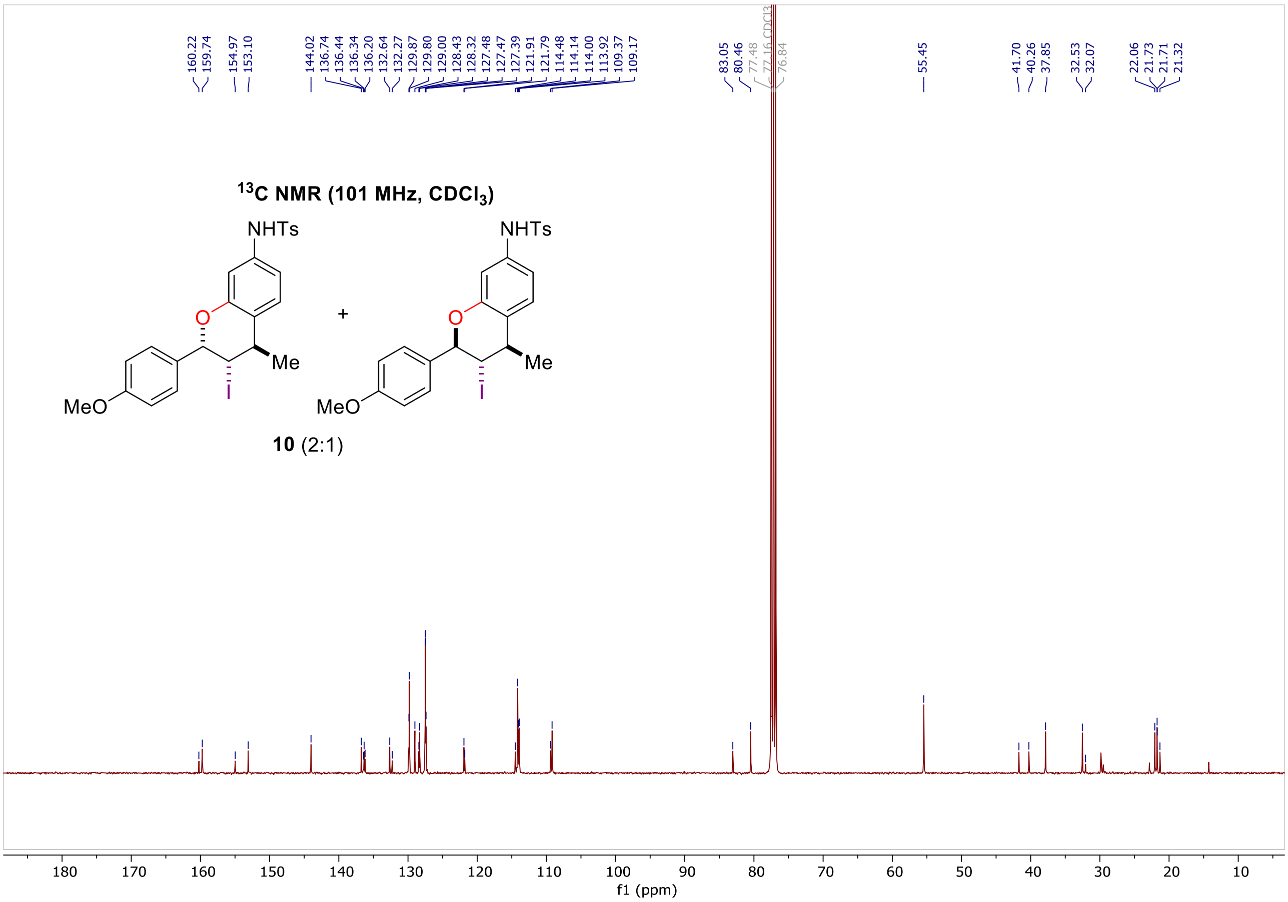
160.22
159.74
154.97
153.10
144.02
136.74
136.44
136.34
136.20
132.64
132.27
129.87
129.80
129.00
128.43
128.32
127.48
127.47
127.39
121.91
121.79
114.48
114.14
114.00
113.92
109.37
109.17
83.05
80.46
77.48
77.16 CDCl3
76.84
55.45
41.70
40.26
37.85
32.53
32.07
22.06
21.73
21.71
21.32
^{13}C NMR (101 MHz, CDCl$_3$)
NHTs
NHTs
O
O
Me
Me
MeO
MeO
I
I
+
10 (2:1)
180
170
160
150
140
130
120
110
100
90
80
70
60
50
40
30
20
10
f1 (ppm)

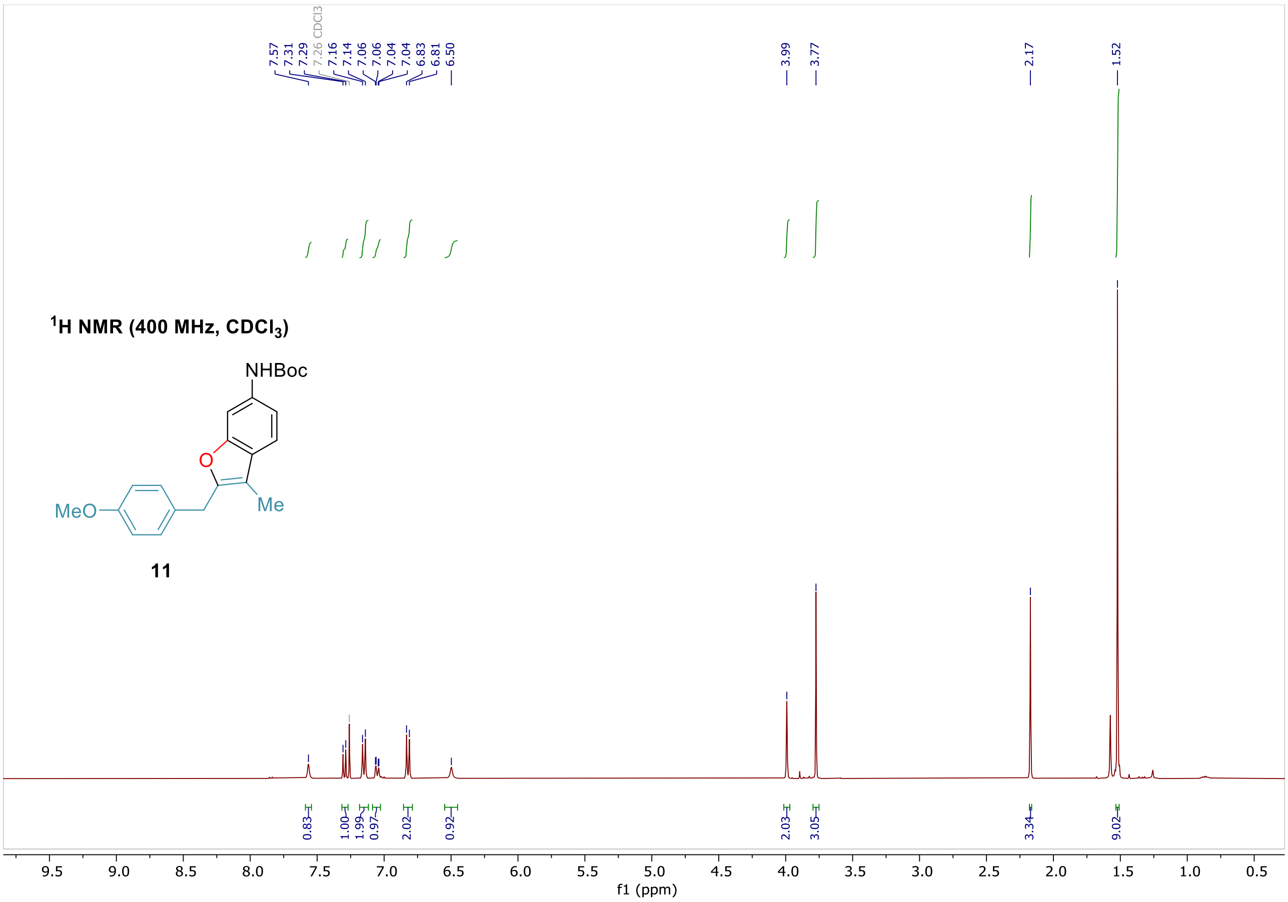
7.57
7.31
7.29
7.26 CDCl3
7.16
7.14
7.06
7.06
7.04
7.04
6.83
6.81
6.50
3.99
3.77
2.17
1.52
^{1}H NMR (400 MHz, CDCl$_3$)
NHBoc
O
MeO
Me
11
0.83
1.00
1.99
0.97
2.02
0.92
2.03
3.05
3.34
9.02
9.5
9.0
8.5
8.0
7.5
7.0
6.5
6.0
5.5
5.0
4.5
4.0
3.5
3.0
2.5
2.0
1.5
1.0
0.5
f1 (ppm)

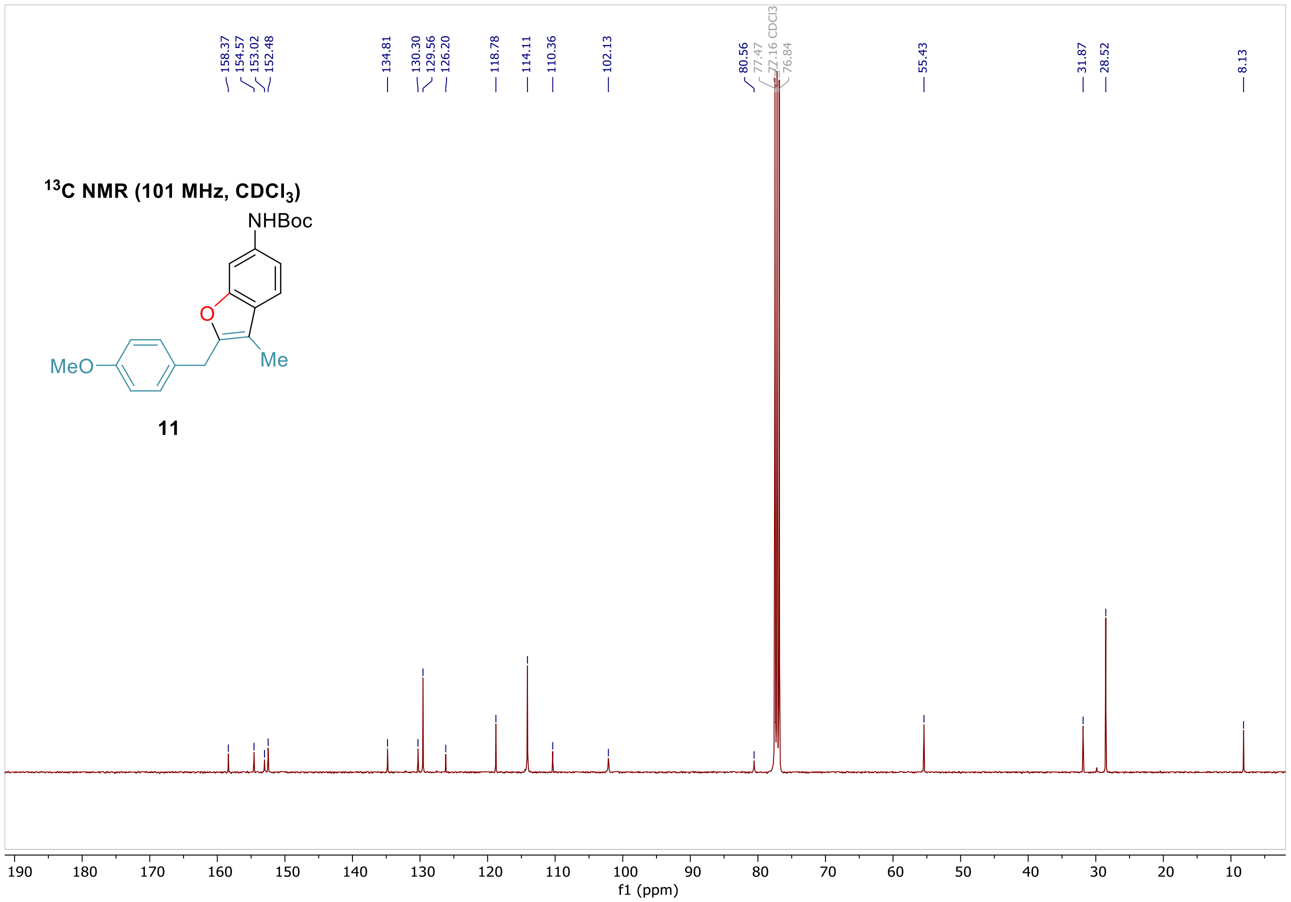
158.37
154.57
153.02
152.48
134.81
130.30
129.56
126.20
118.78
114.11
110.36
102.13
80.56
77.47
77.16 CDCl3
76.84
55.43
31.87
28.52
8.13
^{13}C NMR (101 MHz, $CDCl_3$)
NHBoc
O
MeO
Me
11
f1 (ppm)

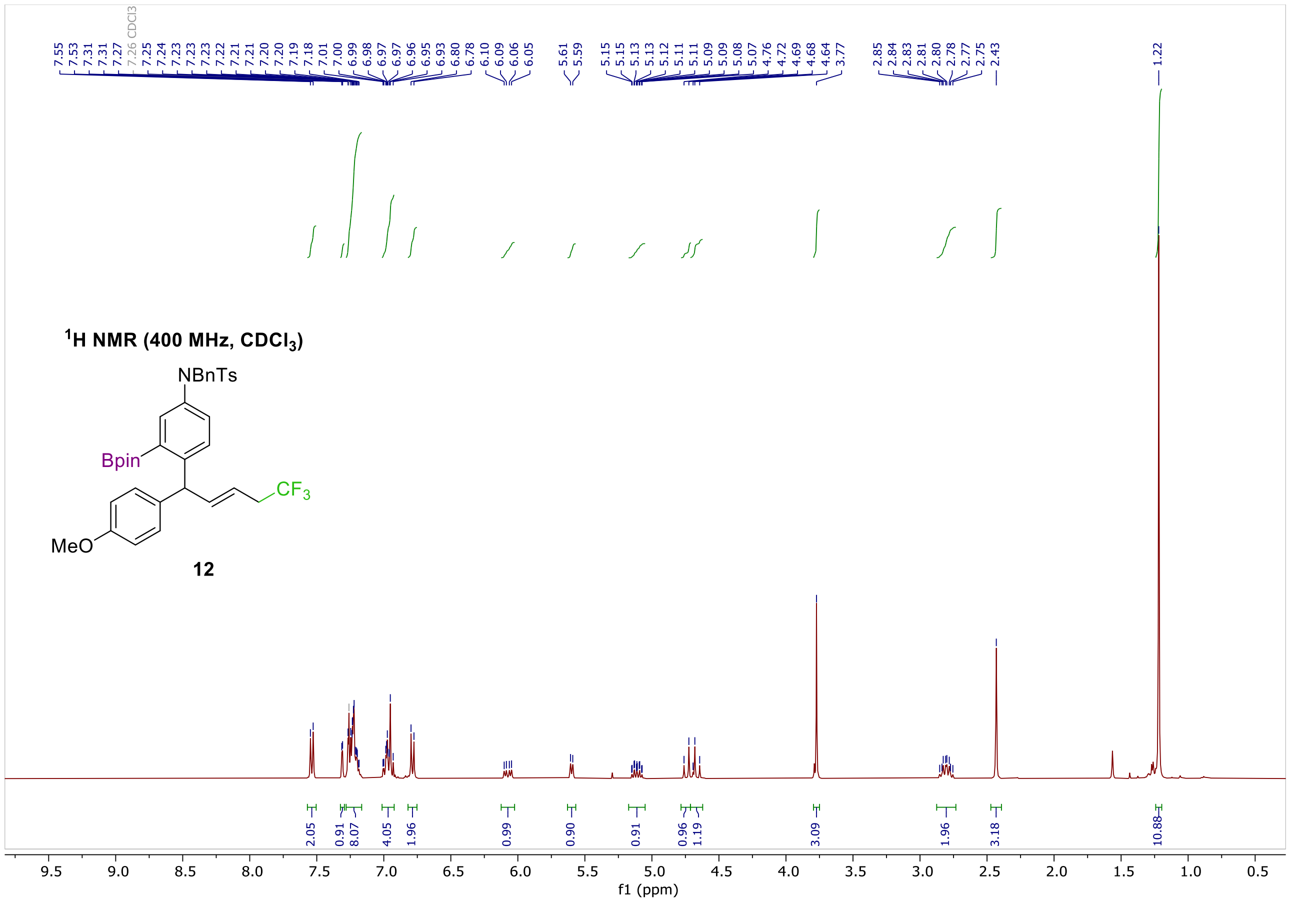
^{1}H NMR (400 MHz, $CDCl_3$)
NBnTs
Bpin
CF_3
MeO
12
2.05
0.91
8.07
4.05
1.96
0.99
0.90
0.91
0.96
1.19
3.09
1.96
3.18
10.88
f1 (ppm)

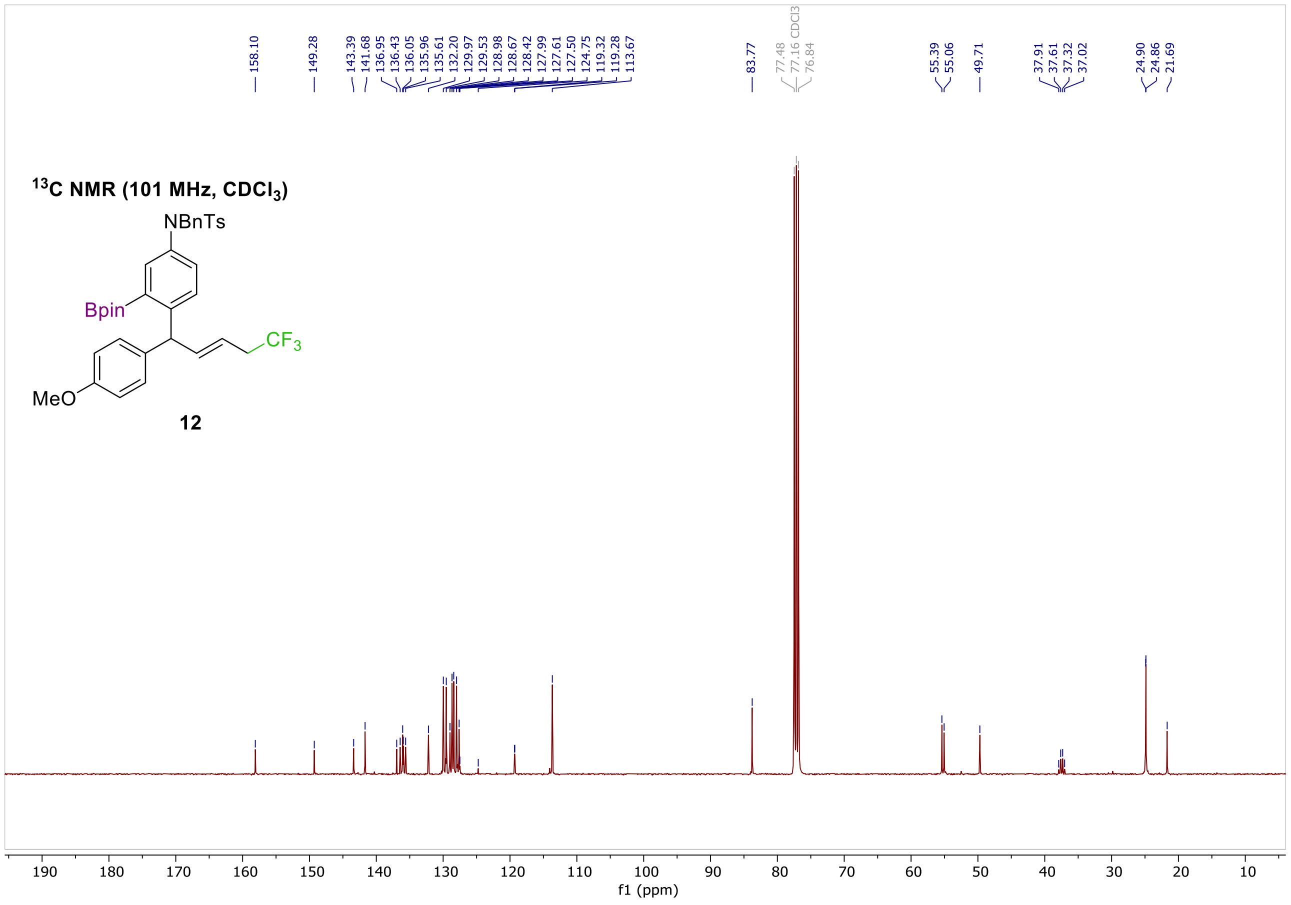

158.10
149.28
143.39
141.68
136.95
136.43
136.05
135.96
135.61
132.20
129.97
129.53
128.98
128.67
128.42
127.99
127.61
127.50
124.75
119.32
119.28
113.67
83.77
77.48
77.16 CDCl3
76.84
55.39
55.06
49.71
37.91
37.61
37.32
37.02
24.90
24.86
21.69
^{13}C NMR (101 MHz, CDCl$_3$)
NBnTs
Bpin
CF$_3$
MeO
12
f1 (ppm)

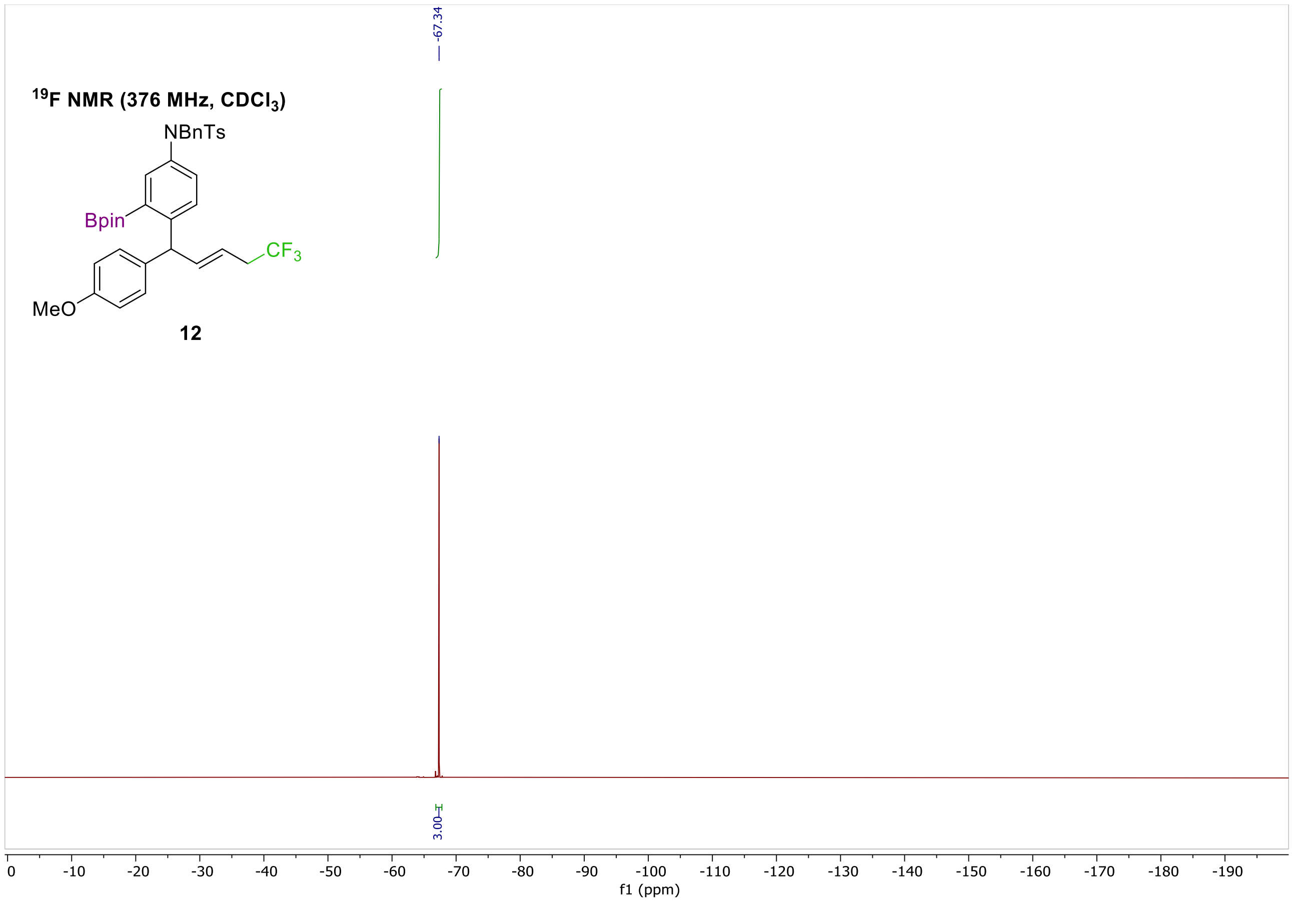

-67.34
^{19}F NMR (376 MHz, CDCl$_3$)
NBnTs
Bpin
CF$_3$
MeO
12
3.00
f1 (ppm)

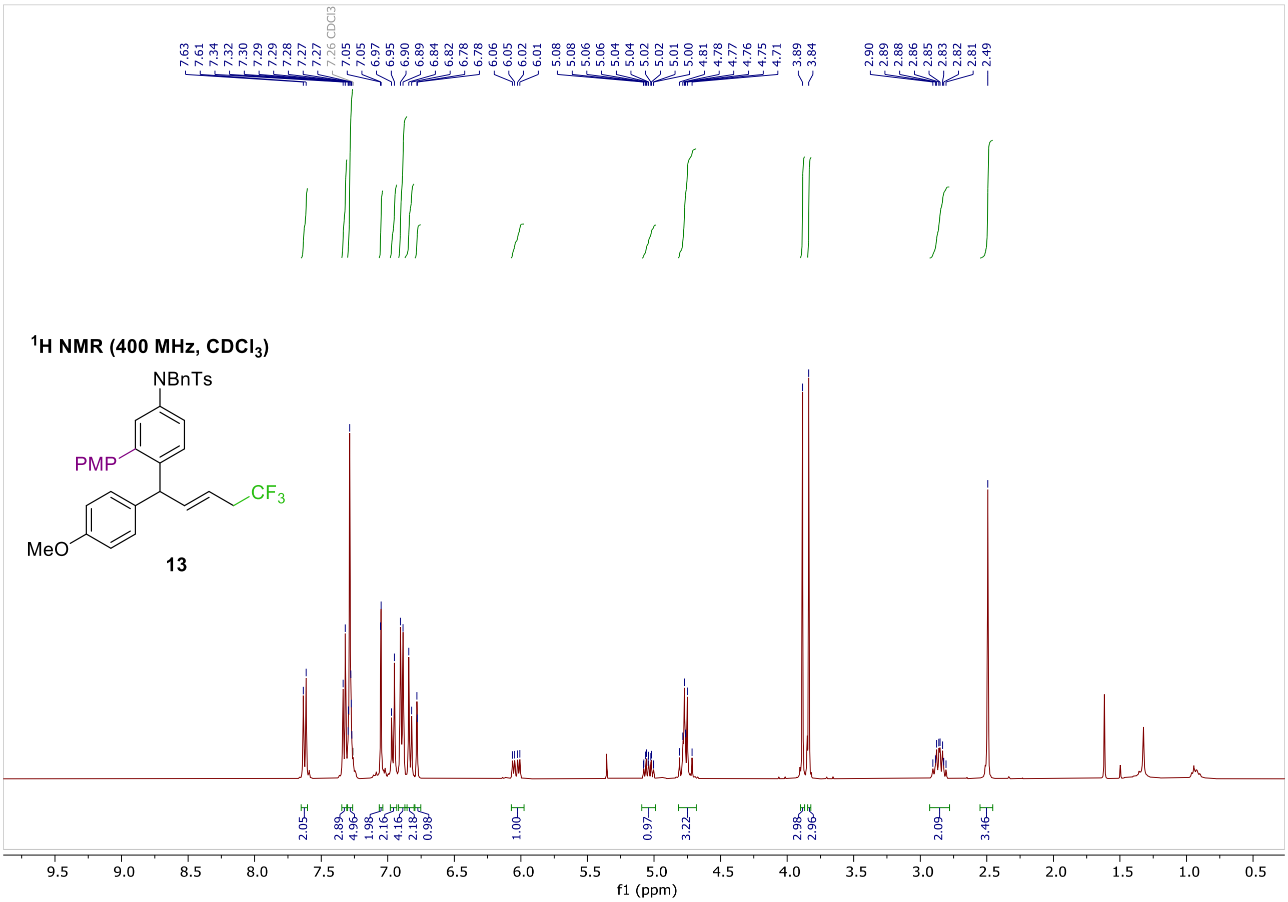
7.63 7.61 7.34 7.32 7.30 7.29 7.29 7.28 7.27 7.27 7.26 CDCl3 7.05 7.05 6.97 6.95 6.90 6.89 6.84 6.82 6.78 6.78 6.06 6.05 6.02 6.01 5.08 5.08 5.06 5.06 5.04 5.04 5.02 5.02 5.01 5.00 4.81 4.78 4.77 4.76 4.75 4.71 3.89 3.84 2.90 2.89 2.88 2.86 2.85 2.83 2.82 2.81 2.49
^{1}H NMR (400 MHz, CDCl$_3$)
NBnTs
PMP
CF$_3$
MeO
13
2.05 2.89 4.96 1.98 2.16 4.16 2.18 0.98 1.00 0.97 3.22 2.98 2.96 2.09 3.46
9.5 9.0 8.5 8.0 7.5 7.0 6.5 6.0 5.5 5.0 4.5 4.0 3.5 3.0 2.5 2.0 1.5 1.0 0.5
f1 (ppm)

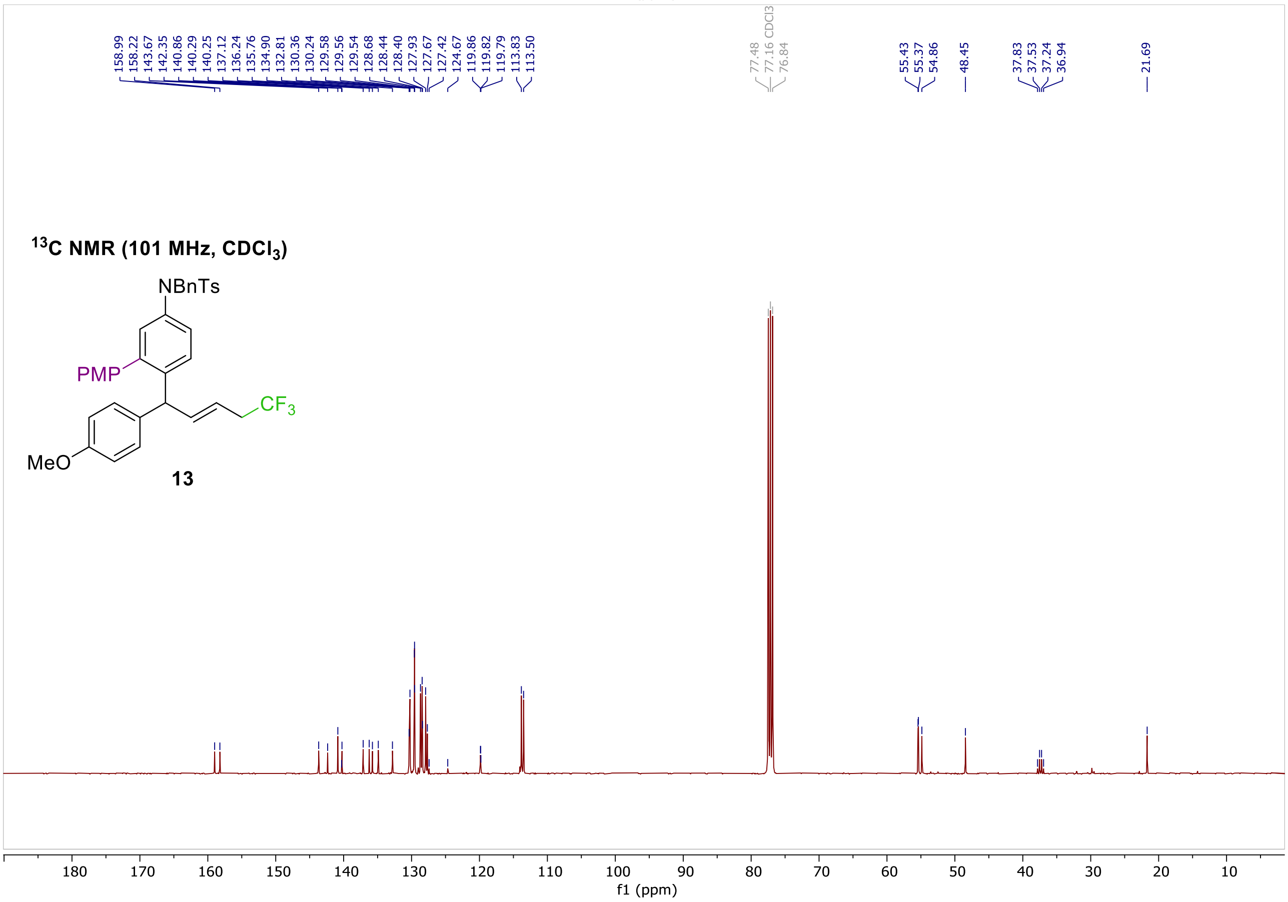
158.99 158.22 143.67 142.35 140.86 140.29 140.25 137.12 136.24 135.76 134.90 132.81 130.36 130.24 129.58 129.56 129.54 128.68 128.44 128.40 127.93 127.67 127.42 124.67 119.86 119.82 119.79 113.83 113.50
77.48 77.16 CDCl3 76.84
55.43 55.37 54.86 48.45 37.83 37.53 37.24 36.94 21.69
^{13}C NMR (101 MHz, CDCl$_3$)
NBnTs
PMP
CF$_3$
MeO
13
180 170 160 150 140 130 120 110 100 90 80 70 60 50 40 30 20 10
f1 (ppm)

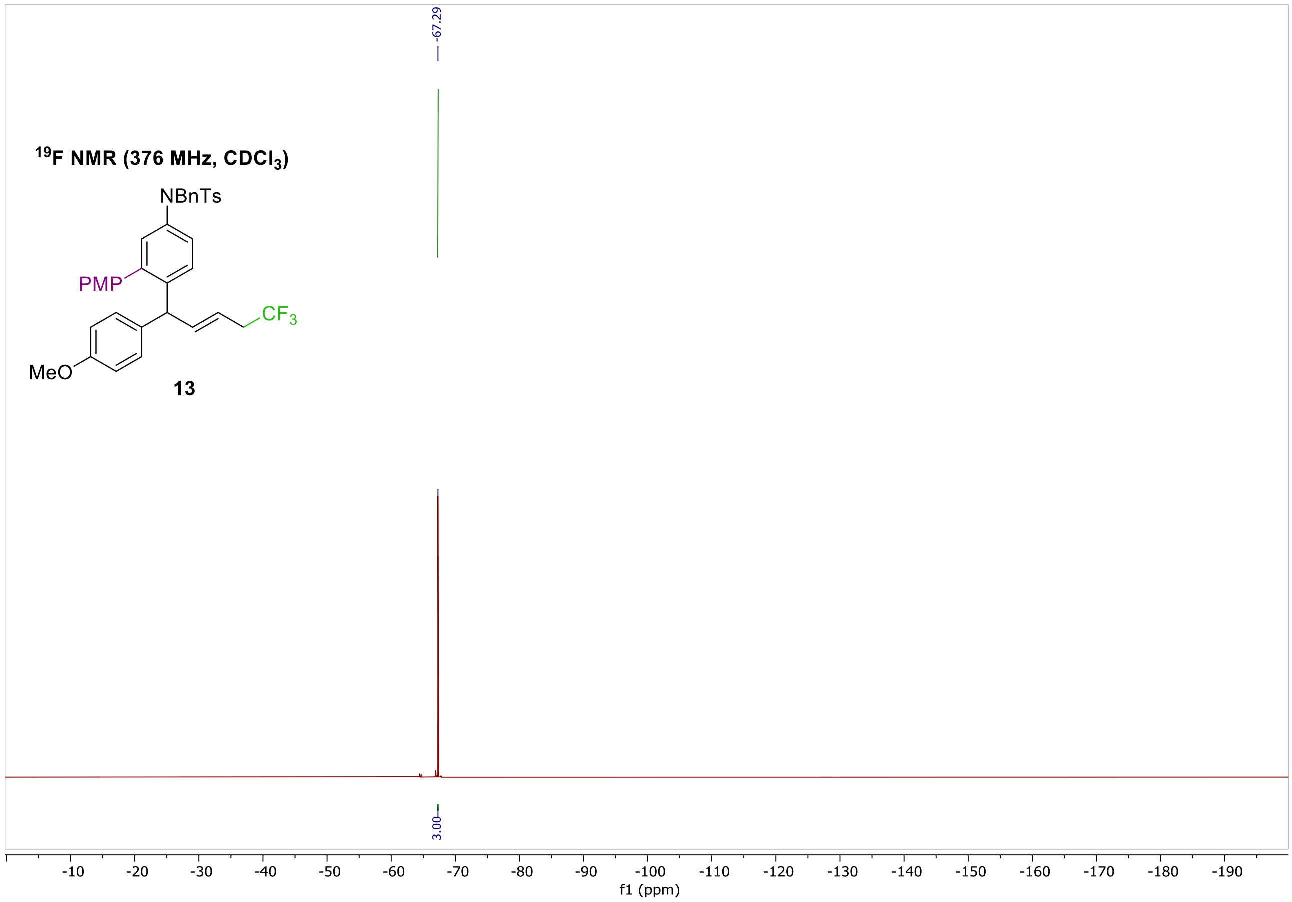
-67.29
19F NMR (376 MHz, CDCl3)
NBnTs
PMP
CF3
MeO
13
3.00
f1 (ppm)

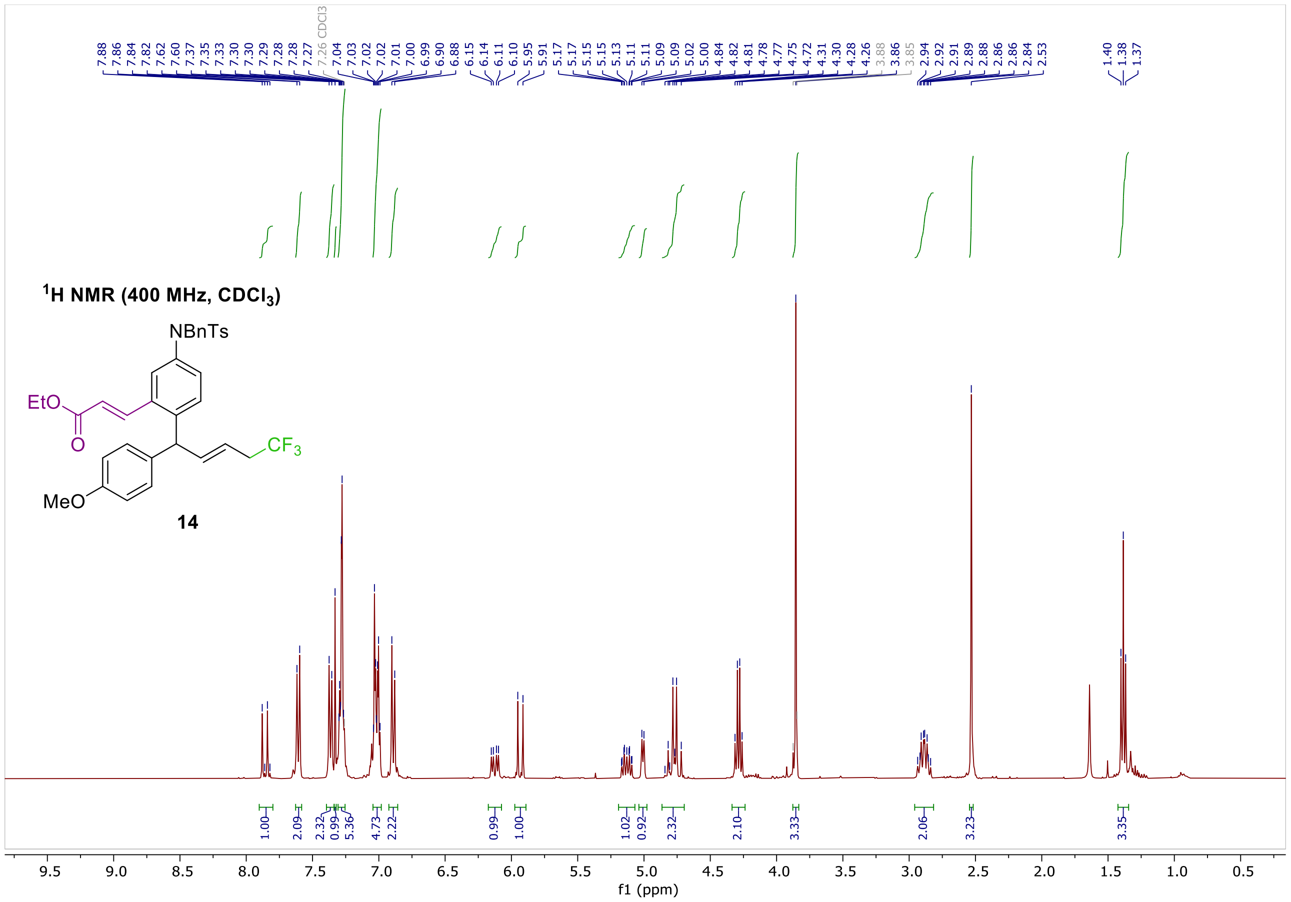
1H NMR (400 MHz, CDCl3)
NBnTs
EtO
O
CF3
MeO
14
f1 (ppm)

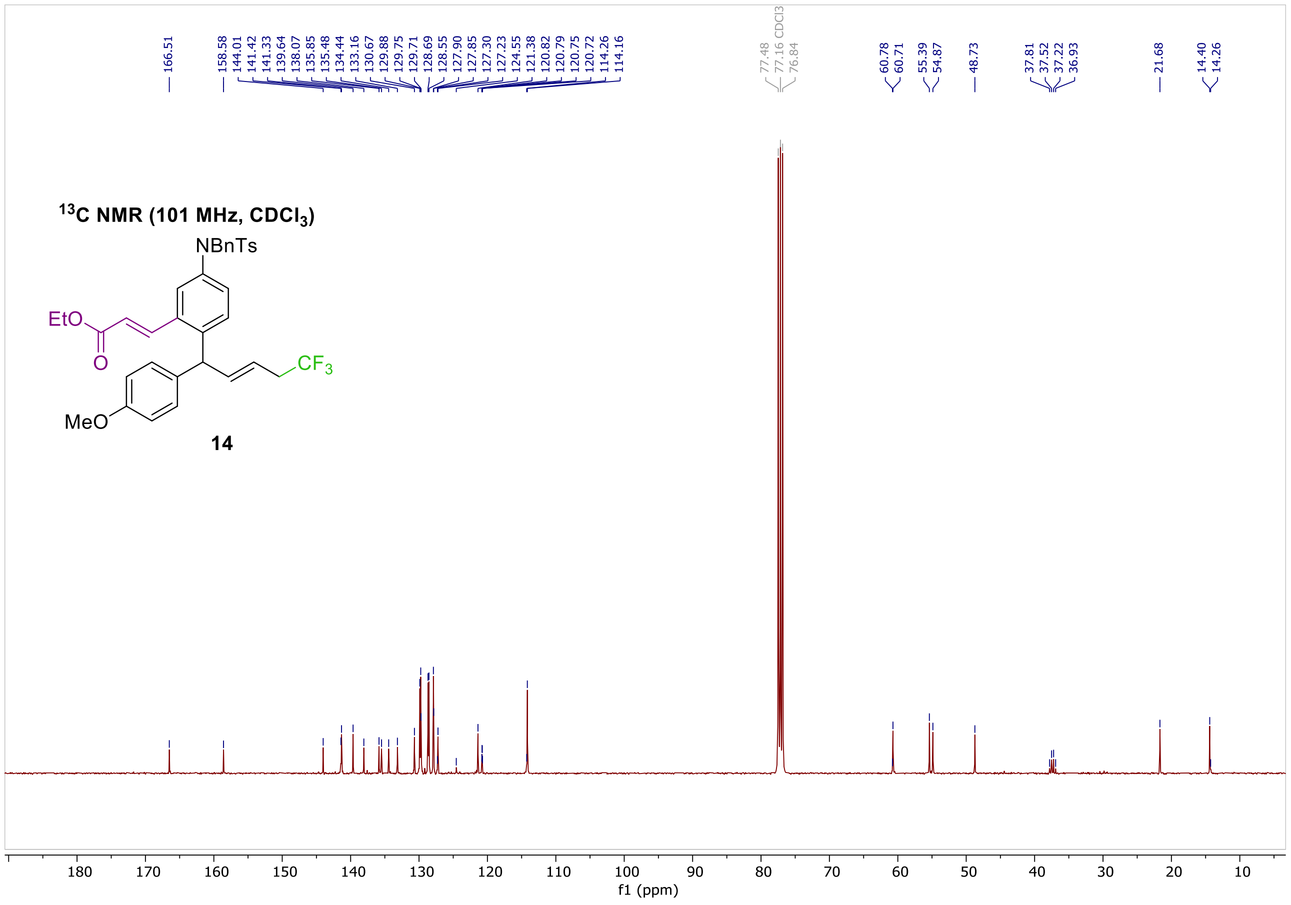

13C NMR (101 MHz, CDCl3)
NBnTs
EtO
O
CF3
MeO
14
f1 (ppm)

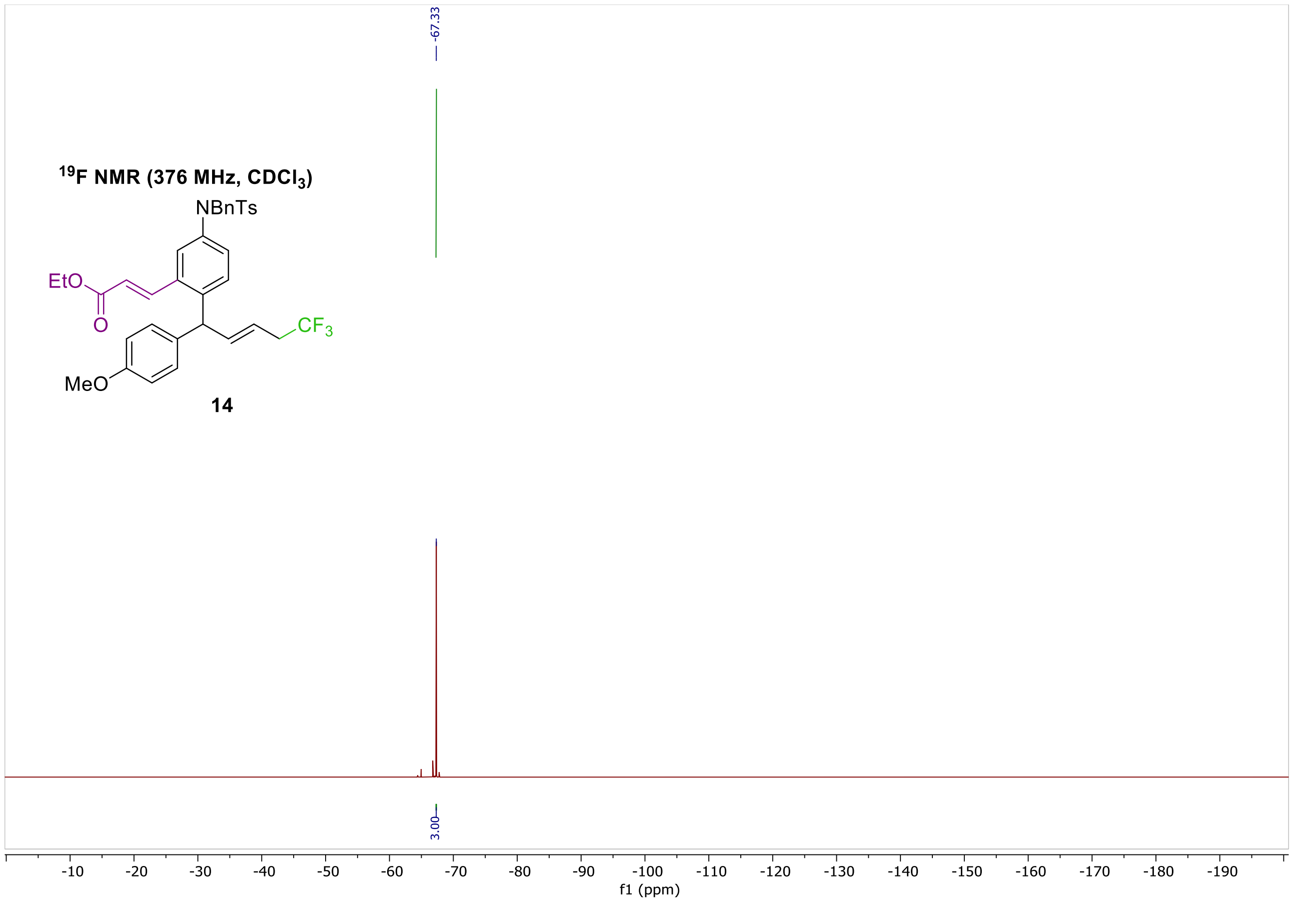

19F NMR (376 MHz, CDCl3)
NBnTs
EtO
O
CF3
MeO
14
-67.33
3.00
f1 (ppm)

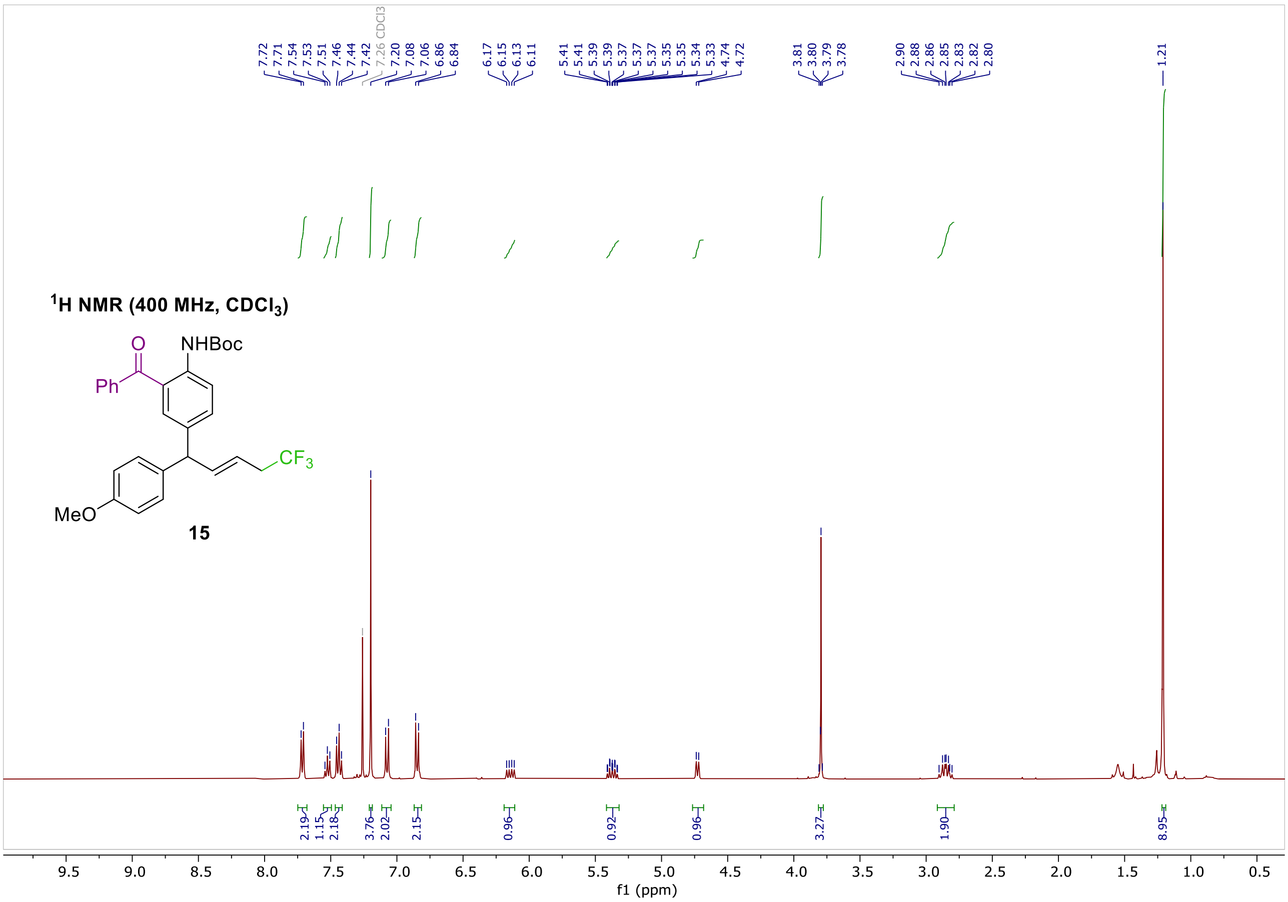

7.72 7.71 7.54 7.53 7.51 7.46 7.44 7.42 7.26 CDCl3 7.20 7.08 7.06 6.86 6.84 6.17 6.15 6.13 6.11 5.41 5.41 5.39 5.39 5.37 5.37 5.37 5.35 5.35 5.34 5.33 4.74 4.72 3.81 3.80 3.79 3.78 2.90 2.88 2.86 2.85 2.83 2.82 2.80 1.21
1H NMR (400 MHz, CDCl3)
O
NHBoc
Ph
CF3
MeO
15
2.19 1.15 2.18 3.76 2.02 2.15 0.96 0.92 0.96 3.27 1.90 8.95
f1 (ppm)

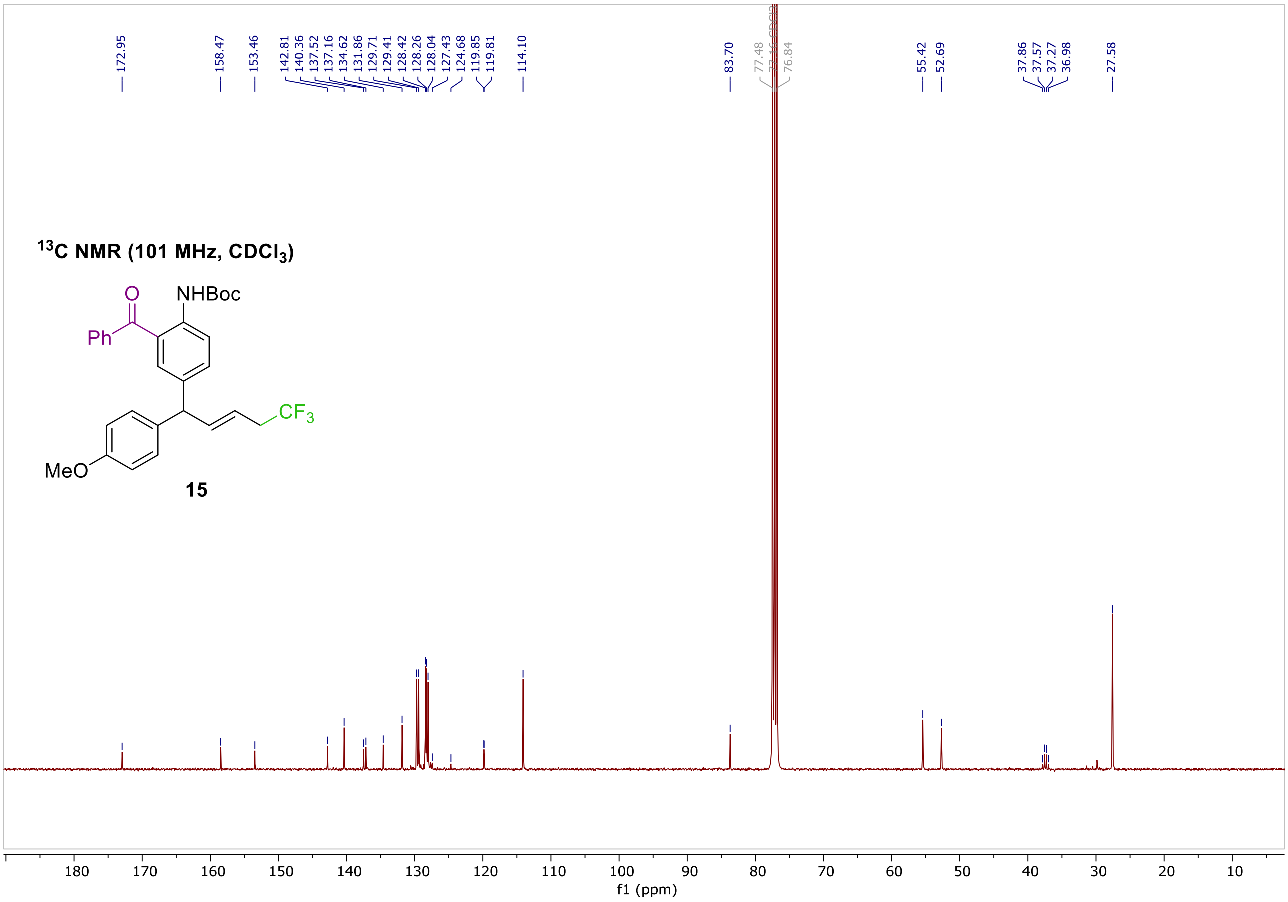

172.95 158.47 153.46 142.81 140.36 137.52 137.16 134.62 131.86 129.71 129.41 128.42 128.26 128.04 127.43 124.68 119.85 119.81 114.10 83.70 77.48 76.84 55.42 52.69 37.86 37.57 37.27 36.98 27.58
13C NMR (101 MHz, CDCl3)
O
NHBoc
Ph
CF3
MeO
15
f1 (ppm)

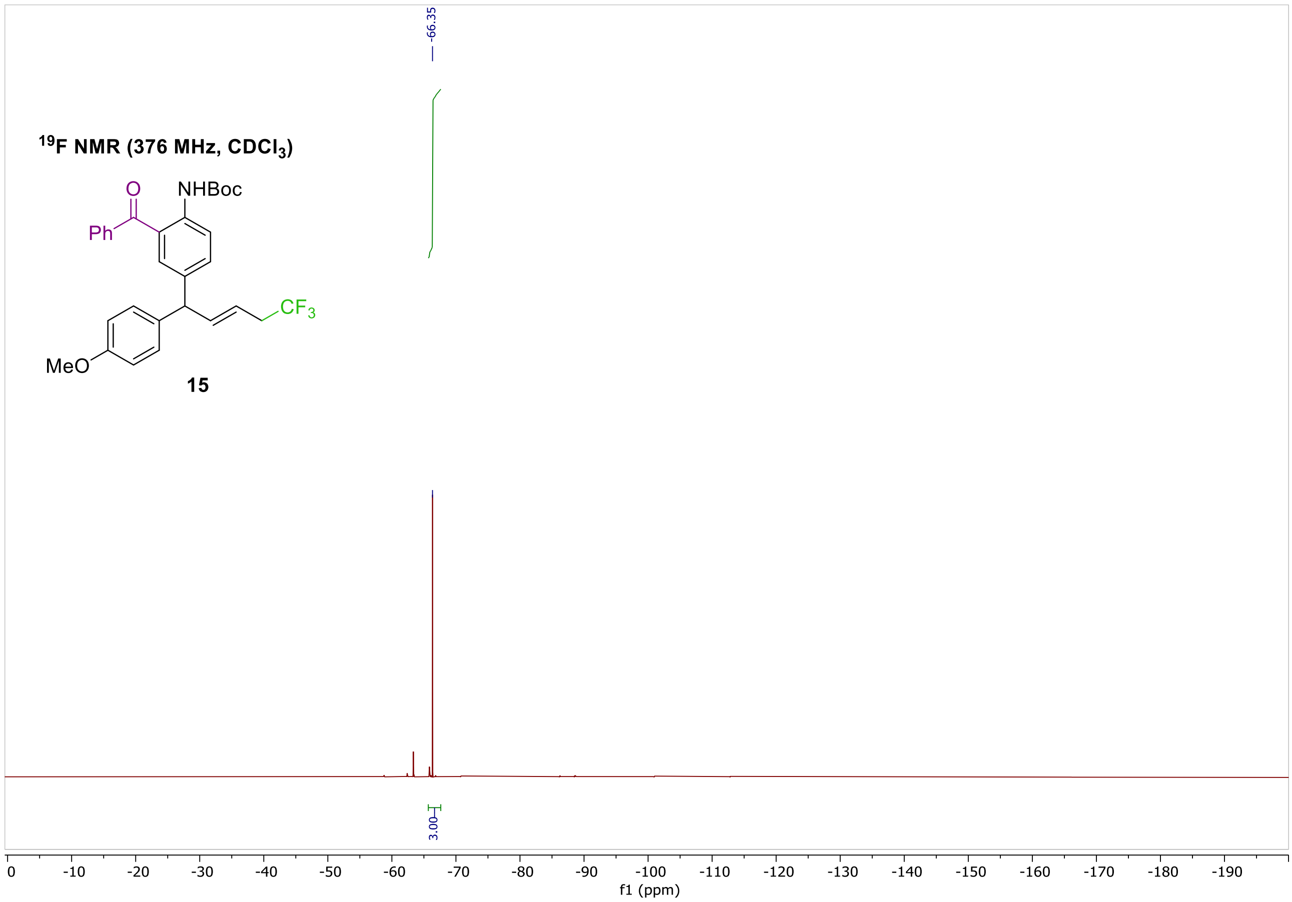
19F NMR (376 MHz, CDCl3)
NHBoc
O
Ph
CF3
MeO
15
-66.35
3.00
f1 (ppm)

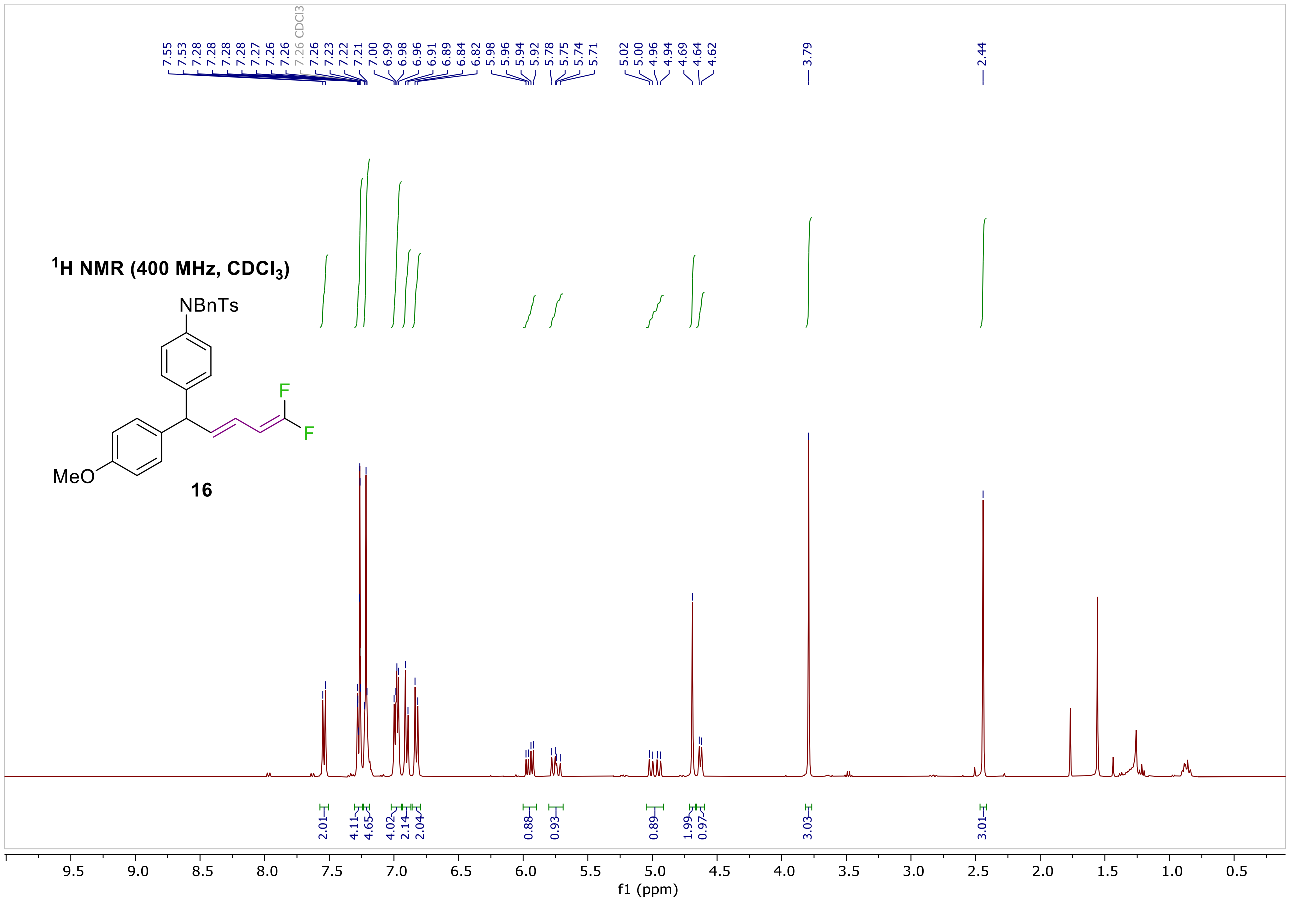
1H NMR (400 MHz, CDCl3)
NBnTs
F
F
MeO
16
3.79
2.44
f1 (ppm)

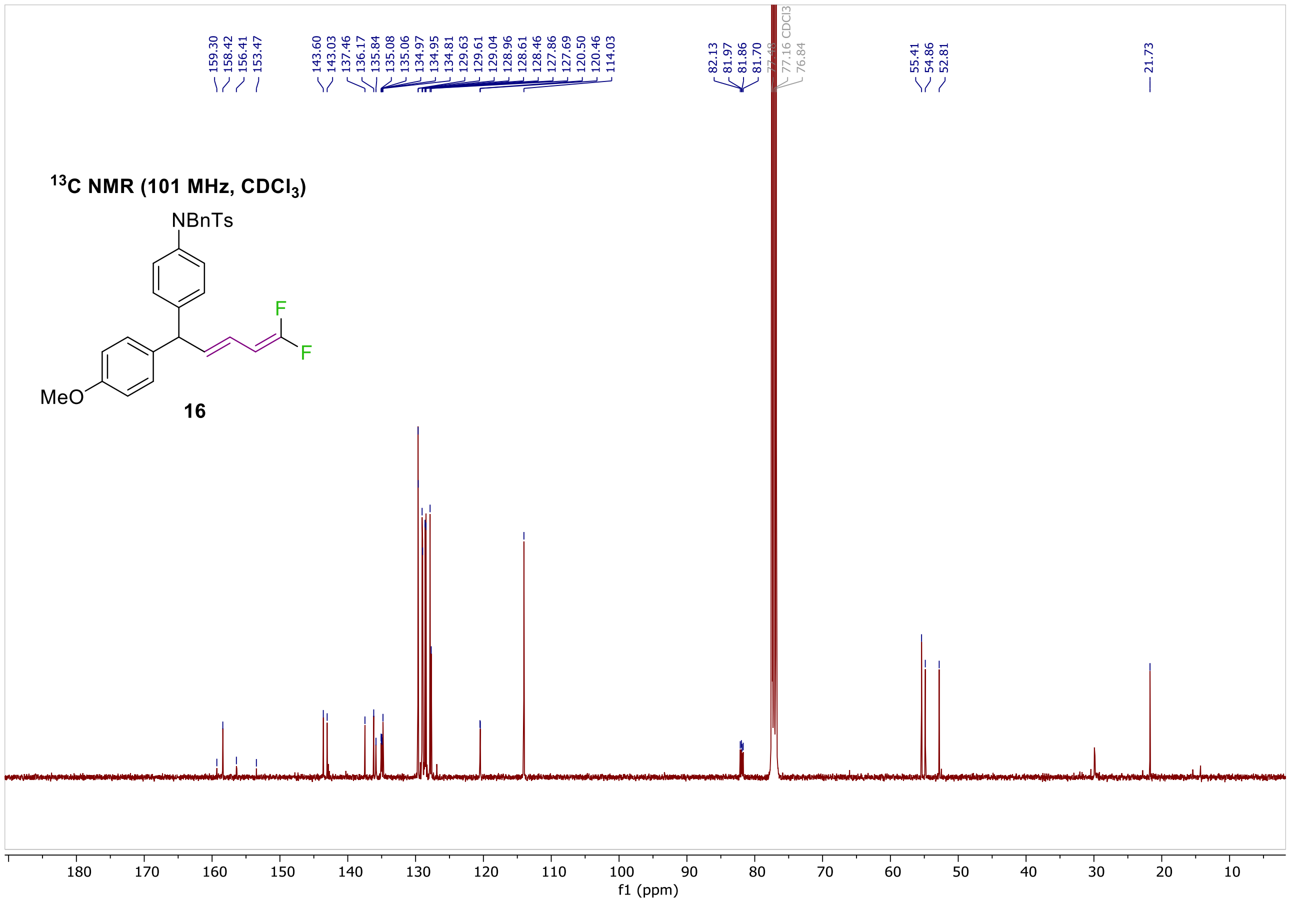

159.30
158.42
156.41
153.47
143.60
143.03
137.46
136.17
135.84
135.08
135.06
134.97
134.95
134.81
129.63
129.61
129.04
128.96
128.61
128.46
127.86
127.69
120.50
120.46
114.03
82.13
81.97
81.86
81.70
77.16 CDCl3
76.84
55.41
54.86
52.81
21.73
13C NMR (101 MHz, CDCl3)
NBnTs
F
F
MeO
16
f1 (ppm)

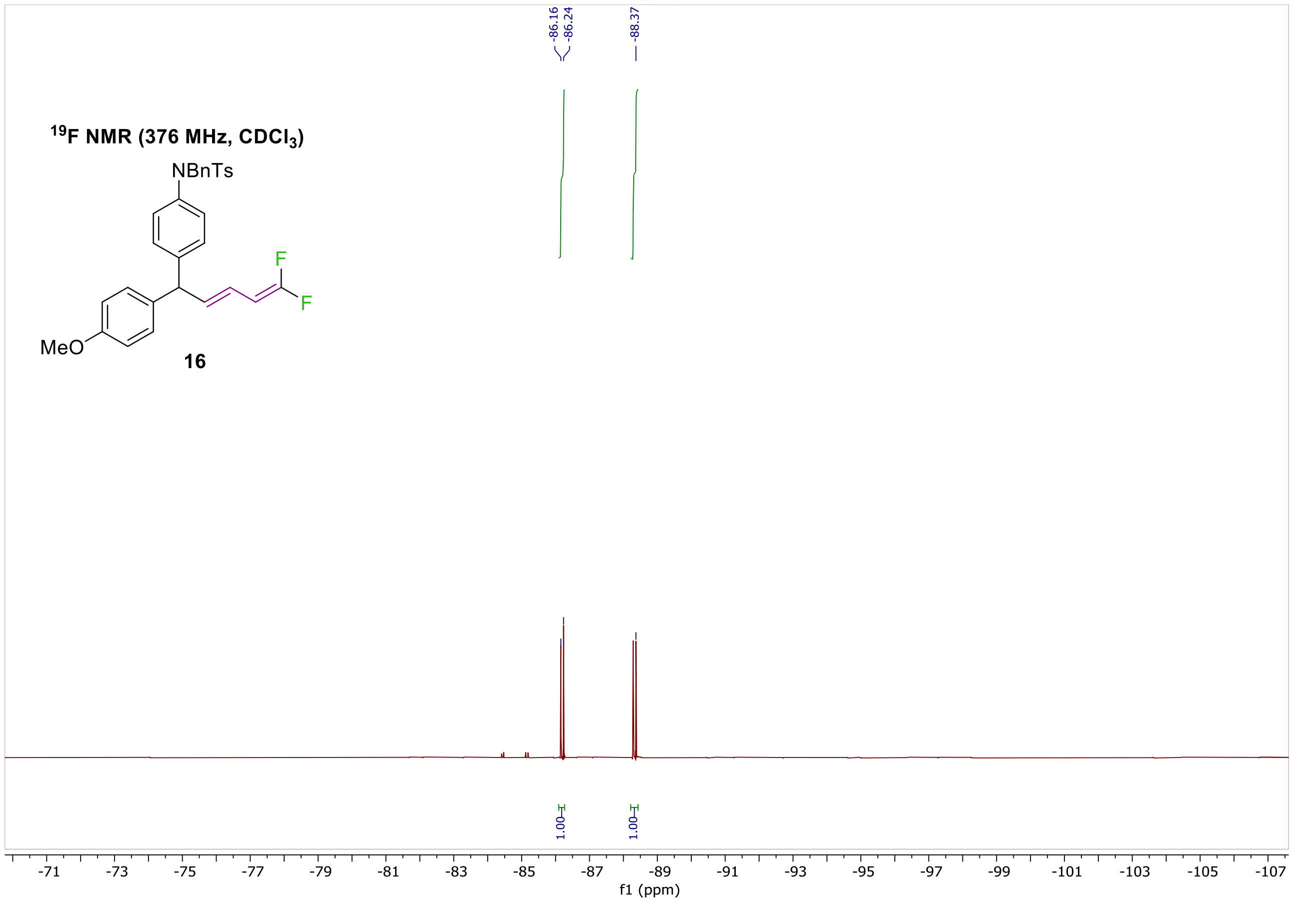

-86.16
-86.24
-88.37
19F NMR (376 MHz, CDCl3)
NBnTs
F
F
MeO
16
1.00
1.00
f1 (ppm)

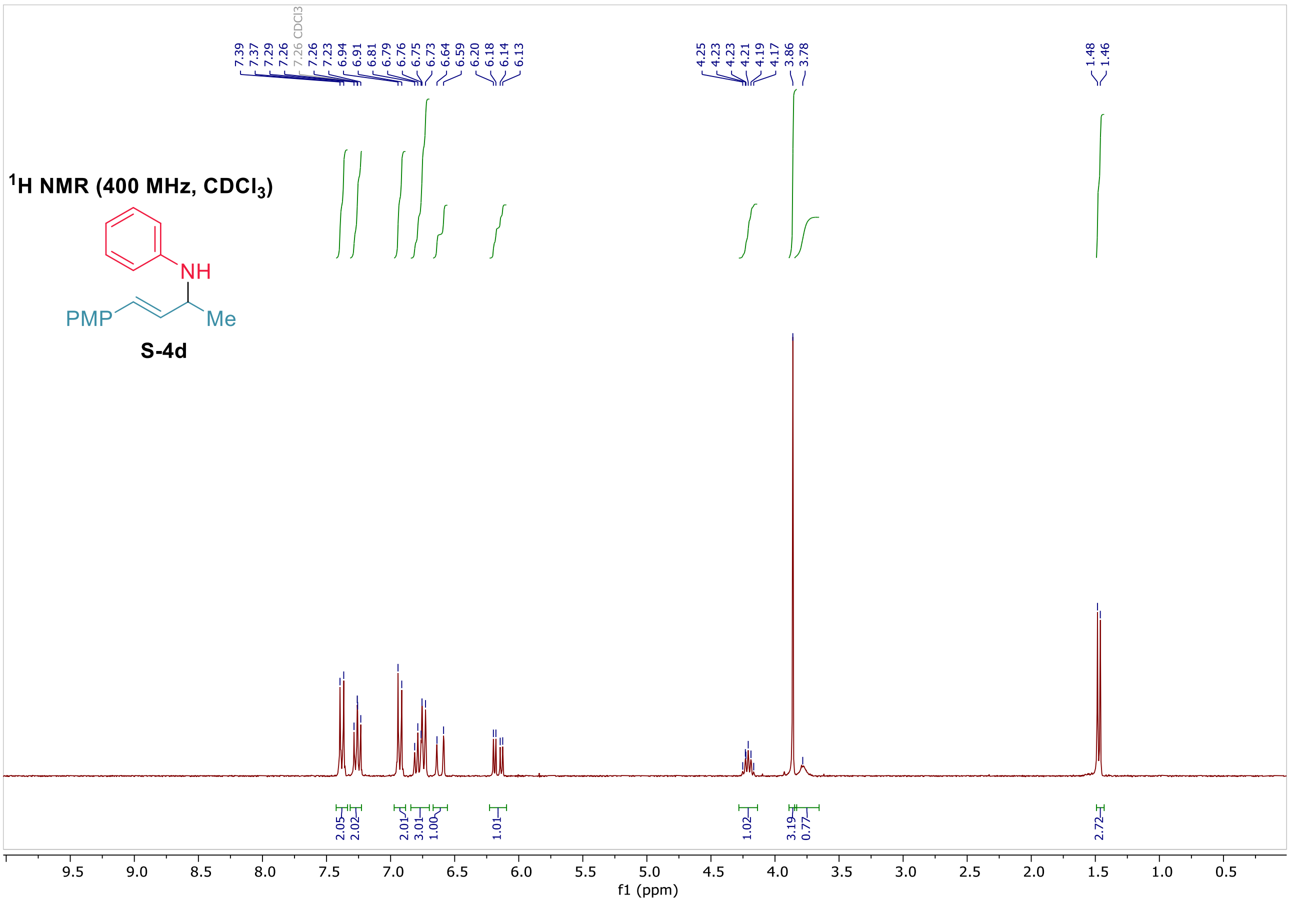
1H NMR (400 MHz, CDCl3)
NH
PMP
Me
S-4d
7.39 7.37 7.29 7.26 7.26 CDCl3 7.26 7.23 6.94 6.91 6.81 6.79 6.76 6.75 6.73 6.64 6.59 6.20 6.18 6.14 6.13
4.25 4.23 4.23 4.21 4.19 4.17 3.86 3.78
1.48 1.46
2.05 2.02 2.01 3.01 1.00 1.01 1.02 3.19 0.77 2.72
f1 (ppm)

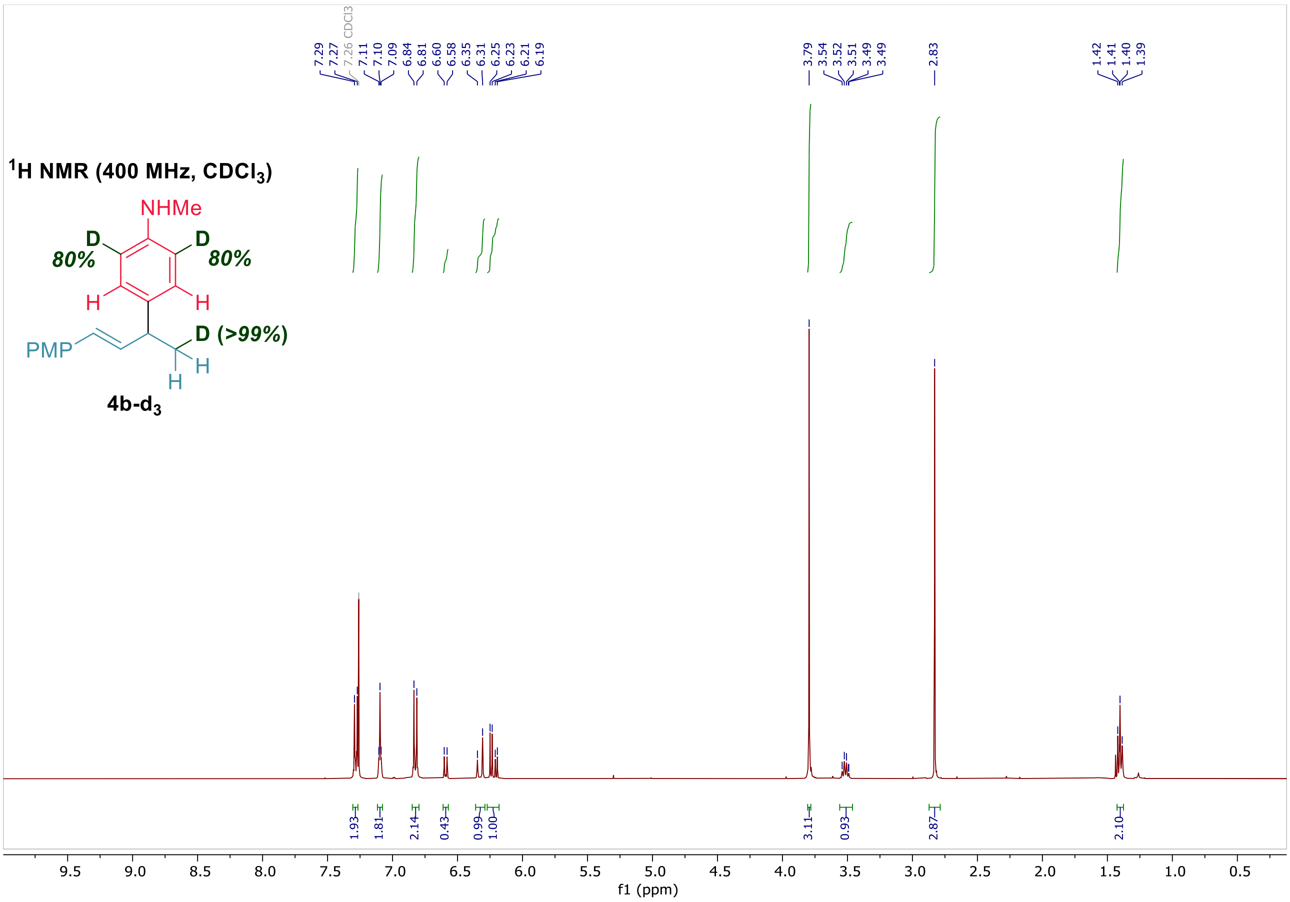
1H NMR (400 MHz, CDCl3)
NHMe
D 80%
D 80%
H
H
PMP
D (>99%)
H
H
4b-d3
7.29 7.27 7.26 CDCl3 7.11 7.10 7.09 6.84 6.81 6.60 6.58 6.35 6.31 6.25 6.23 6.21 6.19
3.79 3.54 3.52 3.51 3.49 3.49 2.83
1.42 1.41 1.40 1.39
1.93 1.81 2.14 0.43 0.99 1.00 3.11 0.93 2.87 2.10
f1 (ppm)

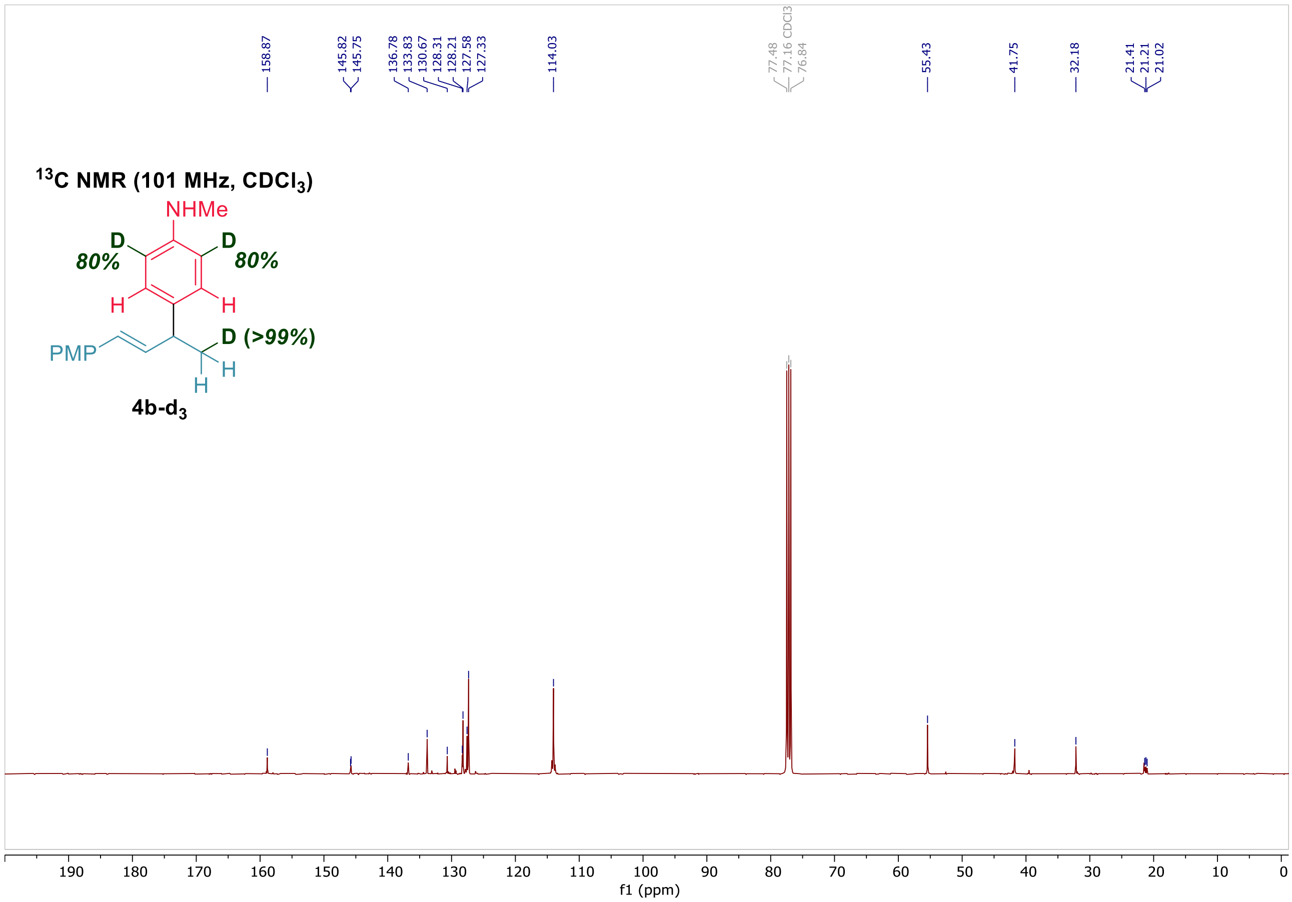
158.87
145.82
145.75
136.78
133.83
130.67
128.31
128.21
127.58
127.33
114.03
77.48
77.16 CDCl3
76.84
55.43
41.75
32.18
21.41
21.21
21.02
13C NMR (101 MHz, CDCl3)
NHMe
D
80%
D
80%
H
H
D (>99%)
PMP
H
H
4b-d3
f1 (ppm)

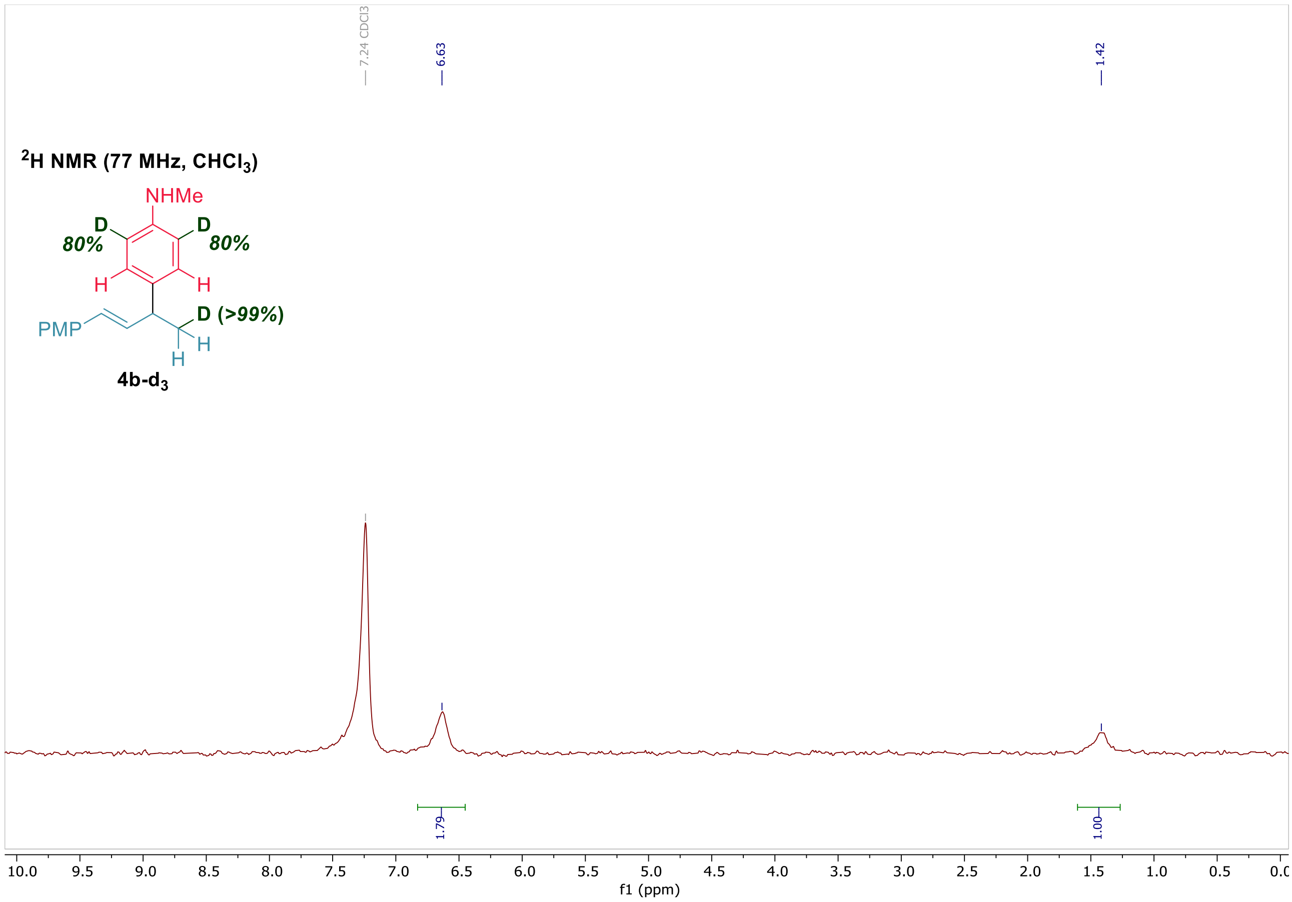
7.24 CDCl3
6.63
1.42
2H NMR (77 MHz, CHCl3)
NHMe
D
80%
D
80%
H
H
D (>99%)
PMP
H
H
4b-d3
1.79
1.00
f1 (ppm)

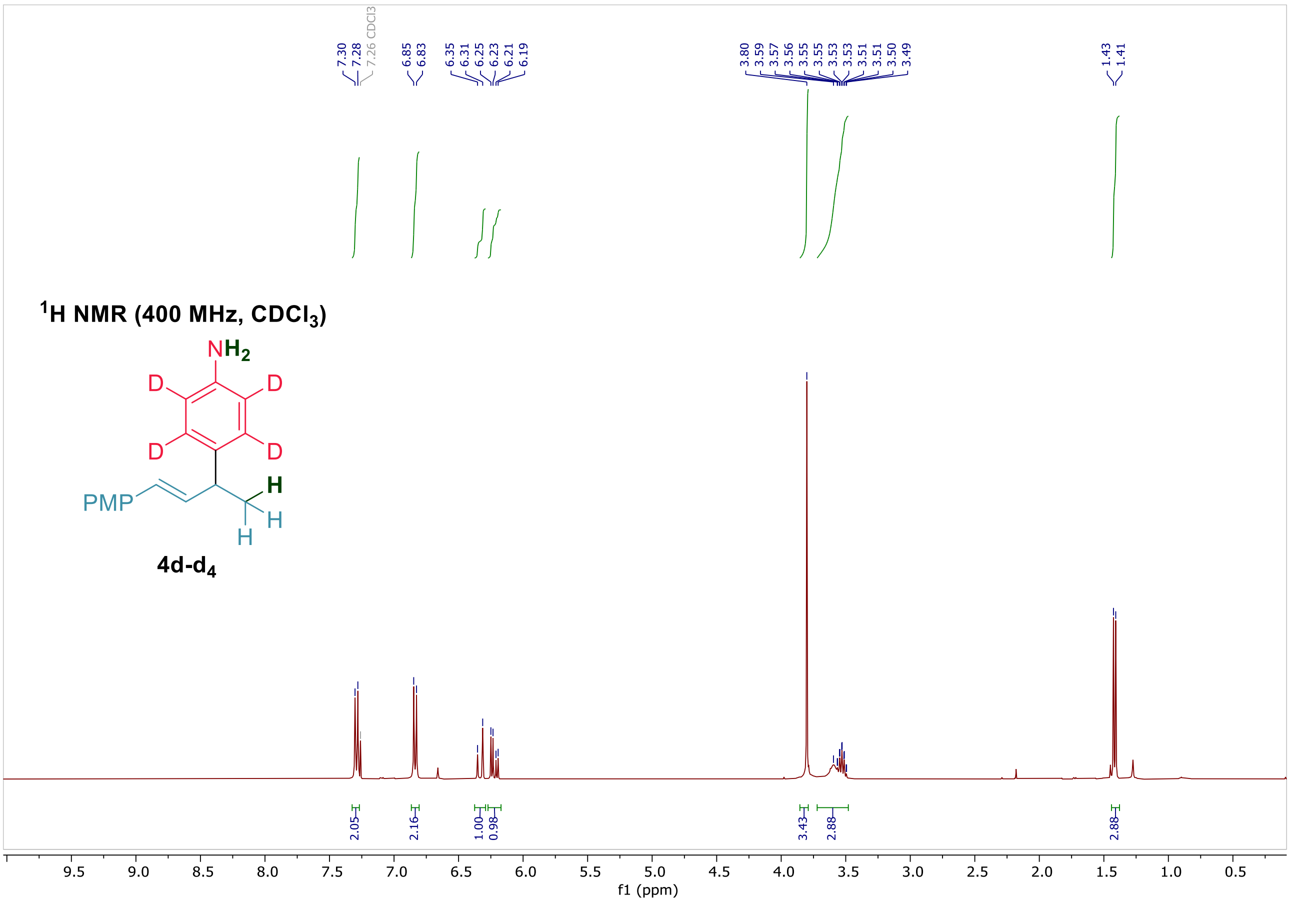

1H NMR (400 MHz, CDCl3)
4d-d4
PMP
NH2
7.30
7.28
7.26 CDCl3
6.85
6.83
6.35
6.31
6.25
6.23
6.21
6.19
3.80
3.59
3.57
3.56
3.55
3.55
3.53
3.53
3.51
3.51
3.50
3.49
1.43
1.41
2.05
2.16
1.00
0.98
3.43
2.88
2.88
f1 (ppm)

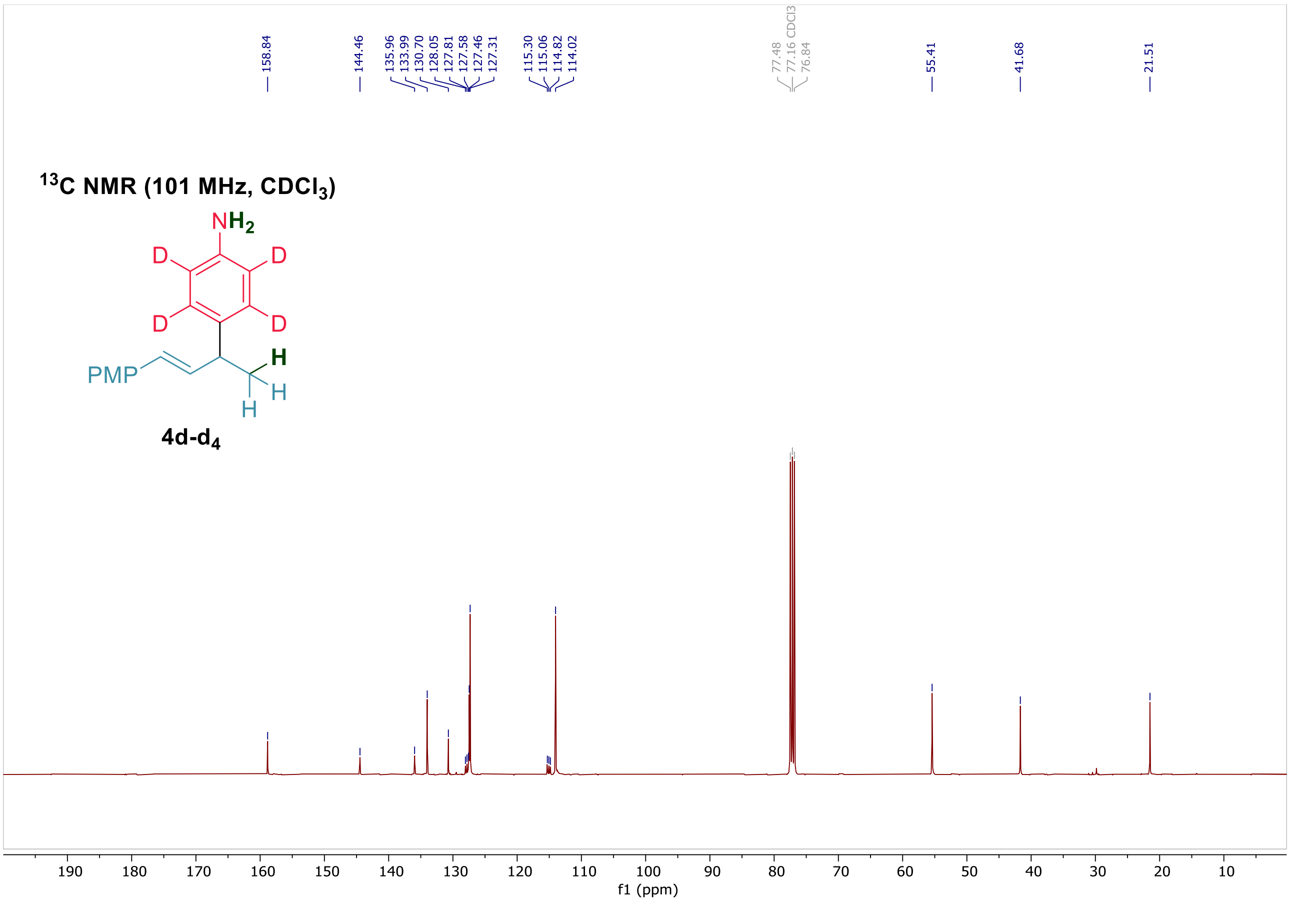

13C NMR (101 MHz, CDCl3)
4d-d4
PMP
NH2
158.84
144.46
135.96
133.99
130.70
128.05
127.81
127.58
127.46
127.31
115.30
115.06
114.82
114.02
77.48
77.16 CDCl3
76.84
55.41
41.68
21.51
f1 (ppm)

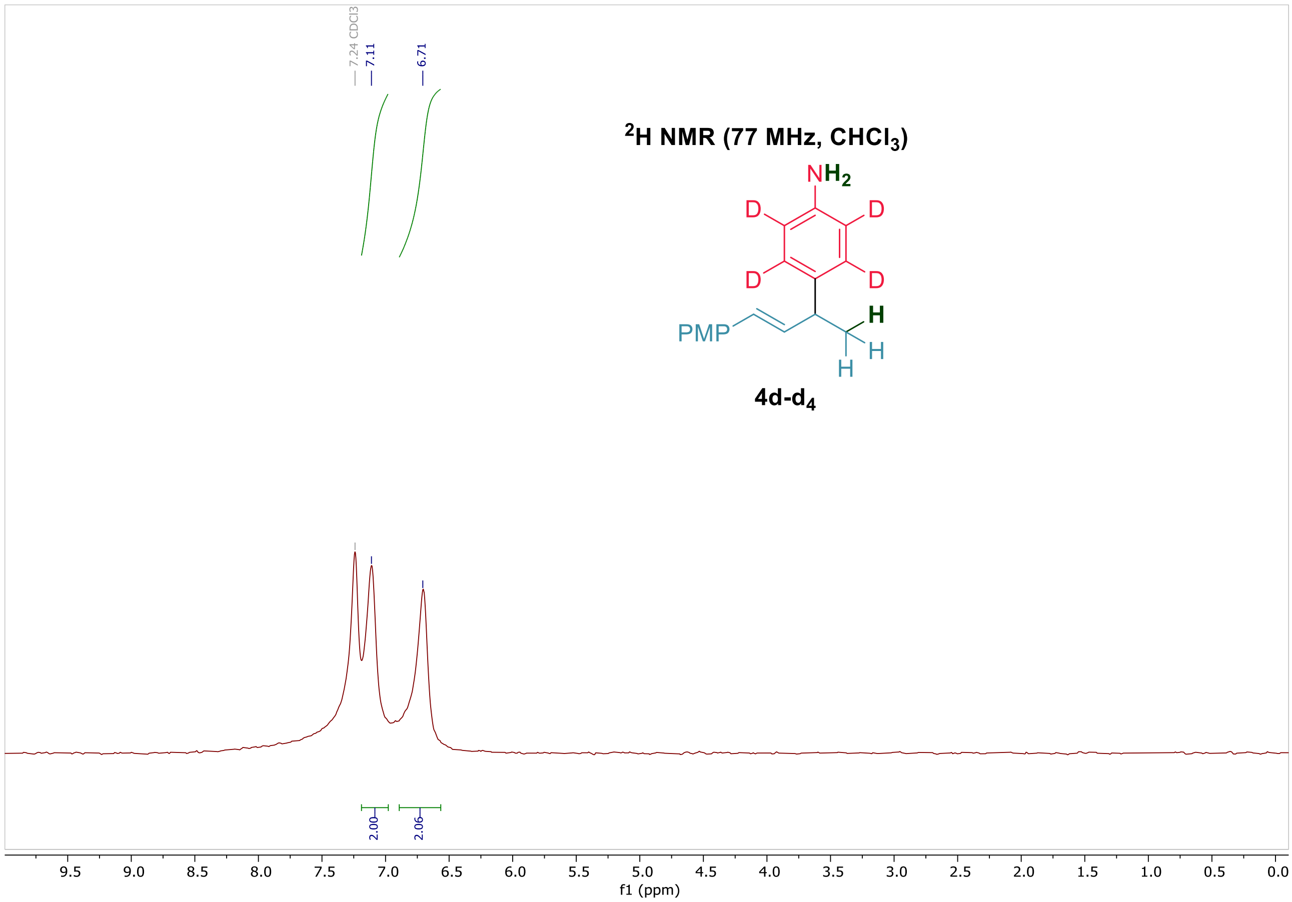

7.24 CDCl3
7.11
6.71
^{2}H NMR (77 MHz, CHCl$_3$)
NH$_2$
D
D
D
D
PMP
H
H
H
4d-d$_4$
2.00
2.06
9.5 9.0 8.5 8.0 7.5 7.0 6.5 6.0 5.5 5.0 4.5 4.0 3.5 3.0 2.5 2.0 1.5 1.0 0.5 0.0
f1 (ppm)
